# A review of the global epidemiology of scrub typhus

**DOI:** 10.1371/journal.pntd.0006062

**Published:** 2017-11-03

**Authors:** Guang Xu, David H. Walker, Daniel Jupiter, Peter C. Melby, Christine M. Arcari

**Affiliations:** 1 Department of Pathology, The University of Texas Medical Branch, Galveston, Texas, United States of America; 2 Department of Preventive Medicine and Community Health, The University of Texas Medical Branch, Galveston, Texas, United States of America; 3 Department of Internal Medicine, Division of Infectious Diseases, The University of Texas Medical Branch, Galveston, Texas, United States of America; Mahidol University, THAILAND

## Abstract

Scrub typhus is a serious public health problem in the Asia-Pacific area. It threatens one billion people globally, and causes illness in one million people each year. Caused by *Orientia tsutsugamushi*, scrub typhus can result in severe multiorgan failure with a case fatality rate up to 70% without appropriate treatment. The antigenic heterogeneity of *O*. *tsutsugamushi* precludes generic immunity and allows reinfection. As a neglected disease, there is still a large gap in our knowledge of the disease, as evidenced by the sporadic epidemiologic data and other related public health information regarding scrub typhus in its endemic areas. Our objective is to provide a systematic analysis of current epidemiology, prevention and control of scrub typhus in its long-standing endemic areas and recently recognized foci of infection.

## Author summary

Scrub typhus is a serious public health problem in the Asia-Pacific area. There is an estimated one million new scrub typhus infections each year, and over one billion people around the world are at risk. Without appropriate treatment, the case fatality rate of scrub typhus can reach 30% or even higher. Scrub typhus has long been a neglected infectious disease so many aspects of the disease, including its diagnosis to prevention, are unknown. We here provide a comprehensive review of the epidemiology, prevention and control of scrub typhus.

## Introduction

**Fig 1:**
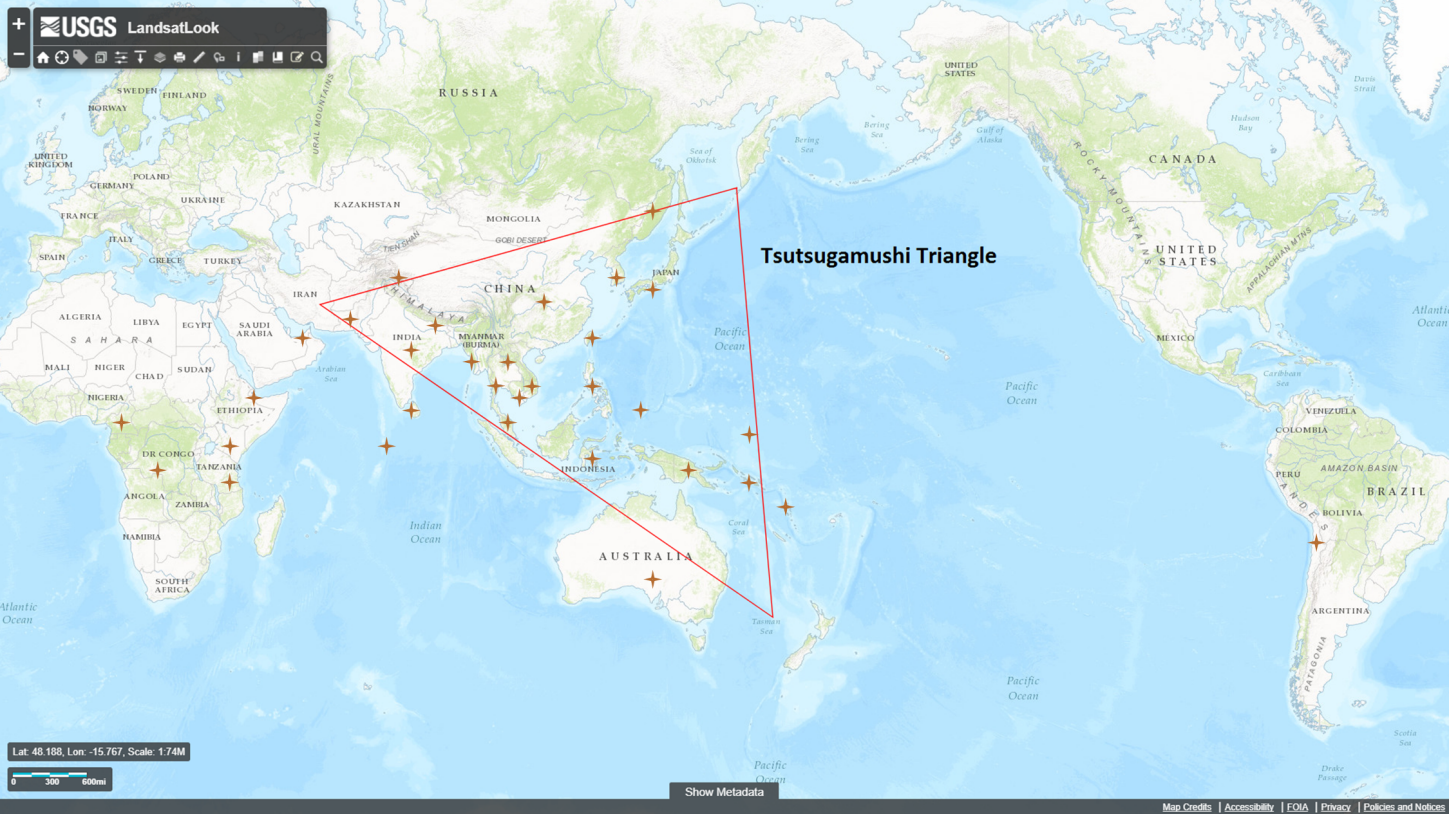
Worldwide map of countries with reported scrub typhus cases. The majority of scrub typhus cases occur in the “tsutsugamushi triangle” in the Asia-Pacific area. Countries with human cases are labeled with a star. [Modified from https://landsatlook.usgs.gov/viewer.html].

Scrub typhus is a serious public health problem in the Asia-Pacific area including, but not limited to, Korea, Japan, China, Taiwan, India, Indonesia, Thailand, Sri Lanka, and the Philippines (**Fig 1**). It threatens one billion people globally, and causes illness in one million people each year [1]. Scrub typhus, also known as tsutsugamushi disease, is caused by the arthropod-borne gram-negative obligately intracellular bacillus *Orientia tsutsugamushi* [2,3,4]. Approximately 5 to 14 days after being bitten by an infected vector, a *Leptotrombidium* mite, patients begin to exhibit manifestations of infection such as non-specific flu-like symptoms, fever, rash, eschar at the bite site, headache, myalgia, cough, generalized lymphadenopathy, nausea, vomiting, and abdominal pain [5,6,7]. Fever and headache are the most common features among scrub typhus patients. Between 95% and 100% of confirmed cases were noted to have fever in several studies [8,9].

An eschar at the site of chigger feeding is a classic clinical feature of scrub typhus. It begins as a papule at the site of chigger feeding and then ulcerates and forms a black crust like a skin burn from a cigarette. When present, it occurs prior to the onset of fever and other symptoms [6,10]. The presentation of eschar varies from 1%–97% of scrub typhus patients depending on the geographic areas and studies [8,11,12]. It is more easily found on Caucasian and East Asian patients than on dark skinned South Asian patients [13]. Most eschars develop on the front of the body (~80%). In male patients, eschars are primarily within 30 cm below the umbilicus. The other common locations are lower extremities and anterior chest. There is a different pattern in female patients, whose anterior chest, head and neck are the most prevalent areas [13,14]. Eschars are also commonly present in the axillae of children in addition to the sites mentioned above [15,16].

Severe complications such as multiorgan failure occur in some cases. The severe multiorgan manifestations include jaundice, acute renal failure, pneumonitis, acute respiratory distress syndrome (ARDS), myocarditis, septic shock, meningoencephalitis, pericarditis, and disseminated intravascular coagulation (DIC) [6,7,9,17,18]. The lung is one of the main target organs for *Orientia*, leading to pulmonary complications of variable severity. Interstitial pneumonia may occur in severe cases [7,19]. Meningitis and/or encephalitis can develop in severe illness, causing patients to become agitated, delirious or even have seizures. Focal neurological signs are rare but have been known to occur. Laboratory tests may demonstrate changes in cerebrospinal fluid similar to those found in viral or tuberculous meningitis [20,21,22,23]. Patients may also develop the complications of hearing loss or hearing impairment during scrub typhus infection [24,25]. The case fatality rate of scrub typhus varies among different countries, regions, and areas as well as different studies [26]. The case fatality can be up to 30–70% if no appropriate treatment is received while the median case fatality rate for untreated patients is 6% and for treated patient is 1.4% [26,27,28]. Therefore, development of effective measures to treat, control and prevent the disease is a critical public health issue.

Other signs can be observed in scrub typhus patients. Marked hyperemia and even hemorrhage can be found on the conjunctiva during the acute phase of the disease. There are reports of hemorrhages and coagulation disorders, mostly gastrointestinal complications, among scrub typhus patients. Severely ill patients can suffer gastrointestinal mucosal hemorrhage, multiple erosions and ulcers. [6,23,29].

*Orientia tsutsugamushi* is transmitted to mammalian hosts including humans by the larval stage of *Leptotrombidium* mites, also called chiggers [30]. Mites act as the primary reservoirs for *O*. *tsutsugamushi*. They remain infected through their life cycle (larva, nymph, adult and egg) [7]. It is thought that larvae of mites only feed once on a mammal host. Chiggers usually feed on thin, tender or wrinkled skin. The feeding lasts 2 to 4 days [31]. It has been shown that chiggers do not pierce the host skin but rather take advantage of hair follicles or pores [6]. The saliva that the mites secrete can dissolve host tissue around the feeding site, and the mites ingest the liquefied tissue. *Orientia tsutsugamushi* has been found in the salivary glands of mites [6]. Transovarial and transstadial transmission are the main mechanisms for maintaining *Orientia* in the mite, and there are a limited number of reports that the bacteria can also be transmitted to mites during co-feeding and/or from wild rodents [32,33,34,35]. Transstadial transmission is maintenance of the pathogen in the vector from one life stage to the next, i.e., passage of *O*. *tsutsugamushi* from mite larva to nymph, and nymph to adult. Transovarial transmission is the process of passing *O*. *tsutsugamushi* from the female to offspring through eggs [12,35]. Both methods of transmission are involved in vertical transmission. There have been rare documented occurrences of horizontal transmission of *Orientia* among mites [36]. During horizontal transmission, a chigger acquires *Orientia* from an infected host, and its offspring infects a new host. Not enough evidence exists to demonstrate that such horizontal transmission is an important means of maintenance of *O*. *tsutsugamushi* in nature [34,36,37,38]. There are no reports of person-to-person transmission of scrub typhus [7]. Further studies on mites and their engorgement on the host can facilitate the control and prevention of scrub typhus.

There are still many unknowns regarding the mechanisms of pathogenesis and the cell biology of the interaction of this bacterium with its host cell, due to the extra research obstacles of studying an obligately intracellular bacterium [39]. The antigenic heterogeneity of, reemergence of, and short-lived immunity to *Orientia* result in a substantial number of primary infections and reinfections. However, both basic research and epidemiological studies of *O*. *tsutsugamushi* have been largely neglected during recent decades [40,41,42]. Preliminary studies demonstrated that the disease has existed in the endemic areas for some time [43,44,45,46,47]. There are only sporadic epidemiological data available regarding scrub typhus in the endemic areas, as well as other parts of the world, resulting in a current gap in knowledge.

We provide here a systematic analysis of current epidemiology, prevention and control of scrub typhus in the endemic areas as well as among travelers from the rest of the world.

### Epidemiology of scrub typhus

The traditional endemic area of scrub typhus is known as the “tsutsugamushi triangle”. It is a region covering more than 8 million km^2^, from the Russian Far East in the north, to Pakistan in the west, Australia in the south, and the Japan in the east [23,48,49]. There are one billion people at risk of infection; the endemic area is highly populated [1]. The progress of globalization and associated travel contributes to the exportation of the infected persons to non-endemic areas [50]. The antigenic and genetic diversity of *O*. *tsutsugamushi* strains, and their unclear correlation with virulence for humans, confound the epidemiological study of scrub typhus [51]. Better understanding of the epidemiology of scrub typhus will help efforts to prevent and control the disease. This part of the article describes studies of the geographic distribution and risk factors of scrub typhus in both the endemic areas and in travelers from the rest of the world.

## Methods

A systematic literature review of epidemiological studies and case reports of scrub typhus was carried out using the methods of Hemingway et al. and the Preferred Reporting Items for Systematic Reviews and Meta-Analysis (PRISMA) statement (**S1 Table**) [52,53].

We first defined the research question as the epidemiology of scrub typhus. Literature was searched in the PubMed and Google Scholar databases. The following search terms were used: (scrub typhus) AND (epidemiology OR distribution OR case report). There was a total of 898 records identified, and 793 records were screened after removal of duplicates. Eighty-two records were further filtered out in PubMed due to being nonhuman studies. Titles and abstracts were used to assess the eligibility of each study. Only English language peer-reviewed articles were included in the study. A number of publications in Chinese, Japanese, Korean, Russian, and other languages were not included in the analysis. Two hundred and forty-two records were excluded due to non-English language and non-human studies. Among the 711 records that remained, 647 records were either simple case reports for which the abstracts provided complete case information, or only abstracts were available during analysis. Sixty-four full-text articles were obtained and assessed. Eight articles which reported imported cases in Belgium, France, Germany, Italy, the Netherlands, New Zealand, Scandinavia, and the U.S. were excluded [54,55,56,57,58,59,60,61,62]. Fifty-six articles were included in this qualitative synthesis and analysis.

We summarized lists of countries or regions with human case reports or other epidemiological studies of scrub typhus in tables. For those countries or areas having long history of endemic transmission, or who which multiple studies were reported, we further did a literature review using Pubmed and Google Scholar to retrieve detailed epidemiological characteristics of the specific country or region, such as Japan, China, South Korea, Thailand, and India. To make the results easier to understand, we have prepared a list of seroprevalence of scrub typhus by county, and the outbreaks of scrub typhus, in separate tables.

### Transmission of scrub typhus

Even with its recent re-emergence in the traditionally endemic areas and worldwide, scrub typhus is still a neglected infectious disease [6,63,64]. The geographic distribution of scrub typhus is determined by the distribution of its vector and reservoir—mites, primarily of the genus *Leptotrombidium*. Humans are accidental hosts [1,51].

Outdoor workers, especially field workers in rural areas, have a higher risk of acquiring the disease [65]. It is reported that rice fields are an under-appreciated location where the biting of mites and transmission of *O*. *tsutsugamushi* occurs in the endemic areas [63]. Tropical weather provides stable and ideal conditions for transmission of the disease. High temperature and high humidity are optimal for mite activity. In more temperate climates, the transmission of scrub typhus is more seasonal due to the temporal activity of chiggers [45,63,66].

Scrub typhus has been a nationally notifiable disease in Bhutan, China, Japan, South Korea, Thailand, and Taiwan [67,68,69,70,71,72]. The reported seroprevalence of *O*. *tsutsugamushi* for each country is shown in **Table 1** [73,74,75,76,77,78,79,80,81,82,83,84,85,86,87,88,89,90,91,92,93,94,95,96,97,98,99,100,101,102,103,104,105,106]. The reports of outbreaks of scrub typhus are summarized in **Table 2**[43,94,107,108,109,110,111,112,113,114,115,116,117,118,119,120,121,122,123,124,125,126,127,128,129,130,131,132,133,134,135,136,137]. The list of other countries with published cases of scrub typhus is found in **S2 Table** [80,136,138,139,140,141,142,143,144,145,146,147,148,149,150,151,152,153,154]. All countries with reported cases are designated in **Fig 1**.

**Table 1:** Global seroprevalence of scrub typhus by Region/Country.

Region and Country / Reported by	Collection Year	Tested Number	Tested Population	Sero-prevalence
***Australia***				
(Graves, Wang et al. 1999)	1996	920	General population	2.6%
***Bangladesh***				
(Maude, Maude et al. 2014)	2010	1,209	General population	23.7%
***China***				
(Horton, Jiang et al. 2016)	2009	100	General population	10%
***India***				
(Shivalli 2016)	Unknown	721	General population	31.8%
(Jakharia, Borkakoty et al. 2016)	Unknown	300	General population	40.0%
(Kalal, Puranik et al. 2016)	2010–2012	103	Hospitalized children with clinical features of rickettsial illness	60.2%
(Sengupta, Anandan et al. 2015)	2013	100	General population	15.0%
(Ramyasree, Kalawat et al. 2015)	2013	100	Clinically suspected cases	39.0%
(Sharma, Mahajan et al. 2005)	2003–2004	150	Patients with fever of unknown origin, diagnosed with the Weil-Felix test. PCR results were negative	34.7%
***Indonesia***				
(Richards, Ratiwayanto et al. 2003)	1997	53	Local villagers and mining employees enrolled in a study of in vivo resistance of malaria and lymphatic filariasis	9.4%
(Richards, Soeatmadji et al. 1997)	1994	464	General population	1.3%
***Japan***				
(Ogawa and Ono 2008)	1984–2005	561	General population	68.4%
***Kenya***				
(Thiga, Mutai et al. 2015)	Unknown	1,401	Patients with fever (>38°C)	4.8%
***Lao***				
(Vallee, Thaojaikong et al. 2010)	2006	2,001	Randomly selected adults (≥35 years) in urban and peri-urban Vientiane City	20.3%
***Malaysia***				
(Tay, Mohamed Zan et al. 2013)	2007–2010	280	General population	17.9%
(Tay, Kamalanathan et al. 2003)	1998–1999	240	Blood donors	5.4%
	1998–1999	292	Febrile patients	43.5%
(Sagin, Ismail et al. 2000)	N/A	261	General population	1.5%
(Tay, Ho et al. 2000)	1995–1997	1596	Febrile patients	24.9%
(Tee, Kamalanathan et al. 1999)	1996–1997	378	70 out of the 300 rubber estate workers in Dec (humid), and 31of 184 examined workers in Mar (dry, 106 were bled previously) were seropositive	16.8–23.3%
(Cadigan, Andre et al. 1972)		690	Blood from 245 adult (>20 yr) and 445 under 20 were collected from Orange Asli in central West Malaysia	0–73%
***Nepal***				
(Blacksell, Sharma et al. 2007)	2002–2004	103	Patients with fever (defined as axillary temperature >38°C).	22%
***Palau***				
(Demma, McQuiston et al. 2006)	2003	212	General population	47.6%
***Papua New Guinea***				
(Faa, Graves et al. 2006)	2002–2003	113	Pregnant patients during routine antenatal blood test	0.0%
(Kende and Graves 2003)	1997	191	Non-randomly residents living in Port Moresby (n = 93) and in the highland villages of Samberigi (n = 98)	1.6%
***South Korea***				
(Jang, Kim et al. 2004)	1992–1993	3,401	General population	35.2%
***Sri Lanka***				
(Premaratna, Ariyaratna et al. 2014)	2008	106	General population	38.7%
(Liyanapathirana and Thevanesam 2011)	2009–2010	615	General population	27.3%
***Thailand***				
(Bhengsri, Baggett et al. 2016)	2002–2005	2,225	General population	4.2%
(Blacksell, Tanganuchitcharnchai et al. 2015)	2006–2007	152	Hospitalized patients in a subset of a febrile illness study	28.3%
(Singhsilarak, Phongtananant et al. 2006)	N/A	194	Adult patients with falciparum malaria, who were enrolled in antimalarial drug trials	14.9%
(Chanyasanha, Kaeburong et al. 1998)	1994	200	Malaria patients at 6 malaria clinics	59.5%^15^
(Frances, Eamsila et al. 1997)	1991–1992	403	Royal Thai Army deployed to Thai-Cambodian border between September, 1991 and October, 1992	2.7%^16^
(Eamsila, Singsawat et al. 1996)	1989–1991	1,218–1,888	Soldiers from royal Thai Army, Border Patrol Police, and Thai Rangers	7.8–10.3%
(Strickman, Tanskul et al. 1994)	N/A	215	General population	21.0%
***Vietnam***				
(Trung, Hoi et al. 2017)	2011–2012	908	General population	1.1%
(Nadjm, Thuy et al. 2014)	2001–2003	7226	Febrile adults & children patients admitted to hospital	3.5%

**Table 2:** Global outbreaks of scrub typhus.

Region and Country	Reported Year	Case #	Notes
***Australia***			
(Harris, Oltvolgyi et al. 2016)	2011	45	
(Faa, McBride et al. 2003)	2000–2001	10	
(McBride, Taylor et al. 1999)	1996–1997	28	17 cases in the 1st outbreak in north Queensland in 1996, 11 cases in another outbreak in 1997
***China***			
(Cao, Che et al. 2016)	2011	45	
(Hu, Tan et al. 2015)	2013	271	
(Wei, Luo et al. 2014)	2012	29	
(Zhang, Jin et al. 2007)	2005	32	
(Xiangrui, Jinju et al. 1991)	1986	138	
***India***			
(Borkakoty, Jakharia et al. 2016)	2013	30	
(Stephen, Sangeetha et al. 2015)	2012–2013	28	
(Sharma, Krishna et al. 2014)	2012–2013	125	2 outbreaks occurred in Rajasthan in 2012 and 2013
(Subbalaxmi, Madisetty et al. 2014)	2011–2012	176	
(Krishna, Vasuki et al. 2015)	2010–2011	52	
(Sinha, Gupta et al. 2014)	2012	42	
(Sethi, Prasad et al. 2014)	2009–2012	45	
(Gurung, Pradhan et al. 2013)	2011	63	
(Tilak, Kunwar et al. 2011)	2005–2007	61	
(Dass, Deka et al. 2011)	2009–2010	24	
(Vivekanandan, Mani et al. 2010)	2006–2008	50	
(Singh, Devi et al. 2010)	2007	38	
(Vaz and Gupta 2006)	2002	12	
(Kumar, Saxena et al. 2004)	2003	113	
(Sharma, Mahajan et al. 2005)	2003	45	Diagnosed by Weil-Felix test, 12 of the 45 positive cases were tested negative by PCR
(Mathai, Rolain et al. 2003)	2001–2002	28	
***Japan***			
(Jiang, Marienau et al. 2003)	2000, 2001	17	2 outbreaks occurred among U.S. Marines training at Camp Fuji, Japan, 9 cases in ~800 Marines in 2000, 8 cases in ~900 Marines in 2011
***Maldives***			
(Lewis, Yousuf et al. 2003)	2002–2003	14+	168 suspected and confirmed cases. AFRIM tested 28 of them, and 14 were positive
***Palau***			
(Durand, Kuartei et al. 2004)	2001–2003	15	
***Papua New Guinea***			
(Spicer, Taufa et al. 2007)	2001	39	
***Solomon Island***			
(Marks, Joshua et al. 2016)	2014	9	
***Thailand***			
(Rodkvamtook, Gaywee et al. 2013)	2006–2007	26+	142 febrile children with clinically suspected ST, 30 Hmong hill tribe children were tested serologically and genetically.
(Rodkvamtook, Ruang-Areerate et al. 2011)	2002	17	
***Taiwan***			
(Bourgeois, Olson et al. 1977)	1975	69	
(Gale, Irving et al. 1974)	1970	19	

### Epidemiology in the Asia Pacific region

The literature review confirmed that the majority of scrub typhus cases were reported in the “tsutsugamushi triangle” in the Asia-Pacific region (**Table 3**) [23,45,105,155,156,157,158]. There were a few cases reported in Central Asia and the Middle East, which are outside the traditional definition of the Asia Pacific region, but neighboring it [49,145,159].

**Table 3:** Characteristics of scrub typhus in main endemic areas.

Country/Region	Age Distribution	Gender Ratio (F:M)	High Risk Season
**China**	<10 yr (11.84%)	1:1	June & July
	10–19 yr (5.10%)		
	20–29 yr (6.39%)		
(Zhang, Wang et al. 2013)	30–39 yr (10.45%)		
	40–49 yr (18.06%)		
	50–59 yr (21.36%)		
	60–69 yr (16.00%)		
	≥70 yr (10.81%)		
**Japan**	0–25 yr (3%)	1:1	November
(Ogawa, Hagiwara et al. 2002)	25–50 yr (21%)		
	51–75 yr (62%)		
	≥76 yr (14%)		
**South Korea**	<10 yr (1.04%)	13:7	October & November
	10–19 yr (1.21%)		
	20–29 yr (2.24%)		
	30–39 yr (4.84%)		
	40–49 yr (12.05%)		
(Lee, Cho et al. 2015)			
	50–59 yr (21.98%)		
	60–69 yr (27.48%)		
	70–79 yr (22.76%)		
	80–89 yr (6.04%)		
	≥90 yr (0.37%)		
**India**	N/A	N/A	August—October
(Kalra 1952)			
**Thailand**	11–29 yr (15.5%)	1:2	N/A
	30–39 yr (21.2%)		
	40–49 yr (20.1%)		
(Suputtamongkol, Suttinont et al. 2009)			
	50–59 yr (22.3%)		
	60–69 yr (14.0%)		
	≥70 yr (6.8%)		
**Vietnam**	N/A	N/A	Summer
(Nadjm, Thuy et al. 2014)			

N/A means the data is not available from literature search.

### China

The cases are primarily found in southwest China, and the southeast coastal and eastern regions of China. May is usually the start of the scrub typhus season, and June and July are the peak months. The pattern correlates with the weather and life cycle of mites [160]. Recent studies showed that the geographic distribution of the disease has expanded to northern China. It has existed in southern China for thousands of years [158,161]. Scrub typhus cases can be divided into 6 clusters in China. Cluster 1, the significant primary cluster, is located in southern and southeast China, which includes provinces of Guangdong, southern Fujian, Jiangxi, and Guangxi. The secondary cluster is mainly in southwest China, which includes Yunnan and Sichuan Provinces. Jiangsu, Anhui and Shandong provinces in East China are the third cluster for scrub typhus. Shaanxi province in the Northwest and Beijing Municipality were recognized as the fourth and fifth clusters in the analysis done by Zhang *et al*. The provinces of Zhejiang and northern Fujian were cluster 6 [158]. In other studies, there were reported cases in Hunan Province and Tibet [160].

Data collected between 2006 and 2012 show that the highest cumulative incidence was in the 60–69 year-old age group (0.66 per 100,000), and the lowest one was in the 10–19 year-old age group (0.11 per 100,000). The 50–60 year-old group accounted for the largest portion of all scrub typhus patients in China (21.36%). There was no difference in incidence between genders [158].

### Japan

A remarkable resurgence and a prominent outbreak occurred between 1976 and 1984 due to an increase of mite populations carrying *O*. *tsutsugamushi*. There was no explanation available for the increased number of mites during that period [162]. The disease has now been found in almost all areas of Japan except in Okinawa and Hokkaido prefectures. A retrospective study in 1998 demonstrated that Kyushu (51% of total cases), Tohoku-Hokuriku (27%) and Kanto (19%) had the largest numbers of cases [45]. In contrast to China, November accounted for the largest proportion of reported cases driven by the large number of cases in Kanto and Kyushu. May had the second highest monthly number of cases due to the cases in Tohoku-Hokuriku [45]. The age distribution differs between Japan and China. In Japan, 62% of cases were 51–75 years old, while in China this age group accounted for less than half of the total patients (~48.2%) [45,158]. No significant gender differences were observed in *Orientia* infection in Japan. Not surprisingly, working in farming and forestry is an important risk factor for scrub typhus [45,162].

### South Korea

Scrub typhus was first reported in South Korea during the Korean War, but it was still unfamiliar to Korean civilians until 1986 [163]. The disease has subsequently been recognized as the most common rickettsial disease in South Korea [156,163,164]. Nation-wide seroepidemiologic and microbiologic surveys demonstrated that 27.7% to 51% of acute febrile illness patients in South Korea were seropositive for *O*. *tsutsugamushi* between 1986 and 1993 [163]. The study confirmed that scrub typhus was widely spread in the country, and that it was frequently underdiagnosed [163]. Scrub typhus became a reportable disease in South Korea in 1994. Physicians must report all confirmed or suspected scrub typhus cases to both the local health bureau and the Korean Centers for Disease Control and Prevention (CDC). The gender inequality of scrub patients is unique in this country. Several studies confirmed that more female patients were reported than male patients (~65% vs 35%) [70,156]. One possible explanation is due to the conventional working behavior in farms in South Korea. Female workers typically work in a squatting position in dry fields, while male farmers tend to stand with tools in rice fields during work [70]. Other characteristics of the epidemiology of scrub typhus in South Korea are the increasing incidence in urban areas and expansion to northern regions [70,164,165]. The proportion of cases identified in urban areas increased from 20% (388 cases) in 2002 to 26.9% (1,345 cases) in 2009, while that in farmers decreased from 43.3% to 25%. However, further analysis revealed that outdoor activity in urban areas is the most common risk factor [70,164]. Similar to Japan, October and November are the peak months for scrub typhus cases. The age group 60–69 years old is the largest group for scrub typhus cases in South Korea (27.48%), and 72.2% patients were 50–79 years-old [70,156].

### India

Scrub typhus was recognized as a typhus-like fever in India in 1917 [1,166]. It was a major cause of fever among military personnel along the Assam-India-Myanmar (formerly Burma) border during World War II, and the 1965 Indo-Pak war [1,43]. The disease resurged at the Pakistan border of India in 1990 [43]. The widespread use of insecticides and empiric treatment of febrile illness as well as changes in lifestyle all contributed to the subsequent decrease in incidence [43,167]. However, scrub typhus is still an under-diagnosed disease in India [43]. Field epidemiology studies indicate that the disease occurs all over India, from South India to Northeast India and Northwest India. There were cases reported from Maharashtra, Tamil Nadu, Karnataka, Kerala, Himachal Pradesh, Jammu and Kashmir, Uttaranchal, Rajasthan, West Bengal, Bihar, Meghalaya, and Nagaland [27,43,110,168,169]. The peak of the disease is between August and October. *Leptotrombidium deliense* is reported as the primary vector of *O*. *tsutsugamushi* [1,155]. Socioeconomic status and occupation are important risk factors for scrub typhus. Most scrub typhus patients in India are uneducated and live in rural areas [27,43].

### Thailand

The tropical climate of Thailand provides an ideal environment for the vectors of *O*. *tsutsugamushi*, *L*. *deliense* and *L*. *chiangraiensis* [1]. Nationwide sero-epidemiological studies revealed a high prevalence of scrub typhus in Thailand [157,170]. The rates of *O*. *tsutsugamushi* antibodies varied from 13% to 31% of residents in suburban Bangkok, to 59% to 77% of residents in the northern and northeastern regions [157]. A human case was first reported from the central region of Thailand in 1952 [157,171]. The pathogen was first isolated from rodents in the same part of the country two years later [172]. There was a substantial increase in the number of confirmed cases in Thailand from the 1980s to the 2000s [157]. Growing awareness of the disease and development of new diagnostic tools may at least partially contribute to this trend [157,173,174]. Different from China and Japan, the male to female gender ratio of scrub typhus patients in Thailand is 2:1 [157]. The age distribution of the disease in Thailand is that the 50–59 year old group is the largest group (22.3%), but both the 30–39 year old and 40–49 year old groups are similar, ~20%. Outdoor activity, especially occupational exposure, is a critical risk factor [157].

### Vietnam

The earliest reports of scrub typhus cases in Vietnam can be dated back to the 1960s during the Vietnam War [175,176,177,178]. Most of the patients in the 1960s and 1970s were military personnel, especially American servicemen in South Vietnam [46,178]. The disease had been neglected in Vietnam since then until the end of the last century and the beginning of 21^st^ century, resulting in a gap in publications during that time [179]. Differing from past studies, current studies and case reports of scrub typhus in Vietnam have been focused on the northern part of the country. There was only one study in the central part of Vietnam, Quang Nam province, which identified that the main genotype in the area was the Karp group [180]. Recent studies of scrub typhus in North Vietnam demonstrated that the cumulative incidence of scrub typhus was about 1.1% among the general population, and ~3.5% among patients admitted to hospitals [103,105]. The peak season in North Vietnam is summer though cases occur throughout the year. The transmission pattern of scrub typhus in tropical South Vietnam may be different because a seasonal pattern is more obvious in a temperate climate [66,105]. There was no significant difference between urban and rural areas [103].

### Other countries

In addition to the countries described above, there are quite a few other countries with reported scrub typhus cases in the tsutsugamushi triangle. Scrub typhus has been recognized on the islands of the southwest Pacific including Indonesia and the Philippines, and the continent of Australia for almost a century [149,181,182,183,184]. It was recognized as “coastal fever” in 1913 and scrub typhus after the 1920s in Australia. The endemic areas in Australia are the tropical coastal periphery of northeastern Queensland, the tropical region of the Northern Territory, and the adjacent Kimberly region of Western Australia [1,181,182]. A new strain, Litchfield, different from those from other Asia-Pacific area countries was isolated in Australia in 1998 [185]. The Philippines did not confirm the occurrence of scrub typhus until World War II. The first but failed US scrub typhus vaccine was prepared from the lungs and spleens of white rats infected with Volner strain of *O*. *tsutsugamushi*. The Volner strain was originally isolated from the blood of a soldier in the Philippines [149,186]. The history of scrub typhus in Malaysia could be traced back to 1915 [187,188,189]. World War II made the disease known in the Solomon Islands, Republic of Vanuatu, and Papua New Guinea [128,146,190,191,192]. Our literature search found isolated *Orientia* infection in human patients in Far East Russia and Pakistan, and further studies may be necessary to confirm the distribution and other epidemiological features of scrub typhus in these countries [48,153,154,193,194,195,196]. The distribution of scrub typhus covers a large and diverse area. In the Asia-Pacific region alone, different countries in the endemic area have different climates, environment, and culture, which all contribute to the different characteristics of epidemiology of the disease [1,64]. Our study has the limitation that we only analyzed literature in English within the databases of PubMed and Google Scholar. There were quite a few publications in Chinese, Japanese, Korean, Russian, and other languages not included in the analysis.

### Epidemiology outside the “tsutsugamushi triangle”

Our literature review determined that there are a few sporadic scrub typhus cases from countries and regions outside the traditional “tsutsugamushi triangle” in the Asia-Pacific area (**Fig 1**).

### United Arab Emirates (UAE)

UAE is outside the traditional endemic triangle. However, the case reported in 2010 demonstrated a scrub typhus case confirmed to be caused by a new *Orientia* species, *O*. *chuto* [49]. The Australian patient concerned had traveled to Dubai, UAE and the United Kingdom before the onset of her febrile illness. She noticed an eschar on her abdomen after the Dubai visit. An IFA, polymerase chain reaction (PCR), and sequencing were employed to determine the etiologic pathogen. The molecular variance of the 47-kDa gene, 56-kDa gene and other nucleotide sequences, and geographical difference led the researchers to propose the new *Orientia* species. There was only one known *Orientia* species, i.e., *O*. *tsutsugamushi*, before this case [23,49].

### Chile

Before the two scrub typhus reports in Chile, there was no reported autochthonous scrub typhus case in the Western Hemisphere. A patient was bitten by terrestrial leeches on Chiloé Island in southern Chile in 2006 [139]. Both PCR, a molecular biological test, and, IFA, a serological test, showed diagnostic confirmation of *O*. *tsutsugamushi* infection. The molecular analysis further indicated that the pathogen is closely related but not identical to other *O*. *tsutsugamushi* and *O*. *chuto* species. It was significantly closer to *Orientia sp*. than to other rickettsiae. The results suggested that the pathogen from the Chilean sample was not a simple import from an endemic area. In addition, the case also reminds us that there might be other vectors, such as leeches, for *Orientia* [139]. The latest study reported three native cases on the same island in 2016 [151]. The researchers used both serological testing and molecular analysis to diagnose the patients. At least one patient received diagnosis by two serological tests from both the local hospital and the Mahidol-Oxford Tropical Medicine Research Unit in Thailand, and molecular analysis at the Lao-Oxford-Mahosot-Hospital-Wellcome Trust Research Unit. They used paired antibody titer comparison for diagnosis, which is more reliable than a single titer diagnosis [197]. The second patient was documented by molecular analysis of the agent but half of the IFA serologic tests were negative. The third patient had two high single-titer IFA results but negative PCR results. No leech bite was observed in these cases [151]. More follow-up studies could fill these gaps.

### Africa

There are case reports from Cameroon, Kenya, Congo, Djibouti and Tanzania in Africa [102,143,144,147]. The only case report from Cameroon was an American who visited Cameroon before developing a febrile illness [143]. The patient’s IFA titer to *O*. *tsutsugamushi* increased from 1:256 to >1:1,024 two weeks after admission, but PCR analysis of formalin-fixed, paraffin-embedded skin samples was negative. Several clinical features were not typical [143]. The researchers in Kenya screened samples of reactive sera from patients with febrile illness in Kenya. Western blot was performed to confirm the specificity. About 5% of the serum samples contained antibodies reactive with *O*. *tsutsugamushi* [102]. The clinical features and serological test, IIP, confirmed the diagnosis of *O*. *tsutsugamushi* Kato strain in the only case in Congo. However, the patient resided and was diagnosed in Japan. The patient visited Congo for 23 days, and noticed symptoms 8 days after he left Congo. The researchers contacted local centers for disease control in Japan where the patient lived and worked. They did not find similar reports in Japan so they concluded that the patient contracted the disease in Congo. No other case in Congo has been reported [147]. Therefore, it is reasonable to suspect the possibility of domestic infection in Japan instead of acquisition in Congo. More epidemiologic studies of scrub typhus are necessary to confirm the existence of scrub typhus in Congo. A new study of 49 abattoir workers in Djibouti demonstrated that three workers were seropositive against *Orientia*, and one worker seroconverted during the study [80]. The titers observed in the study were 100–400 for ELISAs, and 1:128 for IFA. The cut-off titer and methods used in this study may be controversial, and require further validation [197]. In addition, the study did not provide the participants’ travel history even though the authors played down this confounder due to the subjects’ socioeconomic status [80]. A Dutch traveler to Tanzania contracted *O*. *tsutsugamushi* there. Researchers in the Netherlands confirmed the case with clinical features (fever, eschar, etc.) and serological tests, i.e., IFA [144]. No other case has been reported in Tanzania.

## Discussion

### Prevention and control of scrub typhus

#### Diagnosis

*Orientia* causes a flu-like febrile illness similar to many other diseases, which makes the clinical diagnosis of scrub typhus quite difficult. Generally, the diagnosis is based on the patient’s clinical presentation and history. Presence of an eschar and history of travel to or residence in an endemic area favors the diagnosis. An eschar is not observed in every confirmed patient, and cutaneous lesions from other diseases, e.g., spider bites, leishmaniasis, spotted fever rickettsiosis and anthrax, may make the presence of an eschar less than a definitive diagnostic sign. Scrub typhus can be misdiagnosed as many acute febrile illnesses, including malaria, dengue, leptospirosis, other rickettsioses, meningococcal disease, typhoid fever, infectious mononucleosis and HIV [6,198,199].

Laboratory methods for diagnosing rickettsial diseases including scrub typhus are mainly based on serological tests and molecular assays. Cross-reactivity of *Orientia* with other rickettsiae is rare [200,201,202]. The gold standard test for diagnosis of scrub typhus is the indirect immunofluorescence assay (IFA) [199,203]. However, IFA is expensive and complicated, and requires extensive training and a biocontainment facility for production of the reagents. Even though the assay has been available for many years, the application of IFA in the endemic area is still limited for the above mentioned reasons. The test fails to provide diagnostic results at early stages of infection because those antibodies represent adaptive immunity and are not generated during the early acute infection stage. It is more reliable to interpret the serological tests when the antibody titer has a 4-fold rise [197]. There are other limitations of IFA, from the controversial cut-off antibody titer, and subjective determination of results, to imperfect specificity of the test [203,204].

Other serological tests include the indirect immunoperoxidase assay (IIP), Weil-Felix test (W-F), enzyme-linked immunoassays (ELISAs), and various commercially available immunochromatographic tests (ICT) [203,205]. The W-F agglutination test has been commercially available for many years. This test using the *Proteus mirabilis* OXK strain lacks both specificity and sensitivity, especially the latter, for routine diagnosis [95,206]. Studies have shown that the sensitivity is only 50% during the second week of illness [6,199].

IIP is a modification of IFA, which provides a comparable sensitivity and specificity without the requirement for an ultraviolet microscope in diagnosis of scrub typhus [202,207]. Both IFA and IIP have been used as the reference standard. No significant difference was found in the accuracy of the two tests, except for one study which claimed that IIP was more sensitive than the IFA with acute sera (79.6% vs. 68.5% at titer ≥1:400) [208,209].

ELISAs and their variants, such as commercially available dipstick tests, use either pooled cell lysates of different strains of *O*. *tsutsugamushi* as antigen, or recombinant p56 or other outer membrane proteins as the antigen. ELISAs provide sensitive and specific test results. They eventually may replace the IFA and IIP assays. The sensitivity and specificity of dipstick assays are inferior to ELISAs, but these commercially available assays are easy to use, and could be performed in underserved areas [6,208,209] Blacksell et al. reported experience with InBios Scrub Typhus Detect IgM ELISA, which was proven to be easy-to-use, affordable, and to have adequate accuracy for screening and diagnosis [106]. ELISA-based tests should be considered a good alternative to the gold standard IFA.

ICT is another commercially available kit for early rapid diagnosis. The test also uses the recombinant *Orientia* outer membrane proteins to detect IgG, IgM and IgA antibodies to *O*. *tsutsugamushi*. Evaluation of the test indicated that ICT has moderate to high sensitivity (~70%) among scrub typhus patients. The sensitivity increases with the fever duration. Several studies claimed that, similar to the passive hemagglutination assay (PHA), ICT also has a substantial number of false negative results [204]. PHA was replaced by ICT due to the former’s lower sensitivity. However, Lim *et al*. demonstrated that the specificity of IFA IgM is low, which caused inaccurate comparisons between IFA and other diagnostic assays. By contrast, the IgM ICT has comparable sensitivity and significantly better specificity than IFA as assessed using Bayesian latent class models [203,204]. Kingston et al. demonstrated InBios Scrub Typhus Detect IgM Rapid test to have high specificity and promising accuracy [210].

The molecular method to diagnose scrub typhus is detecting the bacteria with PCR assays. PCRs usually target the genes of the outer membrane proteins of 56 kDa, 47 kDa, and groEL genes [203]. It was reported that nested PCR may be more sensitive than the serological tests including the gold standard, IFA [211,212]. This molecular biologic method can detect *Orientia* DNA in blood even during the persistent phase of the infection, when no obvious clinical symptoms are observed. However, the sensitivity of PCRs decreases with treatment [6,199,213,214]

#### Treatment

As a gram-negative bacterium, *Orientia* infection can be effectively treated with the appropriate antibiotics. Early treatment results in better outcomes, i.e., shortening the disease course and reducing fatalities [63]. Oral treatment is effective for mild cases, but the parenteral route is often necessary for severely ill patients. Similar to treatment for other rickettsial diseases, doxycycline is one of most effective antibiotics for treating scrub typhus. Antibiotics are usually able to abate patients’ fever rapidly, and this outcome is even used as a diagnostic indicator. Some randomized clinical trials determined that there is no significant difference in outcomes among tetracycline, doxycycline, telithromycin, and azithromycin, the latter of which is a recommended treatment considered to be as effective as doxycycline [215,216,217]. Rifampicin was shown to be more effective than tetracycline in patients responding poorly to doxycycline [6,217,218]. World Health Organization (WHO) recommends that pregnant women or children can use azithromycin or chloramphenicol. Multiple studies proved that azithromycin and other macrolides are as effective as doxycycline or chloramphenicol but without potential side effects as aplastic anemia in treatment of children with chloramphenicol [216,219,220].Antibiotic resistance has been reported in a few papers [221,222]. There is much unknown regarding antibiotic resistance. It will be useful for the development of new diagnostic tools and treatments to elucidate the mechanisms of poor response in certain patients, as well as the antibiotic resistance of the pathogen [6,63,223].

#### Current prevention methods

There is no vaccine available for any rickettsial infections including scrub typhus. The enormous antigenic variation in different *O*. *tsutsugamushi* strains, and weak and short lived cross protection among different strains hamper the development of an effective vaccine. Vaccine efforts are also hindered by the different antigenically divergent strains of *O*. *tsutsugamushi* in different endemic countries/regions, or even among different strains in the same location [50,224,225].

WHO recommends prophylactic treatment under special circumstances in the endemic areas [226]. A single oral dose of doxycycline, chloramphenicol or tetracycline every 5 days for a total of 35 days provided protection against *Orientia* infection [50]. A prospective randomized double blind study among Taiwanese military personnel confirmed that prophylactic treatment with doxycycline could decrease the incidence of scrub typhus to 1/5 of that in the placebo group [227]. The US army has used a weekly dose of doxycycline to prevent scrub typhus [228]. However, CDC in the U.S. does not recommend using antibiotics as prophylaxis for rickettsial diseases including scrub typhus because the preventive treatment may simply delay onset of disease and make diagnosis more difficult [224,229]. To treat rickettsial diseases more effectively, CDC suggests starting treatment based on clinical suspicion alone [229,230].

General protective measures can be followed to avoid the infection. These precautions are critical for people living in or visiting endemic areas [50,224,226,231]:

1. Avoid outbreaks. Persons should avoid known focal outbreak areas to the extent that this is possible. Travelers can check the regional disease transmission and outbreak information at http://www.cdc.gov/travel.
2. Avoid exposure conditions. Chiggers reside in grass, woodlands, and other vegetated areas. Persons are encouraged to avoid the outdoors or take preventive actions. Do not sit or lie on bare ground or grass; use a sheet or a cover on the ground instead.
3. Wear appropriate clothing. Persons should wear long-sleeved shirts, long pants, boots and hats to reduce exposure. Persons should tuck in shirts and pants, and wear closed shoes.
4. Insect and spatial repellents. Persons should apply insect repellent containing dibutyl phthalate, benzyl benzoate, diethyl toluamide or other chemicals to their skin and permethrin to their clothing, to prevent chigger bites.
5. Insecticides and habitat modification. Farmers and field workers can improve sanitation, clear vegetation, control rodents, use insecticides and chemically treat the soil. These steps can impede the propagation of chiggers and the transmission cycle.
6. Thorough cleaning after visiting high risk areas. Due to its small size of 0.2–0.4 mm, detection of mite larva on clothing or skin is extremely difficult. Prompt removal of clothing and thorough cleaning of skin and clothes with detergent after work or travel and at the end of the day can reduce the risk of infection.

#### Strategies for prevention and control

The reemergence and geographic expansion of scrub typhus in the Asia-Pacific area as well as the increase in antibiotic resistance reminds us of the urgency of developing and adopting effective control and preventive measures [50,225,227,232,233].

Though no vaccine is available, a number of other preventive measures can be taken. To make these measures work, public education on case recognition and personal protection is the priority. WHO recommends that advocacy, awareness and educational activities should be targeted at schoolchildren, teachers and women in endemic areas [226]. However, field workers and outdoor travelers may have higher risks of becoming infected, so the educational program should not be limited to the three groups mentioned above. Farms and other places with bush, wood piles, rodents, and domestic animals increase the risk of infection. One strategy is to avoid high risk areas. For field workers and those people who cannot avoid the risk factors, taking the precautions as described in the last section will help prevent the disease [50,224,225,226,227,231].

Kwak et al. simulated incidence of scrub typhus with selected meteorological predictors, such as temperature, precipitation, relative humidity, wind speed, duration of sunshine, and cloud cover[47]. The study indicated that the seasonality of meteorological factors affects model prediction. Korean CDC and researchers demonstrated the expansion of scrub typhus high incidence area to northern regions of South Korea, which they suggested might be associated with global warming [165]. We should consider these factors when predicting the disease cycle, and employ appropriate strategies of prevention and control [47,224,225,226]

Rodent control and habitat modification are helpful for disease control and prevention. Different rodent control strategies can be employed, from trapping, to poisoning, and use of natural predators [226,234]. Rodent control is important but difficult, especially maintaining the control long-term. Public education is a priority for the control of rodents and mites [234,235]. Poisoning is widely used in the agricultural sector in the endemic areas, but secondary poisoning poses hazards to other animals and humans. For this reason using the natural predators of rodents and other natural forms of control are preferred. Habitat modification, such as good sanitation in and around buildings, clearing vegetation around fields, and secure storage of grain, can make areas less suited for rodents, and prevent them from flourishing in high numbers [50,226,232,233].

The great clinical and public health challenge of scrub typhus is its difficulty in diagnosis, especially early diagnosis. Early diagnosis and early treatment can significantly reduce the complications and fatality rate caused by *Orientia*. WHO suggests improving the awareness of empiric therapy and also urges researchers to develop affordable and easy-to-use diagnostic assays with high sensitivity and specificity [6,50,199,203,225,226]. Broader deployment of diagnostic testing is likely to identify new areas where scrub typhus has emerged.

Even though scrub typhus can be a life-threatening disease, collaborative actions in the countries and regions in the endemic areas using the strategies mentioned above can effectively control and prevent the outbreak of this neglected disease.

## Supporting information

S1 Table PRISMA checklist and flowchart. (PDF)

S2 Table Reported human scrub typhus cases without seroprevalence or outbreak. (PDF)

S3 Table The list of literature found during the systematic review. (PDF)
