# Supplementary material for: A review of the global epidemiology of scrub typhus
Source: PLoS Negl Trop Dis. 2017 Nov 3;11(11):e0006062. doi: 10.1371/journal.pntd.0006062 (PMC5687757; doi:10.1371/journal.pntd.0006062)
Supplement: S2 Table — (PDF) [file pntd.0006062.s002.pdf]

S1 Table. Reported Human Scrub Typhus Cases without Seroprevalence or Outbreak

Region/Country	Reported by	Case #
Cambodia	(Corwin, Soeprapto et al. 1997; Chheng, Carter et al. 2013)	Multiple
Cameroon[1]	(Ghorbani, Ghorbani et al. 1997)	1
Chile	(Balcells, Rabagliati et al. 2011; Weitzel, Dittrich et al. 2016)	4
Djibouti	(Horton, Jiang et al. 2016)	3
Micronesia	(Durand, Kuartei et al. 2004)	
Myanmar (Burma)	(Sayen, Pond et al. 1946; Audy 1947; Parola, Miller et al. 2003)	
Pakistan	(Shirai and Wisseman 1975)	2
PR Congo	(Osuga, Kimura et al. 1991)	1
Russia	(Tarasevich, Kulagin et al. 1968)	
Tajikistan	(Kulagin, Tarasevic et al. 1968)	
Tanzania	(Groen, Nur et al. 1999)	
the Philippines	(Errington, King et al. 1946; Philip, Woodward et al. 1946; Woodward, Philip et al. 1946)	
Vanuatu	(Miles, Austin et al. 1981)	

1. This case could not be confirmed by the researcher (Ghorbani, Ghorbani et al. 1997)
