# Supplementary material for: A review of the global epidemiology of scrub typhus
Source: PLoS Negl Trop Dis. 2017 Nov 3;11(11):e0006062. doi: 10.1371/journal.pntd.0006062 (PMC5687757; doi:10.1371/journal.pntd.0006062)
Supplement: S3 Table — (PDF) [file pntd.0006062.s003.pdf]

S2. The list of literature found during the systematic review

1. Infect Genet Evol. 2015 Dec;36:126-30. doi: 10.1016/j.meegid.2015.09.008. Epub 2015 Sep 12.

Genotype diversity and distribution of Orientia tsutsugamushi in scrub typhus patients and rodents in Shandong, northern China.

Zheng L(1), Bi Z(2), Kou Z(2), Yang H(1), Zhang L(1), Zhao Z(3).

Author information:
(1)Department of Epidemiology, School of Public Health, Shandong University, 44 Wenhuaxi Road, Jinan 250012, China. (2)Institute for Bacterial Infectious Disease Control and Prevention, Shandong Center for Disease Control and Prevention, Jinan 250012, China. (3)Department of Epidemiology, School of Public Health, Shandong University, 44 Wenhuaxi Road, Jinan 250012, China. Electronic address: ztzhao@sdu.edu.cn.

Orientia tsutsugamushi, the etiologic agent of scrub typhus, is transmitted to humans through the bites of infected chiggers. To identify the genotypes of O.tsutsugamushi and their geographical distribution in Shandong, 236 patient's samples and 1606 captured rodents were collected in major-endemic regions of Shandong Province from June 2013 to December 2014. Sequences were determined for the 56-kDa TSA gene, and the relationship between these sequences and those previously determined was assessed. Two genotypes out of 5 previously reported in Shandong were identified, i.e. Kawasaki-related and STA-07. The Kawasaki-related genotype was predominant (82.1% (23/28) in human and 50% (5/10) in rodents), with wide distribution through the endemic areas of Shandong Province. The STA-07 was confined to Tai'an, Linyi and Qingdao districts. The Fuji-related, Shimikoshi-related and Karp-related genotypes were not found, while identified in previous studies. For prevention and control of scrub typhus in Shandong, more attention should be paid to surveillance of Kawasaki-related and STA-07 genotypes.

Copyright © 2015 Elsevier B.V. All rights reserved.

DOI: 10.1016/j.meegid.2015.09.008
PMID: 26371067 [PubMed - indexed for MEDLINE]

2. Infect Genet Evol. 2015 Mar;30:238-43. doi: 10.1016/j.meegid.2014.12.036. Epub 2015 Jan 6.

Scrub typhus caused by Orientia tsutsugamushi Kawasaki-related genotypes in Shandong Province, northern China.

Zhang L(1), Bi Z(2), Kou Z(2), Yang H(3), Zhang A(3), Zhang S(4), Meng X(3), Zheng L(1), Zhang M(1), Yang H(1), Zhao Z(5).

Author information:
(1)Department of Epidemiology and Health Statistics, School of Public Health, Shandong University, 44 Wenhuaxi Road, Jinan 250012, China. (2)Institute for Bacterial Infectious Disease Control and Prevention, Shandong Center for Disease

Control and Prevention, Jinan 250012, China. (3)Tai'an Center for Disease Control and Prevention, Tai'an 271000, China. (4)Yiyuan Center for Disease Control and Prevention, Zibo 256100, China. (5)Department of Epidemiology and Health Statistics, School of Public Health, Shandong University, 44 Wenhuaxi Road, Jinan 250012, China. Electronic address: ztzhao@sdu.edu.cn.

Orientia tsutsugamushi, the causative agent of scrub typhus, is an obligate intracellular bacterium and characterized by dramatic genetic diversity. To elucidate the genotypes of O. tsutsugamushi populating in patients in Shandong Province, a new epidemic zone in China, we sequenced partial of the 56-kDa type-specific antigen gene (TSA) and identified the genotypes of 43 O. tsutsugamushi samples from human patients confirmed with scrub typhus from 2010 to 2013. All of the 43 sequences are in the same clade, 39 of them are in one branch and the other four sequences, nominated as SH1002, SH1306, SH1309, and SH1307 are in four separate branches. To clarify the clinical characterizations caused by Kawasaki-related genotypes, we studied the clinical profiles of these 43 scrub typhus patients. Most patients (88.1%) were farmers lived in rural areas. They presented with fever (100.0%), headache (79.1%), dizziness (32.6%), generalized myalgia (48.8%), fatigue (53.5%), anorexia (53.5%), facial flushing (23.3%), conjunctival congestion (11.6%), skin rashes (58.1%) and lymphadenopathy (23.3%). Eschar (97.7%) was quite common in patients, which provided doctors with a luminous clue for diagnosis of scrub typhus. Thrombocytopenia was seen in 23.1% of patients, and three patients (7.0%) had bronchopneumonia. There was no death report in Shandong Province during the study period. The present study provides beneficial data for clinical, serological, and molecular diagnosis of scrub typhus infections, and also provides foundations for subsequent studies.

Copyright © 2015 Elsevier B.V. All rights reserved.

DOI: 10.1016/j.meegid.2014.12.036
PMID: 25575441 [PubMed - indexed for MEDLINE]

3. Zhonghua Liu Xing Bing Xue Za Zhi. 2016 Feb;37(2):232-7. doi: 10.3760/cma.j.issn.0254-6450.2016.02.017.

[Spatio-temporal distribution of scrub typhus and related influencing factors in coastal beach area of Yancheng, China].

[Article in Chinese]

Chen YZ(1), Li F(1), Xu H(1), Huang LC(1), Gu ZG(1), Sun ZY(1), Yan GJ(2), Zhu YJ(3), Tang C(4).

Author information:
(1)Yancheng Center for Disease Control and Prevention, Yancheng 224002, China.
(2)Dongtai Center for Disease Control and Prevention, Yancheng 224200, China.
(3)Binhai Center for Disease Control and Prevention, Yancheng 224500, China.
(4)Dafeng Center for Disease Control and Prevention, Yancheng 224100, China.

OBJECTIVE: In order to provide better programs on monitoring, early warning and prevention of Scrub Typhus in the coastal beach area, temporal-spatial distribution characteristics of scrub typhus were summarized. Relationships between temporal-spatial clustering of Scrub Typhus, meteorological factors,

rodent distribution and the biological characteristics in coastal beach area of Yancheng city, were studied.
METHODS: Reports on network-based Scrub Typhus epidemics and information on population, weather situation through monitoring those stations, from 2005 to 2014 were collected and processed, in the coastal beach area of Yancheng city. Distribution, density of the population concerned and seasonal fluctuation on rodents were monitored in coastal beach area, from April 2011 to December, 2013. METHODS as descriptive statistics, space-time permutation scantistics, autocorrelation and Cross-correlation analysis etc, were used to analyze the temporal-spatial distribution of Scrub Typhus and correlation with rodent distribution, density fluctuation and meteorological indexes. Zero-inflated Pearson (ZIP) regression model was contributed according to the distribution of related data. All methods were calculated under Excel 2003, SPSS 16.0, Mapinfo 11.0, Satscan 9.0 and Stata/SE 10.0 softwares.
RESULTS: (1) The incidence of Scrub Typhus was gradually increasing and the highest incidence of the year was seen in 2014, as 5.81/10 million. There was an autumn peak of Scrub typhus, with the highest incidence rate as 12.02/10 million in November. The incidence rate of Scrub typhus appeared high in Binhai, Dafeng and Xiangshui, with the average incidence rates appeared as 3.30/10 million, 3.21/10 million and 2.79/10 million, respectively. There were 12 towns with high incidence rates in the coastal beach area, with incidence rate showed between 4.41/10 and 10.03/10 million. (2) There were three incidence clusters of Scrub typhus seen in 25 towns, between October 2012 and November 2012 in Dongtai, Dafeng, Sheyang areas and 5 towns between October and November, 2014 in Xiangshui area, together with another 6 towns in November of 2006, in Binhai area. (3) Apodemus agrarius appeared the dominant species in the coastal area, with the constituent ratio as 89.19%. The rodent density appeared two peaks in winter and summer in 2011 and 2013. The winter peak was seen in January and the summer peak lasting for 5-8 months. Scrub Typhus was seen 10-11 months in a year and the incidence was increasing, parallel with the peak of the rodent density. The peak incidence of Scrub Typhus showed a temperature/rainfall-related peak. Rodent density, temperature, rainfalls were correlated with the incidence of Scrub Typhus, under the Cross correlation analysis. Rains, Mean minimum temperature of a 3-month lagging were directly correlated but the duration of sunshine and relative humidity were negatively correlated with the incidence of Scrub Typhus, under the Zero-inflated Pearson (ZIP) regression model.
CONCLUSION: Temporal-spatial clustering and factors as media creature and weather condition of Scrub Typhu were discovered, which provided evidence for effective measures on prevention and control of the disease.

PMID: 26917522 [PubMed - indexed for MEDLINE]

4. Infect Genet Evol. 2015 Apr;31:1-8. doi: 10.1016/j.meegid.2015.01.005. Epub 2015 Jan 8.

Orientia tsutsugamushi, agent of scrub typhus, displays a single metapopulation with maintenance of ancestral haplotypes throughout continental South East Asia.

Wongprompitak P(1), Duong V(2), Anukool W(3), Sreyrath L(2), Mai TT(4), Gavotte L(5), Moulia C(5), Cornillot E(6), Ekpo P(7), Suputtamongkol Y(8), Buchy P(2), Frutos R(9).

Author information:
(1)Department of Immunology, Faculty of Medicine Siriraj Hospital, Mahidol University, Bangkok 10700, Thailand; UM2, ISEM, UMR 5554, CNRS-UM2-IRD, Université Montpellier 2 CC065, Place E. Bataillon, 34095 Montpellier Cedex 5, France. (2)Virology Unit, Institut Pasteur in Cambodia, 5 Monivong blvd, PO Box 983, Phnom Penh, Cambodia. (3)Department of Immunology, Faculty of Medicine Siriraj Hospital, Mahidol University, Bangkok 10700, Thailand; Graduate Program in Immunology, Faculty of Medicine Siriraj Hospital, Mahidol University, Bangkok 10700, Thailand. (4)Institut Pasteur of Nha Trang, 10 Tran Phu Street, Nha Trang, Viet Nam. (5)UM2, ISEM, UMR 5554, CNRS-UM2-IRD, Université Montpellier 2 CC065, Place E. Bataillon, 34095 Montpellier Cedex 5, France. (6)UM1, CPBS, UMR 5236, CNRS-UM1-UM2, 1919 route de Mende, 34293 Montpellier Cedex 5, France. (7)Department of Immunology, Faculty of Medicine Siriraj Hospital, Mahidol University, Bangkok 10700, Thailand. (8)Department of Immunology, Faculty of Medicine Siriraj Hospital, Mahidol University, Bangkok 10700, Thailand; Department of Medicine, Faculty of Medicine Siriraj Hospital, Mahidol University, Bangkok 10700, Thailand. (9)UM2, CPBS, UMR 5236, CNRS-UM1-UM2, 1919 route de Mende, 34293 Montpellier Cedex 5, France; Cirad, UMR 17, Cirad-Ird, TA-A17/G, Campus International de Baillarguet, 34398 Montpellier Cedex 5, France.
Electronic address: roger.frutos@univ-montp2.fr.

Orientia tsutsugamushi is the causative agent of scrub typhus, a major cause of febrile illness in rural area of Asia-Pacific region. A multi-locus sequence typing (MLST) analysis was performed on strains isolated from human patients from 3 countries in Southeast Asia: Cambodia, Vietnam and Thailand. The phylogeny of the 56-kDa protein encoding gene was analyzed on the same strains and showed a structured topology with genetically distinct clusters. MLST analysis did not lead to the same conclusion. DNA polymorphism and phylogeny of individual gene loci indicated a significant level of recombination and genetic diversity whereas the ST distribution indicated the presence of isolated patches. No correlation was found with the geographic origin. This work suggests that weak divergence in core genome and ancestral haplotypes are maintained by permanent recombination in mites while the 56-kDa protein gene is diverging in higher speed due to selection by the mammalian immune system.

Copyright © 2015 Elsevier B.V. All rights reserved.

DOI: 10.1016/j.meegid.2015.01.005
PMID: 25577986 [PubMed - indexed for MEDLINE]

5. Emerg Infect Dis. 2015 Jan;21(1):64-9. doi: 10.3201/eid2101.140580.

Molecular epidemiology and genetic diversity of Orientia tsutsugamushi from patients with scrub typhus in 3 regions of India.

Varghese GM, Janardhanan J, Mahajan SK, Tariang D, Trowbridge P, Prakash JA, David T, Sathendra S, Abraham OC.

Scrub typhus, an acute febrile illness that is widespread in the Asia-Pacific region, is caused by the bacterium Orientia tsutsugamushi, which displays high levels of antigenic variation. We conducted an investigation to identify the circulating genotypes of O. tsutsugamushi in 3 scrub typhus-endemic geographic regions of India: South India, Northern India, and Northeast India. Eschar

samples collected during September 2010-August 2012 from patients with scrub typhus were subjected to 56-kDa type-specific PCR and sequencing to identify their genotypes. Kato-like strains predominated (61.5%), especially in the South and Northeast, followed by Karp-like strains (27.7%) and Gilliam and Ikeda strains (2.3% each). Neimeng-65 genotype strains were also observed in the Northeast. Clarifying the genotypic diversity of O. tsutsugamushi in India enhances knowledge of the regional diversity among circulating strains and provides potential resources for future region-specific diagnostic studies and vaccine development.

DOI: 10.3201/eid2101.140580
PMCID: PMC4285260
PMID: 25530231 [PubMed - indexed for MEDLINE]

6. PLoS Negl Trop Dis. 2015 Aug 14;9(8):e0003971. doi: 10.1371/journal.pntd.0003971. eCollection 2015.

A Systematic Review of Mortality from Untreated Scrub Typhus (Orientia tsutsugamushi).

Taylor AJ(1), Paris DH(2), Newton PN(1).

Author information:
(1)Lao Oxford-Mahosot Hospital-Wellcome Trust Research Unit (LOMWRU), Microbiology Laboratory, Mahosot Hospital, Vientiane, Laos; Centre for Tropical Medicine and Global Health, Nuffield Department of Clinical Medicine, Churchill Hospital, University of Oxford, Oxford, United Kingdom. (2)Lao Oxford-Mahosot Hospital-Wellcome Trust Research Unit (LOMWRU), Microbiology Laboratory, Mahosot Hospital, Vientiane, Laos; Centre for Tropical Medicine and Global Health, Nuffield Department of Clinical Medicine, Churchill Hospital, University of Oxford, Oxford, United Kingdom; Mahidol-Oxford Research Unit (MORU), Faculty of Tropical Medicine, Mahidol University, Bangkok, Thailand.

BACKGROUND: Scrub typhus, a bacterial infection caused by Orientia tsutsugamushi, is increasingly recognized as an important cause of fever in Asia, with an estimated one million infections occurring each year. Limited access to health care and the disease's non-specific symptoms mean that many patients are undiagnosed and untreated, but the mortality from untreated scrub typhus is unknown. This review systematically summarizes the literature on the untreated mortality from scrub typhus and disease outcomes.
METHODOLOGY/PRINCIPAL FINDINGS: A literature search was performed to identify patient series containing untreated patients. Patients were included if they were symptomatic and had a clinical or laboratory diagnosis of scrub typhus and excluded if they were treated with antibiotics. The primary outcome was mortality from untreated scrub typhus and secondary outcomes were total days of fever, clinical symptoms, and laboratory results. A total of 76 studies containing 89 patient series and 19,644 patients were included in the final analysis. The median mortality of all patient series was 6.0% with a wide range (min-max) of 0-70%. Many studies used clinical diagnosis alone and had incomplete data on secondary outcomes. Mortality varied by location and increased with age and in patients with myocarditis, delirium, pneumonitis, or signs of hemorrhage, but not according to sex or the presence of an eschar or meningitis. Duration of fever was shown to be long (median 14.4 days Range (9-19)).

CONCLUSIONS: Results show that the untreated mortality from scrub typhus appears lower than previously reported estimates. More data are required to clarify mortality according to location and host factors, clinical syndromes including myocarditis and central nervous system disease, and in vulnerable mother-child populations. Increased surveillance and improved access to diagnostic tests are required to accurately estimate the untreated mortality of scrub typhus. This information would facilitate reliable quantification of DALYs and guide empirical treatment strategies.

DOI: 10.1371/journal.pntd.0003971
PMCID: PMC4537241
PMID: 26274584 [PubMed - indexed for MEDLINE]

7. N Engl J Med. 2016 Sep 8;375(10):954-61. doi: 10.1056/NEJMoa1603657.

Endemic Scrub Typhus in South America.

Weitzel T(1), Dittrich S(1), López J(1), Phuklia W(1), Martinez-Valdebenito C(1), Velásquez K(1), Blacksell SD(1), Paris DH(1), Abarca K(1).

Author information:
(1)From Clínica Alemana de Santiago, Facultad de Medicina Clínica Alemana, Universidad del Desarrollo (T.W.), Veterinary Hospital Puente Alto (J.L.), and Departamento de Enfermedades Infecciosas e Inmunología Pediátrica (C.M.-V., K.A.) and Millennium Institute on Immunology and Immunotherapy (K.A.), Escuela de Medicina, Pontificia Universidad Católica de Chile, Santiago, and Hospital de Ancud, Ancud (K.V.) - all in Chile; Lao-Oxford-Mahosot Hospital-Wellcome Trust Research Unit, Vientiane, Laos (S.D., W.P.); Centre for Tropical Medicine and Global Health, Nuffield Department of Medicine, University of Oxford, Oxford, United Kingdom (S.D., S.D.B., D.H.P.); and Mahidol-Oxford Tropical Medicine Research Unit, Faculty of Tropical Medicine, Mahidol University, Bangkok, Thailand (S.D.B., D.H.P.).

Scrub typhus is a life-threatening zoonosis caused by Orientia tsutsugamushi organisms that are transmitted by the larvae of trombiculid mites. Endemic scrub typhus was originally thought to be confined to the so called "tsutsugamushi triangle" within the Asia-Pacific region. In 2006, however, two individual cases were detected in the Middle East and South America, which suggested that the pathogen was present farther afield. Here, we report three autochthonous cases of scrub typhus caused by O. tsutsugamushi acquired on Chiloé Island in southern Chile, which suggests the existence of an endemic focus in South America. (Funded by the Chilean Comisión Nacional de Investigación Científica y Tecnológica and the Wellcome Trust.).

DOI: 10.1056/NEJMoa1603657
PMID: 27602667 [PubMed - indexed for MEDLINE]

8. Parasit Vectors. 2015 Dec 1;8:611. doi: 10.1186/s13071-015-1221-7.

Surveillance of potential hosts and vectors of scrub typhus in Taiwan.

Kuo CC(1), Lee PL(2), Chen CH(3), Wang HC(4).

Author information:
(1)Department of Life Science, National Taiwan Normal University, Taipei, Taiwan. cckuo@ntnu.edu.tw. (2)Center for Research, Diagnostics and Vaccine Development, Centers for Disease Control, Ministry of Health and Welfare, Taipei, No. 161, Kun-Yang Street, Taipei, 11561, Taiwan. leepl@ntu.edu.tw. (3)Center for Research, Diagnostics and Vaccine Development, Centers for Disease Control, Ministry of Health and Welfare, Taipei, No. 161, Kun-Yang Street, Taipei, 11561, Taiwan. hc0257@mail.hcbaphiq.gov.tw. (4)Center for Research, Diagnostics and Vaccine Development, Centers for Disease Control, Ministry of Health and Welfare, Taipei, No. 161, Kun-Yang Street, Taipei, 11561, Taiwan. sjwang@cdc.gov.tw.

BACKGROUND: Scrub typhus is a lethal infectious disease vectored by larval trombiculid mites (i.e. chiggers) infected with Orientia tsutsugamushi (OT) and recent decades have witnessed an emergence of scrub typhus in several countries. Identification of chigger species and their vertebrate hosts is fundamental for the assessment of human risks to scrub typhus under environmental changes, but intensive and extensive survey of chiggers and their hosts is still lacking in Taiwan.
METHODS: Chiggers were collected from shrews and rodents in nine counties of Taiwan and were assayed for OT infections with nested polymerase chain reaction (PCR). PCR products were further sequenced to reveal probable OT strains. Rodents were assessed for OT exposure by immunofluorescent antibody assay. Lastly, incidence rate of scrub typhus in each county was associated with loads and prevalence of chigger infestations, seropositivity rate in rodents, and OT positivity rate in chiggers.
RESULTS: Rattus losea was the most abundant (48.7% of 1,285 individuals) and widespread (occurred in nine counties) small mammal species and hosted the majority of chiggers (76.4% of 128,520 chiggers). Leptotrombidium deliense was the most common (64.9% of all identified chiggers) and widespread (occurred in seven counties) chigger species but was replaced by Leptotrombidium pallidum or Leptotrombidium scutellare during the cold seasons in two counties (Matsu and Kinmen) where winter temperatures were lower than other study sites. Seropositivity rate for OT exposure in 876 assayed rodents was 43.0% and OT positivity rate in 347 pools of chiggers was 55.9%, with 15 OT strains identified in the 107 successfully sequenced samples. Incidence rate of scrub typhus was positively correlated with chigger loads, prevalence of chigger infestations, seropositivity rate but not OT positivity rate in chiggers.
CONCLUSIONS: Our study reveals R. losea as the primary host for chiggers and there exists a geographical and seasonal variation in chigger species in Taiwan. It also emphasizes the importance of recognition of chigger vectors and their vertebrate hosts for a better prediction of human risks to scrub typhus under rapid environmental changes.

DOI: 10.1186/s13071-015-1221-7
PMCID: PMC4666075
PMID: 26626287 [PubMed - indexed for MEDLINE]

9. Trop Doct. 2016 Jul 13. pii: 0049475516658400. [Epub ahead of print]

Multiple eschars in scrub typhus: a case report.

Koraluru M(1), Nandigam M(2), Bairy I(3), Vidyasagar S(4), Varma M(5).

Author information:
(1)Lecturer, Department of Microbiology, Kasturba Medical College, Manipal University, Manipal, Karnataka, India. (2)Intern, Kasturba Medical College, Manipal University, Manipal, Karnataka, India. (3)Professor and Head, Department of Microbiology, MMMC, Manipal Campus, Manipal University, Manipal, Karnataka, India. (4)Professor and Unit Head, Department of Medicine, Kasturba Medical College, Manipal University, Manipal, Karnataka, India. (5)Professor and Unit Head, Department of Medicine, Kasturba Medical College, Manipal University, Manipal, Karnataka, India drmuralidharvarma@yahoo.co.in.

Eschar in scrub typhus aids in early diagnosis and institution of appropriate therapy; however, the eschar positivity rates vary greatly in endemic regions. Multiple eschars in scrub typhus are a rare presentation. Our patient presented with fever and multiple eschars and was empirically started on doxycycline. Nested polymerase chain reaction from all the four eschars and from EDTA blood were positive for 56-kDa type-specific antigen which is specific for Orientia tsutsugamushi The patient recovered completely after 7 days of antibiotic treatment. He was from an area where scrub typhus was not observed previously. An eschar in an acute febrile patient from the "tsutsugamushi triangle" is a valuable sign in scrub typhus diagnosis. A search for multiple eschars in scrub typhus must be made by clinicians.

© The Author(s) 2016.

DOI: 10.1177/0049475516658400
PMID: 27411364 [PubMed - as supplied by publisher]

10. Acta Trop. 2014 Mar;131:117-23. doi: 10.1016/j.actatropica.2013.11.029. Epub 2013 Dec 17.

Field assessment of Orientia tsutsugamushi infection in small mammals and its association with the occurrence of human scrub typhus in Taiwan.

Lin PR(1), Tsai HP(1), Weng MH(1), Lin HC(1), Chen KC(1), Kuo MD(1), Tsui PY(1), Hung YW(1), Hsu HL(1), Liu WT(2).

Author information:
(1)Institute of Preventive Medicine, National Defense Medical Center, Taipei, Taiwan. (2)Institute of Preventive Medicine, National Defense Medical Center, Taipei, Taiwan. Electronic address: jeffwtliu@yahoo.com.tw.

We conducted an extensive study in Taiwan of Orientia tsutsugamushi (OT) infection in small wild mammals. Field trapping was carried out at six districts in eastern and western Taiwan as well as various offshore islands during the period 2006-2010. A total of 1061 specimens representing 11 rodent species were captured. The presence of OT infection was assessed by indirect immunofluorescence assay and polymerase chain reaction assays of 56-kDa type-specific antigen gene. The chigger infestation rate among the animals was 35% (371/1061). Among these, OT was detected in 64% (238/371) of the chiggers from the infested animals and in the spleens from 273 (34.3%) of 797 animals. Excluding animals in the Suncus murinus group, the antibody positive rate of scrub typhus was 69.1% (477 of 690 of serum samples). The prevalence of OT

infection in animals from areas with a low incidence of human cases of scrub typhus was significantly lower than that in rodents obtained from regions with a high incidence of human cases of the disease (44.4%±4.0% vs. 71.2%±9.7%, $p<0.001$). In Taiwan, the prevalence of OT infection in wild rodents is considerably high and appears to correlate positively with the occurrence of scrub typhus in humans.

Copyright © 2013 Elsevier B.V. All rights reserved.

DOI: 10.1016/j.actatropica.2013.11.029
PMID: 24361181 [PubMed - indexed for MEDLINE]

11. Vector Borne Zoonotic Dis. 2016 Aug 18. [Epub ahead of print]

Seroprevalence of Scrub Typhus Infection in Arunachal Pradesh, India.

Jakharia A(1), Borkakoty B(2), Biswas D(1), Yadav K(1), Mahanta J(1).

Author information:
(1)1 Regional Medical Research Centre , Dibrugarh, India . (2)2 Department of Microbiology, Regional Medical Research Centre , Dibrugarh, India .

INTRODUCTION: Scrub typhus is a major reason for febrile illness, caused by a bacterium Orientia tsutsugamushi, a rickettsial pathogen. Few outbreaks of scrub typhus have been reported from Arunachal Pradesh in recent past. However, there is lack of seroprevalence data from the region. In this regard, this study was undertaken using archival serum sample available from seven districts of Arunachal Pradesh.
METHODOLOGY: This serological study was conducted in Regional Medical Research Center for NE Region, Dibrugarh. Reactivity to IgG class of antibodies against scrub typhus was done using Scrub typhus detect IgG ELISA kit as per manufacturer's protocol.
RESULT: Seroprevalence of scrub typhus in seven districts of Arunachal Pradesh was found to be 40% (120/300). The age-specific scrub typhus seroprevalence rose steadily from 5.6% in children <10 years of age to 61.8% in persons aged ≥40 years ($p = 0.0001$). Prevalence is lowest in Papumpare (25.9%) and highest in East Siang (72.5%) ($p = 0.0001$). The seroprevalence in males and females was very similar, however, the female prevalence increases from age group ≥30 years ($p = 0.053$). Moreover, among the farmers, the seroprevalence is higher (58.3%) ($p = 0.0001$).
CONCLUSIONS: As clinical symptoms overlap with other viral/bacterial infections, scrub typhus infection should be considered in differential diagnosis of any acute febrile illness in this part of the country. In view of the high prevalence, empirical therapy of doxycycline/azithromycin may be done in cases of undiagnosed fever. Active surveillance has to be done to understand exact magnitude, epidemiological aspects, and distribution of vector and disease of this reemerging neglected tropical disease.

DOI: 10.1089/vbz.2016.1970
PMID: 27536803 [PubMed - as supplied by publisher]

12. Clin Neuroradiol. 2015 Dec;25(4):415-8. doi: 10.1007/s00062-014-0348-9. Epub 2014

Nov 6.

Hemorrhagic Transformation of Scrub Typhus Encephalitis: A Rare Entity.

Kim HC(1), Yoon KW(1), Yoo DS(1,)(2), Cho CS(3).

Author information:
(1)Department of Neurosurgery, Dankook University College of Medicine, 201 Manghyang-ro Dongnam-gu, 330-715, Cheonan, Chungnam, Republic of Korea. (2)Department of Radiology, Dankook University College of Medicine, Cheonan, Chungnam, Republic of Korea. (3)Department of Neurosurgery, Dankook University College of Medicine, 201 Manghyang-ro Dongnam-gu, 330-715, Cheonan, Chungnam, Republic of Korea. babyface@dankook.ac.kr.

Central nervous system (CNS) involvement of scrub typhus infection is well known. Most CNS involvement of scrub typhus infection present as meningitis or encephalitis. We report on a patient suffering from hemorrhagic transformation of intracranial lesions caused by Orientia tsutsugamushi. A 53-year-old female farmer who was infected by scrub typhus was treated with doxycycline and recovered from the systemic illness. However, headache persisted. Brain radiologic studies revealed acute intracranial hemorrhage and enhancing lesion, which implied a CNS involvement. Hemorrhagic transformation of encephalitis by scrub typhus is very rare complication and to our best knowledge, this is the first report of hemorrhagic transformation of scrub typhus encephalitis. Clinician should consider the possibility of hemorrhagic transformation of encephalitis in cases of scrub typhus infection.

DOI: 10.1007/s00062-014-0348-9
PMID: 25373351 [PubMed - indexed for MEDLINE]

13. PLoS Negl Trop Dis. 2015 May 22;9(5):e0003814. doi: 10.1371/journal.pntd.0003814. eCollection 2015.

Urbanization of scrub typhus disease in South Korea.

Park SW(1), Ha NY(2), Ryu B(3), Bang JH(4), Song H(2), Kim Y(2), Kim G(2), Oh MD(5), Cho NH(2), Lee JK(6).

Author information:
(1)Department of Internal Medicine, Seoul National University College of Medicine, Seoul, Republic of Korea; Department of Internal Medicine, Boramae Medical Center, Seoul, Republic of Korea. (2)Department of Microbiology and Immunology, Seoul National University College of Medicine, Seoul, Republic of Korea; Department of Biomedical Science, Seoul National University College of Medicine and Bundang Hospital, Seoul, Republic of Korea. (3)Medical Research Center, Seoul National University College of Medicine, Seoul, Republic of Korea; Seoul Center for Infectious Disease Control, Seoul, Republic of Korea. (4)Department of Internal Medicine, Boramae Medical Center, Seoul, Republic of Korea; Seoul Center for Infectious Disease Control, Seoul, Republic of Korea. (5)Department of Internal Medicine, Seoul National University College of Medicine, Seoul, Republic of Korea; Seoul Center for Infectious Disease Control, Seoul, Republic of Korea. (6)Seoul Center for Infectious Disease Control, Seoul, Republic of Korea; Department of Family Medicine, Seoul National University

College of Medicine, Seoul, Republic of Korea.

BACKGROUND: Scrub typhus is an endemic disease in Asia. It has been a rural disease, but indigenous urban cases have been observed in Seoul, South Korea. Urban scrub typhus may have a significant impact because of the large population.
METHODS: Indigenous urban scrub typhus was epidemiologically identified in Seoul, the largest metropolitan city in South Korea, using national notifiable disease data from 2010 to 2013. For detailed analysis of clinical features, patients from one hospital that reported the majority of cases were selected and compared to a historic control group. Chigger mites were prospectively collected in the city using a direct chigger mite-collecting trap, and identified using both phenotypic and 18S rDNA sequencing analyses. Their infection with Orientia tsutsugamushi was confirmed by sequencing the 56-kDa antigen gene.
RESULTS: Eighty-eight cases of urban scrub typhus were determined in Seoul. The possible sites of infection were mountainous areas (56.8%), city parks (20.5%), the vicinity of one's own residence (17.0%), and riversides (5.7%). Eighty-seven chigger mites were collected in Gwanak mountain, one of the suspected infection sites in southern Seoul, and seventy-six (87.4%) of them were identified as Helenicula miyagawai and eight (9.2%) as Leptotrombidium scutellare. Pooled DNA extracted from H. miyagawai mites yielded O. tsutsugamushi Boryong strain. Twenty-six patients from one hospital showed low APACHE II score (3.4 ± 2.7), low complication rate (3.8%), and no hypokalemia.
CONCLUSIONS: We identified the presence of indigenous urban scrub typhus in Seoul, and a subgroup of them had mild clinical features. The chigger mite H. miyagawai infected with O. tsutsugamushi within the city was found. In endemic area, urban scrub typhus needs to be considered as one of the differential febrile diseases and a target for prevention.

DOI: 10.1371/journal.pntd.0003814
PMCID: PMC4441427
PMID: 26000454 [PubMed - indexed for MEDLINE]

14. Am J Trop Med Hyg. 2016 Mar;94(3):532-6. doi: 10.4269/ajtmh.15-0663. Epub 2015 Dec 28.

Rapid Increase in Scrub Typhus Incidence in Mainland China, 2006-2014.

Wu YC(1), Qian Q(1), Magalhaes RJ(1), Han ZH(1), Haque U(1), Weppelmann TA(1), Hu WB(1), Liu YX(1), Sun YS(1), Zhang WY(2), Li SL(2).

Author information:
(1)Institute of Disease Control and Prevention Academy of Military Medical Science, Beijing, People's Republic of China; School of Veterinary Science, Centre for Children's Health Research, University of Queensland, Brisbane, Australia; Navy General Hospital of People's Liberation Army, Beijing, People's Republic of China; Emerging Pathogens Institute, University of Florida, Gainesville, Florida; Department of Environmental and Global Health, College of Public Health and Health Professions, University of Florida, Gainesville, Florida; School of Public Health and Social Work, Queensland University of Technology, Brisbane, Australia; Chinese People's Liberation Army General Hospital, Beijing, People's Republic of China; Academy of Military Medical Science, Beijing, People's Republic of China. (2)Institute of Disease Control and Prevention Academy of Military Medical Science, Beijing, People's Republic of

China; School of Veterinary Science, Centre for Children's Health Research, University of Queensland, Brisbane, Australia; Navy General Hospital of People's Liberation Army, Beijing, People's Republic of China; Emerging Pathogens Institute, University of Florida, Gainesville, Florida; Department of Environmental and Global Health, College of Public Health and Health Professions, University of Florida, Gainesville, Florida; School of Public Health and Social Work, Queensland University of Technology, Brisbane, Australia; Chinese People's Liberation Army General Hospital, Beijing, People's Republic of China; Academy of Military Medical Science, Beijing, People's Republic of China zwy0419@126.com lishenlong@sohu.com.

Scrub typhus is a vector-borne disease, which has recently reemerged in China. In this study, we describe the distribution and incidence of scrub typhus cases in China from 2006 to 2014 and quantify differences in scrub typhus cases with respect to sex, age, and occupation. The results of our study indicate that the annual incidence of scrub typhus has increased during the study period. The number of cases peaked in 2014, which was 12.8 times greater than the number of cases reported in 2006. Most (77.97%) of the cases were reported in five provinces (Guangdong, Yunnan, Anhui, Fujian, and Shandong). Our study also demonstrates that the incidence rate of scrub typhus was significantly higher in females compared to males ($P < 0.001$) and was highest in the 60-69 year age group, and that farmers had a higher incidence rate than nonfarmers ($P < 0.001$). Different seasonal trends were identified in the number of reported cases between the northern and southern provinces of China. These findings not only demonstrate that China has experienced a large increase in scrub typhus incidence, but also document an expansion in the geographic distribution throughout the country.

© The American Society of Tropical Medicine and Hygiene.

DOI: 10.4269/ajtmh.15-0663
PMCID: PMC4775887 [Available on 2017-03-02]
PMID: 26711517 [PubMed - indexed for MEDLINE]

15. PLoS Negl Trop Dis. 2015 Dec 17;9(12):e0004161. doi: 10.1371/journal.pntd.0004161. eCollection 2015.

A Spatiotemporal Database to Track Human Scrub Typhus Using the VectorMap Application.

Kelly DJ(1,)(2), Foley DH(3), Richards AL(1,)(4).

Author information:
(1)Viral and Rickettsial Diseases Department, Naval Medical Research Center, Silver Spring, Maryland, United States of America. (2)Department of Evolution, Ecology and Organismal Biology, The Ohio State University, Columbus, Ohio, United States of America. (3)Division of Entomology, Walter Reed Army Institute of Research, Silver Spring, Maryland, United States of America. (4)Preventive Medicine and Biometrics Department, Uniformed Services University of the Health Sciences, Bethesda, Maryland, United States of America.

Scrub typhus is a potentially fatal mite-borne febrile illness, primarily of the Asia-Pacific Rim. With an endemic area greater than 13 million km2 and millions of people at risk, scrub typhus remains an underreported, often misdiagnosed

febrile illness. A comprehensive, updatable map of the true distribution of cases has been lacking, and therefore the true risk of disease within the very large endemic area remains unknown. The purpose of this study was to establish a database and map to track human scrub typhus. An online search using PubMed and the United States Armed Forces Pest Management Board Literature Retrieval System was performed to identify articles describing human scrub typhus cases both within and outside the traditionally accepted endemic regions. Using World Health Organization guidelines, stringent criteria were used to establish diagnoses for inclusion in the database. The preliminary screening of 181 scrub typhus publications yielded 145 publications that met the case criterion, 267 case records, and 13 serosurvey records that could be georeferenced, describing 13,739 probable or confirmed human cases in 28 countries. A map service has been established within VectorMap (www.vectormap.org) to explore the role that relative location of vectors, hosts, and the pathogen play in the transmission of mite-borne scrub typhus. The online display of scrub typhus cases in VectorMap illustrates their presence and provides an up-to-date geographic distribution of proven scrub typhus cases.

DOI: 10.1371/journal.pntd.0004161
PMCID: PMC4683066
PMID: 26678263 [PubMed - indexed for MEDLINE]

16. J Korean Med Sci. 2015 Nov;30(11):1698-700. doi: 10.3346/jkms.2015.30.11.1698. Epub 2015 Oct 16.

Acute Cholecystitis in Patients with Scrub Typhus.

Lee H(1), Ji M(2), Hwang JH(3), Lee JY(3), Lee JH(4), Chung KM(5), Lee CS(6).

Author information:
(1)Division of Pulmonary and Critical Care Medicine, Department of Medicine, Samsung Medical Center, Sungkyunkwan University School of Medicine, Seoul, Korea. (2)Department of Laboratory Medicine, Konkuk University School of Medicine, Seoul, Korea. (3)Department of Internal Medicine, Chonbuk National University Hospital, Jeonju, Korea. (4)Department of Preventive Medicine, Chonbuk National University Hospital, Jeonju, Korea. (5)Department of Microbiology, Chonbuk National University Hospital, Jeonju, Korea. (6)Department of Internal Medicine, Chonbuk National University Hospital, Jeonju, Korea. ; Research Institute of Clinical Medicine of Chonbuk National University-Chonbuk National University Hospital, Jeonju, Korea.

Acute cholecystitis is a rare complication of scrub typhus. Although a few such cases have been reported in patients with scrub typhus, the clinical course is not well described. Of 12 patients, acute cholecystitis developed in 66.7% (8/12) of patients older than 60 yr. The scrub typhus group with acute cholecystitis had marginal significant longer hospital stay and higher cost than the group without cholecystitis according to propensity score matching. Scrub typhus should be kept in mind as a rare etiology of acute cholecystitis in endemic areas because the typical signs of scrub typhus such as skin rash and eschar can present after the abdominal pain.

DOI: 10.3346/jkms.2015.30.11.1698
PMCID: PMC4630489

PMID: 26539017 [PubMed - indexed for MEDLINE]

17. PLoS Negl Trop Dis. 2015 Aug 28;9(8):e0004024. doi: 10.1371/journal.pntd.0004024. eCollection 2015.

The Diversity and Geographical Structure of Orientia tsutsugamushi Strains from Scrub Typhus Patients in Laos.

Phetsouvanh R(1), Sonthayanon P(2), Pukrittayakamee S(3), Paris DH(4), Newton PN(1), Feil EJ(5), Day NP(4).

Author information:
(1)Lao-Oxford-Mahosot Hospital-Wellcome Trust Research Unit, Microbiology Laboratory, Mahosot Hospital, Vientiane, Laos PDR. (2)Department of Molecular Tropical Medicine and Genetics, Faculty of Tropical Medicine, Mahidol University, Bangkok, Thailand; Mahidol-Oxford Tropical Medicine Research Unit, Faculty of Tropical Medicine, Mahidol University, Bangkok, Thailand. (3)Department of Clinical Tropical Medicine, Faculty of Tropical Medicine, Mahidol University, Bangkok, Thailand. (4)Mahidol-Oxford Tropical Medicine Research Unit, Faculty of Tropical Medicine, Mahidol University, Bangkok, Thailand; Centre for Tropical Medicine & Global Health, Nuffield Department of Clinical Medicine, University of Oxford, Oxford, United Kingdom. (5)Department of Biology and Biochemistry, University of Bath, Bath, United Kingdom.

Erratum in
PLoS Negl Trop Dis. 2016 May;10(5):e0004742.

Orientia tsutsugamushi is the causative agent of scrub typhus, a disease transmitted by Leptotrombidium mites which is responsible for a severe and under-reported public health burden throughout Southeast Asia. Here we use multilocus sequence typing (MLST) to characterize 74 clinical isolates from three geographic locations in the Lao PDR (Laos), and compare them with isolates described from Udon Thani, northeast Thailand. The data confirm high levels of diversity and recombination within the natural O. tsutsugamushi population, and a rate of mixed infection of ~8%. We compared the relationships and geographical structuring of the strains and populations using allele based approaches (eBURST), phylogenetic approaches, and by calculating F-statistics (FST). These analyses all point towards low levels of population differentiation between isolates from Vientiane and Udon Thani, cities which straddle the Mekong River which defines the Lao/Thai border, but with a very distinct population in Salavan, southern Laos. These data highlight how land use, as well as the movement of hosts and vectors, may impact on the epidemiology of zoonotic infections.

DOI: 10.1371/journal.pntd.0004024
PMCID: PMC4552799
PMID: 26317624 [PubMed - indexed for MEDLINE]

18. Am J Trop Med Hyg. 2015 May;92(5):972-8. doi: 10.4269/ajtmh.14-0806. Epub 2015 Mar 16.

Clinical and Epidemiological Characteristics of Scrub Typhus and Murine Typhus

among Hospitalized Patients with Acute Undifferentiated Fever in Northern Vietnam.

Hamaguchi S(1), Cuong NC(1), Tra DT(1), Doan YH(1), Shimizu K(1), Tuan NQ(1), Yoshida LM(1), Mai le Q(1), Duc-Anh D(1), Ando S(1), Arikawa J(1), Parry CM(1), Ariyoshi K(1), Thuy PT(2).

Author information:
(1)Department of Clinical Tropical Medicine, Institute of Tropical Medicine, Graduate School of Biomedical Sciences, Nagasaki University, Nagasaki, Japan; Infectious Disease Department, Bach Mai Hospital, Hanoi, Vietnam; Departments of Virology I, National Institute of Infectious Diseases, Tokyo, Japan; Laboratory of Infectious Diseases, Department of Microbiology, Hokkaido University Graduate School of Medicine, Hokkaido, Japan; National Institute of Hygiene and Epidemiology, Hanoi, Vietnam; Department of Clinical Research, London School of Hygiene and Tropical Medicine, London, United Kingdom. (2)Department of Clinical Tropical Medicine, Institute of Tropical Medicine, Graduate School of Biomedical Sciences, Nagasaki University, Nagasaki, Japan; Infectious Disease Department, Bach Mai Hospital, Hanoi, Vietnam; Departments of Virology I, National Institute of Infectious Diseases, Tokyo, Japan; Laboratory of Infectious Diseases, Department of Microbiology, Hokkaido University Graduate School of Medicine, Hokkaido, Japan; National Institute of Hygiene and Epidemiology, Hanoi, Vietnam; Department of Clinical Research, London School of Hygiene and Tropical Medicine, London, United Kingdom phamthanhthuy714@gmail.com.

A descriptive study on rickettsiosis was conducted at the largest referral hospital in Hanoi, Vietnam, to identify epidemiological and clinical characteristics of specific rickettsiosis. Between March 2001 and February 2003, we enrolled 579 patients with acute undifferentiated fever (AUF), excluding patients with malaria, dengue fever, and typhoid fever, and serologically tested for Orientia tsutsugamushi and Rickettsia typhi. Of the patients, 237 (40.9%) and 193 (33.3%) had scrub and murine typhus, respectively, and 149 (25.7%) had neither of them (non-scrub and murine typhus [non-ST/MT]). The proportion of murine typhus was highest among patients living in Hanoi whereas that of scrub typhus was highest in national or regional border areas. The presence of an eschar, dyspnea, hypotension, and lymphadenopathy was significantly associated with a diagnosis of scrub typhus (OR = 46.56, 10.90, 9.01, and 7.92, respectively). Patients with murine typhus were less likely to have these findings but more likely to have myalgia, rash, and relative bradycardia (OR = 1.60, 1.56, and 1.45, respectively). Scrub typhus and murine typhus were shown to be common causes of AUF in northern Vietnam although the occurrence of spotted fever group rickettsiae was not determined. Clinical and epidemiological information may help local clinicians make clinical diagnosis of specific rickettsioses in a resource-limited setting.

© The American Society of Tropical Medicine and Hygiene.

DOI: 10.4269/ajtmh.14-0806
PMCID: PMC4426587
PMID: 25778504 [PubMed - indexed for MEDLINE]

19. Epidemiol Infect. 2015 Aug;143(11):2451-8. doi: 10.1017/S0950268814003598. Epub 2014 Dec 29.

Epidemic characteristics and spatio-temporal patterns of scrub typhus during 2006-2013 in Tai'an, Northern China.

Zheng L(1), Yang HL(2), Bi ZW(3), Kou ZQ(3), Zhang LY(1), Zhang AH(2), Yang L(1), Zhao ZT(1).

Author information:
(1)Department of Epidemiology and Health Statistics,School of Public Health,Shandong University,Jinan,China. (2)Tai'an Center for Disease Control and Prevention,Tai'an,China. (3)Institute for Bacterial Infectious Disease Control and Prevention,Shandong Center for Disease Control and Prevention,Jinan,China.

Tai'an, a famous cultural tourist district, is a new endemic foci of scrub typhus in northern China. Frequent reports of travel-acquired cases and absence of effective vaccine indicated a significant health problem of scrub typhus in Tai'an. Thus, descriptive epidemiological methods and spatial-temporal scan statistics were used to describe the epidemic characteristics and detect the significant clusters of the high incidence of scrub typhus at the town level in Tai'an. Results of descriptive epidemiological analysis showed a total of 490 cases were reported in Tai'an with the annual average incidence ranging from 0·48 to 2·27/100 000 during 2006-2013. Females, the elderly and farmers are the high-risk groups. Monthly changes of scrub typhus cases indicated an obvious epidemic period in autumn. Spatial-temporal distribution analysis, showed significant clusters of high incidence mainly located in eastern and northern Tai'an. Our study suggests that more effective, targeted measures for local residents should be implemented in the eastern and northern areas of Tai'an in autumn. Meanwhile, it may prove beneficial for health policy makers to advise travellers to take preventive measures in order to minimize the risk of infection of scrub typhus in Tai'an.

DOI: 10.1017/S0950268814003598
PMID: 25543665 [PubMed - indexed for MEDLINE]

20. Trans R Soc Trop Med Hyg. 2014 Nov;108(11):739-40. doi: 10.1093/trstmh/tru145. Epub 2014 Sep 23.

Scrub typhus in the northern provinces of Vietnam: an observational study of admissions to a national referral hospital.

Nadjm B(1), Thuy PT(2), Trang VD(3), Ha le D(2), Kinh NV(3), Wertheim HF(4).

Author information:
(1)Oxford University Clinical Research Unit, Hanoi, Vietnam bnadjm@oucru.org. (2)Bach Mai Hospital, Hanoi, Vietnam. (3)National Hospital of Tropical Diseases, Hanoi, Vietnam. (4)Oxford University Clinical Research Unit, Hanoi, Vietnam.

BACKGROUND: Scrub typhus is a common cause of fever in parts of South East and Southern Asia. Little is known about the disease burden in Vietnam.
METHODS: A 2-year observational study of scrub typhus at a tertiary referral hospital in northern Vietnam was carried out. Diagnosis was based on a single serological test in patients with suggestive clinical symptoms.
RESULTS: Scrub typhus was diagnosed in 3.5% (251/7226) of admissions. Cases

occurred throughout the year, with incidence highest in the summer. Although complications were common, mortality was low (1.2%; 3/251).
CONCLUSIONS: These data suggest that scrub typhus is common, with a seasonal distribution in northern Vietnam.

© The Author 2014. Published by Oxford University Press on behalf of Royal Society of Tropical Medicine and Hygiene.

DOI: 10.1093/trstmh/tru145
PMCID: PMC4191438
PMID: 25253616 [PubMed - indexed for MEDLINE]

21. Emerg Infect Dis. 2015 Apr;21(4):688-91. doi: 10.3201/eid2104.141387.

High seroprevalence of antibodies against spotted fever and scrub typhus bacteria in patients with febrile Illness, Kenya.

Thiga JW, Mutai BK, Eyako WK, Ng'ang'a Z, Jiang J, Richards AL, Waitumbi JN.

Serum samples from patients in Kenya with febrile illnesses were screened for antibodies against bacteria that cause spotted fever, typhus, and scrub typhus. Seroprevalence was 10% for spotted fever group, <1% for typhus group, and 5% for scrub typhus group. Results should help clinicians expand their list of differential diagnoses for undifferentiated fevers.

DOI: 10.3201/eid2104.141387
PMCID: PMC4378494
PMID: 25811219 [PubMed - indexed for MEDLINE]

22. Medicine (Baltimore). 2016 Feb;95(8):e2928. doi: 10.1097/MD.0000000000002928.

Successful Treatment of Scrub Typhus-Associated Hemophagocytic Lymphohistiocytosis With Chloramphenicol: Report of 3 Pediatric Cases and Literature Review.

Zhou YH(1), Xia FQ, Van Poucke S, Zheng MH.

Author information:
(1)From the Department of Pediatrics (Y-HZ, F-QX), The Second Affiliated Hospital & Yuying Children's Hospital of Wenzhou Medical University, Wenzhou, Zhejiang, China; Department of Anesthesiology (SVP), Intensive Care, Emergency Medicine and Pain Therapy, Ziekenhuis Oost-Limburg, Genk, Belgium; Department of Infection and Liver Diseases (M-HZ), Liver Research Center, The First Affiliated Hospital of Wenzhou Medical University; and Institute of Hepatology (M-HZ), Wenzhou Medical University, Wenzhou, China.

Scrub typhus is caused by Orientia tsutsugamushi. Any delay in diagnosis can result in delayed treatment and severe complications, including secondary hemophagocytic lymphohistiocytosis, which is rare but potentially fatal.In this paper, the authors present 3 cases of secondary hemophagocytic lymphohistiocytosis associated with scrub typhus, successfully treated with chloramphenicol without additional antineoplastic therapy. All patients cured and

achieved complete resolution.This report highlights the effectiveness of chloramphenicol without the need for chemotherapy in the treatment of scrub typhus-associated hemophagocytic lymphohistiocytosis in a pediatric population under the age of 8 years.

DOI: 10.1097/MD.0000000000002928
PMCID: PMC4779037
PMID: 26937940 [PubMed - indexed for MEDLINE]

23. Int J Infect Dis. 2014 Dec;29:203-7. doi: 10.1016/j.ijid.2014.09.019. Epub 2014 Nov 6.

Risk factors associated with severe scrub typhus in Shandong, northern China.

Zhang L(1), Zhao Z(2), Bi Z(3), Kou Z(3), Zhang M(1), Yang L(1), Zheng L(1).

Author information:
(1)Department of Epidemiology and Health Statistics, School of Public Health, Shandong University, 44 Wenhuaxi Road, Jinan, Shandong 250012, China.
(2)Department of Epidemiology and Health Statistics, School of Public Health, Shandong University, 44 Wenhuaxi Road, Jinan, Shandong 250012, China. Electronic address: ztzhao@sdu.edu.cn. (3)Institute for Bacterial Infectious Disease Control and Prevention, Shandong Center for Disease Control and Prevention, Jinan, China.

OBJECTIVES: The aim of this study was to identify risk factors associated with severe scrub typhus, in order to provide a reference for clinical decision-making.
METHODS: A case-control study was conducted of scrub typhus patients who presented at local hospitals between 2010 and 2013. In total, 46 patients with severe scrub typhus complications (cases) and 194 without severe complications (controls) were included.
RESULTS: There were significant differences in the duration of illness before effective antibiotic therapy, lymphadenopathy, rash, blood platelet count, white blood cell (WBC) count, percentage neutrophils, and percentage lymphocytes between the case and control groups. Multivariate analysis demonstrated that the following four factors were significantly associated with the severe complications of scrub typhus: (1) duration of illness before effective antibiotic therapy (odds ratio (OR) 2.287, 95% confidence interval (CI) 1.096-4.770); (2) the presence of a rash (OR 3.694, 95% CI 1.300-10.495); (3) lymphadenopathy (OR 2.438, 95% CI 1.090-5.458); (4) blood platelet count $<100\times10^{9}$/l (OR 2.226, 95% CI 1.002-4.946).
CONCLUSIONS: This study indicates that improved diagnosis and timely treatment are important factors for the prevention of severe scrub typhus. When scrub typhus patients present with a rash, lymphadenopathy, or blood platelet count $<100\times10^{9}$/l, clinicians should be alert to the appearance of severe complications.

Copyright © 2014 The Authors. Published by Elsevier Ltd.. All rights reserved.

DOI: 10.1016/j.ijid.2014.09.019
PMID: 25461664 [PubMed - indexed for MEDLINE]

24. Lung India. 2016 Jul-Aug;33(4):439-43. doi: 10.4103/0970-2113.184923.

Scrub typhus infection presenting as acute heart failure: A case report and systematic review of literature of cardiopulmonary involvement in scrub typhus infection.

Ray A(1), Nangia V(1), Chatterji RS(1), Dalal N(1).

Author information:
(1)Department of Pulmonary Critical Care and Sleep Medicine, Fortis Flt Lt Rajan Dhall Hospital, New Delhi, India.

We describe a middle aged previoulsy healthy female patient who presented with clinical features suggestive of acute heart failure. Investigations revealed very high NT pro-BNP, right heart enlargement, bilateral pulmonary alveolar edema and bilateral pleural effusion. In view of falling platelet counts and exudative pleural effusion inflammatory/infective causes were considered. Her Weil Felix test was strongly positive and IgM for scrub typhus also returned positive. She was started on doxycycline to which there was dramatic improvement. Thus in this case scrub typhus infection presented as acute right heart failure and the cause seemed elusive at the outset. We also systematically reviewed the existing literature on cardio-pulmonary manifestations of scrub typhus infection.

DOI: 10.4103/0970-2113.184923
PMCID: PMC4948236
PMID: 27578941 [PubMed]

25. Int J Environ Res Public Health. 2015 Jun 29;12(7):7254-73. doi: 10.3390/ijerph120707254.

Scrub Typhus Incidence Modeling with Meteorological Factors in South Korea.

Kwak J(1), Kim S(2), Kim G(3), Singh VP(4), Hong S(5), Kim HS(6).

Author information:
(1)Forecast and Control Division, Nakdong River Flood Control Office, Busan 604-851, Korea. firstsword@korea.kr. (2)Columbia Water Center, Columbia University, New York, NY 10027, USA. soojun78@gmail.com. (3)Department of Hydro Science and Engineering, Korea Institute of Civil Engineering and Building Technology, Goyang-si, Gyeonggi-do 411-712, Korea. kgh0518@kict.re.kr. (4)Department of Biological & Agricultural Engineering and Zachry Dept. of Civil Engineering, Texas A & M University, TX 77843, USA. vsingh@tamu.edu. (5)Department of Civil Engineering, Inha University, Incheon 402-751, Korea. hongsst81@gmail.com. (6)Department of Civil Engineering, Inha University, Incheon 402-751, Korea. sookim@inha.ac.kr.

Since its recurrence in 1986, scrub typhus has been occurring annually and it is considered as one of the most prevalent diseases in Korea. Scrub typhus is a 3rd grade nationally notifiable disease that has greatly increased in Korea since 2000. The objective of this study is to construct a disease incidence model for prediction and quantification of the incidences of scrub typhus. Using data from 2001 to 2010, the incidence Artificial Neural Network (ANN) model, which considers the time-lag between scrub typhus and minimum temperature,

precipitation and average wind speed based on the Granger causality and spectral analysis, is constructed and tested for 2011 to 2012. Results show reliable simulation of scrub typhus incidences with selected predictors, and indicate that the seasonality in meteorological data should be considered.

DOI: 10.3390/ijerph120707254
PMCID: PMC4515655
PMID: 26132479 [PubMed - indexed for MEDLINE]

26. Indian J Med Microbiol. 2015 Jan-Mar;33(1):68-72. doi: 10.4103/0255-0857.148381.

Seroprevalence of Scrub typhus at a tertiary care hospital in Andhra Pradesh.

Ramyasree A, Kalawat U(1), Rani ND, Chaudhury A.

Author information:
(1)Department of Microbiology , Sri Venkateswara Institute of Medical Sciences, Tirupati, Andhra Pradesh, India.

INTRODUCTION: Scrub typhus is a rickettsial infection which is caused by Orientia tsutsugamushi and transmitted by the bite of the chigger of a mite. Delay in diagnosis can be fatal otherwise the treatment is simple, doxycycline being the drug of choice. Indirect immunoflurescence is considered gold standard but it is not used in India as it is costly and also not available. There is need for rapid, economic and simple test for the diagnosis of scrub typhus. This study was taken up to study the seroprevalence of scrub typhus in Andhra Pradesh and to compare two commonly used serological methods; rapid test and IgM ELISA.
MATERIALS AND METHODS: This is a prospective study in which 100 serum samples from clinically suspected cases collected over a period of 3 months were processed for the detection of IgM antibodies for scrub typhus by ELISA and Rapid test. Samples were also tested for leptospirosis and dengue fever which the other common causes of fever prevalent in this region.
RESULTS: Total number of samples processed was 100 of which 52 were males and 48 females. Among the hundred samples 39 were seropositive. Positivity was higher in the age group of patients between 16 and 30 yrs of age. There was 97% correlation between ELISA and rapid method. Of the 100 samples only three samples positive by ELISA were negative by rapid method. Fever was the most common manifestation and there was no eschar and no mortality reported.
CONCLUSION: Scrub typhus should be included in the differential diagnosis of fever of unknown origin along with dengue, malaria and leptospirosis which are the other common endemic infections in this part of the country.

DOI: 10.4103/0255-0857.148381
PMID: 25560004 [PubMed - indexed for MEDLINE]

27. Indian J Med Microbiol. 2014 Oct-Dec;32(4):387-90. doi: 10.4103/0255-0857.142241.

Possibility of scrub typhus in fever of unknown origin (FUO) cases: an experience from Rajasthan.

Bithu R(1), Kanodia V, Maheshwari RK.

Author information:
(1)Department of Microbiology and Immunology, Sawai Man Singh Medial College, Jaipur, Rajasthan, India.

PURPOSE: Fever of unknown origin (FUO) has multiple causes. Scrub typhus is less known cause of FUO in India. The present study reports a recent epidemic of scrub typhus amongst cases of FUO from different areas of Rajasthan, India. There was high mortality in undiagnosed cases of FUO which lead to the diagnosis of scrub typhus.
OBJECTIVE: To study the possibility of scrub typhus as a causative factor in FUO cases by qualitative detection of IgM antibodies with ELISA.
MATERIALS AND METHODS: From September 2012 to December 2012, 271 serum samples of FUO cases were analysed for IgM antibodies to Orientia tsutsugamushi along with dengue, malaria, typhoid, tuberculosis and brucellosis.
RESULTS: Scrub typhus IgM antibodies by ELISA were detected in 133 (49.1%) patients. Scrub typhus positivity was significantly higher among female in comparison to males (P<0.05). Maximum positivity of scrub typhus was found in females of 46-60 years age group. The laboratory parameters were abnormal in most of the patients as evident by thrombocytopenia (63%), deranged liver functions (56%) and renal functions (25%).
CONCLUSION: The present study emphasises the importance of scrub typhus among cases of FUO especially after rainy season and during early cooler months. The study also highlights the significance of ELISA method for rapid and early reporting and ruling out scrub typhus in FUO cases.

DOI: 10.4103/0255-0857.142241
PMID: 25297022 [PubMed - indexed for MEDLINE]

28. N Engl J Med. 2016 Sep 8;375(10):913-5. doi: 10.1056/NEJMp1608499.

Scrub Typhus - Scientific Neglect, Ever-Widening Impact.

Walker DH(1).

Author information:
(1)From the Center for Biodefense and Emerging Infectious Diseases, University of Texas Medical Branch, Galveston.

DOI: 10.1056/NEJMp1608499
PMID: 27602663 [PubMed - indexed for MEDLINE]

29. PLoS Negl Trop Dis. 2016 Aug 1;10(8):e0004875. doi: 10.1371/journal.pntd.0004875. eCollection 2016.

Spatiotemporal Dynamics of Scrub Typhus Transmission in Mainland China, 2006-2014.

Wu YC(1), Qian Q(1), Soares Magalhaes RJ(2,)(3), Han ZH(4), Hu WB(5), Haque U(6,)(7), Weppelmann TA(6,)(8), Wang Y(1), Liu YX(9), Li XL(10), Sun HL(1), Sun YS(11), Clements AC(12), Li SL(1), Zhang WY(1).

Author information:

(1)Institute of Disease Control and Prevention, Academy of Military Medical Science, Beijing, People's Republic of China. (2)Spatial Epidemiology Laboratory, School of Veterinary Science, The University of Queensland, Brisbane, Australia. (3)Child Health Research Center, The University of Queensland, Brisbane, Australia. (4)Navy General Hospital of PLA, Beijing, People's Republic of China. (5)School of Public Health and Social Work, Queensland University of Technology, Brisbane, Australia. (6)Emerging Pathogens Institute, University of Florida, Gainesville, Florida, United States of America. (7)Department of Geography, University of Florida, Gainesville, Florida, United States of America. (8)Department of Environmental and Global Health, College of Public Health and Health Professions, University of Florida, Gainesville, Florida, United States of America. (9)Chinese People's Liberation Army General Hospital, Beijing, People's Republic of China. (10)517 Military Hospital, Kelan, Shanxi, People's Republic of China. (11)Academy of Military Medical Science, Beijing, People's Republic of China. (12)Research School of Population Health, The Australian National University, Canberra, Australian Capital Territory, Australia.

BACKGROUND: Scrub typhus is endemic in the Asia-Pacific region including China, and the number of reported cases has increased dramatically in the past decade. However, the spatial-temporal dynamics and the potential risk factors in transmission of scrub typhus in mainland China have yet to be characterized.
OBJECTIVE: This study aims to explore the spatiotemporal dynamics of reported scrub typhus cases in mainland China between January 2006 and December 2014, to detect the location of high risk spatiotemporal clusters of scrub typhus cases, and identify the potential risk factors affecting the re-emergence of the disease.
METHOD: Monthly cases of scrub typhus reported at the county level between 2006 and 2014 were obtained from the Chinese Center for Diseases Control and Prevention. Time-series analyses, spatiotemporal cluster analyses, and spatial scan statistics were used to explore the characteristics of the scrub typhus incidence. To explore the association between scrub typhus incidence and environmental variables panel Poisson regression analysis was conducted.
RESULTS: During the time period between 2006 and 2014 a total of 54,558 scrub typhus cases were reported in mainland China, which grew exponentially. The majority of cases were reported each year between July and November, with peak incidence during October every year. The spatiotemporal dynamics of scrub typhus varied over the study period with high-risk clusters identified in southwest, southern, and middle-eastern part of China. Scrub typhus incidence was positively correlated with the percentage of shrub and meteorological variables including temperature and precipitation.
CONCLUSIONS: The results of this study demonstrate areas in China that could be targeted with public health interventions to mitigate the growing threat of scrub typhus in the country.

DOI: 10.1371/journal.pntd.0004875
PMCID: PMC4968795
PMID: 27479297 [PubMed - in process]

30. J Infect. 2014 Nov;69(5):462-9. doi: 10.1016/j.jinf.2014.06.018. Epub 2014 Jul 1.

Increased endothelial and macrophage markers are associated with disease severity and mortality in scrub typhus.

Otterdal K(1), Janardhanan J(2), Astrup E(3), Ueland T(4), Prakash JA(5), Lekva T(6), Abraham OC(2), Thomas K(7), Damås JK(8), Mathews P(7), Mathai D(7), Aukrust P(9), Varghese GM(2).

Author information:
(1)Research Institute for Internal Medicine, Oslo University Hospital Rikshospitalet, Sognsvannsveien 20, 0027 Oslo, Norway; Faculty of Medicine, University of Oslo, PO Box 1072 Blindern, 0316 Oslo, Norway. Electronic address: kari.otterdal@rr-research.no. (2)Department of Medicine and Infectious Diseases, Christian Medical College, Vellore 632 004, Tamil Nadu, India. (3)Research Institute for Internal Medicine, Oslo University Hospital Rikshospitalet, Sognsvannsveien 20, 0027 Oslo, Norway; Institute of Clinical Medicine, Akershus University Hospital, Sykehusveien 25, 1478 Lørenskog, Norway. (4)Research Institute for Internal Medicine, Oslo University Hospital Rikshospitalet, Sognsvannsveien 20, 0027 Oslo, Norway; Faculty of Medicine, University of Oslo, PO Box 1072 Blindern, 0316 Oslo, Norway. (5)Department of Microbiology, Christian Medical College, Vellore 632 004, Tamil Nadu, India. (6)Research Institute for Internal Medicine, Oslo University Hospital Rikshospitalet, Sognsvannsveien 20, 0027 Oslo, Norway; Section of Specialized Endocrinology, Department of Endocrinology, Oslo University Hospital Rikshospitalet, Sognsvannsveien 20, 0027 Oslo, Norway; Faculty of Medicine, University of Oslo, PO Box 1072 Blindern, 0316 Oslo, Norway. (7)Department of Medicine, Christian Medical College, Vellore 632 004, Tamil Nadu, India. (8)Centre of Molecular Inflammation Research, Department of Cancer Research and Molecular Medicine, St. Olavs Hospital, PO Box 3250 Sluppen, 7006 Trondheim, Norway; Norwegian University of Science and Technology, PO Box 8905, 7491 Trondheim, Norway. (9)Research Institute for Internal Medicine, Oslo University Hospital Rikshospitalet, Sognsvannsveien 20, 0027 Oslo, Norway; Section of Clinical Immunology and Infectious Diseases, Oslo University Hospital Rikshospitalet, Sognsvannsveien 20, 0027 Oslo, Norway; Faculty of Medicine, University of Oslo, PO Box 1072 Blindern, 0316 Oslo, Norway.

OBJECTIVES: Scrub typhus is endemic in the Asia-Pacific region. Mortality is high even with treatment, and further knowledge of the immune response during this infection is needed. This study was aimed at comparing plasma levels of monocyte/macrophage and endothelial related inflammatory markers in patients and controls in South India and to explore a possible correlation to disease severity and clinical outcome.
METHODS: Plasma levels of ALCAM, VCAM-1, sCD163, sCD14, YKL-40 and MIF were measured in scrub typhus patients (n = 129), healthy controls (n = 31) and in infectious disease controls (n = 31), both in the acute phase and after recovery, by enzyme immunoassays.
RESULTS: Patients had markedly elevated levels of all mediators in the acute phase, differing from both healthy and infectious disease controls. During follow-up levels of ALCAM, VCAM-1, sCD14 and YKL-40 remained elevated compared to levels in healthy controls. High plasma ALCAM, VCAM-1, sCD163, sCD14, and MIF, and in particular YKL-40 were all associated with disease severity and ALCAM, sCD163, MIF and especially YKL-40, were associated with mortality.
CONCLUSIONS: Our findings show that scrub typhus is characterized by elevated levels of monocyte/macrophage and endothelial related markers. These inflammatory markers, and in particular YKL-40, may contribute to disease severity and clinical outcome.

Copyright © 2014 The British Infection Association. Published by Elsevier Ltd. All rights reserved.

DOI: 10.1016/j.jinf.2014.06.018
PMID: 24995849 [PubMed - indexed for MEDLINE]

31. Int J Infect Dis. 2014 Jun;23:39-43. doi: 10.1016/j.ijid.2014.02.009. Epub 2014 Mar 21.

Clinical profile and improving mortality trend of scrub typhus in South India.

Varghese GM(1), Trowbridge P(2), Janardhanan J(3), Thomas K(3), Peter JV(4), Mathews P(5), Abraham OC(3), Kavitha ML(6).

Author information:
(1)Medicine Unit I and Infectious Diseases, Christian Medical College, Vellore, Tamil Nadu, India. Electronic address: georgemvarghese@hotmail.com. (2)Department of Infectious Diseases, Tufts University, Boston, USA. (3)Medicine Unit I and Infectious Diseases, Christian Medical College, Vellore, Tamil Nadu, India. (4)Department of Critical Care, Christian Medical College, Vellore, Tamil Nadu, India. (5)Department of Medicine, Christian Medical College, Vellore, Tamil Nadu, India. (6)Department of Hematology, Christian Medical College, Vellore, Tamil Nadu, India.

BACKGROUND: Scrub typhus, a bacterial zoonosis caused by Orientia tsutsugamushi, may cause multiorgan dysfunction syndrome (MODS) and is associated with significant mortality. This study was undertaken to document the clinical and laboratory manifestations and complications and to study time trends and factors associated with mortality in patients with scrub typhus infection.
METHODS: This retrospective study, done at a university teaching hospital, included 623 patients admitted between 2005 and 2010 with scrub typhus. The diagnosis was established by a positive IgM ELISA and/or pathognomonic eschar with PCR confirmation where feasible. The clinical and laboratory profile, course in hospital, and outcome were documented. Factors associated with mortality were analyzed using multivariate logistic regression analysis.
RESULTS: The most common presenting symptoms were fever (100%), nausea/vomiting (54%), shortness of breath (49%), headache (46%), cough (38%), and altered sensorium (26%). An eschar was present in 43.5% of patients. Common laboratory findings included elevated transaminases (87%), thrombocytopenia (79%), and leukocytosis (46%). MODS was seen in 34% of patients. The overall case-fatality rate was 9.0%. Features of acute lung injury were observed in 33.7%, and 29.5% required ventilatory support. On multivariate analysis, shock requiring vasoactive agents (relative risk (RR) 10.5, 95% confidence interval (CI) 4.2-25.7, $p<0.001$), central nervous system (CNS) dysfunction (RR 5.1, 95% CI 2.4-10.7, $p<0.001$), and renal failure (RR 3.6, 95% CI 1.7-7.5, $p=0.001$) were independent predictors of mortality. Over 4 years, a decreasing trend was observed in the mortality rate.
CONCLUSIONS: Scrub typhus can manifest with potentially life-threatening complications such as lung injury, shock, and meningoencephalitis. MODS occurred in a third of our patients. The overall case-fatality rate was 9%, with shock, renal failure, and CNS associated with a higher mortality.

Copyright © 2014 The Authors. Published by Elsevier Ltd.. All rights reserved.

DOI: 10.1016/j.ijid.2014.02.009

PMID: 24661931 [PubMed - indexed for MEDLINE]

32. J Infect Public Health. 2015 Nov-Dec;8(6):626-9. doi: 10.1016/j.jiph.2015.05.012.
Epub 2015 Jun 27.

Hemophagocytic lymphohistiocytosis with a leukemoid reaction in an infant with scrub typhus.

Pazhaniyandi S(1), Lenin R(2), Sivathanu S(2).

Author information:
(1)Department of Paediatrics, ESIC Medical College & PGIMSR, K.K. Nagar, Chennai 600 078, Tamil Nadu, India. Electronic address: drsunithapazhani@gmail.com.
(2)Department of Paediatrics, ESIC Medical College & PGIMSR, K.K. Nagar, Chennai 600 078, Tamil Nadu, India.

A two-month-old male infant presented with a clinical picture suggestive of fever without focus. Treatment was initiated with broad spectrum intravenous antibiotics. The infant subsequently developed septic shock, hepatosplenomegaly and tachypnoea with oxygen dependency. There was laboratory evidence of anemia, thrombocytopenia and a leukemoid reaction. Infection induced hemophagocytic lymphohistiocytosis with a leukemoid reaction was suspected because of fever, splenomegaly and bicytopenia in conjunction with elevated serum triglycerides and ferritin. Empiric therapy with doxycycline caused a rapid resolution of the fever, and the diagnosis of scrub typhus was confirmed by a positive scrub IgM ELISA. HLH with a leukemoid reaction secondary to scrub typhus has not previously been reported in early infancy.

Copyright © 2015 King Saud Bin Abdulaziz University for Health Sciences.
Published by Elsevier Ltd. All rights reserved.

DOI: 10.1016/j.jiph.2015.05.012
PMID: 26123173 [PubMed - indexed for MEDLINE]

33. Am J Trop Med Hyg. 2016 Jan;94(1):22-5. doi: 10.4269/ajtmh.15-0474. Epub 2015 Oct 26.

Effect of Latitude and Seasonal Variation on Scrub Typhus, South Korea, 2001-2013.

Jeung YS(1), Kim CM(1), Yun NR(1), Kim SW(1), Han MA(1), Kim DM(2).

Author information:
(1)School of Medicine, Chosun University, Gwangju, South Korea; Premedical Science, College of Medicine, Chosun University, Gwangju, South Korea; Department of Internal Medicine, School of Medicine, Chosun University, Gwangju, South Korea; Department of Neurosurgery, School of Medicine, Chosun University, Gwangju, South Korea; Department of Preventive Medicine, School of Medicine, Chosun University, Gwangju, South Korea. (2)School of Medicine, Chosun University, Gwangju, South Korea; Premedical Science, College of Medicine, Chosun University, Gwangju, South Korea; Department of Internal Medicine, School of Medicine, Chosun University, Gwangju, South Korea; Department of Neurosurgery,

School of Medicine, Chosun University, Gwangju, South Korea; Department of
Preventive Medicine, School of Medicine, Chosun University, Gwangju, South Korea
drongkim@chosun.ac.kr.

Comment in
Am J Trop Med Hyg. 2016 Jan;94(1):3-4.

In South Korea, scrub typhus is one of the most common rickettsial diseases. The number of scrub typhus patients has increased in South Korea, a total of 69,210 cases were reported from 2001 to 2013. The seasonality and relation of scrub typhus cases to latitude were analyzed in this article using data obtained from the National Notifiable Diseases Surveillance System website of the Korea Centers for Disease Control and Prevention. The incidence of scrub typhus tended to increase in the later months of the year, especially in October-December. In general, lower latitudes were associated with a later peak incidence. Our results suggest for the first time that the monthly observed incidence tended to increase in the later months of the year as the latitude decreased, and on a yearly basis in Korea.

© The American Society of Tropical Medicine and Hygiene.

DOI: 10.4269/ajtmh.15-0474
PMCID: PMC4710434 [Available on 2017-01-06]
PMID: 26503283 [PubMed - indexed for MEDLINE]

34. Am J Trop Med Hyg. 2015 Feb;92(2):256-61. doi: 10.4269/ajtmh.14-0377. Epub 2014 Dec 1.

Etiologies of acute undifferentiated fever and clinical prediction of scrub typhus in a non-tropical endemic area.

Jung HC(1), Chon SB(1), Oh WS(2), Lee DH(1), Lee HJ(1).

Author information:
(1)Department of Internal Medicine, Kangwon National University School of Medicine, Chuncheon, Republic of Korea; Department of Emergency Medicine, Seoul National University College of Medicine, Seoul, Republic of Korea. (2)Department of Internal Medicine, Kangwon National University School of Medicine, Chuncheon, Republic of Korea; Department of Emergency Medicine, Seoul National University College of Medicine, Seoul, Republic of Korea onesbi@gmail.com.

Scrub typhus usually presents as acute undifferentiated fever. This cross-sectional study included adult patients presenting with acute undifferentiated fever defined as any febrile illness for ≤ 14 days without evidence of localized infection. Scrub typhus cases were defined by an antibody titer of a ≥ fourfold increase in paired sera, a ≥ 1:160 in a single serum using indirect immunofluorescence assay, or a positive result of the immunochromatographic test. Multiple regression analysis identified predictors associated with scrub typhus to develop a prediction rule. Of 250 cases with known etiology of acute undifferentiated fever, influenza (28.0%), hepatitis A (25.2%), and scrub typhus (16.4%) were major causes. A prediction rule for identifying suspected cases of scrub typhus consisted of age ≥ 65 years (two points), recent fieldwork/outdoor activities (one point), onset of illness during

an outbreak period (two points), myalgia (one point), and eschar (two points). The c statistic was 0.977 (95% confidence interval = 0.960-0.994). At a cutoff value ≥ 4, the sensitivity and specificity were 92.7% (79.0-98.1%) and 90.9% (86.0-94.3%), respectively. Scrub typhus, the third leading cause of acute undifferentiated fever in our region, can be identified early using the prediction rule.

© The American Society of Tropical Medicine and Hygiene.

DOI: 10.4269/ajtmh.14-0377
PMCID: PMC4347325
PMID: 25448236 [PubMed - indexed for MEDLINE]

35. J Trop Pediatr. 2016 Apr 27. pii: fmw027. [Epub ahead of print]

Distribution of Eschar in Pediatric Scrub Typhus.

Rose W(1), Rajan RJ(2), Punnen A(2), Ghosh U(2).

Author information:
(1)Department of Pediatrics, Christian Medical College, Vellore 632004, India winsleyrose@cmcvellore.ac.in. (2)Department of Pediatrics, Christian Medical College, Vellore 632004, India.

BACKGROUND: Identifying an eschar in scrub typhus is useful for initiation of prompt and appropriate antibiotic therapy.
METHODS: The distribution of eschars in all children <15 years of age admitted with confirmed scrub typhus over a 5 year period is described.
RESULTS: Of 431 children admitted with scrub typhus, eschars were present in 176 (40.8%) children with the following distribution: head, face and neck, 33 (19.1%); axillae, 37 (21%); chest and abdomen, 21 (11.9%); genitalia, inguinal region and buttocks, 58 (33%); back, 8 (4.5%); upper extremities, 13 (7.4%); and lower extremities, 5 (2.8%). The commonest sites of eschars were scrotum (27 of 106; 25.5%) and axillae (15 of 106; 14.2%) in males and axillae (22 of 70; 31.4%) and groin (16 of 70; 22.9%) in females. Eschars were seen within skin folds in 100 of 176 (56.8%) children.
CONCLUSION: Children should be carefully examined for the presence of eschar especially in the skin folds of the genitalia, axillae and groin to make an early diagnosis of scrub typhus.

© The Author [2016]. Published by Oxford University Press. All rights reserved. For Permissions, please email: journals.permissions@oup.com.

DOI: 10.1093/tropej/fmw027
PMID: 27122479 [PubMed - as supplied by publisher]

36. PLoS Negl Trop Dis. 2015 Aug 28;9(8):e0003990. doi: 10.1371/journal.pntd.0003990. eCollection 2015.

Increased Nucleosomes and Neutrophil Activation Link to Disease Progression in Patients with Scrub Typhus but Not Murine Typhus in Laos.

Paris DH(1), Stephan F(2), Bulder I(2), Wouters D(2), van der Poll T(3), Newton PN(4), Day NP(5), Zeerleder S(6).

Author information:
(1)Mahidol-Oxford Tropical Medicine Research Unit, Faculty of Tropical Medicine, Mahidol University, Bangkok, Thailand; Centre for Tropical Medicine & Global Health, Nuffield Department of Medicine, University of Oxford, Oxford, United Kingdom; Lao-Oxford-Mahosot Hospital-Wellcome Trust Research Unit, Microbiology Laboratory, Mahosot Hospital, Vientiane, Lao PDR. (2)Sanquin Research, Department of Immunopathology, Amsterdam, The Netherlands. (3)Department of Vascular Medicine and Centre for Experimental and Molecular Medicine (CEMM), Academic Medical Centre, University of Amsterdam, Amsterdam, The Netherlands. (4)Centre for Tropical Medicine & Global Health, Nuffield Department of Medicine, University of Oxford, Oxford, United Kingdom; Lao-Oxford-Mahosot Hospital-Wellcome Trust Research Unit, Microbiology Laboratory, Mahosot Hospital, Vientiane, Lao PDR. (5)Mahidol-Oxford Tropical Medicine Research Unit, Faculty of Tropical Medicine, Mahidol University, Bangkok, Thailand; Centre for Tropical Medicine & Global Health, Nuffield Department of Medicine, University of Oxford, Oxford, United Kingdom. (6)Sanquin Research, Department of Immunopathology, Amsterdam, The Netherlands; Department of Hematology, Academic Medical Centre, Amsterdam, The Netherlands.

Cell-mediated immunity is essential in protection against rickettsial illnesses, but the role of neutrophils in these intracellular vasculotropic infections remains unclear. This study analyzed the plasma levels of nucleosomes, FSAP-activation (nucleosome-releasing factor), and neutrophil activation, as evidenced by neutrophil-elastase (ELA) complexes, in sympatric Lao patients with scrub typhus and murine typhus. In acute scrub typhus elevated nucleosome levels correlated with lower GCS scores, raised respiratory rate, jaundice and impaired liver function, whereas neutrophil activation correlated with fibrinolysis and high IL-8 plasma levels, a recently identified predictor of severe disease and mortality. Nucleosome and ELA complex levels were associated with a 4.8-fold and 4-fold increased risk of developing severe scrub typhus, beyond cut off values of 1,040 U/ml for nucleosomes and 275 U/ml for ELA complexes respectively. In murine typhus, nucleosome levels associated with pro-inflammatory cytokines and the duration of illness, while ELA complexes correlated strongly with inflammation markers, jaundice and increased respiratory rates. This study found strong correlations between circulating nucleosomes and neutrophil activation in patients with scrub typhus, but not murine typhus, providing indirect evidence that nucleosomes could originate from neutrophil extracellular trap (NET) degradation. High circulating plasma nucleosomes and ELA complexes represent independent risk factors for developing severe complications in scrub typhus. As nucleosomes and histones exposed on NETs are highly cytotoxic to endothelial cells and are strongly pro-coagulant, neutrophil-derived nucleosomes could contribute to vascular damage, the pro-coagulant state and exacerbation of disease in scrub typhus, thus indicating a detrimental role of neutrophil activation. The data suggest that increased neutrophil activation relates to disease progression and severe complications, and increased plasma levels of nucleosomes and ELA complexes represent independent risk factors for developing severe scrub typhus.

DOI: 10.1371/journal.pntd.0003990
PMCID: PMC4552835
PMID: 26317419 [PubMed - indexed for MEDLINE]

37. PLoS Negl Trop Dis. 2015 Jan 8;9(1):e3420. doi: 10.1371/journal.pntd.0003420.
eCollection 2015.

Burden of disease measured by disability-adjusted life years and a disease forecasting time series model of scrub typhus in Laiwu, China.

Yang LP(1), Liang SY(1), Wang XJ(2), Li XJ(1), Wu YL(1), Ma W(1).

Author information:
(1)Department of Epidemiology and Health Statistics, School of Public Health, Shandong University, Jinan, Shandong, People's Republic of China. (2)Shandong Center for Disease Control and Prevention, Jinan, Shandong, People's Republic of China.

BACKGROUND: Laiwu District is recognized as a hyper-endemic region for scrub typhus in Shandong Province, but the seriousness of this problem has been neglected in public health circles.
METHODOLOGY/PRINCIPAL FINDINGS: A disability-adjusted life years (DALYs) approach was adopted to measure the burden of scrub typhus in Laiwu, China during the period 2006 to 2012. A multiple seasonal autoregressive integrated moving average model (SARIMA) was used to identify the most suitable forecasting model for scrub typhus in Laiwu. Results showed that the disease burden of scrub typhus is increasing yearly in Laiwu, and which is higher in females than males. For both females and males, DALY rates were highest for the 60-69 age group. Of all the SARIMA models tested, the SARIMA(2,1,0)(0,1,0)12 model was the best fit for scrub typhus cases in Laiwu. Human infections occurred mainly in autumn with peaks in October.
CONCLUSIONS/SIGNIFICANCE: Females, especially those of 60 to 69 years of age, were at highest risk of developing scrub typhus in Laiwu, China. The SARIMA (2,1,0)(0,1,0)12 model was the best fit forecasting model for scrub typhus in Laiwu, China. These data are useful for developing public health education and intervention programs to reduce disease.

DOI: 10.1371/journal.pntd.0003420
PMCID: PMC4288724
PMID: 25569248 [PubMed - indexed for MEDLINE]

38. Heart. 2014 Dec;100(23):1844-50. doi: 10.1136/heartjnl-2014-306181. Epub 2014 Aug 21.

Scrub typhus increases the risk of developing acute coronary syndrome: a nationwide cohort study.

Chung WS(1), Lin CL(2), Hsu WH(3), Kao CH(4).

Author information:
(1)Department of Internal Medicine, Taichung Hospital, Ministry of Health and Welfare, Taichung, Taiwan Department of Healthcare Administration, Central Taiwan University of Science and Technology, Taichung, Taiwan. (2)Management Office for Health Data, China Medical University Hospital, Taichung, Taiwan College of Medicine, China Medical University, Taichung, Taiwan. (3)Department of

Respiratory and Critical Care, China Medical University Hospital, Taichung,
Taiwan Graduate Institute of Clinical Medical Science and School of Medicine,
College of Medicine, China Medical University, Taichung, Taiwan. (4)Graduate
Institute of Clinical Medical Science and School of Medicine, College of
Medicine, China Medical University, Taichung, Taiwan Department of Nuclear
Medicine and PET Center, China Medical University Hospital, Taichung, Taiwan.

BACKGROUND: Studies investigating the epidemiological relationship between scrub typhus and the subsequent development of acute coronary syndrome (ACS) are lacking. Therefore, we conducted a nationwide longitudinal cohort study in Taiwan to explore whether patients with scrub typhus are at an increased risk of developing ACS.
METHODS: This study investigated the incidence and risk factors for ACS in 5215 patients newly diagnosed with scrub typhus from the Taiwan National Health Insurance Research Database between 2000 and 2011. The comparison cohort contained 20 860 persons from the general population without scrub typhus. The follow-up period ran from the time of the initial diagnosis for scrub typhus to the date of an ACS event, censoring, or 31 December 2011. We used Cox proportional hazard regression models to analyse the risk of ACS by including the variables of sex, age and comorbidities.
RESULTS: The incidence of ACS was higher in patients with scrub typhus than in the comparison cohort (3.10 vs 1.92 per 1000 person-years). The HR of developing ACS increased by 37% in patients with scrub typhus after adjusting for age, sex and comorbidities. Men, increased age, hypertension, diabetes, hyperlipidaemia, chronic obstructive pulmonary disease and coronary artery disease were identified as independent risk factors of developing ACS after controlling for covariates. The prominent effect of scrub typhus on subsequent ACS development appeared within 1 year after infection.
CONCLUSIONS: This nationwide study determined that patients with scrub typhus exhibited a 37% increase in the risk of subsequently developing ACS compared with that of the general population.

Published by the BMJ Publishing Group Limited. For permission to use (where not already granted under a licence) please go to
http://group.bmj.com/group/rights-licensing/permissions.

DOI: 10.1136/heartjnl-2014-306181
PMID: 25147287 [PubMed - indexed for MEDLINE]

39. Trop Doct. 2015 Apr;45(2):146-7. doi: 10.1177/0049475514565426. Epub 2014 Dec 30.

Scrub typhus with visual hallucinations.

Mahajan SK(1), Kaushik M(2), Raina R(3), Sharma RC(4), Thakur P(5), Bharat Sharma J(5).

Author information:
(1)Assistant Professor, Department of Medicine, I. G. Medical College, Shimla,
Himachal Pradesh, India sanjay_mahajan64@rediffmail.com. (2)Assistant Professor,
Department of Medicine, I. G. Medical College, Shimla, Himachal Pradesh, India.
(3)Professor, Department of Medicine, I. G. Medical College, Shimla, Himachal
Pradesh, India. (4)Professor and Head, Department of Psychiatry, I. G. Medical
College, Shimla, Himachal Pradesh, India. (5)Resident, Department of Medicine, I.

G. Medical College, Shimla, Himachal Pradesh, India.

The occurrence of psychiatric symptoms in scrub typhus is not commonly reported in literature. We present a case of scrub typhus with visual hallucinations.

© The Author(s) 2014 Reprints and permissions:
sagepub.co.uk/journalsPermissions.nav.

DOI: 10.1177/0049475514565426
PMID: 25552462 [PubMed - indexed for MEDLINE]

40. PLoS One. 2015 Feb 17;10(2):e0113968. doi: 10.1371/journal.pone.0113968.
eCollection 2015.

Scrub typhus, a disease with increasing threat in Guangdong, China.

De W(1), Jing K(1), Huan Z(1), Qiong ZH(1), Monagin C(2), Min ZJ(3), Ping H(1), Wen KC(1), Yan LJ(1).

Author information:
(1)Institute of Pathogenic Microbiology, Center for Disease Control and Prevention of Guangdong, Guangzhou, People's Republic of China. (2)Metabiota, Inc., San Francisco, California, United States of America. (3)Department of Biological Sciences, Humboldt State University, Arcata, California 95521, United States of America.

There has been a rapid increase in the number of scrub typhus cases in Guangdong Province, China. For this reason, an epidemiologic study was conducted to understand the characteristics of scrub typhus epidemics in Guangdong. From 2006 to 2013, the incidence of human cases increased from 0.4321 to 3.5917 per 100,000 with a bimodal peak in human cases typically occurring between May and November. To detect the prevalence of Orientia tsutsugamushi among suspected human cases and rodents, we performed ELISA tests of IgM/IgG and nested PCR tests on 59 whole blood samples from the suspected cases and 112 spleen samples from the rodents. Suspected cases tested positive for anti-O. tsutsugamushi IgM and IgG 66.1% (39/59) and 50.8% (30/59) of the time, respectively. Additionally, 20.3% (12/59) of blood samples and 13.4% (15/112) of spleen samples were positive for PCR. Phylogenetic analysis revealed that there were four definable clusters among the 27 nucleotide sequences of the 56-kDa antigen genes: 44.4% Karp (12/27), 25.9% Kato (7/27), 22.2% Gilliam (6/27) and 7.4% TA763 (2/27). We concluded many suspected cases may result in diagnostic errors; therefore, it is necessary to perform laboratory tests on suspected cases in hospitals. The high infection rate of O. tsutsugamushi among the limited rodents tested suggested that further rodent sampling throughout the province is necessary to further define high-risk areas. Furthermore, the multiple co-circulating genotypes of O. tsutsugamushi play a key role in the pervasiveness of scrub typhus in the Guangdong area.

DOI: 10.1371/journal.pone.0113968
PMCID: PMC4331496
PMID: 25689778 [PubMed - indexed for MEDLINE]

41. Indian J Med Microbiol. 2014 Jul-Sep;32(3):247-50. doi: 10.4103/0255-0857.136552.

Recent outbreak of scrub typhus in North Western part of India.

Sinha P, Gupta S(1), Dawra R, Rijhawan P.

Author information:
(1)Department of Microbiology, Mahatma Gandhi Medical College, Jaipur, Rajasthan, India.

BACKGROUND: Scrub typhus usually affects previously healthy active persons and if undiagnosed or diagnosed late, may prove to be life-threatening. Diagnosis of scrub typhus should be largely based on a high index of suspicion and careful clinical, laboratory and epidemiological evaluation.
OBJECTIVE: To describe the diverse clinical and laboratory manifestations of scrub typhus diagnosed in Mahatma Gandhi Medical College and Hospital, Jaipur.
MATERIALS AND METHODS: All cases of febrile illness diagnosed as scrub typhus over a period of 3 months were analysed. Diagnosis was based on ELISA test for antibody detection against 56 kDa antigen.
RESULTS: Forty-two cases of scrub typhus were seen over a period of 3 months (October, 2012-December, 2012). Common symptoms were high grade fever of 4-30 days duration, cough, haemoptysis and breathlessness. Eschar was not seen even in a single patient. Liver enzymes were elevated in nearly all cases (95.9%).
Multiple organ dysfunction syndrome (MODS) was present in 16.66% of our patients (7 out of 42). Hypotension (6 patients, 14.2%), renal impairment (9 out of 15 patients, 60%), acute respiratory distress syndrome (4 patients, 9.52%) and meningitis (4 patients, 9.52%) were some of the important complications. There was a dramatic response to doxycycline in nearly all the patients, but initially when the disease was not diagnosed, seven patients had died.
CONCLUSION: Scrub typhus has emerged as an important cause of febrile illness in Jaipur. Empirical treatment with doxycycline is justified in endemic areas.

DOI: 10.4103/0255-0857.136552
PMID: 25008815 [PubMed - indexed for MEDLINE]

42. Parasit Vectors. 2014 Nov 18;7:513. doi: 10.1186/s13071-014-0513-7.

A city park as a potential epidemic site of scrub typhus: a case-control study of an outbreak in Guangzhou, China.

Wei Y(1), Luo L(2), Jing Q(3), Li X(4), Huang Y(5), Xiao X(6), Liu L(7), Wu X(8), Yang Z(9).

Author information:
(1)Guangzhou Center for Disease Control and Prevention, Guangzhou, Guangdong Province, China. wei_yh0928@163.com. (2)Guangzhou Center for Disease Control and Prevention, Guangzhou, Guangdong Province, China. llyeyq@163.com. (3)Guangzhou Center for Disease Control and Prevention, Guangzhou, Guangdong Province, China. jingqinlong@126.com. (4)School of Public Health, Guangdong Pharmaceutical University, Guangzhou, Guangdong Province, China. ningqzone@163.com. (5)Guangzhou Center for Disease Control and Prevention, Guangzhou, Guangdong Province, China. hyang5556@163.com. (6)Guangzhou Center for Disease Control and Prevention, Guangzhou, Guangdong Province, China. crbfzk@gzcdc.org.cn. (7)Guangzhou Center for Disease Control and Prevention, Guangzhou, Guangdong Province, China.

liulan728@163.com. (8)Guangzhou Center for Disease Control and Prevention, Guangzhou, Guangdong Province, China. tom.wu@tom.com. (9)Guangzhou Center for Disease Control and Prevention, Guangzhou, Guangdong Province, China. yangzc@gzcdc.org.cn.

BACKGROUND: Scrub typhus is an important public health problem in China, especially in Guangzhou city. Typical outbreaks of scrub typhus have been previously reported in rural areas, affecting mainly farmers. We describe an atypical outbreak of the disease with case fatalities, from a park in Haizhu District, Guangzhou, that could turn out to be a potential scrub typhus epidemic site.
METHODS: From May 2012 to June 2012, a case-control study was conducted to identify source and risk factors of this outbreak. Reported cases of scrub typhus in Xiaogang Park were confirmed by Weil-Felix test or a nested polymerase chain reaction (NPCR). Controls were matched with their neighbors by gender and age. Multivariate conditional logistic regression was used to identify risk factors and protective factors.
RESULTS: A total of 29 cases were confirmed by Weil-Felix test, including 4 deaths by both Weil-Felix test and NPCR. All patients presented with fever (100%), while 28 (96.6%) cases had eschars, 10 (34.5%) headache, 10 (34.5%) chills, 6 (20.7%) lymphadenopathy, 5 (17.2%) rash, 2 (6.9%) vomiting and 1 (3.5%) presented with conjunctival congestion. The proportion of cases with activity history in Xiaogang Park was much higher than the control group (72.4% vs 24.1%, $P < 0.001$), and morning exercise in park or field was also as a risk factor for scrub typhus (adjusted OR = 3.0, 95% CI: 1.1-8.2). Four factors were significantly associated with the risk of developing scrub typhus: sitting on the lawn (adjusted OR = 8.0, 95% CI: 1.4-44.5), close contact with rats (adjusted OR = 3.3, 95% CI: 1.2-9.6), sitting near the rat holes (OR = 6.8, 95% CI: 1.2-38.1) and wearing long-sleeved clothing when outside (adjusted OR = 0.3, 95% CI: 0.1-0.7).
CONCLUSIONS: We confirmed an atypical outbreak of scrub typhus in a park in Guangzhou city, which has the potential to develop into an important epidemic site. This public health risk should not be neglected and requires more attention from authorities.

DOI: 10.1186/s13071-014-0513-7
PMCID: PMC4240828
PMID: 25403988 [PubMed - indexed for MEDLINE]

43. Trop Biomed. 2013 Dec;30(4):706-9.

Abducens nerve palsy in a patient with scrub typhus: a case report.

Bhardwaj B(1), Panda P(2), Revannasiddaiah S(3), Bhardwaj H(4).

Author information:
(1)Indira Gandhi Medical College, Department of Pulmonary Medicine and Tuberculosis Shimla, Himachal Pradesh, India: 171001. (2)Department of Cardiology, Indira Gandhi Medical College Shimla, India. (3)Department of Radiation Oncology, Health Care Global-Bangalore Institute of Oncology, Bengaluru, Karnataka, India. (4)Pulmonary Medicine & Critical Care, Internal Medicine, University of Oklahoma Health Sciences Center, Oklahoma City, USA.

Abducens nerve palsy is a known but rare complication of a few bacterial and viral infections like Mycoplasma pneumonia, cytomegalovirus, Epstein-Barr virus, Hanta virus, herpes zoster, and measles. Abducens nerve palsy due to scrub typhus is extremely rare and so far only one case has been reported in the literature. Scrub typhus is a febrile illness caused by rickettsia, Orientia tsutsugamushi, a gram negative intracellular obligate parasite which is endemic in Asia. This disease can present with wide range of clinical manifestations with involvement of any organ system, alone or in combination. Central nervous system involvement is very common and includes meningism, altered sensorium to focal neurological deficits. We present a rare manifestation of Scrub typhus in the form of sixth cranial nerve involvement which responded to the treatment with doxycycline.

PMID: 24522141 [PubMed - indexed for MEDLINE]

44. Epidemiol Infect. 2014 Oct;142(10):2217-26. doi: 10.1017/S0950268813003208. Epub 2014 Jan 2.

Effects of meteorological factors on scrub typhus in a temperate region of China.

Yang LP(1), Liu J(1), Wang XJ(2), Ma W(1), Jia CX(1), Jiang BF(1).

Author information:
(1)Department of Epidemiology and Health Statistics, School of Public Health,Shandong University,Jinan,China. (2)Shandong Center for Disease Control and Prevention, Jinan,China.

Scrub typhus is emerging and re-emerging in many areas: climate change may affect its spread. To explore the effects of meteorological factors on scrub typhus, monthly cases of scrub typhus from January 2006 to December 2012 in the Laiwu district of temperate northern China were analysed. We examined the correlations between scrub typhus and meteorological factors (and their delayed effects). We built a time-series adjusted negative binomial model to reflect the relationships between climate variables and scrub typhus cases. The key determinants of scrub typhus transmission were temperature, relative humidity and precipitation. Each 1°C increase in monthly average temperature in the previous 3 months, each 1% increase in monthly relative humidity in the previous 2 months and each 1 mm increase in monthly precipitation in the previous 3 months induced 15·4%, 12·6% and 0·7% increases in the monthly number of cases, respectively. In conclusion, scrub typhus is affected by climate change in temperate regions.

DOI: 10.1017/S0950268813003208
PMID: 24800904 [PubMed - indexed for MEDLINE]

45. BMC Res Notes. 2015 Sep 15;8:438. doi: 10.1186/s13104-015-1428-x.

Scrub typhus mimicking Parkinson's disease.

Premaratna R(1), Wijayalath SH(2), Miththinda JK(3), Bandara NK(4), de Silva HJ(5).

Author information:

(1)Colombo North Teaching Hospital, Ragama, Sri Lanka. ranjanp64@gmail.com.
(2)Colombo North Teaching Hospital, Ragama, Sri Lanka. nuwancw84@gmail.com.
(3)Colombo North Teaching Hospital, Ragama, Sri Lanka. jkndmiththinda@gmail.com.
(4)Colombo North Teaching Hospital, Ragama, Sri Lanka.
wijesinghekbandara@gmail.com. (5)Colombo North Teaching Hospital, Ragama, Sri
Lanka. hjanakadesilva@gmail.com.

BACKGROUND: Scrub typhus is a re-emerging infection in Sri Lanka. It often poses a diagnostic challenge and tends to present as a febrile illness of uncertain origin. Undiagnosed illness may progress to serious multi-systemic complications. Here we report a case of scrub typhus presenting with features of Parkinsonism.
CASE PRESENTATION: A 62-year-old previously healthy Sri Lankan native male from the Western province of Sri Lanka presented with high fever with malaise, myalgia and arthralgia for 17 days. On the 5th day of illness he developed intermittent resting tremor in his right arm and leg associated with stiffness, difficulty in carrying out normal work and difficulty in smiling. He denied similar previous episodes. There were no other associated neurological manifestations. Clinical examination revealed a high amplitude low frequency resting tremor in his right hand, a mask-like face and increased muscle tone limited to the right side with normal reflexes. The rest of the system examination was normal except for an eschar over the abdomen. His investigations revealed lymphocytic leukocytosis, high erythrocyte sedimentation rate and immunofluorescence assay-IgM and IgG against Orientia tsutsugamushi Karp antigen were positive with rising titers. With oral doxycycline and azithromycin his fever settled within 48 h and a complete recovery of Parkinson's features was observed within 2 weeks.
CONCLUSION: Doctors practicing in endemic regions should be familiar with delayed clinical manifestations of scrub typhus and should carefully look for an eschar in order to avoid delay in the diagnosis.

DOI: 10.1186/s13104-015-1428-x
PMCID: PMC4570152
PMID: 26369561 [PubMed - indexed for MEDLINE]

46. Arch Gerontol Geriatr. 2014 Mar-Apr;58(2):196-200. doi:
10.1016/j.archger.2013.10.011. Epub 2013 Nov 2.

Differences in the clinical presentation and the frequency of complications between elderly and non-elderly scrub typhus patients.

Jang MO(1), Kim JE(1), Kim UJ(1), Ahn JH(1), Kang SJ(1), Jang HC(1), Jung SI(1),
Park KH(2).

Author information:
(1)Department of Internal Medicine, Chonnam National University Hospital,
Gwang-ju, Republic of Korea. (2)Department of Internal Medicine, Chonnam National
University Hospital, Gwang-ju, Republic of Korea. Electronic address:
iammedkid@naver.com.

Age can affect the clinical features and severity of infectious disorders, such as scrub typhus. We performed this study to examine differences between elderly and non-elderly scrub typhus patients, and to identify risk factors predictive of disease outcomes. This retrospective study included patients admitted to a tertiary hospital with scrub typhus between 2001 and 2011. A total of 615

patients were enrolled in this study, 328 of which were >65 years of age. Of the elderly patients, 46.0% (151/328) experienced at least one complication compared to only 23.0% (66/287) in younger patients. A linear trend was observed between age and complication rates (p=0.002). The most common complication in elderly patients was acute kidney injury (75, 22.9%). Treatment failure was reported in 10 elderly patients (3.0%) compared to one non-elderly patient (0.3%). Mental confusion and dyspnea of clinical manifestations at admission were common in elderly patients. Frequency of fever, rash, and eschar were similar in both groups. The following four factors were significantly associated with severe scrub typhus in elderly patients: (1) white blood cell (WBC) counts>10,000/mm(3) (OR=2.569, CI=1.298-5.086), (2) MDRD GFR<60mL/min (OR=3.525, CI=1.864-6.667), (3) albumin≤3.0g/dL (OR=4.976, CI=2.664-9.294), and (4) acute physiology and chronic health evaluation II (APACHE II) score>10 points (OR=3.304, CI=1.793-60.87). Complications and mortality were more common in elderly patients, often associated with delays in diagnosis and treatment.

Copyright © 2013 Elsevier Ireland Ltd. All rights reserved.

DOI: 10.1016/j.archger.2013.10.011
PMID: 24268946 [PubMed - indexed for MEDLINE]

47. Clin Nucl Med. 2015 Oct;40(10):e484-5. doi: 10.1097/RLU.0000000000000879.

18F-FDG PET/CT Findings of Scrub Typhus.

Kim J(1), Kwon SY, Kang SR, Cho SG, Song HC.

Author information:
(1)From the Department of Nuclear Medicine Chonnam National University Medical School and Hospital, Gwangju, Republic of Korea.

Scrub typhus is an acute infectious disease caused by Orienta tsutsugamushi, which is clinically manifested by fever, generalized lymphadenopathy, diffuse myalgia, headache, maculopapular rash, and eschars at the site of chigger feedings. Diagnosis of scrub typhus requires compatible clinical features, history of exposure, and result of selorogic testing. In recent years, F-FDG PET/CT is seen as having increasing potential for use in examination and management of patients with infectious or inflammatory disorders. This is a PET/CT case demonstrating scrub typhus in a patient without evidence of recurrence of thyroid papillary cancer.

DOI: 10.1097/RLU.0000000000000879
PMID: 26098289 [PubMed - indexed for MEDLINE]

48. PLoS One. 2015 May 8;10(5):e0125999. doi: 10.1371/journal.pone.0125999. eCollection 2015.

Clinical characteristics and risk factors of an outbreak with scrub typhus in previously unrecognized areas, Jiangsu province, China 2013.

Hu J(1), Tan Z(1), Ren D(2), Zhang X(3), He Y(3), Bao C(1), Liu D(1), Yi Q(3), Qian W(4), Yin J(4), Xu Z(4), Yu C(3), Wang S(1), Wu B(1), Yang H(3), Yue M(5),

Zhang Y(6), Liu W(1), Zhu Y(1), Zhou M(1), Tang F(1).

Author information:
(1)Jiangsu Province Center for Disease Control and Prevention, Nanjing, China.
(2)Tongren Municipal Center for Disease Control and Prevention, Tongren, China.
(3)Taizhou Municipal Center for Disease Control and Prevention, Taizhou, China.
(4)Jingjiang County Center for Disease Control and Prevention, Jingjiang, China.
(5)The First Affiliated Hospital of Nanjing Medical University, Nanjing, China.
(6)Huadong Research Institute for Medicine and Biotechnics, Nanjing, China.

Scrub typhus, caused by Orientia tsutsugamushi, has emerged recently in Jingjiang City, China where the disease had not been known to exist. We analyzed epidemiological data, clinical characteristics and risk factors of scrub typhus outbreak in Jingjiang City, 2013. The 271 clinically diagnosed patients were predominantly farmers 50 to 69 years old and the peak of onset was early to mid-November. For the 187 laboratory-confirmed cases, the major clinical manifestations of the patients were fever (100%), eschar (88.2%), rash (87.7%), chills (87.7%), and headache (66.8%). A community-based case-control study was carried out to investigate the risk factors of the scrub typhus outbreak.
Bundling or moving waste straw (OR=9.0, 95%CI 4.6-17.8) and living at the edge of village (OR=0.6, 95%CI 0.4-0.9) posed the highest risks through single- and multi-variable conditional logistic regression. Phylogenetic analysis of the 56-kDa TSA gene showed that the new cluster (GB-C2) and the previously reported cluster (GB-C1) of O. tsutsugamushi were associated with this outbreak. These findings are useful for the establishment of a detailed control strategy for scrub typhus infection in previously unrecognized areas of Jiangsu Province, China.

DOI: 10.1371/journal.pone.0125999
PMCID: PMC4425678
PMID: 25954814 [PubMed - indexed for MEDLINE]

49. Zhonghua Er Ke Za Zhi. 2014 Sep;52(9):683-7.

[Clinical analysis of scrub typhus-associated hemophagocytic syndrome].

[Article in Chinese]

He S(1), Ge L(1), Jin Y(1), Huang A(1).

Author information:
(1)Department of Emergency Medicine, Yu Ying Children's Hospital, Wenzhou Medical College, Wenzhou 325027, China.

OBJECTIVE: To analyze the clinical manifestations and intervention against fulminant scrub typhus-associated hemophagocytic syndrome.
METHOD: The medical records for the onset time of hemophagocytic syndrome, the clinical course, the chest radiographic findings, laboratory data, antibiotic therapy, clinical outcome and its prognosis were retrospectively reviewed.
RESULT: (1) Four patients were diagnosed as scrub typhus based on clinical manifestations only, while 15 patients met the criteria of laboratory diagnosis.
All 19 patients with scrub typhus had hemophagocytic syndrome. Eschar lesion was identified in 12 patients, 7 patients were described as an ulcer. A seasonal

pattern (78.9% from June through September in 15 patients) was observed. Clinical misdiagnosis was common (all 19 cases). There were 9 patients with admitting diagnosis of scrub typhus, 10 patients were not diagnosed as scrub typhus after admission. In 5 cases within 3 days after admission diagnosis was corrected as scrub typhus. Until discharge from the hospital, 5 cases were not diagnosed with scrub typhus. In this study, the length of time from the illness onset (beginning of fever) to the occurrence of clinical symptoms was (9 ± 4) days. (2) All 19 patients had changed AST levels (149 ± 37) U/L, albumin levels (23 ± 4) g/L, C-reactive protein levels (103 ± 51) mg/L, and platelet count (48 ± 41) × 10^9/L; bone marrow aspiration revealed in 16 patients marked hemophagocytosis. Weil-Felix agglutination test revealed positive results in 6 of 15 cases. Diagnostic IFA results were positive for 14 patients; 19 patients had interstitial pneumonitis and 17 patients had pleural effusion. (3) Five cases with failure to diagnose the disease had ineffective antibiotics treatment (imipenem or β-lactam-based regimens). These patients did not receive appropriate treatment with antibiotics against scrub typhus. Fourteen patients with admitting diagnosis of scrub typhus were successfully treated with appropriate antibiotics, 8 cases with chloramphenicol, 3 cases with azithromycin, and in 3 patients (2 cases of azithromycin and one case of erythromycin), therapy was then switched to chloramphenicol. Four patients were treated with methylprednisolone and 10 patients with dexamethasone. (4) During their hospitalization, the clinical course in five cases with failure to diagnose the disease rapidly developed and progressed to the life-threatening MODS, four of five cases died. However, the course in 14 patients were relieved and did not progress to MODS.
CONCLUSION: The diagnosis of scrub typhus was frequently delayed, the early course of scrub typhus could be associated with hemophagocytic syndrome. Serious complications of MODS generally occur without antibiotic treatment. Scrub typhus-associated hemophagocytic syndrome should be taken into consideration among patients with acute systemic febrile illness, significant increases in levels of CRP, hypoalbuminemia, thrombocytopenia, splenomegaly, pneumonitis with pleural effusion, especially those with suspected exposure history. It was not easily recognized without careful observation and was present for a few days in each patient.

PMID: 25476431 [PubMed - indexed for MEDLINE]

50. PLoS Negl Trop Dis. 2014 Jan 30;8(1):e2605. doi: 10.1371/journal.pntd.0002605. eCollection 2014.

Scrub typhus is an under-recognized cause of acute febrile illness with acute kidney injury in India.

Kumar V(1), Kumar V(1), Yadav AK(1), Iyengar S(1), Bhalla A(2), Sharma N(2), Aggarwal R(3), Jain S(2), Jha V(4).

Author information:
(1)Departments of Nephrology, Postgraduate Institute of Medical Education and Research, Chandigarh, India. (2)Internal Medicine, Postgraduate Institute of Medical Education and Research, Chandigarh, India. (3)Pulmonary and Critical Care Medicine, Postgraduate Institute of Medical Education and Research, Chandigarh, India. (4)Departments of Nephrology, Postgraduate Institute of Medical Education and Research, Chandigarh, India ; George Institute for Global Health, New Delhi,

India.

BACKGROUND: Infection-related acute kidney injury (AKI) is an important preventable cause of morbidity and mortality in the tropical region. The prevalence and outcome of kidney involvement, especially AKI, in scrub typhus is not known. We investigated all patients with undiagnosed fever and multisystem involvement for scrub typhus and present the pattern of renal involvement seen.
METHODS: From September 2011 to November 2012, blood samples of all the patients with unexplained acute febrile illness and/or varying organ involvement were evaluated for evidence of scrub typhus. A confirmed case of scrub typhus was defined as one with detectable Orientia tsutsugamushi deoxyribonucleic acid (DNA) in patient's blood sample by nested polymerase chain reaction (PCR) targeting the gene encoding 56-kDa antigen and without any alternative etiological diagnosis. Renal involvement was defined by demonstration of abnormal urinalysis and/or reduced glomerular filtration rate. AKI was defined as per Kidney Disease: Improving Global Outcomes (KDIGO) definition.
RESULTS: Out of 201 patients tested during this period, 49 were positive by nested PCR for scrub typhus. Mean age of study population was 34.1±14.4 (range 11-65) years. Majority were males and a seasonal trend was evident with most cases following the rainy season. Overall, renal abnormalities were seen in 82% patients, 53% of patients had AKI (stage 1, 2 and 3 in 10%, 8% and 35%, respectively). The urinalysis was abnormal in 61%, with dipstick positive albuminuria (55%) and microscopic hematuria (16%) being most common. Acute respiratory distress syndrome (ARDS) and shock were seen in 57% and 16% of patients, respectively. Hyperbilirubinemia was associated with AKI ($p = 0.013$). A total of 8 patients (including three with dialysis dependent AKI) expired whereas rest all made uneventful recovery. Jaundice, oliguria, ARDS and AKI were associated with mortality. However, after multivariate analysis, only oliguric AKI remained a significant predictor of mortality ($p = 0.002$).
CONCLUSIONS: Scrub typhus was diagnosed in 24% of patients presenting with unexplained febrile illness according to a strict case definition not previously used in this region. Renal abnormalities were seen in almost 82% of all patients with evidence of AKI in 53%. Our finding is contrary to current perception that scrub typhus rarely causes renal dysfunction. We suggest that all patients with unexplained febrile illness be investigated for scrub typhus and AKI looked for in scrub typhus patients.

DOI: 10.1371/journal.pntd.0002605
PMCID: PMC3907360
PMID: 24498445 [PubMed - indexed for MEDLINE]

51. J Vector Borne Dis. 2015 Jun;52(2):171-4.

Molecular detection of scrub typhus in Tirupati, Andhra Pradesh, India.

Usha K, Kumar E, Kalawat U, Kumar BS, Chaudhury A, Gopal DV(1).

Author information:
(1)Department of Virology, Sri Venkateswara University, Tirupati, India.

PMID: 26119551 [PubMed - indexed for MEDLINE]

52. PLoS Negl Trop Dis. 2014 Nov 20;8(11):e3327. doi: 10.1371/journal.pntd.0003327.
eCollection 2014.

Pregnancy outcome in relation to treatment of murine typhus and scrub typhus
infection: a fever cohort and a case series analysis.

McGready R(1), Prakash JA(2), Benjamin SJ(2), Watthanaworawit W(3), Anantatat T(4), Tanganuchitcharnchai A(4), Ling CL(1), Tan SO(3), Ashley EA(1), Pimanpanarak M(3), Blacksell SD(5), Day NP(5), Singhasivanon P(4), White NJ(5), Nosten F(1), Paris DH(5).

Author information:
(1)Shoklo Malaria Research Unit (SMRU), Mahidol-Oxford Tropical Medicine Research Unit, Mae Sot, Tak, Thailand; Centre for Tropical Medicine, Nuffield Department of Medicine, University of Oxford, Oxford, United Kingdom. (2)Immunology Laboratories, Department of Clinical Microbiology, Christian Medical College, Vellore, Tamil Nadu, India. (3)Shoklo Malaria Research Unit (SMRU), Mahidol-Oxford Tropical Medicine Research Unit, Mae Sot, Tak, Thailand. (4)Mahidol-Oxford Tropical Medicine Research Unit (MORU), Faculty of Tropical Medicine, Mahidol University, Bangkok, Thailand. (5)Centre for Tropical Medicine, Nuffield Department of Medicine, University of Oxford, Oxford, United Kingdom; Mahidol-Oxford Tropical Medicine Research Unit (MORU), Faculty of Tropical Medicine, Mahidol University, Bangkok, Thailand.

BACKGROUND: There is a paucity of published reports on pregnancy outcome following scrub and murine typhus despite these infections being leading causes of undifferentiated fever in Asia. This study aimed to relate pregnancy outcome with treatment of typhus.
METHODOLOGY/PRINCIPAL FINDINGS: Data were analyzed from: i) pregnant women with a diagnosis of scrub and/or murine typhus from a fever cohort studies; ii) case series of published studies in PubMed using the search terms "scrub typhus" (ST), "murine typhus" (MT), "Orientia tsutsugamushi", "Rickettsia tsutsugamushi", "Rickettsia typhi", "rickettsiae", "typhus", or "rickettsiosis"; and "pregnancy", until February 2014 and iii) an unpublished case series. Fever clearance time (FCT) and pregnancy outcome (miscarriage and delivery) were compared to treatment. Poor neonatal outcome was a composite measure for pregnancies sustained to 28 weeks or more of gestation ending in stillbirth, preterm birth, or delivery of a growth restricted or low birth weight newborn.
RESULTS: There were 26 women in the fever cohort. MT and ST were clinically indistinguishable apart from two ST patients with eschars. FCTs (median [range] hours) were 25 [16-42] for azithromycin (n=5), 34 [20-53] for antimalarials (n=5) and 92 [6-260] for other antibiotics/supportive therapy (n=16). There were 36.4% (8/22) with a poor neonatal outcome. In 18 years, 97 pregnancies were collated, 82 with known outcomes, including two maternal deaths. Proportions of miscarriage 17.3% (14/81) and poor neonatal outcomes 41.8% (28/67) were high, increasing with longer FCTs (p=0.050, linear trend). Use of azithromycin was not significantly associated with improved neonatal outcomes (p=0.610).
CONCLUSION: The published ST and MT world literature amounts to less than 100 pregnancies due to under recognition and under diagnosis. Evidence supporting the most commonly used treatment, azithromycin, is weak. Collaborative, prospective clinical trials in pregnant women are urgently required to reduce the burden of adverse maternal and newborn outcomes and to determine the safety and efficacy of antimicrobial treatment.

DOI: 10.1371/journal.pntd.0003327
PMCID: PMC4238995
PMID: 25412503 [PubMed - indexed for MEDLINE]

53. Indian Pediatr. 2014 Aug;51(8):651-3.

Clinical profile of scrub typhus in children and its association with hemophagocytic lymphohistiocytosis.

Sankhyan N(1), Saptharishi LG, Sasidaran K, Kanga A, Singhi SC.

Author information:
(1)Department of Pediatrics, PGIMER, Chandigarh; and *Department of Microbiology, Indira Gandhi Medical College, Shimla, India. Correspondence to: Prof. Sunit C Singhi, Professor and Head, Department of Pediatrics, Advanced Pediatric Centre, Post Graduate Institute of Medical Education and Research, Chandigarh 160 012, India. sunit.singhi@gmail.com.

OBJECTIVE: To study the clinical profile of children with scrub typhus and its association with hemophagocytic lymphohistiocytosis.
METHODS: Children presenting with unexplained fever and multi-systemic involvement between May to December 2011 were tested for scrub typhus using IgM ELISA kits. Occurrence of Hemophagocytic lymphohistiocytosis in IgM positive cases of scrub typhus was studied.
RESULTS: Of the 35 children with unexplained fever and multi-systemic involvement, 15 children (9 boys) tested positive for scrub typhus. Thrombocytopenia, hypoalbuminemia and raised hepatic transaminases were observed in all children. Out of seven children evaluated for hemophagocytic lymphohistiocytosis. 3 met the criteria for hemophagocytosis. Two children (one with hemophagocytic lymphohistiocytosis) died.
CONCLUSIONS: Scrub typhus is a common cause of unexplained fever in children in northern India. Hemophagocytic lymphohistiocytosis can occasionally complicate scrub typhus in children.

PMID: 25129000 [PubMed - indexed for MEDLINE]

54. PLoS One. 2014 Jul 9;9(7):e101976. doi: 10.1371/journal.pone.0101976. eCollection 2014.

Rapid increase of scrub typhus: an epidemiology and spatial-temporal cluster analysis in Guangzhou City, Southern China, 2006-2012.

Wei Y(1), Huang Y(1), Luo L(1), Xiao X(1), Liu L(1), Yang Z(1).

Author information:
(1)Guangzhou Center for Disease Control and Prevention, Guangdong Province, Guangzhou, China.

BACKGROUND: Scrub typhus has been increasingly reported in Southern China, and public health authorities are concerned about its increased incidence.

Additionally, little evidence is available on the epidemiology of scrub typhus in Southern China. This study aims to analyze the epidemiological and geographic features of ST in Guangzhou City, Southern China, to guide the future prevention efforts.
METHODS: Scrub typhus surveillance data in Guangzhou City during 2006-2012 were obtained from the Chinese National Communicable Disease Surveillance Network. We first conducted a descriptive analysis to analyze the epidemiological features of scrub typhus. Then we used space-time scan statistic based on a discrete Poisson model to detect and evaluate high-risk spatial-temporal clusters of scrub typhus.
RESULTS: There were 4,001 cases of scrub typhus in Guangzhou City during the study period. The incidence of scrub typhus increased from 3.29 per 100,000 in 2006 to 9.85 per 100,000 in 2012. A summer peak was observed in June and July with a second peak in September and October except year 2009 and 2011. The majority of the cases (71.4%) were among persons aged ≥40 years, and female incidence was higher than male incidence in persons ≥50 years. In the space-time analysis, high-risk clusters were concentrated in rural areas in Guangzhou City. Over the past 7 years, Haizhu District, an urban area, was found to be a high-risk cluster for the first time in 2012.
CONCLUSION: The resurgence of scrub typhus epidemics in Guangzhou population in 2012 necessitates more effective measures for minimizing future epidemics. Consideration of high-risk population and historical spatial-temporal clusters may help prevent scrub typhus. The risk of scrub typhus in urban areas should not be neglected and needs more attention from public health authorities.

DOI: 10.1371/journal.pone.0101976
PMCID: PMC4090214
PMID: 25006820 [PubMed - indexed for MEDLINE]

55. Indian J Public Health. 2014 Oct-Dec;58(4):281-3. doi: 10.4103/0019-557X.146299.

Scrub typhus-an emerging entity: a study from a tertiary care hospital in North India.

Oberoi A(1), Varghese SR.

Author information:
(1)Department of Microbiology, Christian Medical College and Hospital, Ludhiana, Punjab, India.

Scrub typhus is a tropical febrile zoonotic disease caused by Orientia tsutsugamushi of the rickettsial family. These are obligate; intracellular Gram-negative coccobacilli transmitted by the bite of infected mites. It is usually under-diagnosed in India due to its varied and nonspecific clinical presentation, limited awareness, and low index of suspicion among clinicians and lack of diagnostic facilities. This study was planned to monitor the level of scrub typhus-specific antibodies among febrile patients in a tertiary care hospital over a period of 1 year for which a rapid qualitative immunochromatographic assay (Standard Diagnostics, Korea) was introduced for the detection of IgM, IgG and IgA antibodies to O. tsutsugamushi from the serum of suspected febrile patients. A total of 98 out of 772 fever patients (12.69%) tested positive for the presence of antibodies against O. tsutsugamushi. Persistent high-grade fever was the defining characteristic in all the cases with the presence of an eschar in only 10.2% (10/98) of cases. Three patients died

during the study period while the rest responded to treatment with doxycycline.

DOI: 10.4103/0019-557X.146299
PMID: 25491523 [PubMed - indexed for MEDLINE]

56. PLoS Negl Trop Dis. 2014 Feb 6;8(2):e2648. doi: 10.1371/journal.pntd.0002648.
eCollection 2014.

Cytokine network in scrub typhus: high levels of interleukin-8 are associated with disease severity and mortality.

Astrup E(1), Janardhanan J(2), Otterdal K(3), Ueland T(3), Prakash JA(4), Lekva T(5), Strand ØA(6), Abraham OC(2), Thomas K(7), Damås JK(8), Mathews P(7), Mathai D(7), Aukrust P(9), Varghese GM(2).

Author information:
(1)Institute of Clinical Medicine, Akershus University Hospital, Lørenskog, Norway ; Research Institute for Internal Medicine, Oslo University Hospital Rikshospitalet, Oslo, Norway. (2)Department of Medicine and Infectious Diseases, Christian Medical College, Vellore, Tamil Nadu, India. (3)Research Institute for Internal Medicine, Oslo University Hospital Rikshospitalet, Oslo, Norway ; Faculty of Medicine, University of Oslo, Oslo, Norway. (4)Department of Microbiology, Christian Medical College, Vellore, Tamil Nadu, India. (5)Research Institute for Internal Medicine, Oslo University Hospital Rikshospitalet, Oslo, Norway ; Faculty of Medicine, University of Oslo, Oslo, Norway ; Section of Specialized Endocrinology, Department of Endocrinology, Oslo University Hospital Rikshospitalet, Oslo, Norway. (6)Department of Infectious Diseases, Akershus University Hospital, Lørenskog, Norway. (7)Department of Medicine, Christian Medical College, Vellore, Tamil Nadu, India. (8)Institute of Cancer Research and Molecular Medicine, Norwegian University of Science and Technology, Trondheim, Norway ; Department of Infectious Diseases, St. Olav's Hospital, Trondheim, Norway. (9)Research Institute for Internal Medicine, Oslo University Hospital Rikshospitalet, Oslo, Norway ; Faculty of Medicine, University of Oslo, Oslo, Norway ; Section of Clinical Immunology and Infectious Diseases, Oslo University Hospital Rikshospitalet, Oslo, Norway.

BACKGROUND: Scrub typhus, caused by Orientia tsutsugamushi, is endemic in the Asia-Pacific region. Mortality is high if untreated, and even with treatment as high as 10-20%, further knowledge of the immune response during scrub typhus is needed. The current study was aimed at comparing plasma levels of a variety of inflammatory mediators in scrub typhus patients and controls in South India in order to map the broader cytokine profile and their relation to disease severity and clinical outcome.
METHODOLOGY/PRINCIPAL FINDINGS: We examined plasma levels of several cytokines in scrub typhus patients (n = 129) compared to healthy controls (n = 31) and infectious disease controls (n = 31), both in the acute phase and after recovery, by multiplex technology and enzyme immunoassays. Scrub typhus patients were characterized by marked changes in the cytokine network during the acute phase, differing not only from healthy controls but also from infectious disease controls. While most of the inflammatory markers were raised in scrub typhus, platelet-derived mediators such as RANTES were markedly decreased, probably reflecting enhanced platelet activation. Some of the inflammatory markers, including various chemokines (e.g., interleukin-8, monocyte chemoattractant

peptide-1 and macrophage inflammatory protein-1β) and downstream markers of inflammation (e.g., C-reactive protein and pentraxin-3), were also associated with disease severity and mortality during follow-up, with a particular strong association with interleukin-8.
CONCLUSIONS/SIGNIFICANCE: Our findings suggest that scrub typhus is characterized by a certain cytokine profile that includes dysregulated levels of a wide range of mediators, and that this enhanced inflammation could contribute to disease severity and clinical outcome.

DOI: 10.1371/journal.pntd.0002648
PMCID: PMC3916254
PMID: 24516677 [PubMed - indexed for MEDLINE]

57. Cell Biochem Biophys. 2013;67(3):1461-6. doi: 10.1007/s12013-013-9646-0.

Consistency of the key genotypes of Orientia tsutsugamushi in scrub typhus patients, rodents, and chiggers from a new endemic focus of northern China.

Liu YX(1), Jia N, Xing YB, Suo JJ, Du MM, Jia N, Gao Y, Xie LJ, Liu BW, Ren SW.

Author information:
(1)Department of Nosocomial Infection Management and Disease Control, Institute of Hospital Management, Chinese People's Liberation Army General Hospital, 28 Fuxing Road, Haidian District, Beijing, 100853, People's Republic of China, Yunxiliu6511@yahoo.com.cn.

Scrub typhus is one of the most common infectious diseases of rural south and southeastern Asia and the western Pacific. It emerged in Shandong Province in northern China from autumn to winter of 1986. Since then, the "autumn-winter type scrub typhus" has been found in many areas of northern China. However, the principle genotypes of Orientia tsutsugamushi still remain unknown. This study was undertaken to identify the genotypes of O. tsutsugamushi obtained from scrub typhus patients, chigger mites and rodents from the focal point of the problem in Shandong Province. Forty-four isolates from patients, rodents, and chiggers, 47 blood clots from patients during the acute phase, 10 eschars from patients during the convalescence phase and 16 pools of larval chiggers were tested for the scrub typhus antigen 56-kD protein (Sta56) gene by nested PCR methodology and additional sequence analysis including DNA sequence alignment and phylogenetic analysis. Based on nested PCR, ninety-five initial PCR-positive samples produced amplicons using Kawasaki strain-specific primers, while the other two (the FXS4 and LHGM2 strains) produced amplicons using Karp strain-specific primers. The partial Sta56 gene sequence analysis indicated that the sequence homologies of 3 selected isolates (the B16, FXS2, and XDM2 strains) and 7 eschars out of the 95 samples, which were nested PCR-positive using Kawasaki strain-specific primers, were 94-98% to that of Kawasaki strain. The sequence homology of the FXS4 and LHGM2 strains to that of the Karp strain was respectively 83 and 96%. These findings implied that the key genotypes of O. tsutsugamushi in patients, rodents, and chiggers in Shandong Province were identical and similar to Kawasaki strains.

DOI: 10.1007/s12013-013-9646-0
PMID: 23760611 [PubMed - indexed for MEDLINE]

58. J Assoc Physicians India. 2014 Dec;62(12):24-9.

Analysis of Two Outbreaks of Scrub Typhus in Rajasthan: A Clinico-epidemiological Study.

Sharma R, Krishna VP, Manjunath, Singh H, Shrivastava S, Singh V, Dariya SS, Soni M, Sharma S.

AIMS AND OBJECTIVES: To describe the diversity of clinical manifestations, laboratory findings and outcome of scrub typhus in hospitalised patients of SMS Hospital, Jaipur during 2012 and 2013.
MATERIAL AND METHODS: All the cases of febrile illness with thrombocytopenia diagnosed as scrub typhus were analysed. Diagnosis was made by ELISA based IgM serology.
OBSERVATIONS AND RESULTS: A total of 125 patients were studied. All of them presented with fever; the other major symptoms were headache, cough, dyspnoea and myalgias. On examination, patients had hepatosplenomegaly, lymphadenopathy and eschar. On investigation elevated SGOT, SGPT with normal or elevated bilirubin levels were the most common findings. Other laboratory findings were thrombocytopenia and deranged renal function tests. Most common X-ray finding observed in these patients was bilateral lung infiltrates. Other complications were MODS, ARDS, hypotension and meningoencephalitis. Majority responded to doxycycline.
CONCLUSION: Scrub typhus though prevalent is under-reported in our country. It should be considered as an important differential diagnosis in a febrile patient with thrombocytopenia, deranged liver or renal functions, and B/L chest opacities. Early diagnosis and appropriate treatment is rewarding and prevents morbidity and mortality.

PMID: 26259419 [PubMed - indexed for MEDLINE]

59. Jpn J Infect Dis. 2014;67(6):458-63.

Epidemiology of scrub typhus and the eschars patterns in South Korea from 2008 to 2012.

Park JH(1), Kim SJ, Youn SK, Park K, Gwack J.

Author information:
(1)Division of Epidemic Intelligence Service, Korea Centers for Disease Control and Prevention.

In South Korea, scrub typhus is the most common acute febrile illness in autumn. We analyzed scrub typhus cases reported from 2008 to 2012 to describe the epidemiology of scrub typhus as well as eschar patterns. A total of 30,478 cases were reported from 2008 to 2012; the incidence rates were higher in the southern and western regions of South Korea. The common clinical symptoms of confirmed scrub typhus cases from 2010 to 2012 were fever/chills (95.2%), eschars (78.9%), and myalgia (61.7%). The primary sites of eschars were the lower extremities (19.0%), abdomen/waist (13.4%), and axilla (11.5%) in men and the shoulder/frontal chest (15.1%), lower extremities (14.5%), and abdomen/waist (13.6%) in women. Regardless of gender, eschars tended to be more on the lower

extremities among the leisure activities group. Among the occupational farm work group, who usually lived in rural areas, eschars appeared most frequently on the abdomen/waist in men and on the shoulder/frontal chest in women. Eschar patterns were influenced by gender and activities. These results could facilitate the prevention of scrub typhus and clarify the current status of scrub typhus in South Korea.

PMID: 25410561 [PubMed - indexed for MEDLINE]

60. Zhonghua Liu Xing Bing Xue Za Zhi. 2016 May;37(5):682-5. doi:
10.3760/cma.j.issn.0254-6450.2016.05.019.

[Spatial analysis of autumn-winter type scrub typhus in Shandong province, 2006-2014].

[Article in Chinese]

Yang H(1), Bi ZW(2), Kou ZQ(2), Zheng L(1), Zhao ZT(1).

Author information:
(1)Department of Epidemiology, School of Public Health, Shandong University, Jinan 250012, China. (2)Institute for Bacterial Infectious Disease Control and Prevention, Shandong Provincial Center for Disease Control and Prevention, Jinan 250014, China.

OBJECTIVE: To discuss the spatial-temporal distribution and epidemic trends of autumn-winter type scrub typhus in Shandong province, and provide scientific evidence for further study for the prevention and control of the disease.
METHODS: The scrub typhus surveillance data during 2006-2014 were collected from Shandong Disease Reporting Information System. The data was analyzed by using software ArcGIS 9.3(ESRI Inc., Redlands, CA, USA), GeoDa 0.9.5-i and SatScan 9.1.1. The Moran' s I, log-likelihood ratio(LLR), relative risk(RR)were calculated and the incidence choropleth maps, local indicators of spatial autocorrelation cluster maps and space scaning cluster maps were drawn.
RESULTS: A total of 4 453 scrub typhus cases were reported during 2006-2014, and the annual incidence increased with year. Among the 17 prefectures(municipality)in Shandong, 13 were affected by scrub typhus. The global Moran's I index was 0.501 5(P<0.01). The differences in local Moran' s I index among 16 prefectures were significant(P<0.01). The " high-high" clustering areas were mainly Wulian county, Lanshan district and Juxian county of Rizhao, Xintai county of Tai' an, Gangcheng and Laicheng districts of Laiwu, Yiyuan county of Zibo and Mengyin county of Linyi. Spatial scan analysis showed that an eastward moving trend of high-risk clusters and two new high-risk clusters were found in Zaozhuang in 2014. The centers of the most likely clusters were in the south central mountainous areas during 2006-2010 and in 2012, eastern hilly areas in 2011, 2013 and 2014, and the size of the clusters expanded in 2008, 2011, 2013 and 2014. One spatial-temporal cluster was detected from October 1, 2014 to November 30, 2014, the center of the cluster was in Rizhao and the radius was 222.34 kilometers.
CONCLUSION: A positive spatial correlation and spatial agglomerations were found in the distribution of autumn-winter type scrub typhus in Shandong. Since 2006, the epidemic area of the disease has expanded and the number of high-risk areas

has increased. Moreover, the eastward moving and periodically expanding trends of high-risk clusters were detected.

PMID: 27188362 [PubMed - in process]

61. J Neuroophthalmol. 2015 Sep;35(3):284-6. doi: 10.1097/WNO.0000000000000259.

Ophthalmoplegia Due to Scrub Typhus.

Kim J(1), Kim JS, Shin DI, Lee SH, Lee SS, Choi SY.

Author information:
(1)Department of Neurology (JK, JSK, D-IS, S-HL, S-SL), Chungbuk National University College Medicine, Chungbuk National University Hospital, Cheoungju, Korea; and Department of Neurology (S-YC), Eulji University Hospital, Daejeon, Korea.

Scrub typhus is an acute febrile infectious disease caused by Orientia tsutsugamushi. The illness is usually characterized by fever, rash, and lymphadenopathy, but severe cases progress to pulmonary and neurological involvement. We report a 69-year-old man who developed ptosis and ophthalmoplegia with a focal nodular lesion in the anterior cavernous sinus detected with magnetic resonance imaging. Found to have scrub typhus, the ptosis and ophthalmoplegia resolved after treatment with doxycycline.

DOI: 10.1097/WNO.0000000000000259
PMID: 25993123 [PubMed - indexed for MEDLINE]

62. J Clin Diagn Res. 2015 May;9(5):OD10-1. doi: 10.7860/JCDR/2015/13692.5924. Epub 2015 May 1.

Scrub Typhus with Acute Respiratory Distress Syndrome (ARDS) and its Management in Intensive Care Unit: A Case Report.

Sankuratri S(1), Kalagara P(2), Samala KB(3), Veledandi PK(3), Atiketi SB(4).

Author information:
(1)Senior Consultant, Department of Critical Care, Medical ICU, Krishna Institute of Medical Sciences Hospitals , Secunderabad, India . (2)Assistant Professor, Department of Microbiology, Apollo Institute of Medical Sciences & Research , Jubilee Hills, Hyderabad, India . (3)Junior Consultant, Department of Critical Care, Medical ICU, Krishna Institute of Medical Sciences Hospitals , Secunderabad, India . (4)Junior Consultant, Department of Critical Care, EMD, Krishna Institute of Medical Sciences Hospitals , Secunderabad, India .

Scrub typhus is zoonotic disease caused by Orientia tsutsugamushi (O tsutsugamushi). It is transmitted to humans by the bite of trombiculid mite larvae (chiggers). It is a re-emerging infectious disease in India. Clinical manifestations include fever, headache, anorexia, myalgia, eschar, adenopathy and maculopapular rash. Complications of Scrub typhus develop after first week of illness. Complications include meningoencephalitis, jaundice, myocarditis, ARDS

and renal failure. Eschar and rash may be unnoticed or absent. Thorough physical examination, identification of eschar/rash throws light in thinking about scrub typhus, treating and preventing further complications. Here, we report a case of scrub typhus with Acute Respiratory Distress Syndrome (ARDS) and its management with non invasive ventilation in the intensive care unit.

DOI: 10.7860/JCDR/2015/13692.5924
PMCID: PMC4484103
PMID: 26155511 [PubMed]

63. Indian Pediatr. 2015 Oct;52(10):891-2.

Scrub Typhus Co-infection in an Adolescent Girl with Varicella.

Chandramohan A(1), Venkatesh S, Dhandapany G, Stephen S.

Author information:
(1)Departments of Pediatrics and *Microbiology, Mahatma Gandhi Medical College and Research Institute, Pillaiyarkuppam, Puducherry, India. Correspondence: Dr V Soma, Assistant Professor, Department of Pediatrics, MGMCRI, Pillaiyarkuppam, Puducherry 607 402, India. drsomavenky@gmail.com.

BACKGROUND: Co-infections with scrub typhus have been described quite frequently in adults but less frequently in children.
CASE CHARACTERISTICS: An adolescent girl with varicella infection who had persistent fever. Associated clinical features like pain abdomen, vomiting, and features of third space losses made us suspect a co-infection. IgM and IgG antibodies by ELISA in acute and convalescent serum were suggestive of scrub typhus.
OUTCOME: She recovered following a course of oral doxycycline.
MESSAGE: In unexplained prolonged fever or atypical clinical manifestations not explainable by the primary disease process, co-infection needs to be considered.

PMID: 26499018 [PubMed - indexed for MEDLINE]

64. Int J Infect Dis. 2013 Nov;17(11):e981-7. doi: 10.1016/j.ijid.2013.05.017. Epub 2013 Jul 26.

Scrub typhus in South India: clinical and laboratory manifestations, genetic variability, and outcome.

Varghese GM(1), Janardhanan J, Trowbridge P, Peter JV, Prakash JA, Sathyendra S, Thomas K, David TS, Kavitha ML, Abraham OC, Mathai D.

Author information:
(1)Medicine Unit I and Infectious Diseases, Christian Medical College, Vellore, Tamil Nadu, India. Electronic address: georgemvarghese@hotmail.com.

OBJECTIVES: This study sought to document the clinical and laboratory manifestations, genetic variability, and outcomes of scrub typhus, an often severe infection caused by Orientia tsutsugamushi, in South India.

METHODS: Patients admitted to a large teaching hospital with IgM ELISA-confirmed scrub typhus were evaluated. Clinical examination with a thorough search for an eschar, laboratory testing, chest X-ray, and outcome were documented and analyzed. Additionally, a 410-bp region of the 56-kDa type-specific antigen gene of O. tsutsugamushi was sequenced and compared with isolates from other regions of Asia.
RESULTS: Most of the 154 patients evaluated presented with fever and non-specific symptoms. An eschar was found in 86 (55%) patients. Mild hepatic involvement was seen in most, with other organ involvement including respiratory, cardiovascular, and renal. Multi-organ dysfunction was noted in 59 (38.3%), and the fatality rate was 7.8%. Hypotension requiring vasoactive agents was found to be an independent predictor of mortality (p<0.001). The phylogeny of 26 samples showed 17 (65%) clustering with the Kato-like group and eight (31%) with the Karp-like group.
CONCLUSIONS: The presentation of scrub typhus can be variable, often non-specific, but with potentially severe multi-organ dysfunction. Prompt recognition is key to specific treatment and good outcomes. Further study of the circulating strains is essential for the development of a successful vaccine and sensitive point-of-care testing.

Copyright © 2013 International Society for Infectious Diseases. Published by Elsevier Ltd. All rights reserved.

DOI: 10.1016/j.ijid.2013.05.017
PMID: 23891643 [PubMed - indexed for MEDLINE]

65. Trop Doct. 2014 Jan;44(1):36-7. doi: 10.1177/0049475513512646. Epub 2013 Nov 12.

Central nervous system involvement in scrub typhus.

Boorugu H(1), Chrispal A, Gopinath KG, Chandy S, Prakash JJ, Abraham AM, Abraham OC, Thomas K.

Author information:
(1)Assistant Professor, Department of Internal Medicine, Christian Medical College, Vellore, India.

Scrub typhus is an emerging infectious disease in India. Among its protean clinical manifestations, central nervous system involvement is common. In this prospective observational study, altered sensorium, headache, seizures and aseptic meningitis were found to be common central nervous system manifestations. Prompt treatment with doxycycline reduces morbidity and mortality.

DOI: 10.1177/0049475513512646
PMID: 24226290 [PubMed - indexed for MEDLINE]

66. PLoS One. 2014 Dec 12;9(12):e113193. doi: 10.1371/journal.pone.0113193. eCollection 2014.

Coincidence between geographical distribution of Leptotrombidium scutellare and scrub typhus incidence in South Korea.

Roh JY(1), Song BG(1), Park WI(1), Shin EH(1), Park C(2), Park MY(1), Chang

KS(1), Lee WG(1), Lee HI(3), Shin EH(1).

Author information:
(1)Division of Medical Entomology, Center for Immunology and Pathology, Korea National Institute of Health, Cheongju, Korea. (2)Division of Arboviruses, Center for Immunology and Pathology, Korea National Institute of Health, Cheongju, Korea. (3)Division of Biosafety Evaluation and Control, Korea National Institute of Health, Cheongju, Korea.

To clarify the geographical distribution of scrub typhus vectors in Korea, a survey of larval trombiculid mites was conducted from 2005 to 2007 by collecting wild small mammals twice a year (spring and autumn) at 24 sites nationwide. A total of 67,325 mites representing 4 genera and 14 species were collected from 783 trapped rodents, corresponding to a chigger index (number of chigger mites per rodent) of 86.0. The predominant mite species were Leptotrombidium pallidum (52.6%), Leptotrombiduim scutellare (27.1%), Leptotrombidium palpale (8.2%), Leptotrombidium orientale (5.6%), and Neotrombicula tamiyai (1.7%). However, the proportions of L. scutellare in southern areas, including endemic provinces such as Jeollabuk-Do (34.3%), Jeollanam-Do (49.0%), and Gyeongsangnam-Do (88%), were relatively higher than in central Korean regions where L. pallidum was predominant. In autumn, the ratio of L. scutellare increased to 42% while the ratio of L. pallidum decreased. The geographical distribution map of the L. scutellare chigger index was identical to the incidence pattern of scrub typhus, whereas those of overall mites and L. pallidum showed no relationship with case incidence patterns. Distribution mapping analysis shows an identical geographical distribution of L. scutellare and epidemic incidence of scrub typhus in South Korea. L. pallidum could be another vector at all other parts of the Korean peninsula, including the eastern and northern regions that have a low level of scrub typhus incidence.

DOI: 10.1371/journal.pone.0113193
PMCID: PMC4264734
PMID: 25500568 [PubMed - indexed for MEDLINE]

67. Clin Exp Nephrol. 2013 Oct;17(5):725-9. doi: 10.1007/s10157-012-0753-9. Epub 2013 Jan 5.

Acute kidney injury in scrub typhus.

Attur RP(1), Kuppasamy S, Bairy M, Nagaraju SP, Pammidi NR, Kamath V, Kamath A, Rao L, Bairy I.

Author information:
(1)Department of Nephrology, Kasturba Medical College and Hospital, Manipal, Manipal University, Madhavnagar, Manipal, Udupi District, 576104, Karnataka, India, aravindraprabhu@gmail.com.

BACKGROUND: We studied the urinary abnormalities and acute kidney injury (AKI) as per RIFLE criteria in scrub typhus.
METHODS: A prospective case record-based study of scrub typhus was carried out from January 2009 to December 2010 in a tertiary hospital in South India. Patients were followed up until renal recovery or for at least 3 months after discharge. Univariate, chi-squared tests and multivariate logistic regression

analyses were performed to identify the predictors of AKI.
RESULTS: Scrub typhus was diagnosed in 259 patients. Urinary abnormalities were seen in 147 patients (56.7%) with 60 patients (23.2%) having AKI. All AKI patients had urinary abnormalities and 17 (28.3%) were oliguric. Applying RIFLE (risk, injury, failure, loss, end-stage kidney disease) criteria, R, I, F were present in 23 (38.33%), 13 (21.67%), and 24 patients (40%), respectively. Creatine phosphokinase (CPK) was raised in 33 patients (55%) and hemodialysis was required in 6 patients (10%). The case fatality rate in this study was 2 out of 259 (0.77%), both having AKI and others recovering clinically. Significant predictors of AKI were tachycardia [odds ratio (OR) 2.28], breathlessness (OR 2.281), intensive care requirement (OR 2.43), mechanical ventilation (OR 3.33), thrombocytopenia (OR 2.90) and CPK>80 U/L (OR 1.76) by univariate analysis and intensive care requirement (adjusted OR 2.89) and thrombocytopenia (AOR 2.28) by multivariable logistic regression.
CONCLUSION: Scrub typhus should be part of the differential diagnosis of acute febrile illness with AKI. AKI in scrub typhus is usually mild, non-oliguric, and renal recovery occurs in most patients. Rhabdomyolysis may be contributory to AKI. Thrombocytopenia and intensive care requirement are significant predictors of AKI in scrub typhus.

DOI: 10.1007/s10157-012-0753-9
PMID: 23292176 [PubMed - indexed for MEDLINE]

68. Am J Trop Med Hyg. 2015 Sep;93(3):435. doi: 10.4269/ajtmh.15-0140.

Incidentally Discovered Splenic Infarction Associated with Scrub Typhus.

Hwang JH(1), Lee CS(2).

Author information:
(1)Department of Internal Medicine, Chonbuk National University Medical School, Jeonju, Republic of Korea; Research Institute of Clinical Medicine, Chonbuk National University-Chonbuk National University Hospital, Jeonju, Republic of Korea. (2)Department of Internal Medicine, Chonbuk National University Medical School, Jeonju, Republic of Korea; Research Institute of Clinical Medicine, Chonbuk National University-Chonbuk National University Hospital, Jeonju, Republic of Korea lcsmd@jbnu.ac.kr.

DOI: 10.4269/ajtmh.15-0140
PMCID: PMC4559675
PMID: 26333728 [PubMed - indexed for MEDLINE]

69. J Assoc Physicians India. 2014 Jun;62(6):490-6.

Outbreak of scrub typhus in Andhra Pradesh--experience at a tertiary care hospital.

Subbalaxmi MV, Madisetty MK, Prasad AK, Teja VD, Swaroopa K, Chandra N, Upadhyaya AC, Shetty M, Rao MN, Raju YS, Lakshmis V.

AIM: To describe the clinical features, laboratory manifestations, complications in patients diagnosed with scrub typhus at a tertiary care hospital in south

India.
MATERIAL AND METHODS: All cases of acute onset fever diagnosed to have scrub typhus August 2011 to December 2012 were analysed. Cases of scrub typhus confirmed by the well felix test with a titre of 1 in 80 or more and a positive immunochromatography test were studied.
RESULTS: 176 confirmed cases of scrub typhus were studied over a period of 18 months. Majority (96%) of patients are from rural background. Farmers constituted 60% of the patients. Most common symptoms were due to the involvement of respiratory tract in the form of cough in 94 (53%) patients followed by breathlessness in 84 (47.7%). Signs of consolidation were seen in 80 (45.5%). Central nervous system involvement in the form of altered sensorium was seen in 43 (24.4%) and seizures in 11 (6.3%) patients. Eshcar was seen in 23 (13%) patients. Transaminases were elevated in 153 (86%) patients, serum alkaline phosphatase in 110 (62.5%) patients. Renal failure was seen in 49 (27.8%) cases and respiratory failure was seen in 11 (6.2%). Eight (4.5%) patients died in our study.
CONCLUSION: Scrub typhus should be suspected in patients with rural background with fever and multi system involvement. The predominant symptoms were cough and breathlessness. Central nervous system abnormalities in the form of altered sensorium was seen in 43 (24.4%). Most common laboratory abnormality noted in our patients with scrub typhus was elevated liver enzymes which were seen in 153 (86%) cases.

PMID: 25856913 [PubMed - indexed for MEDLINE]

70. Parasit Vectors. 2015 Apr 19;8:238. doi: 10.1186/s13071-015-0858-6.

Current situation of scrub typhus in South Korea from 2001-2013.

Lee HW(1), Cho PY(2), Moon SU(3), Na BK(4), Kang YJ(5), Sohn Y(6), Youn SK(7), Hong Y(8), Kim TS(9).

Author information:
(1)Department of Pathology, Immunology, and Laboratory Medicine, College of Medicine, University of Florida, Gainesville, FL, 32610, USA. rainlee67@naver.com. (2)Departments of Parasitology and Tropical Medicine, Inha University School of Medicine, Incheon, 400-712, Republic of Korea. chopy74@daum.net. (3)Department of Internal Medicine, Seoul National University Bundang Hospital, Seongnam, 463-939, Korea. mboy2@korea.com. (4)Department of Parasitology and Institute of Health Sciences, Gyeongsang National University School of Medicine, Jinju, 660-751, Korea. bkna@gnu.ac.kr. (5)Department of Biomedical Science, Jungwon University, Goesan, Chungbuk, 367-805, Republic of Korea. yjkang@jwu.ac.kr. (6)Department of Anatomy, College of Korean Medicine, Institute of Korean Medicine, Kyung Hee University, Seoul, 130-701, Korea. youngjoos@khu.ac.kr. (7)Department of Epidemiology, National Institute of Health, Osong, 363-951, Republic of Korea. yunki7777@naver.com. (8)Department of Public Health, Sahmyook University, Seoul, 139-742, Republic of Korea. jnh3549@naver.com. (9)Departments of Parasitology and Tropical Medicine, Inha University School of Medicine, Incheon, 400-712, Republic of Korea. tongsookim@inha.ac.kr.

BACKGROUND: The bacteria Orientia tsutsugamushi is the causative agent of scrub

typhus, mite-borne disease, which causes an acute febrile illness in patients. An
epidemiologic study was conducted to understand the characteristics of scrub
typhus in South Korea.
FINDINGS: Reporting of tsutsugamushi disease is mandatory in South Korea since
1994. To investigate the prevalence of tsutsugamushi disease from 2001 to 2013,
medical records from the Korea Center for Disease Control and Prevention were
reviewed. In total, 70,914 cases were reported during 2001-2013. Of these, 37.16%
(26,349) were male and 62.84% (44,565) were female. The highest number of cases
was in the 60-69-year-old age group (19,484; 27.48%), and 72.22% (51,212) were in
the 50-79-year-old age group. There were 65,100 cases (91.80%) reported during
October (24,964; 35.20%) and November (40,136; 56.60%). An almost four-fold
increase in the number of patients was observed in 2013 (10,485 cases) compared
to 2001 (2,637 cases). The highest number of patients was reported in the Jeonbuk
(9,425; 13.29%) and lowest in the Jeju (362; 0.51%).
CONCLUSIONS: A rapid increase in the incidence of patients with tsutsugamushi
disease was observed in most areas from 2001 to 2013, with the majority of cases
reported in the western and southern coast.

DOI: 10.1186/s13071-015-0858-6
PMCID: PMC4416255
PMID: 25928653 [PubMed - indexed for MEDLINE]

71. BMC Infect Dis. 2014 Mar 12;14:139. doi: 10.1186/1471-2334-14-139.

Meteorological factors and risk of scrub typhus in Guangzhou, southern China,
2006-2012.

Li T, Yang Z, Dong Z, Wang M(1).

Author information:
(1)Guangzhou Center for Disease Control and Prevention, No 1, Qide Rd, Jiahe,
Baiyun District, Guangzhou, Guangdong Province 510440, China.
mingwang@gzcdc.org.cn.

BACKGROUND: Scrub typhus is becoming the most common vector born disease in
Guangzhou, southern China. In this study, we aimed to examine the effect of
weather patterns on the incidence of Scrub typhus in the subtropical city of
Guangzhou for the period 2006-2012, and assist public health prevention and
control measures.
METHODS: Scrub typhus reported cases during the period of 2006-2012 in Guangzhou
were obtained from National Notifiable Disease Report System (NNDRS).
Simultaneous meteorological data including temperature, relative humidity,
atmospheric pressure, sunshine, and rainfall were obtained from the documentation
of the Guangzhou Meteorological Bureau. A negative binomial regression was used
to identify the relationship between meteorological variables and scrub typhus.
RESULTS: Annual incidence rates of scrub typhus from 2006 to 2012 were 3.25,
2.67, 3.81, 4.22, 4.41, 5.12, and 9.75 (per 100 000) respectively. Each 1°C rise
in temperature corresponded to an increase of 14.98% (95% CI 13.65% to 16.33%) in
the monthly number of scrub typhus cases, while a 1 hPa rise in atmospheric
pressure corresponded to a decrease in the number of cases by 8.03% (95% CI
-8.75% to -7.31%). Similarly, a 1 hour rise in sunshine corresponded to an
increase of 0.17% or 0.54%, and a 1 millimeter rise in rainfall corresponded to
an increase of 0.05% or 0.10%, in the monthly number of scrub typhus cases,

depending on the variables considered in the model.
CONCLUSION: Our study provided evidence that climatic factors were associated with occurrence of scrub typhus in Guangzhou city, China. Temperature, duration of sunshine, and rainfall were positively associated with scrub typhus incidence, while atmospheric pressure was inversely associated with scrub typhus incidence. These findings should be considered in the prediction of future patterns of scrub typhus transmission.

DOI: 10.1186/1471-2334-14-139
PMCID: PMC3995673
PMID: 24620733 [PubMed - indexed for MEDLINE]

72. Am J Trop Med Hyg. 2016 Aug 29. pii: 16-0088. [Epub ahead of print]

Scrub Typhus in Northeastern Thailand: Eschar Distribution, Abnormal Electrocardiographic Findings, and Predictors of Fatal Outcome.

Thipmontree W(1), Tantibhedhyangkul W(2), Silpasakorn S(3), Wongsawat E(3), Waywa D(3), Suputtamongkol Y(3).

Author information:
(1)Department of Medicine, Maharat Nakhon Ratchasima Hospital, Nakhon Ratchasima, Thailand. wthipmontree@gmail.com. (2)Department of Immunology, Faculty of Medicine Siriraj Hospital, Mahidol University, Bangkok, Thailand. (3)Department of Medicine, Faculty of Medicine Siriraj Hospital, Mahidol University, Bangkok, Thailand.

Scrub typhus is endemic in Thailand. Of the 495 patients with acute undifferentiated fever studied in Maharat Nakhon Ratchasima Hospital, Nakhon Ratchasima, Thailand, from June 1, 2011, to December 31, 2012, 146 patients (29.5%) had confirmed scrub typhus. The majority of cases were male, farmers, with the mean (± standard deviation) age of 54.1 ± 15.2 years. A total of 59 patients (40.4%) had eschar lesion. The commonest sites for an eschar in male patients were the perineum, inguinal, and buttock area; whereas in females, it was the head and neck area. Abnormal electrocardiogram was found in 39 of 79 patients (49.4%) with sinus tachycardia being the most frequent finding (17, 21.5%). A total of 73 patients (50%) had at least one complication. Myocarditis was the cause of complete heart block in a scrub typhus patient, and he fully recovered after receiving intravenous chloramphenicol treatment. The case fatality rate was 6.2% (nine deaths).The independent predictors for fatal outcome were age over 65 years (odds ratio [OR] = 14.49, 95% confidence interval [CI] = 1.26-166.44, P = 0.03), acute kidney injury (OR = 12.75, 95% CI = 1.77-92.07, P = 0.01), and hyperbilirubinemia (OR = 24.82, 95% CI = 2.12-286.61, P = 0.01). Early diagnosis and prompt appropriate treatment can improve the patient's outcome.

© The American Society of Tropical Medicine and Hygiene.

DOI: 10.4269/ajtmh.16-0088
PMID: 27573633 [PubMed - as supplied by publisher]

73. J Assoc Physicians India. 2014 Dec;62(12):19-23.

Clinical Manifestations and Complications of Scrub Typhus : A Hospital Based Study from North Eastern India.

Jamil M, Lyngrah KG, Lyngdoh M, Hussain M.

OBJECTIVES: To study the different clinical manifestations and complications associated with scrub typhus.
METHODS: A hospital based prospective observational study of patients of acute febrile illness above 18 years age. Diagnosis was based on clinical and serological data.
RESULTS: A total of 61 patients were diagnosed with scrub typhus, with males more than females. Most (42.37%) belonged to 18 to 30 years age group. Fever with headache was the commonest presentation (94.91%). Multiorgan dysfunction syndrome was the most common complication (16.94%). The mortality rate was 8.47%.
CONCLUSION: The study reveals the clinical manifestations and complications of a well known mite borne disease in the state of Meghalaya. The varied presentations and high mortality requires a high index of suspicion. The study highlights the clustering of cases during the months of September to November.

PMID: 26259418 [PubMed - indexed for MEDLINE]

74. Jpn J Infect Dis. 2014;67(2):115-7.

A case report of scrub typhus-associated hemophagocytic syndrome and a review of literature.

Lin YH(1), Lin YH, Shi ZY.

Author information:
(1)Division of Infection, Taiwan Landseed Hospital.

A 34-year-old woman presented with septic shock, disseminated intravascular coagulation (DIC), and multiorgan dysfunction with a 1-week history of fever, abdominal pain in the right upper quadrant, and dull pain in the right flank. Physical and laboratory data showed cytopenia (thrombocytopenia and anemia), splenomegaly, hyperferritinemia, hypofibrinogenemia, and an elevated level of interleukin-2 receptor (soluble CD25). Bone marrow examinations disclosed hypercellular marrow with increased infiltration of histiocytes with hemophagocytosis. This diagnosis was confirmed by positive Weil-Felix test results (Proteus mirabilis OX-K titer, 1:80), the presence of IgG and IgM antibodies, and positive PCR results for Orientia tsutsugamushi. The patient developed a severe intracranial hemorrhage 3 days after admission and expired due to systemic inflammatory response syndrome with DIC and multiorgan failure on the 13th day of hospitalization. Scrub typhus with hemophagocytic syndrome can be complicated by DIC and multiorgan failure. Patients with scrub typhus usually have an excellent response to treatment; therefore, early diagnosis and prompt administration of antimicrobial therapy may prevent the development of serious complications.

PMID: 24647254 [PubMed - indexed for MEDLINE]

75. Indian J Med Microbiol. 2016 Jul-Sep;34(3):293-8. doi: 10.4103/0255-0857.188315.

Scrub typhus and spotted fever among hospitalised children in South India: Clinical profile and serological epidemiology.

Kalal BS(1), Puranik P(2), Nagaraj S(1), Rego S(2), Shet A(2).

Author information:
(1)Department of Microbiology, St. John's Medical College Hospital, Bengaluru, Karnataka, India. (2)Department of Pediatrics, St. John's Medical College Hospital, Bengaluru, Karnataka, India.

BACKGROUND: Rickettsial infections are re-emerging. In India, they are now being reported from several areas where they were previously unknown.
OBJECTIVES: The objective of this study was to describe the epidemiology, clinical profile and outcome of serologically-confirmed scrub typhus and spotted fever among children in a tertiary care hospital in Bengaluru.
MATERIALS AND METHODS: Hospitalised children aged <18 years, with clinical features suggestive of rickettsial disease admitted between January 2010 and October 2012 were included prospectively. Diagnosis was based on scrub typhus and spotted fever-specific IgM and IgG by enzyme-linked immunosorbent assay (ELISA).
RESULTS: Of 103 children with clinical features suggestive of rickettsial illness, ELISA test confirmed 53 cases for scrub typhus, 23 cases for spotted fever group and 14 with mixed infection. The average age was 7.3 (±3.9) years and 44 (71.0%) children were male. Majority of cases were from Karnataka (50%), Andhra Pradesh (32.3%) and Tamil Nadu (17.7%). Common clinical features included fever (100%, average duration 11 days), nausea and vomiting (44%), rash (36%); eschar was rare. Compared to the ELISA test, Weil-Felix test (OX-K titre of 1:80) had a sensitivity and specificity of 88.7% and 43.9%, respectively. Treatment with chloramphenicol or doxycycline was given to the majority of the children. Complications included meningoencephalitis (28%), shock (10%), retinal vasculitis (10%) and purpura fulminans (7%).
CONCLUSIONS: These findings suggest that the burden of rickettsial infection among children in India is high, with a substantially high complication rate. Rickettsial-specific ELISA tests can help in early diagnosis and early institution of appropriate treatment that may prevent life-threatening complications.

DOI: 10.4103/0255-0857.188315
PMID: 27514949 [PubMed - in process]

76. Emerg Infect Dis. 2013 May;19(5):774-7. doi: 10.3201/eid1905.121445.

Scrub typhus outbreak, northern Thailand, 2006-2007.

Rodkvamtook W(1), Gaywee J, Kanjanavanit S, Ruangareerate T, Richards AL, Sangjun N, Jeamwattanalert P, Sirisopana N.

Author information:
(1)Armed Forces Research Institute of Medical Science, Bangkok, Thailand.

During a scrub typhus outbreak investigation in Thailand, 4 isolates of O.

tsutsugamushi were obtained and established in culture. Phylogenetic analysis based on the 56-kDa type-specific antigen gene demonstrated that the isolates fell into 4 genetic clusters, 3 of which had been previously reported and 1 that represents a new genotype.

DOI: 10.3201/eid1905.121445
PMCID: PMC3647508
PMID: 23647883 [PubMed - indexed for MEDLINE]

77. J Postgrad Med. 2013 Jul-Sep;59(3):177-8. doi: 10.4103/0022-3859.118033.

Eschar in scrub typhus: a valuable clue to the diagnosis.

Kundavaram AP(1), Jonathan AJ, Nathaniel SD, Varghese GM.

Author information:
(1)Department of Medicine, Christian Medical College, Vellore, Tamil Nadu, India.

Comment in
J Postgrad Med. 2013 Oct-Dec;59(4):343.
J Postgrad Med. 2013 Oct-Dec;59(4):342-3.

BACKGROUND: Scrub typhus is an acute febrile illness widely prevalent in the 'tsutsugamushi triangle' region of the world. Clinical features include fever, myalgia, headache, rash, and a pathognomonic eschar. An eschar is formed by the bite of chigger mite that inoculates the causative agent of Scrub typhus Orientia tsutsugamushi. The aim of this study is to determine the most common sites of eschars over the bodies of patients with Scrub typhus.
MATERIALS AND METHODS: In a retrospective analysis, we examined a total of 418 patients who presented to Christian Medical College, Vellore between 2009 and 2012 with an acute febrile illness and an eschar on clinical examination and confirmed to have scrub typhus with a positive Scrub typhus IgM ELISA test. We studied the distribution of eschars over the bodies of 418 patients with Scrub typhus.
RESULTS: There was a significant difference in the distribution of eschars between males and females with a preponderance of the chest and abdomen (42.3%) among females and the axilla, groin and genitalia (55.8%) in males. Some unusual sites of an eschar were the cheek, ear lobe and dorsum of the feet.
CONCLUSION: The eschar is the most useful diagnostic clue in patients with acute febrile illness in areas endemic for Scrub typhus and therefore should be thoroughly examined for its presence especially over the covered areas such as the groin, genitalia, infra-mammary area and axilla.

DOI: 10.4103/0022-3859.118033
PMID: 24029193 [PubMed - indexed for MEDLINE]

78. Vector Borne Zoonotic Dis. 2013 Mar;13(3):154-9. doi: 10.1089/vbz.2012.1059. Epub 2013 Feb 19.

Scrub typhus cases in a teaching hospital in Penghu, Taiwan, 2006-2010.

Wang YC(1), Chen PC, Lee KF, Wu YC, Chiu CH.

Author information:
(1)Department of Family Medicine, Tri-Service General Hospital, National Defense Medical Center, Taipei, Taiwan, Republic of China.

Scrub typhus is a mite-borne infectious disease caused by Orientia tsutsugamushi (previously called Rickettsia tsutsugamushi). The severity of this disease varies from only mild symptoms to death, and its manifestations are nonspecific. Therefore, clinicians may not correctly diagnose scrub typhus early enough for successful treatment. Reports of infections in travelers returning from Asia to their home countries are increasingly common. Thus, it is important that even clinicians in nonepidemic regions be alert for this disease. Here we describe the epidemiological aspects and clinical manifestations of scrub typhus encountered at a teaching hospital in Penghu, Taiwan, over the past 5 years. A total of 126 patients were confirmed to be positive for scrub typhus at the hospital from 2006 to 2010. All cases were confirmed by the Centers for Disease Control and Prevention or its contract laboratory through pathogen isolation and an indirect immunofluorescence assay. Medical records of these patients were reviewed, and demographic and clinical characteristics, laboratory data, seasonal data, geographic distribution, complications, and outcome were analyzed. The incidence of scrub typhus peaked in individuals aged 0-10 and 51-60 years, with the highest incidence among those ≤10 years of age. No significant difference was noted between sexes. Fever was the most common symptom (93.6%), followed by chills (23.8%), cough (18.3%), and headache (14.3%). Eschars were observed in 78 (61.9%) patients, with the axilla being the most frequent site (n=17; 21.8%). Most patients were retirees (n=63; 50%), followed by students (n=16; 12.7%). Patients were more likely to live in rural areas than urban areas. Scrub typhus was epidemic in the spring (April to June) and fall (October to December) in a bimodal distribution similar to that observed in Japan. Leukocytosis was not common, but most patients had abnormal C-reactive protein levels, thrombocytopenia, and elevated liver function test results. Residents of Penghu, particularly Makung City and Husi Township, as well as travelers to the region during the spring and fall seasons should be educated about the signs and symptoms of scrub typhus. All physicians who come into contact with individuals residing in or traveling to or from epidemic regions should remain alert about the manifestations of this disease.

DOI: 10.1089/vbz.2012.1059
PMID: 23421889 [PubMed - indexed for MEDLINE]

79. Int J Infect Dis. 2013 Aug;17(8):e644-5. doi: 10.1016/j.ijid.2012.12.023. Epub 2013 Feb 9.

Scrub typhus complicated by acute respiratory distress syndrome and acute liver failure: a case report from Northeast India.

Goswami D(1), Hing A, Das A, Lyngdoh M.

Author information:
(1)Department of General Medicine, North Eastern Indira Gandhi Regional Institute of Health and Medical Science, Mawdiangdiang, Shillong, Meghalaya, India.

Comment in

Int J Infect Dis. 2013 Aug;17(8):e662.

Acute respiratory distress syndrome (ARDS) is a serious complication of scrub typhus. Only a few cases of scrub typhus complicated by ARDS have been discussed in the literature to date. Herein we report the case of a patient who presented with scrub typhus complicated by ARDS and acute liver failure (ALF) and who was successfully treated in our institute. Due to the non-specificity and diversity of the initial presenting symptoms, a lack of awareness about the disease amongst physicians, and the lack of accessibility to facilities for serodiagnosis in developing countries, there is a chance of misdiagnosis during the early stage. At the same time, early diagnosis and prompt treatment are crucial to prevent life-threatening complications. Our patient was initially misdiagnosed with a common cold and then malaria. By the time a correct diagnosis was made, complications had already developed. To the best of our knowledge, this is the first case of scrub typhus complicated by ARDS and ALF to be reported from the northeastern region of India.

Copyright © 2013 International Society for Infectious Diseases. Published by Elsevier Ltd. All rights reserved.

DOI: 10.1016/j.ijid.2012.12.023
PMID: 23402799 [PubMed - indexed for MEDLINE]

80. Korean J Intern Med. 2014 Mar;29(2):253-5. doi: 10.3904/kjim.2014.29.2.253. Epub 2014 Feb 27.

Diagnosis of scrub typhus: introduction of the immunochromatographic test in Korea.

Lee KD(1), Moon C(2), Oh WS(3), Sohn KM(4), Kim BN(5).

Author information:
(1)Division of Infectious Diseases, Department of Internal Medicine, Eulji General Hospital, Seoul, Korea. (2)Division of Infectious Diseases, Department of Internal Medicine, Inje University Busan Paik Hospital, Busan, Korea. (3)Division of Infectious Diseases, Department of Internal Medicine, Kangwon National University School of Medicine, Chuncheon, Korea. (4)Division of Infectious Diseases, Chungnam National University Hospital, Daejeon, Korea. (5)Department of Internal Medicine, Inje University Sanggye Paik Hospital, Seoul, Korea.

DOI: 10.3904/kjim.2014.29.2.253
PMCID: PMC3956999
PMID: 24648812 [PubMed - indexed for MEDLINE]

81. Am J Trop Med Hyg. 2013 Jul;89(1):123-9. doi: 10.4269/ajtmh.12-0728. Epub 2013 May 28.

Laboratory diagnosis and genotype identification of scrub typhus from Pinggu district, Beijing, 2008 and 2010.

Li W(1), Dou X, Zhang L, Lyu Y, Du Z, Tian L, Zhang X, Sun Y, Guan Z, Chen L, Li X, Wang Q.

Author information:
(1)Beijing Center for Disease Prevention and Control, Beijing, People's Republic of China. liweihong@yahoo.cn

This study was conducted to determine the diagnosis and genotype of Orientia tsutsugamushi in Pinggu district, Beijing. Indirect immunofluorescence assay (IFA) was performed to detect O. tsutsugamushi-specific immunoglobulin M (IgM) and immunoglobulin G (IgG) antibodies. Nested polymerase chain reaction (PCR) and DNA sequencing analysis targeting the O. tsutsugamushi-specific groEL gene and 56 kDa protein gene were performed on whole-blood samples from scrub typhus patients. We confirmed that 47 patients were infected with scrub typhus in Pinggu district, Beijing. Representative sequences amplified by primers according to the groEL gene (BJ-PG-2008; GenBank accession No. JQ894502) and the 56 kDa protein gene (PG-56kDa; GenBank accession No. JX843795) both clustered with Kawasaki. PG-56kDa had sequence homology of 100% with TADY12-0308, shandong-XDM2, Neimeng-90, and sdu-1 and sequence homology of 96% with Kawasaki, Taguchi, Oishi, and Kanda. We confirmed the genotype of O. tsutsugamushi in Pinggu district, Beijing, as Kawasaki, and the patient in 2008 confirmed in this study was the first patient with confirmed scrub typhus in Beijing.

DOI: 10.4269/ajtmh.12-0728
PMCID: PMC3748468
PMID: 23716411 [PubMed - indexed for MEDLINE]

82. Trop Doct. 2014 Jul;44(3):160-2. doi: 10.1177/0049475514530688. Epub 2014 Apr 15.

A prospective study on distribution of eschar in patients suspected of scrub typhus.

Munegowda KC(1), Nanda S(2), Varma M(3), Bairy I(4), Vidyasagar S(5).

Author information:
(1)Senior Research Fellow, Department of Microbiology, Melaka Manipal Medical College, India. (2)Postgraduate, Department of Medicine, Kasturba Medical College, India. (3)Associate Professor, Department of Medicine, Kasturba Medical College, India drmuralidharvarma@yahoo.co.in. (4)Professor, Department of Microbiology, Melaka Manipal Medical College, India. (5)Professor and Head, Department of Medicine, Kasturba Medical College, India.

Scrub typhus is an acute febrile illness caused by a tick bite infected with the bacteria Orientia tsutsugamushi The clinical diagnosis is difficult as the symptoms are similar to other febrile illnesses such as dengue, typhoid, leptospirosis and so on. An eschar, if present, will narrow down the provisional diagnosis towards scrub typhus. There are no data on the preferential sites of tick bites in an Indian population. We present here the preferential sites of tick bites in a South Indian population of 123 cases positive for eschar. Geographically, clothing styles vary leading to the differences in the areas of skin exposed to the bite and thus the formation of eschars. Scrub typhus, if not treated, may lead to fatal complications. As scrub typhus is one of the most under-reported illnesses in the world, clinicians should look for the presence of an eschar in the preferential sites at least to narrow down the diagnosis and treatment.

© The Author(s) 2014 Reprints and permissions:
sagepub.co.uk/journalsPermissions.nav.

DOI: 10.1177/0049475514530688
PMID: 24737886 [PubMed - in process]

83. Geospat Health. 2013 Nov;8(1):229-39.

Bayesian spatial modelling and the significance of agricultural land use to scrub typhus infection in Taiwan.

Wardrop NA, Kuo CC, Wang HC, Clements AC, Lee PF, Atkinson PM.

Scrub typhus is transmitted by the larval stage of trombiculid mites. Environmental factors, including land cover and land use, are known to influence breeding and survival of trombiculid mites and, thus, also the spatial heterogeneity of scrub typhus risk. Here, a spatially autoregressive modelling framework was applied to scrub typhus incidence data from Taiwan, covering the period 2003 to 2011, to provide increased understanding of the spatial pattern of scrub typhus risk and the environmental and socioeconomic factors contributing to this pattern. A clear spatial pattern in scrub typhus incidence was observed within Taiwan, and incidence was found to be significantly correlated with several land cover classes, temperature, elevation, normalized difference vegetation index, rainfall, population density, average income and the proportion of the population that work in agriculture. The final multivariate regression model included statistically significant correlations between scrub typhus incidence and average income (negatively correlated), the proportion of land that contained mosaics of cropland and vegetation (positively correlated) and elevation (positively correlated). These results highlight the importance of land cover on scrub typhus incidence: mosaics of cropland and vegetation represent a transitional land cover type which can provide favourable habitats for rodents and, therefore, trombiculid mites. In Taiwan, these transitional land cover areas tend to occur in less populated and mountainous areas, following the frontier establishment and subsequent partial abandonment of agricultural cultivation, due to demographic and socioeconomic changes. Future land use policy decision-making should ensure that potential public health outcomes, such as modified risk of scrub typhus, are considered.

DOI: 10.4081/gh.2013.69
PMID: 24258898 [PubMed - indexed for MEDLINE]

84. BMJ Case Rep. 2014 Apr 1;2014. pii: bcr2013201849. doi: 10.1136/bcr-2013-201849.

Pancreatitis in scrub typhus: a rare complication.

Dhakal M(1), Dhakal OP, Bhandari D.

Author information:
(1)Department of Medicine, Sikkim Manipal Institute of Medical Sciences, Gangtok, Sikkim, India.

Scrub typhus is a zoonosis transmitted by a trombiculid mite which introduces bacteria of Orientia tsutsugamushi by its bite. The acute febrile illness is characterised by eschar at the site of the bite with maculopapular rashes and local and/or generalised lymphadenopathy. The disease is endemic in the tsutsugamushi triangle. Sikkim, a small Himalayan north-eastern state, is also not unaffected where outbreaks of the disease have been reported. The clinical spectrum of the disease ranges from mild to fatal depending on the virulence of the bacterial strain, susceptibility of the host and promptness of the treatment. In severe cases, there can be multiple organ involvement. Pancreatitis is a serious and unusual complication of this disease, which was seen in our presentation. A 22-year-old man, diagnosed to have scrub typhus, developed pancreatitis in the second week of the illness and responded well to medical treatment.

DOI: 10.1136/bcr-2013-201849
PMCID: PMC3987298
PMID: 24692372 [PubMed - indexed for MEDLINE]

85. BMJ Case Rep. 2014 Jan 17;2014. pii: bcr2013200687. doi: 10.1136/bcr-2013-200687.

Massive consolidation: a rare manifestation of paediatric Scrub typhus.

Manickam K(1), Sunderkumar S, Chinnaraj S, Sivathanu S.

Author information:
(1)Department of Pediatrics, ESI-PGIMSR, Chennai, Tamil Nadu, India.

Despite resurgence in the number of Scrub typhus cases, it still poses a diagnostic challenge as there is no prototype presentation. We report a case of a child with Scrub typhus who developed a massive consolidation. Despite such an extensive consolidation, respiratory symptoms such as cough and breathlessness were inconspicuous thereby posing a diagnostic dilemma. Upon serological confirmation, doxycycline therapy was initiated with a rapid and complete resolution of the pneumonia, both clinically and radiologically. The case is being reported to highlight this unusual presentation of Scrub typhus in children.

DOI: 10.1136/bcr-2013-200687
PMCID: PMC3902318
PMID: 24443332 [PubMed - indexed for MEDLINE]

86. Natl Med J India. 2015 Jan-Feb;28(1):12-3.

Surgical manifestations of scrub typhus: A diagnostic dilemma.

Deshpande GA(1), Mittal R(2), Jesudasan MR(2), Perakath B(2).

Author information:
(1)Department of General Surgery, Christian Medical College, Vellore 632004, Tamil Nadu, gayatrisabne@rediffmail.com. (2)Department of General Surgery, Christian Medical College, Vellore 632004, Tamil Nadu.

BACKGROUND: Scrub typhus, a zoonosis caused by Orientia tsutsugamushi, is a systemic febrile illness. The disease presents with diverse clinical manifestations, ranging from subclinical disease to multiorgan failure and fatal disease. It may rarely present as an acute abdomen which may lead to a diagnostic dilemma. We describe two serologically confirmed cases of scrub typhus presenting as acute abdomen-one mimicking acute appendicitis and the other acute cholecystitis, both managed non-operatively. A high index of suspicion, along with subtle indicators in the history and clinical examination help avoid unnecessary surgery in such cases.

Copyright 2015, NMJI.

PMID: 26219315 [PubMed - indexed for MEDLINE]

87. Emerg Infect Dis. 2014 Mar;20(3):484-5. doi: 10.3201/eid2003.121349.

Mixed scrub typhus genotype, Shandong, China, 2011.

Zhang M, Zhao ZT, Wang XJ, Li Z, Ding L, Ding SJ, Yang LP.

DOI: 10.3201/eid2003.121349
PMCID: PMC3944857
PMID: 24565414 [PubMed - indexed for MEDLINE]

88. Trop Doct. 2016 Jul;46(3):170-2. doi: 10.1177/0049475515619512. Epub 2015 Dec 8.

Scrub typhus presenting as acute respiratory distress syndrome: case report.

Baruah U(1), Sachdeva HC(2), Sahni A(1).

Author information:
(1)Department of Anaesthesiology and Critical Care, Safdarjung Hospital, New Delhi, India. (2)Department of Anaesthesiology and Critical Care, Safdarjung Hospital, New Delhi, India sachdeva_hc04@yahoo.com.

DOI: 10.1177/0049475515619512
PMID: 26655686 [PubMed - in process]

89. Indian J Med Res. 2014 Dec;140(6):792.

Classical eschar in scrub typhus.

Bafna P, Kadhiravan T(1).

Author information:
(1)Department of Medicine, Jawaharlal Institute of Postgraduate Medical Education & Research, Dhanvantri Nagar, Puducherry 605 006, India.

PMCID: PMC4365358

PMID: 25758581 [PubMed - indexed for MEDLINE]

90. Intern Med. 2016;55(7):805-9. doi: 10.2169/internalmedicine.55.5304. Epub 2016 Apr 1.

The Rapid Effectiveness of Minocycline against Scrub Typhus Meningoencephalitis.

Naoi T(1), Shimazaki H, Sawada M.

Author information:
(1)Division of Neurology, Department of Internal Medicine, Shin-Oyama City Hospital, Japan.

Scrub typhus is associated with various clinical symptoms. However, the pathogenesis of scrub typhus infection remains to be elucidated. A 73-year-old man was admitted to our hospital with consciousness disturbance and suspected meningoencephalitis. The patient's laboratory data showed deterioration and were indicative of hemophagocytic lymphohistiocytosis (HLH). A whole body examination to detect the trigger disease revealed an eschar, which is a characteristic of scrub typhus, on his back. His symptoms showed dramatic improvement after the administration of minocycline (MINO). This case report highlights that the clinical course of a case of scrub typhus meningoencephalitis that was cured with MINO.

DOI: 10.2169/internalmedicine.55.5304
PMID: 27041169 [PubMed - in process]

91. N Engl J Med. 2015 Dec 17;373(25):2455. doi: 10.1056/NEJMicm1503639.

IMAGES IN CLINICAL MEDICINE. Scrub Typhus.

Lee CS(1), Hwang JH.

Author information:
(1)Chonbuk National University, Jeonju, South Korea lcsmd@jbnu.ac.kr.

DOI: 10.1056/NEJMicm1503639
PMID: 26672847 [PubMed - indexed for MEDLINE]

92. Clin Nucl Med. 2015 Oct;40(10):838-9. doi: 10.1097/RLU.0000000000000856.

Imaging of Scrub Typhus by PET/CT.

Lv J(1), Liu S, Pan Y, Ju H, Zhang Y.

Author information:
(1)From the *Department of Nuclear Medicine, Ruijin Hospital, Shanghai Jiao Tong University School of Medicine; and †Department of Nuclear Medicine, Cancer Hospital, Shanghai Fu Dan University School of Medicine, Shanghai, People's Republic of China.

A 19-year-old man had an unexplained fever, dizziness, headache, fatigue, and pain in the scrotum. An FDG PET/CT imaging was acquired to assess fever of unknown origin. The images showed multiple foci of increased FDG activity in the enlarged lymph nodes in the body. In addition, mildly increased activity in the enlarged spleen and lung bases was also noted. The patient was eventually diagnosed with scrub typhus based on positive results of the Weil-Felix agglutination test, eschar in the scrotum, and effective therapy.

DOI: 10.1097/RLU.0000000000000856
PMID: 26252322 [PubMed - indexed for MEDLINE]

93. BMC Res Notes. 2014 Oct 14;7:719. doi: 10.1186/1756-0500-7-719.

Emerging scrub typhus infection in the northern region of Sri Lanka.

Pradeepan JA, Ketheesan N, Murugananthan K(1).

Author information:
(1)Department of Pathology, Faculty of Medicine, University of Jaffna, Jaffna, Sri Lanka. kalamathy6@gmail.com.

BACKGROUND: There is an increasing trend in rickettsioses or typhus fevers in the island of Sri Lanka. The seroepidemiological mapping previously published did not include the northern region of the island. This study was conducted to demonstrate the presence of scrub typhus (ST) and to characterise the clinical presentation of ST in this region.
FINDINGS: Serum samples from patients (n = 64) with clinical symptoms suspected of typhus fever following exclusion of other common febrile illnesses commonly seen in the northern region of Sri Lanka were selected and screened for ST using specific IgM and IgG ELISA (ImBios, USA). ST was confirmed by serology in 54 patients, with typical eschar being found in 49 of cases positive for ST. Fever was the sole presenting complaint of these patients with the duration of febrile illness varying from 2-14 days. Of these patients 44.4% had regional lymphadenopathy, 18.5% hepatomegaly, 12.9% pneumonitis and 9.3% splenomegaly. None of the patients had a rash.
CONCLUSIONS: This study confirms the presence of high numbers of patients with ST in northern Sri Lanka. It was found that 84.4% of the patients presenting with clinical features of rickettsioses (54 of the 64) were seropositive for ST with a significant majority having a typical eschar. This data provided will enable clinicians to be vigilant of ST in this region and provide appropriate therapy and also facilitate planning for preventive measures aimed at reducing the burden of ST.

DOI: 10.1186/1756-0500-7-719
PMCID: PMC4216347
PMID: 25316171 [PubMed - indexed for MEDLINE]

94. PLoS One. 2013 Jun 14;8(6):e66595. doi: 10.1371/journal.pone.0066595. Print 2013.

Scrub typhus meningitis in South India--a retrospective study.

Viswanathan S(1), Muthu V, Iqbal N, Remalayam B, George T.

Author information:
(1)Department of General Medicine, Indira Gandhi Medical College, Kathirkamam, Pondicherry, India. stalinviswanathan@ymail.com

BACKGROUND: Scrub typhus is prevalent in India although definite statistics are not available. There has been only one study on scrub typhus meningitis 20 years ago. Most reports of meningitis/meningoencephalitis in scrub typhus are case reports.
METHODS: A retrospective study done in Pondicherry to extract cases of scrub typhus admitted to hospital between February 2011 and January 2012. Diagnosis was by a combination of any one of the following in a patient with an acute febrile illness--a positive scrub IgM ELISA, Weil-Felix test, and an eschar. Lumbar puncture was performed in patients with headache, nuchal rigidity, altered sensorium or cranial nerve deficits.
RESULTS: Sixty five cases of scrub typhus were found, and 17 (17/65) had meningitis. There were 33 males and 32 females. Thirteen had an eschar. Median cerebrospinal fluid (CSF) cell count, lymphocyte percentage, CSF protein, CSF glucose/blood glucose, CSF ADA were 54 cells/µL, 98%, 88 mg/dL, 0.622 and 3.5 U/mL respectively. Computed tomography was normal in patients with altered sensorium and cranial nerve deficits. Patients with meningitis had lesser respiratory symptoms and signs and higher urea levels. All patients had received doxycycline except one who additionally received chloramphenicol.
CONCLUSION: Meningitis in scrub typhus is mild with quick and complete recovery. Clinical features and CSF findings can mimic tuberculous meningitis, except for ADA levels. In the Indian context where both scrub typhus and tuberculosis are endemic, ADA and scrub IgM may be helpful in identifying patients with scrub meningitis and in avoiding prolonged empirical antituberculous therapy in cases of lymphocytic meningitis.

DOI: 10.1371/journal.pone.0066595
PMCID: PMC3682970
PMID: 23799119 [PubMed - indexed for MEDLINE]

95. J Vector Borne Dis. 2015 Sep;52(3):267-9.

Atypical eschar: An unusual cutaneous manifestation of scrub typhus.

Audhya M(1), Abirami D, Srikanth S.

Author information:
(1)Department of DVL, Mahatma Gandhi Medical College and Research Institute, Puducherry, India.

PMID: 26418661 [PubMed - indexed for MEDLINE]

96. Antimicrob Agents Chemother. 2014;58(3):1488-93. doi: 10.1128/AAC.01996-13. Epub 2013 Dec 23.

Outcome of intravenous azithromycin therapy in patients with complicated scrub typhus compared with that of doxycycline therapy using propensity-matched

analysis.

Jang MO(1), Jang HC, Kim UJ, Ahn JH, Kang SJ, Jung SI, Shin HY, Park KH.

Author information:
(1)Department of Infectious Disease, Chonnam National University Medical School, Gwang-ju, Republic of Korea.

There are no well-matched, controlled studies comparing azithromycin with doxycycline for the treatment of complicated scrub typhus. A retrospective propensity score-matched case-control study was performed for patients who presented with complicated scrub typhus and were treated with doxycycline or azithromycin between 2001 and 2011. Data on comorbidities, clinical manifestations, laboratory studies, treatments, and outcomes were extracted for analysis. The clinical characteristics and outcomes of the azithromycin-treated group (n=73) were compared to those of the doxycycline-treated group (n=108). Of 181 patients, 73 from each group were matched by propensity scores. There were no significant differences in baseline characteristics between the matched groups. The treatment success and survival rates were not significantly different (89% [65/73 patients] versus 96% [70/73 patients] and 96% [70/73 patients] versus 96% [70/73 patients], respectively [P>0.05]). No difference was observed in the time to defervescence or length of hospital stay between the two groups (P>0.05). In complicated scrub typhus patients (n=181), multivariate analysis showed that only APACHE II score was an independent risk factor for mortality (95% confidence interval, 1.11 to 1.56; P<0.001). Our data suggest that outcomes of azithromycin therapy are comparable to those of doxycycline therapy in patients with complicated scrub typhus.

DOI: 10.1128/AAC.01996-13
PMCID: PMC3957884
PMID: 24366734 [PubMed - indexed for MEDLINE]

97. BMC Infect Dis. 2013 Apr 29;13:191. doi: 10.1186/1471-2334-13-191.

Scrub typhus islands in the Taiwan area and the association between scrub typhus disease and forest land use and farmer population density: geographically weighted regression.

Tsai PJ(1), Yeh HC.

Author information:
(1)Center for General Education, Aletheia University, New Taipei 25103, Republic of China. puijentsai@gmail.com

BACKGROUND: The Taiwan area comprises the main island of Taiwan and several small islands located off the coast of the Southern China. The eastern two-thirds of Taiwan are characterized by rugged mountains covered with tropical and subtropical vegetation. The western region of Taiwan is characterized by flat or gently rolling plains. Geographically, the Taiwan area is diverse in ecology and environment, although scrub typhus threatens local human populations. In this study, we investigate the effects of seasonal and meteorological factors on the incidence of scrub typhus infection among 10 local climate regions. The correlation between the spatial distribution of scrub typhus and cultivated

forests in Taiwan, as well as the relationship between scrub typhus incidence and the population density of farm workers is examined.
METHODS: We applied Pearson's product moment correlation to calculate the correlation between the incidence of scrub typhus and meteorological factors among 10 local climate regions. We used the geographically weighted regression (GWR) method, a type of spatial regression that generates parameters disaggregated by the spatial units of analysis, to detail and map each regression point for the response variables of the standardized incidence ratio (SIR)-district scrub typhus. We also applied the GWR to examine the explanatory variables of types of forest-land use and farm worker density in Taiwan in 2005.
RESULTS: In the Taiwan Area, scrub typhus endemic areas are located in the southeastern regions and mountainous townships of Taiwan, as well as the Pescadore, Kinmen, and Matou Islands. Among these islands and low-incidence areas in the central western and southwestern regions of Taiwan, we observed a significant correlation between scrub typhus incidence and surface temperature. No similar significant correlation was found in the endemic areas (e.g., the southeastern region and the mountainous area of Taiwan). Precipitation correlates positively with scrub typhus incidence in 3 local climate regions (i.e., Taiwan's central western and southwestern regions, and the Kinmen Islands). Relative humidity correlates positively with incidence in Southwestern Taiwan and the Kinmen Islands. The number of wet days correlates positively with incidence in Southwestern Taiwan. The duration of sunshine correlates positively with incidence in Central Western Taiwan, as well as the Kinmen and Matou Islands. In addition, the 10 local climatic regions can be classified into the following 3 groups, based on the warm-cold seasonal fluctuations in scrub typhus incidence: (a) Type 1, evident in 5 local climate regions (Taiwan's northern, northwestern, northeastern, and southeastern regions, as well as the mountainous area); (b) Type 2 (Taiwan's central western and southwestern regions, and the Pescadore Islands); and (c) Type 3 (the Kinmen and Matou Islands). In the GWR models, the response variable of the SIR-district scrub typhus has a statistically significantly positive association with 2 explanatory variables (farm worker population density and timber management). In addition, other explanatory variables (recreational forests, natural reserves, and "other purpose" areas) show positive or negative signs for parameter estimates in various locations in Taiwan. Negative signs of parameter estimates occurred only for the explanatory variables of national protectorates, plantations, and clear-cut areas.
CONCLUSION: The results of this study show that scrub typhus in Taiwan can be classified into 3 types. Type 1 exhibits no climatic effect, whereas the incidence of Type 2 correlates positively with higher temperatures during the warm season, and the incidence of Type 3 correlates positively with higher surface temperatures and longer hours of sunshine. The results also show that in the mountainous township areas of Taiwan's central and southern regions, as well as in Southeastern Taiwan, higher SIR values for scrub typhus are associated with the following variables: farm worker population density, timber management, and area type (i.e., recreational forest, natural reserve, or other purpose).

DOI: 10.1186/1471-2334-13-191
PMCID: PMC3648375
PMID: 23627966 [PubMed - indexed for MEDLINE]

98. Trop Doct. 2014 Jan;44(1):43-5. doi: 10.1177/0049475513512640. Epub 2013 Nov 18.

Scrub typhus and malaria co-infection causing severe sepsis.

Mahajan SK(1), Kaushik M, Raina R, Thakur P.

Author information:
(1)Assistant Professor, Department of Medicine, IG Medical College, Shimla (HP), India.

We report a case of dual infection of scrub typhus and malaria in a 48-year-old woman.

DOI: 10.1177/0049475513512640
PMID: 24249027 [PubMed - indexed for MEDLINE]

99. Indian J Med Res. 2012 Dec;136(6):1020-4.

Scrub typhus in patients reporting with acute febrile illness at a tertiary health care institution in Goa.

Narvencar KP(1), Rodrigues S, Nevrekar RP, Dias L, Dias A, Vaz M, Gomes E.

Author information:
(1)Department of Medicine, Goa Medical College, Goa, India.
kedarnarvenkar@yahoo.com

BACKGROUND & OBJECTIVES: Scrub typhus is one of the differential diagnoses of haemorrhagic fevers especially if associated with jaundice and/or renal failure. Goa State in the western region of India has been witnessing increased incidence of such fevers, therefore, the present study was undertaken to identify whether scrub typhus is the aetiological agent.
METHODS: Adult patients presenting with undiagnosed febrile illness between June 2009 to October 2010, were evaluated. Testing was done using a commercial ELISA kit for specific IgM antibodies against Orientia tsutsugamushi.
RESULTS: Of the 44 patients included in the study, 15 (34%) were found to be positive for IGM antibodies against O. tsutsugamushi. The cases were seen mainly in the months between July to November. The common symptoms noted were fever, myalgias, gastrointestinal complaints, followed by breathlessness, rash and jaundice. The pathognomonic features such as eschar and lymphadenopathy were seen only in two patients. Nearly two third of the patients had leukocytosis (67%) and low serum albumin (60%). The most common complication noticed was hepatitis (80%) followed by acute respiratory distress syndrome (ARDS) (60%), thrombocytopenia (40%) and acute renal failure (33%). Five patients died in the course of illness.
INTERPRETATION & CONCLUSIONS: Our results showed that scrub typhus should be considered in the differential diagnosis of acute febrile illness associated with gastrointestinal symptoms, rash, myalgia , including those with organ dysfunctions such as hepatorenal syndrome, coagulopathy or ARDS. Empirical treatment with doxycycline or macrolides may be given in cases with strong suspicion of scrub typhus.

PMCID: PMC3612306
PMID: 23391799 [PubMed - indexed for MEDLINE]

100. Am J Trop Med Hyg. 2015 Jan;92(1):145-7. doi: 10.4269/ajtmh.14-0478. Epub 2014
Nov 10.

Neuritis and gastrointestinal hemorrhage in scrub typhus patients.

Kim DM(1), Yun NR(1), Lim SC(2).

Author information:
(1)Division of Infectious Diseases, Department of Internal Medicine, Chosun
University College of Medicine, Gwangju, Republic of Korea; Department of
Pathology, Chosun University College of Medicine, Gwangju, Republic of Korea.
(2)Division of Infectious Diseases, Department of Internal Medicine, Chosun
University College of Medicine, Gwangju, Republic of Korea; Department of
Pathology, Chosun University College of Medicine, Gwangju, Republic of Korea
sclim@chosun.ac.kr.

A 78-year-old woman with scrub typhus exhibited gastric hemorrhage with multiple
gastric ulcers. This is the first report to confirm pathologically the presence
of not only vasculitis but also, neuritis. The results suggest the necessity of
studies confirming neuritis as the cause of gastric ulcer and bleeding in scrub
typhus.

© The American Society of Tropical Medicine and Hygiene.

DOI: 10.4269/ajtmh.14-0478
PMCID: PMC4347371
PMID: 25385859 [PubMed - indexed for MEDLINE]

101. J Clin Microbiol. 2013 Aug;51(8):2787-90. doi: 10.1128/JCM.00463-13. Epub 2013
Jun 12.

Scrub typhus with sepsis and acute respiratory distress syndrome.

Kurup A(1), Issac A, Loh JP, Lee TB, Chua R, Bist P, Chao CC, Lewis M, Gubler DJ,
Ching WM, Ooi EE, Sukumaran B.

Author information:
(1)Mount Elizabeth Hospital, Singapore.

Scrub typhus is a major infectious threat in the Asia-Pacific region. We report
an unusual case of scrub typhus in a patient in Singapore who presented with
sepsis and acute respiratory distress syndrome but lacked the pathognomonic
eschar. The patient recovered after appropriate diagnosis and doxycycline
treatment. Rickettsial diseases should be included in the differential diagnosis
of febrile illnesses in regions where the diseases are endemic, and absence of
eschar should not be the criterion used to rule out scrub typhus.

DOI: 10.1128/JCM.00463-13
PMCID: PMC3719619
PMID: 23761149 [PubMed - indexed for MEDLINE]

102. Australas Med J. 2014 Mar 31;7(3):164-7. doi: 10.4066/AMJ.2014.1951. eCollection

2014.

Transient adrenal insufficiency and post-treatment bradycardia in scrub typhus - a case report.

Mookkappan S(1), Basheer A(1), Chidambaram S(1), Natarajan N(1), Shrimanth B(1).

Author information:
(1)Department of General Medicine, Pondicherry Institute of Medical Sciences.

Scrub typhus appears throughout the Asia-Pacific rim. This disease is known for its diverse clinical manifestations and complications. There is no literature on the association of scrub typhus with adrenal insufficiency. Relative bradycardia has been reported in scrub typhus during the febrile phase but not during convalescence. We report the case of a 45- year-old woman with scrub typhus whose blood pressure was persistently low due to acute adrenal insufficiency. Adrenal failure responded to supplementation with steroids. She also developed sinus bradycardia during the afebrile period following treatment.

DOI: 10.4066/AMJ.2014.1951
PMCID: PMC3973930
PMID: 24719653 [PubMed]

103. Trop Doct. 2014 Jul;44(3):156-9. doi: 10.1177/0049475514523761. Epub 2014 Feb 20.

Outbreak of scrub typhus in North India: a re-emerging epidemic.

Sethi S(1), Prasad A(2), Biswal M(3), Hallur VK(4), Mewara A(4), Gupta N(5), Galhotra S(4), Singh G(4), Sharma K(6).

Author information:
(1)Additional Professor, Department of Medical Microbiology, Postgraduate Institute of Medical Education and Research, Chandigarh, India sunilsethi10@hotmail.com. (2)Senior Resident, Department of Medical Microbiology, Postgraduate Institute of Medical Education and Research, Chandigarh, India sunilsethi10@hotmail.com. (3)Assistant Professor, Department of Medical Microbiology, Postgraduate Institute of Medical Education and Research, Chandigarh, India. (4)Senior Resident, Department of Medical Microbiology, Postgraduate Institute of Medical Education and Research, Chandigarh, India. (5)Deputy Director, National Center for Disease Control, New Delhi, India. (6)Associate Professor, Department of Medical Microbiology, Postgraduate Institute of Medical Education and Research, Chandigarh, India.

Scrub typhus is re-emerging in India. We describe an outbreak of 45 cases from our tertiary care center in north India. This outbreak included city dwellers who had no history of travel to hilly areas. The classical feature of scrub typhus, the eschar, was also noted rarely in these patients. The changing epidemiology of scrub typhus should be kept in mind while attending patients with acute febrile illness.

© The Author(s) 2014 Reprints and permissions: sagepub.co.uk/journalsPermissions.nav.

DOI: 10.1177/0049475514523761
PMID: 24557641 [PubMed - in process]

104. Am J Trop Med Hyg. 2013 May;88(5):932-6. doi: 10.4269/ajtmh.12-0325. Epub 2013 Mar 11.

Inter- and intra-operator variability in the reading of indirect immunofluorescence assays for the serological diagnosis of scrub typhus and murine typhus.

Phetsouvanh R(1), Thojaikong T, Phoumin P, Sibounheuang B, Phommasone K, Chansamouth V, Lee SJ, Newton PN, Blacksell SD.

Author information:
(1)Lao-Oxford-Mahosot Hospital-Wellcome Trust Research Unit, Microbiology Laboratory, Mahosot Hospital, Vientiane, Lao People's Democratic Republic. rattanaphone@tropmedres.ac

Inter- and intra-observer variation was examined among six microscopists who read 50 scrub typhus (ST) and murine typhus (MT) indirect immunofluorescence assay (IFA) immunoglobulin M (IgM) slides. Inter-observer agreement was moderate (κ = 0.45) for MT and fair (κ = 0.32) for ST, and was significantly correlated with experience (P = 0.03 and P = 0.004, respectively); κ-scores for intra-observer agreement between morning and afternoon readings (range = 0.35-0.86) were not correlated between years of experience for ST and MT IFAs (Spearman's ρ = 0.31, P = 0.54 and P = 0.14, respectively; P = 0.78). Storage at 4°C for 2 days showed a change from positive to negative in 20-32% of slides. Although the titers did not dramatically change after 14 days of storage, the final interpretation (positive to negative) did change in 36-50% of samples, and it, therefore, recommended that slides should be read as soon as possible after processing.

DOI: 10.4269/ajtmh.12-0325
PMCID: PMC3752761
PMID: 23478577 [PubMed - indexed for MEDLINE]

105. Am J Trop Med Hyg. 2013 Jul;89(1):119-22. doi: 10.4269/ajtmh.13-0094. Epub 2013 May 28.

Scrub typhus and cerebrovascular injury: a phenomenon of delayed treatment?

Chung JH(1), Yun NR, Kim DM, Lee JW, Yoon SH, Kim SW.

Author information:
(1)Department of Internal Medicine, Chosun University College of Medicine,Gwang-ju, Republic of Korea.

Abstract. Three patients diagnosed with scrub typhus through serology and polymerase chain reaction tests, experienced delayed administration of effective antibiotics after the appearance of symptoms, presented with subdural hemorrhage, intracerebral hemorrhage, or cerebral infarction in the late acute phase. Orientia tsutsugamushi should be considered as a causal or provoking factor for cerebrovascular accidents in regions where scrub typhus is endemic, especially in

those who receive delayed treatment.

DOI: 10.4269/ajtmh.13-0094
PMCID: PMC3748467
PMID: 23716407 [PubMed - indexed for MEDLINE]

106. Glob J Health Sci. 2013 Feb 15;5(3):101-14. doi: 10.5539/gjhs.v5n3p101.

Scrub typhus and comparisons of four main ethnic communities in taiwan in 2004 versus 2008 using geographically weighted regression.

Tsai PJ(1).

Author information:
(1)The Center for General Education, Aletheia University, New Taipei, Taiwan.
puijentsai@gmail.com

PURPOSE: On the main island of Taiwan, a higher risk of scrub typhus infection has been reported in endemic clusters in Southeastern Taiwan and in mountainous township areas. However, research on health care problems associated with scrub typhus in Taiwanese ethnic peoples is limited. This study employs spatial analysis of areal data to determine spatial features related to scrub typhus and the four main Taiwanese ethnicities: Hoklo, Hakka, Mainlander, and aboriginal communities, respectively.
METHODS: We used a GWR spatial method to analyze the local regressed relationships between scrub typhus incidence and ethnic community percentage in 349 townships in Taiwan, and the subsequent spatial regressed resultants and local parameter estimates were compared between two periods of 2004 and 2008 by kappa statistics.
RESULTS: In the GWR models, the spatial regressed relationships of scrub typhus incidences and the Hoklo communities showed significant and negative parameter estimates in numerous locations, showing clusters in Southeastern and Southwestern Taiwan, and areas of the central and southern mountainous townships. Both Hakka and Mainlander communities in the mountainous townships showed less-regressed clusters with scrub typhus prevalence. However, clusters of Aboriginal populations were positively correlated with scrub typhus in highly infected mountainous areas and in Southeastern Taiwan. The kappa value results and the comparisons of local parameter estimates in the 349 townships in Taiwan between 2004 and 2008 indicated that the incidence of scrub typhus in the Hoklo communities was substantial, in the Hakka communities was fair, in the Mainlander communities was slight, and in the aboriginal communities was moderate, respectively.
CONCLUSION: The aboriginal communities have been closely associated with higher risks of scrub typhus in the mountainous townships and in the southeastern portion of Taiwan.

DOI: 10.5539/gjhs.v5n3p101
PMCID: PMC4776805
PMID: 23618480 [PubMed - indexed for MEDLINE]

107. J Formos Med Assoc. 2013 Apr;112(4):201-7. doi: 10.1016/j.jfma.2012.02.002. Epub 2012 May 22.

Milder clinical manifestation of scrub typhus in Kinmen, Taiwan.

Su TH(1), Liu CJ, Chen DS, Kao JH.

Author information:
(1)Graduate Institute of Clinical Medicine, National Taiwan University College of Medicine, Taipei, Taiwan.

BACKGROUND/PURPOSE: Kinmen, an offshore island of Taiwan, is a popular location for sightseeing and an important entry port between Taiwan and China. Kinmen is also highly endemic for scrub typhus. The authors aimed to investigate the disease characteristics there, which remained largely unknown.
METHODS: The authors conducted a retrospective study on patients with scrub typhus in Kinmen during 2005-2008. The clinical information was reviewed from medical records for statistical analysis.
RESULTS: There were 261 patients with scrub typhus included with a bimodal summer-autumn type of distribution, with most patients (40%) age 20-29 years and a large proportion of patients (26%) older than 60 years. The disease manifestation, laboratory examinations, and treatment outcomes were comparable in summer and autumn. Fever (97%), eschar (93%), and relative bradycardia (67%) were the most common presentations, whereas lymphadenopathy (18%) and skin rash (8%) were infrequent. Elevated liver function, C-reactive protein levels, and low to normal platelet counts were frequent findings. A correct diagnosis was made made in an average 3.7 days after fever or 1.6 visits of medical consultation, and minocycline was prescribed in a timely manner. Most patients had good recovery and only 12 patients (5%) had severe infection with acute renal failure, shock, gastrointestinal bleeding, or respiratory failure; no mortality was found. Older age, longer fever duration, thrombocytopenia, abnormal liver and renal function, hyponatremia, and elevated C-reactive protein levels were significantly associated with severe complications and prolonged treatment duration.
CONCLUSION: A unique summer-autumn type of scrub typhus with milder disease manifestations is identified in Kinmen. The younger patient population, rapid diagnosis, and prompt treatment may be associated with a shortened disease course and lead to a better outcome.

Copyright © 2012. Published by Elsevier B.V.

DOI: 10.1016/j.jfma.2012.02.002
PMID: 23537866 [PubMed - indexed for MEDLINE]

108. J Clin Imaging Sci. 2015 Feb 27;5:11. doi: 10.4103/2156-7514.152340. eCollection 2015.

Role of advanced MRI brain sequences in diagnosing neurological complications of scrub typhus.

Sood S(1), Sharma S(1), Khanna S(1).

Author information:
(1)Department of Radiodiagnosis and Imaging, Indira Gandhi Medical College, Shimla, Himachal Pradesh, India.

Scrub typhus is a rare disease affecting many organs and causing vasculitis by affecting the endothelium of blood vessels. Review of literature shows that there are only a few case reports describing the neuroradiological manifestations of scrub typhus. This case report describes how newer and advanced MRI sequences are able to diagnose neurological complications of scrub typhus, such as hemorrhages, meningoencephalitis, infarctions, cranial nerve involvement, thrombosis, and hypoperfusion, that are not picked up on routine magnetic resonance imaging (MRI) sequences.

DOI: 10.4103/2156-7514.152340
PMCID: PMC4374194
PMID: 25861545 [PubMed]

109. BMJ Case Rep. 2014 Oct 29;2014. pii: bcr2014207824. doi: 10.1136/bcr-2014-207824.

A typical case of scrub typhus (tsutsugamushi disease).

Fujiwara S(1), Shimizu T(2).

Author information:
(1)Iwate Prefectural Chubu Hospital, Iwate, Japan. (2)Joto Hospital, Tokyo, Japan.

DOI: 10.1136/bcr-2014-207824
PMID: 25355755 [PubMed - indexed for MEDLINE]

110. BMJ Case Rep. 2016 May 9;2016. pii: bcr2015213929. doi: 10.1136/bcr-2015-213929.

Scrub typhus masquerading as acute pancreatitis.

Chaturvedi A(1), Gupta M(2), Bhardwaj S(1), Handa D(1).

Author information:
(1)Department of General Medicine, Government Medical College and Hospital, Chandigarh, Punjab, India. (2)Department of Medicine, Government Medical College and Hospital, Chandigarh, Punjab, India.

The clinical spectrum of scrub typhus ranges from mild to fatal depending on the virulence of bacterial strain, susceptibility of the host and promptness with which treatment is started. We report a case of a 14-year-old child with scrub typhus who developed acute pancreatitis. On serological confirmation, doxycycline therapy was started. The patient responded well and had no complications on follow-up. This case report highlights the importance of recognising an uncommon presentation of this common tropical disease, and its prompt diagnosis and early treatment for prevention of serious complications of the condition.

2016 BMJ Publishing Group Ltd.

DOI: 10.1136/bcr-2015-213929
PMID: 27161204 [PubMed - in process]

111. J Gastroenterol Hepatol. 2015 Mar;30(3):435. doi: 10.1111/jgh.12824.

Education and imaging. Gastrointestinal: a case of histologically confirmed scrub typhus gastritis.

Min KW(1), Kim DY, Lee SO, Hong SM.

Author information:
(1)Department of Pathology, Asan Medical Center, University of Ulsan College of Medicine, Seoul, Korea.

DOI: 10.1111/jgh.12824
PMID: 25707787 [PubMed - indexed for MEDLINE]

112. J Clin Microbiol. 2011 Jul;49(7):2584-9. doi: 10.1128/JCM.00355-11. Epub 2011 May 18.

Genotype diversity and distribution of Orientia tsutsugamushi causing scrub typhus in Thailand.

Ruang-Areerate T(1), Jeamwattanalert P, Rodkvamtook W, Richards AL, Sunyakumthorn P, Gaywee J.

Author information:
(1)Research Division, Armed Forces Research Institute of Medical Science, Bangkok 10400, Thailand. toonr@afrims.go.th

Scrub typhus, caused by antigenically disparate isolates of Orientia tsutsugamushi, is a widely distributed mite-borne human disease in the Asia Pacific region. Information regarding the heterogeneity of the immunodominant 56-kDa type-specific antigen (TSA) gene is crucial for the design and evaluation of scrub typhus-specific diagnostic assays and vaccines. Using indirect immunofluorescence assays (IFA) and PCR assays, O. tsutsugamushi was detected samples from rodents and patients with fever of unknown origin obtained from six provinces of Thailand during 2004 to 2007. Sequences were determined for a fragment of the 56-kDa TSA gene, and the relationship between these sequences and those previously determined were assessed. The phylogenetic analyses of partial 56-kDa TSA gene sequences demonstrated wide diversity and distribution of O. tsutsugamushi genotypes in Thailand. Furthermore, the genetic diversity grouped the scrub typhus agents into two commonly and five infrequently found genotypes within six provinces of Thailand. The two most commonly found genotypes of O. tsutsugamushi described in this study do not associate with the prototype strains that are widely used for the design and evaluation of diagnostic assays and vaccine candidates. Thus, these new genotypes should be considered for future scrub typhus assay and vaccine development.

DOI: 10.1128/JCM.00355-11
PMCID: PMC3147819
PMID: 21593255 [PubMed - indexed for MEDLINE]

113. Emerg Med Australas. 2012 Oct;24(5):577-80. doi: 10.1111/j.1742-6723.2012.01600.x.

Case of scrub typhus complicated by severe disseminated intravascular coagulation and death.

Ono Y(1), Ikegami Y, Tasaki K, Abe M, Tase C.

Author information:
(1)Department of Emergency and Critical Care Medicine, Fukushima Medical University, Fukushima, Japan. windmill@fmu.ac.jp

Scrub typhus is an infectious disease that is caused by Orientia tsutsugamushi. The authors describe an autopsied case of scrub typhus complicated with severe disseminated intravascular coagulation (DIC). An 82-year-old man complained of fever 4 days after climbing a mountain. The patient was admitted to an urban hospital, and meropenem and ceftriaxone were administered. The patient's condition deteriorated and he was transferred to a second hospital. On physical examination, a black scab was found and scrub typhus was suspected. Despite intensive treatment, the patient died on the fifth day. High levels of O. tsutsugamushi IgM antibody were confirmed. An autopsy revealed systemic vasculitis and perivasculitis. The endothelial tissue of the white pulp of the spleen was markedly infiltrated by plasma cells. The authors speculated that a severe immune reaction against O. tsutsugamushi enhanced an inflammatory response, leading to DIC. This case is a warning to doctors who are not familiar with scrub typhus.

© 2012 The Authors. EMA © 2012 Australasian College for Emergency Medicine and Australasian Society for Emergency Medicine.

DOI: 10.1111/j.1742-6723.2012.01600.x
PMID: 23039302 [PubMed - indexed for MEDLINE]

114. J Trop Pediatr. 2013 Feb;59(1):67-9. doi: 10.1093/tropej/fms030. Epub 2012 Jun 25.

Life-threatening scrub typhus with hemophagocytosis and acute respiratory distress syndrome in an infant.

Kwon HJ(1), Yoo IH, Lee JW, Chung NG, Cho B, Kim HK, Kang JH.

Author information:
(1)Department of Pediatrics, College of Medicine, Korea University, 152-703 Seoul, Republic of Korea.

Scrub typhus is a rickettsial disease, caused by Orientia tsutsugamushi, which is transmitted via the bite of a chigger. This disease is one of the most important infectious diseases in the Asia-Pacific area; however, a severe infant case has not yet been reported. Here, we present the case of an 8-month-old boy with scrub typhus accompanied by hemophagocytic lymphohistiocytosis (HLH). His rapid course was complicated by acute respiratory distress syndrome (ARDS), status epilepticus and disseminated intravascular coagulation (DIC). He recovered after clarithromycin therapy and intensive supportive care. Although being extremely rare, scrub typhus can be life-threatening in an infant; therefore, physicians in endemic countries should be aware of the necessity for early recognition and

prompt treatment of suspected cases.

DOI: 10.1093/tropej/fms030
PMID: 22735791 [PubMed - indexed for MEDLINE]

115. Zhonghua Liu Xing Bing Xue Za Zhi. 2016 Aug 10;37(8):1112-6. doi: 10.3760/cma.j.issn.0254-6450.2016.08.012.

[Study on the epidemiological characteristics and influencing factors of scrub typhus in the autumn-winter natural foci, from 2006 to 2013].

[Article in Chinese]

Sun Y(1), Fang LQ, Cao WC.

Author information:
(1)The State Key Laboratory of Pathogen and Biosecurity, Beijing Institute of Microbiology and Epidemiology, Beijing 100071, China.

OBJECTIVE: To investigate the spatiotemporal expansion and risk factors of scrub typhus (ST) in Shandong, Jiangsu and Anhui provinces.
METHODS: Based on the reported cases in Shandong and Jiangsu from 2006 to 2013, and Anhui from 2008 to 2013 epidemiological characteristics of ST and associated environmental factors were analyzed, using the panel negative binomial regression model.
RESULTS: A total of 2 968, 2 331 and 3 447 ST cases were respectively reported in Shandong, Jiangsu and Anhui during 2006-2013, with the average annual incidence rates as 0.39, 0.38 and 0.94 per 100 000 population. Uptrend in Shandong and Jiangsu, but a slight rollback seen in Anhui were observed. Expansion of natural foci was found in the 3 provinces, with affected counties accounted for 38.0%, 48.2% and 46.5% in Shandong, Jiangsu and Anhui, respectively in 2013. Cases were clustered in autumn but with a peak of single epidemic appeared in October or November, in all the 3 provinces. More female and elderly patients were seen, than in the other age groups, in all the 3 provinces. Majority of the cases were farmers, with an increasing trend in incidence, in all these provinces, followed by housekeepers in Shandong and preschool children in Anhui. The risk factors of transmission in all the 3 provinces were negatively associated with the monthly precipitation, and showing an " inverted-U" pattern in association with monthly temperature. A positive relationship between the risk of transmission and monthly relative humidity was found in Shandong and Jiangsu provinces. However, an " inverted-U" pattern between the risk of transmission and the monthly sunshine hour, appeared in Shandong and Anhui provinces. The incidence of Scrub typhus in Shandong was also positively related to the coverage of forest.
CONCLUSION: Surveillance programs and health education measures should focus on the warm and moist areas in all the 3 provinces, and also on forestry areas in Shandong. Health education and prevention on mite-bite should be targeted, especially on high-risk populations as the elderly and farmers, in the 3 provinces. Housekeepers in Shandong and children in Anhui should also be under special concern.

PMID: 27539343 [PubMed - in process]

116. Osong Public Health Res Perspect. 2013 Feb;4(1):39-44. doi: 10.1016/j.phrp.2013.01.002.

Are there spatial and temporal correlations in the incidence distribution of scrub typhus in Korea?

Noh M(1), Lee Y, Chu C, Gwack J, Youn SK, Huh S.

Author information:
(1)Department of Statistics, Pukyong National University, Busan, Korea .

OBJECTIVES: A hierarchical generalized linear model (HGLM) was applied to estimate the transmission pattern of scrub typhus from 2001 to 2011 in the Republic of Korea, based on spatial and temporal correlation.
METHODS: Based on the descriptive statistics of scrub typhus incidence from 2001 to 2011 reported to the Korean Centers for Disease Control and Prevention, the spatial and temporal correlations were estimated by HGLM. Incidences according to age, sex, and year were also estimated by the best-fit model out of nine HGLMs. A disease map was drawn to view the annual regional spread of the disease.
RESULTS: The total number of scrub typhus cases reported from 2001 to 2011 was 51,136: male, 18,628 (36.4%); female, 32,508 (63.6%). The best-fit model selected was a combination of the spatial model (Markov random-field model) and temporal model (first order autoregressive model) of scrub typhus transmission. The peak incidence was 28.80 per 100,000 persons in early October and the peak incidence was 40.17 per 100,000 persons in those aged 63.3 years old by the best-fit HGLM. The disease map showed the spread of disease from the southern central area to a nationwide area, excepting Gangwon-do (province), Gyeongsangbuk-do (province), and Seoul.
CONCLUSION: In the transmission of scrub typhus in Korea, there was a correlation to the incidence of adjacent areas, as well as that of the previous year. According to the disease map, we are unlikely to see any decrease in the incidence in the near future, unless ongoing aggressive measures to prevent the exposure to the vector, chigger mites, in rural areas, are put into place.

DOI: 10.1016/j.phrp.2013.01.002
PMCID: PMC3747676
PMID: 24159528 [PubMed]

117. Korean J Parasitol. 2012 Dec;50(4):327-31. doi: 10.3347/kjp.2012.50.4.327. Epub 2012 Nov 26.

Monthly occurrence of vectors and reservoir rodents of scrub typhus in an endemic area of Jeollanam-do, Korea.

Lee SH(1), Lee YS, Lee IY, Lim JW, Shin HK, Yu JR, Sim S.

Author information:
(1)Department of Microbiology, Konkuk University School of Medicine, Chungju 380-701, Korea.

Monthly surveys were conducted to investigate the occurrence of chigger mites and seroprevalence of scrub typhus among small mammals in Jeollanam-do, the

southwestern part of Korea, from November 2006 through October 2007. Fifty-eight small mammals, including 57 Apodemus agrarius (98.3%) and 1 Crocidura lasiura (1.7%), were captured, and a total of 4,675 chigger mites representing 4 genera and 8 species were collected from them. The chigger infestation rate among small mammals was 69.0%. The most predominant species in A. agrarius was Leptotrombidium scutellare (54.0%), followed by Leptotrombidium pallidum (39.4%), Leptotrombidium orientale (4.4%), Leptotrombidium palpale (1.1%), Neotrombicula tamiyai (0.6%), Eushoengastia koreaensis (0.3%), Neotrombicula gardellai (0.3%), and Cheladonta ikaoensis (<0.1%). The chigger index of A. agrarius was the highest in October (740.0), followed by November (242.0), September (134.6), March (98.3), February (38.2), January (35.3), December (34.5), April (30.8), and May (1.7). The average antibody positive rate of scrub typhus in wild rodents was 50.0%. The seropositive rates were high in October (100.0%) and November (83.3%), whereas those in other months were relatively low (28.6-57.1%). The chigger index of L. scutellare rapidly increased in September to form an acuminate peak in October, followed by a gradual decline. These results suggest that the outbreak of scrub typhus in the southwestern part of Korean peninsula is mostly due to L. scutellare.

DOI: 10.3347/kjp.2012.50.4.327
PMCID: PMC3514424
PMID: 23230330 [PubMed - indexed for MEDLINE]

118. Zhonghua Liu Xing Bing Xue Za Zhi. 2012 Jul;33(7):698-701.

[Study on the characteristics of temporal distribution and the epidemic trend of autumn-winter type scrub typhus under time series analysis].

[Article in Chinese]

Ding L(1), Ding SJ, Zhang M, Wang XJ, Li Z, Zhao ZT.

Author information:
(1)Institute of Epidemiology and Health Statistics, School of Public Health, Shandong University, Jinan 250012, China.

OBJECTIVE: To explore the characteristics of temporal distribution and epidemic trend of autumn-winter type scrub typhus using the time series analysis.
METHODS: Based on the data of scrub typhus collected from Shandong Diseases Reporting Information System from 2006 to 2011, both spectral analysis and moving average analysis were used to analyze the annual data of scrub typhus while scrub typhus incidence in 2012 - 2014 was forecasted. Seasonal decomposition analysis was applied to analyze the monthly data from January of 2006 to October of 2011, followed by Autoregressive Integrated Moving Average Model (ARIMA) which was constructed to forecast case number in November and December of 2011 and compared to the actual incidence.
RESULTS: The results of spectral analysis showed that the prevalence of autumn-winter type scrub typhus had a feature of '3-year-periodicity'. A long-term up-trend was confirmed by method of moving average analysis, with annually case numbers of 310, 337 and another number of 366 forecasted for 2012 to 2014, respectively, with the annual increase rate as 9% per-year. Data from analysis of monthly data of scrub typhus showed that through multiple seasonal decomposition analysis, the results indicated that the prevalence of this disease

possessed a typical autumn-winter type. The seasonality indexes for scrub typhus
in October and November were 8.454 and 2.230, respectively, while others were
less than 1.000. The ARIMA(0,1,1) (0,1,0)(12) model of (1-B)(1-B(12))X(t) =
(1-0.811B)u(t) that was used to forecast the prevalence of autumn-winter type
scrub typhus and was constructed with the residual error of 16 lags as white
noise. The Box-Jenkins test statistic for the model was 3.116, giving a P value
of 0.999. The model fitted the data well. Good accordance was achieved between
the observed values and the forecasted values of scrub typhus in November and
December of 2011 which was produced by the ARIMA model, and all observed values
were within the forecasted 95%CI.
CONCLUSION: The prevalence of autumn-winter type scrub typhus showed a
3-year-periodicity, with a long-term up-trend, and the case numbers of 2012 to
2014 were forecasted, rising on the end with an increasing rate of 9% per year,
which occurred seasonally with October as the peak time in every year. The
ARIMA(0,1,1) (0,1,0)(12) model seemed to be quite appropriate in predicting the
autumn-winter type scrub typhus.

PMID: 22968019 [PubMed - indexed for MEDLINE]

119. Am J Trop Med Hyg. 2013 Aug;89(2):301-7. doi: 10.4269/ajtmh.13-0064.

Unresolved problems related to scrub typhus: a seriously neglected
life-threatening disease.

Paris DH(1), Shelite TR, Day NP, Walker DH.

Author information:
(1)Mahidol Oxford Tropical Medicine Research Unit, Faculty of Tropical Medicine,
Mahidol University, Bangkok, Thailand. parigi@tropmedres.ac

DOI: 10.4269/ajtmh.13-0064
PMCID: PMC3741252
PMID: 23926142 [PubMed - indexed for MEDLINE]

120. Am J Trop Med Hyg. 2012 Dec;87(6):1099-104. doi: 10.4269/ajtmh.2012.12-0306. Epub
2012 Oct 22.

Scrub typhus: surveillance, clinical profile and diagnostic issues in Shandong,
China.

Zhang M(1), Zhao ZT, Wang XJ, Li Z, Ding L, Ding SJ.

Author information:
(1)Department of Epidemiology and Health Statistics, School of Public Health,
Shandong University, Jinan, Shandong Province, People's Republic of China.

To elucidate the epidemic status, clinical profile, and current diagnostic issues
of scrub typhus in Shandong Province, we analyzed the surveillance data of scrub
typhus from 2006 to 2011 and conducted a hospital-based disease survey in 2010.
Scrub typhus was clustered in mountainous and coastal areas in Shandong Province,
with an epidemic period from September to November. The most common

manifestations were fever (100%), eschar or skin ulcer (86.3%), fatigue (71.6%), anorexia (71.6%), and rash (68.6%). Predominant complications included bronchopneumonia, toxic hepatitis, and acute cholecystitis in 21.6%, 3.9%, and 2.9% of the cases, respectively. Severe complications including toxic myocarditis, heart failure, pneumonedema, pleural effusion, and emphysema were first reported in Shandong. Missed and delayed diagnosis of scrub typhus was common in local medical institutions. Alarm should be raised for changes of clinical features and current diagnostic issues of scrub typhus in newly developed endemic areas.

DOI: 10.4269/ajtmh.2012.12-0306
PMCID: PMC3516082
PMID: 23091193 [PubMed - indexed for MEDLINE]

121. Trop Doct. 2013 Jan;43(1):41-2. doi: 10.1177/0049475513480775.

Pure cerebellitis due to scrub typhus: a unique case report.

Karanth SS(1), Gupta A, Prabhu M.

Author information:
(1)Department of Medicine, Kasturba Medical College, Manipal, India.

We report the case of a 24-year old Indian man who presented with: high fever; drowsiness; an eschar and gross cerebellar dysfunction with horizontal gaze nystagmus; ataxic speech; and truncal ataxia. Scrub typhus was diagnosed by serological tests. This is the first case of a pure cerebellar involvement as the only manifestation of scrub typhus in the published literature.

DOI: 10.1177/0049475513480775
PMID: 23550204 [PubMed - indexed for MEDLINE]

122. J Postgrad Med. 2012 Oct-Dec;58(4):296-7. doi: 10.4103/0022-3859.105453.

Opsoclonus in scrub typhus.

D'sa S(1), Singh S, Sowmya S.

Author information:
(1)Department of Medicine, Christian Medical College, Vellore, Tamil Nadu, India.

Scrub typhus is a mite borne infectious disease caused by Orientia tsutsugamushi. It is a common cause of undifferentiated febrile illness in the Indian subcontinent. We present a case of scrub typhus with a rare ophthalmic manifestation.Our patient presented with fever and opsoclonus, was diagnosed to have scrub typhus and completely improved upon treatment. Opsoclonus complicates various medical diseases, including viral infections, toxin, encephalitis, brain tumors, and paraneoplastic syndromes. There has been only one previously reported case of opsoclonus in scrub typhus. This phenomenon highlights the increasingly complex presentation of common diseases. It also indicates there is much to be discovered about the immunopathogenesis of this infectious disease.

DOI: 10.4103/0022-3859.105453
PMID: 23298927 [PubMed - indexed for MEDLINE]

123. Indian J Pediatr. 2012 Nov;79(11):1459-62. doi: 10.1007/s12098-012-0721-0. Epub
2012 Feb 29.

Clinical profile of scrub typhus in children.

Palanivel S(1), Nedunchelian K, Poovazhagi V, Raghunadan R, Ramachandran P.

Author information:
(1)Department of Pediatrics, Institute of Child Health and Hospital for Children,
Egmore, Chennai-8, Tamil Nadu, India. drpalanivel84@gmail.com

OBJECTIVE: To study the clinical profile and outcome of scrub typhus cases
admitted in an urban referral centre.
METHODS: This descriptive study describes the clinical profile of 67 children
with scrub typhus, who were admitted in an urban referral centre(ICH &
HC,Chennai) during the period between October 2010 and March 2011.The diagnosis
was confirmed by IgM ELISA.
RESULTS: All children presented with fever. Eschar and rash were present in 46%
and 35% cases, respectively. Cough, vomiting, altered sensorium and oliguria were
present in 73%,59%,58% and 43%, respectively. Hepatosplenomegaly and pallor were
the commonest findings encountered in more than 80% children. Other findings were
edema, lymphadenopathy and icterus.Thrombocytopenia, elevated liver enzymes and
leukocytosis were seen in 77%, 64% and 49% cases, respectively. Pleural effusion,
ascites, shock and respiratory failure were seen in 61%, 47%,45%,34% cases,
respectively. Acute renal failure, hepatic failure, multiorgan dysfunction
syndrome (MODS), meningoencephalitis and acute respiratory distress
syndrome(ARDS) were seen in 10%,10%,7%,6% and 4% cases, respectively. Doxycycline
and azithromycin were the antibiotics used. The overall mortality rate was
11.94%. Causes of death were shock, ARDS, acute renal failure(ARF), MODS and
disseminated intravascular coagulation(DIVC).
CONCLUSIONS: When a child presents with acute febrile illness, maculopapular or
erythematous rash, hepatosplenomegaly, lymphadenopathy, thrombocytopenia and
features suggestive of capillary leak, diagnosis of Scrub typhus must be
considered and the child should be started on empirical therapy with doxycycline
or azithromycin which is life saving.

DOI: 10.1007/s12098-012-0721-0
PMID: 22374234 [PubMed - indexed for MEDLINE]

124. QJM. 2014 Jun;107(6):483. doi: 10.1093/qjmed/hct213. Epub 2013 Oct 22.

Scrub typhus after a trip to India.

Henry J(1), Beckwith H(1), Dubrey S(1).

Author information:
(1)The Department of Cardiology, Hillingdon Hospital, Pield Heath Road, Uxbridge,
Middlesex UB8 3NN, UK.email: simon.dubrey@thh.nhs.uk.

DOI: 10.1093/qjmed/hct213
PMID: 24149283 [PubMed - indexed for MEDLINE]

125. World J Gastroenterol. 2012 Sep 28;18(36):5138-41. doi: 10.3748/wjg.v18.i36.5138.

Scrub typhus hepatitis confirmed by immunohistochemical staining.

Chung JH(1), Lim SC, Yun NR, Shin SH, Kim CM, Kim DM.

Author information:
(1)Department of Internal Medicine, Chosun University College of Medicine, Gwangju 501-717, South Korea.

Scrub typhus is an acute febrile disease caused by Orientia tsutsugamushi (O. tsutsugamushi). We report herein the case of a woman who presented with fever and elevated serum levels of liver enzymes and who was definitively diagnosed with scrub typhus by histopathological examination of liver biopsy specimens, serological tests and nested polymerase chain reaction. Immunohistochemical staining using a monoclonal anti-O. tsutsugamushi antibody showed focally scattered positive immunoreactions in the cytoplasm of some hepatocytes. This case suggests that scrub typhus hepatitis causes mild focal inflammation due to direct liver damage without causing piecemeal necrosis or interface hepatitis. Thus, scrub typhus hepatitis differs from acute viral hepatitis secondary to liver damage due to host immune responses, which causes severe lobular disarray with diffuse hepatocytic degeneration, necrosis and apoptosis as well as findings indicative of hepatic cholestasis, such as hepatic bile plugs or brown pigmentation of hepatocytes.

DOI: 10.3748/wjg.v18.i36.5138
PMCID: PMC3460345
PMID: 23049227 [PubMed - indexed for MEDLINE]

126. Am J Trop Med Hyg. 2012 Jun;86(6):1046-8. doi: 10.4269/ajtmh.2012.11-0586.

Peritonitis in patients with scrub typhus.

Lee CH(1), Lee JH, Yoon KJ, Hwang JH, Lee CS.

Author information:
(1)Department of Internal Medicine, Chonbuk National University Medical School and Research Institute of Clinical Medicine, Jeonju, Republic of Korea.
lcsmd@jbnu.ac.kr

Various complications have been reported in scrub typhus cases including acute respiratory distress syndrome, encephalitis, pneumonia, pericarditis, acute renal failure, and acute hepatic failure. Few studies have reported on the gastrointestinal manifestations of scrub typhus. Typical gastrointestinal manifestations in patients with scrub typhus include abdominal pain, nausea, vomiting, hematemesis, melena, and diarrhea. The two cases presented in this study are the first reported cases of peritonitis associated with scrub typhus. This study shows that scrub typhus should also be included in the differential diagnosis of peritonitis in areas where Orientia tsutsugamushi is endemic.

DOI: 10.4269/ajtmh.2012.11-0586
PMCID: PMC3366520
PMID: 22665616 [PubMed - indexed for MEDLINE]

127. Indian J Med Microbiol. 2013 Jan-Mar;31(1):72-4. doi: 10.4103/0255-0857.108729.

Outbreak of scrub typhus in the North East Himalayan region-Sikkim: an emerging threat.

Gurung S(1), Pradhan J, Bhutia PY.

Author information:
(1)Department of Microbiology, Sir Thotub Namgyal Memorial Hospital, Gangtok, Sikkim, India.

Scrub typhus is an acute febrile illness that is known to be endemic in the South East Asian countries and the Western Pacific region. We here report an outbreak in the tiny Himalayan state of Sikkim. Patients with pyrexia of unknown origin were evaluated. They were screened by Weil-Felix test and the rapid immunochromatographic method. Samples that were positive by either Weil-Felix agglutination test or by rapid immunochromatography were confirmed by IgM enzyme-linked immunosorbent assay (ELISA). A total 204 samples were screened. Sixty-three patients were confirmed positive among which 42 were male and 21 were female. Effective management and early administration of antibiotics will help prevent the complications and mortality associated with scrub typhus.

DOI: 10.4103/0255-0857.108729
PMID: 23508434 [PubMed - indexed for MEDLINE]

128. Endocrine. 2013 Oct;44(2):546-8. doi: 10.1007/s12020-013-9947-5. Epub 2013 Apr 7.

Subacute painful thyroiditis accompanied by scrub typhus infection.

Kim Sh(1), Park TS, Baek HS, Jin HY.

Author information:
(1)Division of Endocrinology and Metabolism, Department of Internal Medicine, Research Institute of Clinical Medicine of Chonbuk National University, Chonbuk National University Hospital, Chonbuk National University Medical School, 634-18, Keum-Am Dong, Jeonju, 561-712, South Korea.

Erratum in
   Endocrine. 2013 Oct;44(2):549. Kim, Sunhee [corrected to Kim, Sun hee].

DOI: 10.1007/s12020-013-9947-5
PMID: 23564597 [PubMed - indexed for MEDLINE]

129. Infection. 2012 Aug;40(4):359-65. doi: 10.1007/s15010-011-0239-9. Epub 2012 Jan 12.

The clinical differences between dengue and scrub typhus with acute respiratory failure in southern Taiwan.

Chang HC(1), Lin MC, Liu SF, Su MC, Fang WF, Chen YC, Tseng CC, Lai CH, Huang KT, Wang CC.

Author information:
(1)Division of Pulmonary and Critical Care Medicine, Department of Internal Medicine, Kaohsiung Chang Gung Memorial Hospital and Chang Gung University College of Medicine, No. 123, Dapi Road, Niaosong District, Kaohsiung 83301, Taiwan.

BACKGROUND: For both dengue and scrub typhus, acute respiratory failure (ARF) is a serious complication. The present study was carried out in order to investigate the clinical courses and outcomes of adult dengue and scrub typhus patients with ARF, and to identify the clinical differences between adult dengue and scrub typhus patients with ARF.
METHODS: We conducted a retrospective study of the serologically confirmed adult dengue or scrub typhus patients admitted between 1998 and 2008 at Kaohsiung Chang Gung Memorial Hospital. A total of 980 dengue and 102 scrub typhus adult patients were included in our study.
RESULTS: Eighteen of the 980 adult dengue patients and 8 of the 102 adult scrub typhus patients had ARF. There were significant differences that existed for eschar ($P = 0.001$; dengue 0%; scrub 62.5%), cough ($P = 0.016$; dengue 55.6%; scrub typhus 100%), white blood cell (WBC) count [$P = 0.026$; dengue 7.40 ± 5.74; scrub typhus 11.84 ± 4.95 (×10(3)/μL)], platelet count [$P = 0.008$; dengue 42.2 ± 33.9; scrub typhus 104.1 ± 93.3 (×10(9)/L)], prothrombin time (PT) [$P = 0.007$; dengue 12.82 ± 1.36; scrub typhus 10.74 ± 0.98 (s)], activated partial thromboplastin time (APTT) [$P = 0.002$; dengue 50.81 ± 10.08; scrub typhus 37.44 ± 4.06 (s)], blood urea nitrogen (BUN) [$P < 0.001$; dengue 64.6 ± 43.2; scrub typhus 20.9 ± 9.1 (mg/dL)], creatinine [$P < 0.001$; dengue 3.77 ± 3.37; scrub typhus 1.05 ± 0.37 (mg/dL)], admission day (A-day) [$P = 0.027$; dengue 2.9 ± 1.3; scrub typhus 5.4 ± 2.6 (days)], and ventilator duration [$P = 0.022$; dengue 9.4 ± 14.0; scrub typhus 14.8 ± 10.4 (days)] between both groups.
CONCLUSIONS: This study provides relatively rare data regarding the clinical differences between adult dengue and scrub typhus patients with ARF.

DOI: 10.1007/s15010-011-0239-9
PMID: 22237473 [PubMed - indexed for MEDLINE]

130. J Vector Borne Dis. 2014 Mar;51(1):69-70.

Epididymo-orchitis: a rare manifestation of scrub typhus in a child.

Shanmugapriya V(1), Sangeetha DA, Sampath S, Kasthuri RK.

Author information:
(1)Department of Paediatrics, ESIC Medical College and PGIMSR, Chennai, India.

PMID: 24717207 [PubMed - indexed for MEDLINE]

131. J Am Acad Nurse Pract. 2012 Mar;24(3):160-5. doi:
10.1111/j.1745-7599.2011.00706.x. Epub 2012 Feb 24.

Acute respiratory distress syndrome following scrub typhus: a case report.

Tseng CC(1), Tung HH, Wu SF, Wang TJ.

Author information:
(1)Shin Kong Wu Ho-Su Memorial Hospital, National Taipei University of Nursing
and Health Science, Taipei, Taiwan.

PURPOSE: To discuss a case of scrub typhus complicated with acute respiratory distress syndrome (ARDS) in a 67-year-old female. An overview of the prevalence and pathogenesis of the disorder, clinical diagnosis, and treatment are presented.
DATA SOURCES: Case report, diagnostic evidence, and scientific literature.
CONCLUSIONS: If not properly treated, scrub typhus can cause serious complications, such as ARDS and septicemia. When left untreated, the mortality rate for scrub typhus will be increased. When there is a cause to believe that scrub typhus is present, doxycycline treatment leads to rapid symptom improvement.
IMPLICATIONS FOR PRACTICE: People might not be aware of the possibility of scrub typhus because it had been a more common disease in the past. Nurse practitioners (NPs) should take a detail history and be aware of common clinical manifestations to promote the early recognition and appropriate treatment of scrub typhus, which would prevent severe complications and reduce mortality. A resurgence of the disease could occur at any time among military troops serving in endemic regions.

©2012 The Author(s) Journal compilation ©2012 American Academy of Nurse
Practitioners.

DOI: 10.1111/j.1745-7599.2011.00706.x
PMID: 22404794 [PubMed - indexed for MEDLINE]

132. Eur J Neurol. 2013 Dec;20(12):e129-30. doi: 10.1111/ene.12268.

Bilateral optic neuritis associated with scrub typhus.

Cho HJ(1), Choi JH, Sung SM, Jung DS, Choi KD.

Author information:
(1)Department of Neurology, Pusan National University Hospital, Pusan National
University College of Medicine and Biomedical Research Institute, Busan, Korea.

DOI: 10.1111/ene.12268
PMID: 24433476 [PubMed - indexed for MEDLINE]

133. Acta Radiol. 2012 Jul;53(6):657-61. doi: 10.1258/ar.2012.120012. Epub 2012 May
27.

High-resolution computed tomography findings of swine-origin influenza A (H1N1)
virus (S-OIV) infection: comparison with scrub typhus.

Jo BS(1), Lee IJ, Im HJ, Lee K.

Author information:
(1)Department of Radiology, Hallym University College of Medicine, Seoul, Korea.

BACKGROUND: Swine-origin influenza A (H1N1) virus (S-OIV) infection and scrub typhus, also known as tsutsugamushi disease can manifest as acute respiratory illnesses, particularly during the late fall or early winter, with similar radiographic findings, such as a predominance of ground-glass opacity (GGO).
PURPOSE: To differentiate S-OIV infection from scrub typhus using high-resolution computed tomography (HRCT).
MATERIAL AND METHODS: We retrospectively reviewed the HRCT findings of 14 patients with S-OIV infection and 10 patients with scrub typhus. We assessed the location, cross-sectional distribution, and the presence of a peribronchovascular distribution of GGO and consolidations on HRCT. We also assessed the presence of interlobular septal thickening, bronchial wall thickening, pneumothorax, pneumomediastinum, pleural effusion, and mediastinal or axillary lymph node enlargement.
RESULTS: Scrub typhus was more common than S-OIV in elderly patients (P < 0.001). The monthly incidences of S-OIV and scrub typhus infection reached a peak between October and November. About 86% of S-OIV patients and 80% of scrub typhus patients presented with GGO. About 67% of the GGO lesions in S-OIV had a peribronchovascular distribution, but this was absent in scrub typhus (P = 0.005). Consolidation (93% vs. 10%, P < 0.001) and bronchial wall thickening (43% vs. 0%, P = 0.024) were more frequent in S-OIV infection than scrub typhus. Interlobular septal thickening (90% vs. 36%, P = 0.013) and axillary lymphadenopathy (90% vs. 0%, P < 0.001) were more common in scrub typhus than S-OIV infection.
CONCLUSION: There was considerable overlap in HRCT findings between S-OIV infection and scrub typhus. However, S-OIV showed a distinctive peribronchovascular distribution of GGO lesions. Consolidation and bronchial wall thickening were seen more frequently in S-OIV infection, whereas interlobular septal thickening and axillary lymphadenopathy were more common in scrub typhus. Thus, CT could be helpful for differential diagnosis between S-OIV infection and scrub typhus.

DOI: 10.1258/ar.2012.120012
PMID: 22637640 [PubMed - indexed for MEDLINE]

134. Travel Med Infect Dis. 2013 May-Jun;11(3):197-9. doi: 10.1016/j.tmaid.2012.08.006. Epub 2012 Sep 23.

Imported scrub typhus in The Netherlands.

Vliegenthart-Jongbloed K(1), de Mendonça Melo M, Slobbe L, Beersma MF, van Genderen PJ.

Author information:
(1)Department of Internal Medicine, Havenziekenhuis and Institute for Tropical Diseases, Haringvliet 72, 3011 TG Rotterdam, The Netherlands.

Two cases of travel-acquired scrub typhus imported in the Netherlands are

described. The characteristic eschar was absent in both cases. One case acquired scrub typhus in non-rural surroundings in India, highlighting that scrub typhus must also be considered a (sub) urban zoonosis.

Copyright © 2012 Elsevier Ltd. All rights reserved.

DOI: 10.1016/j.tmaid.2012.08.006
PMID: 23009943 [PubMed - indexed for MEDLINE]

135. Parasit Vectors. 2015 Mar 21;8:172. doi: 10.1186/s13071-015-0784-7.

Detection of Orientia sp. DNA in rodents from Asia, West Africa and Europe.

Cosson JF(1), Galan M(2), Bard E(3), Razzauti M(4), Bernard M(5), Morand S(6,)(7), Brouat C(8), Dalecky A(9,)(10), Bâ K(11), Charbonnel N(12), Vayssier-Taussat M(13).

Author information:
(1)INRA, CBGP, 755 Avenue Campus Agropolis, Montferrier sur Lez, CS30016, 34988, France. cosson@supagro.inra.fr. (2)INRA, CBGP, 755 Avenue Campus Agropolis, Montferrier sur Lez, CS30016, 34988, France. galan@supagro.inra.fr. (3)INRA, EpiA, Clermont-Ferrand, France. ebard@clermont.inra.fr. (4)INRA, CBGP, 755 Avenue Campus Agropolis, Montferrier sur Lez, CS30016, 34988, France. maria.razzauti@supagro.inra.fr. (5)INRA, GABI, Sigenae, Domaine de Vilvert, Jouy en Josas, 78352, France. mabernard@jouy.inra.fr. (6)CNRS-CIRAD, Centre d'Infectiologie Christophe Mérieux du Laos, Vientiane, Lao PDR. serge.morand@cirad.fr. (7)Department of Helminthology, Faculty of Tropical Medicine, Mahidol University, Bangkok, Thailand. serge.morand@cirad.fr. (8)Ird, CBGP, 755 avenue du campus Agropolis, Montferrier sur Lez cedex, CS 30016, 34988, France. carine.brouat@ird.fr. (9)Ird, CBGP, 755 avenue du campus Agropolis, Montferrier sur Lez cedex, CS 30016, 34988, France. ambroise.dalecky@ird.fr. (10)IRD, Aix Marseille Université, LPED (UMR IRD-AMU), Marseille, France. ambroise.dalecky@ird.fr. (11)Ird, CBGP, Campus ISRA/IRD de Bel Air, BP 1386, Dakar, CP 18524, Senegal. khalilou.Ba@ird.fr. (12)INRA, CBGP, 755 Avenue Campus Agropolis, Montferrier sur Lez, CS30016, 34988, France. nathalie.charbonnel@supagro.inra.fr. (13)INRA, Bipar, 23 Av. Général de Gaulle, Maisons-Alfort, France. mvayssier@vet-alfort.fr.

Orientia bacterium is the agent of the scrub typhus, a seriously neglected life-threatening disease in Asia. Here, we report the detection of DNA of Orientia in rodents from Europe and Africa. These findings have important implications for public health. Surveillance outside Asia, where the disease is not expected by sanitary services, needs to be improved.

DOI: 10.1186/s13071-015-0784-7
PMCID: PMC4374543
PMID: 25884521 [PubMed - indexed for MEDLINE]

136. J Assoc Physicians India. 2014 Jan;62(1):58-61.

Triple trouble--macrophage activation syndrome in a case of severe leptospirosis and scrub typhus co-infection.

Diwan AG, Shewale R, Iyer S, Nisal A, Agrawa P.

Macrophage activation syndrome is a potentially life threatening phenomenon characterised by aggressive proliferation of macrophages and T lymphocytes leading to haemophagocytosis of other blood cells and multi organ failure. Here we present a very unusual combination of leptospirosis and scrub typhus infection leading to macrophage activation syndrome. Scrub typhus associated with macrophage activation syndrome has rarely been reported in India. A 40 year old female presented with high grade fever, seizures, bodyache, arthralgia and severe breathlessness. Investigations revealed persistent thrombocytopenia, impaired liver function tests, renal dysfunction, leptospiral IgM ELISA positive and a positive Weil Felix test. There was evidence of haemophagocytosis in bone marrow. Macrophage activation syndrome if left untreated has been associated with rapidly fatal outcome and early treatment can help us save that one precious thing..called life..!

PMID: 25327097 [PubMed - indexed for MEDLINE]

137. ASAIO J. 2012 Sep-Oct;58(5):540-1. doi: 10.1097/MAT.0b013e31825f336f.

Extracorporeal membrane oxygenation for extremely complicated scrub typhus.

Oh WS(1), Yie K.

Author information:
(1)Department of Internal medicine, Kangwon National University and Hospital, Chuncheon, South Korea.

Scrub typhus is a mite-borne disease caused by Orientia tsutsugamushi. Although early diagnosis and appropriate antibiotic therapy improve the prognosis for the majority of patients, life-threatening complications are not uncommon. Here, we present a case of successfully performed veno-veno type extracorporeal membrane oxygenation for scrub typhus-induced complications, including acute respiratory distress syndrome, sudden cardiac arrest, and multiorgan dysfunction. To our knowledge, this is the first case report of successful extracorporeal membrane oxygenation in complicated scrub typhus.

DOI: 10.1097/MAT.0b013e31825f336f
PMID: 22858805 [PubMed - indexed for MEDLINE]

138. BMJ Case Rep. 2014 Nov 12;2014. pii: bcr2014204695. doi: 10.1136/bcr-2014-204695.

Diagnosing scrub typhus: meticulous physical examination is the key.

Rajasekharan C(1), Anu J(1), Neeraj V(1), Parvathy R(2).

Author information:
(1)Department of Internal Medicine, Medical College Hospital, Thiruvananthapuram, Kerala, India. (2)Department of Internal Medicine, MOSC, Medical College, Ernakulam, Kerala, India.

DOI: 10.1136/bcr-2014-204695
PMID: 25391821 [PubMed - indexed for MEDLINE]

139. J Microbiol Immunol Infect. 2012 Jun;45(3):251-4. doi:
10.1016/j.jmii.2011.09.015. Epub 2011 Dec 6.

Successful treatment of septic shock and respiratory failure due to leptospirosis and scrub typhus coinfection with penicillin, levofloxacin, and activated protein C.

Wei YF(1), Chiu CT, Lai YF, Lai CH, Lin HH.

Author information:
(1)Division of Pulmonary Medicine, Department of Internal Medicine, E-Da Hospital/I-Shou University, Kaohsiung County, Taiwan.

Comment in
J Microbiol Immunol Infect. 2015 Feb;48(1):121-2.

Leptospirosis and scrub typhus are common zoonoses and coinfection with both diseases has been reported sporadically, particularly in tropical and subtropical areas. A 53-year-old male presented with acute hypoxemic respiratory failure and septic shock due to leptospirosis and scrub typhus coinfection confirmed by serological assessments. Antibiotics, including intravenous penicillin and levofloxacin, were administered and human recombinant activated protein C was added because of a high risk of death due to septic shock with multiple organ failure. The patient's hemodynamics and hypoxemia substantially improved 4 days later and he had a complete recovery from the disease after 10 days of hospitalization. Coinfection of leptospirosis and scrub typhus may easily go unrecognized by physicians in febrile travelers or patients in the region where both diseases are endemic. In severe and critical cases of leptospirosis, scrub typhus, or coinfection with both, the use of APC in addition to appropriate antibiotic treatment and standard critical care might provide a greater chance for survival and a favorable outcome.

Copyright © 2012. Published by Elsevier B.V.

DOI: 10.1016/j.jmii.2011.09.015
PMID: 22153761 [PubMed - indexed for MEDLINE]

140. Jpn J Infect Dis. 2014;67(2):122-6.

A case series of possibly recrudescent Orientia tsutsugamushi infection presenting as pneumonia.

Im JH(1), Baek JH, Lee JS, Chung MH, Lee SM, Kang JS.

Author information:
(1)Department of Internal Medicine, Inha University School of Medicine.

Orientia tsutsugamushi remains latent in humans after scrub typhus. Pneumonia

occurs as a complication of scrub typhus in the late-phase disease. However, pneumonia may also occur as a presenting manifestation of O. tsutsugamushi infection. We reviewed the cases of 3 patients with atypical pneumonia who presented at our hospital and were later confirmed to have O. tsutsugamushi infection by serology, nested polymerase chain reaction (PCR), and cell culture. All patients were young adults with no history of scrub typhus, and none claimed to have recently been exposed to areas where scrub typhus is endemic. Two cases occurred in non-outbreak seasons. Furthermore, eschar was not observed. Pneumonia was documented within 4 days after fever onset. The immunoglobulin (Ig) G antibody titers against O. tsutsugamushi were higher than the IgM titers, although the serologic test results were less helpful in the diagnosis. Nested PCR and cell culture of blood specimens confirmed the diagnosis of O. tsutsugamushi infection. These findings suggest that pneumonia can occur as a result of recrudescence of latent O. tsutsugamushi infection.

PMID: 24647257 [PubMed - indexed for MEDLINE]

141. J Pediatr Hematol Oncol. 2012 Oct;34(7):531-3.

Scrub typhus-associated severe hemophagocytic lymphohistiocytosis with encephalomyelitis leading to permanent sequelae: a case report and review of the literature.

Han DK(1), Baek HJ, Shin MG, Kim JW, Kook H, Hwang TJ.

Author information:
(1)Department of Pediatrics, Chonnam National University Hwasun Hospital, Chonnam National University Medical School, Gwangju, Chollanamdo, Korea.

Hemophagocytic lymphohistiocytosis (HLH) is a rare but potentially fatal disorder. There have been a few reports on HLH secondary to scrub typhus in adults. Here, we describe the case of a 9-year-old Korean girl who presented with the typical findings of HLH. Despite adequate antirickettsial and HLH treatment, the neurological impairment worsened and remained. This is the first case report of severe neurological impairment resulting from the very rare association of HLH with scrub typhus. Therefore, in endemic areas, a high index of suspicion for scrub typhus is warranted in patients presenting with HLH.

DOI: 10.1097/MPH.0b013e318257a442
PMID: 22627574 [PubMed - indexed for MEDLINE]

142. PLoS One. 2013 May 14;8(5):e63668. doi: 10.1371/journal.pone.0063668. Print 2013.

A case-control study of risk factors associated with scrub typhus infection in Beijing, China.

Lyu Y(1), Tian L, Zhang L, Dou X, Wang X, Li W, Zhang X, Sun Y, Guan Z, Li X, Wang Q.

Author information:
(1)Institute for Infectious Disease and Endemic Disease Prevention and Control,

Beijing Center for Disease Prevention and Control, Beijing, China.

To investigate the risk factors of scrub typhus infection in Beijing, China, a case-control study was carried out. Cases (n = 56) were defined as persons who were diagnosed by PCR and serological method within three years. Three neighborhood control subjects were selected by matching for age and occupation. Living at the edge of the village, living in the houses near grassland, vegetable field or ditch, house yard without cement floor, piling weeds in the house or yard, all of these were risk factors for scrub typhus infection. Working in vegetable fields and hilly areas, and harvesting in autumn posed the highest risks, with odds ratios (ORs) and 95% confidence intervals (CIs) of 3.7 (1.1-11.9), 8.2 (1.4-49.5), and 17.2 (5.1-57.9), respectively. These results would be useful for the establishment of a detail control strategy for scrub typhus infection in Beijing, China.

DOI: 10.1371/journal.pone.0063668
PMCID: PMC3653850
PMID: 23691083 [PubMed - indexed for MEDLINE]

143. PLoS Negl Trop Dis. 2013 Aug 29;7(8):e2163. doi: 10.1371/journal.pntd.0002163. eCollection 2013.

Concurrent Infection with murine typhus and scrub typhus in southern Laos--the mixed and the unmixed.

Phommasone K(1), Paris DH, Anantatat T, Castonguay-Vanier J, Keomany S, Souvannasing P, Blacksell SD, Mayxay M, Newton PN.

Author information:
(1)Lao-Oxford-Mahosot Hospital-Wellcome Trust Research Unit (LOMWRU), Microbiology Laboratory, Mahosot Hospital, Vientiane, Lao PDR.

DOI: 10.1371/journal.pntd.0002163
PMCID: PMC3757080
PMID: 24009783 [PubMed - indexed for MEDLINE]

144. Kaohsiung J Med Sci. 2011 Dec;27(12):573-6. doi: 10.1016/j.kjms.2011.10.003. Epub 2011 Nov 25.

Bilateral simultaneous facial palsy following scrub typhus meningitis: a case report and literature review.

Lin WR(1), Chen TC, Lin CY, Lu PL, Chen YH.

Author information:
(1)Division of Infectious Diseases, Department of Internal Medicine, Kaohsiung Medical University Hospital, Kaohsiung Medical University, Kaohsiung, Taiwan.

Scrub typhus is widely distributed across the Asia-Pacific region, Taiwan included. The clinical manifestations and complications of scrub typhus vary and the illness ranges in severity from mild to fatal. The etiology of facial nerve palsy varies and infectious agents have been associated with this condition.

Rickettsiae species have, however, rarely been reported as the causative agents.
We report the case of a 49-year-old man who had fever, malaise, headache, oligouria and tea-colored urine. Bilateral pneumonitis, acute renal failure, acalculous cholecystitis and aseptic meningitis were diagnosed after a series of examinations. The patient recovered after doxycycline treatment but he developed bilateral facial palsy during the convalescent phase, which improved after the administration of a steroid. The diagnosis of infection with Orientia tsutsugamushi was confirmed by the Taiwan Center of Disease Control and the tests for Leptospira, Rickettsia typhi and Coxiella burnetii were all negative. This case indicates that scrub typhus needs to be included in the differential diagnoses of cases of bilateral and simultaneous facial nerve palsy, particularly in areas where the disease is endemic.

Copyright © 2011. Published by Elsevier B.V.

DOI: 10.1016/j.kjms.2011.10.003
PMID: 22208541 [PubMed - indexed for MEDLINE]

145. Osong Public Health Res Perspect. 2013 Feb;4(1):4-15. doi:
10.1016/j.phrp.2012.12.007.

Spatial distribution analysis of scrub typhus in Korea.

Jin HS(1), Chu C, Han DY.

Author information:
(1)Department of Mathematics, Chonnam National University, Gwangju, Korea .

OBJECTIVE: This study analyzes the spatial distribution of scrub typhus in Korea.
METHODS: A spatial distribution of Orientia tsutsugamushi occurrence using a geographic information system (GIS) is presented, and analyzed by means of spatial clustering and correlations.
RESULTS: The provinces of Gangwon-do and Gyeongsangbuk-do show a low incidence throughout the year. Some districts have almost identical environmental conditions of scrub typhus incidence. The land use change of districts does not directly affect the incidence rate.
CONCLUSION: GIS analysis shows the spatial characteristics of scrub typhus. This research can be used to construct a spatial-temporal model to understand the epidemic tsutsugamushi.

DOI: 10.1016/j.phrp.2012.12.007
PMCID: PMC3747683
PMID: 24159523 [PubMed]

146. Ren Fail. 2013;35(10):1338-43. doi: 10.3109/0886022X.2013.828257. Epub 2013 Aug
19.

Scrub typhus associated acute kidney injury--a study from a tertiary care hospital from western Himalayan State of India.

Vikrant S(1), Dheer SK, Parashar A, Gupta D, Thakur S, Sharma A, Kaushal SS, Kanga A.

Author information:
(1)Department of Nephrology .

OBJECTIVE: To report the clinical profile of acute kidney injury (AKI) associated with scrub typhus.
METHODS: Retrospective study of hospitalized patients of acute febrile illness who were diagnosed scrub typhus and had AKI.
RESULTS: 174 (35%) patients (75.9% female), mean age (41.4 ± 15.9 years) were studied. The laboratory abnormalities were: anemia (63.2%), leukocytosis (44.3%), thrombocytopenia (61.5%), hyponatremia (35.6%), hypernatremia (2.9%), and hypokalemia (12.1%), hyperkalemia (11.5%), hypoalbuminemia (56.9%), hepatic dysfunction (70%) and metabolic acidosis (28.7%). The complications of hypotension (5.7%), septic shock (3.4%), pneumonia (10.9%), acute respiratory distress syndrome (ARDS) (11.5%), meningoencephalitis (6.9%), encephalopathy (5.2%), gastrointestinal bleed (1.3%), myocarditis (3.4%), disseminated intravascular coagulation (2.9%) and multi organ failure (MOF) (10.3%) developed during course of hospitalization in these patients. Twenty-five (14.4%) patients required intensive care support (ICU) support and seven (4%) patients were dialyzed. 146 (83.9%) patients survived. Twenty-eight (16.1%) patients died. There was a significant difference in the age, various hematological & biochemical abnormalities, complications and need for ICU support in the non-survival group as compared the survival group.
CONCLUSION: This study shows that AKI in scrub typhus is common and a severe disease. Age, a shorter hospital stay, severities of leukocytosis, thrombocytopenia, azotemia, hypoalbuminemia, hepatic dysfunction and the complications of ARDS, encephalopathy, MOF and need for ICU support are the factors associated with mortality.

DOI: 10.3109/0886022X.2013.828257
PMID: 23952649 [PubMed - indexed for MEDLINE]

147. Zhonghua Yu Fang Yi Xue Za Zhi. 2012 Apr;46(4):338-42.

[Analysis of epidemic features of scrub typhus between year 2006 and 2010 in Shandong province, China].

[Article in Chinese]

Ding L(1), Li Z, Wang XJ, Ding SJ, Zhang M, Zhao ZT.

Author information:
(1)Department of Epidemiology, School of Public Health, Shandong University, Jinan 250012, China.

OBJECTIVE: To explore the epidemic features of scrub typhus between year 2006 and 2010 in Shandong Province.
METHODS: Based on the data collected through Diseases Reporting Information System between year 2006 and 2010 in Shandong province, 1291 cases of scrub typhus were selected. The study described the population distribution features of the scrub typhus patients, and explored the temporal and spatial distribution features of the disease by applying the methods of spatial thematic mapping, inverse distance weighted, spatial autocorrelation analysis, spatial clustering

analysis, temporal clustering analysis and spatial variation analysis in temporal trends based on Geographic Information software (ArcGIS 9.3) and Spatial Clustering Software (SatScan 7.0).
RESULTS: The onset age of the 1291 patients ranged between 1 and 92 years old.639 out of 1291 patients were over 55 years old, accounting for 49.5%.640 patients were male and the other 651 patients were female, occupying 49.6% and 50.4% respectively. The gender ratio was 1:1.02. Patients were found in farmers, workers, students and preschool children. However, most of the cases were farmers, up to 84.8% (1095/1291). Global Moran's I index was 0.324 (P < 0.01). The local Moran's I index in 8 locations were proved to have statistical significance (P < 0.01); all of which were H-H clustering areas. Gangcheng (38 cases), Laicheng (154 cases), Xintai (160 cases) and Donggang (105 cases) were important locations, whose local Moran's I index were 2.111, 1.642, 1.277 and 0.775 respectively. The clustering period of scrub typhus in respective year were as follows: 2006.09.23 - 2006.11.20 (202 cases), 2007.10.02 - 2007.11.11 (197 cases), 2008.09.30 - 2008.11.07 (302 cases), 2009.09.25 - 2009.11.10 (204 cases), and 2010.10.05 - 2010.11.13 (226 cases), whose RR values were separately 45.55, 34.60, 50.64, 53.09 and 79.84 (P < 0.01). Two spatial clustering area were found in the study, one was the area centered Taian and Xintai with radiation radius at 58.28 km (542 cases) and the other one was the area centered Rizhao and Donggang with radiation radius at 22.68 km (134 cases), whose RR values were 4.52 and 3.96 (P < 0.01). The spatial features of the two clustering areas were inland low hills area and coastal hills area. The highest annual growth rate of the disease was 45.04%, found in the area centered Linyi and Mengyin counties, with the radiation radius at 45.82 km. The RR value was 3.68 (P < 0.01).
CONCLUSION: The majority of the scrub typhus patients were middle-aged and elderly farmers. The epidemic peak was between the last 10 days of September and the first 10 days of November. A positive spatial correlation of the disease was found; and most cases clustered in inland low hills area and costal hills area; especially the area around Linyi and Mengyin, with the highest annual growth rates of the disease.

PMID: 22800633 [PubMed - indexed for MEDLINE]

148. J Vector Borne Dis. 2014 Mar;51(1):71-2.

Rare concurrent infection with scrub typhus, dengue and malaria in a young female.

Kumar S(1), Kumar PS, Kaur G, Bhalla A, Sharma N, Varma S.

Author information:
(1)Department of Internal Medicine, Postgraduate Institute of Medical Education and Research, Chandigarh, India.

PMID: 24717208 [PubMed - indexed for MEDLINE]

149. Indian J Dermatol Venereol Leprol. 2014 Mar-Apr;80(2):165-6. doi: 10.4103/0378-6323.129406.

Scrub typhus in a child: looking behind the ear and beyond.

Venkatesh C(1), Arulraj R, Soundararajan P, Srinivasan S.

Author information:
(1)Department of Pediatrics, Mahatma Gandhi Medical College and Research Institute, Pillaiyarkuppam, Puducherry, India.

DOI: 10.4103/0378-6323.129406
PMID: 24685862 [PubMed - indexed for MEDLINE]

150. J Infect. 2016 Sep 1. pii: S0163-4453(16)30218-3. doi: 10.1016/j.jinf.2016.08.014. [Epub ahead of print]

Acute encephalitis syndrome in Gorakhpur, Uttar Pradesh, India - Role of scrub typhus.

Murhekar MV(1), Mittal M(2), Prakash JA(3), Pillai VM(4), Mittal M(5), Girish Kumar CP(6), Shinde S(6), Ranjan P(6), Oak C(6), Gupta N(7), Mehendale S(8), Arora R(7), Gupte M(7).

Author information:
(1)Dept of Epidemiology, National Institute of Epidemiology, Indian Council of Medical Research, Chennai, Tamil Nadu, India. Electronic address: mmurhekar@gmail.com. (2)Dept of Pediatrics, Baba Raghav Das Medical College, Gorakhpur, Uttar Pradesh, India. (3)Dept of Clinical Microbiology, Christian Medical College, Vellore, Tamil Nadu, India. (4)Dept of Medicine, Jawaharlal Institute of Postgraduate Medical Education & Research, Puducherry, India. (5)Dept of Medicine, Baba Raghav Das Medical College, Gorakhpur, Uttar Pradesh, India. (6)Dept of Epidemiology, National Institute of Epidemiology, Indian Council of Medical Research, Chennai, Tamil Nadu, India. (7)Dept of Epidemiology and Communicable Diseases, Indian Council of Medical Research, New Delhi, India. (8)National Institute of Epidemiology, Indian Council of Medical Research, Chennai, Tamil Nadu, India.

DOI: 10.1016/j.jinf.2016.08.014
PMID: 27592263 [PubMed - as supplied by publisher]

151. Vector Borne Zoonotic Dis. 2011 Mar;11(3):209-14. doi: 10.1089/vbz.2009.0180.

Detection of Orientia tsutsugamushi, the causative agent of scrub typhus, in a novel mite species, Eushoengastia koreaensis, in Korea.

Lee HI(1), Shim SK, Song BG, Choi EN, Hwang KJ, Park MY, Park C, Shin EH.

Author information:
(1)Division of Medical Entomology, Korea Centers for Disease Control and Prevention, Seoul, Korea.

To identify potential vector species of scrub typhus in the Republic of Korea (ROK), chigger mites were harvested from wild rodents captured at nine localities in October 2005. The bodies of the chigger mites were individually punctured with

a fine pin, squeezed out internal contents, and examined for Orientia tsutsugamushi DNA by nested polymerase chain reaction. The exoskeleton of associated chiggers was mounted on glass slides with polyvinylalcohol (PVA) medium for identification. Among 830 individuals belonging to 4 genera and 14 species, O. tsutsugamushi was detected from 22 chiggers of six species, with an overall infection rate of 2.7%. The infection rate was highest for Leptotrombidium palpale (5.3%), followed by Neotrombicula japonica (4.3%), Leptotrombidium scutellare (3.7%), Leptotrombidium orientale (3.6%), Eushoengastia koreaensis (1.9%), and Leptotrombidium pallidum (1.5%). This study first reported O. tsutsugamushi infection from N. japonica and E. koreaensis larvae in the ROK. The population densities of L. pallidum (33.4 chiggers/rodent), historically confirmed as a primary vector of scrub typhus in the ROK, were high, whereas its infection rate was relatively low (1.5%). However, E. koreaensis was only collected from 154 individuals at seven collection sites and its infection rate was demonstrated relatively high (mean 1.9%). Additional studies are needed to determine the role of vector species in the epidemiology of scrub typhus.

DOI: 10.1089/vbz.2009.0180
PMID: 21443412 [PubMed - indexed for MEDLINE]

152. J Clin Neurosci. 2013 Jan;20(1):182-3. doi: 10.1016/j.jocn.2012.01.047. Epub 2012 Sep 23.

Scrub typhus associated with transient parkinsonism and myoclonus.

Chiou YH(1), Yang CJ, Lai TH.

Author information:
(1)Neurology Section, Department of Internal Medicine, Far Eastern Memorial Hospital, 21 Nan-Ya S. Road, Sec. 2, Pan-Chiao, New Taipei 220, Taiwan. hippus.chiou@msa.hinet.net

Parkinsonism with myoclonus is rarely associated with infectious disease in adults. We present a 55-year-old man experiencing acute onset bilateral limb tremor, rigidity, and myoclonus with small-stepped gait, and skin rash involving the trunk and limbs, after a fever. Serum was positive for anti-Orientia tsutsugamushi immunoglobulin M antibody. Brain MRI revealed no abnormalities. The fever improved with oral doxycycline, and the parkinsonism and myoclonus improved with amantadine and clonazepam. This is a rare case of parkinsonism with myolonus associated with scrub typhus infection.

Copyright © 2012 Elsevier Ltd. All rights reserved.

DOI: 10.1016/j.jocn.2012.01.047
PMID: 23010430 [PubMed - indexed for MEDLINE]

153. Ecol Appl. 2012 Sep;22(6):1803-16.

Cascading effect of economic globalization on human risks of scrub typhus and tick-borne rickettsial diseases.

Kuo CC(1), Huang JL, Shu PY, Lee PL, Kelt DA, Wang HC.

Author information:
(1)Department of Wildlife, Fish, and Conservation Biology, University of California, One Shields Avenue, Davis, California 95616, USA.

The increase in global travel and trade has facilitated the dissemination of disease vectors. Globalization can also indirectly affect vector-borne diseases through the liberalization of cross-border trade, which has far-reaching, worldwide effects on agricultural practices and may in turn influence vectors through the modification of the ecological landscape. While the cascading effect of economic globalization on vector-borne diseases, sometimes acting synergistically with regional agricultural policy, could be substantial and have significant economic, agricultural, and public health implications, research into this remains very limited. We evaluated how abandonment of rice paddies in Taiwan after joining the World Trade Organization, along with periodic plowing, an agricultural policy to reduce farm pests in abandoned fields can unexpectedly influence risks to diseases transmitted by ticks and chiggers (larval trombiculid mites), which we collected from their small-mammal hosts. Sampling was limited to abandoned (fallow) and plowed fields due to the challenge of trapping small mammals in flooded rice paddies. Striped field mice (Apodemus agrarius) are the main hosts for both vectors. They harbored six times more ticks and three times more chiggers in fallow than in plowed plots. The proportion of ticks infected with Rickettsia spp. (etiologic agent of spotted fever) was three times higher in fallow plots, while that of Orientia tsutsugamushi (scrub typhus) in chiggers was similar in both treatments. Fallow plots had more ground cover and higher vegetation than plowed ones. Moreover, ticks and chiggers in both field types were dominated by species known to infest humans. Because ticks and chiggers should exhibit very low survival in flooded rice paddies, we propose that farm abandonment in Taiwan, driven by globalization, may have inadvertently led to increased risks of spotted fever and scrub typhus. However, periodic plowing can unintentionally mitigate vector burdens. Economic globalization can have unexpected consequences on disease risk through modification of the agricultural landscape, but the outcome may also be influenced by agricultural policies, calling for further research on vector-borne diseases and their control from broader perspectives.

PMID: 23092017 [PubMed - indexed for MEDLINE]

154. Am J Trop Med Hyg. 2014 Sep;91(3):580-3. doi: 10.4269/ajtmh.13-0570. Epub 2014 Aug 4.

Serosurveillance of Orientia tsutsugamushi and Rickettsia typhi in Bangladesh.

Maude RR(1), Maude RJ(2), Ghose A(2), Amin MR(2), Islam MB(2), Ali M(2), Bari MS(2), Majumder MI(2), Tanganuchitcharnchai A(2), Dondorp AM(2), Paris DH(2), Bailey RL(2), Faiz MA(2), Blacksell SD(2), Day NP(2).

Author information:
(1)Mahidol-Oxford Tropical Medicine Research Unit, Faculty of Tropical Medicine, Mahidol University, Bangkok, Thailand; Centre for Tropical Medicine, Nuffield Department of Clinical Medicine, Churchill Hospital, Oxford, United Kingdom;

Chittagong Medical College, Chittagong, Bangladesh; Dhaka Medical College, Dhaka, Bangladesh; Comilla Medical College, Comilla, Bangladesh; Shaheed Ziaur Rahman Medical College, Bogra, Bangladesh; MAG Osmani Medical College, Sylhet, Bangladesh; Sir Salimullah Medical College, Dhaka, Bangladesh; London School of Hygiene and Tropical Medicine, London, United Kingdom rapeephan.maude@gmail.com.
(2)Mahidol-Oxford Tropical Medicine Research Unit, Faculty of Tropical Medicine, Mahidol University, Bangkok, Thailand; Centre for Tropical Medicine, Nuffield Department of Clinical Medicine, Churchill Hospital, Oxford, United Kingdom; Chittagong Medical College, Chittagong, Bangladesh; Dhaka Medical College, Dhaka, Bangladesh; Comilla Medical College, Comilla, Bangladesh; Shaheed Ziaur Rahman Medical College, Bogra, Bangladesh; MAG Osmani Medical College, Sylhet, Bangladesh; Sir Salimullah Medical College, Dhaka, Bangladesh; London School of Hygiene and Tropical Medicine, London, United Kingdom.

Scrub and murine typhus infections are under-diagnosed causes of febrile illness across the tropics, and it is not known how common they are in Bangladesh. We conducted a prospective seroepidemiologic survey across six major teaching hospitals in Bangladesh by using an IgM enzyme-linked immunosorbent assay. Results indicated recent exposure (287 of 1,209, 23.7% seropositive for Orientia tsutsugamushi and 805 of 1,209, 66.6% seropositive for Rickettsia typhi). Seropositive rates were different in each region. However, there was no geographic clustering of seropositive results for both organisms. There was no difference between those from rural or urban areas. Rickettsia typhi seroreactivity was positively correlated with age. Scrub typhus and murine typhus should be considered as possible causes of infection in Bangladesh.

© The American Society of Tropical Medicine and Hygiene.

DOI: 10.4269/ajtmh.13-0570
PMCID: PMC4155564
PMID: 25092819 [PubMed - indexed for MEDLINE]

155. Vector Borne Zoonotic Dis. 2013 Jun;13(6):367-75. doi: 10.1089/vbz.2012.1112. Epub 2013 Apr 16.

Diversity of the 47-kD HtrA nucleic acid and translated amino acid sequences from 17 recent human isolates of Orientia.

Jiang J(1), Paris DH, Blacksell SD, Aukkanit N, Newton PN, Phetsouvanh R, Izzard L, Stenos J, Graves SR, Day NP, Richards AL.

Author information:
(1)Naval Medical Research Center , Silver Spring, Maryland, USA.

Orientia tsutsugamushi, the etiologic agent of potentially fatal scrub typhus, is characterized by a high antigenic diversity, which complicates the development of a broadly protective vaccine. Efficacy studies in murine and nonhuman primate models demonstrated the DNA vaccine candidate pKarp47, based upon the O. tsutsugamushi Karp 47-kD HtrA protein gene, to be a successful immunoprophylactic against scrub typhus. To characterize 47-kD HtrA protein diversity among human isolates of Orientia, we sequenced the full open reading frame (ORF) of the 47-kD HtrA gene and analyzed the translated amino acid sequences of 17 patient isolates from Thailand (n=13), Laos (n=2), Australia (n=1), and the United Arab Emirates

(UAE) (n=1) and 9 reference strains: Karp (New Guinea), Kato (Japan), Ikeda (Japan), Gilliam (Burma), Boryong (Korea), TA763, TH1811 and TH1817 (Thailand), and MAK243 (China). The percentage identity (similarity) of translated amino acid sequences between 16 new isolates and 9 reference strains of O. tsutsugamushi ranged from 96.4% to 100% (97.4% to 100%). However, inclusion of the recently identified Orientia chuto sp. nov. reduced identity (similarity) values to 82.2% to 83.3% (90.4% to 91.4%). These results demonstrate the diversity of Orientia 47-kD HtrA among isolates encountered by humans and therefore provide support for the necessity of developing a broadly protective scrub typhus vaccine that takes this diversity into account.

DOI: 10.1089/vbz.2012.1112
PMCID: PMC3669598
PMID: 23590326 [PubMed - indexed for MEDLINE]

156. Acta Trop. 2011 Oct-Nov;120(1-2):52-8. doi: 10.1016/j.actatropica.2011.05.018. Epub 2011 Jun 14.

Spatial analysis of scrub typhus infection and its association with environmental and socioeconomic factors in Taiwan.

Kuo CC(1), Huang JL, Ko CY, Lee PF, Wang HC.

Author information:
(1)Research and Diagnostic Center, Centers for Disease Control, Department of Health, Taipei, Taiwan, ROC.

We analyzed the spatial distribution of human cases of scrub typhus on the main island of Taiwan from 2003 to 2008 and implemented an island-wide survey of scrub typhus vectors (trombiculid chiggers) in 2007 and 2008. The standardized incidence rate 'SIR' incorporating inter-district variations in population, gender and age was correlated with environmental and socioeconomic variables. Higher incidence and SIR rates were clustered in the less developed, mountainous regions of central and eastern Taiwan. Higher SIRs were also associated with a higher proportion of dry-field farmers in the population, a higher normalized difference vegetation index (NDVI) and lower mean annual temperature, but was not associated with rainfall. Small mammal hosts in high-SIR districts harbored more chiggers and had higher rates of seropositivity against Orientia tsutsugamushi Hyashi, the etiologic agent of scrub typhus, compared to low-SIR districts. The concurrence of a higher proportion of dry-field farmers and higher NDVI has likely led to the clustering of scrub typhus in the mountainous regions of Taiwan. Further individual-level study of the risk factors associated with scrub typhus, and a better understanding of the effect of environmental factors on chigger abundance, should help to prevent scrub typhus in Taiwan.

Copyright © 2011 Elsevier B.V. All rights reserved.

DOI: 10.1016/j.actatropica.2011.05.018
PMID: 21703220 [PubMed - indexed for MEDLINE]

157. Neurol India. 2014 Jan-Feb;62(1):82-3. doi: 10.4103/0028-3886.128340.

A rare case of Guillain-Barre syndrome following scrub typhus.

Sawale VM(1), Upreti S, Singh TS, Singh NB, Singh TB.

Author information:
(1)Department of Medicine, Regional Institute of Medical Sciences, Imphal, Manipur, India.

DOI: 10.4103/0028-3886.128340
PMID: 24608469 [PubMed - indexed for MEDLINE]

158. Ann Emerg Med. 2013 Mar;61(3):370-5. doi: 10.1016/j.annemergmed.2012.07.111.

Young man with fever and cough. Scrub typhus pneumonitis.

Hung Y(1), Lin TY.

Author information:
(1)Division of Infectious Diseases and Tropical Medicine, Department of Internal Medicine, Tri-Service General Hospital, National Defense Medical Center, Taipei, Taiwan.

DOI: 10.1016/j.annemergmed.2012.07.111
PMID: 23433021 [PubMed - indexed for MEDLINE]

159. Zhonghua Liu Xing Bing Xue Za Zhi. 2013 Sep;34(9):946-7.

[Epidemiological investigation on a scrub typhus outbreak in a village from Guangdong province, China].

[Article in Chinese]

Liu J, Chen BH, Wu D, Liu WH, Yao LJ, Mao XT, Xiao LH, Zhong HJ, Peng ZQ.

PMID: 24380098 [PubMed - indexed for MEDLINE]

160. Emerg Infect Dis. 2013 Jul;19(7):1135-7. doi: 10.3201/eid1907.120984.

Genetic variants of Orientia tsutsugamushi in domestic rodents, northern China.

Zhang M, Zhao ZT, Wang XJ, Li Z, Ding L, Ding SJ, Yang HL.

We screened Orientia tsutsugamushi from 385 domestic rodents and 19 humans with scrub typhus in rural Tai'an District, Shandong Province, a new scrub typhus epidemic area in northern China. Sequence analysis identified 7 genotypes in the rodents, of which 2 were also identified in the humans.

DOI: 10.3201/eid1907.120984
PMCID: PMC3713967
PMID: 23764295 [PubMed - indexed for MEDLINE]

161. Trans R Soc Trop Med Hyg. 2012 Jan;106(1):48-53. doi:
10.1016/j.trstmh.2011.09.005. Epub 2011 Oct 26.

Chest radiographic presentation in patients with scrub typhus.

Chen HC(1), Chang HC, Chang YC, Liu SF, Su MC, Huang KT, Lin MC, Wang CC.

Author information:
(1)Division of Pulmonary and Critical Care Medicine, Department of Internal Medicine, Chang Gung Memorial Hospital-Kaohsiung Medical Center, Chang Gung University College of Medicine, Kaohsiung, Taiwan.

We conducted a retrospective study of the adult patients (age ≥18 years) with serologically confirmed scrub typhus admitted between January 1998 and December 2009 at Kaohsiung Chang Gung Memorial Hospital. There were 63 adult scrub typhus patients with chest radiographic examinations. A total of 147 chest radiographs were obtained and reviewed. The most frequent abnormal findings in this study were parenchymal infiltration with bilateral and lower lung predilection. The distribution of abnormal chest radiographs was observed more than 50% during the first week. Furthermore, the progressive change was frequently observed during the first week. There is a significant correlation among laboratory findings, clinical course, and outcome. Chest radiography may be a complementary tool to evaluate the clinical course of scrub typhus and chest radiographic examinations should be taken during the first week after the onset of illness.

Copyright © 2011 Royal Society of Tropical Medicine and Hygiene. Published by Elsevier Ltd. All rights reserved.

DOI: 10.1016/j.trstmh.2011.09.005
PMID: 22033144 [PubMed - indexed for MEDLINE]

162. J Glob Infect Dis. 2014 Jan;6(1):28-30. doi: 10.4103/0974-777X.127947.

Pancreatitis in scrub typhus.

Bhatt A(1), Menon AA(1), Bhat R(1), Gurusiddana SG(1).

Author information:
(1)Department of General Medicine, Kasturba Medical College, Manipal, Karnataka, India.

Scrub typhus is a rickettsial infection prevalent in most parts of India. Acute pancreatitis with pseudocyst formation is a rare complication of this condition. This paper reports acute renal failure, pancreatitis and pseudocyst formation in a 48-year-old female with scrub typhus. Ultrasonography of the abdomen revealed a bulky pancreas with fluid seen along the body of the pancreas in the lesser sac. The infection was successfully treated with doxycycline and supportive treatment. Pancreatitis was managed conservatively. This case report highlights the importance of identifying and managing uncommon complications of a common tropical disease for optimum outcome.

DOI: 10.4103/0974-777X.127947
PMCID: PMC3982352
PMID: 24741228 [PubMed]

163. J Vector Borne Dis. 2016 Apr-Jun;53(2):185-187.

Molecular confirmation of scrub typhus infection and characterization of Orientia tsutsugamushi genotype from Karnataka, India.

Koraluru M(1), Bairy I(2), Singh R(3), Varma M(3), Stenos J(4).

Author information:
(1)Department of Microbiology, Kasturba Medical College. Manipal, Karnataka, India. (2)Department of Microbiology, Melaka Manipal Medical College, Manipal, Karnataka, India. (3)Department of Medicine, Kasturba Medical College, Manipal University, Manipal, Karnataka, India. (4)Australian Rickettsial Reference Laboratory, Geelong, Victoria, Australia.

PMID: 27353590 [PubMed - as supplied by publisher]

164. Risk Manag Healthc Policy. 2014 Feb 18;7:29-34. doi: 10.2147/RMHP.S56974. eCollection 2014.

Validation of a clinical risk-scoring algorithm for severe scrub typhus.

Sriwongpan P(1), Patumanond J(2), Krittigamas P(3), Tantipong H(4), Tawichasri C(5), Namwongprom S(6).

Author information:
(1)Clinical Epidemiology Program, Faculty of Medicine, Chiang Mai University, Chiang Mai ; Department of Social Medicine, Chiangrai Prachanukroh Hospital, Chiang Rai. (2)Clinical Epidemiology Program, Faculty of Medicine, Thammasat University, Bangkok. (3)Department of General Pediatrics, Nakornping Hospital, Chiang Mai. (4)Department of Medicine, Chonburi Hospital, Chonburi. (5)Clinical Epidemiology Society at Chiang Mai, Chiang Mai. (6)Clinical Epidemiology Program, Faculty of Medicine, Chiang Mai University, Chiang Mai ; Department of Radiology, Faculty of Medicine, Chiang Mai University, Chiang Mai, Thailand.

OBJECTIVE: The aim of the study reported here was to validate the risk-scoring algorithm for prognostication of scrub typhus severity.
METHODS: The risk-scoring algorithm for prognostication of scrub typhus severity developed earlier from two general hospitals in Thailand was validated using an independent dataset of scrub typhus patients in one of the hospitals from a few years later. The predictive performances of the two datasets were compared by analysis of the area under the receiver-operating characteristic curve (AuROC). Classification of patients into non-severe, severe, and fatal cases was also compared.
RESULTS: The proportions of non-severe, severe, and fatal patients by operational definition were similar between the development and validation datasets. Patient, clinical, and laboratory profiles were also similar. Scores were similar in both datasets, both in terms of discriminating non-severe from severe and fatal

patients (AuROC =88.74% versus 91.48%, P=0.324), and in discriminating fatal from severe and non-severe patients (AuROC =88.66% versus 91.22%, P=0.407). Over- and under-estimations were similar and were clinically acceptable.
CONCLUSION: The previously developed risk-scoring algorithm for prognostication of scrub typhus severity performed similarly with the validation data and the first dataset. The scoring algorithm may help in the prognostication of patients according to their severity in routine clinical practice. Clinicians may use this scoring system to help make decisions about more intensive investigations and appropriate treatments.

DOI: 10.2147/RMHP.S56974
PMCID: PMC3933538
PMID: 24600256 [PubMed]

165. J Infect Public Health. 2012 Mar;5(1):82-8. doi: 10.1016/j.jiph.2011.11.001. Epub 2011 Dec 24.

Scrub typhus in children at a tertiary hospital in southern India: clinical profile and complications.

Kumar M(1), Krishnamurthy S, Delhikumar CG, Narayanan P, Biswal N, Srinivasan S.

Author information:
(1)Department of Pediatrics, Jawaharlal Institute of Postgraduate Medical Education and Research, Pondicherry, India.

OBJECTIVE: To study the clinical profile of and complications in children with scrub typhus.
DESIGN: Prospective observational study.
SETTING: Tertiary care hospital.
METHODS: Children up to 12 years of age who had a fever for more than five days without an identifiable infection were included. All children who were suspected of having rickettsial infections were defined as having scrub typhus if they had a positive Weil-Felix test result (OX-K 1:80 or more) and one or more of the following clinical features (after exclusion of other diagnoses): rash, edema, hepatosplenomegaly, lymphadenopathy, an eschar, and a tick bite or tick exposure.
RESULTS: Thirty-five children were diagnosed with scrub typhus between February 2010 and February 2011. The age of the patients ranged from 1.5 to 12 years. Edema, crackles/rhonchi, hepatomegaly and hypotension were encountered in 60%, 23%, 91% and 34% of patients, respectively. An eschar was observed in 11% of the cases. Complications included myocarditis with cardiogenic shock in 34% of the cases and acute kidney injury in 20% of the cases. Anicteric hepatitis and thrombocytopenia were observed in 31% and 61% of cases, respectively. One patient died.
CONCLUSIONS: High incidences of myocarditis and acute kidney injury were observed, which indicates that the children were treated at a late stage of the disease. Clinicians should be cognizant that myocarditis and acute kidney injury are serious manifestations of pediatric scrub typhus.

Copyright Â© 2011 King Saud Bin Abdulaziz University for Health Sciences. Published by Elsevier Ltd. All rights reserved.

DOI: 10.1016/j.jiph.2011.11.001

PMID: 22341847 [PubMed - indexed for MEDLINE]

166. Int J Infect Dis. 2013 Apr;17(4):e284. doi: 10.1016/j.ijid.2012.11.014. Epub 2012 Dec 17.

Scrub typhus pneumonitis.

Premaratna R(1), Ariyaratna N, Botheju WI, Bandara NK, de Silva HJ.

Author information:
(1)Department of Medicine, Faculty of Medicine, University of Kelaniya, PO Box 6, Thalagolla Road, Ragama, Sri Lanka. ranjan_premaratna@lycos.com

DOI: 10.1016/j.ijid.2012.11.014
PMID: 23253908 [PubMed - indexed for MEDLINE]

167. Emerg Infect Dis. 2011 Sep;17(9):1659-63. doi: 10.3201/eid1709.100960.

Endemic scrub typhus-like illness, Chile.

Balcells ME(1), Rabagliati R, García P, Poggi H, Oddó D, Concha M, Abarca K, Jiang J, Kelly DJ, Richards AL, Fuerst PA.

Author information:
(1)School of Medicine, Pontificia Universidad Católica de Chile, Santiago, Chile.

We report a case of scrub typhus in a 54-year-old man who was bitten by several terrestrial leeches during a trip to Chiloé Island in southern Chile in 2006. A molecular sample, identified as related to Orientia tsutsugamushi based on the sequence of the16S rRNA gene, was obtained from a biopsy specimen of the eschar on the patient's leg. Serologic analysis showed immunoglobulin G conversion against O. tsutsugamushi whole cell antigen. This case and its associated molecular analyses suggest that an Orientia-like agent is present in the Western Hemisphere that can produce scrub typhus-like illness. The molecular analysis suggests that the infectious agent is closely related, although not identical, to members of the Orientia sp. from Asia.

DOI: 10.3201/eid1709.100960
PMCID: PMC3322051
PMID: 21888791 [PubMed - indexed for MEDLINE]

168. Jpn J Infect Dis. 2013;66(6):552-4.

Scrub typhus in South India: a re-emerging infectious disease.

Stephen S(1), Kandhakumari G, Pradeep J, Vinithra SM, Siva PK, Hanifah M, Vanithadevi E.

Author information:
(1)Department of Microbiology, Mahatma Gandhi Medical College and Research Institute.

PMID: 24270151 [PubMed - indexed for MEDLINE]

169. Southeast Asian J Trop Med Public Health. 2014 Mar;45(2):395-401.

Canine Orientia tsutsugamushi infection: report of a case and its epidemicity.

Namikawa K, Tanabe A, Satake S, Enishi H, Kusaka H, Ide N, Neo S, Lynch J, Orito K, Morita T, Sahara H.

A lethargic household dog was referred to a private hospital in Japan. Diagnosis was carried out by the polymerase chain reaction (PCR) method developed for human Orientia tsutsugamushi infection using the dog's anticoagulated peripheral blood. Karp, Kato and Kuroki-type genomes were detected and the dog was diagnosed with O. tsutsugamushi infection. These findings demonstrate that dogs can act as a host for O. tsutsugamushi and the PCR method developed for human beings can be used for the diagnosis of canine O. tsutsugamushi infection. A concurrent epidemiological study examined 10 asymptomatic dogs that were fed in the same area as the sick dog. Kuroki-type genome in all dogs, Gilliam-type genome in 6 dogs and Kawasaki-type genome in 3 dogs were detected. These results provide further evidence that dogs can be naturally infected with O. tsutsugamushi outdoors and that dogs play a role as a host in the lifecycle of O. tsutsugamushi.

PMID: 24968681 [PubMed - indexed for MEDLINE]

170. Am J Trop Med Hyg. 2011 Nov;85(5):873-7. doi: 10.4269/ajtmh.2011.09-0703.

A comparative study of hepatitis caused by scrub typhus and viral hepatitis A in South Korea.

Lee J(1), Kim DM, Yun NR, Byeon YM, Kim YD, Park CG, Kim MW, Han MA.

Author information:
(1)Department of Internal Medicine, Research Center for Resistant Cells, School of Medicine, Chosun University, Gwangju, South Korea. joon3640@hanmail.net

We compared clinical features and laboratory findings of 104 patients with hepatitis A and 197 patients with scrub typhus. Nausea, vomiting, abdominal pain, hepatomegaly, and jaundice were common in patient with hepatitis A, and fever and headache were significantly more common in patients with scrub typhus. At presentation, an alanine aminotransferase (ALT) level ≥ 500 U/L was observed in 1% of scrub typhus patients and in 87.5% of hepatitis A patients (P < 0.001). A bilirubin level ≥ 1.3 mg/dL was observed in 16.8% of scrub typhus patients and 90.4% of hepatitis A patients. The ALT:lactate dehydrogenase ratio was ≤ 5 in 97.4% of the patients with scrub typhus and > 5 in 95.2% of those with hepatitis A (P < 0.001). Fever, headache, rash, and eschar are findings that indicate scrub typhus. An ALT level ≥ 500 U/L (adjusted odds ratio = 0.011) a bilirubin level ≥ 1.3 (adjusted odds ratio = 0.024), an ALT:lactate dehydrogenase ratio > 5, and hepatomegaly are indications of viral hepatitis A.

DOI: 10.4269/ajtmh.2011.09-0703
PMCID: PMC3205633
PMID: 22049041 [PubMed - indexed for MEDLINE]

171. Int J Infect Dis. 2012 Dec;16(12):e889-90. doi: 10.1016/j.ijid.2012.05.1030. Epub 2012 Jul 15.

Re-emergence of scrub typhus in northeast India.

Khan SA, Dutta P, Khan AM, Topno R, Borah J, Chowdhury P, Mahanta J.

DOI: 10.1016/j.ijid.2012.05.1030
PMID: 22796321 [PubMed - indexed for MEDLINE]

172. Risk Manag Healthc Policy. 2013 Dec 16;7:11-7. doi: 10.2147/RMHP.S55305. eCollection 2013.

Clinical risk-scoring algorithm to forecast scrub typhus severity.

Sriwongpan P(1), Krittigamas P(2), Tantipong H(3), Patumanond J(4), Tawichasri C(5), Namwongprom S(6).

Author information:
(1)Clinical Epidemiology Program, Chiang Mai University, Chiang Mai, Thailand ; Department of Social Medicine, Chiangrai Prachanukroh Hospital, Chiang Rai, Thailand. (2)Department of General Pediatrics, Nakornping Hospital, Chiang Mai, Thailand. (3)Department of Medicine, Chonburi Hospital, Chonburi, Thailand. (4)Clinical Epidemiology Program, Thammasat University, Bangkok, Thailand. (5)Clinical Epidemiology Society at Chiang Mai, Chiang Mai, Thailand. (6)Clinical Epidemiology Program, Chiang Mai University, Chiang Mai, Thailand ; Department of Radiology, Chiang Mai University, Chiang Mai, Thailand.

PURPOSE: To develop a simple risk-scoring system to forecast scrub typhus severity.
PATIENTS AND METHODS: Seven years' retrospective data of patients diagnosed with scrub typhus from two university-affiliated hospitals in the north of Thailand were analyzed. Patients were categorized into three severity groups: nonsevere, severe, and dead. Predictors for severity were analyzed under multivariable ordinal continuation ratio logistic regression. Significant coefficients were transformed into item score and summed to total scores.
RESULTS: Predictors of scrub typhus severity were age >15 years, (odds ratio [OR] =4.09), pulse rate >100/minute (OR 3.19), crepitation (OR 2.97), serum aspartate aminotransferase >160 IU/L (OR 2.89), serum albumin ≤3.0 g/dL (OR 4.69), and serum creatinine >1.4 mg/dL (OR 8.19). The scores which ranged from 0 to 16, classified patients into three risk levels: non-severe (score ≤5, n=278, 52.8%), severe (score 6-9, n=143, 27.2%), and fatal (score ≥10, n=105, 20.0%). Exact severity classification was obtained in 68.3% of cases. Underestimations of 5.9% and overestimations of 25.8% were clinically acceptable.
CONCLUSION: The derived scrub typhus severity score classified patients into their severity levels with high levels of prediction, with clinically acceptable under- and overestimations. This classification may assist clinicians in patient

prognostication, investigation, and management. The scoring algorithm should be validated by independent data before adoption into routine clinical practice.

DOI: 10.2147/RMHP.S55305
PMCID: PMC3872011
PMID: 24379733 [PubMed]

173. Risk Manag Healthc Policy. 2013 Oct 11;6:43-9. doi: 10.2147/RMHP.S52470. eCollection 2013.

Clinical indicators for severe prognosis of scrub typhus.

Sriwongpan P(1), Krittigamas P, Kantipong P, Kunyanone N, Patumanond J, Namwongprom S.

Author information:
(1)Clinical Epidemiology Program, Faculty of Medicine, Chiang Mai University, Chiang Mai, Thailand ; Department of Social Medicine, Chiang Rai Prachanukroh Hospital, Chiang Rai, Thailand.

BACKGROUND: The study explored clinical risk characteristics that may be used to forecast scrub typhus severity under routine clinical practices.
METHODS: Retrospective data were collected from patients registered at two university-affiliated tertiary care hospitals in the north of Thailand, from 2004 to 2010. Key information was retrieved from in-patient records, out patient cards, laboratory reports and registers. Patients were classified into three severity groups: nonsevere, severe (those with at least one organ involvement), and deceased. Prognostic characteristics for scrub typhus severity were analyzed by a multivariable ordinal continuation ratio regression.
RESULTS: A total of 526 patients were classified into nonsevere (n = 357), severe (n = 100), and deceased (n = 69). The significant multivariable prognostic characteristics for scrub typhus severity were increased body temperature (odds ratio [OR] = 0.58, 95% confidence interval [CI] = 0.45-0.74, P < 0.001), increased pulse rate (OR = 1.03, 95% CI = 1.01-1.05, P < 0.001), presence of crepitation (OR = 3.25, 95% CI = 1.52-6.96, P = 0.001), increased percentage of lymphocytes (OR = 0.97, 95% CI = 0.95-0.98, P = 0.001), increased aspartate aminotransferase (every 10 IU/L) (OR = 1.04, 95% CI = 1.02-1.06, P < 0.001), increased serum albumin (OR = 0.47, 95% CI = 0.27-0.80, P = 0.001), increased serum creatinine (OR = 1.83, 95% CI = 1.50-2.24, P < 0.001), and increased levels of positive urine albumin (OR = 1.43, 95% CI = 1.17-1.75, P < 0.001).
CONCLUSION: Patients suspicious of scrub typhus with low body temperature, rapid pulse rate, presence of crepitation, low percentage of lymphocyte, low serum albumin, elevated aspartate aminotransferase, elevated serum creatinine, and positive urine albumin should be monitored closely for severity progression.

DOI: 10.2147/RMHP.S52470
PMCID: PMC3826289
PMID: 24235852 [PubMed]

174. J Clin Neurosci. 2014 Dec;21(12):2251-2. doi: 10.1016/j.jocn.2014.04.014. Epub 2014 Jul 22.

Miller Fisher syndrome related to Orientia tsutsugamushi infection.

Kim KW(1), Kim YH(1), Kim BH(1), Lee CY(1), Oh MS(1), Yu KH(1), Lee BC(2).

Author information:
(1)Department of Neurology, Hallym University Sacred Heart Hospital, Hallym University College of Medicine, 22, Gwanpyeong-ro 170 beon-gil, Dongan-gu, Anyang-si, Gyeonggi-do, Republic of Korea. (2)Department of Neurology, Hallym University Sacred Heart Hospital, Hallym University College of Medicine, 22, Gwanpyeong-ro 170 beon-gil, Dongan-gu, Anyang-si, Gyeonggi-do, Republic of Korea. Electronic address: ssbrain@hallym.ac.kr.

Miller Fisher syndrome is typically associated with a preceding infection, especially with Campylobacter jejuni. We describe a patient with Miller Fisher syndrome following Orientia tsutsugamushi infection, which to our knowledge has not been previously reported.

Copyright © 2014 Elsevier Ltd. All rights reserved.

DOI: 10.1016/j.jocn.2014.04.014
PMID: 25065842 [PubMed - indexed for MEDLINE]

175. Am J Trop Med Hyg. 2011 Apr;84(4):599-607. doi: 10.4269/ajtmh.2011.09-0768.

Isolation and characterization of Orientia tsutsugamushi from rodents captured following a scrub typhus outbreak at a military training base, Bothong district, Chonburi province, central Thailand.

Rodkvamtook W(1), Ruang-Areerate T, Gaywee J, Richards AL, Jeamwattanalert P, Bodhidatta D, Sangjun N, Prasartvit A, Jatisatienr A, Jatisatienr C.

Author information:
(1)Department of Biology, Faculty of Science, Chiang Mai University, Chiang Mai, Thailand. wuttikornr@afrims.go.th

Orientia tsutsugamushi, an obligate intracellular Gram-negative bacterium, is the causative agent of scrub typhus, a vector-borne disease transmitted by infected chiggers (trombiculid mite larvae). In 2002, an outbreak of scrub typhus occurred among Royal Thai Army troops during the annual field training at a military base in Bothong district, Chonburi province, central Thailand. This report describes the outbreak investigation including its transmission cycle. Results showed that 33.9% of 174 trained troops had scrub typhus-like signs and symptoms and 9.8% of those were positive for O. tsutsugamushi-specific antibodies by indirect fluorescence antibody assay. One hundred thirty-five rodents were captured from this training area, 43% of them had antibodies against O. tsutsugamushi. Six new O. tsutsugamushi isolates were obtained from captured rodent tissues and successfully established in cell culture. Phylogenetic studies showed that these six isolates were either unique or related to a native genotype of previously described isolates from Thailand.

DOI: 10.4269/ajtmh.2011.09-0768
PMCID: PMC3062456
PMID: 21460017 [PubMed - indexed for MEDLINE]

176. Indian Pediatr. 2012 Apr;49(4):322-4.

Pediatric Scrub typhus in South Sikkim.

Gupta N(1), Mittal V, Gurung B, Sherpa U.

Author information:
(1)Zoonosis Division, National Centre for Disease Control, 22, Sham Nath Marg, Delhi-110054, India. nicdnaveen@gmail.com

We present five cases of paediatric Scrub typhus from Community Health Centre, Namchi, South Sikkim emphasize timely diagnosis of scrub typhus for appropriate management. Response to doxycycline was good, with fever subsiding within 48-72 hrs of starting the treatment. Four out of five cases completely recovered once appropriate medication was given.

PMID: 22565079 [PubMed - indexed for MEDLINE]

177. Infect Genet Evol. 2013 Apr;15:53-8. doi: 10.1016/j.meegid.2011.06.008. Epub 2011 Jun 25.

Broad-coverage molecular epidemiology of Orientia tsutsugamushi in Thailand.

Wongprompitak P(1), Anukool W, Wongsawat E, Silpasakorn S, Duong V, Buchy P, Morand S, Frutos R, Ekpo P, Suputtamongkol Y.

Author information:
(1)Department of Immunology, Faculty of Medicine Siriraj Hospital, Mahidol University, Bangkok 10700, Thailand. sipwp@mahidol.ac.th

Orientia tsutsugamushi, an obligate intracellular bacterium closely related to the genus Rickettsia, is the causative agent of scrub typhus, a major cause of febrile illness in rural areas of Asia-Pacific region. Scrub typhus is transmitted by the bite of infected mites of the genus Leptotrombidium. The region of the 56-kDa TSA gene spanning from variable domain I (VDI) to variable domain IV (VDIV) was sequenced and used for genotyping 77 O. tsutsugamushi samples from human patients confirmed with scrub typhus from 2001 to 2003 and 2009 to 2010 in different regions of Thailand. These sequences were also compared to previously published 56-kDa TSA sequences. Only 4 genotypes out of 8 previously reported in Thailand were identified, i.e. Karp, JG-v, TA763 and Kato, respectively. Two strains were not associated with known genotypes but were closely related to Taiwanese strains. The Karp genotype was confirmed as the predominant clade. The JG-v and TA763 genotypes, in contrast to other studies, also were found. The genotype TA716 was not found, except for one strain previously described.

Copyright © 2011 Elsevier B.V. All rights reserved.

DOI: 10.1016/j.meegid.2011.06.008
PMID: 21712103 [PubMed - indexed for MEDLINE]

178. Zhongguo Ji Sheng Chong Xue Yu Ji Sheng Chong Bing Za Zhi. 2013 Aug;31(4):Inside back cover.

[One case of scrub typhus patient with clinical manifestation of acute pyelonephritis].

[Article in Chinese]

Shu LH, Xu Y.

PMID: 24812870 [PubMed - indexed for MEDLINE]

179. Indian J Public Health. 2011 Apr-Jun;55(2):92-9. doi: 10.4103/0019-557X.85239.

Emergence of Schoengastiella ligula as the vector of scrub typhus outbreak in Darjeeling: has Leptotrombidium deliense been replaced?

Tilak R(1), Kunwar R, Wankhade UB, Tilak VW.

Author information:
(1)Department of Community Medicine, Armed Forces Medical College, Pune, India.
rinatilak@hotmail.com

BACKGROUND: Following a suspected outbreak of scrub typhus in Kurseong, Darjeeling, the Armed Forces Medical College, Pune was requested by the National Institute of Epidemiology and the State authorities to undertake investigation of the ongoing scrub typhus outbreak and suggest containment measures.
MATERIALS AND METHODS: The epidemic team undertook clinical, entomological and serological studies to understand the local disease pattern and delineate high risk areas, host diversity by rodent trapping using Sherman traps, mite fauna diversity, abundance and vector species identification by phase contrast microscopy for preparation of electronic database and rodent and human serological studies by Weil Felix and PCR.
RESULTS: The results indicate no association of scrub typhus with age and sex (P=0.37 and 0.74 respectively). The maximum cases occurred amongst the tea garden workers (73%) in the age group of 25-44 years. The predominant clinical presentation was fever (100%) with headache (75%), lymphadenopathy (45%) and presence of eschar (76.7%). The dominant host species (50% of trapped rodents) was shrew Suncus murinus, the index animal for scrub typhus, which contributed maximally to the vector abundance (52.96%) with a chigger index of 61.56. The trombiculid mite Schoengastiella ligula was the vector species much against the expected mite vector Leptotrombidium deliense, in the area. The study found the presence and abundance of vector species which corroborated well with the occurrence of cases in the various localities within the subdivisions.
CONCLUSION: The study thus establishes Schoengastiella ligula as the vector of scrub typhus outbreak in Kurseong, Darjeeling. Preventive and containment measures with emphasis on reduction of man - vector contact were suggested to the state authorities to contain the outbreak.

DOI: 10.4103/0019-557X.85239

PMID: 21941043 [PubMed - indexed for MEDLINE]

180. Indian J Pediatr. 2011 Nov;78(11):1365-70. doi: 10.1007/s12098-011-0470-5. Epub 2011 Jun 1.

Characteristics of pediatric scrub typhus during an outbreak in the North Eastern region of India: peculiarities in clinical presentation, laboratory findings and complications.

Dass R(1), Deka NM, Duwarah SG, Barman H, Hoque R, Mili D, Barthakur D.

Author information:
(1)Department of Pediatric Disciplines, North Eastern Indira Gandhi Regional Institute of Health and Medical Sciences (NEIGRIHMS), Mawdiangdiang, Shillong, Meghalaya, India. rashnadass@rediffmail.com

OBJECTIVE: To identify and report a recent outbreak of scrub typhus cases recorded from October 2009 to January 2010 in the state of Meghalaya, India.
METHODS: The case sheets of all the children were retrieved and reviewed retrospectively. Twenty four (24) patients, who were both clinically and serologically confirmed as scrub typhus cases were included in the study.
RESULTS: Fever for more than 1 wk duration was the most common manifestation (100%) followed by splenomegaly (45.8%), eschar (41.7%), cough (37.5%), hepatomegaly (33.3%), headache and body ache (25%), pain abdomen (25%), vomiting (20.8%), altered sensorium (16.6%), seizures (12.5%) lymphadenopathy (12.5%), and loose stools (8.3%). Meningoencephalitis was the most common complication (29.2%) followed by pneumonia (16.3%) and subconjunctival hemorrhage (8.3%).Cortical blindness, septic shock, peritonitis, myocarditis with CCF, pancytopenia, acute renal failure, coagulopathy, prolonged oxygen dependency and urinary tract infection (UTI) were found in one of each case. Hyponatremia (66.7%), elevated liver enzymes without significant rise of bilirubin (58.3%), hypoalbuminemia (52.2%) and thrombocytopenia (26%) were the other significant laboratory findings. Patients were treated with chloramphenicol, doxycycline and azithromycin. There was no mortality.
CONCLUSIONS: This is the first outbreak report from the north eastern region of India with varied clinical presentations, laboratory investigations and complications. Weil Felix test still remains fruitful for diagnosing this disease in a resource limited set up.

DOI: 10.1007/s12098-011-0470-5
PMID: 21630069 [PubMed - indexed for MEDLINE]

181. PLoS Negl Trop Dis. 2010 Dec 7;4(12):e909. doi: 10.1371/journal.pntd.0000909.

Contrasting spatial distribution and risk factors for past infection with scrub typhus and murine typhus in Vientiane City, Lao PDR.

Vallée J(1), Thaojaikong T, Moore CE, Phetsouvanh R, Richards AL, Souris M, Fournet F, Salem G, Gonzalez JP, Newton PN.

Author information:
(1)Institut de Recherche pour le Développement, UMR190, Marseille, France.

valleej@yahoo.fr

BACKGROUND: The aetiological diagnostic of fevers in Laos remains difficult due to limited laboratory diagnostic facilities. However, it has recently become apparent that both scrub and murine typhus are common causes of previous undiagnosed fever. Epidemiological data suggests that scrub typhus would be more common in rural areas and murine typhus in urban areas, but there is very little recent information on factors involved in scrub and murine typhus transmission, especially where they are sympatric - as is the case in Vientiane, the capital of the Lao PDR.
METHODOLOGY AND PRINCIPAL FINDINGS: We therefore determined the frequency of IgG seropositivity against scrub typhus (Orientia tsutsugamushi) and murine typhus (Rickettsia typhi), as indices of prior exposure to these pathogens, in randomly selected adults in urban and peri-urban Vientiane City (n = 2,002, ≥35 years). Anti-scrub and murine typhus IgG were detected by ELISA assays using filter paper elutes. We validated the accuracy of ELISA of these elutes against ELISA using serum samples. The overall prevalence of scrub and murine typhus IgG antibodies was 20.3% and 20.6%, respectively. Scrub typhus seropositivity was significantly higher among adults living in the periphery (28.4%) than in the central zone (13.1%) of Vientiane. In contrast, seroprevalence of murine typhus IgG antibodies was significantly higher in the central zone (30.8%) as compared to the periphery (14.4%). In multivariate analysis, adults with a longer residence in Vientiane were at significant greater risk of past infection with murine typhus and at lower risk for scrub typhus. Those with no education, living on low incomes, living on plots of land with poor sanitary conditions, living in large households, and farmers were at higher risk of scrub typhus and those living in neighborhoods with high building density and close to markets were at greater risk for murine typhus and at lower risk of scrub typhus past infection.
CONCLUSIONS: This study underscores the intense circulation of both scrub and murine typhus in Vientiane city and underlines difference in spatial distribution and risk factors involved in the transmission of these diseases.

DOI: 10.1371/journal.pntd.0000909
PMCID: PMC2998433
PMID: 21151880 [PubMed - indexed for MEDLINE]

182. Front Cell Infect Microbiol. 2015 Feb 3;4:186. doi: 10.3389/fcimb.2014.00186. eCollection 2014.

Orientia tsutsugamushi ankyrin repeat-containing protein family members are Type 1 secretion system substrates that traffic to the host cell endoplasmic reticulum.

VieBrock L(1), Evans SM(1), Beyer AR(1), Larson CL(2), Beare PA(2), Ge H(3), Singh S(1), Rodino KG(1), Heinzen RA(2), Richards AL(3), Carlyon JA(1).

Author information:
(1)Department of Microbiology and Immunology, Virginia Commonwealth University School of Medicine Richmond, VA, USA. (2)Coxiella Pathogenesis Section, Rocky Mountain Laboratories, National Institute of Allergy and Infectious Diseases, National Institutes of Health Hamilton, MT, USA. (3)Viral and Rickettsial Diseases Department, Naval Medical Research Center Silver Spring, MD, USA.

Scrub typhus is an understudied, potentially fatal infection that threatens one
billion persons in the Asia-Pacific region. How the causative obligate
intracellular bacterium, Orientia tsutsugamushi, facilitates its intracellular
survival and pathogenesis is poorly understood. Many intracellular bacterial
pathogens utilize the Type 1 (T1SS) or Type 4 secretion system (T4SS) to
translocate ankyrin repeat-containing proteins (Anks) that traffic to distinct
subcellular locations and modulate host cell processes. The O. tsutsugamushi
genome encodes one of the largest known bacterial Ank repertoires plus T1SS and
T4SS components. Whether these potential virulence factors are expressed during
infection, how the Anks are potentially secreted, and to where they localize in
the host cell are not known. We determined that O. tsutsugamushi
transcriptionally expresses 20 unique ank genes as well as genes for both T1SS
and T4SS during infection of mammalian host cells. Examination of the Anks'
C-termini revealed that the majority of them resemble T1SS substrates.
Escherichia coli expressing a functional T1SS was able to secrete chimeric
hemolysin proteins bearing the C-termini of 19 of 20 O. tsutsugamushi Anks in an
HlyBD-dependent manner. Thus, O. tsutsugamushi Anks C-termini are
T1SS-compatible. Conversely, Coxiella burnetii could not secrete heterologously
expressed Anks in a T4SS-dependent manner. Analysis of the subcellular
distribution patterns of 20 ectopically expressed Anks revealed that, while 6
remained cytosolic or trafficked to the nucleus, 14 localized to, and in some
cases, altered the morphology of the endoplasmic reticulum. This study identifies
O. tsutsugamushi Anks as T1SS substrates and indicates that many display a
tropism for the host cell secretory pathway.

DOI: 10.3389/fcimb.2014.00186
PMCID: PMC4315096
PMID: 25692099 [PubMed - indexed for MEDLINE]

183. Intern Med. 2012;51(17):2313-20. Epub 2012 Sep 1.

Meta-analysis of drug treatment for scrub typhus in Asia.

Fang Y(1), Huang Z, Tu C, Zhang L, Ye D, Zhu BP.

Author information:
(1)School of Public Health, Anhui Medical University, China.

OBJECTIVE: Scrub typhus is an important febrile disease in Asia, and antibiotics
have been used to treat this disease. The purpose of this study was to generate
large-scale evidence of the efficacy of different antibiotic regimens for
treating scrub typhus using a meta-analysis.
METHODS: PubMed, Elsevier ScienceDirect, Chinese Biomedical Literature Database
(CBM), China National Knowledge Infrastructure (CNKI), and Wanfang (Chinese) were
searched to identify relevant articles. The data from eligible citations were
extracted by two reviewers. All analyses were performed using the Cochrane
Collaboration Review Manager 4.2 and Stata 10.0 software programs.
RESULTS: We conducted a meta-analysis of 17 separate studies that evaluated the
efficacy of treatment with the different antibiotic regimens for scrub typhus.
The median time (h) to clearance of fever in the azithromycin-treated group was
longer than that in the chloramphenicol-treated group (weighted mean difference
[WMD] = 12.66, 95% confidence interval [CI]: 2.26,23.06). Adverse events were
2.95 (95%CI: 1.32, 6.61) times more likely to occur in the azithromycin-treated

group than in the chloramphenicol-treated group. The clearance time (days) for the main symptoms (including fever, headache, rash and lymphadenectasis) in the doxycycline-treated group was shorter than that in the chloramphenicol-treated group (WMD = -0.4, 95%CI: -0.53, -0.26) in five trials. Adverse drug events occurred significantly less frequently in the azithromycin-treated group than in the doxycycline-treated group (relative risk [RR] = 0.47, 95%CI: 0.31,0.71). CONCLUSION: Doxycycline was found to act more quickly, but more adverse drug events occur when using this regimen compared to azithromycin and chloramphenicol.

PMID: 22975540 [PubMed - indexed for MEDLINE]

184. J Med Microbiol. 2012 Feb;61(Pt 2):291-4. doi: 10.1099/jmm.0.034678-0. Epub 2011 Sep 22.

A case of scrub typhus with acalculous cholecystitis, aseptic meningitis and mononeuritis multiplex.

Hayakawa K(1), Oki M, Moriya Y, Mizuma A, Ohnuki Y, Yanagi H, Fukuda R, Ozawa H, Takizawa S, Takagi A.

Author information:
(1)Division of General Internal Medicine, Department of Medicine, Tokai University School of Medicine, Kanagawa 259-1193, Japan. khayakaw@med.wayne.edu

We present an unusual case of a patient with scrub typhus who developed acalculous cholecystitis, aseptic meningitis and mononeuritis multiplex. The patient was successfully treated with oral minocycline. To our knowledge, this is the first report of mononeuritis multiplex caused by scrub typhus.

DOI: 10.1099/jmm.0.034678-0
PMID: 21940653 [PubMed - indexed for MEDLINE]

185. Diagn Microbiol Infect Dis. 2011 Mar;69(3):271-4. doi: 10.1016/j.diagmicrobio.2010.10.005.

Scrub typhus meningoencephalitis occurring during doxycycline therapy for Orientia tsutsugamushi.

Kim DM(1), Kim YS, Cho HY, Lee YB.

Author information:
(1)Division of Infectious Diseases, Department of Internal Medicine, and Research Center for Resistant Cells, Chosun University College of Medicine, Gwangju, Republic of Korea.

We report on a scrub typhus patient who contracted meningoencephalitis during doxycycline administration. This is the first case of scrub typhus in which doxycycline concentrations were measured in serum and cerebrospinal fluid. Clinicians should be alerted to the possibility that meningoencephalitis can occur because of inadequate maintenance of serum doxycycline level caused by

antacids administered along with doxycycline.

Copyright © 2011 Elsevier Inc. All rights reserved.

DOI: 10.1016/j.diagmicrobio.2010.10.005
PMID: 21353950 [PubMed - indexed for MEDLINE]

186. PLoS Negl Trop Dis. 2013 Aug 1;7(8):e2341. doi: 10.1371/journal.pntd.0002341. Print 2013.

Antibody prevalence and factors associated with exposure to Orientia tsutsugamushi in different aboriginal subgroups in West Malaysia.

Tay ST(1), Mohamed Zan HA, Lim YA, Ngui R.

Author information:
(1)Tropical Infectious Diseases Research and Education Centre, Department of Medical Microbiology, Faculty of Medicine, University of Malaya, Kuala Lumpur, Malaysia. tayst@um.edu.my

BACKGROUND: Limited data is available on the current status of scrub typhus infection in the aboriginal population in Malaysia. This study was aimed to provide recent data on the degree of exposure of 280 individuals from seven aboriginal subgroups to Orientia tsutsugamushi (causative agent of scrub typhus) in West Malaysia. The environment, socioeconomic and behavioural risk factors associated with the disease were also investigated.
METHODS/FINDINGS: The antibody prevalence to O. tsutsugamushi ranged from 0 to 36.4% in seven subgroups, with high prevalence rates noted in subgroups involved in agricultural activity and the lowest prevalence rates noted in subgroups whose main occupations were associated to fishing. Univariate analysis indicated populations with age above 18 years (OR = 1.15, 95% CI = 1.02-1.30, P = 0.015), working (OR = 1.99, 95% CI = 1.01-3.92, P = 0.044), working at agriculture area (OR = 1.18, 95% CI = 0.98-1.42, P = 0.031), receiving household income less than US$ 166.7 (RM500) per month (OR = 2.43, 95% CI = 1.16-5.11, P = 0.016) and having close contact with animal pets (OR = 4.06, 95% CI = 1.20-13.76, P = 0.016) are significantly associated with exposure to O. tsutsugamushi. Multivariate analysis confirms that participants who are above 18 years old, receiving household income less than US$ 166.7 (RM500) per month and having close contact with animal pets are 3.6 times (95% CI = 1.81-7.03, P<0.001), 1.3 times (95% CI = 1.14-1.64, P = 0.002) and 1.2 times (95% CI = 1.05-1.06, P = 0.006) more likely to have exposure to O. tsutsugamushi, respectively.
CONCLUSION: The present study indicates that scrub typhus is still an important disease in the aboriginal population in Malaysia. Awareness about the disease and education on the preventive measures are important in reducing the risk of acquiring scrub typhus in the population studied.

DOI: 10.1371/journal.pntd.0002341
PMCID: PMC3731234
PMID: 23936576 [PubMed - indexed for MEDLINE]

187. J Korean Med Sci. 2012 Mar;27(3):231-5. doi: 10.3346/jkms.2012.27.3.231. Epub 2012 Feb 23.

Persistence of Orientia tsutsugamushi in humans.

Chung MH(1), Lee JS, Baek JH, Kim M, Kang JS.

Author information:
(1)Department of Internal Medicine, Inha University School of Medicine, Incheon, Korea.

We investigated the persistence of viable Orientia tsutsugamushi in patients who had recovered from scrub typhus. Blood specimens were available from six patients with scrub typhus who were at 1 to 18 months after the onset of the illness. The EDTA-treated blood specimens were inoculated into ECV304 cells, and cultures were maintained for 7 months. Sequencing of the 56-kDa type-specific antigen gene of O. tsutsugamushi was performed to ascertain the homology of isolates. O. tsutsugamushi was isolated from all six patients, and nucleotide sequences of isolates serially collected from each patient were identical in all five patients in whom nucleotide sequences were compared. One patient relapsed 2 days after completion of antibiotic therapy; two patients complained of weakness for 1 to 2.5 months after the illness; one patient underwent coronary angioplasty 6 months later; and one patient suffered from a transient ischemic attack 8 months later. This finding suggests that O. tsutsugamushi causes chronic latent infection, which may be associated with certain clinical illnesses, preceded by scrub typhus. Antibiotic therapy abates the symptoms of scrub typhus, but does not eradicate O. tsutsugamushi from the human body.

DOI: 10.3346/jkms.2012.27.3.231
PMCID: PMC3286767
PMID: 22379331 [PubMed - indexed for MEDLINE]

188. Med Vet Entomol. 2011 Jun;25(2):169-77. doi: 10.1111/j.1365-2915.2010.00941.x. Epub 2011 Jan 12.

Identification of potential hosts and vectors of scrub typhus and tick-borne spotted fever group rickettsiae in eastern Taiwan.

Kuo CC(1), Huang CL, Wang HC.

Author information:
(1)Research and Diagnostic Centre, Centres for Disease Control, Taipei, Taiwan, R.O.C.

Scrub typhus and tick-borne spotted fever group (SFG) rickettsioses are transmitted by chiggers (larval trombiculid mites) and hard ticks, respectively. We assessed exposure to these disease vectors by extensively sampling both chiggers and ticks and their small mammal hosts in eastern Taiwan during 2007 and 2008. The striped field mouse Apodemus agrarius Pallas (Rodentia: Muridae) was the most common of the small mammals (36.1% of 1393 captures) and presented the highest rate of infestation with both chiggers (47.8% of 110 760) and ticks (78.1% of 1431). Leptotrombidium imphalum Vercammen-Grandjean & Langston (Trombidiformes: Trombiculidae) and immature Rhipicephalus haemaphysaloides Supino (Ixodida: Ixodidae) were the most abundant chiggers (84.5%) and ticks (>99%) identified, respectively. Immunofluorescent antibody assay revealed high

seropositive rates of rodents against Orientia tsutsugamushi Hyashi
(Rickettsiales: Rickettsiaceae), the aetiological agent of scrub typhus (70.0% of
437 rodents), and tick-borne SFG rickettsiae (91.9% of 418 rodents). The current
study represents a first step towards elucidating the potential hosts and vectors
in the enzootic transmission of O. tsutsugamushi and tick-borne SFG rickettsiae
in Taiwan. Further studies should focus on characterizing pathogens in L.
imphalum and R. haemaphysaloides, as well as the proclivity of both vectors to
humans. Uncovering the main hosts of adult ticks is also critical for the
prevention of SFG rickettsial infections.

DOI: 10.1111/j.1365-2915.2010.00941.x
PMID: 21223345 [PubMed - indexed for MEDLINE]

189. Future Microbiol. 2015;10(4):537-64. doi: 10.2217/fmb.14.141.

Strategies for detecting rickettsiae and diagnosing rickettsial diseases.

Luce-Fedrow A(1), Mullins K, Kostik AP, St John HK, Jiang J, Richards AL.

Author information:
(1)Naval Medical Research Center, 503 Robert Grant Avenue, Silver Spring, MD
20910, USA.

Rickettsial diseases and scrub typhus constitute a group of the oldest known
vector-borne diseases. The cosmopolitan distribution of the vectors that transmit
rickettsiae and orientiae leads to a worldwide prevalence of these diseases.
Despite their significant historical status, detection and diagnosis of these
diseases are still evolving today. Serological methods remain among the most
prevalent techniques used for the detection/diagnosis of rickettsial diseases and
scrub typhus. Molecular techniques have been instrumental in increasing the
sensitivity/specificity of diagnosis, identifying new Rickettsia and Orientia
species and have enhanced epidemiological capabilities when used in combination
with serological methods. In this review, we discuss these techniques and their
associated pros and cons.

DOI: 10.2217/fmb.14.141
PMID: 25865193 [PubMed - indexed for MEDLINE]

190. Am J Trop Med Hyg. 2012 Apr;86(4):559. doi: 10.4269/ajtmh.2012.11-0616.

Scrub typhus without eruption.

Yoshimoto T(1), Yoshimoto T.

Author information:
(1)Hitachiota City Kegano Clinic, Hitachiota City, Ibaraki, Japan.
ymta@beige.ocn.ne.jp

DOI: 10.4269/ajtmh.2012.11-0616
PMCID: PMC3403754
PMID: 22492134 [PubMed - indexed for MEDLINE]

191. J Indian Med Assoc. 2012 Apr;110(4):255, 257.

A case of scrub typhus with haematuria.

Devarajan TV(1), Victor SM, Khan S, Shrilekha V.

Author information:
(1)Department of Internal Medicine, Apollo First Med Hospitals, Chennai 600010.

Scrub typhus, once endemic in India, has virtually disappeared from our country. Recently, there have been many case reports of scrub typhus. Haematuria in scrub typhus is a rare presentation, but it has been reported earlier. Here one such case of a 55-year-old man is reported who presented with fever and arthralgia. There was an erythematous maculopapular rash on his body. One enlarged lymph node in the right axilla and hepatomegaly were elicited. Weil-Felix reaction Ox-K was positive with 1:360 dilution. Doxycycline was advised for 7 days. Two months after follow-up, he had no symptoms.

PMID: 23025229 [PubMed - indexed for MEDLINE]

192. Vector Borne Zoonotic Dis. 2013 Aug;13(8):545-9. doi: 10.1089/vbz.2012.1049. Epub 2013 May 13.

Serological evidence for exposure of dogs to Rickettsia conorii, Rickettsia typhi, and Orientia tsutsugamushi in Sri Lanka.

Nanayakkara DM(1), Rajapakse RP, Wickramasinghe S, Kularatne SA.

Author information:
(1)Department of Pathobiology, Faculty of Veterinary Medicine and Animal Science, University of Peradeniya, Sri Lanka.

Vector-borne rickettsial infection is a major cause of febrile illnesses throughout the world. Although vertebrates hosting the vectors play a vital role in the natural cycle of rickettsiae, studies have not been conducted on them in Sri Lanka. Therefore, the present study was designed to determine the exposure of dog population in Rajawatta, Thambavita, and areas of the Western Slopes and Unawatuna of Sri Lanka to rickettsial pathogens. A total of 123 dog blood samples were collected from those areas. Samples were tested for antibodies against Rickettsia conorii (RC) of the spotted fever group (SFG), Rickettsia typhi (RT) of the typhus group (TG), and Orientia tsutsugamushi (OT) of the scrub typhus group (ST) of rickettsiae by indirect immunofluorescence antibody test (IFA). Samples with titers ≥ 1:64 were considered as positive in this study. Collectively, 49% dogs were found to have antibodies against the rickettsial agents. Of the dogs, 42%, 24%, and 2% had antibodies against RC, OT, and RT, respectively. The seropositive rate of 100% was observed in areas of the Western Slopes, whereas the lowest rate of 20% was in Unawatuna. Among the positive samples, antibody titers against RC and OT ranged from 1/64 to 1/8192. In contrast, the few dogs that tested positive for RT showed very low titers of 1/64 and 1/128. Results of this study show the extent of exposure to the pathogen and its dispersion in the natural ecology. We suggest that dogs could be acting as

reservoirs in the rickettsial transmission cycle or could be effective tracer animals that can be used to detect areas with potential for future outbreaks.

DOI: 10.1089/vbz.2012.1049
PMCID: PMC3741424
PMID: 23930973 [PubMed - indexed for MEDLINE]

193. Indian J Med Res. 2015 Apr;141(4):417-22. doi: 10.4103/0971-5916.159279.

DHR-ICMR Guidelines for diagnosis & management of Rickettsial diseases in India.

Rahi M(1), Gupte MD, Bhargava A, Varghese GM, Arora R.

Author information:
(1)Writing Committee of the DHR-ICMR Guidelines for Diagnosis & Management of Rickettsial Diseases in India; Indian Council of Medical Research Task Force on Development of Guidelines for Diagnosis & Management of Rickettsial Diseases, India.

Rickettsial diseases, caused by a variety of obligate intracellular, gram-negative bacteria from the genera Rickettsia, Orientia, Ehrlichia, Neorickettsia, Neoehrlichia, and Anaplasma, belonging to the Alphaproteobacteria, are considered some of the most covert emerging and re-emerging diseases and are being increasingly recognized. Among the major groups of rickettsioses, commonly reported diseases in India are scrub typhus, murine flea-borne typhus, Indian tick typhus and Q fever. Rickettsial infections are generally incapacitating and difficult to diagnose; untreated cases have case fatality rates as high as 30-45 per cent with multiple organ dysfunction, if not promptly diagnosed and appropriately treated. The vast variability and non-specific presentation of this infection have often made it difficult to diagnose clinically. Prompt antibiotic therapy shortens the course of the disease, lowers the risk of complications and in turn reduces morbidity and mortality due to rickettsial diseases. There is a distinct need for physicians and health care workers at all levels of care in India to be aware of the clinical features, available diagnostic tests and their interpretation, and the therapy of these infections. Therefore, a Task Force was constituted by the Indian Council of Medical Research (ICMR) to formulate guidelines for diagnosis and management of rickettsial diseases. These guidelines include presenting manifestations, case definition, laboratory criteria (specific and supportive investigations) and treatment.

DOI: 10.4103/0971-5916.159279
PMCID: PMC4510721
PMID: 26112842 [PubMed - indexed for MEDLINE]

194. Trop Doct. 2011 Jul;41(3):185-6. doi: 10.1258/td.2011.110079.

Scrub typhus presenting as acute abdomen.

Mahajan SK(1), Babu SN, Sharma D, Singh D, Kanga A, Kaushal SS.

Author information:
(1)Department of Medicine, IG Medical College, Shimla, Himachal Pradesh, India.

sanjay_mahajan64@rediffmail.com

Scrub typhus, a zoonosis, is a systemic febrile illness with diverse clinical manifestations. It may also present with signs and symptoms of acute abdomen. We present two serologically confirmed cases of scrub typhus presenting with acute abdomen that were managed conservatively with antibiotics.

DOI: 10.1258/td.2011.110079
PMID: 21724691 [PubMed - indexed for MEDLINE]

195. Am J Trop Med Hyg. 2014 Sep;91(3):451-60. doi: 10.4269/ajtmh.14-0191. Epub 2014 Jun 23.

Rickettsial infections in Southeast Asia: implications for local populace and febrile returned travelers.

Aung AK(1), Spelman DW(2), Murray RJ(2), Graves S(2).

Author information:
(1)Department of Infectious Diseases and Microbiology, Alfred Hospital, Melbourne, Victoria, Australia; Monash University, Melbourne, Victoria, Australia; Department of Infectious Diseases and Microbiology and Pathwest Laboratory Medicine, Sir Charles Gairdner Hospital, Perth, Western Australia, Australia; School of Pathology and Laboratory Medicine, University of Western Australia, Perth, Western Australia, Australia; Australian Rickettsial Reference Laboratory Foundation, Geelong Hospital, Geelong, Victoria, Australia; New South Wales Health Pathology, Newcastle, New South Wales, Australia; University of Newcastle, Newcastle, New South Wales, Australia a.aung@alfred.org.au.
(2)Department of Infectious Diseases and Microbiology, Alfred Hospital, Melbourne, Victoria, Australia; Monash University, Melbourne, Victoria, Australia; Department of Infectious Diseases and Microbiology and Pathwest Laboratory Medicine, Sir Charles Gairdner Hospital, Perth, Western Australia, Australia; School of Pathology and Laboratory Medicine, University of Western Australia, Perth, Western Australia, Australia; Australian Rickettsial Reference Laboratory Foundation, Geelong Hospital, Geelong, Victoria, Australia; New South Wales Health Pathology, Newcastle, New South Wales, Australia; University of Newcastle, Newcastle, New South Wales, Australia.

Rickettsial infections represent a major cause of non-malarial febrile illnesses among the residents of Southeast Asia and returned travelers from that region. There are several challenges in recognition, diagnosis, and management of rickettsioses endemic to Southeast Asia. This review focuses on the prevalent rickettsial infections, namely, murine typhus (Rickettsia typhi), scrub typhus (Orientia tsutsugamushi), and members of spotted fever group rickettsiae. Information on epidemiology and regional variance in the prevalence of rickettsial infections is analyzed. Clinical characteristics of main groups of rickettsioses, unusual presentations, and common pitfalls in diagnosis are further discussed. In particular, relevant epidemiologic and clinical aspects on emerging spotted fever group rickettsiae in the region, such as Rickettsia honei, R. felis, R. japonica, and R. helvetica, are presented. Furthermore, challenges in laboratory diagnosis and management aspects of rickettsial infections unique to Southeast Asia are discussed, and data on emerging resistance to antimicrobial drugs and treatment/prevention options are also reviewed.

© The American Society of Tropical Medicine and Hygiene.

DOI: 10.4269/ajtmh.14-0191
PMCID: PMC4155544
PMID: 24957537 [PubMed - indexed for MEDLINE]

196. PLoS Negl Trop Dis. 2013 Dec 26;7(12):e2493. doi: 10.1371/journal.pntd.0002493. eCollection 2013.

Scrub typhus in mainland China, 2006-2012: the need for targeted public health interventions.

Zhang WY(1), Wang LY(1), Ding F(2), Hu WB(3), Soares Magalhaes RJ(3), Sun HL(1), Liu YX(4), Liu QY(2), Huang LY(1), Clements AC(3), Li SL(1), Li CY(1).

Author information:
(1)Institute of Disease Control and Prevention, Academy of Military Medical Science, Beijing, P.R. China. (2)Chinese Center for Disease Control and Prevention, Beijing, P.R. China. (3)School of Population Health, Infectious Disease Epidemiology Unit, The University of Queensland, Brisbane, Australia. (4)Chinese People's Liberation Army General Hospital, Beijing, P.R. China.

DOI: 10.1371/journal.pntd.0002493
PMCID: PMC3873277
PMID: 24386495 [PubMed - indexed for MEDLINE]

197. Infect Genet Evol. 2013 Apr;15:35-42. doi: 10.1016/j.meegid.2011.01.004. Epub 2011 Jan 15.

Molecular epidemiology of Orientia tsutsugamushi in Cambodia and Central Vietnam reveals a broad region-wide genetic diversity.

Duong V(1), Mai TT, Blasdell K, Lo le V, Morvan C, Lay S, Anukool W, Wongprompitak P, Suputtamongkol Y, Laurent D, Richner B, Ra C, Chien BT, Frutos R, Buchy P.

Author information:
(1)Institut Pasteur in Cambodia, Virology Unit, 5 Monivong Blvd., Phnom Penh, Cambodia.

Scrub typhus is an acute infectious disease caused by an obligate intracellular bacterium Orientia tsutsugamushi following the bite of infected trombiculid mites of the genus Leptotrombidium. This zoonotic disease is a major cause of febrile illness in the Asia-Pacific region, with a large spectrum of clinical manifestations from unapparent or mild disease to fatal disease. O. tsutsugamushi is characterized by a very high genomic plasticity and a large number of antigenic variants amongst strains. The 56-kDa type specific antigen (TSA) gene, encoding the major antigenic protein, was used as reference to investigate the genetic relationships between the strains and to genotype O. tsutsugamushi isolates. The open reading frame of the 56-kDa TSA gene of 41 sequences (28 Cambodian and 13 Vietnamese strains) from patient samples were sequenced and used

for genotyping. The 28 Cambodian isolates clustered into 5 major groups, including Karp (43.5%), JG-v (25%), Kato/TA716 (21.5%), TA763 (3.5%) and Gilliam (3.5%). Karp (77%), TA763 (15.5%) and JG-v (7.5%) strains were identified amongst the 13 Vietnamese isolates. This is the first countrywide genotyping description in Cambodia and in Central Vietnam. These results demonstrate the considerable diversity of genotypes in co-circulation in both countries. The genotyping result might raise awareness amongst Cambodian and Vietnamese clinicians of the high genetic diversity of circulating O. tsutsugamushi strains and provides unique and beneficial data for serological and molecular diagnosis of scrub typhus infections as well as raw materials for future studies and vaccine development.

Copyright © 2011 Elsevier B.V. All rights reserved.

DOI: 10.1016/j.meegid.2011.01.004
PMID: 21241829 [PubMed - indexed for MEDLINE]

198. Vector Borne Zoonotic Dis. 2013 Aug;13(8):565-71. doi: 10.1089/vbz.2012.1083. Epub 2013 May 13.

Co-infection with Arsenophonus nasoniae and Orientia tsutsugamushi in a traveler.

Edouard S(1), Subramanian G, Lefevre B, Dos Santos A, Pouedras P, Poinsignon Y, Mediannikov O, Raoult D.

Author information:
(1)Aix Marseille Université, URMITE, UM63, CNRS 7278, IRD 198, Inserm 1095, Marseille, France.

Here we report a case of co-infection with Orientia tsutsugamushi, the causative agent of scrub typhus, and Arsenophonus nasoniae in a woman with a rash and an eschar who returned from a trip to Southeast Asia. A. nasoniae was previously considered to be a secondary insect and tick endosymbiont of unknown pathogenicity in humans. We amplified both O. tsutsugamushi and A. nasoniae DNA from a skin eschar with qPCR, and a seroconversion for O. tsutsugamushi and A. nasoniae was observed with immunofluorescence assays and western blotting for this patient. And we used 2-D western blotting with an A. nasoniae antigen and polyclonal mouse anti-A. nasoniae antibodies produced in our laboratory to detect the specific antigenic A. nasoniae proteins.

DOI: 10.1089/vbz.2012.1083
PMID: 23930974 [PubMed - indexed for MEDLINE]

199. Emerg Infect Dis. 2015 Feb;21(2):373-5. doi: 10.3201/eid2102.140860.

Orientia tsutsugamushi in lung of patient with acute respiratory distress syndrome, France, 2013.

Angelakis E, Patrick G, Peloni JM, Wey PF, Perreal C, Raoult D.

DOI: 10.3201/eid2102.140860
PMCID: PMC4313642
PMID: 25625312 [PubMed - indexed for MEDLINE]

200. Infect Genet Evol. 2013 Apr;15:25-34. doi: 10.1016/j.meegid.2010.08.015. Epub 2010 Sep 18.

Diversity of Orientia tsutsugamushi clinical isolates in Cambodia reveals active selection and recombination process.

Duong V(1), Blassdell K, May TT, Sreyrath L, Gavotte L, Morand S, Frutos R, Buchy P.

Author information:
(1)Virology Unit, Institut Pasteur in Cambodia, 5 Monivong blvd, PO Box 983, Phnom Penh, Cambodia.

Orientia tsutsugamushi, the causative agent of scrub typhus in South East Asia and Pacific, is an obligate intracellular bacterium closely related to the Rickettsia. The pathogen is transmitted to humans through the bites of infected larvae of trombiculid mites of the genus Leptotrombidium in which is maintained trough vertical transmission mechanism. The infection in rodents has been described in over 20 species. Scrub typhus is commonly confused with other tropical fevers and late diagnosis and treatment can lead to severe organ failures and a strain-dependent mortality rate of up to 50%. A MLST scheme associating seven core function genes: adk, lepB, lipA, lipB, secY, sodB and sucA was developed and validated on seven Cambodian strains detected in patients and two complete reference genomes from Korea and Japan. Sequence data were analyzed both with respect to sequence type (ST) diversity and DNA polymorphism. Differing trends were revealed. DNA polymorphism and phylogeny of individual gene loci indicated a significant level of recombination and genetic diversity. However, the ST distribution is clearly clonal and the clinical situation can be summarized by the formula: one patient, one strain, one ST. This contradiction is only apparent and is most likely the consequence of the unique life cycle of O. tsutsugamushi. The quasi exclusive vertical transmission mode in mites generates repeated bottlenecks and small-size populations and strongly limits genetic diversity. O. tsutsugamushi has developed specific mechanisms for generating genetic diversity which include recombination, duplication and conjugation. Recombination and other mechanisms for increasing genetic diversity are likely to occur in rodents which can act as maintenance hosts, although occurrence in mites cannot be excluded. Consequences for the epidemiology of scrub typhus are discussed.

Copyright © 2010 Elsevier B.V. All rights reserved.

DOI: 10.1016/j.meegid.2010.08.015
PMID: 20854933 [PubMed - indexed for MEDLINE]

201. Braz J Infect Dis. 2012 Jul-Aug;16(4):407-8. doi: 10.1016/j.bjid.2012.06.014.

Eschar: a cutaneous clue to scrub typhus.

Aggarwal S(1), Sharma A, Sharma V.

Author information:

(1)Department of Medicine, University College of Medical Sciences, University of Delhi, India.

DOI: 10.1016/j.bjid.2012.06.014
PMID: 22846139 [PubMed - indexed for MEDLINE]

202. Front Cell Infect Microbiol. 2013 Jan 4;2:170. doi: 10.3389/fcimb.2012.00170. eCollection 2012.

Approaches to vaccines against Orientia tsutsugamushi.

Valbuena G(1), Walker DH.

Author information:
(1)Department of Pathology, University of Texas Medical Branch Galveston, TX, USA. gvalbuen@utmb.edu

Scrub typhus is a severe mite-borne infection caused by Orientia tsutsugamushi, an obligately intracellular bacterium closely related to Rickettsia. The disease explains a substantial proportion of acute undifferentiated febrile cases that require hospitalization in rural areas of Asia, the North of Australia, and many islands of the Pacific Ocean. Delayed antibiotic treatment is common due to the lack of effective commercially available diagnostic tests and the lack of specificity of the early clinical presentation. The systemic infection of endothelial cells that line the vasculature with Orientia can lead to many complications and fatalities. In survivors, immunity does not last long, and is poorly cross-reactive among numerous strains. In addition, chronic infections are established in an unknown number of patients. All those characteristics justify the pursuit of a prophylactic vaccine against O. tsutsugamushi; however, despite continuous efforts to develop such a vaccine since World War II, the objective has not been attained. In this review, we discuss the history of vaccine development against Orientia to provide a clear picture of the challenges that we continue to face from the perspective of animal models and the immunological challenges posed by an intracellular bacterium that normally triggers a short-lived immune response. We finish with a proposal for development of an effective and safe vaccine for scrub typhus through a new approach with a strong focus on T cell-mediated immunity, empirical testing of the immunogenicity of proteins encoded by conserved genes, and assessment of protection in relevant animal models that truly mimic human scrub typhus.

DOI: 10.3389/fcimb.2012.00170
PMCID: PMC3539663
PMID: 23316486 [PubMed - indexed for MEDLINE]

203. Int J Infect Dis. 2013 Nov;17(11):e988-92. doi: 10.1016/j.ijid.2013.05.014. Epub 2013 Jul 17.

Rickettsioses in the central hills of Sri Lanka: serological evidence of increasing burden of spotted fever group.

Kularatne SA(1), Rajapakse RP, Wickramasinghe WM, Nanayakkara DM, Budagoda SS, Weerakoon KG, Edirisinghe JS, Premaratna R.

Author information:
(1)Department of Medicine, Faculty of Medicine, University of Peradeniya, Kandy, Sri Lanka.

OBJECTIVES: To study the epidemiology, clinical features, and changing pattern of rickettsial infections on the western slopes of the hilly Central Province of Sri Lanka over 6 years.
METHODS: All patients with rickettsial infections who presented to the Teaching Hospital, Peradeniya were studied prospectively from January 2002 to December 2007. An immunofluorescent antibody assay (IFA) was used to confirm the diagnosis.
RESULTS: Of the 371 clinical cases of rickettsial infection, 122 underwent IFA to confirm the diagnosis. Species-specific IgG antibodies were positive in 105/122 (86.1%) cases: 43/105 (40.9%) to Rickettsia conorii and 6/105 (5.7%) to Orientia tsutsugamushi, with mixed antibody reactivity to more than one antigen in 56/105 (53.3%) cases, including Rickettsia typhi in 27/105 (25.7%). Among those with mixed IgG reactivity, IgM assays were used to detect pathogens responsible for acute infections. Finally, a total of 55 spotted fever group (SFG) infections, seven scrub typhus infections, and one case of murine typhus were identified. Of the 105 positive cases, 53 (50.5%) were male and 52 (49.5%) were female, and the mean age was 40 years (range 11-83 years). In the SFG patients, 13/55 (24%) had severe vasculitis with fern leaf type skin necrosis and 17/55 (31%) had arthritis. Three patients (5%) had an altered level of consciousness. A patient with scrub typhus had transient deafness. None of the 105 patients had an eschar.
CONCLUSIONS: It appears that SFG rickettsioses are on the rise in the hilly Central Province of Sri Lanka, whilst murine typhus and scrub typhus are present at a lower rate.

Copyright © 2013 International Society for Infectious Diseases. Published by Elsevier Ltd. All rights reserved.

DOI: 10.1016/j.ijid.2013.05.014
PMID: 23871280 [PubMed - indexed for MEDLINE]

204. PLoS Negl Trop Dis. 2016 Apr 6;10(4):e0004577. doi: 10.1371/journal.pntd.0004577. eCollection 2016.

The Aetiologies and Impact of Fever in Pregnant Inpatients in Vientiane, Laos.

Chansamouth V(1,)(2), Thammasack S(3), Phetsouvanh R(1), Keoluangkot V(2,)(3), Moore CE(4), Blacksell SD(4,)(5), Castonguay-Vanier J(1), Dubot-Pérès A(1,)(4,)(6), Tangkhabuanbutra J(1), Tongyoo N(5), Souphaphonh P(7), Sengvilaipaseuth O(1), Vongsouvath M(1), Phommasone K(1), Sengdethka D(1), Seurbsanith A(1), Craig SB(8), Hermann L(9), Strobel M(2), Newton PN(1,)(4).

Author information:
(1)Lao-Oxford-Mahosot Hospital-Wellcome Trust Research Unit (LOMWRU), Microbiology Laboratory, Mahosot Hospital, Vientiane, Lao PDR. (2)Institut de la Francophonie pour la Medecine Tropicale (IFMT), Vientiane, Lao PDR. (3)Infectious Disease Center, Mahosot Hospital, Vientiane, Lao PDR. (4)Centre for Tropical Medicine and Global Health, Nuffield Department of Medicine Research Building, University of Oxford, United Kingdom. (5)Mahidol-Oxford Research Unit (MORU),

Faculty of Tropical Medicine, Mahidol University, Bangkok, Thailand. (6)UMR_D 190 "Emergence des Pathologies Virales", Aix- Marseille University, IRD French Institute of Research for Development, EHESP French School of Public Health, Marseille, France. (7)Department of Gynecology and Obstetrics, Mahosot Hospital, Vientiane, Lao PDR. (8)WHO/FAO/OIE Collaborating Centre for Reference and Research on Leptospirosis, Queensland Health Forensic and Scientific Services, Australia. (9)Department of Virology, Armed Forces Research Institute of Medical Sciences (AFRIMS), Bangkok, Thailand.

INTRODUCTION: Laos has the highest maternal mortality ratio in mainland Southeast Asia and a high incidence of infectious diseases. Globally, malaria has been the pathogen most intensively investigated in relation to impact on pregnancy, but there has been relatively little research on the aetiology and impact of other diseases. We therefore aimed to determine the causes and impact of fever in pregnant women admitted to two central hospitals in Vientiane City, Lao PDR (Laos).
MATERIALS AND METHODS: This hospital-based prospective study was conducted in Mahosot Hospital and the Mother and Child Hospital, Vientiane, between 2006 and 2010, with the aim to recruit 250 consenting pregnant women admitted with tympanic temperature ≥37.5°C. Primary outcome was the cause of fever and secondary outcomes were pregnancy outcomes. Specific investigations (culture, antigen, molecular and serological tests) were performed to investigate causes of fever. After discharge, all pregnant women were asked to return for review and convalescence serum on day 10-14 and were monitored until delivery.
PRINCIPLE FINDINGS: 250 pregnant women were recruited to this study between February 2006 and November 2010. Fifty percent were pregnant for the first time. Their median (range) gestational age on admission was 24 (4-43) weeks. The median (range) tympanic admission temperature was 38.5°C (37.5-40.5°C). Fifteen percent of patients stated that they had taken antibiotics before admission. Headache, myalgia, back pain and arthralgia were described by >60% of patients and 149 (60%) were given a laboratory diagnosis. Of those with confirmed diagnoses, 132 (53%) had a single disease and 17 (7%) had apparent mixed diseases. Among those who had a single disease, dengue fever was the most common diagnosis, followed by pyelonephritis, scrub typhus, murine typhus and typhoid. Patients were also diagnosed with tuberculosis, appendicitis, Staphylococcus aureus septicemia, leptospirosis, Japanese encephalitis virus infection and Plasmodium falciparum malaria. Severe consequences, including maternal death, miscarriage, stillbirth, low birth weight and preterm birth, were found among 28 (78%) mothers with dengue fever, rickettsioses and typhoid.
CONCLUSION: Fevers other than malaria, such as dengue, pyelonephritis, rickettsioses and typhoid are common causes of fever during pregnancy in the Asian tropics. Further investigations of their impact in the community on maternal death, fetal loss, vertical transmission, low birth weight and preterm birth are needed.

DOI: 10.1371/journal.pntd.0004577
PMCID: PMC4822858
PMID: 27050192 [PubMed - indexed for MEDLINE]

205. Am J Trop Med Hyg. 2013 Oct;89(4):797-9. doi: 10.4269/ajtmh.13-0402. Epub 2013 Sep 3.

Molecular confirmation of co-infection by pathogenic Leptospira spp. and Orientia

tsutsugamushi in patients with acute febrile illness in Thailand.

Sonthayanon P(1), Chierakul W, Wuthiekanun V, Limmathurotsakul D, Amornchai P, Smythe LD, Day NP, Peacock SJ.

Author information:
(1)Department of Molecular Tropical Medicine and Genetics, Department of Clinical Tropical Medicine, Mahidol-Oxford Tropical Medicine Research Unit, Department of Tropical Hygiene, and Department of Microbiology and Immunology, Faculty of Tropical Medicine, Mahidol University, Bangkok, Thailand; WHO/FAO/OIE Collaborating Centre for Reference and Research on Leptospirosis, Western Pacific Region, Communicable Disease Unit, Queensland Health Forensic and Scientific Services, Brisbane, Queensland, Australia; Center for Tropical Medicine, Nuffield Department of Clinical Medicine, University of Oxford, Oxford, United Kingdom; Department of Medicine, University of Cambridge, Addenbrooke's Hospital, Cambridge, United Kingdom.

Leptospirosis and scrub typhus are major causes of acute febrile illness in rural Asia, where co-infection is reported to occur based on serologic evidence. We re-examined whether co-infection occurs by using a molecular approach. A duplex real-time polymerase chain reaction was developed that targeted a specific 16S ribosomal RNA gene of pathogenic Leptospira spp. and Orientia tsutsugamushi. Of 82 patients with an acute febrile illness who had dual infection on the basis of serologic tests, 5 (6%) had polymerase chain reaction results positive for both pathogens. We conclude that dual infection occurs, but that serologic tests may overestimate the frequency of co-infections.

DOI: 10.4269/ajtmh.13-0402
PMCID: PMC3795116
PMID: 24002486 [PubMed - indexed for MEDLINE]

206. BMC Infect Dis. 2011 Mar 30;11:79. doi: 10.1186/1471-2334-11-79.

Otalgia and eschar in the external auditory canal in scrub typhus complicated by acute respiratory distress syndrome and multiple organ failure.

Lee BJ(1), Chen CY, Hu SY, Tsan YT, Lin TC, Wang LM.

Author information:
(1)Intensive Care Unit, Department of Internal Medicine, Taichung Veterans General Hospital, Taichung City, 00407, Taiwan.

BACKGROUND: Scrub typhus, a mite-transmitted zoonosis caused by Orientia tsutsugamushi, is an endemic disease in Taiwan and may be potentially fatal if diagnosis is delayed.
CASE PRESENTATIONS: We encountered a 23-year-old previously healthy Taiwanese male soldier presenting with the right ear pain after training in the jungle and an eleven-day history of intermittent high fever up to 39°C.
Amoxicillin/clavulanate was prescribed for otitis media at a local clinic. Skin rash over whole body and abdominal cramping pain with watery diarrhea appeared on the sixth day of fever. He was referred due to progressive dyspnea and cough for 4 days prior to admission in our institution. On physical examination, there were cardiopulmonary distress, icteric sclera, an eschar in the right external

auditory canal and bilateral basal rales. Laboratory evaluation revealed thrombocytopenia, elevation of liver function and acute renal failure. Chest x-ray revealed bilateral diffuse infiltration. Doxycycline was prescribed for scrub typhus with acute respiratory distress syndrome and multiple organ failure. Fever subsided dramatically the next day and he was discharged on day 7 with oral tetracycline for 7 days.
CONCLUSION: Scrub typhus should be considered in acutely febrile patients with multiple organ involvement, particularly if there is an eschar or a history of environmental exposure in endemic areas. Rapid and accurate diagnosis, timely administration of antibiotics and intensive supportive care are necessary to decrease mortality of serious complications of scrub typhus.

DOI: 10.1186/1471-2334-11-79
PMCID: PMC3079651
PMID: 21450057 [PubMed - indexed for MEDLINE]

207. Am J Trop Med Hyg. 2014 Mar;90(3):507-10. doi: 10.4269/ajtmh.13-0511. Epub 2014 Jan 20.

Unusual genotypic distribution of Orientia tsutsugamushi strains causing human infections on Jeju Island.

Yoo JR(1), Heo ST, Koh YS, Kim S, Kim S.

Author information:
(1)Division of Infectious Disease, Samsung Medical Center, Sungkynkwan University School of Medicine, Seoul, Republic of Korea; Departments of Internal Medicine, Microbiology, and Immunology, Jeju National University School of Medicine, Jeju, Republic of Korea; Department of Internal Medicine, Seogwipo Medical Center, Jeju, Republic of Korea.

We investigated the clinical characteristics and serologic types of tsutsugamushi disease on the largest island of South Korea. There were 141 patients with tsutsugamushi disease at Jeju National University Hospital and Seogwipo Medical Center between November of 2003 and December of 2012. Median age of patients was 61 years, and 59% were women. The major clinical manifestations were fever (80.5%) and skin rash (55.7%), with eschars evident in 75.8% of the patients. Genotype analysis of Orientia tsutsugamushi was conducted in 33 specimens. The genotype was identified as Boryong type in 17 of 33 patients and Taguchi type in 15 of 33 patients. In our study, although the Taguchi genotype is rarely reported in the endemic area, it was common on Jeju Island. This genotype may be associated with mild and moderate forms of tsutsugamushi disease.

DOI: 10.4269/ajtmh.13-0511
PMCID: PMC3945696
PMID: 24445200 [PubMed - indexed for MEDLINE]

208. Clin Microbiol Infect. 2010 May;16(5):447-51. doi: 10.1111/j.1469-0691.2009.02865.x. Epub 2009 Sep 23.

A serosurvey of Orientia tsutsugamushi from patients with scrub typhus.

Kim DM(1), Lee YM, Back JH, Yang TY, Lee JH, Song HJ, Shim SK, Hwang KJ, Park MY.

Author information:
(1)Departments of Internal Medicine and Research Center for Resistant Cells, School of Medicine, National Institute of Health, Republic of Korea.

Many countries where scrub typhus is endemic use their own cutoff values for antibody titres to differentiate between cured cases and current infections. To establish an antibody titre cutoff value, one needs to investigate the seroprevalence in endemic areas, and the duration of the increase in titre after complete cure. We conducted a follow-up study of anti-Orientia tsutsugamushi antibody titres using indirect immunofluorescence assays (IFA) and passive haemagglutination assays (PHA) in patients with scrub typhus. After the onset of symptoms, IgM antibody titres increased gradually over 2-3 weeks, peaked at about 4 weeks, and started to decrease rapidly between 4 and 5 weeks. At 1-year follow-up, the median IgM value was 1:10. Out of 77 patients who were tested at that time, 36 (47%) had IgM titres > or =1:20, and none had titres exceeding 1:80. Over the first 2 weeks, IgG antibody titres increased sharply, peaked at about 4 weeks and decreased rather gradually thereafter, with a median titre of 1:128 maintained up to the 18th month. At 1-year follow-up, five out of 77 patients (6.5%) had titres > or =1:1,024 and 57% had titres > or =1:128. Based on these results, a cutoff value of > or =1:160 for IgM antibody should differentiate between previous and current infections in endemic areas such as Korea and Japan, where scrub typhus occurs mainly in the autumn.

DOI: 10.1111/j.1469-0691.2009.02865.x
PMID: 19778303 [PubMed - indexed for MEDLINE]

209. Parasit Vectors. 2013 Oct 29;6(1):312. doi: 10.1186/1756-3305-6-312.

Molecular epidemiology of Orientia tsutsugamushi in chiggers and ticks from domestic rodents in Shandong, northern China.

Zhang M, Zhao ZT(1), Yang HL, Zhang AH, Xu XQ, Meng XP, Zhang HY, Wang XJ, Li Z, Ding SJ, Yang L, Zhang LY.

Author information:
(1)Department of Epidemiology and Health Statistics, School of Public Health, Shandong University, Jinan, China. ztzhao@sdu.edu.cn.

BACKGROUND: Scrub typhus is endemic to a 13,000,000-km² area of the Asia-Pacific region, and causes an annual incidence of 1 million people. The mortality rate of scrub typhus ranges from 6.1% to 25% in Southeast Asia. Natural infection of Orientia tsutsugamushi has been identified in domestic rodents in Shandong Province. However, infestation of chiggers and ticks on the domestic rodents and prevalence and genotypes of O. tsutsugamushi in these Acarina remain unclear.
METHODS: During September 2010 to March 2012, 3134 chiggers and 89 ticks were collected from domestic rodents captured in three counties of Shandong Province. We amplified and sequenced the 56-kDa type-specific antigen gene of O. tsutsugamushi from DNA samples of these Acarina and designated to genotype according to sequence analysis.
RESULTS: Overall, the infestation rate of chiggers on domestic rodents was 17.0%, and the chigger index was 5.38. The infestation rate of ticks on rodents was

3.1%. Natural infection of O. tsutsugamushi was found in Leptotrombidium taishanicum, L. linhuaikongense, L. intermedium, L. scutellare, L. palpale, and Ixodes spp., the minimum positive rates of which were 5.9%, 3.2%, 1.2%, 0.8%, 0.8%, and 2.2%, respectively. Kawasaki-like genotypes were predominant in chiggers and ticks on domestic rodents, which were detected from L. taishanicum, L. intermedium, L. scutellare, L. palpale, and Ixodes spp. Shimokoshi-like genotype was detected from L. palpale.
CONCLUSIONS: In the present study we investigated the infestation of chiggers and ticks on domestic rodents in Shandong Province, and identified the prevalence and genotypes of O. tsutsugamushi in the Acarina. Infestation of vector chiggers in domestic rodents, prevalence of O. tsutsugamushi in infested chiggers, and high nucleotide homologies among the O. tsutsugamushi sequences from the Acarina, their animal hosts and scrub typhus patients, implied that domestic rodents may play an important role in the transmission of scrub typhus in Shandong, China. Further studies are needed to verify the vector significance of chiggers and ticks that tested positive for O. tsutsugamushi, and to assess the risk of human exposure to chiggers and ticks on domestic rodents.

DOI: 10.1186/1756-3305-6-312
PMCID: PMC3843596
PMID: 24499627 [PubMed - indexed for MEDLINE]

210. Korean J Intern Med. 2011 Dec;26(4):474-6. doi: 10.3904/kjim.2011.26.4.474. Epub 2011 Nov 28.

Two cases of scrub typhus presenting with Guillain-Barré syndrome with respiratory failure.

Ju IN, Lee JW, Cho SY, Ryu SJ, Kim YJ, Kim SI, Kang MW.

DOI: 10.3904/kjim.2011.26.4.474
PMCID: PMC3245400
PMID: 22205852 [PubMed - indexed for MEDLINE]

211. Zhonghua Liu Xing Bing Xue Za Zhi. 2014 Jan;35(1):88-92.

[Recent advances in molecular epidemiology of Orientia tsutsugamushi in China].

[Article in Chinese]

Zhang L(1), Bi Z(2), Zhao Z(3).

Author information:
(1)Department of Epidemiology and Health Statistics, School of Public Health, Shandong University, Jinan 250012, China. (2)Institute for Bacterial Infectious Disease Control and Prevention, Shandong Center for Disease Control and Prevention. (3)Department of Epidemiology and Health Statistics, School of Public Health, Shandong University, Jinan 250012, China. Email: ztzhao@sdu.edu.cn.

PMID: 24685046 [PubMed - indexed for MEDLINE]

212. Nihon Naika Gakkai Zasshi. 2012 Jan 10;101(1):164-7.

[Case report; November fever--the clinical features of scrub typhus by Leptotrombidium scutellare in south-central Fukushima Prefecture].

[Article in Japanese]

Narita M(1), Unuma N, Ito H, Sato N, Hoshino C, Inoue M, Yamamoto S, Ando S, Fujita H.

Author information:
(1)General Internal Medicine Department, Ohta Nishinouchi General Hospital, Japan.

PMID: 22413475 [PubMed - indexed for MEDLINE]

213. Korean J Intern Med. 2011 Sep;26(3):365. doi: 10.3904/kjim.2011.26.3.365. Epub 2011 Sep 13.

Unusually high number of eschars on the face of a patient with scrub typhus.

Hwang IT(1), Lee JH.

Author information:
(1)Department of Internal Medicine, Wonkwang University College of Medicine, Iksan, Korea.

DOI: 10.3904/kjim.2011.26.3.365
PMCID: PMC3192213
PMID: 22016601 [PubMed - indexed for MEDLINE]

214. J Postgrad Med. 2011 Jul-Sep;57(3):262-3. doi: 10.4103/0022-3859.85231.

Altered sensorium in scrub typhus.

Remalayam B, Viswanathan S, Muthu V, Mookappan S.

DOI: 10.4103/0022-3859.85231
PMID: 21941079 [PubMed - indexed for MEDLINE]

215. Vector Borne Zoonotic Dis. 2010 Mar;10(2):191-3. doi: 10.1089/vbz.2008.0199.

First report of an Orientia tsutsugamushi type TA716-related scrub typhus infection in Thailand.

McGready R(1), Blacksell SD, Luksameetanasan R, Wuthiekanun V, Jedsadapanpong W, Day NP, Nosten F.

Author information:

(1)Shoklo Malaria Research Unit, Tak, Thailand.

Orientia tsutsugamushi causes scrub typhus and is a rural zoonosis endemic in the Asia Pacific region. This is the first report of O. tsutsugamushi TA716-like strain in a human in Thailand. The patient was in the 1st trimester of pregnancy when she developed scrub typhus. The O. tsutsugamushi strain TA716 was detected from her admission blood sample, and the pregnancy ended in spontaneous abortion. The effects of scrub typhus in pregnant women and the pregnancy outcome are sparsely documented in the published medical literature. Improved clinical recognition and laboratory diagnosis will be essential to better define the morbidity caused by this zoonosis especially in pregnancy.

DOI: 10.1089/vbz.2008.0199
PMCID: PMC2924790
PMID: 19492945 [PubMed - indexed for MEDLINE]

216. Trop Doct. 2010 Oct;40(4):249-50. doi: 10.1258/td.2010.100056. Epub 2010 Jul 28.

Scrub typhus associated macrophage activation syndrome.

Gopal GK(1), Anugrah C, Boorugu H.

Author information:
(1)Department of Medicine - 3, Christian Medical College, Vellore, India. gops95@yahoo.com

Macrophage activation syndrome (MAS) is a rare phenomenon that occurs either primarily or secondary to a multitude of conditions, including juvenile rheumatoid arthritis most commonly, and other infections like enteric fever and tuberculosis. It has been reported as an extremely rare complication of scrub typhus with no cases presented from India. We report three cases of scrub typhus presenting with confirmed MAS between January 2007 and December 2007 to a tertiary care hospital in South India. All three patients had clinical and laboratory evidence for scrub typhus and MAS. All the patients responded promptly to antibiotics and made an uneventful recovery. These three patients are presented to highlight the importance of considering scrub typhus in patients with MAS following acute febrile illnesses.

DOI: 10.1258/td.2010.100056
PMID: 20667920 [PubMed - indexed for MEDLINE]

217. Trop Doct. 2010 Jul;40(3):129-33. doi: 10.1258/td.2010.090452. Epub 2010 Apr 1.

Scrub typhus: an unrecognized threat in South India - clinical profile and predictors of mortality.

Chrispal A(1), Boorugu H, Gopinath KG, Prakash JA, Chandy S, Abraham OC, Abraham AM, Thomas K.

Author information:
(1)Department of Medicine Unit 2, Christian Medical College, Vellore 632004, Tamil Nadu, India. anugrahchrispal@gmail.com

Scrub typhus is an important cause of acute undifferentiated febrile illnesses in the Indian subcontinent. Delay in diagnosis and in the initiation of appropriate treatment can result in severe complications such as acute respiratory distress syndrome (ARDS), septic shock and multisystem organ failure culminating in death. We conducted a prospective, observational study to delineate the clinical profile and predictors of mortality in scrub typhus in adults admitted to the medical wards of a tertiary care, referral hospital in South India over a one-year period. The case fatality rate in this study was 12.2%. Metabolic acidosis (odds ratio [OR] 6.1), ARDS (OR 3.6), altered sensorium (OR 3.6) and shock (OR 3.1) were independent predictors of mortality. It appears that scrub typhus has four possible overlapping clinical presentations: mild disease; respiratory predominant disease; central nervous system predominant disease (meningoencephalitis); or sepsis syndrome. Given the telltale presence of an eschar (evident in 45.5%), the characteristic clinical profile and the dramatic therapeutic response to a cheap, yet effective, drug such as doxycycline, medical practitioners in the region should have ample opportunity to reach an early diagnosis and initiate treatment which could, potentially, reduce the mortality and morbidity associated with scrub typhus.

DOI: 10.1258/td.2010.090452
PMID: 20360426 [PubMed - indexed for MEDLINE]

218. Lancet Glob Health. 2015 Feb;3(2):e104-12. doi: 10.1016/S2214-109X(14)70289-X.

Orientia, rickettsia, and leptospira pathogens as causes of CNS infections in Laos: a prospective study.

Dittrich S(1), Rattanavong S(2), Lee SJ(3), Panyanivong P(2), Craig SB(4), Tulsiani SM(5), Blacksell SD(3), Dance DA(1), Dubot-Pérès A(6), Sengduangphachanh A(2), Phoumin P(2), Paris DH(3), Newton PN(7).

Author information:
(1)Lao-Oxford-Mahosot Hospital-Wellcome Trust Research Unit (LOMWRU), Microbiology Laboratory, Mahosot Hospital, Vientiane, Lao PDR; Centre for Tropical Medicine and Global Health, Nuffield Department of Medicine, Churchill Hospital, University of Oxford, Oxford, UK. (2)Lao-Oxford-Mahosot Hospital-Wellcome Trust Research Unit (LOMWRU), Microbiology Laboratory, Mahosot Hospital, Vientiane, Lao PDR. (3)Centre for Tropical Medicine and Global Health, Nuffield Department of Medicine, Churchill Hospital, University of Oxford, Oxford, UK; Mahidol-Oxford Tropical Medicine Research Unit (MORU), Faculty of Tropical Medicine, Mahidol University, Bangkok, Thailand. (4)University of the Sunshine Coast, Faculty of Science Health, Education and Engineering, Sippy Downs, Australia; Queensland Health Forensic and Scientific Service, WHO Collaborating Centre for Reference and Research on Leptospirosis, Brisbane, Australia. (5)Queensland Health Forensic and Scientific Service, WHO Collaborating Centre for Reference and Research on Leptospirosis, Brisbane, Australia; Copenhagen Centre for Disaster Research Department of International Health, Immunology and Microbiology, University of Copenhagen, Copenhagen, Denmark. (6)Lao-Oxford-Mahosot Hospital-Wellcome Trust Research Unit (LOMWRU), Microbiology Laboratory, Mahosot Hospital, Vientiane, Lao PDR; Centre for Tropical Medicine and Global Health, Nuffield Department of Medicine, Churchill Hospital, University of Oxford, Oxford, UK; UMR_D 190 "Emergence des Pathologies

Virales", Aix-Marseille University, IRD French Institute of Research for Development, EHESP French School of Public Health, Marseille, France.
(7)Lao-Oxford-Mahosot Hospital-Wellcome Trust Research Unit (LOMWRU), Microbiology Laboratory, Mahosot Hospital, Vientiane, Lao PDR; Centre for Tropical Medicine and Global Health, Nuffield Department of Medicine, Churchill Hospital, University of Oxford, Oxford, UK. Electronic address: paul@tropmedres.ac.

Comment in
Lancet Glob Health. 2015 Feb;3(2):e67-8.

BACKGROUND: Scrub typhus (caused by Orientia tsutsugamushi), murine typhus (caused by Rickettsia typhi), and leptospirosis are common causes of febrile illness in Asia; meningitis and meningoencephalitis are severe complications. However, scarce data exist for the burden of these pathogens in patients with CNS disease in endemic countries. Laos is representative of vast economically poor rural areas in Asia with little medical information to guide public health policy. We assessed whether these pathogens are important causes of CNS infections in Laos.
METHODS: Between Jan 10, 2003, and Nov 25, 2011, we enrolled 1112 consecutive patients of all ages admitted with CNS symptoms or signs requiring a lumbar puncture at Mahosot Hospital, Vientiane, Laos. Microbiological examinations (culture, PCR, and serology) targeted so-called conventional bacterial infections (Streptococcus pneumoniae, Neisseria meningitidis, Haemophilus influenzae, S suis) and O tsutsugamushi, Rickettsia typhi/Rickettsia spp, and Leptospira spp infections in blood or cerebrospinal fluid (CSF). We analysed and compared causes and clinical and CSF characteristics between patient groups.
FINDINGS: 1051 (95%) of 1112 patients who presented had CSF available for analysis, of whom 254 (24%) had a CNS infection attributable to a bacterial or fungal pathogen. 90 (35%) of these 254 infections were caused by O tsutsugamushi, R typhi/Rickettsia spp, or Leptospira spp. These pathogens were significantly more frequent than conventional bacterial infections (90/1051 [9%] vs 42/1051 [4%]; p<0·0001) by use of conservative diagnostic definitions. CNS infections had a high mortality (236/876 [27%]), with 18% (13/71) for R typhi/Rickettsia spp, O tsutsugamushi, and Leptospira spp combined, and 33% (13/39) for conventional bacterial infections (p=0·076).
INTERPRETATION: Our data suggest that R typhi/Rickettsia spp, O tsutsugamushi, and Leptospira spp infections are important causes of CNS infections in Laos. Antibiotics, such as tetracyclines, needed for the treatment of murine typhus and scrub typhus, are not routinely advised for empirical treatment of CNS infections. These severely neglected infections represent a potentially large proportion of treatable CNS disease burden across vast endemic areas and need more attention.
FUNDING: Wellcome Trust UK.

Copyright © 2015 Dittrich et al. Open Access article published under the terms of CC BY. Published by .. All rights reserved.

DOI: 10.1016/S2214-109X(14)70289-X
PMCID: PMC4547322
PMID: 25617190 [PubMed - indexed for MEDLINE]

219. Trop Doct. 2010 Jul;40(3):149-51. doi: 10.1258/td.2010.090508.

Scrub typhus in Southern India: are we doing enough?

Razak A(1), Sathyanarayanan V, Prabhu M, Sangar M, Balasubramanian R.

Author information:
(1)Department of Internal Medicine, Kasturba Medical College, Manipal, Karnataka, India.

Scrub typhus is a potentially fatal infectious disease caused by Orientia tsutsugamushi. We report on a retrospective analysis of clinical manifestations, lab investigations and outcome of patients admitted to our hospital from January 2009 to December 2009 with scrub typhus. The Weil-Felix test was the serological method used to diagnose scrub typhus. A total of 29 patients were diagnosed with scrub typhus and the majority of them presented with fever (100%), headache (51%), myalgia (51%) and gastrointestinal symptoms (44%) as predominant features. Six patients (20%) presented with altered sensorium and signs of meningial irritation. There is an emergence of rickettsial infection in southern India, posing diagnostic difficulty and delay in treatment. Early diagnosis and treatment are important as it prevents serious complications associated with the disease.

DOI: 10.1258/td.2010.090508
PMID: 20555045 [PubMed - indexed for MEDLINE]

220. J Assoc Physicians India. 2011 Sep;59:602.

Gastro-intestinal bleeding in scrub typhus.

Mahajan SK.

PMID: 22334982 [PubMed - indexed for MEDLINE]

221. Indian Pediatr. 2011 Dec;48(12):991.

Persistent thrombocytopenia due to Scrub Typhus.

Kumar KJ, Manjunath VG.

PMID: 22253168 [PubMed - indexed for MEDLINE]

222. BMC Infect Dis. 2014 Dec 20;14:3864. doi: 10.1186/s12879-014-0688-8.

Rickettsial infection among military personnel deployed in Northern Sri Lanka.

Premaratna R(1), Ariyaratna N(2), Attanayake C(3), Bandara W(4), Chandrasena N(5), de Silva HJ(6).

Author information:

(1)Departments of Medicine, Faculty of Medicine, University of Kelaniya, Ragama, Sri Lanka. ranjanp64@gmail.com. (2)Military Hospital, Colombo, Sri Lanka. nimalkaa@yahoo.com. (3)Military Hospital, Palali, Sri Lanka. champaka_attanayaka@yahoo.com. (4)Department of Microbiology, Faculty of Medicine, University of Kelaniya, Ragama, Sri Lanka. bandarankb@gmail.com. (5)Department of Parasitology, Faculty of Medicine, University of Kelaniya, Ragama, Sri Lanka. nilminicha@gmail.com. (6)Departments of Medicine, Faculty of Medicine, University of Kelaniya, Ragama, Sri Lanka. hjanakadesilva@gmail.com.

BACKGROUND: Military personnel deployed in field actvities report on frequent tick bites. Therefore they may run the risk of exposure to rickettsial organisms.
METHODS: In order to assess the risk of exposure to rickettsial organisms, two groups of military personnel who were deployed in field activities of Nothern Sri Lanka were investigated. The first group was studied in order to assess the sero-prevalence of rickettsioses and consisted of soldiers who were admitted following injuries during field activities. The second group was studied to identify the incidence of acute rickettsioses during their acute febrile presentations. They were tested with IFA-IgG against spotted fever group rickettsioses (SFG), scrub typhus (ST) and murine typhus.
RESULTS: In the first group, 48/57 (84%) military personnel had serological evidence of exposure to rickettsioses (in all, IFA-IgG titer ≥ 1:128): 33/50 (66%) to SFG rickettsioses, 1/50 (2%) to ST and 14/50 (28%) had mixed titers for both (in all, titers were higher for SFG). While all of them were in military uniform most of the time and frequently slept on scrub land, 35/57 (61.4%) had never used insect repellents and none were on doxycycline prophylaxis. 48/57 (84%) had experienced tick bites during field activity. In the second group, there were 49 who presented with acute febrile illness with a mean duration of 8.5 days (SD 3.2). 33/49 (67.3%) were serologically positive for acute rickettsioses (IgG ≥1:256); 26 (79%) due to ST and 7 (21%) due to SFG rickettsioses,
CONCLUSIONS: Exposure to rickettsial disease was common among soldiers who were deployed in Northern Sri Lanka. Scrub typhus was the predominent species accounting for acute febrile illness. Further studies are needed to understand the reasons for very high sero-prevalence for SFG rickettsioses with no anticedent febrile illness. Use of preventive measures was not satisfactory. The high sero-prevelence of SFG rickettsioses is likely to interfere with serological diagnosis of acute SFG rickettsioses in this population.

DOI: 10.1186/s12879-014-0688-8
PMCID: PMC4335424
PMID: 25527099 [PubMed - indexed for MEDLINE]

223. Parasit Vectors. 2016 Jan 27;9:45. doi: 10.1186/s13071-016-1318-7.

Prevalence of antibodies against Ehrlichia spp. and Orientia tsutsugamushi in small mammals around harbors in Taiwan.

Tsai KH(1,)(2), Chang SF(3), Yen TY(4), Shih WL(5,)(6), Chen WJ(7), Wang HC(8), Yu XJ(9), Wen TH(10), Wu WJ(11), Shu PY(12).

Author information:
(1)Institute of Environmental Health, College of Public Health, National Taiwan University, No. 17, Xu-Zhou Road, Taipei, 10055, Taiwan. kunhtsai@ntu.edu.tw.

(2)Department of Public Health, College of Public Health, National Taiwan University, No. 17, Xu-Zhou Road, Taipei, 10055, Taiwan. kunhtsai@ntu.edu.tw. (3)Center for Research, Diagnostics and Vaccine Development, Centers for Disease Control, Ministry of Health and Welfare, No.161, Kun-Yang Street, Taipei, 11561, Taiwan. vivi@cdc.gov.tw. (4)Institute of Epidemiology and Preventive Medicine, College of Public Health, National Taiwan University, No. 17, Xu-Zhou Road, Taipei, 10055, Taiwan. d00849002@ntu.edu.tw. (5)Institute of Epidemiology and Preventive Medicine, College of Public Health, National Taiwan University, No. 17, Xu-Zhou Road, Taipei, 10055, Taiwan. d92842003@ntu.edu.tw. (6)Infectious Diseases Research and Education Center, Ministry of Health and Welfare and National Taiwan University, No. 17, Xu-Zhou Road, Taipei, 10055, Taiwan. d92842003@ntu.edu.tw. (7)Infectious Diseases Research and Education Center, Ministry of Health and Welfare and National Taiwan University, No. 17, Xu-Zhou Road, Taipei, 10055, Taiwan. frog4125@yahoo.com.tw. (8)Center for Research, Diagnostics and Vaccine Development, Centers for Disease Control, Ministry of Health and Welfare, No.161, Kun-Yang Street, Taipei, 11561, Taiwan. sjwang@cdc.gov.tw. (9)Departmentof Pathology, University of Texas Medical Branch, Galveston, TX, USA. xuyu@utmb.edu. (10)Department of Geography, National Taiwan University, No. 1, Sec. 4, Roosevelt Road, Taipei, 10617, Taiwan. wenthung@ntu.edu.tw. (11)Department of Entomology, College of Bioresources and Agriculture, National Taiwan University, No. 1, Sec. 4, Roosevelt Road, Taipei, 10617, Taiwan. wuwj@ntu.edu.tw. (12)Center for Research, Diagnostics and Vaccine Development, Centers for Disease Control, Ministry of Health and Welfare, No.161, Kun-Yang Street, Taipei, 11561, Taiwan. pyshu@cdc.gov.tw.

BACKGROUND: Tick-borne ehrlichiosis and mite-borne scrub typhus represent important emerging zoonotic rickettsial diseases. Although scrub typhus has been recognized by the Taiwanese public health system, information on ehrlichial infections is scarce in Taiwan. In this study, the risk of spread of ectoparasites on rodents through aerial and marine transportation was assessed in international and domestic harbors. Here, we report the first systematic surveillance of seroprevalence against Ehrlichia spp. in small mammals on the main island of Taiwan.
METHODS: In total, 1648 small mammals were trapped from 8 international ports, 18 domestic fishing harbors, and 7 local public health centers around Taiwan from November 2004 to December 2008. Sera were analyzed using indirect immunofluorescence assays to detect IgG antibodies against Ehrlichia chaffeensis and Orientia tsutsugamushi. A serum titer of 1:80 was considered positive.
RESULTS: Antibodies against Ehrlichia spp. and O. tsutsugamushi were detected in 3.28% and 4.92% of small mammals active around harbors, respectively. The seropositive rate against Ehrlichia was higher in northern Taiwan from 2005 to 2008. However, O. tsutsugamushi infections increased in southern Taiwan during this period. The serological evidence of ehrlichial and O. tsutsugamushi infections in all international ports were included in the study. No significant differences were found among the seropositive rates of Ehrlichia spp. and O. tsutsugamushi in small mammals trapped between international and local harbors.
CONCLUSIONS: The overall prevalence of Ehrlichia spp. and O. tsutsugamushi infections in small mammals active around harbors was 3.28% and 4.92%, respectively. The results provided serological evidence supporting the potential risks of transporting pathogens through air and maritime traffic. This study highlights serious issues of the emergence and spread of rickettsial diseases in Taiwan. The incidence of human ehrlichiosis requires further investigation.

DOI: 10.1186/s13071-016-1318-7

PMCID: PMC4728797
PMID: 26817445 [PubMed - in process]

224. Intern Med. 2011;50(21):2675-7. Epub 2011 Nov 1.

Scrub typhus-induced serious gastric ulcer bleeding.

Hoshino C(1), Narita M, Yamabe A, Sekikawa Y, Ishihara K, Ikeda H, Satoh N, Inoue M.

Author information:
(1)General Internal Medicine, Ohta-Nishinouchi Hospital, Japan.
gim-hoshino@ohta.hp.or.jp

A 67-year-old woman presented with melena and general weakness. Upper gastrointestinal (GI) endoscopy revealed multiple ulcers and projectile bleeding in the stomach. She also complained of a 10-day history of a fever and was diagnosed with scrub typhus based on a positive result of the eschar polymerase chain reaction (PCR) testing. She fully recovered with endoscopic hemostasis, and administration of minocycline and omeprazole. In a patient with GI manifestations, scrub typhus, a condition with pathologically systemic vasculitis, should be considered in the possible background in endemic areas. The eschar PCR testing is a rapid and useful diagnostic tool to identify a specific strain.

PMID: 22041379 [PubMed - indexed for MEDLINE]

225. BMC Infect Dis. 2010 Apr 30;10:108. doi: 10.1186/1471-2334-10-108.

Clinical and laboratory findings associated with severe scrub typhus.

Kim DM(1), Kim SW, Choi SH, Yun NR.

Author information:
(1)Division of Infectious Diseases, Department of Internal Medicine, Chosun University School of Medicine, Gwangju City, Republic of Korea.

BACKGROUND: Scrub typhus is a mite-borne bacterial infection of humans caused by Orientia tsutsugamushi that causes a generalized vasculitis that may involve the tissues of any organ system. The aim of this study was to identify factors associated to severe complications from scrub typhus.
METHODS: We conducted this prospective, case-control study on scrub typhus patients who presented to the Department of Internal Medicine at Chosun University Hospital between September, 2004 and December, 2006. Cases were 89 scrub typhus patients with severe complications and controls were 119 scrub typhus patients without severe complications.
RESULTS: There were significant differences in the absence of eschar, white blood cell (WBC) counts, hemoglobin, albumin, serum creatinine, fibrinogen, C-reactive protein (CRP), and active partial thromboplastin time (aPTT) between the two groups. Multivariate analysis demonstrated that only the following four factors were significantly associated with the severe complications of scrub typhus: (1)

age >or= 60 years (odd ratio [OR] = 3.13, P = 0.002, confidence interval [CI] = 1.53-6.41), (2) the absence of eschar (OR = 6.62, P = 0.03, CI = 1.22-35.8, (3) WBC counts > 10, 000/mm3 (OR = 3.6, P = 0.001, CI = 1.65-7.89), and (4) albumin <or= 3.0 g/dL (OR = 5.01, P = 0.004, CI = 1.69-14.86).
CONCLUSIONS: Our results suggest that clinicians should be aware of the potential for complications, when scrub typhus patients are older (>or= 60 years), presents without eschar, or laboratory findings such as WBC counts > 10, 000/mm3, and serum albumin level <or= 3.0 g/dL. Close observation and intensive care for scrub typhus patients with the potential for complications may prevent serious complications with subsequent reduction in its mortality rate.

DOI: 10.1186/1471-2334-10-108
PMCID: PMC2877676
PMID: 20433689 [PubMed - indexed for MEDLINE]

226. Am J Trop Med Hyg. 2016 Jul 6;95(1):43-9. doi: 10.4269/ajtmh.15-0752. Epub 2016 May 2.

Sennetsu Neorickettsiosis, Spotted Fever Group, and Typhus Group Rickettsioses in Three Provinces in Thailand.

Bhengsri S(1), Baggett HC(2), Edouard S(3), Dowell SF(4), Dasch GA(5), Fisk TL(6), Raoult D(3), Parola P(3).

Author information:
(1)Thailand Ministry of Public Health-U.S. Centers for Disease Control and Prevention Collaboration, Nonthaburi, Thailand. hpx4@cdc.gov. (2)Thailand Ministry of Public Health-U.S. Centers for Disease Control and Prevention Collaboration, Nonthaburi, Thailand. Division of Global Health Protection, Centers for Disease Control and Prevention, Atlanta, Georgia. (3)University Hospital Institute for Infectious Diseases and Tropical Medicine, National Reference Centre for Rickettsioses, Aix-Marseille University, Marseille, France. (4)Bill & Melinda Gates Foundation, Seattle, Washington. (5)Division of Vector-Borne Diseases, Centers for Disease Control and Prevention, Atlanta, Georgia. (6)Division of Infectious Disease, Emory University, Atlanta, Georgia.

We estimated the seroprevalence and determined the frequency of acute infections with Neorickettsia sennetsu, spotted fever group rickettsiae, Rickettsia typhi, and Orientia tsutsugamushi among 2,225 febrile patients presenting to community hospitals in three rural Thailand provinces during 2002-2005. The seroprevalence was 0.2% for sennetsu neorickettsiosis (SN), 0.8% for spotted fever group (SFG) rickettsiae, 4.2% for murine typhus (MT), and 4.2% for scrub typhus (ST). The frequency of acute infections was 0.1% for SN, 0.6% for SFG, 2.2% for MT, and 1.5% for ST. Additional studies to confirm the distribution of these pathogens and to identify animal reservoirs and transmission cycles are needed to understand the risk of infection.

© The American Society of Tropical Medicine and Hygiene.

DOI: 10.4269/ajtmh.15-0752
PMCID: PMC4944706
PMID: 27139448 [PubMed - in process]

227. J Clin Microbiol. 2010 Apr;48(4):1241-4. doi: 10.1128/JCM.01784-09. Epub 2010 Feb 3.

Scrub typhus in previously unrecognized areas of endemicity in China.

Zhang S(1), Song H, Liu Y, Li Q, Wang Y, Wu J, Wan J, Li G, Yu C, Li X, Yin W, Xu Z, Liu B, Zhang Q, Wan K, Li G, Fu X, Zhang J, He J, Hai R, Yu D, Walker DH, Xu J, Yu XJ.

Author information:
(1)State Key Lab for Infectious Diseases Prevention and Control, National Institute for Communicable Diseases Prevention and Control, China Centers for Disease Control and Prevention, Beijing 102206, China.

Scrub typhus, caused by Orientia tsutsugamushi, has emerged recently in areas of northern China where the disease had not been known to exist. We analyzed epidemiological, clinical, and laboratory data for 104 patients who were admitted to a hospital in Fuyang City between 26 September and 1 November 2008. We showed that the major clinical manifestations of the patients were fever (100%), headache (82%), myalgias (77%), eschar (67%), rash (52%), and unusual facial flushing (62%). Among the 104 patients, the sera of 98% contained IgM antibodies to O. tsutsugamushi detected by indirect immunofluorescence assays (IFA), and DNA of the O. tsutsugamushi 56-kDa gene was amplified by PCR from the blood of 36 patients. We conclude that 104 patients were infected with scrub typhus in Fuyang City, Anhui Province. Our study indicates that physicians need to consider the diagnosis of scrub typhus for febrile patients living in northern China, where scrub typhus had not been considered to exist in the past.

DOI: 10.1128/JCM.01784-09
PMCID: PMC2849583
PMID: 20129967 [PubMed - indexed for MEDLINE]

228. Pathology. 2011 Jan;43(1):83-4. doi: 10.1097/PAT.0b013e3283419df6.

Transient monoclonal gammopathy in a patient with scrub typhus.

Kwok JS, Cheung RC, Chow KM, Chan MH.

DOI: 10.1097/PAT.0b013e3283419df6
PMID: 21240076 [PubMed - indexed for MEDLINE]

229. Am J Trop Med Hyg. 2013 Jun;88(6):1217-9. doi: 10.4269/ajtmh.12-0740. Epub 2013 Apr 15.

Tsutsugamushi disease caused by Shimokoshi-type Orientia tsutsugamushi: the first report in Western Japan.

Ikegaya S(1), Iwasaki H, Takada N, Yamamoto S, Ueda T.

Author information:
(1)Division of Hematology and Oncology, Division of Infection Control and Senior

Fellow Laboratory, University of Fukui, Fukui, Japan. sikegaya@u-fukui.ac.jp

An 85-year-old female farmer was admitted to our hospital for fever, general fatigue, and skin rash. Cephalosporin was not effective and minocycline was dramatically effective. An eschar was discovered on her inguinal region after the defervescence. Laboratory examination of serum taken 12 days after onset of the illness showed elevated titers of antibodies against the Shimokoshi strain of Orientia tsutsugamushi. The gene sequence analysis of specimen from the patient's eschar revealed high similarity to the Shimokoshi strain by nested polymerase chain reaction. Therefore, this patient was diagnosed as a case of Shimokoshi-type tsutsugamushi disease, which has not previously been reported in Western Japan. Recently, cases of this type have also been confirmed in northeastern Japan, suggesting the need for further epidemiological studies.

DOI: 10.4269/ajtmh.12-0740
PMCID: PMC3752827
PMID: 23589529 [PubMed - indexed for MEDLINE]

230. Osong Public Health Res Perspect. 2010 Dec;1(1):55-60. doi: 10.1016/j.phrp.2010.12.012. Epub 2010 Dec 7.

Epidemiological characteristics of scrub typhus in Korea, 2009.

Kim S(1), Kim JS, Lee H.

Author information:
(1)Division of Epidemic Intelligence Service, Korea Centers for Disease Control and Prevention, Seoul, Korea.

OBJECTIVES: Currently, the incidence of scrub typhus has increased in urban areas. In this study, we described the epidemiological characteristics of scrub typhus cases reported in the urban areas of Korea in 2009.
METHODS: We analysed the case investigation reports of scrub typhus cases that were collected in Korea in 2009. Specially, the different risk factors such as fieldwork and outdoor activity were compared to urban and rural areas, and six urban cities. Statistical analysis was performed using $\chi(2)$ test.
RESULTS: A total of 4,461 cases (including 1,663 suspected cases) were analysed in this study. Among these, the case reports of 4,254 cases had complete addresses. The cases with outdoor activities were 720 (85.2%) in urban areas. In Daegu and Daejeon, the number of cases participated in outdoor activities was 32 (34.4%) and 23 (31.5%), respectively. In other urban areas, cases with outdoor activities were more than 85%.
CONCLUSION: The most common infection risk factor was outdoor activity in urban areas. However, the proportion and distribution of outdoor activities were different in urban areas. These results will be used to establish strategies for effective prevention and management in urban areas.

DOI: 10.1016/j.phrp.2010.12.012
PMCID: PMC3766886
PMID: 24159441 [PubMed]

231. Clin Neurol Neurosurg. 2011 Apr;113(3):250-3. doi:

10.1016/j.clineuro.2010.11.007. Epub 2010 Dec 3.

Scrub typhus meningo-encephalitis with focal neurologic signs and associated brain MRI abnormal findings: literature review.

Yum KS(1), Na SJ, Lee KO, Ko JH.

Author information:
(1)Department of Neurology, Konyang University College of Medicine, 685 Gasuwon-dong, Seo-gu, Daejeon-city 302-718, Republic of Korea.

DOI: 10.1016/j.clineuro.2010.11.007
PMID: 21129847 [PubMed - indexed for MEDLINE]

232. Int J Environ Res Public Health. 2014 Aug 20;11(8):8542-51. doi: 10.3390/ijerph110808542.

Human leptospirosis trends: northeast Thailand, 2001-2012.

Thipmontree W(1), Suputtamongkol Y(2), Tantibhedhyangkul W(3), Suttinont C(4), Wongswat E(5), Silpasakorn S(6).

Author information:
(1)Maharat Nakhon Ratchasima Hospital, Nakhon Ratchasima Province 30000, Thailand. noonmed@yahoo.com. (2)Department of Medicine, Faculty of Medicine Siriraj Hospital, Mahidol University, Bangkok 10700, Thailand. yupin.sup@mahidol.ac.th. (3)Department of Medicine, Faculty of Medicine Siriraj Hospital, Mahidol University, Bangkok 10700, Thailand. wiwit167@yahoo.co.th. (4)Maharat Nakhon Ratchasima Hospital, Nakhon Ratchasima Province 30000, Thailand. Chuanpit_s@hotmail.com. (5)Department of Medicine, Faculty of Medicine Siriraj Hospital, Mahidol University, Bangkok 10700, Thailand. Dave_bio4@hotmail.com. (6)Department of Medicine, Faculty of Medicine Siriraj Hospital, Mahidol University, Bangkok 10700, Thailand. saowaluk_8@yahoo.com.

The objective of this study was to determine the changing trend of leptospirosis over time in Thailand using two prospective hospital-based studies conducted amongst adult patients with acute undifferentiated fever (AUFI) admitted to Maharat Nakhon Ratchasima Hospital, Nakhon Ratchasima Province, Thailand between July 2001 to December 2002 and between July 2011 to December 2012. During the first period, leptospirosis (98 patients, 40%) and scrub typhus (59 patients, 24.1%) were the two major causes of AUFI. In the second period, scrub typhus (137 patients, 28.3%) was found to be more common than leptospirosis (61 patients, 12.7%). Amongst patients with leptospirosis, the proportion of male patients and the median age were similar. Leptospira interrogans serogroup Autumnalis was the major infecting serogroup in both study periods. The case fatality rate of leptospirosis was significantly higher in 2011-2012 as compared with the case fatality rate in 2001-2002 (19.7% vs. 6.3%, $p < 0.001$). In summary, we found that number of leptospirosis cases had decreased over time. This trend is similar to reportable data for leptospirosis complied from passive surveillance by the Ministry of Public Health, Thailand. However, the case fatality rate of severe leptospirosis has increased. Severe lung hemorrhage associated with leptospirosis remained the major cause of death.

DOI: 10.3390/ijerph110808542
PMCID: PMC4143876
PMID: 25141000 [PubMed - indexed for MEDLINE]

233. Clin Microbiol Infect. 2010 May;16(5):419-24. doi:
10.1111/j.1469-0691.2009.02825.x. Epub 2009 Jul 14.

Scrub typhus in patients with liver cirrhosis: a preliminary study.

Kim IH(1), Lee HB, Hwang JH, Kwon KS, Lee CS.

Author information:
(1)Department of Internal Medicine, Chonbuk National University Medical School,
Jeonju, Korea.

Scrub typhus is a potentially fatal infectious disease caused by the organism Orientia tsutsugamushi. However, to date, there have been no clearly identified determinants or reports published on the clinical severity of scrub typhus in liver cirrhosis (LC) patients. This study was carried out by retrospectively reviewing medical records accumulated over 7 years at a tertiary hospital. Sixteen patients of 160 had underlying LC, and they were defined as 'cases'; those without underlying LC were defined as 'controls'. The duration of hospital stay (23.0 +/- 24.8 days for cases and 6.8 +/- 7.0 days for controls, p 0.020) and APACHE II scores (14.1 +/- 6.0 for cases and 7.2 +/- 4.6 for controls, p <0.001) were, respectively, significantly longer and significantly higher on admission in the cases than in the controls. Surprisingly, hospital mortality was significantly higher in the cases than in the controls (31.3% and 3.5%, respectively, p 0.001). Among the LC group, the highest Model for End-stage Liver Disease (MELD) score during hospitalization (MELD-Peak) (p 0.024) and the lowest blood sodium concentration during hospitalization (MELD-Na-Lo) (p 0.003) were higher in fatal cases than in the survivors (MELD-Na-to). Physicians should be aware of an adverse relationship between LC and scrub typhus, and patients with LC should be advised to avoid exposure to O. tsutsugamushi, particularly in endemic areas and epidemic seasons.

DOI: 10.1111/j.1469-0691.2009.02825.x
PMID: 19624507 [PubMed - indexed for MEDLINE]

234. Korean J Intern Med. 2013 Nov;28(6):728-31. doi: 10.3904/kjim.2013.28.6.728. Epub
2013 Oct 29.

A case of Tsutsugamushi disease presenting with nephrotic syndrome.

Lee JH(1), Lee MJ, Shin DH, Kang SW, Choi KH, Yoo TH.

Author information:
(1)Division of Nephrology, Department of Internal Medicine, Yonsei University
College of Medicine, Seoul, Korea.

Nephrotic syndrome associated with Tsutsugamushi disease has not been previously reported. We are describing a case of Tsutsugamuchi disease presenting with nephrotic syndrome. A 72-year-old woman presented with fever and generalized

edema. Laboratory studies revealed a leukocytosis, hypoalbuminemia, and hypercholesterolemia. Her urine protein excretion was 5.4 g/day. The anti-Tsutsugamushi antibody test was strongly positive (1:2,560). A renal biopsy was performed, and pathologic findings revealed membranous glomerulonephritis. The patient's clinical symptoms improved markedly after treatment with doxycycline.

DOI: 10.3904/kjim.2013.28.6.728
PMCID: PMC3847001
PMID: 24307851 [PubMed - indexed for MEDLINE]

235. Trop Doct. 2010 Jul;40(3):188-90. doi: 10.1258/td.2010.090447.

Scrub typhus in Uttarakhand, India: a common rickettsial disease in an uncommon geographical region.

Ahmad S(1), Srivastava S, Verma SK, Puri P, Shirazi N.

Author information:
(1)Department of Medicine, Himalayan Institute of Medical Sciences, HIHT University, Swami Ram Nagar, Jolly Grant, PO Doiwala, Dehradun-248140, Uttarakhand, India. sohadia@hotmail.com

Scrub typhus is a commonly encountered rickettsial disease of the Indian subcontinent. Humans are infected accidentally and the case fatality can be significantly high if the disease is not identified in time. We report nine cases of scrub typhus from the Garhwal region of the newly created north Indian state of Uttarakhand, a region not previously known to harbour the vector. Entomological studies are needed to study the density of the vector and to institute vector control measures in order to prevent this relatively benign, yet potentially fatal, clinical entity from spiralling into a major public health issue.

DOI: 10.1258/td.2010.090447
PMID: 20555054 [PubMed - indexed for MEDLINE]

236. CMAJ. 2011 Oct 18;183(15):E1152. doi: 10.1503/cmaj.101929. Epub 2011 Sep 12.

Eschar: a clue to scrub typhus.

Shiao CC(1), Lin SY.

Author information:
(1)Department of Internal Medicine, Saint Mary's Hospital, Luodong, Taiwan. chungyy2001@yahoo.com.tw

DOI: 10.1503/cmaj.101929
PMCID: PMC3193135
PMID: 21911554 [PubMed - indexed for MEDLINE]

237. Emerg Infect Dis. 2010 Oct;16(10):1641-3. doi: 10.3201/eid1610.100456.

Scrub typhus involving central nervous system, India, 2004-2006.

Mahajan SK, Rolain JM, Kanga A, Raoult D.

DOI: 10.3201/eid1610.100456
PMCID: PMC3294396
PMID: 20875303 [PubMed - indexed for MEDLINE]

238. QJM. 2011 Jun;104(6):537-8. doi: 10.1093/qjmed/hcq073. Epub 2010 May 13.

Scrub typhus pneumonitis.

Chen CH(1), Liao WC, Tu CY.

Author information:
(1)Division of Pulmonary and Critical Care Medicine, Department of Internal Medicine, China Medical University Hospital, Taichung, Taiwan.

DOI: 10.1093/qjmed/hcq073
PMID: 20466757 [PubMed - indexed for MEDLINE]

239. Transfusion. 2010 Feb;50(2):467-70. doi: 10.1111/j.1537-2995.2009.02442.x. Epub 2009 Oct 15.

Scrub typhus induced by peripheral blood stem cell transplantation in the immunocompromised patient: diagnostic usefulness of nested polymerase chain reaction.

Kang SJ(1), Park KH, Jung SI, Jang HC, Ji SY, Ahn JS, Kim HJ, Shin JH, Kim DM.

Author information:
(1)Department of Internal Medicine, Chonnam National University Medical School, Gwangju, Korea.

Scrub typhus (Orientia tsutsugamushi) is a Gram-negative rickettsial disease in parts of Asia, transmitted from wild rodents to human by mites. This is a case report of scrub typhus contraction in an acute leukemia patient by transfusion of peripheral blood stem cells collected during the incubation period. Although human-to-human transmission of scrub typhus by needle-stick injury or transplacental transmission has previously been reported, this is the first case confirmed by polymerase chain reaction (PCR) and DNA sequencing. This type of incident shows the need to heighten awareness of the threat of rickettsial agents in transfused blood. Nested PCR is a useful diagnostic method to confirm the diagnosis during incubation period and in the early phase of disease, especially for immunocompromised patients.

DOI: 10.1111/j.1537-2995.2009.02442.x
PMID: 19843286 [PubMed - indexed for MEDLINE]

240. Zhonghua Liu Xing Bing Xue Za Zhi. 2011 Apr;32(4):419-23.

[Current epidemic status and issues on prevention and control of scrub typhus].

[Article in Chinese]

Zhang M(1), Wang XJ, Zhao ZT.

Author information:
(1)Department of Epidemiology and Health Statistics, School of Public Health, Shandong University, Jinan 250012, China.

PMID: 21569679 [PubMed - indexed for MEDLINE]

241. J AAPOS. 2010 Oct;14(5):460-1. doi: 10.1016/j.jaapos.2010.06.016.

Isolated abducens nerve palsy in a patient with scrub typhus.

Lee YH(1), Yun YJ, Jeong SH.

Author information:
(1)Department of Ophthalmology, Chungnam National University Hospital, Daejeon, Korea. opticalyh@hanmail.net, ieye@cnuh.co.kr

Abducens nerve palsies associated with infectious diseases are infrequent. Scrub typhus is an acute, febrile, infectious illness caused by Orientia tsutsugamushi (formerly Rickettsia tsutsugamushi), an obligate-intracellular gram-negative bacterium. The organism has been reported to be capable of entering the nervous system, causing meningitis and focal neurologic abnormalities. We report a case of isolated abducens nerve palsy associated with a scrub typhus infection.

Copyright © 2010 American Association for Pediatric Ophthalmology and Strabismus. Published by Mosby, Inc. All rights reserved.

DOI: 10.1016/j.jaapos.2010.06.016
PMID: 21035078 [PubMed - indexed for MEDLINE]

242. Zoonoses Public Health. 2013 Nov;60(7):487-93. doi: 10.1111/zph.12025. Epub 2012 Dec 31.

Epidemiological and clinical features of three clustered cases co-infected with Lyme disease and rickettsioses.

Xuefei D(1), Qin H, Xiaodi G, Zhen G, Wei L, Xuexia H, Jiazhen G, Xiuping F, Meimei T, Jingshan Z, Yunru L, Xiaoling F, Kanglin W, Xingwang L.

Author information:
(1)Beijing Ditan Hospital, Capital Medical University, Beijing, China State Key Laboratory for Infectious Disease Prevention and Control, National Institute for Communicable Disease Control and Prevention, Chinese Center for Disease Control and Prevention, Beijing, China.

Lyme disease and rickettsioses are two common diseases in China. However, the concomitant occurrence of both diseases in a single individual has been reported infrequently in literature. We reported three related female patients admitted at Beijing Ditan Hospital from October to December 2010. They had similar epidemiological histories. At the beginning, they only got a single diagnosis, respectively, but after specific screenings, the final diagnoses were made. Because arthropods can harbour more than one disease-causing agent, patients can be infected with more than one pathogen at the same time, so the possibility of co-infection could be higher than what was thought previously. These observations suggested that clinicians should enhance the complete screening of arthropod-related infectious diseases so as to make an accurate diagnosis and to avoid diagnostic errors.

© 2012 Blackwell Verlag GmbH.

DOI: 10.1111/zph.12025
PMID: 23280084 [PubMed - indexed for MEDLINE]

243. Southeast Asian J Trop Med Public Health. 2007 May;38(3):482-6.

Follow-up analysis on the epidemic strains of Orientia tsutsugamushi in the first outbreak of scrub typhus in Henan Province, China.

Zhang L(1), Jin Z, Xia S, Zhang J, Li M, Fu X, Luan M.

Author information:
(1)Department of Rickettsiology, National Institute of Communicable Disease Control and Prevention, Chinese Center for Disease Control and Prevention, Beijing 102206, China. zhanglijuan@icdc.cn

To obtain knowledge of the genetic characteristics and types of the epidemic strains of Orientia tsutsugamushi in the first outbreak of scrub typhus in Henan Province, genus and type-specific primers were employed to amplify a fragment of the gene of 56 kDa protein. Serotyping demonstrated that, of the 19 patients [15 patients in recovery phase (10-40 days) and 4 of patients in acute phase (1-7 days)], 4 were infected with Gilliam type, 8 with Kato type, 6 with Karp type, and 1 with an unknown type. Successful genotyping was obtained for only 3 patients, indicating that 2 were infected with Karp type and 1 with Taiwan Kato type. Thus the outbreak of scrub typhus in Henan Province was caused by at least two epidemic strains.

PMID: 17877223 [PubMed - indexed for MEDLINE]

244. Nihon Kokyuki Gakkai Zasshi. 2010 Sep;48(9):706-10.

[A case of scrub typhus with lung involvement].

[Article in Japanese]

Takizawa H(1), Yamaguchi B, Hase I, Tasaka T, Ishii Y.

Author information:
(1)Department of Respiratory Medicine, Southern Tohoku Research Institute for Neuroscience, Southern Tohoku General Hospital.

A 65-year-old-woman complained of lumbago from the end of March 2008. Three weeks later, she visited a local clinic because of high fever, and she was given a diagnosis of urinary tract infection. Although levofloxacin was given, her condition did not improve and she was referred to the urology department of our hospital. Two days after hospitalization, she rapidly developed respiratory failure. Chest CT revealed bilateral pleural effusion, interlobular septal thickening, diffuse ground-glass opacities and mediastinal lymphadenopathy. We suspected scrub typhus because we noticed a localized necrotic skin lesion on her left lower leg. When minocycline was administered, both her clinical condition and radiographic imaging promptly improved. Because lung involvement with scrub typhus is very rare in Japan, we report this case of scrub typhus with various lung findings.

PMID: 20954375 [PubMed - indexed for MEDLINE]

245. BMC Infect Dis. 2010 Jul 21;10:216. doi: 10.1186/1471-2334-10-216.

Clinical significance of hypoalbuminemia in outcome of patients with scrub typhus.

Lee CS(1), Min IS, Hwang JH, Kwon KS, Lee HB.

Author information:
(1)Department of Internal Medicine, Chonbuk National University Medical School and Research Institute of Clinical Medicine, Geumam-dong, Jeonju 561180, Republic of Korea. lcsmd@jbnu.ac.kr

BACKGROUND: This study was designed to investigate the clinical significance of hypoalbuminemia as a marker of severity and mortality in patients with Scrub typhus.
METHODS: The patients with scrub typhus were divided into two groups based on the serum albumin levels; Group I (serum albumin <3.0 g/dL) and Group II (serum albumin >or=3.0 g/dL). The outcome of patients with hypoalbuminemia was compared with that of normoalbuminemia.
RESULTS: Of the total 246 patients who underwent the study, 84 patients (34.1%) were categorized as Group I and 162 patients were (65.9%) as Group II. Group I showed significantly higher incidence of confusion (24.6% vs. 5.3%, p < 0.001), pulmonary edema (15.8% vs. 3.2%, p = 0.002), pleural effusion (22.8% vs. 11.1%, p = 0.03), arrhythmia (12.3% vs. 2.6%, p = 0.008) and non-oliguric acute renal failure (40.4% vs. 11.1%, p < 0.001) compared to group II. Hypoalbuminemic group had a higher APACHE II score (11.37 +/- 5.0 vs. 6.94 +/- 4.2, p < 0.001), longer hospital stay (19.9 +/- 42.1 days vs 7.5 +/- 13.8 days, p = 0.012), and higher hospital cost compared to Group II.
CONCLUSIONS: This study showed hypoalbuminemia in scrub typhus was closely related to the frequency of various complication, longer hospital stay, consequently the higher medical cost, necessitating more efficient management of patients, including medical resources.

DOI: 10.1186/1471-2334-10-216
PMCID: PMC2919547
PMID: 20646323 [PubMed - indexed for MEDLINE]

246. Clin Infect Dis. 2009 Mar 15;48 Suppl 3:S203-30. doi: 10.1086/596576.

Scrub typhus: the geographic distribution of phenotypic and genotypic variants of Orientia tsutsugamushi.

Kelly DJ(1), Fuerst PA, Ching WM, Richards AL.

Author information:
(1)Department of Evolution, Ecology, and Organismal Biology, The Ohio State University, Columbus, OH 43210, USA. Kelly.350@osu.edu

Orientia tsutsugamushi is the etiological agent of scrub typhus, an acute, mite-borne, febrile illness that occurs in the Asia-Pacific region. Historically, strain characterization used serological analysis and revealed dramatic antigenic diversity. Eyeing a recommendation of potential vaccine candidates for broad protection, we review geographic diversity and serological and DNA prevalences. DNA analysis together with immunological analysis suggest that the prototype Karp strain and closely related strains are the most common throughout the region of endemicity. According to serological analysis, approximately 50% of isolates are seroreactive to Karp antisera, and approximately one-quarter of isolates are seroreactive to antisera against the prototype Gilliam strain. Molecular methods reveal greater diversity. By molecular methods, strains phylogenetically similar to Karp make up approximately 40% of all genotyped isolates, followed by the JG genotype group (Japan strains serotypically similar to the Gilliam strain but genetically non-Gilliam; 18% of all genotyped isolates). Three other genotype groups (Kato-related, Kawasaki-like, and TA763-like) each represent approximately 10% of genotyped isolates. Strains genetically similar to the Gilliam strain make up only 5% of isolates. Strains from these groups should be included in any potential vaccine.

DOI: 10.1086/596576
PMID: 19220144 [PubMed - indexed for MEDLINE]

247. J Assoc Physicians India. 2010 Jan;58:24-8.

Outbreak of scrub typhus in Pondicherry.

Vivekanandan M(1), Mani A, Priya YS, Singh AP, Jayakumar S, Purty S.

Author information:
(1)Department of Medicine & Microbiology, Pondicherry Institute of Medical Sciences, Kalapet, Pondicherry - 605 014.

AIM: To describe the diverse clinical and laboratory manifestations of scrub typhus diagnosed in Pondicherry Institute of Medical Sciences, Pondicherry.
MATERIALS AND METHODS: All cases of febrile illness diagnosed as scrub typhus over a period of 2 years were analysed. Diagnosis was based on the presence of the eschar and/or positive Weil Felix test with a titre of > 1:80.

RESULTS: Fifty cases of scrub typhus were seen over a period of 2 years (April 2006 and April 2008). Common symptoms were high grade fever of 7-14 days duration, nausea, vomiting, headache, myalgia, cough and breathlessness. Eschar was seen in 23 cases (46%) and the common sites were axilla, breast and groin. Weil Felix test was positive in 39 cases (78%). Liver enzymes were elevated in nearly all cases (95.9%). Multiple Organ Dysfunction Syndrome (MODS) was present in one third of our patients (17 out of 50, 34%). Hypotension (8 patients, 16%), renal impairment (6 patients, 12%), ARDS (4 patients, 8%) and meningitis (7 patients, 14%) were some of the important complications. There was a dramatic response to doxycycline in nearly all the patients.
CONCLUSION: Scrub typhus has emerged as an important cause of febrile illness in Pondicherry. Empirical treatment with doxycycline is justified in endemic areas.

PMID: 20649095 [PubMed - indexed for MEDLINE]

248. Pediatr Infect Dis J. 2009 Dec;28(12):1111-4. doi: 10.1097/INF.0b013e3181af8287.

Characteristics of pediatric scrub typhus in a new endemic region of northern China.

Liu YX(1), Jia N, Suo JJ, Xing YB, Liu G, Xiao HJ, Jia N, Zhao ZT, Min JS, Feng PT, Ma SB, Richardus JH, Cao WC.

Author information:
(1)Chinese People's Liberation Army General Hospital, Department of Hospital Infection Control, Beijing, People's Republic of China.

BACKGROUND: Scrub typhus emerged in northern China in 1986. Our objective was to document epidemiologic, clinical and laboratory features of pediatric scrub typhus in this new endemic area.
METHODS: The pediatric patients diagnosed with scrub typhus during the 12-year period from 1995 through 2006 in Feixian County, Shandong province were enrolled in the study. The cases were diagnosed based on either specific antibody detection using the indirect immunofluorescent assay or detection of partial Orientia tsutsugamushi gene by polymerase chain reaction.
RESULTS: Seventy pediatric scrub typhus cases were included in the study. The cases occurred from September through November. The common clinical manifestations included headache (100%), skin rash (91%), eschar (84%), lymphadenopathy (61%), and gastrointestinal signs (56%). None of the patients had neurologic involvement or thrombocytopenia. All children responded well to treatment with chloramphenicol. The patients who presented with abnormal chest radiography took significantly longer time to defervescence ($Z[r] = 2.528$, $P = 0.011$). Three strains of O. tsutsugamushi were isolated and all were identified as Kawasaki type.
CONCLUSIONS: The manifestations of pediatric scrub typhus cases in the novel endemic region of northern China may be less severe than in other regions. Careful examination of skin eschars is helpful for the clinical diagnosis.

DOI: 10.1097/INF.0b013e3181af8287
PMID: 19935272 [PubMed - indexed for MEDLINE]

249. FEMS Immunol Med Microbiol. 2008 Apr;52(3):335-42. doi: 10.1111/j.1574-695X.2007.00375.x. Epub 2008 Feb 27.

Genetic typing of the 56-kDa type-specific antigen gene of contemporary Orientia tsutsugamushi isolates causing human scrub typhus at two sites in north-eastern and western Thailand.

Blacksell SD(1), Luksameetanasan R, Kalambaheti T, Aukkanit N, Paris DH, McGready R, Nosten F, Peacock SJ, Day NP.

Author information:
(1)Oxford Tropical Medicine Research Unit, Mahidol University, Bangkok, Thailand. stuart@tropmedres.ac

Orientia tsutsugamushi is the causative agent of scrub typhus, a major cause of febrile illness in the rural areas of Southeast Asia. Twenty-three strains of O. tsutsugamushi were isolated from patients with scrub typhus in north-east (Udorn Thani province) and western Thailand (Tak province) between 2003 and 2005. The isolates were characterized by sequencing the entire ORF of the 56-kDa-type-specific antigen gene, followed by phylogenetic analysis. The majority (15/23) of isolates clustered with the Karp-type strain, six with a Gilliam-type strain and one each with the TA716- and TA763-type strains. Overall, there was considerable diversity in sequence, comparable to that seen in strains from across the rest of the scrub typhus-endemic world. There was no significant difference in the distributions of strains between the two provinces (P=0.08, Fisher's exact) nor a temporal change in distribution with year of isolation (P=0.80, Fisher's exact). Within this diversity there were also examples of isolates with identical 56-kDa genotypes that were cultured from patients from the same geographical areas.

DOI: 10.1111/j.1574-695X.2007.00375.x
PMID: 18312580 [PubMed - indexed for MEDLINE]

250. J Postgrad Med. 2010 Oct-Dec;56(4):301-2. doi: 10.4103/0022-3859.70949.

Hemophagocytosis in scrub typhus.

Valsalan R(1), Kosaraju K, Sohanlal T, Kumar PS.

Author information:
(1)Division of Medicine, Lyell McEwin Hospital, South Australia, Australia. drrohithv@gmail.com

DOI: 10.4103/0022-3859.70949
PMID: 20935405 [PubMed - indexed for MEDLINE]

251. Vector Borne Zoonotic Dis. 2010 Mar;10(2):125-33. doi: 10.1089/vbz.2008.0123.

Serological surveillance of scrub typhus, murine typhus, and leptospirosis in small mammals captured at firing points 10 and 60, Gyeonggi province, Republic of Korea, 2001-2005.

O'Guinn ML(1), Klein TA, Lee JS, Richards AL, Kim HC, Ha SJ, Shim SH, Baek LJ, Song KJ, Chong ST, Turell MJ, Burkett DA, Schuster A, Lee IY, Yi SH, Sames WJ, Song JW.

Author information:
(1)Virology Division, U.S. Army Medical Research Institute of Infectious Diseases, Fort Detrick, Maryland, USA.

Soldiers from the Republic of Korea and the United States conducting peacetime military operations at various training sites and multiple range complexes located near the demilitarized zone separating North and South Korea are exposed to rodents and their potentially disease-carrying ectoparasites. These diseases include scrub typhus, murine typhus, and leptospirosis. Many of the training sites are rural or semi-rural, surrounded or co-located with various forms of agriculture, and are infested with rodents and insectivores (as well as their ectoparasites), which are commonly found in association with unmanaged tall grasses, scrub, and crawling vegetation habitats. For 5 years, rodents and insectivores were collected seasonally (spring, summer, fall, and winter) at firing points 10 and 60 near the demilitarized zone and serologically tested for the presence of scrub typhus, murine typhus, and leptospirosis antibodies. Of the nine species of small mammals collected, Apodemus agrarius, the common striped field mouse and known reservoir of scrub typhus, was the most frequently collected (90.6%). Only four of the nine species captured, A. agrarius (60.9%), Micromys minutus (100%), Mus musculus (55.6%), and Rattus norvegicus (46.7%), were positive for scrub typhus. Of all the small mammals captured, only A. agrarius was positive for murine typhus (0.3%) and leptospirosis (1.3%). Seasonal and annual prevalence rates based on weight and sex are presented.

DOI: 10.1089/vbz.2008.0123
PMID: 19402761 [PubMed - indexed for MEDLINE]

252. Am J Trop Med Hyg. 2005 Apr;72(4):458-64.

Induction of protective immunity against scrub typhus with a 56-kilodalton recombinant antigen fused with a 47-kilodalton antigen of Orientia tsutsugamushi Karp.

Yu Y(1), Wen B, Wen B, Niu D, Chen M, Qiu L.

Author information:
(1)Beijing Institute of Microbiology and Epidemiology, Fengtai, Beijing, People's Republic of China.

A partial gene sequence encoding the 56-kD scrub typhus antigen (Sta56) was amplified from genomic DNA of the Orientia tsutsugamushi Karp strain by a polymerase chain reaction (PCR). The PCR product was ligated with the 47-kD scrub typhus antigen (Sta47) gene in the pQE30/47 expression vector, and the resulting recombinant expression vector was designated pQE30/56-47. A fusion antigen (Sta56-47) was expressed in Escherichia coli cells transformed with pQE30/56-47 after induction with isopropyl-beta-d-thiogalactopyranoside. The Sta56-47 antigen was recognized by both Sta47 and Sta56 immune sera and by immune serum to Sta56-47 in an immunoblot assay. This antigen was purified and used to immunize BALB/c mice. The animals immunized with Sta56-47 exhibited profound humoral and

cellular immune responses, as well as increased resistance to O. tsutsugamushi Karp compared with mice immunized with Sta56 or Sta47. These results strongly suggest that Sta56-47 contains antigenic epitopes of the Sta56 and Sta47 antigens of O. tsutsugamushi Karp, and is a more suitable candidate for replacing whole-cell antigen of O. tsutsugamushi Karp to induce protective immunity against scrub typhus.

PMID: 15827286 [PubMed - indexed for MEDLINE]

253. Emerg Infect Dis. 2006 Jul;12(7):1109-12.

Orientia tsutsugamushi in eschars from scrub typhus patients.

Liu YX(1), Cao WC, Gao Y, Zhang JL, Yang ZQ, Zhao ZT, Foley JE.

Author information:
(1)Beijing Institute of Microbiology and Epidemiology, Beijing, People's Republic of China.

To verify the value of eschars for the diagnosis of scrub typhus and to characterize genotypes of Orientia tsutsugamushi in patients, we examined eschars and blood specimens of 7 patients from Shandong Province, People's Republic of China, for O. tsutsugamushi by polymerase chain reaction targeting the Sta56 gene. All 7 eschars and acute-phase blood samples were positive, while no specific DNA amplicons were obtained from the 7 convalescent-phase blood samples collected after antimicrobial drug therapy. The findings indicate that patients' eschars can be used for detection and genetic characterization of O. tsutsugamushi during the convalescent phase.

DOI: 10.3201/eid1207.050827
PMCID: PMC3375733
PMID: 16836828 [PubMed - indexed for MEDLINE]

254. Am J Trop Med Hyg. 2012 Apr;86(4):711-2. doi: 10.4269/ajtmh.2012.11-0424.

Detection of rickettsioses and Q fever in Sri Lanka.

Angelakis E(1), Munasinghe A, Yaddehige I, Liyanapathirana V, Thevanesam V, Bregliano A, Socolovschi C, Edouard S, Fournier PE, Raoult D, Parola P.

Author information:
(1)Université de la Méditerranée, URMITE UMR, CNRS-IRD, Faculté de Médecine et de Pharmacie, Marseille, France. angelotasmanos@msn.com

Current serological evidence suggests the presence of scrub typhus and spotted fever group (SFG) rickettsiosis in Sri Lanka. Our objective was to identify rickettsial agents/Q fever as aetiological causes for patients who were presumed having rickettsioses by the presence of an eschar or a rash. Sera from patients with unknown origin fever from Matara were tested by immunofluorescence for SFG rickettsial antigens, typhus group rickettsiae, Orientia tsutsugamushi, and Coxiella burnetii antigens. Thirteen (7.3%) of the patients presented with a

rash, 11 (6.1%) had an inoculation eschar, and 16 patients recalled a tick or flea bite. We found that 25 (14%) patients had scrub typhus, 6 (3%) SFG rickettsioses, 3 (1.6%) acute Q fever, 3 (1.6%) murine typhus, and 3 (1.6%) were infected by Rickettsia felis. In addition to already described scrub and murine typhus, we found that R. felis and C. burnetii infections should be considered in Sri Lanka.

DOI: 10.4269/ajtmh.2012.11-0424
PMCID: PMC3403782
PMID: 22492158 [PubMed - indexed for MEDLINE]

255. Korean J Parasitol. 2009 Dec;47(4):381-6. doi: 10.3347/kjp.2009.47.4.381. Epub 2009 Dec 1.

Geographical distribution and relative abundance of vectors of scrub typhus in the Republic of Korea.

Lee IY(1), Kim HC, Lee YS, Seo JH, Lim JW, Yong TS, Klein TA, Lee WJ.

Author information:
(1)Department of Environmental Medical Biology, Yonsei University College of Medicine, Seoul 120-752, Korea.

A survey to determine the geographical distribution and relative abundance of potential vectors of scrub typhus was conducted from October to November 2006 at 13 localities throughout the Republic of Korea. Apodemus agrarius accounted for 97.6% (80/82) of all rodents, while only 2 Myodes regulus (2/82) were collected. A total of 10,860 chiggers were collected from A. agrarius belonging to 4 genera and 8 species, while only Walchia fragilis (40) was collected from Myodes regulus. Leptotrombidium pallidum (8,137; 74.9%), a vector of scrub typhus, was the predominant species collected from A. agrarius followed by Leptotrombidium scutellare (2,057, 18.9%), Leptotrombidium palpale (279; 2.7%), Leptotrombidium orientale (232; 2.1%), and Leptotrombidium zetum (79; 0.7%), Neotrombicula tamiyai (58; 0.5%), Euschoengastica koreaensis (16; 0.1%), and Cheladonta ikaoensis (2; < 0.1%). L. pallidum was the predominant chigger collected at collection sites in Gangwon (100%), Gyeonggi (87.2%), Chungnam (100%), Chungbuk (100%), Jeonbuk (73.9%), Jeonnam (77.0%), and Gyeongbuk (66.1%) provinces, whereas L. scutellare was the predominant chigger collected in Gyeongnam province (77.9%) and Jeju Island (62.3%). Data suggest a correlation between chigger population abundance and human cases of scrub typhus in Korea.

DOI: 10.3347/kjp.2009.47.4.381
PMCID: PMC2788717
PMID: 19967086 [PubMed - indexed for MEDLINE]

256. J Assoc Physicians India. 2010 Aug;58:520.

Reporting a case of scrub typhus from Andhra Pradesh.

Boorugu H, Dinaker M, Roy ND, Jude JA.

PMID: 21189708 [PubMed - indexed for MEDLINE]

257. Mod Pathol. 2001 Aug;14(8):752-9.

Identification of the target cells of Orientia tsutsugamushi in human cases of scrub typhus.

Moron CG(1), Popov VL, Feng HM, Wear D, Walker DH.

Author information:
(1)Department of Pathology, University of Texas Medical Branch at Galveston, 301 University Blvd., Galveston, TX 77555, USA.

Orientia tsutsugamushi is the etiologic agent of scrub typhus, a chigger-borne zoonosis that is a highly prevalent, life-threatening illness of greatest public health importance in tropical Asia and the islands of the western Pacific Ocean. The target cell of this bacterium is poorly defined in humans. In this study, O. tsutsugamushi were identified by immunohistochemistry using a rabbit polyclonal antibody raised against O. tsutsugamushi Karp strain in paraffin-embedded archived autopsy tissues of three patients with clinical suspicion of scrub typhus who died during World War II and the Vietnam War. Rickettsiae were located in endothelial cells in all of the organs evaluated, namely heart, lung, brain, kidney, pancreas, and skin, and within cardiac muscle cells and in macrophages located in liver and spleen. Electron microscopy confirmed the location of rickettsiae in endothelium and cardiac myocytes.

DOI: 10.1038/modpathol.3880385
PMID: 11504834 [PubMed - indexed for MEDLINE]

258. Pediatr Infect Dis J. 2003 Apr;22(4):341-5.

Epidemiologic, clinical and laboratory features of scrub typhus in thirty Thai children.

Sirisanthana V(1), Puthanakit T, Sirisanthana T.

Author information:
(1)Faculty of Medicine, Chiang Mai University, Chiang Mai, Thailand.

BACKGROUND: Scrub typhus, a potentially fatal rickettsial infection, is common in Asia. Although serologic surveys suggested that as many as one-fourth of cases of scrub typhus might be in children, very few reports of childhood scrub typhus are available in the medical literature.
OBJECTIVES: To document the clinical, laboratory and epidemiologic characteristics of pediatric patients with scrub typhus.
METHODS: From January 1, 2000 to December 31, 2001, all pediatric patients at Chiang Mai University Hospital who had obscure fever for >5 days were tested for indirect immunofluorescent antibody (IFA) against Orientia tsutsugamushi, the causative organism of scrub typhus. Scrub typhus was diagnosed on the basis of either a single IFA titer against O. tsutsugamushi > or =1/400 or a 4-fold or greater rise in IFA titer to at least 1/200.
RESULTS: Thirty children with scrub typhus were enrolled. Most were diagnosed

during the rainy months of June to November. Common physical signs included lymphadenopathy (93%), hepatomegaly (73%), eschar (68%), conjunctival hyperemia (33%), maculopapular rash (30%) and splenomegaly (23%). Eleven patients had interstitial pneumonitis and 1 patient had meningitis. All patients responded well to doxycycline or chloramphenicol. The average interval to defervescence after treatment was 29 h (range, 6 to 72).
CONCLUSIONS: Clinical and epidemiologic features of 30 pediatric patients with scrub typhus are reported in a prospective study. The presence of eschar was helpful in making the diagnosis. Complications included pneumonitis and meningitis. All cases responded well to treatment with antibiotic.

DOI: 10.1097/01.inf.0000059400.23448.57
PMID: 12690274 [PubMed - indexed for MEDLINE]

259. Zhonghua Er Ke Za Zhi. 2008 Feb;46(2):128-31.

[Clinical manifestations and epidemic factors of autumn-winter type scrub typhus in children from northern new endemic area].

[Article in Chinese]

Liu YX(1), Zhao ZT, Feng PT, Ma SB, Min JS, Qin DT, Yuan YE, Wei H, Suo JJ, Xing YB, Jia N, Gao Y.

Author information:
(1)Department of Nosocomial Infection Management and Disease Control, Institute of Hospital Management, General Hospital of the People's Liberation Army, Beijing 100853, China.

OBJECTIVE: Scrub typhus is an infectious disease due to Orientia tsutsugamushi transmitted by infected chigger mites. Scrub typhus has long been recognized to occur in southern areas of China, but has recently been increasingly often reported from the north since the first case was reported in Mengyin County, Shandong Province in 1986. The key objectives of the present study were to investigate the clinical manifestations and epidemic factors of scrub typhus in children from the northern new natural foci.
METHODS: The case records of 56 children with scrub typhus who were admitted to the 5 hospitals of Fei County from September 1993 to January 2004 were reviewed. Orientia tsutsugamushi (Ot) was isolated from the cases. Based on ecological observations on the composition, seasonal fluctuation of animal hosts and chigger mites, Ot was isolated from rodents and chiggers. IgG antibodies to Ot was detected by IFA. Genotypes of the Ot isolates were also identified by nested PCR.
RESULTS: Among 56 children scrub typhus cases, 46 were male, 10 were female; 96% exhibited typical eschars or ulcers, 100% cases had high fever, skin rashes were observed in 55 cases (98%), and regional lymphadenopathy occurred in 48 cases (86%). All cases came from countryside, and all had histories of exposure to the crop field. fifty-one serum samples of suspected patients with scrub typhus were collected, 48 were positive for antibodies to Ot. The serotypes were Gilliam types. The cases only appeared in September to December with the peak at mid and late October. Leptotrombidium (L.) scutellare was the most important vector causing scrub typhus in the foci. Apodemus (A.) agrarius was the main host animals of Ot in the crop field. Totally 26 strains were isolated from patients, rodents, and chigger mites. The serotypes of 24 out of the 26 isolates were

Gilliam types, while the genotypes of these isolates were Kawasaki types. The serotypes of the other 2 isolates were identical and both were Karp types. CONCLUSION: Children scrub typhus patients were frequently seen in the new natural foci of Shandong province. Exposure history, typical eschars or ulcers, and presence of IgG antibody were the important indexes to diagnose the disease.

PMID: 19099688 [PubMed - indexed for MEDLINE]

260. P N G Med J. 2007 Sep-Dec;50(3-4):172-83.

Scrub typhus (Orientia tsutsugamushi), spotted fever (Rickettsia australis) and dengue fever as possible causes of mysterious deaths in the Strickland Gorge area of Southern Highlands and West Sepik Provinces of Papua New Guinea.

Spicer PE(1), Taufa T, Benjamin AL.

Author information:
(1)HMAS Cairns Naval Base, Australia.

A medical investigation was carried out in April 2001 into an outbreak of a mysterious haemorrhagic disease and deaths in the remote picturesque Strickland River area of Papua New Guinea (PNG). The area is in part of the Southern Highlands and West Sepik Provinces and situated downstream from the Porgera Joint Venture gold mine. 9 villages were visited and 140 persons, consisting of immediate blood relatives of the deceased (cases) and others in the village picked at random (controls), were physically examined. Specimens of blood, urine and faeces were collected from each person for laboratory tests in PNG and Australia. Positive sera for dengue (15%) and Japanese encephalitis (JE) (6%) were identified. Surprisingly, a number of the sera were positive for scrub typhus (Orientia tsutsugamushi) (28%) and spotted fever (Rickettsia australis) (11%). The last reported cases of scrub typhus in PNG were during World War Two among the allied troops. This is the first time spotted fever (R. australis) has been reported in PNG. These conditions may have been the cause of the deaths described by the villagers. However, there were significantly more dengue-positive results among relatives of the deceased than non-relatives though no such difference was found with rickettsial infections: haemorrhagic dengue fever is thus the most likely cause of this recurring outbreak. Mining did not appear to be a direct causal factor for the deaths in the area.

PMID: 19583101 [PubMed - indexed for MEDLINE]

261. Southeast Asian J Trop Med Public Health. 2004 Jun;35(2):358-60.

Scrub typhus during pregnancy: a case report and review of the literature.

Phupong V(1), Srettakraikul K.

Author information:
(1)Department of Obstetrics and Gynecology, Faculty of Medicine, Chulalongkorn University, Bangkok, Thailand. vorapong.p@chula.ac.th

Scrub typhus is a rickettsial disease that is uncommon during pregnancy. We report a case of a 33-year-old woman, G1P0, 29 weeks pregnancy who presented to hospital with high fever, chill and headache for two weeks. Her diagnosis of scrub typhus was confirmed by serum immunofluorescent assay. She was successfully treated with chloramphenicol, but preterm delivery occurred. Her infant died from respiratory distress syndrome. No vertical transmission was demonstrated in this case. Scrub typhus should be listed in the differential diagnosis of acute febrile illness in pregnant women, who either live in, or return from, endemic areas. Chloramphenicol can be used safely during pregnancy if it is not circulating at the time of delivery.

PMID: 15691136 [PubMed - indexed for MEDLINE]

262. J Gastroenterol Hepatol. 2010 Jul;25(7):1331. doi: 10.1111/j.1440-1746.2010.06401.x.

Education and imaging. Gastrointestinal: Scrub typhus induced acute gastric ulceration.

Cho SW(1), Hwang JT, Oh TH.

Author information:
(1)Department of Internal Medicine, Sanggye Paik Hospital, Inje University College of Medicine, Seoul, Korea.

DOI: 10.1111/j.1440-1746.2010.06401.x
PMID: 20594266 [PubMed - indexed for MEDLINE]

263. Am J Trop Med Hyg. 2007 Jun;76(6):1148-52.

Acute respiratory distress syndrome in scrub typhus.

Wang CC(1), Liu SF, Liu JW, Chung YH, Su MC, Lin MC.

Author information:
(1)Division of Pulmonary and Critical Care Medicine, Department of Internal Medicine, Chang Gung Memorial Hospital, Kaohsiung Medical Center, Chang Gung University College of Medicine, Kaohsiung, Taiwan, Republic of China.

Scrub typhus is a mite-borne infectious disease caused by Orientia tsutsugamushi. Acute respiratory distress syndrome (ARDS) is a serious complication of scrub typhus. This study retrospectively reviewed the medical records of 72 patients diagnosed with scrub typhus from January 1998 to August 2006 in Kaohsiung Chang Gung Memorial Hospital in Taiwan. Eight of 72 scrub typhus patients with ARDS were included in the study; the other patients without ARDS were used as controls. The mortality rate for the scrub typhus patients with ARDS was 25%. The eight patients seldom had underlying diseases. Initial presentations of dyspnea and cough, white blood cell count, hematocrit, total bilirubin, and delayed used of appropriate antibiotics use were significant predictors of ARDS. Multivariate analysis showed that albumin, prothrombin time, and delayed use of appropriate

antibiotics were independent predictors of ARDS. Identification of these relative risk factors may help clinicians evaluate clinical cases of scrub typhus with ARDS.

PMID: 17556627 [PubMed - indexed for MEDLINE]

264. Southeast Asian J Trop Med Public Health. 2002 Dec;33(4):780-6.

Septic shock secondary to scrub typhus: characteristics and complications.

Thap LC(1), Supanaranond W, Treeprasertsuk S, Kitvatanachai S, Chinprasatsak S, Phonrat B.

Author information:
(1)Malaria National Center, Phnom Penh, Cambodia. lonchanthap@yahoo.com

Scrub typhus is an acute febrile illness caused by infection with Orientia tsutsugamushi transmitted by the bite of larval trombiculid mites (chiggers). A prospective study was conducted in septic shock patients in Maharat Hospital, Nakhon Ratchasima Province, Thailand, from 12 November 2001 to 5 January 2002. Of the 51 septic shock patients studied during the 7 week period, 18 (35.3%) were found to have evidence of scrub typhus infection; 3 patients (16.7%) died. In this study, septic shock caused by Orientia tsutsugamushi is the most prominent (35.3%) in endemic area of scrub typhus. Scrub typhus with septic shock patients results in organ failure: respiratory failure, DIC were predominant, followed by renal and hepatic involvement. Two deaths were due to respiratory failure and one death was as a result of combined respiratory and renal failure. Fever was the most common symptom, followed by headache, myalgia and dyspnea; lymphadenophathy and eschar are common signs. Laboratory findings revealed that almost all of the patients had a mild leukocytosis, reduced hematocrit and thrombocytopenia; SGOT, ALP, direct bilirubin (DB), total billirubin (TB), BUN, Cr were elevated; hypoalbuminemia was noted. Urinalysis showed that 88.9% of the patients had albuminuria. 77.8% of patients had abnormal chest X-rays.

PMID: 12757226 [PubMed - indexed for MEDLINE]

265. Changgeng Yi Xue Za Zhi. 1992 Dec;15(4):193-7.

[Epidemiology of scrub typhus in Matsu Peikang Island].

[Article in Chinese]

Lin TS(1), Leu HS.

Author information:
(1)Department of Internal Medicine, Chang Gung Memorial Hospital, Taipei, Taiwan, R.O.C.

A retrospective survey of the epidemiology of scrub typhus in 1990 in Matsu Peikang Island was made at the end of December of 1990. The diagnosis of scrub

typhus was based on the presence of escar, typical clinical manifestation and good response to tetracycline treatment. There were 89 cases of scrub typhus studied, and except for one civilian, the other 88 cases were military personnel, occurring in 2.2% of the total military personnel on the island. However it occurred in 0.08% of the civilian population. Seasonal distribution of scrub typhus was limited to between June and October of 1990. The peak incidence occurred in July. Scrub typhus was distributed over the whole island. A higher incidence of scrub typhus occurred in soldiers and sergeants than in officers. A significant different incidence was found in different units. No fatalities were reported. Different units. No fatalities were reported. Different military activities, duties and the location of the military unit may affect the incidence of scrub typhus. The scrub typhus epidemic was also related to temperature.

PMID: 1295653 [PubMed - indexed for MEDLINE]

266. Am J Trop Med Hyg. 2008 Jun;78(6):968-72.

Comparative analysis of nucleotide sequences of Orientia tsutsugamushi in different epidemic areas of scrub typhus in Shandong, China.

Yang LP(1), Zhao ZT, Li Z, Wang XJ, Liu YX, Bi P.

Author information:
(1)Department of Epidemiology and Health Statistics, School of Public Health, Shandong University, Jinan, People's Republic of China.

Scrub typhus, caused by Orientia tsutsugamushi, is a zoonosis that spreads rapidly in Shandong, China. To investigate the molecular characterization of O. tsutsugamushi in new and old epidemic areas in Shandong Province, we compared the genetic relationships of O. tsutsugamushi between Linyi and Tai'an districts, typical old and new epidemic areas in Shandong, respectively. O. tsutsugamushi was detected in blood from 12 of 16 patients in Linyi and in eschar from 3 of 4 patients in Tai'an; 17 of 128 rodents were found to be infected with O. tsutsugamushi in Linyi, and 4 of 68 rodents were found to be O. tsutsugamushi-positive in Tai'an. The results indicated less genetic variation in O. tsutsugamushi between the new and old epidemic areas, and the Sdu-1 type, similar to Japan Kawasaki, was the main genotype of O. tsutsugamushi in Shandong Province.

PMID: 18541778 [PubMed - indexed for MEDLINE]

267. Microbiol Immunol. 1997;41(6):503-7.

Prevalence of genotypes of Orientia tsutsugamushi in patients with scrub typhus in Miyazaki Prefecture.

Horinouchi H(1), Murai K, Okayama A, Nagatomo Y, Tachibana N, Tsubouchi H.

Author information:
(1)Department of Internal Medicine II, Miyazaki University School of Medicine,

Japan.

The genotypes of Orientia tsutsugamushi in patients with scrub typhus in Miyazaki Prefecture were examined by polymerase chain reaction (PCR) and the restriction fragment length polymorphism method. Specific patterns for genotypes Irie, Hirano, Tazume and Yoshimura were detected in 26, 6, 5 and 2 of 39 DNA samples obtained from peripheral blood mononuclear cells, respectively. DNA sequences of the PCR products from the Tazume strain were genetically very close to the Hirano strain and the Yoshimura strain was also very close to the Karp strain. Furthermore, the DNA sequences from the Irie and Tazume strains were completely homologous to the reported sequences of the Kawasaki and Kuroki strains, respectively.

PMID: 9251062 [PubMed - indexed for MEDLINE]

268. Ann N Y Acad Sci. 2003 Jun;990:359-64.

Outbreak of scrub typhus in southern India during the cooler months.

Mathai E(1), Rolain JM, Verghese GM, Abraham OC, Mathai D, Mathai M, Raoult D.

Author information:
(1)Department of Clinical Microbiology, Christian Medical College and Hospital, Vellore 632004, Tamil Nadu, India.

Orientia tsutsugamushi, the agent of scrub typhus, is a strict intracellular bacterium which is found in many parts of Asia including India. During the past few years, the number of patients with rickettsial infection and scrub typhus has increased, especially during the cooler months. We report in this study a recent outbreak of scrub typhus recorded during the cooler months (October 2001 to February 2002) in patients admitted to our hospital with acute febrile illness associated with diverse signs and symptoms. Overall, 28 patients were clinically and serologically confirmed to have scrub typhus. Fever for more than one week was the only common manifestation. Myalgias was the next most common feature (52%), and rash was observed in only 22% of the cases. Seventeen patients treated with doxycycline recovered in 1 to 3 days, as well as two patients who received chloramphenicol. In five patients who received ciprofloxacin, fever subsided only after five days. Finally three patients (10.7%) died, including one patient treated with doxycycline. These data indicate that scrub typhus is a reemerging infectious disease in India with a possibility of drug resistance. This reemergence emphasizes the need for further prospective studies to design effective control measures.

PMID: 12860654 [PubMed - indexed for MEDLINE]

269. Asian Pac J Allergy Immunol. 1998 Jun-Sep;16(2-3):119-25.

Seroprevalence of scrub typhus infection in patients with pyrexia at some malaria clinics in three western provinces of Thailand.

Chanyasanha C(1), Kaeburong K, Chenchittikul M, Sujirarat D.

Author information:
(1)Department of Microbiology, Faculty of Public Health, Mahidol University, Bangkok, Thailand.

In Thailand, the epidemiological data on scrub typhus infection represents only "the tip of an iceberg" especially in malaria clinics where patients come to seek attention because of other febrile illnesses that may have initial clinical signs that are indistinguishable from malaria. The objectives of this study were to determine the prevalence of antibody titers to Orientia tsutsugamushi, and its various strains, among patients at some malaria clinics in three western provinces of Thailand. The sample was represented by 200 patients from 6 malaria clinics in Ratchaburi, Petchaburi and Kanchanaburi provinces between June and November, 1994. Blood specimens were collected with their consent. Immunofluorescent antibody assays (IFA) were used for measuring IgM and IgG antibody titers for scrub typhus infection. The results showed that the prevalence rate for scrub typhus infection (IgM and/or IgG titer > or = 50) was 59.50% (119 cases). The immunofluorescent antibody response to various strains of O. tsutsugamushi showed that co-infections with the Karp, the Gilliam and the Kato strains were the most common (found in 68.10% of cases). Geometric mean antibody titers (GMT) were highest for the Karp strain, followed by the Gilliam then Kato strains. In conclusion, this study indicates that the prevalence rate of scrub typhus is not rare in these areas.

PMID: 9876950 [PubMed - indexed for MEDLINE]

270. Emerg Infect Dis. 2006 Feb;12(2):290-5.

Scrub typhus, Republic of Palau.

Demma LJ(1), McQuiston JH, Nicholson WL, Murphy SM, Marumoto P, Sengebau-Kingzio M, Kuartei S, Durand AM, Swerdlow DL.

Author information:
(1)Centers for Disease Control and Prevention, Atlanta, Georgia 30333, USA.
ldemma@cdc.gov

Scrub typhus, caused by Orientia tsutsugamushi, is a severe febrile illness transmitted to humans by trombiculid mites, which normally feed on rodents. The first known outbreak of scrub typhus in Palau occurred in 2001 to 2003 among residents of the remote southwest islands. To determine the extent of scrub typhus distribution in Palau, we tested serum samples from humans and rodents for antibodies to O. tsutsugamushi. Of 212 Palau residents surveyed in 2003, 101 (47.6%) had immunoglobulin G (IgG) antibody titers >1:64, and 56 (26.4%) had concurrent IgG and IgM antibody titers >1:512 and 1:64, respectively. Of 635 banked serum samples collected from Palau residents in 1995, 34 (5.4%) had IgG antibody titers >1:64. Sera collected from rodents (Rattus norvegicus and R. rattus) in 2003 and 2005 were tested, and 18 (28.6%) of 63 had IgG antibody titers >1:64. These findings suggest that scrub typhus is endemic in Palau.

DOI: 10.3201/eid1202.050967

PMCID: PMC3373099
PMID: 16494757 [PubMed - indexed for MEDLINE]

271. Trop Doct. 2010 Jul;40(3):169-70. doi: 10.1258/td.2010.090468.

An outbreak of scrub typhus in Bishnupur district of Manipur, India, 2007.

Singh SI(1), Devi KP, Tilotama R, Ningombam S, Gopalkrishna Y, Singh TB, Murhekar MV.

Author information:
(1)Field Epidemiology Training Programme, National Institute of Epidemiology (ICMR), R-127, Tamil Nadu Housing Board, Ayapakkam, Ambattur, Chennai-600 077, Tamil Nadu, India.

Seasonal outbreaks of febrile illness with eschar have been occurring in Bishnupur district of Manipur since 2001. The aetiology of these outbreaks was unknown. We investigated a similar outbreak in 2007 in order to confirm the aetiology and identify its risk factors. We identified 38 patients who met the case definition (attack rate: 3.4/1000), including two deaths (case fatality ratio = 5.3%). Half of the female patients had eschar on the perineal area. The clinical picture and Weil-Felix positivity suggested that the outbreak was due to scrub typhus. The disease was more common among individuals who defecated or urinated in the jungle or bushy areas from a squatting position. We recommended educating the community about the common symptoms of the disease, encouraging them to seek early treatment from public health facilities and suggested that they be taught to avoid defecating/urinating from a squatting position in the jungle.

DOI: 10.1258/td.2010.090468
PMID: 20555047 [PubMed - indexed for MEDLINE]

272. Korean J Lab Med. 2009 Apr;29(2):116-21. doi: 10.3343/kjlm.2009.29.2.116.

[Incidence and type of monoclonal or biclonal gammopathies in scrub typhus].

[Article in Korean]

Cho JH(1), Park DS.

Author information:
(1)Department of Laboratory Medicine, School of Medicine, Wonkwang University, Iksan, Korea.

BACKGROUND: Korea is an endemic area of scrub typhus and it is a common seasonal febrile illness. Although, various humoral immune responses to scrub typhus have been documented, no association between gammopathy and scrub typhus has ever been reported. We analyzed the incidences and types of monoclonal and biclonal gammopathies in scrub typhus for better coping with those gammopathies in scrub typhus.
METHODS: Anti-Orientia tsutsugamushi antibody-positive sera identified by indirect immunofluorescence assay were acquired from 40 patients with confirmed

scrub typhus. Monoclonal and biclonal gammopathies were screened by protein electrophoresis and were confirmed using immunofixation electrophoresis (IFE). Laboratory findings on admission of the patients with monoclonal or biclonal gammopathy were investigated retrospectively to characterize the gammopathies.
RESULTS: Monoclonal or biclonal gammopathies were detected in 30% (12/40) of patients with scrub typhus (IgG-lambda, 40%; IgG-kappa, 30%; IgM-kappa, 10%; IgM-lambda, 10%; IgA-kappa, 5%; IgA-lambda, 5%). Concentrations of clonal immunoglobulin were less than 3 g/dL in all gammopathies, and hypercalcemia was not detected in any of the patients.
CONCLUSIONS: Our results suggest possible association between gammopathies and scrub typhus. Further studies in larger series will be needed for exact incidence and clinical course of gammopathies in scrub typhus.

DOI: 10.3343/kjlm.2009.29.2.116
PMID: 19411777 [PubMed - indexed for MEDLINE]

273. Chin Med J (Engl). 2007 Aug 5;120(15):1314-8.

Molecular epidemic survey on co-prevalence of scrub typhus and marine typhus in Yuxi city, Yunnan province of China.

Zhang LJ(1), Li XM, Zhang DR, Zhang JS, Di Y, Luan MC, Fu XP.

Author information:
(1)Department of Rickettsiology, National Institute of Communicable Disease Control and Prevention, Chinese Center for Disease Control and Prevention, Beijing 102206, China. zhanglijuan@icdc.cn

BACKGROUND: Human rickettsioses are worldwide zoonoses and it is not easy to differentiate them from other infectious diseases because of their atypical manifestation. In recent years the number of patients with fever of unknown causes from Hongta District CDC, Yuxi city of Yunnan Province has been increasing significantly in the summer. Diagnosis of scrub typhus was made by local clinicians. In order to ascertain the disease, we undertook a laboratory investigation for such patients from August 18 to 26, 2005.
METHODS: Active surveillance was conducted by Hongta District CDC Yuxi city of Yunnan Province from 2002 to 2004 and basic data were obtained from cases confirmed according to clinical definitions. Average incidences and town-level incidences were calculated during the study periods. Blood samples were analyzed by PCR and serological test. Based on the groEL gene sequences a paired general outer primers (Gro-1 and Gro-2) targeting typhus, spotted fever as well as scrub typhus and two paired inner primers (SF1, SR2 and TF1, TR2) for typhus together with spotted fever and scrub typhus, respectively, were designed to perform a multiplex-nested PCR. Serological assay was carried out by indirect immunofluorescence assay with 7 different rickettsial antigens, i.e., R.mossori, R.sibirica, R.conorii, O.tsutsugamushi, B.quintana, B.henselae and Coxilella burnetii phase II Ag.
RESULTS: Epidemiological surveillance showed that from 2002 to 2004, the average incidences of the scrub typhus or scrub typhus with murine typhus were 222.1/10(5), 204.3/10(5) and 109.6/10(5), respectively. Of 13 blood samples taken during acute stage of illness, 6 showed the amplified products for scrub typhus and the sequenced products showed 100%, 99%, 99%, 99%, 99%, 99% similarity to O.tsutsugamushi Karp but they shared the same deduced amino acid sequences, which

indicated 100% identity with the heat shock protein of the O.tsutsugamushi Karp strain. Five yielded PCR products for murine typhus and their corresponding nucleotide sequences exhibited 100%, 100%, 99%, 99% and 99% similarity to R. mossori Wilmington and the analyses of predicted amino acid sequences indicated 100%, 100%, 98%, 98% and 98% identity with the heat shock protein of R. mossori Wilmington strain. Of the 8 PCR positive patients, 3 showed a co-infection of scrub typhus with murine typhus. All the 13 serum samples from febrile patients were positive against O. tsutsugamushi and 8 of them were positive against R. mossori. All of the 8 paired specimens had four-fold elevation of antibody against O. tsutsugamushi, and seroconversion for typhus was demonstrated in 3 paired serum samples. Another finding in the study was that a high seropositive prevalence (76.9%) of Q fever was detected.
CONCLUSION: It's confirmed that co-prevalence of scrub typhus with murine typhus are occurring in Yuxi city of Yunnan province, China. Other rickettsial diseases also need to be investigated in these areas.

PMID: 17711736 [PubMed - indexed for MEDLINE]

274. Parasitol Res. 2016 May;115(5):1923-38. doi: 10.1007/s00436-016-4934-4. Epub 2016 Feb 2.

An updated distribution and hosts: trombiculid mites (Acari: Trombidiformes) associated with small mammals in Yunnan Province, southwest China.

Peng PY(1), Guo XG(2), Ren TG(1), Dong WG(1), Song WY(1).

Author information:
(1)Vector Laboratory, Institute of Pathogens and Vectors, Dali University (Branch of Yunnan Provincial Key Laboratory for Zoonosis Control and Prevention), Dali, Yunnan Province, 671000, People's Republic of China. (2)Vector Laboratory, Institute of Pathogens and Vectors, Dali University (Branch of Yunnan Provincial Key Laboratory for Zoonosis Control and Prevention), Dali, Yunnan Province, 671000, People's Republic of China. xianguoguo@yahoo.com.

Trombiculid mites (or chigger mites) are a large group of arthropods, and some of these species are vectors of Orientia tsutsugamushi, the causative agent of tsutsugamushi disease (scrub typhus). Yunnan Province is situated in the southwest of China, and its complicated topography, special altitude gradients, and high biodiversity have aroused the interest of many scientists to study the fauna and species diversity of plants and animals. To replenish our former faunal study, this paper listed all the scientific names of trombiculid mites in Yunnan Province, together with their hosts and collection sites (geographical distribution). A total of 120,138 individuals of trombiculid mites were collected from the body surface of 13,760 small mammal hosts (89.06 % of them are rodents) in 29 collection sites (counties) of Yunnan Province from 2001 to 2013. The 120,138 mites were identified as comprising 2 families (Trombiculidae and Leeuwenhoekiidae), 26 genera, and 274 species. The genus Leptotrombidium had the most abundant species (109 species) of 26 genera. Of the six main vectors of scrub typhus in China, five of them were found in Yunnan. Of the 274 chigger mite species, 23 were determined as the newly recorded species (new records), which were found in Yunnan Province for the first time. The identified 274 species of trombiculid mites in the present paper are much more than those from other

provinces in China and even largely exceeded the species of trombiculid mites recorded from some other regions and countries in the world. Based on the formula of Chao 1, the total number of chigger mite species in Yunnan was approximately estimated to be 346 species, and about 72 species might have been missed in our sampling process.

DOI: 10.1007/s00436-016-4934-4
PMID: 26833324 [PubMed - in process]

275. BMC Infect Dis. 2009 Jun 4;9:82. doi: 10.1186/1471-2334-9-82.

Clinical characteristics of the autumn-winter type scrub typhus cases in south of Shandong province, northern China.

Liu YX(1), Feng D, Suo JJ, Xing YB, Liu G, Liu LH, Xiao HJ, Jia N, Gao Y, Yang H, Zuo SQ, Zhang PH, Zhao ZT, Min JS, Feng PT, Ma SB, Liang S, Cao WC.

Author information:
(1)Department of Nosocomial Infection Management and Disease Control, Institute of Hospital Management, Chinese People's Liberation Army General Hospital, Beijing, PR China. Yunxiliu6511@yahoo.com.cn

BACKGROUND: Before 1986, scrub typhus was only found endemic in southern China. Because human infections typically occur in the summer, it is called "summer type". During the autumn-winter period of 1986, a new type of scrub typhus was identified in Shandong and northern Jiangsu province of northern China. This newly recognized scrub typhus was subsequently reported in many areas of northern China and was then called "autumn-winter type". However, clinical characteristics of associated cases have not been reported.
METHODS: From 1995 to 2006, all suspected scrub typhus cases in five township hospitals of Feixian county, Shandong province were enrolled. Indirect immunofluorescent assay (IFA) was used as confirmatory serodiagnosis test. Polymerase chain reaction (PCR) connected with restriction fragment length polymorphism (RFLP) and sequence analyses were used for genotyping of O. tsutsugamushi DNAs. Clinical symptoms and demography of confirmed cases were analyzed.
RESULTS: A total of 480 scrub typhus cases were confirmed. The cases occurred every year exclusively between September and December with a peak occurrence in October. The case numbers were relatively higher in 1995, 1996, 1997, and 2000 than in other years. 57.9% of cases were in the group aged 21-50. More cases occurred in male (56%) than in female (44%). The predominant occupational group of the cases was farmers (85.0%). Farm work was reported the primary exposure to infection in 67.7% of cases. Fever, rash, and eschar were observed in 100.0%, 90.4%, and 88.5% of cases, respectively. Eschars formed frequently on or around umbilicus, abdomen areas, and front and back of waist (34.1%) in both genders. Normal results were observed in 88.7% (WBC counts), 84.5% (PLT counts), and 89.7% (RBC counts) of cases, respectively. Observations from the five hospitals were compared and no significant differences were found.
CONCLUSION: The autumn-winter type scrub typhus in northern China occurred exclusively from September to December with a peak occurrence in October, which was different from the summer type in southern China. In comparison with the summer type, complications associated with autumn-winter type scrub typhus were less severe, and abnormalities of routine hematological parameters were less

obvious.

DOI: 10.1186/1471-2334-9-82
PMCID: PMC2703643
PMID: 19493361 [PubMed - indexed for MEDLINE]

276. Southeast Asian J Trop Med Public Health. 2004 Dec;35(4):845-51.

Gastrointestinal manifestations of septic patients with scrub typhus in Maharat Nakhon Ratchasima Hospital.

Aung-Thu(1), Supanaranond W, Phumiratanaprapin W, Phonrat B, Chinprasatsak S, Ratanajaratroj N.

Author information:
(1)Department of Medical Research, Lower Myanmar, Ministry of Health, Myanmar, Thailand. athudmr@mail4u.com.mm

Scrub typhus is an acute febrile illness caused by Orientia induced vasculitis, which is common in Asia and the Pacific Islands and is sometimes also encountered in Western countries. Even though it can cause multi-organ dysfunctions, there is limited information regarding the relationship between scrub typhus infection and gastrointestinal dysfunction. Therefore, a cross-sectional study was conducted to discover the gastrointestinal manifestations of septic patients with scrub typhus infection. During the study period, 80 septic cases were recruited, and according to the results of immunofluorescent antibody testing (IFA), 20 (25%) were found to have scrub typhus infection. The most common gastrointestinal symptoms of scrub typhus patients were vomiting 13 (65%), nausea 12 (60%), diarrhea 9 (45%), and hametamesis or melena 5 (25%). Gastrointestinal signs included hepatomegaly 8 (40%), jaundice 7 (35%), and abdominal pain 4 (20%). Elevation of SGOT, SGPT, and alkaline phosphatase were 16 (80%), 14 (70%), and 16 (80%), respectively. Direct bilirubin was elevated in 19 (95%) of the cases and half of the cases had a low serum protein level. Of scrub typhus cases, 8 (40%) had eschars. The sites of eschars were mostly in hidden areas, such as on the back, genitalia and abdomen. Three of the five patients with eschar had hepatomegaly on ultrasound examination. The significant findings of the scrub typhus septic patients with eschar on endoscopic examination were gastritis in two cases, gastritis with gastric erosion in two cases, and one case showed a duodenal ulcer and erosion. The differentiating point for endoscopic findings in scrub typhus compared to the other causes was that the stomach lesions were more frequent and severe than the duodenal lesions. According to our endoscopic findings, physicians should be aware of gastric and duodenal lesions in febrile patients with gastrointestinal symptoms, such as abdominal pain or discomfort and indigestion. Scrub typhus can cause gastrointestinal and liver dysfunction.

PMID: 15916079 [PubMed - indexed for MEDLINE]

277. Southeast Asian J Trop Med Public Health. 2001 Mar;32(1):132-6.

Evaluation of a newly developed dipstick test for the rapid diagnosis of scrub typhus in febrile patients.

Chinprasatsak S(1), Wilairatana P, Looareesuwan S, Chappuis F, Akkhavong K,
Laferl H, Eamsila C.

Author information:
(1)Department of Medicine, Maharaj Nakhon Ratchasima Hospital, Nakhon Ratchasima,
Thailand.

Scrub typhus is a potentially fatal, febrile disease prevalent in rural Asia. The etiological agent, Orientia tsutsugamushi, is transmitted to humans by the bite of a larval trombiculid mite. No current diagnostic test is sufficiently practical for use by physicians working in rural areas. A new dipstick test using a dot blot immunoassay format has been developed for the serodiagnosis of scrub typhus. We evaluated this test on 83 patients presenting with acute fever of unknown origin at Maharaj Hospital, a tertiary care medical center in Nakhon Ratchasima, Northeast Thailand. The diagnosis of scrub typhus was confirmed in 30 of these patients (36%) by the indirect immunoperoxidase test. The sensitivity of the test was 87% and its specificity was 94%. The dot blot immunoassay dipstick is accurate, rapid, easy to use, and relatively inexpensive. It appears to be the best currently available test for diagnosing scrub typhus in rural areas where this disease predominates.

PMID: 11485073 [PubMed - indexed for MEDLINE]

278. Mil Med. 2010 Jan;175(1):48-54.

Serological surveillance of scrub typhus, murine typhus, and leptospirosis in small mammals captured at Twin Bridges Training Area, Gyeonggi Province, Republic of Korea, 2005-2007.

Sames WJ(1), Klein TA, Kim HC, Gu SH, Kang HJ, Shim SH, Ha SJ, Chong ST, Lee IY,
Richards AL, Yi SH, Song JW.

Author information:
(1)Defense Logistics Agency, Attn: DES-E, Fort Belvoir, VA 22060-6221, USA.

Soldiers from the Republic of Korea and the United States conduct armistice military operations at Twin Bridges Training Area (TBTA) located near the demilitarized zone (DMZ) and are exposed to zoonotic disease pathogens that small mammals and their potentially disease-carrying ectoparasites transmit. TBTA is a 36 km2 rural training site with small villages and various forms of agriculture along its boundary. At TBTA, rodents, insectivores, and their ectoparasites are commonly found in association with unmanaged habitats of various densities of tall grasses, herbaceous plants, shrubs, briars, and crawling vegetation. Rodents and insectivores were collected during the winter (November-December 2005 and December 2006) and early spring (March 2007), and serologically tested for the presence of scrub typhus, murine typhus, and leptospirosis antibodies. Of the six species of small mammals collected, Apodemus agrarius, the common striped field mouse and known reservoir of scrub typhus, was the most frequently collected (96.1%), followed by Crocidura lasiura (2.5%), Micromys minutus (0.5%), Myodes regulus (0.5%), Mus musculus (0.3%), and Rattus rattus (0.1%). A. agrarius (56.1%), M. musculus (66.7%), M. minutus (25%), and R. rattus (100%) were

positive for scrub typhus antibodies. Only A. agrarius (14.7%) and C. lasiura (4.5%) were positive for murine typhus antibodies, whereas only A. agrarius (1.5%) was seropositive for leptospirosis. Seroprevalence rates of scrub typhus and murine typhus based on weight and sex of A. agrarius are presented.

PMID: 20108842 [PubMed - indexed for MEDLINE]

279. Emerg Infect Dis. 2008 Sep;14(9):1483-5. doi: 10.3201/eid1409.071259.

Genotyping of Orientia tsutsugamushi from humans with scrub typhus, Laos.

Parola P, Blacksell SD, Phetsouvanh R, Phongmany S, Rolain JM, Day NP, Newton PN, Raoult D.

DOI: 10.3201/eid1409.071259
PMCID: PMC2603112
PMID: 18760027 [PubMed - indexed for MEDLINE]

280. Am J Trop Med Hyg. 2007 May;76(5):806-9.

Distribution of eschars on the body of scrub typhus patients: a prospective study.

Kim DM(1), Won KJ, Park CY, Yu KD, Kim HS, Yang TY, Lee JH, Kim HK, Song HJ, Lee SH, Shin H.

Author information:
(1)Division of Infectious Diseases, Department of Internal Medicine, Chosun University College of Medicine, Gwangju, Korea. drongkim@chosun.ac.kr

Eschar is an important finding for the diagnosis of scrub typhus. The IFA test for possible scrub typhus was performed. The presence or absence of eschar was thoroughly examined. Among the 176 scrub typhus cases confirmed by IFA, 162 (92.0%) cases had eschar; 128 patients (79.5%) had eschars on the front of the body. Eschars were primarily detected in males within 30 cm below the umbilicus (19 patients, 35.8%). Distributions on the lower extremities and the front chest above the umbilicus were 22.6% (12 patients) and 20.8% (11 patients), respectively. A different pattern was seen in females. The most prevalent area was the front chest above the umbilicus, which accounted for 40.7% (44 patients) of all the detected eschars. Our study is the first report of a schematic diagram that shows the differences between the males and females with respect to eschar location in scrub typhus patients.

PMID: 17488895 [PubMed - indexed for MEDLINE]

281. J Obstet Gynaecol Res. 1997 Feb;23(1):75-8.

Pregnancy with scrub typhus and vertical transmission: a case report.

Suntharasaj T(1), Janjindamai W, Krisanapan S.

Author information:
(1)Department of Obstetrics and Gynaecology, Faculty of Medicine, Prince of Songkla University, Thailand.

Scrub typhus is a rickettsian disease which is seldom found in pregnancy. A 31-year-old, 34 weeks pregnant woman presented with fever, chill and cough for 6 weeks. Fetal jeopardy was found then a cesarean section was performed to deliver a 2,200 g male with hepatosplenomegaly. The mother's diagnosis was confirmed by positive Weil-Felix (OXK titer 1:320) and scrub typhus (titer 1:1600) tests. Vertical transmission was also demonstrated by a positive scrub typhus IgM in her child.

PMID: 9094822 [PubMed - indexed for MEDLINE]

282. Am J Trop Med Hyg. 2003 Jul;69(1):60-6.

Laboratory diagnosis of two scrub typhus outbreaks at Camp Fuji, Japan in 2000 and 2001 by enzyme-linked immunosorbent assay, rapid flow assay, and Western blot assay using outer membrane 56-kD recombinant proteins.

Jiang J(1), Marienau KJ, May LA, Beecham HJ 3rd, Wilkinson R, Ching WM, Richards AL.

Author information:
(1)Rickettsial Diseases Department, Naval Medical Research Center, Silver Spring, Maryland 20910-7500, USA.

Two scrub typhus outbreaks occurred among U.S. Marines training at Camp Fuji, Japan, between October 25 and November 3, 2000 and October 17 and November 30, 2001. Nine cases in approximately 800 Marines in 2000 and eight cases in approximately 900 Marines in 2001 (approximate attack rates = 1.1% and 0.9%, respectively) reported with signs and symptoms of fever, rash, headache, lymphadenopathy, myalgia, and eschar. Serologies and rapid response to doxycycline treatment indicated they had scrub typhus. Sixty-four convalescent serum samples (18 suspected cases and 46 negative controls) from U.S. Marines training at Camp Fuji during the outbreaks were assessed by enzyme-linked immunosorbent assay (ELISA), rapid flow assay (RFA), and Western blot assay for evidence of infection with Orientia tsutsugamushi, the causative agent of scrub typhus. All but one suspected case had serologic evidence of scrub typhus and all 46 control sera were non-reactive to O. tsutsugamushi antigens. The recombinant 56-kD antigen (r56) from the Karp, Kato and Gilliam strains of O. tsutsugamushi in an ELISA format provided better results than Karp r56 alone (ELISA and RFA) or whole cell antigen preparation from Karp, Kato and Gilliam (ELISA).

PMID: 12932099 [PubMed - indexed for MEDLINE]

283. Am J Trop Med Hyg. 2015 Sep;93(3):517-20. doi: 10.4269/ajtmh.14-0772. Epub 2015 Jul 6.

Causes of Fever in Rural Southern Laos.

Mayxay M(1), Sengvilaipaseuth O(2), Chanthongthip A(2), Dubot-Pérès A(2), Rolain JM(2), Parola P(2), Craig SB(2), Tulsiani S(2), Burns MA(2), Khanthavong M(2), Keola S(2), Pongvongsa T(2), Raoult D(2), Dittrich S(2), Newton PN(1).

Author information:
(1)Microbiology Laboratory, Lao-Oxford-Mahosot Hospital-Wellcome Trust Research Unit, Mahosot Hospital, Vientiane, Lao People's Democratic Republic; Faculty of Postgraduate Studies, University of Health Sciences, Vientiane, Lao People's Democratic Republic; Centre for Tropical Medicine and Global Health, Churchill Hospital, University of Oxford, Oxford, United Kingdom; UMR_D190 "Emergence des Pathologies Virales," Aix-Marseille University, IRD French Institute of Research for Development, EHESP French School of Public Health, Marseille, France; WHO Collaborating Center for Rickettsioses and other Arthropod-Borne Bacterial Diseases, Aix-Marseille University, Marseille, France; WHO Collaborating Centre for Reference and Research on Leptospirosis, Queensland, Australia; Faculty of Science Health, Education and Engineering, University of the Sunshine Coast, Queensland, Australia; Department of International Health, Immunology and Microbiology, Copenhagen Centre for Disaster Research, University of Copenhagen, Copenhagen, Denmark; Centre of Malariology, Parasitology and Entomology, Vientiane, Lao People's Democratic Republic; Phalanxay District Hospital, Savannakhet Province, Lao People's Democratic Republic; Savannakhet Provincial Malaria Station, Savannakhet Province, Lao People's Democratic Republic mayfong@tropmedres.ac paul@tropmedres.ac. (2)Microbiology Laboratory, Lao-Oxford-Mahosot Hospital-Wellcome Trust Research Unit, Mahosot Hospital, Vientiane, Lao People's Democratic Republic; Faculty of Postgraduate Studies, University of Health Sciences, Vientiane, Lao People's Democratic Republic; Centre for Tropical Medicine and Global Health, Churchill Hospital, University of Oxford, Oxford, United Kingdom; UMR_D190 "Emergence des Pathologies Virales," Aix-Marseille University, IRD French Institute of Research for Development, EHESP French School of Public Health, Marseille, France; WHO Collaborating Center for Rickettsioses and other Arthropod-Borne Bacterial Diseases, Aix-Marseille University, Marseille, France; WHO Collaborating Centre for Reference and Research on Leptospirosis, Queensland, Australia; Faculty of Science Health, Education and Engineering, University of the Sunshine Coast, Queensland, Australia; Department of International Health, Immunology and Microbiology, Copenhagen Centre for Disaster Research, University of Copenhagen, Copenhagen, Denmark; Centre of Malariology, Parasitology and Entomology, Vientiane, Lao People's Democratic Republic; Phalanxay District Hospital, Savannakhet Province, Lao People's Democratic Republic; Savannakhet Provincial Malaria Station, Savannakhet Province, Lao People's Democratic Republic.

The etiology of fever in rural Lao People's Democratic Republic (Laos) has remained obscure until recently owing to the lack of laboratory facilities. We conducted a study to determine the causes of fever among 229 patients without malaria in Savannakhet Province, southern Laos; 52% had evidence of at least one diagnosis (45% with single and 7% with apparent multiple infections). Among patients with only one diagnosis, dengue (30.1%) was the most common, followed by leptospirosis (7.0%), Japanese encephalitis virus infection (3.5%), scrub typhus (2.6%), spotted fever group infection (0.9%), unspecified flavivirus infection (0.9%), and murine typhus (0.4%). We discuss the empirical treatment of fever in relation to these findings.

© The American Society of Tropical Medicine and Hygiene.

DOI: 10.4269/ajtmh.14-0772
PMCID: PMC4559689
PMID: 26149859 [PubMed - indexed for MEDLINE]

284. Scand J Infect Dis. 2006;38(3):200-2.

Scrub typhus imported to Scandinavia.

Jensenius M(1), Montelius R, Berild D, Vene S.

Author information:
(1)Department of Internal Medicine, Aker University Hospital, Oslo, Norway.
mogens.jensenius@ioks.uio.no

We present the first 3 cases of confirmed scrub typhus imported to Scandinavia. The patients were infected in Thailand, Laos and Sri Lanka, respectively. Treatment with ciprofloxacin and doxycycline lead to rapid cure in 2 of the patients, while the third patient, who was not treated, had a prolonged convalescence. Specific antibodies against Orientia tsutsugamushi, the causative agent of scrub typhus, were detected in all 3 cases.

DOI: 10.1080/00365540500277342
PMID: 16500780 [PubMed - indexed for MEDLINE]

285. Trans R Soc Trop Med Hyg. 2009 Nov;103(11):1153-8. doi:
10.1016/j.trstmh.2009.02.006. Epub 2009 Mar 14.

Scrub typhus in Darjeeling, India: opportunities for simple, practical prevention measures.

Sharma PK(1), Ramakrishnan R, Hutin YJ, Barui AK, Manickam P, Kakkar M, Mittal V, Gupte MD.

Author information:
(1)Field Epidemiology Training Programme, National Institute of Epidemiology, Indian Council of Medical Research, R 127 Third Avenue, Tamil Nadu Housing Board Phase I and II, Ayapakkam, Chennai, Tamil Nadu 600077, India.

To identify risk factors for scrub typhus in Darjeeling, India, we compared 62 scrub typhus cases (acute fever with eschar and specific IgM) with 62 neighbourhood controls. Cases were more likely to live close to bushes [matched odds ratio (MOR) 10; 95% CI 2.3-63] and wood piles (MOR 3.5; 95% CI 1.5-9.5), to work on farms (MOR 10; 95% CI 2.7-63), to observe rodents at home (MOR 3.6; 95% CI 1.4-11) and at work (MOR 9; 95% CI 2.4-57), and to rear domestic animals (MOR 2.4; 95% CI 1.1-5.7). Cases were less likely to wash after work (MOR 0.4; 95% CI 0.1-0.9) and change clothes to sleep (MOR 0.2; 95% CI 0.1-0.5). A cleaner, rodent-controlled environment may prevent exposure to scrub typhus. Personal protection measures and better hygiene could further reduce individual risk.

DOI: 10.1016/j.trstmh.2009.02.006
PMID: 19286238 [PubMed - indexed for MEDLINE]

286. Am J Trop Med Hyg. 2007 Oct;77(4):719-22.

Severe scrub typhus confirmed early via immunohistochemical staining.

Kim DM(1), Lim SC, Won KJ, Choi YJ, Park KH, Jang WJ.

Author information:
(1)Department of Internal Medicine, Chosun University College of Medicine, Gwangju, Korea. drongkim@chosun.ac.kr

We describe a case of interstitial pneumonia that was confirmed as scrub typhus by immunohistochemical (IHC) staining of an eschar. When a patient presents with interstitial pneumonia accompanied by generalized lymphadenopathy on the thoracic CT scan, clinicians should suspect scrub typhus, especially when the patient has a history of travel to a scrub typhus-endemic area. IHC staining on an eschar revealed invasion by Orientia tsutsugamushi coccobacilli of the patient's sweat ducts and glands as well as vascular endothelium. IHC staining of an eschar could be used as an early-confirmation diagnostic method to establish scrub typhus.

PMID: 17978077 [PubMed - indexed for MEDLINE]

287. Ren Fail. 2003 May;25(3):397-410.

Scrub typhus: a frequently overlooked cause of acute renal failure.

Yen TH(1), Chang CT, Lin JL, Jiang JR, Lee KF.

Author information:
(1)Division of Nephrology, Chang Gung Memorial Hospital, Chang Gung Medical College and University, Taipei, Taiwan.

Acute renal failure associated with scrub typhus infection is not rare as previously thought. The possibility of scrub typhus should be borne in mind when patients present with fever and varying degrees of acute renal failure, particularly if an eschar exists, along with a history of environmental exposure in an area like Taiwan, where scrub typhus is endemic. Prompt diagnosis and the use of appropriate antibiotics can rapidly alter the clinical course of the disease and prevent the development of serious or fatal complications. To illustrate the above point, this study reports 3 cases of scrub typhus associated with acute renal failure. They were seen at Chang Gung Memorial Hospital in a 2-year interval. Case 1 was referred from district hospital with clinical features of multiple organ dysfunctions, including shock, fever, acute respiratory failure, acute renal failure, and acute hepatitis. Case 2 was admitted with the chief problems of shock, fever, acute renal failure, and DIC. Case 3 visited our outpatient clinic due to fever, maculopapular rash and acute renal failure. In all these patients, the diagnosis was confirmed using immunofluorescence techniques, which showed that Orientia tsutsugamushi had an IgM titer of 1:80 or greater. Notably, despite having varying degrees of acute

renal deterioration, the patients responded very well to doxycycline therapy and recovered completely. Additionally, a total of 4 similar cases of scrub typhus associated with acute renal failure were reviewed from the past literature.

PMID: 12803503 [PubMed - indexed for MEDLINE]

288. J Korean Med Sci. 2000 Jun;15(3):343-5.

Acute respiratory distress syndrome associated with scrub typhus: diffuse alveolar damage without pulmonary vasculitis.

Park JS(1), Jee YK, Lee KY, Kim KY, Myong NH, Seo PW.

Author information:
(1)Department of Internal Medicine, College of Medicine, Dankook University, Cheonan, Korea. jspark@anseo.dankook.ac.kr

Pathologic findings of scrub typhus have been characterized by vasculitis of the microvasculature of the involved organ resulting from a direct invasion by Orientia tsutsugamushi. We experienced a case of acute respiratory distress syndrome (ARDS) associated with scrub typhus. The case was proven by eschar and high titer of serum IgM antibody (positive at 1:1280). Open lung biopsy showed diffuse alveolar damage (DAD) in the organizing stage without evidence of vasculitis. Immunofluorescent antibody staining and polymerase chain reaction for O. tsutsugamushi failed to demonstrate the organism in the lung tissue. The patient expired due to progressive respiratory failure despite doxycycline therapy. Immunologic mechanism, without direct invasion of the organism, may participate in the pathogenesis of ARDS associated with scrub typhus.

DOI: 10.3346/jkms.2000.15.3.343
PMCID: PMC3054636
PMID: 10895979 [PubMed - indexed for MEDLINE]

289. Am J Trop Med Hyg. 2002 Aug;67(2):162-5.

Scrub typhus in Japan: epidemiology and clinical features of cases reported in 1998.

Ogawa M(1), Hagiwara T, Kishimoto T, Shiga S, Yoshida Y, Furuya Y, Kaiho I, Ito T, Nemoto H, Yamamoto N, Masukawa K.

Author information:
(1)Department of Virology I, National Institute of Infectious Diseases, Tokyo, Japan. ogawam@nih.go.jp

Surveillance for scrub typhus was conducted in Japan in 1998 using a questionnaire. A total of 462 cases were reported. Scrub typhus occurred in both the fall and spring in the northern part of Honshu (the main island), and in the fall in the central part of Honshu and on the island of Kyushu. The occurrence of the disease varied with age, gender, and activity. Seventy-six percent of the patients were more than 51 years old, and 36% and 16% of the patients were

engaged in farm work and forestry, respectively. Fever, rash, and eschar were detected in 98%, 93%, and 97% of the patients, respectively. Elevated levels of C-reactive protein, aspartate transaminase, and alanine transaminase were detected in 96%, 87%, and 77% of the patients, respectively. Disseminated intravascular coagulation developed in 34 cases and had a unique regional distribution. This study shows the status of scrub typhus in Japan in 1998 and provides important information for diagnosis and prevention.

PMID: 12389941 [PubMed - indexed for MEDLINE]

290. Pediatr Neonatol. 2009 Jun;50(3):96-101. doi: 10.1016/S1875-9572(09)60043-7.

Clinical manifestations, laboratory findings and complications of pediatric scrub typhus in eastern Taiwan.

Jim WT(1), Chiu NC, Chan WT, Ho CS, Chang JH, Huang SY, Wu S.

Author information:
(1)Department of Pediatrics, Taitung Branch of Mackay Memorial Hospital, Taitung, Taiwan. waitim@ms1.mmh.org.tw

BACKGROUND: Scrub typhus is a clinically important endemic disease in Taiwan. The aims of this study were to analyze the clinical manifestations, laboratory data and complications of pediatric scrub typhus in eastern Taiwan.
PATIENTS AND METHODS: We searched medical records for all patients with scrub typhus who were hospitalized between 1992 and 2002 at the Taitung branch of Mackay Memorial Hospital, Taiwan. Records of children under the age of 18 with a confirmed diagnosis were selected for retrospective review.
RESULTS: During the study period, 145 patients fulfilled the diagnostic criteria for scrub typhus, of whom 106 (73%) were adults and 39 (27%) were children. The mean age of the children was 7.6+/-4.6 years. The most common clinical manifestations of pediatric scrub typhus were fever (n=39; 100%), cough (n=28; 72%), anorexia (72%), eschar (69%), chill (67%) and lymphadenopathy (64%). The most common complications were hepatic dysfunction (77%) and pneumonitis (54%). Three children (8%) required intensive care, but the overall survival rate was 97%. One child died with multi-organ failure within 8 hours after admission.
CONCLUSION: Scrub typhus should be considered in children with fever and hepatic dysfunction, particularly in those with a history of environmental exposure in an endemic area for scrub typhus. The presence of an eschar offers an important diagnostic clue, but not for all cases. Children with scrub typhus may develop serious complications and may even die if appropriate treatment is not given. Doxycycline is an effective antibiotic for pediatric scrub typhus in Taiwan.

DOI: 10.1016/S1875-9572(09)60043-7
PMID: 19579755 [PubMed - indexed for MEDLINE]

291. J Assoc Physicians India. 2010 Jan;58:11-2.

Diagnosis and treatment of scrub typhus--the Indian scenario.

Chogle AR.

PMID: 20649092 [PubMed - indexed for MEDLINE]

292. J Korean Med Sci. 2004 Oct;19(5):668-73.

Clinical role of interstitial pneumonia in patients with scrub typhus: a possible marker of disease severity.

Song SW(1), Kim KT, Ku YM, Park SH, Kim YS, Lee DG, Yoon SA, Kim YO.

Author information:
(1)Department of Radiology, College of Medicine, The Catholic University of Korea, Seoul, Korea.

Interstitial pneumonia (IP) frequently occurs in patients with scrub typhus, but its clinical significance is not well known. This study was designed to evaluate interstitial pneumonia as a marker of severity of the disease for patients with scrub typhus. We investigated clinical parameters representing the severity of the disease, and the chest radiographic findings for 101 patients with scrub typhus. We then compared these clinical factors between patients with and without IP. We also studied the relationship between IP and other chest radiographic findings. The chest radiography showed IP (51.4%), pleural effusion (42.6%), cardiomegaly (14.9%), pulmonary alveolar edema (20.8%), hilar lymphadenopathy (13.8%) and focal atelectasis (11.8%), respectively. The patients with IP (n=52) had higher incidences in episode of hypoxia (p=0.030), hypotension (p=0.024), severe thrombocytopenia (p=0.036) and hypoalbuminemia (p=0.013) than the patients without IP (n=49). The patients with IP also had higher incidences of pleural effusion (p<0.001), focal atelectasis (p=0.019), cardiomegaly (p<0.001), pulmonary alveolar edema (p=0.011) and hilar lymphadenopathy (p<0.001) than the patients without IP. Our data suggest that IP frequently occurs for patients with scrub typhus and its presence is closely associated with the disease severity of scrub typhus.

DOI: 10.3346/jkms.2004.19.5.668
PMCID: PMC2816328
PMID: 15483341 [PubMed - indexed for MEDLINE]

293. Med J Aust. 1999 Apr 5;170(7):318-20.

Scrub typhus in north Queensland.

McBride WJ(1), Taylor CT, Pryor JA, Simpson JD.

Author information:
(1)Department of Pathology, Cairns Base Hospital, QLD. mcbrij@health.qld.gov.au

Scrub typhus was once common in north Queensland, but no reports from this region have been published for nearly 30 years, and the focus has turned to cases from the Northern Territory and Western Australia. In 1996, diagnosis of scrub typhus in a Queensland soldier led to recognition of an earlier outbreak with up to 17 cases. Another outbreak occurred a year later with 11 confirmed cases. All cases

were in soldiers who had visited a training area near Innisfail. Review of other laboratory diagnoses of scrub typhus shows it is still prevalent in north Queensland, with several "hot spots".

PMID: 10327973 [PubMed - indexed for MEDLINE]

294. Am J Trop Med Hyg. 1983 Sep;32(5):1101-7.

Diagnostic criteria for scrub typhus: probability values for immunofluorescent antibody and Proteus OXK agglutinin titers.

Brown GW, Shirai A, Rogers C, Groves MG.

The sensitivities and specificities of the indirect microimmunofluorescent antibody (IFA) and Weil-Felix (OXK) tests for scrub typhus were established for a range of titers using groups of diseased and control (other febrile illnesses) patients diagnosed by other methods. At a cut-off point of greater than or equal to 1:400, the IFA test was 0.96 specific, and at greater than or equal to 1:320, the OXK was 0.97 specific. Using either these highly specific levels of antibody or other rigorous diagnostic criteria (isolation or 4-fold rising titers), the prevalence of scrub typhus infection was determined to be 0.22 in an unselected population of febrile patients in a rural Malaysian hospital. Probability values (Pr) for the correct diagnosis of scrub typhus were then calculated from the specificity, sensitivity and prevalence determination for a range of titers. The Pr for an OXK titer of greater than or equal to 1:320 was 0.79, and the Pr for an IFA titer of greater than or equal to 1:400 was 0.78. When both these titers were present in a single specimen, the Pr increased to 0.96.

PMID: 6414321 [PubMed - indexed for MEDLINE]

295. Ann Acad Med Singapore. 1997 Nov;26(6):794-800.

Scrub typhus in the Western Pacific region.

Silpapojakul K(1).

Author information:
(1)Department of Medicine, Prince of Songkla University, Songkla, Thailand.

Scrub typhus is widely endemic in Asia. Man's behaviour and climatic changes greatly influenced the occurrence of the disease. Increasing prevalences of scrub typhus have been reported from some Asian countries and may coincide with the widespread use of beta-lactam antibiotics or to improve diagnostic facilities and/or more urbanisation into rural areas. Many cases acquired in Asia surfaced in Europe and America. The disease probably is overlooked among paediatric patients. Most patients with scrub typhus present with acute fever of unknown origin (acute FUO). Eschars are rare among Southeast Asian patients. Complications usually develop after the first week of illness. The complications include pneumonitis, meningoencephalitis, renal failure and jaundice. Improved serologic and molecular diagnostic tests are now available. Although

drug-resistant strain of Orientia tsutsugamushi has been reported, the infection usually responds to simple but unpopular drugs such as doxycycline or chloramphenicol.

PMID: 9522982 [PubMed - indexed for MEDLINE]

296. Acta Trop. 2015 Mar;143:121-33. doi: 10.1016/j.actatropica.2014.10.012. Epub 2014 Oct 25.

Distribution of rickettsioses in Oceania: past patterns and implications for the future.

Derne B(1), Weinstein P(2), Musso D(3), Lau C(4).

Author information:
(1)Queensland Children's Medical Research Institute, Level 4 Foundation Building, Royal Children's Hospital, Herston Rd, Herston, Brisbane 4029, QLD, Australia; Barbara Hardy Institute, City East Campus, University of South Australia, GPO Box 2471, Adelaide 5000, SA, Australia. Electronic address: bonnie.derne@uqconnect.edu.au. (2)School of Pharmacy and Medicine, City East Campus, University of South Australia, GPO Box 2471, Adelaide, 5000, SA, Australia; School of Biological Sciences, North Terrace Campus, University of Adelaide, Adelaide 5000, SA, Australia. (3)Unit of Emerging Infectious Diseases, Institut Louis Malardé, BP 30, Pape'ete 98713, Tahiti, French Polynesia. (4)Queensland Children's Medical Research Institute, Level 4 Foundation Building, Royal Children's Hospital, Herston Rd, Herston, Brisbane 4029, QLD, Australia; Research School of Population Health, Australian National University, Acton 2601, ACT, Australia.

Rickettsioses present a threat to human health worldwide, but relatively little is known on their epidemiology and ecology in Oceania. These bacteria are the cause of potentially fatal febrile illnesses in humans (categorized into scrub typhus, typhus group and spotted fever group rickettsioses). They are transmitted by arthropod vectors such as ticks, mites, fleas and lice, which are associated with vertebrate host animals including rodents and companion animals. We conducted a search in the scientific and grey literature of Rickettsia spp. and Orientia tsutsugamushi within the Oceania region. Human case reports, human serosurveys and PCR-based testing of vectors and host animals reviewed here highlight the widespread distribution of these pathogens in the region, with the majority of human serological and vector surveys reporting positive results. These findings suggest that rickettsioses may have a significantly higher burden of disease in Oceania than is currently appreciated due to diagnostic challenges. Furthermore, consideration of the ecology and risk factors for rickettsioses reported for Oceania suggests that their importance as a cause of undifferentiated acute febrile illness may grow in the future: environmental and social changes driven by predicted climate change and population growth have the potential to lead to the emergence of rickettsioses as a significant public health problem in Oceania.

Copyright © 2014 Elsevier B.V. All rights reserved.

DOI: 10.1016/j.actatropica.2014.10.012

PMID: 25446172 [PubMed - indexed for MEDLINE]

297. Emerg Infect Dis. 2009 Oct;15(10):1659-61. doi: 10.3201/eid1510.090007.

Acute Q fever and scrub typhus, southern Taiwan.

Lai CH(1), Chen YH, Lin JN, Chang LL, Chen WF, Lin HH.

Author information:
(1)E-Da Hospital and I-Shou University, Kaoh siung City, Taiwan.

Acute Q fever and scrub typhus are zoonoses endemic to southern Taiwan. Among the 137 patients with acute Q fever (89, 65.0%) or scrub typhus (43, 31.4%), we identified 5 patients (3.6%) who were co-infected with Coxiella burnetii and Orientia tsutsugamushi.

DOI: 10.3201/eid1510.090007
PMCID: PMC2866387
PMID: 19861068 [PubMed - indexed for MEDLINE]

298. Vector Borne Zoonotic Dis. 2011 Jan;11(1):15-9. doi: 10.1089/vbz.2009.0186. Epub 2010 Jun 16.

Serological survey of five zoonoses, scrub typhus, Japanese spotted fever, tularemia, Lyme disease, and Q fever, in feral raccoons (Procyon lotor) in Japan.

Inoue K(1), Kabeya H, Fujita H, Makino T, Asano M, Inoue S, Inokuma H, Nogami S, Maruyama S.

Author information:
(1)Laboratory of Veterinary Public Health, Department of Veterinary Medicine, College of Bioresource Sciences, Nihon University, Fujisawa, Kanagawa, Japan.

We investigated the seroprevalence of five tick- or mite-borne zoonoses, scrub typhus (Orientia tsutsugamushi), Japanese spotted fever (Rickettsia japonica), tularemia (Francisella tularensis), Lyme disease (Borrelia afzelii and Borrelia garinii), and Q fever (Coxiella burnetii), in feral raccoons (Procyon lotor) captured in Hokkaido and Kanagawa Prefectures in Japan. Of the 559 raccoons captured in Hokkaido, 8 (1.4%), 3 (0.5%), 1 (0.2%), and 1 (0.2%) carried antibodies against O. tsutsugamushi (Gilliam type), F. tularensis, B. afzelii, and B. garinii, respectively. Of the 193 animals investigated in Kanagawa, 31 (16.1%) and 14 (7.3%) carried antibodies against O. tsutsugamushi and R. japonica, respectively, and the major serotype (27/31) of O. tsutsugamushi was Kuroki. No antibodies against C. burnetii were detected in either area examined. Therefore, feral raccoons could be an indicator of the prevalence of these four tick- or mite-borne zoonoses in the peridomestic environment in Japan.

DOI: 10.1089/vbz.2009.0186
PMID: 20553108 [PubMed - indexed for MEDLINE]

299. Pediatr Infect Dis J. 2003 Feb;22(2):130-3.

Roxithromycin treatment of scrub typhus (tsutsugamushi disease) in children.

Lee KY(1), Lee HS, Hong JH, Hur JK, Whang KT.

Author information:
(1)Department of Pediatrics, College of Medicine, The Catholic University of Korea, Seoul, Republic of Korea. leekyungyil@yahoo.com

BACKGROUND: Although chloramphenicol and doxycycline have been used for the treatment of scrub typhus, a difficulty exists in determining which drug to use in treating children because of such potential complications as aplastic anemia or tooth discoloration. We evaluated the effect of roxithromycin, a macrolide antibiotic, on scrub typhus in children.
METHODS: A retrospective analysis was conducted on 39 children with scrub typhus who were treated with doxycycline (DC), chloramphenicol (CM) or roxithromycin (RM) between 1991 and 2000. We divided the patients into the DC-treated group (DC group; 16 children), CM-treated group (CM group; 14 children) and RM- treated group (RM group; 9 children) and compared these groups.
RESULTS: Most cases (97%) occurred in October and November. Fever and rash were observed in all 39 cases, and an eschar was noted in 36 cases (92%). No statistical differences could be found between the 3 groups in mean age, duration of fever before admission, white blood cell (WBC) count and complications including abnormal liver enzymes. In most cases defervescence after treatment was within 24 h (34 cases, 87%) and during 24 to 48 h in 2 cases in the DC group, 1 case in the CM group and 2 cases in the RM group (no statistical difference).
CONCLUSION: Roxithromycin was as effective as conventional doxycycline or chloramphenicol in children with scrub typhus and may be safer to use.

DOI: 10.1097/01.inf.0000047864.80791.20
PMID: 12586976 [PubMed - indexed for MEDLINE]

300. Emerg Infect Dis. 1998 Oct-Dec;4(4):641-4.

New Orientia tsutsugamushi strain from scrub typhus in Australia.

Odorico DM(1), Graves SR, Currie B, Catmull J, Nack Z, Ellis S, Wang L, Miller DJ.

Author information:
(1)James Cook University, Townsville, Queensland, Australia.

In a recent case of scrub typhus in Australia, Orientia tsutsugamushi isolated from the patient's blood was tested by sequence analysis of the 16S rDNA gene. The sequence showed a strain of O. tsutsugamushi that was quite different from the classic Karp, Kato, and Gilliam strains. The new strain has been designated Litchfield.

DOI: 10.3201/eid0404.980416
PMCID: PMC2640248
PMID: 9866742 [PubMed - indexed for MEDLINE]

301. Scand J Infect Dis. 1997;29(6):634-35.

Scrub typhus associated with multiorgan failure: a case report.

Chi WC(1), Huang JJ, Sung JM, Lan RR, Ko WC, Chen FF.

Author information:
(1)Department of Internal Medicine, Yuan's General Hospital, Kaohsiung, Taiwan.

The spectrum of clinical severity for scrub typhus ranges from inapparent, mild, to severe or fatal. The pathologic changes are focal or disseminated multiorgan vasculitis of the small blood vessels, a fact that helps explain the great diversity of clinical manifestations that can be encountered. We reported a case of scrub typhus with unusual and serious multiorgan involvement, including tubulointerstitial nephritis (TIN) with acute renal failure (ARF), interstitial pneumonitis with adult respiratory distress syndrome (ARDS), disseminated intravascular coagulation (DIC), liver function impairment, upper gastrointestinal bleeding, prolonged hyperamylasaemia and hyperlipasaemia. Chloramphenicol administration rapidly altered the clinical course, but with sequelae of renal impairment and prolonged hyperamylasaemia and hyperlipasaemia for 10 months.

PMID: 9571750 [PubMed - indexed for MEDLINE]

302. Trans R Soc Trop Med Hyg. 1977;71(4):338-42.

Epidemiological and serological study of scrub typhus among Chinese military in the Pescadores islands of Taiwan.

Bourgeois AL, Olson JG, Ho CM, Fang RC, Van Peenen PF.

An outbreak of 69 cases of scrub typhus occurred among Chinese military personnel stationed in the Pescadores Islands, Taiwan Province, Republic of China between May and November 1975. A retrospective epidemiological study of this outbreak indicated that military personnel over 40 were more likely to have scrub typhus than those under 40. High risk groups included the Garrison Force (home guard), anti-aircraft gunners and infantry and armoured units stationed at Hsing-jen. The onset of symptoms in 69% occurred within one year of residence in the Pescadores. The clinical course of scrub typhus and the serological response to infection were also studied. Eschar formation, fever, headache chills and lymph node enlargement were the predominant clinical manifestations noted. The indirect immunofluorescent antibody test (IFAT) demonstrated diagnostic (four-fold) rises in antibody titres to Rickettsia tsutsugamushi reference strains in 36 of 41 paired sera tested. 11 of 19 patients from whom only single sera were obtained had IFA titres presumptive of scrub typhus (greater than or equal to 1:160). Of 19 patients experiencing possible primary infections, 13 (68%) responded with antibodies directed against more than one reference strain of R. tsutsugamushi. These results suggest that several antigenically diverse strains of R. tsutsugamushi may be active in the Pescadores.

PMID: 413217 [PubMed - indexed for MEDLINE]

303. J Infect Public Health. 2014 Sep-Oct;7(5):450-2. doi: 10.1016/j.jiph.2014.04.005.
Epub 2014 Jun 20.

Lost in a haystack: the importance of physical re-examination.

Jain D(1), Viswanathan S(2), Shanmugam J(3).

Author information:
(1)Department of General Medicine, Indira Gandhi Medical College & RI,
Pondicherry 605009, India. Electronic address: dhirajlunawat@yahoo.com.
(2)Department of General Medicine, Indira Gandhi Medical College & RI,
Pondicherry 605009, India. Electronic address: stalinviswanathan@ymail.com.
(3)Department of General Medicine, Indira Gandhi Medical College & RI,
Pondicherry 605009, India. Electronic address: jagatheeswari@gmail.com.

We report an unusual case of varicella zoster in a 19-year-old, who presented with persisting fever even after her lesions began forming scabs. Fever workup for secondary complications was negative. A re-examination revealed an axillary eschar. She recovered completely with doxycycline. This report suggests the importance of keeping in mind, epidemiological data of common diseases in the locality, as well as the significance of a thorough physical examination in patients with fever.

Copyright © 2014 King Saud Bin Abdulaziz University for Health Sciences.
Published by Elsevier Ltd. All rights reserved.

DOI: 10.1016/j.jiph.2014.04.005
PMID: 24954067 [PubMed - indexed for MEDLINE]

304. BMC Infect Dis. 2009 Oct 22;9:173. doi: 10.1186/1471-2334-9-173.

Acute sensorineural hearing loss and severe otalgia due to scrub typhus.

Kang JI(1), Kim DM, Lee J.

Author information:
(1)Division of Infectious Diseases, Department of Internal Medicine, Chosun
University, School of Medicine, Gwangju City, Republic of Korea. jeankk@naver.com

BACKGROUND: Scrub typhus is an acute febrile illness caused by Orientia tsutsugamushi.
CASE PRESENTATIONS: We encountered a patient with sensorineural hearing loss complicating scrub typhus, and three patients with scrub typhus who complained of otalgia, which was sudden onset, severe, paroxysmal, intermittent yet persistent pain lasting for several seconds, appeared within 1 week after the onset of fever and rash. The acute sensorineural hearing loss and otalgia were resolved after antibiotic administration.
CONCLUSION: When patients in endemic areas present with fever and rash and have sensorineural hearing loss or otalgia without otoscopic abnormalities, clinicians should suspect scrub typhus and consider empirical antibiotic therapy.

DOI: 10.1186/1471-2334-9-173
PMCID: PMC2773783
PMID: 19849842 [PubMed - indexed for MEDLINE]

305. Am J Trop Med Hyg. 2007 May;76(5):801-5.

Key differentiating features between scrub typhus and hemorrhagic fever with renal syndrome in northern China.

Liu YX(1), Feng D, Zhang Q, Jia N, Zhao ZT, De Vlas SJ, Li J, Zhang PH, Yang H, Min JS, Feng PT, Ma SB, Cao WC.

Author information:
(1)Beijing Institute of Microbiology and Epidemiology, State Key Laboratory of Pathogen and Biosecurity, Beijing, People's Republic of China.

Both scrub typhus and hemorrhagic fever with renal syndrome (HFRS) are severely epidemic in northern China and often present with acute undifferentiated fever. To correctly distinguish the two diseases at an early stage, we collected and compared clinical and routine laboratory data of 46 patients with confirmed scrub typhus and 49 patients with confirmed HFRS presenting to the outpatient departments of three town hospitals in northern China. Most patients with HFRS but none of the patients with scrub typhus had hemorrhagic manifestations. Retro-orbital pain, lumbar back pain, flank tenderness, proteinuria, and occult blood in urine often occurred in patients with HFRS. However, skin eschar, regional lymphadenopathy, and maculopapular rash were more commonly found in patients with scrub typhus. In addition, platelet counts in patients with HFRS were significantly lower than in patients with scrub typhus. These findings will be useful for physicians to distinguish scrub typhus from HFRS.

PMID: 17488894 [PubMed - indexed for MEDLINE]

306. Kansenshogaku Zasshi. 2008 Jul;82(4):335-40.

[Tsutsugamushi disease during the last seven years in the past in Hamamatsu City, Shizuoka Prefecture--including evaluation of hyponatremia in scrub typhus].

[Article in Japanese]

Shichi D(1), Tanizawa T, Honda K.

Author information:
(1)Department of General Internal Medicine, Seirei Mikatahara General Hospital.

Six cases of scrub typhus (tsutsugamushi disease) were reported to the Shizuoka Prefecture Hamamatsu City public health center during the seven years from 2001 to 2007. The content of the clinical record of the five cases were investigated. High serum titers of antibody to Gilliam-type Orientia tsutsugamushi were detected by immunofluorescense assay in most of these patients. Fever, rash, headache and relative bradycardia seen at a high frequency. On peripheral blood smear examination, atypical lymphocytes were detected in 3 cases. Serum

electrolyte examination revealed hyponatremia in 4 (80%) patients; SIADH was suspected in one of these cases. All the patients improved promptly following start of therapy with intravenous or oral minocycline.

PMID: 18697486 [PubMed - indexed for MEDLINE]

307. Am J Trop Med Hyg. 2009 Sep;81(3):484-8.

Risk factors leading to fatal outcome in scrub typhus patients.

Lee CS(1), Hwang JH, Lee HB, Kwon KS.

Author information:
(1)Departments of Internal Medicine, Preventive Medicine, Chonbuk National University Medical School, and Research Institute of Clinical Medicine, Jeonju, Republic of Korea. lcsmd@chonbuk.ac.kr

Scrub typhus is a potentially fatal infectious disease. However, to this date, no epidemiologic study on mortality of complicated scrub typhus has been reported. We reviewed the clinical records of 302 patients with diagnosis of scrub typhus who were admitted to our institute between January 2000 and December 2006. In total, 297 patients with scrub typhus were analyzed and the mortality rate of this study group was 6.1%. Multivariate logistic regression analysis revealed absence of eschar, event of intensive care unit admission and higher APACHE II score were independent predictive variables. Identification of these risk factors leading to fatal outcome may help physicians to start early intensive management of complicated scrub typhus.

PMID: 19706919 [PubMed - indexed for MEDLINE]

308. Southeast Asian J Trop Med Public Health. 2003 Dec;34(4):826-30.

Epidemiological studies on host animals of scrub typhus of the autumn-winter type in Shandong Province, China.

Liu Y(1), Zhao Z, Yang Z, Zhang J, Xu J, Wu Q, Peng Z, Miao Z.

Author information:
(1)Medical Institute of Jinan Military Command, PLA, Jinan, China.

In order to elucidate the host animals of scrub typhus in Shandong Province, epidemiological studies on host rodents of the autumn-winter type scrub typhus were carried out from 1995 to 2002 at four localities in the Shandong Province. Based upon ecological observations of the composition, seasonal fluctuation of animal hosts, isolation of Orientia tsutsugamushi, detection and identification of serotypes of antibodies to O. tsutsugamushi were conducted. Two thousand eight hundred and eighty-four rodents and insectivores were captured, including 2,055 Apodemus agrarius (71.26%), 408 Cricetulus triton (14.15%), 64 C. barabensis (2.22%), 12 Crocidura suaveolens (0.42%), 313 Rattus (R.) norvegicus (10.85%), 32 Mus (M.) musculus (1.11%). A. agrarius was predominant in the field and the

seasonal fluctuation was correlated significantly to that of scrub typhus (r=0.810, p<0.005). R. norvegicus was predominant indoors. The average capture rate per year in the field was 12.76% from 1995 to 1997. Of the total 2,884 rodents and insectivores captured out- and in-doors, 527 were living rodents (including 335 A. agrarius, 119 C. triton, 6 C. barabensis, 2 C. suaveolens, 63 R. norvegicus and 2 M. musculus, and 15,467 chigger mites were collected from them. Two hundred and fifty-three of 335 A. agrarius were parasitized by chiggers, showing 75.52% (253/335) of the infestation rate and 17.53 of the chigger index; 106 C. triton were parasitized by chiggers, showing 89.08% (106/119) infestation rate and 75.93 of the chigger index. The average antibody positive rate of rodents was 14.78%. The seasonal change of the antibody positive rate was higher during December-February (the second year), and varied from 20% to 28%, but the level of antibodies remained relatively low (5.26-16.67%) during March-November. The results of serotyping with 47 antibody-positive sera were as followings: 39 sera were Gilliam types, 7 sera were Karp types, 1 serum was Kato type. Twelve strains of O. tsutsugamushi were isolated from A. agrarius (8 strains), C. triton (3 strains) and R. norvegicus (1 strain), out of the isolated 12 strains, 10 were Gilliam strains, 2 were Karp strains. A. agrarius and R. norvegicus were the main host animals in out- and indoors respectively.

PMID: 15115095 [PubMed - indexed for MEDLINE]

309. J Assoc Physicians India. 2005 Nov;53:954-8.

Scrub typhus.

Mahajan SK(1).

Author information:
(1)Department of Medicine, I G Medical College, Shimla (H P).

Scrub typhus, a dreaded disease in pre-antibiotic era, is an important military disease which caused thousands of cases in the Far East during Second World War. It is a zoonosis and is a widespread disease in Asia and Pacific Islands. Scrub typhus is an acute febrile illness which generally causes non-specific symptoms and signs. The clinical manifestations of this disease range from sub-clinical disease to organ failure to fatal disease. Deaths are attributable to late presentation, delayed diagnosis, and drug resistance. The public health importance of this disease is underestimated because of difficulties with clinical diagnosis and lack of laboratory methods in many geographical areas. Scrub typhus is known to occur all over India and physicians should be aware of this potentially serious but easily treatable disease.

PMID: 16515236 [PubMed - indexed for MEDLINE]

310. J Formos Med Assoc. 2009 May;108(5):367-76. doi: 10.1016/S0929-6646(09)60080-2.

Epidemiology of acute q Fever, scrub typhus, and murine typhus, and identification of their clinical characteristics compared to patients with acute febrile illness in southern taiwan.

Lai CH(1), Huang CK, Chen YH, Chang LL, Weng HC, Lin JN, Chung HC, Liang SH, Lin HH.

Author information:
(1)Division of Infectious Diseases, Department of Internal Medicine, E-Da Hospital/I-Shou University, Taiwan.

BACKGROUND/PURPOSE: In Taiwan, acute Q fever, scrub typhus, and murine typhus (QSM diseases) are the most common rickettsioses, but their epidemiology and clinical characteristics have not been clarified. Diagnosis of these three diseases based on clinical manifestations is difficult, and most of their reported characteristics are identified by describing the predominant manifestations, without being compared with other diseases.
METHODS: Serological tests for QSM diseases were examined simultaneously in patients suspected of the three diseases, regardless of which one was suspected. Clinical manifestations were recorded retrospectively from their charts. The characteristics of QSM diseases were identified by comparison with patients who had non-QSM diseases.
RESULTS: From April 2004 to April 2007, a total of 226 cases of suspected QSM diseases were included. One hundred (44.2%) cases were serologically confirmed as QSM diseases (68 acute Q fever, 23 scrub typhus, and 9 murine typhus), and 126 (55.8%) cases were non-QSM diseases. Only 33 cases (33.0%) of QSM diseases were initially suspected at the time of hospital visit, whereas 54 cases (42.9%) of non-QSM diseases were incorrectly suspected as QSM diseases. Cases of Q fever and scrub typhus were distributed over plain and mountain areas, respectively. By multivariate analysis, relative bradycardia (OR [95% CI], 2.885 [1.3-6.4]; p = 0.009), radiographic hepatomegaly (OR [95% CI], 4.454 [1.6-12.3]; p = 0.004), and elevated serum aminotransferases (OR [95% CI], 5.218 [1.2-23.1]; p = 0.029) were independent characteristics for QSM diseases, and leukocytosis (OR [95% CI], 0.167 [0.052-0.534]; p = 0.003) was negative for the diagnosis of QSM diseases.
CONCLUSION: In southern Taiwan, acute Q fever is the most common rickettsiosis. QSM diseases should be suspected in febrile patients who present with relative bradycardia, hepatomegaly, and elevated serum aminotransferases, but without leukocytosis.

DOI: 10.1016/S0929-6646(09)60080-2
PMID: 19443290 [PubMed - indexed for MEDLINE]

311. Am J Trop Med Hyg. 2005 Nov;73(5):936-41.

Protection against scrub typhus by a plasmid vaccine encoding the 56-KD outer membrane protein antigen gene.

Ni YS(1), Chan TC, Chao CC, Richards AL, Dasch GA, Ching WM.

Author information:
(1)Rickettsial Diseases Department, U.S. Naval Medical Research Center, Silver Spring, Maryland 20910, USA.

The 56-kD outer membrane protein of Orientia tsutsugamushi has previously been shown to be the immunodominant antigen in scrub typhus infections. Its gene was cloned into the DNA vaccine vector pVR1012 as a vaccine candidate (pKarp56). The

in vitro expression of this 56-kD antigen by pKarp56 was confirmed in tissue culture by an indirect fluorescence assay and Western blot analysis. The initial antibody responses of mice immunized with varied doses of the pKarp56 were barely detected, but increases were observed after each of three subsequent booster immunizations. Although no protection was observed with a single immunization of pKarp56, after four immunizations, 60% of the mice survived a 1,000 x 50% lethal dose (LD(50)) challenge. These results specifically confirm the importance of the 56-kD protein antigen in protective immunity against O. tsutsugamushi and demonstrate the feasibility of DNA vaccines for the prevention of scrub typhus.

PMID: 16282307 [PubMed - indexed for MEDLINE]

312. J Microbiol Immunol Infect. 2003 Dec;36(4):285-7.

Acute acalculous cholecystitis and pancreatitis in a patient with concomitant leptospirosis and scrub typhus.

Wang NC(1), Ni YH, Peng MY, Chang FY.

Author information:
(1)Division of Infectious Diseases and Tropical Medicine, Department of Internal Medicine, Tri-Service General Hospital, National Defense Medical Center, Taipei, Taiwan, ROC.

Concomitant leptospirosis and scrub typhus is rare. The spectrum of clinical severity for both scrub typhus and leptospirosis ranges from mild to fatal. Acute pancreatitis and cholecystitis are infrequent complications in adult patients with either leptospirosis or scrub typhus. We report a case of leptospirosis and scrub typhus coinfection in a 41-year-old man presenting with acute acalculous cholecystitis, pancreatitis and acute renal failure. Abdominal computed tomography revealed edematous change of the gallbladder without intrahepatic or pancreatic lesions. The patient was successfully treated with doxycycline and ceftriaxone, and supportive management.

PMID: 14723261 [PubMed - indexed for MEDLINE]

313. Clin Infect Dis. 2000 Jul;31(1):191-2.

Multiple organ failure complicating probable scrub typhus.

Cracco C(1), Delafosse C, Baril L, Lefort Y, Morelot C, Derenne JP, Bricaire F, Similowski T.

Author information:
(1)Service de Pneumologie et Réanimation, Département des Maladies Infectieuses et Tropicales, Parasitologie et Santé Publique, 75651 Paris Cedex 13, France.

This report describes a case of life-threatening acute respiratory distress syndrome with multiple organ failure complicating probable scrub typhus. Favorable outcome was associated with fluoroquinolone therapy. Scrub typhus

should be suspected in travelers returning from Southeast Asia presenting with unexplained respiratory manifestations.

DOI: 10.1086/313906
PMID: 10913423 [PubMed - indexed for MEDLINE]

314. Jpn J Infect Dis. 2000 Apr;53(2):77-8.

Occurrence of scrub typhus (Tsutsugamushi) in Kanagawa Prefecture and types of Orientia tsutsugamushi involved.

Furuya Y(1), Katayama T, Hara M, Yoshida Y, Imai M, Hagiwara T.

Author information:
(1)Kanagawa Prefectural Public Health Laboratory, Kanagawa 241-0815, Japan.

PMID: 10871922 [PubMed - indexed for MEDLINE]

315. Am J Trop Med Hyg. 1997 Jul;57(1):91-5.

Seroepidemiologic evidence for murine and scrub typhus in Malang, Indonesia.

Richards AL(1), Soeatmadji DW, Widodo MA, Sardjono TW, Yanuwiadi B, Hernowati TE, Baskoro AD, Roebiyoso, Hakim L, Soendoro M, Rahardjo E, Putri MP, Saragih JM, Strickman D, Kelly DJ, Dasch GA, Olson JG, Church CJ, Corwin AL.

Author information:
(1)U.S. Naval Medical Research Unit No. 2, Jakarta, Indonesia.

Indonesian military personnel stationed in Malang, East Java were among troops deployed to central Cambodia as part of the United Nations' Transition Authority Cambodia peace-keeping operation in 1992. Predeployment blood samples obtained from a cohort of Indonesian soldiers indicated a high prevalence of antibodies to antigens of Rickettsia typhi or Orientia (formerly Rickettsia) tsutsugamushi, the etiologic agents for murine and scrub typhus, respectively. To evaluate the potential risk of these rickettsial diseases in the Malang area, a subsequent seroepidemiologic survey was conducted. This study involved civilian personnel residing within one of three Malang kelurahans (neighborhoods) representing urban, suburban, and rural communities. The heads-of-households from 197 homes completed a detailed epidemiologic survey. In addition, blood samples were collected from 464 individuals residing within the households surveyed. Examination of civilian blood samples disclosed that 34.7% and 1.3% of the study participants were seroreactive to R. typhi and O. tsutsugamushi, respectively. These results were similar to those obtained earlier from the military samples. In addition, assessment of 78 blood samples obtained from peridomestic rodents trapped from within or near the households surveyed showed that 28 were reactive to R. typhi antigens and four were reactive to O. tsutsugamushi antigens. These data indicate that military and civilian personnel living in the Malang area of East Java are at risk of infection with rickettsiae that are antigenically indistinguishable from those that cause murine and scrub typhus.

PMID: 9242326 [PubMed - indexed for MEDLINE]

316. Trans R Soc Trop Med Hyg. 2010 Apr;104(4):309-10. doi:
10.1016/j.trstmh.2009.08.011. Epub 2009 Sep 17.

Scrub typhus mimicking enteric fever; a report of three patients.

Premaratna R(1), Nawasiwatte BM, Niriella MA, Chandrasena TG, Bandara NK,
Rajapakse RP, de Silva HJ.

Author information:
(1)Department of Medicine, University of Kelaniya, P.O. Box 6, Thalagolla Road,
Ragama, Sri Lanka. ranjan premaratna@lycos.com

We report three patients who presented with fever and late onset diarrhoea
mimicking enteric fever. All three patients were diagnosed with an Orientia
tsutsugamushi infection and responded dramatically to doxycycline treatment.
Clinicians practicing in rickettsial disease endemic areas should be made aware
of similar clinical presentations in order to prevent morbidity and mortality
associated with rickettsioses.

Copyright 2009 Royal Society of Tropical Medicine and Hygiene. Published by
Elsevier Ltd. All rights reserved.

DOI: 10.1016/j.trstmh.2009.08.011
PMID: 19762057 [PubMed - indexed for MEDLINE]

317. Diagn Microbiol Infect Dis. 2008 Feb;60(2):237-9. Epub 2007 Nov 7.

Acute transverse myelitis associated with scrub typhus: case report and a review
of literatures.

Lee KL(1), Lee JK, Yim YM, Lim OK, Bae KH.

Author information:
(1)Department of Rehabilitation Medicine, Gachon University of Medicine and
Science, Incheon 405-760, Korea.

We describe a rare case of acute transverse myelitis associated with scrub
typhus. Magnetic resonance imaging scans, clinical characteristics, skin biopsy,
and cerebrospinal fluid cytology confirmed a diagnosis of acute transverse
myelitis associated with scrub typhus. To our knowledge, this was not seen in a
prior publication. Our case indicates that Orientia tsutsugamushi can invade
spinal cord.

DOI: 10.1016/j.diagmicrobio.2007.09.015
PMID: 17997258 [PubMed - indexed for MEDLINE]

318. Jpn J Infect Dis. 2006 Aug;59(4):235-8.

Epidemiology of scrub typhus in eastern Taiwan, 2000-2004.

Lee YS(1), Wang PH, Tseng SJ, Ko CF, Teng HJ.

Author information:
(1)The Sixth Branch Office, Center for Disease Control, Taipei, Taiwan.

The epidemiology of scrub typhus in eastern Taiwan was studied by analyzing the data from the CDC Web reporting system. A total of 1,396 cases with 403 confirmed cases were reported in the period of 2000 to 2004. The cases were commonly found in all counties with the highest number in Yuli Township, Hualien County (53 cases) and Taitung City, Taitung County (40 cases). Monthly changes in the number of cases showed epidemic periods in the spring with a peak in May, and again in the fall, with an October-November peak. The occurrence of disease varied with age, gender, and occupation. Our results showed that the infection rates in the elderly (50-69 years old), males (62.8%), and farmers (25.6%) were higher than those in other age groups, females, and other occupations. Five major clinical symptoms, fever, headache, eschar, rash, and lymphadenopathy, were observed in 90.1, 61.9, 23.1, 21.6, and 10.7% of the cases, respectively. Almost 90% (89.3%) of the cases showed 1-3 clinical symptoms and some showed 4-5 symptoms (10%). Only one patient with no symptoms (0.8%) was found. This paper reports the status of scrub typhus in eastern Taiwan, and suggests that a health education program could train individuals to self-recognize the disease symptoms.

PMID: 16936341 [PubMed - indexed for MEDLINE]

319. J Med Entomol. 2004 Jan;41(1):107-14.

Mite vectors (Acari: Trombiculidae) of scrub typhus in a new endemic area in northern Kyoto, Japan.

Takahashi M(1), Misumi H, Urakami H, Nakajima S, Furui S, Yamamoto S, Furuya Y, Misumi M, Matsumoto I.

Author information:
(1)Department of Anesthesiology, Saitama Medical School, Moroyama-machi, Iruma-gun, 350-0495 Japan. mamoru0917@hotmail.com

Between 1983 and 1999, 27 human cases of scrub typhus (two fatal) occurred in the Nodagawa River basin of northern Kyoto, Japan, an area where no cases had been previously reported. Antibody screening of infected patients' sera showed that nine of 15 patients had high titers against the Gilliam type of Orientia tsutsugamushi (Hayashi). To determine the vector mite transmitting the disease, we studied rodent and chigger populations in and near a rice field alongside the Nodagawa River between 1996 and 1999. The most common rodent species was Microtus montebelli (Milne-Edwards), representing 73.3% (33/45) of the population. The mite index (average number of mites per infested host) was highest (190.8) in Leptotrombidium pallidum Nagayo, Mitamura & Tamiya parasitizing on M. montebelli, followed by Leptotrombidium intermedium (Nagayo, Mitamura & Tamiya) (174.9) on the same host species. Orientia tsutsugamushi was isolated from 60.5% (23/38) of rodents and from 71.2% (37/52) of pools of engorged L. pallidum. The Gilliam type of O. tsutsugamushi was most prevalent in rodents, and in engorged L. pallidum

and it was the only type recovered from 10 isolates inoculated into L 929 cells for indirect immunofluorescence examination. Orientia tsutsugamushi infected 14.3% (181/1263) and 14.8% (306/2066) of engorged and unfed L. pallidum larvae, respectively, and was also detected in 0.055% (2/3634) of unfed L. intermedium, although previous studies suggest that this mite rarely bites humans. These results show that L. pallidum is the primary vector species of scrub typhus in this new endemic area in Japan.

PMID: 14989353 [PubMed - indexed for MEDLINE]

320. J Infect. 2006 Jan;52(1):56-60.

Scrub typhus among hospitalised patients with febrile illness in South India: magnitude and clinical predictors.

Varghese GM(1), Abraham OC, Mathai D, Thomas K, Aaron R, Kavitha ML, Mathai E.

Author information:
(1)Christian Medical College, Vellore 632004, India. georgemvarghese@hotmail.com

OBJECTIVES: To derive a clinical algorithm for diagnosis of scrub typhus among patients hospitalized with febrile illness and to determine predictors of bad prognosis.
METHODS: Patients hospitalized with febrile illness of 5-30 days duration were evaluated for common aetiologies. Sera were tested for antibodies (IgM and IgG) to Orientia tsutsugamushi using ELISA kit.
RESULTS: Among 207 patients, 50 had elevated levels of IgM antibodies. The data of these patients were compared with that of 16 controls having febrile illnesses in whom rickettsial infection was ruled out. Transaminase elevation (>twice normal) was present in 90% and was significantly (P=0.004) more common in those with scrub typhus. If a combination of elevated transaminases, thrombocytopenia and leukocytosis is used, the specificity and positive predictive value are about 80%. Case fatality rate was 14%. Univariate analysis showed that hyperbilirubinemia (>1.5mg%) has a RR of 9 (95% CI=1.48-58.5) and elevated creatinine level (>1.4 mg%) had a RR of 43.99 (95% CI=3.65-530.5) for death. Elevated creatinine level was found to be an independent predictor of mortality (P=0.02).
CONCLUSION: In developing countries with limited diagnostic facilities, it is prudent to recommend empiric therapy in patients with undifferentiated febrile illness having evidence of multiple system involvement especially if there is transaminase elevation. Elevated creatinine may predict bad outcome.

DOI: 10.1016/j.jinf.2005.02.001
PMID: 16368461 [PubMed - indexed for MEDLINE]

321. J Commun Dis. 2004 Dec;36(4):277-83.

Outbreak investigation of scrub Typhus in Himachal Pradesh (India).

Kumar K(1), Saxena VK, Thomas TG, Lal S.

Author information:
(1)National Institute of Communicable Diseases, 22 Sham Nath Marg,Delhi-110 054.

Scrub Typhus outbreak investigation was undertaken during September 2003 in the three worst affected districts Shimla, Solan and Sirmaur in Himachal Pradesh (India). A total of 113 cases and 19 deaths (17.27 percent case fatality rate) were reported from the eight districts, which were reporting cases. Cases were prevalent in all age groups and in both the sexes among the persons frequenting forest for occupational work. 35.7 percent of the patients serum samples showed a titer of > 1: 80 against OX 19 and OXK antigen is suggestive of Scrub Typhus. Entomologically Rattus rattus (39.5 per cent) was the most prevalent species followed by Suncus murinus (22.91 percent), Bandicoota bengalensis (29.16 percent) and Bandicoota indica and Mus musculus (4.16 percent each). Vector species Leptotrombidium deliense and Gahrliepia (schoengastilla) sp. were recorded. The chigger index 23.0 was found to be highest in Vill. Baldian and 5.0 in Vill. Bhatakuffer (Shimla) and 1.0 in Vill. Rebon (Solan), which is above the critical limit of 0.69 per rodents. Other mite species, ixodid ticks, fleas and lice have been recorded.

PMID: 16506551 [PubMed - indexed for MEDLINE]

322. J Clin Microbiol. 2010 Dec;48(12):4404-9. doi: 10.1128/JCM.01526-10. Epub 2010 Oct 6.

Isolation of a novel Orientia species (O. chuto sp. nov.) from a patient infected in Dubai.

Izzard L(1), Fuller A, Blacksell SD, Paris DH, Richards AL, Aukkanit N, Nguyen C, Jiang J, Fenwick S, Day NP, Graves S, Stenos J.

Author information:
(1)The Australian Rickettsial Reference Laboratory/Barwon Biomedical Research, The Geelong Hospital, Bellarine Street, Geelong, Victoria,Australia.

In July 2006, an Australian tourist returning from Dubai, in the United Arab Emirates (UAE), developed acute scrub typhus. Her signs and symptoms included fever, myalgia, headache, rash, and eschar. Orientia tsutsugamushi serology demonstrated a 4-fold rise in antibody titers in paired serum collections (1:512 to 1:8,192), with the sera reacting strongest against the Gilliam strain antigen. An Orientia species was isolated by the in vitro culture of the patient's acute blood taken prior to antibiotic treatment. The gene sequencing of the 16S rRNA gene (rrs), partial 56-kDa gene, and the full open reading frame 47-kDa gene was performed, and comparisons of this new Orientia sp. isolate to previously characterized strains demonstrated significant sequence diversity. The closest homology to the rrs sequence of the new Orientia sp. isolate was with three strains of O. tsutsugamushi (Ikeda, Kato, and Karp), with a nucleotide sequence similarity of 98.5%. The closest homology to the 47-kDa gene sequence was with O. tsutsugamushi strain Gilliam, with a nucleotide similarity of 82.3%, while the closest homology to the 56-kDa gene sequence was with O. tsutsugamushi strain TA686, with a nucleotide similarity of 53.1%. The molecular divergence and geographically unique origin lead us to believe that this organism should be considered a novel species. Therefore, we have proposed the name "Orientia

chuto," and the prototype strain of this species is strain Dubai, named after the location in which the patient was infected.

DOI: 10.1128/JCM.01526-10
PMCID: PMC3008486
PMID: 20926708 [PubMed - indexed for MEDLINE]

323. Am J Trop Med Hyg. 2007 Mar;76(3):553-8.

Preparation of recombinant antigen of O. tsutsugamushi Ptan strain and development of rapid diagnostic reagent for scrub typhus.

Cao M(1), Guo H, Tang T, Wang C, Li X, Pan X, Jin Z, Tang J.

Author information:
(1)Department of Epidemiology and Microbiology, Research Institute for Medicine of Nanjing Command, Jiangsu, China.

Spring scrub typhus has frequently occurred in Pingtan Island, China, since 2000. In this study, we amplified a 1352-bp DNA fragment encoding a truncated 56-kDa outer membrane protein of the Ptan strain, which was isolated from a serum sample of a patient with spring scrub typhus, and cloned it into the pET28a vector for expression. The expression product was a recombinant polypeptide containing a His-tag to facilitate purification on a Ni2+ chromatography column. The recombinant protein was further identified by Western blotting and enzyme-linked immunosorbent assay (ELISA) and appeared to be a good diagnostic antigen candidate. A rapid colloidal gold immunochromatographic assay (CIA) for detecting serum total antibodies, IgG and IgM, which are anti-Orientia tsutsugamushi, was developed, using a mixture of the r56 of the Gilliam and Ptan strains as the diagnostic antigen. CIA performance was tested on a panel of 112 control sera from confirmed cases of scrub typhus. The detection sensitivities of CIA against anti-O. tsutsugamushi total antibodies, IgM, and IgG were 98.2%, 81.2%, and 94.6%, respectively, while that of IFA (using the lysate of the O. tsutsugamushi Gilliam-infected chicken yolk sac as the antigen) against IgG was 85.7%. One hundred five serum samples from healthy individuals and patients with other febrile diseases were tested with CIA as negative controls. Specificities of CIA against anti-O. tsutsugamushi total antibodies, IgM, and IgG were 98.1%, 100%, and 98.9%, respectively, while the specificity of IFA against IgG was 98.9%. These results indicated that CIA was a good assay and could substitute for conventional immunofluorescence assays for diagnosis of scrub typhus.

PMID: 17360883 [PubMed - indexed for MEDLINE]

324. Kansenshogaku Zasshi. 2009 May;83(3):256-60.

[Case of imported scrub typhus contracted in Myanmar].

[Article in Japanese]

Matsumura Y(1), Shimizu T.

Author information:
(1)Department of Infectious Diseases, Kyoto City Hospital.

Scrub typhus is widespread in rural south and southeastern Asia and the western Pacific. The scrub typhus incidence is the highest among vector-borne diseases in Japan, but imported cases are extremely rare. A 49-year-old man admitted for persistent fever, headache, and rash after returning from Myanmar had been exposed to mosquito and tick bites while doing a 12-day forest inventory in Myanmar. On admission, he had a generalized maculopapular rash but no apparent eschars characteristic of scrub typhus. Blood examination and abdominal ultrasonography showed elevated liver enzymes, thrombocytopenia, and hepatosplenomegaly. Repeated blood smears and blood cultures were negative for malaria infection and bacteremia. Dengue fever was denied by both PCR and serology. The patient deteriorated on the ninth day and suffered complications of rhabdomyolysis, pneumonia, and enteritis. Based on a tentative diagnosis of typhoid fever or rickettiosis, we administratered ceftriaxone and minocycline, which dramatically reduced clinical signs and symptoms. After discharge on day 19, immunofluorescence assay showed significantly increased antibodies for Orienta tsutsugamushi serotype Gilliam, first discovered in Myanmar. All serological results were negative for other rickettioses, leptospirosis and Q fever. Given the many travelers from Japan visiting endemic scrub typhus areas, we must recognize cases of imported scrub typhus among those travelers with fever and rash returning from endemic areas.

PMID: 19522310 [PubMed - indexed for MEDLINE]

325. Southeast Asian J Trop Med Public Health. 1996 Sep;27(3):580-3.

Diagnosis of scrub typhus in Malaysian aborigines using nested polymerase chain reaction.

Tay ST(1), Nazma S, Rohani MY.

Author information:
(1)Bacteriology Division, Institute for Medical Research, Jalan Pahang, Kuala Lumpur, Malaysia.

A rapid diagnostic system for scrub typhus using nested polymerase chain reaction (PCR) was applied to clinical samples from Malaysian Aborigines. Whole blood from twenty-four patients suspected of scrub typhus infection were tested using nested polymerase chain reaction and sera were evaluated by the indirect immunoperoxidase test. Antibody responses towards Rickettsia tsutsugamushi were observed in seventeen patients with the majority having high titers of IgG antibodies. Seven patients were seronegative. The nested PCR amplified R. tsutsugamushi DNA from six patients, of which two were negative serologically and four had high titers of IgG antibodies. Second samples collected seven days after treatment were negative by PCR testing. Nested PCR is highly sensitive and specific and may be used to provide rapid confirmation of scrub typhus cases in endemic region.

PMID: 9185274 [PubMed - indexed for MEDLINE]

326. Am J Trop Med Hyg. 1995 Jul;53(1):43-6.

Effectiveness of a dot-blot immunoassay of anti-Rickettsia tsutsugamushi antibodies for serologic analysis of scrub typhus.

Weddle JR(1), Chan TC, Thompson K, Paxton H, Kelly DJ, Dasch G, Strickman D.

Author information:
(1)Viral and Rickettsial Diseases Program, Naval Medical Research Institute, Bethesda, Maryland, USA.

We compared a commercially available dot-blot immunoassay system with the indirect immunofluorescence assay (IFA) in tests of known negative and known positive sera from scrub typhus cases. Using a panel of 100 sera from patients with various rickettsial and nonrickettsial infections, we observed that the IFA was 99% specific and the dipstick assay was 98% specific. In tests of 91 sera (30 negative and 61 positive for scrub typhus antibodies) from a study of febrile patients in Malaysia, using the standard of an IFA titer < 1:64 as negative, an IFA titer > 1:128 as positive, and an IFA titer = 1:64 as either positive or negative (supported by clinical records), dipsticks were 83% specific and 90% sensitive. The quantitative correlation of the dipsticks to IFA titers was confirmed by significant differences in geometric means of inverse IFA titers corresponding to the number of positive dipstick spots (no dots = 8.5, one dot = 43.3, two dots = 206.7, and three dots = 676.9). The assay would enable physicians and public health workers who deal with patients to quickly diagnose and appropriately treat most cases of the disease, especially in areas of high prevalence where the proportion of false-positive results to true-positive results would be low.

PMID: 7625532 [PubMed - indexed for MEDLINE]

327. Ann N Y Acad Sci. 2006 Oct;1078:188-96.

The foci of scrub typhus and strategies of prevention in the Spring in Pingtan Island, Fujian Province.

Hengbin G(1), Min C, Kaihua T, Jiaqi T.

Author information:
(1)Huadong Research Institute of Medicine and Biotechnics, Nanjing (21002), China. hbguo2003@yahoo.com.cn

This study investigates the foci of tsutsugamushi disease on Pingtan Island, China, with emphasis on cases of illness that occur in the spring. The investigation of the physical and medical geography in the endemic area and detection of 141 sera samples in the population of Pingtan Island is described. The serum samples were taken from 102 soldiers and 39 residents and were tested by the indirect immunofluorescence assay. An island resident's history of illness inquiry was also conducted, and the results are discussed. We were able to determine the most prevalent types of rats and mites from the rat bodies. We

isolated the DNA from the spleen of 40 rats and from five groups of mites by polymerase chain reaction. Orientia tsutsugamushi was isolated by perctoneally injecting KM mice with the patient's untreated blood along with ground rat viscera (R. losea) and ground mites (L. deliense), respectively. The isolates were typed at the molecular level. This study will then present direct prevention strategies that emphasize personal hygiene, methods of personal protection, keeping living environments clean, and it will provide strategies to eliminate rats and mites. As a result of adopting these strategies, no case of scrub typhus occurred in this region in approximately 2003-2004.

DOI: 10.1196/annals.1374.130
PMID: 17114707 [PubMed - indexed for MEDLINE]

328. Ann Acad Med Singapore. 1981 Jan;10(1):107-11.

Scrub typhus 1980.

Twartz JC.

Scrub typhus is a widespread and at times serious infection in Asia. If results from central Malaysia can be applied, it appears to be economically important. Diagnosis is often difficult and treatment prone to fail if short courses of antibiotics are used. Prophylaxis is the key area of research with the development of a vaccine being the ultimate goal.

PMID: 6792975 [PubMed - indexed for MEDLINE]

329. Jpn J Infect Dis. 2008 Mar;61(2):148-50.

Comparative research on epidemiological aspects of tsutsugamushi disease (scrub typhus) between Korea and Japan.

Bang HA(1), Lee MJ, Lee WC.

Author information:
(1)Faculty of Public Health, The Korean Public Health Association, Seoul, Korea.

In order to compare the epidemiological aspects of tsutsugamushi disease (scrub typhus) between Korea and Japan, we analyzed the current state of tsutsugamushi disease outbreaks and related risk factors. The average prevalence rate of tsutsugamushi disease from 2001 to 2005 in Korea was higher than that in Japan (7.2 and 0.3 per 100,000 population, respectively) ($P<0.01$). The seasonal distribution in Korea showed that the incidence of tsutsugamushi disease in autumn (94.5% of total cases) was higher than in Japan (38.2%) ($P<0.01$), while the incidences of outbreaks in other seasons were much higher in Japan ($P<0.01$). In Korea, more females (64.8%) were infected than males (35.2%) ($P<0.01$), while there was no significant difference in Japan. The remarkable difference between the gender distribution in Korea and Japan is believed to reflect cultural differences between the two countries in terms of work, dress and ornamentation. In both countries, elderly people (over 60 years old), especially in rural areas, showed a very high prevalence rate (50%), which is possibly due to increased

outdoor activities and a decreased number of young people in those areas. These differences in tsutsugamushi disease risk factors reflect the different influences of vectors/hosts, climate, and geographical and cultural characteristics between the two countries.

PMID: 18362409 [PubMed - indexed for MEDLINE]

330. J Clin Microbiol. 2006 Mar;44(3):1169-71.

Usefulness of eschar PCR for diagnosis of scrub typhus.

Lee SH(1), Kim DM, Cho YS, Yoon SH, Shim SK.

Author information:
(1)Department of Pediatrics, Seonam University College of Medicine, Gwangju, South Korea.

We report here on the case of a child who was infected with scrub typhus, and we made the diagnosis according to the serology and by performing PCRs on the child's eschar. The patient was treated with azithromycin, and he did not experience any complications. Performing nested PCR on the eschar might be both a rapid diagnostic test for scrub typhus in the early acute stage and a differential test as to whether or not a scab is a scrub typhus eschar.

DOI: 10.1128/JCM.44.3.1169-1171.2006
PMCID: PMC1393074
PMID: 16517922 [PubMed - indexed for MEDLINE]

331. Southeast Asian J Trop Med Public Health. 2009 Jul;40(4):789-94.

Scrub typhus in children in a teaching hospital in eastern Taiwan, 2000-2005.

Huang CT(1), Chi H, Lee HC, Chiu NC, Huang FY.

Author information:
(1)Department of Pediatrics, Mackay Memorial Hospital, Taipei, Taiwan.

Scrub typhus is an endemic disease in eastern Taiwan. We conducted a study of scrub typhus cases among hospitalized pediatric patients. Twenty-eight pediatric cases were confirmed to be scrub typhus (either by immunofluorescence assay or polymerase chain reaction) from 2000 to 2005. The medical records of these patients were reviewed for demographics and clinical manifestations. The majority of the children (60.7%) diagnosed with scrub typhus were male. Approximately half the patients were < 5 years old and the mean age (SD) was 6.1 (3.66) years. Patients were more likely to live in rural rather than urban areas. The greatest number of cases was seen in the spring and summer. The primary clinical symptoms included fever (100%), cough (50%), eschar (50%), rash (35.7%), poor appetite (42.9%), lymphadenopathy (42.9%), headache (39.3%), and hepatomegaly (35.7%). AC-reactive protein (CRP) was elevated in 100%, an aspartate aminotransferase (AST) was elevated in 100%, an alanine aminotransferase (ALT) level was elevated in 91.3%, hypoalbuminemia was found in 88.9% and proteinuria in 50%. The mean

(SD) duration of antibiotics was 11.0 (2.68) days and the mean (SD) duration for fever resolution after treatment was 2.8 (2.51) days. Meningoencephalitis was noted in 6 patients. Our case series had no mortalities. These results suggest that a diagnosis of scrub typhus should be suspected in children with fever and laboratory evidence of liver dysfunction who live in rural eastern Taiwan.

PMID: 19842416 [PubMed - indexed for MEDLINE]

332. Mil Med. 1993 Apr;158(4):269-73.

Scrub typhus in Korea: importance of early clinical diagnosis in this newly recognized endemic area.

Yi KS(1), Chong Y, Covington SC, Donahue BJ, Rothen RL, Rodriguez J, Arthur JD.

Author information:
(1)Yi's Clinic of Internal Medicine, Chinhae, Korea.

Scrub typhus became a well recognized infectious disease threat to military operations in the Pacific Theater during World War II. Early diagnosis and treatment with tetracycline or chloramphenicol dramatically reduces the mortality and morbidity of this disease. Korea is a newly recognized scrub typhus endemic country. We report our experience with 189 scrub typhus patients seen at a civilian outpatient clinic in Chinhae, Republic of Korea, from 1985 through 1990, and verify the accuracy of clinical diagnosis by serologic tests.

PMID: 8479637 [PubMed - indexed for MEDLINE]

333. J Trop Med Hyg. 1991 Apr;94(2):97-101.

Scrub typhus in Hong Kong.

Heap BJ(1).

Author information:
(1)Department of Medicine, British Military Hospital, Hong Kong.

A retrospective analysis of cases of scrub typhus in military personnel in Hong Kong during the years 1979-1989 is presented. Fifty-nine cases were identified, most occurring during the hot humid season between May and October. Few civilian cases of scrub typhus are notified to the Hong Kong Medical and Health Department and possible reasons for the disparity in case numbers between military and civilian personnel are discussed. Reports from other areas of South-east Asia have documented a previously unrecognized high incidence of scrub typhus in the indigenous population. It is suggested that a prospective study utilizing specific serological diagnostic techniques would be of value in assessing the importance of scrub typhus as a cause of 'fever of undetermined origin' in the civilian population of Hong Kong.

PMID: 2023295 [PubMed - indexed for MEDLINE]

334. Pathology. 2008 Apr;40(3):268-71. doi: 10.1080/00313020801911488.

Pulmonary pathology in patients associated with scrub typhus.

Hsu YH(1), Chen HI.

Author information:
(1)Department of Pathology, Tzu Chi Hospital and University, Hualien, Taiwan.

AIMS: Scrub typhus is a zoonotic disease caused by Orientia tsutsugamushi. Severe cases resulting in mortality from this disease have rarely been reported. We present two scrub typhus cases (a man and a girl) who died of acute respiratory distress syndrome (ARDS).
METHODS: Autopsies were performed. Histopathological and immunohistochemical stains were employed using specific antibody for O. tsutsugamushi and inducible nitric oxide synthase (iNOS).
RESULTS: These subjects developed respiratory distress shortly after admission, and expired following respiratory failure. At autopsy, generalised lymphadenopathy was observed. The lung weight was about two-fold the normal value. Gross inspection revealed oedematous and haemorrhagic lungs. Microscopic examination revealed diffuse alveolar damage with hyaline membrane formation and interstitial pneumonitis with infiltration of inflammatory cells. Immunohistochemical stain showed O. tsutsugamushi antigen depositions in the endothelial cells. We also demonstrated iNOS in the alveolar macrophages and lung tissue debris in both cases.
CONCLUSION: Scrub typhus is usually a mild infectious disease. Our cases present the most dramatic example of sudden death due to ARDS in a short period of time. The clinical investigation and analysis suggest direct endothelial cell invasion of the organism and marked iNOS expression may be involved in the pathogenesis of ARDS associated with scrub typhus.

DOI: 10.1080/00313020801911488
PMID: 18428046 [PubMed - indexed for MEDLINE]

335. Ir J Med Sci. 2009 Sep;178(3):347-50. doi: 10.1007/s11845-008-0226-z. Epub 2008 Oct 16.

Scrub typhus as a possible aetiology of Guillain-Barré syndrome: two cases.

Lee MS(1), Lee JH, Lee HS, Chang H, Kim YS, Cho KH, Ahn SH, Song JH, Yoo M, Han JK, Park HY.

Author information:
(1)Department of Medicine, Wonkwang University Hospital, Wonkwang University School of Medicine, Iksan, Jeonbuk 570-180, South Korea.

Neurological complications of scrub typhus are reported to be rare. Peripheral nervous system involvement has been reported in only one case. We present two cases of Guillan-Barré syndrome (GBS) associated with scrub typhus. In both cases, the findings of an elevated indirect immunofluorescent antibody titer for

Orientia tsutsugamushi and nerve conduction study showing sensory-motor
polyneuropathy, have led us to believe that scrub typhus could be one of the
antecedent illnesses associated with GBS.

DOI: 10.1007/s11845-008-0226-z
PMID: 19009332 [PubMed - indexed for MEDLINE]

336. Klin Padiatr. 2012 Jul;224(4):270-1. Epub 2012 Apr 27.

Epistaxis, maculopapular rash and Fever in a german boy after a stay in Thailand
caused by Orientia tsutsugamushi.

Keller C, Fähndrich K, Müller W, Nüsslein TG, Fleischer B, Hegasy G.

DOI: 10.1055/s-0032-1301931
PMID: 22549469 [PubMed - indexed for MEDLINE]

337. Emerg Infect Dis. 2003 Dec;9(12):1638-41.

Scrub typhus reemergence in the Maldives.

Lewis MD(1), Yousuf AA, Lerdthusnee K, Razee A, Chandranoi K, Jones JW.

Author information:
(1)Armed Forces Research Institute of Medical Sciences, Bangkok, Thailand.
michael.lewis14@us.army.mil

In summer 2002, an outbreak of febrile illness began in the Maldives in the
Indian Ocean. Through April 2003, officials recorded 168 cases with 10 deaths.
The Armed Forces Research Institute of Medical Sciences in Bangkok confirmed
Orientia tsutsugamushi and conducted a joint investigation with the Ministry of
Health, Maldives. These cases of scrub typhus were the first in the Maldives
since World War II.

DOI: 10.3201/eid0912.030212
PMCID: PMC3034347
PMID: 14720413 [PubMed - indexed for MEDLINE]

338. Intern Med. 2008;47(22):1997-2000. Epub 2008 Nov 17.

Scrub typhus mimicking deep neck infection.

Tsai CC(1), Chiang DH, Duh RW.

Author information:
(1)Department of Medicine, Section of Infectious Disease, Buddhist Dalin Tzu Chi
General Hospital and Tzu Chi University, Chiayi, Taiwan. antibody_1@msn.com

Scrub typhus is not uncommon in Asia and it is life threatening without correct
treatment. The presentation of scrub typhus mimicking deep neck infection is
rarely encountered by clinical physicians and delays the determination of the

correct diagnosis. Here, we report a case of scrub typhus presenting symptoms like deep neck infection. The patient was admitted due to fever and a tender swelling on left side of his neck, which progressed to respiratory failure and acute renal failure under empirical use of ceftriaxone and metronidazole. After repetitive physical examinations, an eschar was found on his scalp. Finally he was successfully treated with tetracycline antibiotics.

PMID: 19015616 [PubMed - indexed for MEDLINE]

339. J Assoc Physicians India. 2007 Jul;55:512-4.

Acute reversible hearing loss in scrub typhus.

Mahajan SK(1), Bakshi D.

Author information:
(1)Deen Dayal Upadhayaya Zonal Hospital, Shimla, Himachal Pradesh, India.

Scrub typhus usually presents as pyrexia with or without multiple organ involvement. Acute hearing loss occurs in about one third of cases and is a useful clue toward the diagnosis. We present two cases of scrub typhus with acute reversible hearing loss from an endemic area. The diagnosis was confirmed by nested PCR.

PMID: 17907503 [PubMed - indexed for MEDLINE]

340. Curr Opin Infect Dis. 2003 Oct;16(5):429-36.

Scrub typhus and tropical rickettsioses.

Watt G(1), Parola P.

Author information:
(1)Department of Retrovirology, Armed Forces Research Institute of Medical Sciences, Bangkok, Thailand. wattgh@thai.amedd.mil

PURPOSE OF REVIEW: Recent developments in molecular taxonomic methods have led to a reclassification of rickettsial diseases. The agent responsible for scrub typhus (Orientia tsutsugamushi) has been removed from the genus Rickettsia and a bewildering array of new rickettsial pathogens have been described. An update of recent research findings is therefore particularly timely for the nonspecialist physician.
RECENT FINDINGS: An estimated one billion people are at risk for scrub typhus and an estimated one million cases occur annually. The disease appears to be re-emerging in Japan, with seasonal transmission. O. tsutsugamushi has evolved a variety of mechanisms to remain viable in its intracellular habitat. Slowing the release of intracellular calcium inhibits apoptosis of macrophages. Subsets of chemokine genes are induced in infected cells, some in response to transcription factor activator protein 1. Cardiac involvement is uncommon and clinical complications are predominantly pulmonary. Serious pneumonitis occurred in 22% of

Chinese patients. Dual infections with leptospirosis have been reported. Standardized diagnostic tests are being developed and attempts to improve treatment of women and children are being made. Of the numerous tick-borne rickettsioses identified in recent years, African tick-bite fever appears to be of particular importance to travellers. The newly described flea-borne spotted fever caused by Rickettsia felis may be global in distribution.
SUMMARY: Rash and fever in a returning traveler could be rickettsial and presumptive doxycycline treatment can be curative. Recent research findings raise more questions than answers and should stimulate much needed research.

DOI: 10.1097/01.qco.0000092814.64370.70
PMID: 14501995 [PubMed - indexed for MEDLINE]

341. Indian J Pediatr. 2010 Aug;77(8):918. doi: 10.1007/s12098-010-0157-3.

The eschar of scrub typhus.

So S, Sendil KD.

DOI: 10.1007/s12098-010-0157-3
PMID: 20721699 [PubMed - indexed for MEDLINE]

342. J Med Assoc Thai. 1990 Oct;73(10):585-91.

Scrub typhus in suburban Bangkok: first cases.

Eamsila C(1), Buranakitjaroen P, Tanskul P, Watcharapichat P.

Author information:
(1)Division of Research, Royal Thai Army Component, Armed Forces Research Institute of Medical Sciences (AFRIMS), Bangkok.

Three patients, two of whom had eschars, were admitted with fulminant febrile disease suggestive of scrub typhus. Elevated IgG and IgM to Rickettsia tsutsugamushi were detected in all three by IFA. The cases lived in widely separated areas of western Bangkok where fruit trees are grown and claimed not to have left the areas of their homes during the estimated incubation periods. A field survey collected rodents, treeshrews, and mites fro around the homes of each case. R. tsutsugamushi was isolated from one rat and known vector species of mites were found. These are the first confirmed cases of scrub typhus transmission in Metropolitan Bangkok. It is suggested that typhus incidence around Bangkok may be substantial but has been unreported because of widespread self-medication with antibiotics and the difficulties of laboratory diagnosis.

PMID: 2126275 [PubMed - indexed for MEDLINE]

343. Kansenshogaku Zasshi. 2001 May;75(5):353-8.

[Tsutsugamushi disease (scrub typhus) in Japan: epidemiological aspects].

[Article in Japanese]

Ogawa M(1), Hagiwara T, Kishimoto T, Shiga S, Yoshida Y, Furuya Y, Kaiho I, Ito T, Nemoto H, Yamamoto N, Masukawa K.

Author information:
(1)Kanagawa Prefecture Public Health Laboratory.

Epidemiological aspects of tsutsugamushi disease (scrub typhus) in Japan in 1998 were analyzed using questionnaires. Four hundred and sixteen scrub typhus cases were reported in 24 prefectures in 1998. The annual number of the patients in 1998 was similar to those in the preceding three years. There was no sex difference. The patients at the age of 51 or greater accounted for 72% of the total cases. Patients engaged in farming and forestry accounted for 32% and 14% cases, respectively. Fifty-six, 21 and 19% were reported in Kyusyu, Kanto and Tohoku-Hokuriku districts, respectively, 96% of the total cases being reported in these 3 districts. Most cases were reported from April through June with some from October through December in Tohoku-Hokuriku districts, while most cases were reported from October through December in other districts, including Kyusyu and Kanto districts. Thus, there was a difference in epidemic seasons among the districts. Serotypes of scrub typhus rickettsia were analyzed by serum antibody titers in the Kyusyu district. The novel Kawasaki and Kuroki types were major strains; however, no geographical difference was seen within the Kyusyu district. Interestingly, 24 cases were diagnosed only by the new serotypes not by the classical serotypes (Kato, Karp and Gilliam) in serological tests. This result suggests that further investigations are required to determine the prevalent serotypes in each district and to improve the serological tests. This was the first comprehensive report of epidemiology of scrub typus in entire Japan. Information obtained in the present study provides deep insight into prediction, diagnosis, treatment and prevention of scrub typhus in Japan.

PMID: 11424483 [PubMed - indexed for MEDLINE]

344. J Theor Biol. 2010 Sep 7;266(1):154-61. doi: 10.1016/j.jtbi.2010.06.015. Epub 2010 Jun 23.

A model for the transmission dynamics of Orientia tsutsugamushi among its natural reservoirs.

Kim BN(1), Gordillo LF, Kim Y.

Author information:
(1)Department of Mathematics, Kyungpook National University, Daegu 702-701, Republic of Korea.

The bacteria Orientia tsutsugamushi is the causative agent of scrub typhus, a prevalent disease in Asian countries that can affect humans and which shows an alarming increase of cases during the last years, especially in rural areas. Unfortunately, there is no vaccine for scrub typhus, and antibiotic treatments successfully used in the past appear to be inefficient to treat some strains of O. tsutsugamushi. We introduce a mathematical model that approximates the dynamics of the bacteria among its natural reservoirs. After computing the basic

reproductive number from the proposed model, we explore its sensitivity to the parameter values that may be affected by application of control measures. This theoretical model may be of interest to pest managers as well as health authorities interested in gaining insight into the public management of the disease, through a better understanding of its qualitative dynamics.

Copyright 2010 Elsevier Ltd. All rights reserved.

DOI: 10.1016/j.jtbi.2010.06.015
PMID: 20600142 [PubMed - indexed for MEDLINE]

345. Am J Trop Med Hyg. 1974 Jul;23(4):679-84.

Scrub typhus in eastern Taiwan, 1970.

Gale JL, Irving GS, Wang HC, Lien JC, Chen WF, Cross JH.

PMID: 4211123 [PubMed - indexed for MEDLINE]

346. Clin Infect Dis. 1994 Apr;18(4):624-6.

Life-threatening scrub typhus in a traveler returning from Thailand.

Watt G(1), Strickman D.

Author information:
(1)Department of Medicine, Armed Forces Research Institute of Medical Science, Bangkok, Thailand.

Scrub typhus is not one of the more commonly encountered diseases in travelers returning from Asia, but it deserves more consideration in view of its severity and the availability of specific chemotherapy and chemoprophylaxis. We describe a case of scrub typhus that was associated with coma and multiorgan failure in a traveler returning to the United States from Thailand. The diagnosis was made only retrospectively despite a travel history and clinical signs that suggested infection with Rickettsia tsutsugamushi. No specific therapy was given, and marked neurological impairment persisted 6 months after the beginning of the illness. An increased awareness of scrub typhus is a prerequisite for recommending prophylaxis and instituting prompt therapy.

PMID: 8038320 [PubMed - indexed for MEDLINE]

347. Infection. 2000 May-Jun;28(3):178-9.

Scrub typhus-associated hemophagocytic syndrome.

Chen YC(1), Chao TY, Chin JC.

Author information:

(1)Div of Hematology/Oncology, Tri-Service General Hospital, Taipei, Taiwan, Republic of China. jo607@ms6.hinet.net

A patient was admitted to our hospital with fever of unknown origin, lymphadenopathy and moderate anemia. The diagnosis of scrub typhus (tsutsugamushi disease) was established on specific serologic demonstration of antibodies to the cross-reacting proteins OX-K antigen and reaffirmed by successful treatment with doxycycline. The diagnosis of hemophagocytic syndrome (HPS) was made on the cytologic findings of many histiocytes containing phagocytosed blood cells in the marrow aspirate. The hemophagocytosis phenomenon disappeared after the scrub typhus was successfully treated, thus suggesting the relationship between scrub typhus and hemophagocytosis. In a patient with rickettsial diseases including scrub typhus, associated with HPS, it is important to understand the relationship between the two disorders since the prognosis for HPS, if untreated, is very poor.

PMID: 10879646 [PubMed - indexed for MEDLINE]

348. J Gastroenterol Hepatol. 1995 Jul-Aug;10(4):484-7.

Granulomatous hepatitis associated with scrub typhus.

Chien RN(1), Liu NJ, Lin PY, Liaw YF.

Author information:
(1)Liver Unit, Chang Gung Memorial Hospital, Chang Gung Medical College, Taipei, Taiwan.

A 56 year old patient with scrub typhus infection having unusual presentation of hepatic injury resembling acute hepatitis is described. The clinical features of fever, headache, eschar, lymphadenopathy, lymphocytosis and high Rickettsia tsutsugamushi immunofluorescence titres confirmed the diagnosis of scrub typhus. Acute hepatitis was proven by hepatic biochemical tests and liver biopsy. The patient had a complete recovery soon after antibiotic treatment. The presentation of this case suggests that scrub typhus infection should be included in the list of differential diagnosis of acute hepatitis or granulomatous hepatitis, at least in the Asian Pacific region where scrub typhus still prevails.

PMID: 8527719 [PubMed - indexed for MEDLINE]

349. Emerg Infect Dis. 2003 Apr;9(4):480-2.

Scrub typhus in the Torres Strait islands of north Queensland, Australia.

Faa AG(1), McBride WJ, Garstone G, Thompson RE, Holt P.

Author information:
(1)Thursday Island Hospital, Queensland, Australia.

Scrub typhus, caused by Orientia tsutsugamushi, occurs throughout Southeast Asia.

We descript ten cases that occurred in the Torres Strait islands of northern Australia during 2000 and 2001. Preceding heavy rain may have contributed to the outbreak. The successful use of azithromycin in two pediatric patients is also reported.

DOI: 10.3201/eid0904.020509
PMCID: PMC2957983
PMID: 12702230 [PubMed - indexed for MEDLINE]

350. Southeast Asian J Trop Med Public Health. 2002 Jun;33(2):312-3.

Acute scrub typhus in Northern Thailand: EKG changes.

Watt G(1), Kantipong P, Jirajarus K.

Author information:
(1)Department of Retrovirology, Armed Forces Research Institute of Medical Sciences, Bangkok, Thailand.

The electrocardiographic (EKG) manifestations of scrub typhus were prospectively evaluated in 29 adult patients who acquired Orientia tsutsugamushi infection in Chiang Rai, Northern Thailand. EKGs were normal in 22 of the 29 patients (76%); minor non-specific changes were found in the other 7 patients; ie ST segment/T wave changes (10%), U waves (7%), and premature ventricular contractions (4%). These results suggest that EKG changes in scrub typhus acquired in areas of diminished antibiotic susceptibility are similar to those observed in O. tsutsugamushi infection acquired elsewhere.

PMID: 12236430 [PubMed - indexed for MEDLINE]

351. Southeast Asian J Trop Med Public Health. 1977 Jun;8(2):232-5.

Presence of antibodies to scrub typhus and murine typhus in dogs from Selangor, Peninsular, Malaysia.

Huxsoll DL, Shirai A, Robinson DM, Yap LF, Lim BL.

Dog sera, collected from different communities throughout Selangor, Peninsular Malaysia, were investigated for the presence of antibodies to R. tsutsugamushi and R. typhi. Scrub typhus antibodies were present in animals from the rural areas only, whereas murine typhus antibodies were observed in equal numbers of dogs from both rural and metropolitan areas. Greater percentage of dogs from suburban areas had demonstrable antibody titers to murine typhus than from the urban area.

PMID: 411180 [PubMed - indexed for MEDLINE]

352. Pediatrics. 1992 May;89(5 Pt 1):965-8.

Neonatal scrub typhus: a case report.

Wang CL(1), Yang KD, Cheng SN, Chu ML.

Author information:
(1)Department of Pediatrics, Tri-Service General Hospital, National Defense Medical Center, Taipei, Taiwan, Republic of China.

PMID: 1579412 [PubMed - indexed for MEDLINE]

353. Biomedica. 2013 Sep;33 Suppl 1:161-78.

[Tick-borne rickettsioses in the Americas: clinical and epidemiological advances, and diagnostic challenges].

[Article in Spanish]

Hidalgo M(1), Faccini-Martínez ÁA(1), Valbuena G(2).

Author information:
(1)Grupo de Enfermedades Infecciosas, Departamento de Microbiología, Facultad de Ciencias, Pontificia Universidad Javeriana. (2)Pathology Department, University of Texas Medical Branch, Pontificia Universidad Javeriana.

Rickettsioses are a group of zoonotic diseases caused by strict intracellular bacteria of the genus Rickettsia and Orientia which belong to the Rickettsiaceae family. Their ecology is influenced by environmental factors and the presence of specific vectors that determine the establishment and epidemiology in different world regions. In America, during the 20 th century, only three of these diseases were recognized: Rocky Mountain spotted fever, epidemic typhus and endemic typhus. However, since 2000, more than 10 different species that had previously been unknown in this continent have been described, both in arthropods and in clinical cases, fact that classifies them as emerging and re-emerging diseases. Given the clinical manifestations of the diseases caused by rickettsias, being the majority unspecific and, therefore, shared with other infectious diseases, especially viral and bacterial, they have been framed within the differential diagnoses of acute febrile syndrome in urban and tropical areas. Nowadays, there are direct and indirect diagnostic methods, which are useful in the definition of the infectious agent, in this case, the cause of rickettsioses.

PMID: 24652260 [PubMed - indexed for MEDLINE]

354. J Clin Microbiol. 2014 Mar;52(3):832-8. doi: 10.1128/JCM.02786-13. Epub 2013 Dec 26.

Loop-mediated isothermal amplification for Rickettsia typhi (the causal agent of murine typhus): problems with diagnosis at the limit of detection.

Dittrich S(1), Castonguay-Vanier J, Moore CE, Thongyoo N, Newton PN, Paris DH.

Author information:
(1)Lao-Oxford-Mahosot Hospital-Wellcome Trust Research Unit (LOMWRU), Microbiology Laboratory, Mahosot Hospital, Vientiane, Lao People's Democratic Republic.

Murine typhus is a flea-borne disease of worldwide distribution caused by Rickettsia typhi. Although treatment with tetracycline antibiotics is effective, treatment is often misguided or delayed due to diagnostic difficulties. As the gold standard immunofluorescence assay is imperfect, we aimed to develop and evaluate a loop-mediated isothermal amplification (LAMP) assay. LAMP assays have the potential to fulfill the WHO ASSURED criteria (affordable, sensitive, specific, user friendly, robust and rapid, equipment free, deliverable to those who need them) for diagnostic methodologies, as they can detect pathogen-derived nucleic acid with low technical expenditure. The LAMP assay was developed using samples of bacterial isolates (n=41), buffy coat specimens from R. typhi PCR-positive Lao patients (n=42), and diverse negative controls (n=47). The method was then evaluated prospectively using consecutive patients with suspected scrub typhus or murine typhus (n=266). The limit of detection was 40 DNA copies/LAMP reaction, with an analytical sensitivity of <10 DNA copies/reaction based on isolate dilutions. Despite these low cutoffs, the clinical sensitivity was disappointing, with 48% (95% confidence interval [95% CI], 32.5 to 62.7%) (specificity, 100% [95% CI, 100 to 100%]) in the developmental phase and 33% (95% CI, 9.2 to 56.8%) (specificity, 98.5% [95% CI, 97.0% to 100%]) in the prospective study. This low diagnostic accuracy was attributed to low patient R. typhi bacterial loads (median, 210 DNA copies/ml blood; interquartile range, 130 to 500). PCR-positive but LAMP-negative samples demonstrated significantly lower bacterial loads than LAMP-positive samples. Our findings highlight the diagnostic challenges for diseases with low pathogen burdens and emphasize the need to integrate pathogen biology with improved template production for assay development strategies.

DOI: 10.1128/JCM.02786-13
PMCID: PMC3957756
PMID: 24371248 [PubMed - indexed for MEDLINE]

355. Zhonghua Yi Xue Za Zhi (Taipei). 1989 Oct;44(4):274-8.

[Scrub typhus--one case report].

[Article in Chinese]

Lee ML, Lin MT.

Scrub typhus (tsutsugamushi disease) is an acute infectious disease caused by Rickettsia tsutsugamushi transmitted through the bite of larvae of certain trombiculid mites. Geographical distribution in Asian-Pacific region is much of the roughly triangular area bounded by Japan, Pakistan and Australia. It is an endemic illness in the Pescadores Islands, but has scarcely been reported in central Taiwan. An eleven-year-old boy was admitted to Changhua Christian Hospital with the chief complaints of fever, lethargy and skin rash for seven days. On physical examination, he was found to have painless eschar, conjunctivitis, meningoencephalitis, pneumonitis, ascites, jaundice, hepatomegaly, liver function impairment and thrombocytopenia. His Proteus OX-K

agglutinin titer increased from 1:160 in the acute sera to 1:640 in the convalescent sera. A greater than four-fold rise (greater than 1:640) in antibody titers to Karp, Gilliam, Kato strains of R. tsutsugamushi between acute and convalescent sera were demonstrated by immunofluorescent antibody. The patient was treated with minocycline and chloramphenicol and was completely recovered.

PMID: 2634464 [PubMed - indexed for MEDLINE]

356. Commun Dis Intell Q Rep. 2004;28(2):267-9.

Scrub typhus in the Northern Territory: exceeding the boundaries of Litchfield National Park.

Ralph A(1), Raines M, Whelan P, Currie BJ.

Author information:
(1)Infectious Diseases Registrar, Royal Darwin Hospital, Darwin, Northern Territory.

Scrub typhus is recognised as an important differential diagnosis of fever, rash and sepsis in patients with a history of travel to Litchfield National Park in the Top End of the Northern Territory. All confirmed scrub typhus cases to date from the Northern Territory have visited the Park, but the presence of similar rainforest pockets elsewhere in the Top End suggested further infectious locations might be identified with increased tourism. We report a case of serologically confirmed Orientia tsutsugamushi infection in a man who had not been within Litchfield Park, but had visited another discrete Top End rainforest area.

PMID: 15460968 [PubMed - indexed for MEDLINE]

357. Trans R Soc Trop Med Hyg. 2003 Sep-Oct;97(5):570-2.

Case reports: scrub typhus during pregnancy in India.

Mathai E(1), Rolain JM, Verghese L, Mathai M, Jasper P, Verghese G, Raoult D.

Author information:
(1)Department of Clinical Microbiology, Christian Medical College and Hospital, Vellore, Tamil Nadu, India.

Scrub typhus, caused by Orientia tsutsugamushi, is a rural zoonosis endemic in the Asian Pacific region. Doxycycline and chloramphenicol, the recommended drugs for treating this infection, may not be safe during pregnancy. We report on 5 patients with scrub typhus during pregnancy who were seen in India between October 2001 and February 2002. Four of the 5 women were treated initially with ciprofloxacin. Three women had stillbirths, 1 an abortion and 1 a low birthweight baby, which suggests that ciprofloxacin should not be used for treating pregnant women and that scrub typhus leads to severe adverse effects during pregnancy. Randomized controlled trials are urgently needed to ascertain the optimal drug

choice, given that currently recommended drugs are contraindicated in pregnant women.

PMID: 15307429 [PubMed - indexed for MEDLINE]

358. Trans R Soc Trop Med Hyg. 1982;76(1):85-8.

Population indices of chiggers (Leptotrombidium deliense) and incidence of scrub typhus in Chinese military personnel, Pescadores Islands of Taiwan, 1976-77.

Olson JG, Bourgeois AL, Fang RC.

Larval Leptotrombidium deliense were recovered from live-trapped Suncus murinus, Rattus rattus and R. norvegicus captured in the Pescadores Islands of Taiwan during 1976 and 1977. Weekly and monthly indices of chigger infestation expressed as a percentage of hosts infested and the number of chiggers per host for S. murinus and Rattus spp. were calculated. Close correlations were observed between monthly indices of L. deliense measured for S. murinus and Rattus spp. and the number of laboratory-confirmed cases of scrub typhus per month. Weekly indices from S. murinus correlated more closely with number of cases than did weekly indices from Rattus spp. The critical level of chigger infestation necessary for a single case of scrub typhus in a month was estimated at 0.69 chigger per S. murinus and 0.68 chigger per Rattus spp. The weekly index of chigger infestation necessary for a single case of scrub typhus during a week was calculated at 0.47 chiggers per shrew. Critical levels of chigger infestations expressed as percentages of hosts infested were not calculated.

PMID: 7080163 [PubMed - indexed for MEDLINE]

359. Boll Ist Sieroter Milan. 1989;68(1):1-4.

[Scrub typhus: an imported case seen in Milan].

[Article in Italian]

Bianchi GB(1), Croce G, Cellurale U.

Author information:
(1)Divisione Medicina Generale V Piano, Ospedale San Carlo Borromeo, Milano.

The Authors describe a scrub typhus case imported from Indo-China and observed in Milan, which is probably the first case reported in Italy. The patient, on holiday journey, exposed herself unwisely to the danger of catching the infection. The diagnosis was based on clinical grounds and Weil-Felix reaction. The problems of the diagnosis reliability and of the imported diseases are briefly discussed as well.

PMID: 2535243 [PubMed - indexed for MEDLINE]

360. Ann Intern Med. 1973 Jul;79(1):26-30.

Scrub typhus in South Vietnam. A study of 87 cases.

Berman SJ, Kundin WD.

PMID: 4198459 [PubMed - indexed for MEDLINE]

361. J Commun Dis. 2004 Dec;36(4):284-9.

Scrub Typhus--A short review.

Gupta V(1), Gautam V.

Author information:
(1)Department of Microbiology, Government Medical College and Hospital, Chandigarh.

PMID: 16506552 [PubMed - indexed for MEDLINE]

362. Trans R Soc Trop Med Hyg. 1976;70(5-6):444-8.

Scrub typhus: a common cause of illness in indigenous populations.

Brown GW, Robinson DM, Huxsoll DL, Ng TS, Lim KJ.

An explanation was sought for the disparity between the low reported incidence of scrub typhus and the high prevalence of antibody to Rickettsia tsutsugamushi in the rural population of Malaysia. A combination of isolation of the organism, titration of antibody by indirect immunofluorescence, and the Weil-Felix test was used to confirm infections. Scrub typhus was found to be very common, causing 23% of all febrile illnesses at one hospital. The infection was particularly prevalent in oil-palm workers, causing an estimated 400 cases annually in a population of 10,000 people living on one plantation. The clinical syndrome, whether mild or severe, was difficult to distinguish from that due to other infections. Eschars, rashes and adenopathy were uncommon. When used to examine early sera, the Weil-Felix test failed to confirm the diagnosis in most infections.20

PMID: 402722 [PubMed - indexed for MEDLINE]

363. Trans R Soc Trop Med Hyg. 2004 Jun;98(6):354-9.

Paediatric scrub typhus in Thailand: a study of 73 confirmed cases.

Silpapojakul K(1), Varachit B, Silpapojakul K.

Author information:
(1)Department of Paediatrics, Hat-yai Hospital, Hat-yai, Songkla, Thailand.

We studied 73 Thai children with scrub typhus (median age 9 years, range 3-14 years, male:female ratio 1.8:1). Most patients (86%) lived in rural areas. They presented with subacute fever (median, 9 d) with vomiting (35%), hepatomegaly (59%), splenomegaly (18%), and tachypnea (26%). Skin rash (7%), eschar (7%), and history of mite bite were rare. Blood leucocyte counts were usually normal but 19% of patients were thrombocytopenic. Twenty (22%) patients had pneumonia and six (8%) had neurological involvement. Defervescence occurred a median of 1 d and 3 d after initiation of doxycycline and chloramphenicol, respectively, and these responses were more rapid than in those who received other antibiotics or no treatment (P < 0.001). There was one death. Only 55% of the patients were initially diagnosed as having scrub typhus.

DOI: 10.1016/j.trstmh.2003.10.011
PMID: 15099991 [PubMed - indexed for MEDLINE]

364. Zhonghua Min Guo Wei Sheng Wu Ji Mian Yi Xue Za Zhi. 1993 Nov;26(4):166-70.

[Surveillance of scrub typhus in Taiwan].

[Article in Chinese]

Chen HL(1), Chen HY, Horng CB.

Author information:
(1)National Institute of Preventive Medicine, Department of Health, Executive Yuan Taipei, Taiwan, Republic of China.

Although scrub typhus, a rickettsial exanthematous febrile disease, has designated as a reportable communicable disease in Taiwan since 1955, only Pescadores island, Hualien and Taitung counties had more reported and confirmed cases. Suspected dengue fever and scrub typhus serum specimens sent from the Bureau of Health and health stations to this institute from January 1991 to December 1992 were tested for antibody to scrub typhus by indirect fluorescent antibody technique (IFA). The results showed that: 1) there were serologically positive cases in many counties except Hsinchu and Iilan, 2) the disease occurred mostly from July to October, 3) more males than females were infected and their age ranged from 21 to 60 years old with a peak of the twenties and 4) higher incidence rate in children under five years old was observed in Pescadores and Orchid islands. The above data call the attention of people and physicians to be aware of the fact that there were probable scrub typhus cases in every county in Taiwan.

PMID: 7988283 [PubMed - indexed for MEDLINE]

365. BMC Res Notes. 2012 Nov 30;5:662. doi: 10.1186/1756-0500-5-662.

Clinically helpful rickettsial disease diagnostic IgG titers in relation to duration of illness in an endemic setting in Sri Lanka.

Premaratna R(1), Weerasinghe S, Ranaweera A, Chandrasena TG, Bandara NW, Dasch GA, de Silva HJ.

Author information:
(1)Department of Medicine, Faculty of Medicine, University of Kelaniya, Colombo, Sri Lanka. ranjanp64@gmail.com

BACKGROUND: Although an initial IFA-IgG titer greater or equal to 1/64 or 1/128 is considered positive in presumptive diagnosis, in clinical practice in an endemic setting for rickettsioses in Sri Lanka, some patients with IFA-IgG titer of 1/128 for either spotted fever group (SFG) or scrub typhus (ST) did not respond to treatment.
FINDINGS: To determine a clinically helpful diagnostic algorithm, IFA-IgG results of serologically confirmed treatment responders were analyzed in relation to duration of illness at sampling. Of 146 suspected SFG, 3 responders of 25 patients had titers ≤1/128 with < 7 days of illness while all 9 with titers ≥1/256 responded (false negative with 1/256 cutoff was 12%, false positive was 0%). For illness > 7 days, the false negative and positive rates were 4.3% (3/59) and 11.3% (6/53). Of 115 suspected ST, false negative and positive rates with ≥1/256 cutoff at <7 days of illness were 14.2% (2/14) and 0% (0/8) respectively while > 7 days, false negative and positive rates were 2% (1/51) and 0% (0/42).
CONCLUSIONS: For clinical decision making, duration of illness at sampling is important in interpreting serology results in an endemic setting. If sample is obtained ≤7 day of illness, an IgG titer of ≤1/128 requires a follow up sample in the diagnosis and > 7 days of illness, a single ≥1/256 titer is diagnostic for all ST and 90% of SFG.

DOI: 10.1186/1756-0500-5-662
PMCID: PMC3536648
PMID: 23198969 [PubMed - indexed for MEDLINE]

366. Am J Epidemiol. 1977 Aug;106(2):172-5.

Rickettsia tsutsugamushi infection and scrub typhus incidence among Chinese military personnel in the Pescadores Islands.

Olson JG, Bourgeois AL.

Personnel assigned to infantry units of the army of the Republic of China (Taiwan) stationed in the Pescadores Islands experienced the highest incidence rate of scrub typhus (4%) and the highest rate of Rickettsia tsutsugamushi infection (12%) of the units studied during 1976. The ratio of scrub typhus to infection with R. tsutsugamushi was 30%. Non-immune personnel, when infected, were more likely to develop scrub typhus than were those who had previously been infected. There was a correlation between clinical illness and high antibody titers developed in response to the infection.

PMID: 888820 [PubMed - indexed for MEDLINE]

367. Jpn J Infect Dis. 2005 Aug;58(4):208-10.

Investigation of an outbreak of scrub typhus in the himalayan region of India.

Sharma A(1), Mahajan S, Gupta ML, Kanga A, Sharma V.

Author information:
(1)Department of Microbiology, Indira Gandhi Medical College and Hospital, Himachal Pradesh, India. dr_anu03@yahoo.co.in

In Indira Gandhi Medical College, Himachal Pradesh, India, during autumn of 2003 (September-November), more than 100 cases of fever of unknown origin (FUO) were reported with 15 ensuing deaths. In addition to all routine investigations and cultures, the Weil-Felix test was incorporated for the investigation of these cases. Antigen was procured from the Central Research Institute, Kasauli. Forty-six percent (45/96) of the cases demonstrated a > or =1:80 titer of agglutinins against OXK antigen. A team from the National Institute of Communicable Diseases, New Delhi, confirmed the antibodies for scrub typhus in some of the serum samples tested for leptospirosis, dengue fever, and rickettsial infections. Twelve blood samples positive for OXK antigen were sent to the Defense Research Development Establishment, Gwalior, for polymerase chain reaction studies, but none of the samples were positive, as all of the patients were already on broad-spectrum antibiotics and had reported to our hospital after 7-10 days of fever. At our institute, the Weil-Felix test has now been rountinely introduced for the investigation of cases of FUO, and the results until April 2004 (150 cases) revealed the presence of other rickettsial infections prevalent in the region. To evaluate the epidemiology and magnitude of the problem, further prospective studies are required.

PMID: 16116251 [PubMed - indexed for MEDLINE]

368. Am J Epidemiol. 1979 Feb;109(2):236-43.

Changing risk of scrub typhus in relation to socioeconomic development in the Pescadores islands of Taiwan.

Olson JG, Bourgeois AL.

Long term changes in risk of Rickettsia tsutsugamushi infection among civilian residents of the Pescadores Islands of Taiwan were associated with changing social conditions. Age specific incidence rates of scrub typhus in the Pescadores before 1940 were highest among children under 5 years of age. Rickettsiae can be demonstrated in vectors, wild animals continue to be infected and scrub typhus occurs in military personnel. A serologic survey for antibody to R. tsutsugamushi was conducted during 1975 and 1977 and failed to show evidence of previous infection among children. Two events appear to be associated with the decreased incidence of rickettsial infection in the young: increased urbanization and increased enrollment in schools. Both changes accompanied socioeconomic development which took place in the islands during the past 50 years. Prevalence of antibody to R. tsutsugamushi continues to be equal in the sexes and is undoubtedly due to similar occupational exposure of both sexes in fields and farms where vectors are numerous. The absence of apparent morbidity due to scrub typhus among the civilian populace was attributed to the mild nature of the

disease caused by Pescadores strains, misdiagnosis and a lack of obligatory reporting.

PMID: 425961 [PubMed - indexed for MEDLINE]

369. J Trop Med Hyg. 1964 Sep;67:215-9.

INVESTIGATION OF SCRUB TYPHUS IN THAILAND.

TRISHNANANDA M, VASUVAT C, HARINASUTA C.

PMID: 14200810 [PubMed - indexed for MEDLINE]

370. Bull Soc Pathol Exot Filiales. 1963 Jul-Aug;56:598-606.

[HISTORICAL OUTLINE OF RESEARCH ON THE ETIOLOGY AND EPIDEMIOLOGY OF SCRUB TYPHUS IN THE PLANTATION POPULATION OF SUMATRA].

[Article in French]

WOLFF JW.

PMID: 14108515 [PubMed - indexed for MEDLINE]

371. Am J Trop Med Hyg. 1964 Nov;13:833-8.

SCRUB TYPHUS IN THE PESCADORES ISLANDS: AN EPIDEMIOLOGIC AND CLINICAL STUDY.

COOPER WC, LIEN JC, HSU SH, CHEN WF.

PMID: 14222438 [PubMed - indexed for MEDLINE]

372. J Parasitol. 2016 Apr;102(2):193-8. doi: 10.1645/15-760. Epub 2015 Dec 10.

Geographical Distribution and Seasonal Indices of Chigger Mites on Small Mammals Collected on the East Coast of the Republic of Korea.

Park GM(1), Shin HS(1).

Author information:
(1)Department of Environmental Medical Biology and Institute for Clinical and Translational Research, Catholic Kwandong University College of Medicine, 24 Beomil-ro 579 Beon-gil, Gangneung-si, Gangwon-do, Korea.

The geographical distributions and relative population densities of scrub typhus vector mites collected from small mammals were determined for 5 locations on the

east coast of the Republic of Korea. Collection sites included Goseong, Gangneung, and Hoengseong in Gangwon province and Uljin and Yeongdeok in Gyeongbuk province. A total of 275 small mammals including members of Rodentia (rodents) and Soricomorpha (shrews, such as Crocidura lasiura) belonging to 4 genera and 4 species were captured in the field from 2012 to 2013. Apodemus agrarius was collected most frequently (220, 80%), followed by C. lasiura (25, 9.1%), Mus musculus (15, 5.5%), and Myodes regulus (15, 5.5%). A total of 23,436 larval chigger mites (Family Trombiculidae) belonging to 3 genera and 8 species (Leptotrombidium pallidum, Leptotrombidium scutellare, Leptotrombidium palpale, Leptotrombidium orientale, Leptotrombidium zetum, Neotrombicula tamiyai, Neotrombicula japonica, and Euschoengastica koreaensis) were collected from the small mammals. The predominant chigger species collected during the spring and fall seasons from A. agrarius were L. pallidum (57.6%), L. palpale (14.5%), and L. scutellare (7.9%). Leptotrombidium scutellare was collected only along the southeastern coast at Yeongdeok, Gyeongbuk province. The geographical distribution of scrub typhus vectors and reservoir hosts are important aspects of understanding the epidemiology of the disease as well as the potential impacts of climate change and health risks.

DOI: 10.1645/15-760
PMID: 26653927 [PubMed - in process]

373. Kansenshogaku Zasshi. 1963 Jul;37:130-5.

[STUDIES ON INAPPARENT SCRUB TYPHUS INFECTION. III. ON THE RELATION OF MILD FEBRILE DISEASES OF UNKNOWN ORIGIN TO INAPPARENT SCRUB TYPHUS INFECTION].

[Article in Japanese]

SHISHIDO A, HIKITA M, KITAOKA M.

PMID: 14044995 [PubMed - indexed for MEDLINE]

374. Emerg Infect Dis. 2006 Sep;12(9):1463-5.

Spring scrub typhus, People's Rrepublic of China.

Cao M, Guo H, Tang T, Wang C, Li X, Pan X, Tang J.

DOI: 10.3201/eid1209.060257
PMCID: PMC3294749
PMID: 17073108 [PubMed - indexed for MEDLINE]

375. J Infect Dis. 1973 Aug;128(2):223-6.

The silvered leaf-monkey of Malaysia, Presbytis cristatus: disease model for human scrub typhus.

Walker JS, Cadigan FC, Vosdingh RA, Chye CT.

PMID: 4198721 [PubMed - indexed for MEDLINE]

376. Indian J Pediatr. 2008 Sep;75(9):947-9. doi: 10.1007/s12098-008-0198-z. Epub 2008 Nov 15.

Pediatric scrub typhus in Indian Himalayas.

Mahajan SK(1), Rolain JM, Sankhyan N, Kaushal RK, Raoult D.

Author information:
(1)Department of Pediatrics, IG Medical College, Shimla, HP, India.
sanjay_mahajan64@rediffmail.com

To retrospectively confirm the suspected rickettsial disease (Scrub typhus) using a gold standard diagnostic test i.e. microimmunofluorescence in pediatric patients with acute febrile illness of unknown etiology. Two serological tests, Weil-Felix and Microimmunofluorescence were used to confirm infection. All five children had fever, vomiting and generalized lymphadenopathy, but none had eschar or rash. One was cured with doxycycline, remaining four patients treated with azithromycin and one died despite treatment. Scrub typhus is a cause of fever of unknown origin in Himalayan region of India and azithromycin is an effective alternative to doxycycline in treating this disease.

DOI: 10.1007/s12098-008-0198-z
PMID: 19011809 [PubMed - indexed for MEDLINE]

377. Emerg Infect Dis. 2004 Oct;10(10):1838-40.

Scrub typhus in the Republic of Palau, Micronesia.

Durand AM(1), Kuartei S, Togamae I, Sengebau M, Demma L, Nicholson W, O'Leary M.

Author information:
(1)Department of Health Services, Yap State, Colonia, Yap, Federated States of Micronesia 96943. durand@mail.fm

Erratum in
Emerg Infect Dis. 2004 Nov;10(11):2059.

In October 2001, an outbreak of febrile illness began in the southwest islands group of the Republic of Palau. Through October 2003, a total of 15 southwest islanders experienced fever >39.5 degrees C and abdominal distress, both lasting >7 days. Orientia tsutsugamushi, the agent of scrub typhus, was subsequently identified as the cause.

DOI: 10.3201/eid1010.040288
PMCID: PMC3323260
PMID: 15504273 [PubMed - indexed for MEDLINE]

378. Am J Trop Med Hyg. 1997 Jan;56(1):38-43.

Evaluation of an enzyme-linked immunosorbent assay in Thai scrub typhus patients.

Suwanabun N(1), Chouriyagune C, Eamsila C, Watcharapichat P, Dasch GA, Howard RS, Kelly DJ.

Author information:
(1)Department of Entomology, United States Army Medical Component, Armed Forces Research Institute of Medical Sciences, Bangkok, Thailand.

We report the development of an improved enzyme-linked immunosorbent assay (ELISA) to detect Orientia (formerly Rickettsia) tsutsugamushi antibody in human sera. Results were compared with a standard test, the indirect immunoperoxidase assay (IIP). Control serum samples were collected from 96 American soldiers and 198 Royal Thai Army soldiers with no recent history of clinical illness. Sera were examined from 79 febrile, Thai scrub typhus patients presenting at Chiang Rai (76) and Bangkraui Nontaburi (3) Provincial hospitals (cases confirmed by elevated IIP IgG levels > or = 1:1,600, IgM levels > or = 1:400, or presence of an eschar). The mean + 2 SD, used for the upper limit of normal reactions in the IgG ELISA, was 0.10 for U.S. soldiers and 0.42 for Thai soldiers. Using the 0.10 cutoff value, 29% of the asymptomatic Thai soldiers would be designated as antibody positive. Variability of IgG ELISA values was greater in the Thai soldier group than in American soldiers, possibly reflecting previous exposure to O. tsutsugamushi. In the Thai patients, there was a significant correlation between IIP titers and single serum dilution (1:100) ELISA values (IgG, r = 0.75, n = 104; P < 0.0005; IgM, r = 0.70, n = 75; P < 0.0005) and between IIP titers and ELISA titers (IgG, r = 0.87, n = 103; P < 0.0005; IgM, r = 0.76, n = 75; P < 0.0005). The single serum dilution ELISA was as effective as the titration in determining presence of specific antibodies. The O. tsutsugamushi ELISA is a rapid and objective test amenable to accurately testing the large numbers of sera often obtained in seroepidemiologic investigations.

PMID: 9063359 [PubMed - indexed for MEDLINE]

379. Zhonghua Liu Xing Bing Xue Za Zhi. 2007 Jun;28(6):547-9.

[Surveillance on Rickettsia in epidemic areas of scrub typhus in Xinyang areas of Henan].

[Article in Chinese]

Fu XP(1), Zhang JS, Shen XJ, Luan MC, Li ML, Zhang LJ.

Author information:
(1)Rickettsia Disease Department, National Institute of Communicable Disease Control and Prevention, Chinese Center for Disease Control and Prevention, Beijing 102206, China.

OBJECTIVE: To understand the epidemic status of Rickettsia in Xinyang areas of Henan province.
METHODS: Samples including liver, spleen, kidney from mouse and chigger mites from Xinyang areas and serum samples were detected by nested-polymerase chain

reaction (PCR) and indirect immunofluorescence assay (IFA).
RESULTS: In 62 viscus samples from mice organs, the positive rates were 16.13%, 8.06% and 6.45% for Orientia tsutsugamushi, R. typhii and Spotted fever group rickettsiae respectively. In blood clots samples from mice, the positive rates were 8.06%, 6.45% and 1.61 % for O. tsutsugamushi, R. typhii and Spotted fever group rickettsiae respectively. Three out of 26 mouse serum samples were positive for the predicted fluorexcent intensity O. tsutsugamushi.
CONCLUSION: Using nested-PCR and IFA methods, O. tsutsugamushi, R. typhii and Spotted fever group rickettsiae were detected in the captured mice living in Xinyang areas of Henan province. Results showed that there were intensive natural reserviors of Rickettsia in Henan province, suggesting that the risk of outbreak of Rickettsia in these areas was high.

PMID: 17939381 [PubMed - indexed for MEDLINE]

380. Am J Trop Med Hyg. 2016 Sep 19. pii: 16-0186. [Epub ahead of print]

Comparison of Preferred Bite Sites Between Mites and Ticks on Humans in Korea.

Jang MS(1), Kim CM(2), Kim DM(3), Yoon NR(1), Han MA(4), Kim HK(5), Oh WS(6), Yoon HJ(7), Wie SH(8), Hur JA(9).

Author information:
(1)Department of Internal Medicine, College of Medicine, Chosun University, Gwangju, South Korea. (2)Premedical Science, College of Medicine, Chosun University, Gwangju, South Korea. (3)Department of Internal Medicine, College of Medicine, Chosun University, Gwangju, South Korea. drongkim@chosun.ac.kr. (4)Department of Preventive Medicine, College of Medicine, Chosun University, Gwangju, South Korea. (5)Department of Pulmonary and Critical Care Medicine, Haeundae Paik Hospital, Inje University College of Medicine, Busan, Korea. (6)Department of Internal Medicine, Kangwon National University School of Medicine, Chuncheon, Korea. (7)Department of Infectious Diseases, Seoul Metropolitan Government Seobuk Hospital, Seoul, Korea. (8)Department of Internal Medicine, St. Vincent's Hospital, School of Medicine, The Catholic University of Korea, Suwon, South Korea. (9)Department of Internal Medicine, Yeungnam University, College of Medicine, Daegu, South Korea.

Identification of mite and tick bite sites provides important clinical information. The predominant mite species in Korea associated with scrub typhus are Leptotrombidium pallidum and Leptotrombidium scutellare The most abundant tick species is Haemaphysalis longicornis To date, there has been no comparative study on preferred bite sites between mites and ticks in humans. This study included a review of medical records and a field study. For mite bite sites, eschars were checked on 506 patients with scrub typhus, confirmed by indirect immunofluorescence assay or nested polymerase chain reaction on the 56-kDa type-specific antigen gene of Orientia tsutsugamushi Tick bite sites were identified and marked on a diagram for 91 patients who experienced tick bites within the previous year through a field epidemiological investigation. The mite and tick bite sites in Koreans were compared. The most frequently observed mite bite sites were the anterior chest, including the axillae (29.1%) and the abdominal region, including the inguinal area (26.1%). Tick bite sites were most frequent on the lower extremities (33.0%), followed by the abdominal region,

including the inguinal area (26.4%), and upper extremities (26.4%). The distribution was significantly different between mite and tick bite sites (P < 0.001). There was a statistically significant difference in the mite bite (P = 0.001), but not tick bite sites (P = 0.985), between men and women. This is the first report on the differences between tick and mite bite sites, and may help clinicians reach a rapid diagnosis of mite- or tick-borne infection.

© The American Society of Tropical Medicine and Hygiene.

DOI: 10.4269/ajtmh.16-0186
PMID: 27645781 [PubMed - as supplied by publisher]

381. Zhonghua Liu Xing Bing Xue Za Zhi. 2011 Mar;32(3):256-9.

[Sero-epidemiological investigation on Rickettsia typhi, Bartonella henselae and Orientia tsutsugamushi in farmers from rural areas of Tianjin, 2007 - 2009].

[Article in Chinese]

Zhang Y(1), Zhang ZL, Yin JY, Lv J, Yu HL, Liang CW, Wang SW, Zhao JB, Zhang LJ.

Author information:
(1)Tiangjin Center for Disease Control and Prevention, China.

OBJECTIVE: To study the sero-epidemiological status regarding Rickettsia (R.) typhi, Bartonella (B.) henselae and Orientia (O.) tsutsugamushi in farmers from rural areas of Tianjin.
METHODS: Field epidemiological surveys were performed in 8 districts (county) of Tianjin city from 2007 to 2009. 886 farmers were randomly recruited and their serum samples collected to detect the specific antibodies of R. typhi, B. henselae and O. tsutsugamushi by micro-indirect immunofluorescence (IFA).
RESULTS: The total antibody positive rates of R. typhi increased from 5.0% to 58.2% while B. henselae had an increase from 2.6% to 14.5% and O. tsutsugamushi increased from 1.8% to 39.8%. Geographic distribution showed that farmers living in the central and southeast areas were higher than that in other areas.
CONCLUSION: Infections of both R. typhi, B. henselae and O. tsutsugamushi in farmers from Tianjin areas were popular and the antibody positive rates of R. typhi, B. henselae and O. tsutsugamushi had an annual increase.

PMID: 21457660 [PubMed - indexed for MEDLINE]

382. Acta Med Biol (Niigata). 1967 Dec;15:61-7.

Physiogeographic distribution of scrub typhus in Thailand.

Elisberg BL, Sangkasuvana V, Campbell JM, Bozeman FM, Bodhidatta P.

PMID: 4968952 [PubMed - indexed for MEDLINE]

383. Rinsho Shinkeigaku. 2007 Jun;47(6):362-4.

[Case of tsutsugamushi disease (scrub typhus) presenting with fever and pain indistinguishable from trigeminal neuralgia].

[Article in Japanese]

Arai M(1), Nakamura A, Shichi D.

Author information:
(1)Department of Neurology, Seirei Mikatahara General Hospital.

A 64-year-old man visited our clinic with a 9-day history of headache and fever. He had frequent, severe, electric shock-like pain in his left eye, forehead, and scalp. The body temperature was 37.1 degrees. Cranial nerve functions were intact. Limb weakness and stiff neck were absent. There were injection of the conjunctiva, a red rash on the trunk, and an eschar in the axilla. Abnormal laboratory findings included AST 40 IU, ALT 44 IU, CRP 16.0 mg/dl, WBC 11,090/microl, and proteinuria. CT scan was unremarkable. The cerebrospinal fluid (CSF) showed 2 polymorphs/microl, 6 lymphocytes/microl, 65 mg/dl of glucose, and 42 mg/dl of protein. A diagnosis of scrub typhus was made. Treatment with minocycline brought about prompt disappearance of the fever and dramatic clinical improvement. Increased antibody titers confirmed the diagnosis. Although almost all patients present with high fever and severe headache, only a small number of patients have CSF pleocytosis. The present case illustrates that pain in scrub typhus is, on rare occasions, indistinguishable from trigeminal neuralgia. Neurologists should have a high index of suspicion in patients with fever and headache during the epidemic season and should be familiar with the systemic symptoms and signs.

PMID: 17633112 [PubMed - indexed for MEDLINE]

384. Intern Med. 2002 Aug;41(8):667-70.

Afebrile scrub typhus (Tsutsugamushi disease) with acute respiratory distress syndrome.

Ichimura K(1), Uchida Y, Arai K, Nakazawa K, Sasaki J, Kobayashi K, Iwai K, Kubo N.

Author information:
(1)Department of Internal Medicine, Saiseikai Utsunomiya Hospital.

A 74-year-old Japanese man with scrub typhus presented without the typical symptom of high fever and subsequently developed the complication of acute respiratory distress syndrome. It was suspected that exposure occurred at the river side of Kinugawa, Tochigi Prefecture, Japan. His body temperature was below 38.0 degrees C. After intensive supportive care and minocycline therapy, he dramatically recovered. With the increase in popularity of outdoor recreation, scrub typhus can be found in clinics all over Japan. Physicians should therefore be aware of the manifestations of the disease and the necessity of early treatment in suspected cases.

PMID: 12211540 [PubMed - indexed for MEDLINE]

385. Infection. 2001 Jan-Feb;29(1):54-6.

Scrub typhus pneumonitis acquired through the respiratory tract in a laboratory worker.

Oh M(1), Kim N, Huh M, Choi C, Lee E, Kim I, Choe K.

Author information:
(1)Dept. of Internal Medicine and Clinical Research Institute, Seoul National University Hospital, Korea.

We report a case of scrub typhus pneumonitis in a laboratory worker who apparently acquired it through the respiratory tract. The patient was suffering from fever, cough and dyspnea. He had both cervical and axillary lymphadenopathy, and hepatomegaly. A chest X-ray showed interstitial infiltrates. A diagnosis of scrub typhus was established upon isolation of Orientia tsutsugamushi. 12 days before the patient showed symptoms, he had purified O. tsutsugamushi proteins from infected cells using an ultrasonication method which could generate aerosols containing O. tsutsugamushi.

PMID: 11261762 [PubMed - indexed for MEDLINE]

386. Acta Neurol Taiwan. 2006 Dec;15(4):251-4.

Scrub typhus-associated acute disseminated encephalomyelitis.

Chen PH(1), Hung KH, Cheng SJ, Hsu KN.

Author information:
(1)Department of Neurology, Mackay Memorial Hospital, Taitung, Taiwan.
a766288.chen@msa.hinet.net

BACKGROUND: Acute disseminated encephalomyelitis (ADEM) is a monophasic demyelinating disease of the central nervous system, typically occurring after infections or vaccinations. To our knowledge, scrub typhus has not been described in association with ADEM.
CASE REPORT: A 77-year-old man was admitted with fever, convulsions and an altered level of consciousness. On neurological examination, the patient was stuporous and had nuchal rigidity and left hemiparesis. A generalized tonic-clonic seizure was observed. Serum and cerebrospinal fluid samples were positive for anti-Orientia tsutsugamushi antibody. Despite a 10-day course of parenteral minocycline, his clinical condition deteriorated. Serial cranial magnetic resonance images demonstrated progressively extensive areas of signal hyperintensity on conventional T2-weighted and fluid attenuated inversion recovery sequence images, mainly affecting the periventricular white matter. After administration of intravenous high-dose corticosteroids, the patient had limited improvement.

CONCLUSIONS: This is the first identifiable case of ADEM temporally associated with scrub typhus alone.

PMID: 17214088 [PubMed - indexed for MEDLINE]

387. Zhonghua Liu Xing Bing Xue Za Zhi. 1992 Aug;13(4):212-5.

[Report on first finding an epidemic of Scrub typhus in north rural areas].

[Article in Chinese]

Yu C(1).

Author information:
(1)Municipal Sanitary and Disease Prevention Centre, Tianjin.

In the autumn of 1989 and 1990, an epidemic of Scrub typhus occurred in north rural areas in Tianjin. The authors investigated the epidemic on clinical, epidemiological, serological and etiological features, 44 patients were diagnosed serologically or clinically as Scrub typhus and 42 of them (95.5%) were diagnosed serologically by IFA method. 10 sera specimens collected from the patients were determined distinctively by CF method, 8 of them were > or = 1:20 titre to Gilliam type antigen of tsutsugamushi. The try for isolating pathogens failed of success. The epidemic areas is situated in 39.45'-40.05' north latitudes and showed it was the new epidemic area in the north of China.

PMID: 1301265 [PubMed - indexed for MEDLINE]

388. Am J Trop Med Hyg. 2013 Feb;88(2):397-404. doi: 10.4269/ajtmh.12-0551. Epub 2013 Jan 16.

Acute and potentially life-threatening tropical diseases in western travelers--a GeoSentinel multicenter study, 1996-2011.

Jensenius M(1), Han PV, Schlagenhauf P, Schwartz E, Parola P, Castelli F, von Sonnenburg F, Loutan L, Leder K, Freedman DO; GeoSentinel Surveillance Network.

Collaborators: Jensenius M, Han PV, Schlagenhauf P, Schwartz E, Parola P, Castelli F, von Sonnenburg F, Loutan L, Leder K, Freedman DO, Simon F, Delmont J, Carosi G, Chappuis F, Caumes E, Pérignon A, Burchard GD, Torresi J, Brown G, Kain K, Boggild A, Keystone JS, Pandey P, Pradhan R, Murphy H, Coyle CM, Wittner M, de Vries P, Grobusch MC, Gadroen K, Rapp C, Aoun O, Kanagawa S, Kato Y, Mizunno Y, Gkrania-Klotsas E, McCarthy A, Weber R, Steffen R, Hagmann S, Henry M, Miller AO, Hale DC, Anand R, Gelman SS, Kozarsky PE, Fairley J, Franco-Paredes C, Barnett ED, Libman MD, Ward B, Maclean J, Tachikawa N, Kurai H, Sagara H, López-Vélez R, Pérez-Molina JA, Chen LH, Wilson ME, Cahill JD, McKinley G, Licitra C, Crespo A, Doyle P, Ghesquiere W, Stauffer WM, Walker PF, Gurtman A, Basto F, Abreu C, Hynes N, Sack R, McKenzie R, Askling HH, Bronner U, Lalloo DG, Beeching NJ, Connor BA, Shaw M, Hern A, Haulman N, Roesel D, Jong EC, Field V, Lim PL, Wilder-Smith A, Piyaphanee W, Silachamroon U, Lynch MW, Tenenboim S, McLellan S, Mendelson M,

Vincent P, Vincelette J, Holtom P, Goad J, Anglim A, Wang A, Eason J, MacDonald S, Nutman TB, Klion AD, Phu PT, Anderson N, Batchelor T, Meisch D, Muller R, Valdez LM, Siu H, Kass R, Perret C, Valdivieso F, Yates J, Ansdell V, Anderson S.

We performed a descriptive analysis of acute and potentially life-threatening tropical diseases among 82,825 ill western travelers reported to GeoSentinel from June of 1996 to August of 2011. We identified 3,655 patients (4.4%) with a total of 3,666 diagnoses representing 13 diseases, including falciparum malaria (76.9%), enteric fever (18.1%), and leptospirosis (2.4%). Ninety-one percent of the patients had fever; the median time from travel to presentation was 16 days. Thirteen (0.4%) patients died: 10 with falciparum malaria, 2 with melioidosis, and 1 with severe dengue. Falciparum malaria was mainly acquired in West Africa, and enteric fever was largely contracted on the Indian subcontinent; leptospirosis, scrub typhus, and murine typhus were principally acquired in Southeast Asia. Western physicians seeing febrile and recently returned travelers from the tropics need to consider a wide profile of potentially life-threatening tropical illnesses, with a specific focus on the most likely diseases described in our large case series.

DOI: 10.4269/ajtmh.12-0551
PMCID: PMC3583336
PMID: 23324216 [PubMed - indexed for MEDLINE]

389. Acta Med Biol (Niigata). 1967 Dec;15:69-85.

Epidemiological survey by means of complement fixation test on scrub typhus in Japan.

Kitaoka M, Okubo K, Asanuma K.

PMID: 4968953 [PubMed - indexed for MEDLINE]

390. Lin Chung Er Bi Yan Hou Tou Jing Wai Ke Za Zhi. 2013 Sep;27(17):964.

[Nasal bleeding as the first symptom of tsutsugamushi disease: a case report].

[Article in Chinese]

Tang Z, Wang J, Tu ZF.

A case of 76-years-old male patient with nasal bleeding as the first symptom in our hospital, who was finally diagnosed as tsutsugamushi disease. This old man was bited by insect in farmland 2 days before the symptom occurred. PE: Left thigh and right buttock have eschar, with splenomegaly. Routine blood test: WBC (decrease) 3.9 x 10(9)/L, RBC (decrease) 3.86 x 10(9)/L, PLT (decrease) 41 x 10(9)/L, HGB (decrease) 117 g/L; Chest CT: lung interstitial pneumonia, a small amount of bilateral pleural effusion. Oxk-ag 1:320. The patient was discharged after treatment with chloramphenicol for 8 days.

PMID: 24358803 [PubMed - indexed for MEDLINE]

391. Australas Ann Med. 1961 Nov;10:256-67.

The incidence and distribution of scrub typhus in North Queensland.

DERRICK EH.

PMID: 13885615 [PubMed - indexed for MEDLINE]

392. J Pediatr. 2016 Feb;169:321-321.e1. doi: 10.1016/j.jpeds.2015.10.088. Epub 2015 Nov 23.

Fever, Hepatosplenomegaly, and a Typical Skin Lesion.

Sarthak S(1), Samprathi M(2).

Author information:
(1)All India Institute of Medical Sciences, Bhubaneswar, India. (2)Department of Pediatrics, All India Institute of Medical Sciences, Bhubaneswar, India.

DOI: 10.1016/j.jpeds.2015.10.088
PMID: 26616252 [PubMed - indexed for MEDLINE]

393. PLoS One. 2014 Apr 22;9(4):e95810. doi: 10.1371/journal.pone.0095810. eCollection 2014.

Human spotted fever group rickettsioses are underappreciated in southern Taiwan, particularly for the species closely-related to Rickettsia felis.

Lai CH(1), Chang LL(2), Lin JN(3), Tsai KH(4), Hung YC(5), Kuo LL(6), Lin HH(7), Chen YH(8).

Author information:
(1)Graduate Institute of Medicine, College of Medicine, Kaohsiung Medical University, Kaohsiung City, Taiwan; Division of Infectious Diseases, Department of Internal Medicine, E-Da Hospital/I-Shou University, Kaohsiung City, Taiwan; Division of Infection Control Laboratory, E-Da Hospital/I-Shou University, Kaohsiung City, Taiwan. (2)Graduate Institute of Medicine, College of Medicine, Kaohsiung Medical University, Kaohsiung City, Taiwan; Faculty of Medicine, Department of Microbiology, College of Medicine, Kaohsiung Medical University, Kaohsiung City, Taiwan. (3)Graduate Institute of Medicine, College of Medicine, Kaohsiung Medical University, Kaohsiung City, Taiwan; Division of Infectious Diseases, Department of Internal Medicine, E-Da Hospital/I-Shou University, Kaohsiung City, Taiwan. (4)Institute of Environmental Health, College of Public, Health, National Taiwan University, Taipei City, Taiwan; Infectious Diseases Research and Education Center, Ministry of Health and Welfare and National Taiwan University, Taipei City, Taiwan. (5)Division of Infection Control Laboratory, E-Da Hospital/I-Shou University, Kaohsiung City, Taiwan. (6)Graduate Institute of Medicine, College of Medicine, Kaohsiung Medical University, Kaohsiung City, Taiwan; Research and Diagnostic Center, Centers for Disease Control, Department

of Health, Taipei City, Taiwan. (7)Division of Infectious Diseases, Department of Internal Medicine, E-Da Hospital/I-Shou University, Kaohsiung City, Taiwan; Institute of Clinical Medicine, National Yang-Ming University, Taipei City, Taiwan. (8)Graduate Institute of Medicine, College of Medicine, Kaohsiung Medical University, Kaohsiung City, Taiwan; Division of Infectious Diseases, Department of Internal Medicine, Kaohsiung Medical University Hospital, Kaohsiung Medical University, Kaohsiung City, Taiwan; School of Medicine, College of Medicine, Kaohsiung Medical University, Kaohsiung City, Taiwan.

BACKGROUND: Despite increased identification of spotted fever group rickettsioses (SFGR) in animals and arthropods, human SFGR are poorly characterized in Taiwan.
METHODS: Patients with suspected Q fever, scrub typhus, murine typhus, leptospirosis, and dengue fever from April 2004 to December 2009 were retrospectively investigated for SFGR antibodies (Abs). Sera were screened for Rickettsia rickettsii Abs by indirect immunofluorescence antibody assay (IFA), and those with positive results were further examined for Abs against R. rickettsii, R. typhi, R. felis, R. conorii, and R. japonica using micro-immunofluorescence (MIF) tests. Polymerase chain reaction (PCR) for detection of SFGR DNA was applied in those indicated acute infections. Case geographic distribution was made by the geographic information system software.
RESULTS: A total of 413 cases with paired serum, including 90 cases of Q fever, 47 cases of scrub typhus, 12 cases of murine typhus, 6 cases of leptospirosis, 3 cases of dengue fever, and 255 cases of unknown febrile diseases were investigated. Using IFA tests, a total of 49 cases with 47 (11.4%) and 4 (1.0%) cases had sera potentially positive for R. rickettsii IgG and IgM, respectively. In the 49 cases screened from IFA, MIF tests revealed that there were 5 cases of acute infections (3 possible R. felis and 2 undetermined SFGR) and 13 cases of past infections (3 possible R. felis and 10 undetermined SFGR). None of the 5 cases of acute infection had detectable SFGR DNA in the blood specimen by PCR. Possible acute infection of R. felis was identified in both one case of Q fever and scrub typhus. The geographic distribution of SFGR cases is similar with that of scrub typhus.
CONCLUSIONS: Human SFGR exist and are neglected diseases in southern Taiwan, particularly for the species closely-related to R. felis.

DOI: 10.1371/journal.pone.0095810
PMCID: PMC3995941
PMID: 24755560 [PubMed - indexed for MEDLINE]

394. Eur J Epidemiol. 1991 May;7(3):304-6.

Recent studies on scrub typhus and Rickettsia tsutsugamushi in Shandong Province--China.

Xiangrui C(1), Jinju W, Yongguo Z, Yanan W, Chunmu Y, Zhaoping X, Junli W, Yufu Y.

Author information:
(1)Institute of Microbiology and Epidemiology, Beijing, China.

In an outbreak of scrub typhus occurred 138 cases in 1986 in Menying County of Shandong Province of China which were confirmed through clinical and epidemiological surveys and laboratory techniques. In the endemic areas, the

predominant species of mite is Leptotrombidium (L.) scutelarae and the predominant rodent is Apodemus agrarius, which may be the main vector and reservoir. Several strains of Rickettsia tsutsugamushi (R.T.) were obtained from mites, wild mice and patients, of which 3 human strains (SDH871, SDH875, SDH878) cause illness and some deaths in mice, except SDH871. The LD50 of SDH878 is 3.4. Cross-protection was observed among the strains, SDH871, SDH878, and prototype strain karp. Also the antibodies in mucoid peritoneal fluid were detectable with high titer. Shandong R.T. belongs to the serotype of the Gilliam strain.

PMID: 1909245 [PubMed - indexed for MEDLINE]

395. Kaohsiung J Med Sci. 2008 Feb;24(2):92-8. doi: 10.1016/S1607-551X(08)70103-7.

Immunohistochemical study of scrub typhus: a report of two cases.

Tseng BY(1), Yang HH, Liou JH, Chen LK, Hsu YH.

Author information:
(1)Department of Pathology, Buddhist Tzu Chi General Hospital and University, Hualien, Taiwan.

Scrub typhus is a zoonotic disease caused by Orientia tsutsugamushi, which is transmitted by chiggers. The target cells of this rickettsia are poorly defined in humans. Immunohistochemical staining of tissue sections of patients with scrub typhus is helpful in investigating the target cells of this rickettsia in different organs. We studied two autopsy specimens by immunohistochemical staining using a specific antibody against O. tsutsugamushi. Rickettsiae were located in endothelial cells in all of the organs evaluated, namely heart, lung, brain, kidney, appendix and skin, within cardiac muscle cells and renal tubular epithelial cells, and in macrophages located in the lymph node, liver and spleen. In conclusion, O. tsutsugamushi may disseminate into multiple organs through endothelial cells and macrophages, resulting in the development of fatal complications.

DOI: 10.1016/S1607-551X(08)70103-7
PMID: 18281226 [PubMed - indexed for MEDLINE]

396. Korean J Parasitol. 2016 Jun;54(3):307-13. doi: 10.3347/kjp.2016.54.3.307. Epub 2016 Jun 30.

Seroepidemiological Survey of Zoonotic Diseases in Small Mammals with PCR Detection of Orientia tsutsugamushi in Chiggers, Gwangju, Korea.

Park JW(1), Chung JK(1), Kim SH(1), Cho SJ(1), Ha YD(1), Jung SH(1), Park HJ(1), Song HJ(2), Lee JY(2), Kim DM(3), Pyus J(3), Ha DR(1), Kim ES(1), Lee JI(4).

Author information:
(1)Division of Infectious Disease Investigation, Health and Environment Research Institute of Gwangju City, Gwangju 61986, Korea. (2)Clinical Pathology, Gwangju Health University, Gwangju 62287, Korea. (3)Department of Internal Medicine, School of Medicine, Chosun University, Gwangju 61452, Korea. (4)Department of

Veterinary Public Health, College of Veterinary Medicine, Chonnam National
University, Gwangu 62287, Korea.

Serosurveillance for zoonotic diseases in small mammals and detection of chiggers, the vector of Orientia tsutsugamushi, were conducted from September 2014 to August 2015 in Gwangju Metropolitan Area. Apodemus agrarius was the most commonly collected small mammals (158; 91.8%), followed by Myodes regulus (8; 4.6%), and Crocidura lasiura (6; 3.5%). The highest seroprevalence of small mammals for O. tsutsugamushi (41; 26.3%) was followed by hantaviruses (24; 15.4%), Rickettsia spp. (22; 14.1%), and Leptospira (2; 1.3%). A total of 3,194 chiggers were collected from small mammals, and 1,236 of 3,194 chiggers were identified with 7 species of 3 genera: Leptotrombidium scutellare was the most commonly collected species (585; 47.3%), followed by L. orientale (422; 34.1%), Euchoengastia koreaensis (99; 8.0%), L. palpale (58; 4.7%), L. pallidum (36; 2.9%), Neotrombicula gardellai (28; 2.3%), and L. zetum (8; 0.6%). L. scutellare was the predominant species. Three of 1,236 chigger mites were positive for O. tsutsugamushi by PCR. As a result of phylogenetic analysis, the O. tsutsugamushi strain of chigger mites had sequence homology of 90.1-98.2% with Boryong. This study provides baseline data on the distribution of zoonotic diseases and potential vectors for the development of prevention strategies of vector borne diseases in Gwangju metropolitan area.

DOI: 10.3347/kjp.2016.54.3.307
PMCID: PMC4977789
PMID: 27417085 [PubMed - in process]

397. Am J Trop Med Hyg. 1974 Sep;23(5):993-9.

Transmission of Rickettsia orientalis to man by Leptotrombidium akamushi at a scrub typhus endemic area in Akita Prefecture, Japan.

Kitaoka M, Asanuma K, Otsuji J.

During the summer seasons of 1956 through 1970, 93 larval trombiculid mites were removed from 386 individuals who had been bitten by chiggers in Jumonji, Akita Prefecture. All 87 larvae that were available for examination were identified as Leptotrombidium akamushi. Infestation of man occurred predominantly during July and August, but the period extended from June to November. The duration of attachment was approximately 1 to 3 days. Usually only 1 chigger was found on a victim but on occasion as many as 7 were removed at one time. Scrub typhus developed in 45 (11.7%) of the 386 farmers bitten by chiggers. Although patients with scrub typhus may have sustained multiple bites, only one eschar was found. However, 76% of the patients did not recognize a bite either at the site where an eschar subsequently appeared or elsewhere prior to the onset of disease. If it is assumed that workers who did not subsequently become ill were equally unaware of chigger bites, then the probable incidence of disease in chigger victims was 3.1%. The minimum infectivity rate of L. akamushi larvae in Akita Prefecture was estimated to be 2.3%. Based upon the assumption that infection was transmitted as the result of a single bite, the infective rate of chiggers attacking man was calculated to be 2.5%.

PMID: 4451238 [PubMed - indexed for MEDLINE]

398. Am J Trop Med Hyg. 2009 Mar;80(3):442-6.

A community-based case-control study of behavioral factors associated with scrub typhus during the autumn epidemic season in South Korea.

Kweon SS(1), Choi JS, Lim HS, Kim JR, Kim KY, Ryu SY, Lee SD, Im HK, Kwon JW.

Author information:
(1)Jeonnam Regional Cancer Center, Chonnam National University Hwasun Hospital, Hwasun, South Korea. ujingogo@paran.com

A community-based, case-control study was carried out to investigate risk factors for scrub typhus in South Korea. Cases (n = 299) were defined as persons who were diagnosed serologically within the past two weeks. Two neighborhood control subjects were selected by matching for sex, age, and occupation. Taking a rest directly on the grass, working in short sleeves, working with bare hands, and squatting to defecate or urinate posed the highest risks, with adjusted odds ratios (aORs) and 95% confidence intervals (CIs) of 1.7 (1.2-2.3), 1.6 (1.1-2.4), 1.7 (1.2-2.4), and 2.0 (1.4-2.9), respectively. Wearing a long-sleeved shirt while working, keeping work clothes off the grass, and always using a mat to rest outdoors showed protective associations, with aORs and 95% CIs of 0.5 (0.3-0.9), 0.6 (0.4-0.9), and 0.7 (0.5-0.9), respectively. These results might be useful in the establishment of a detailed control strategy for scrub typhus.

PMID: 19270296 [PubMed - indexed for MEDLINE]

399. J Infect Dis. 1998 Mar;177(3):800-2.

Performance of a dot blot immunoassay for the rapid diagnosis of scrub typhus in a longitudinal case series.

Watt G(1), Strickman D, Kantipong P, Jongsakul K, Paxton H.

Author information:
(1)Department of Medicine, Armed Forces Research Institute of Medical Sciences, Bangkok, Thailand. LtcGeorgeWatt@wrsmtpCCmail.army.MIL

A rapid dipstick test for scrub typhus was prospectively evaluated in Chiangrai, northern Thailand. Sera from 162 patients with fever of unclear etiology were tested by a dot blot immunoassay using two different antigen concentrations. Dipsticks coated with lower concentration of antigen lacked sensitivity compared with the indirect immunoperoxidase test. Dipsticks with higher antigen concentration had increased sensitivity that was equivalent to that of the immunoperoxidase test. By increasing the antigen concentration on the dipstick, sensitivity increased from 67% to 100%, positive predictive value increased from 90% to 93%, and negative predictive value rose from 92% to 100%. The specificity of both antigen concentrations was 98%. This study establishes that scrub typhus can be confirmed serologically by use of a dipstick assay and that serodiagnosis can be effectively tailored to a target population.

PMID: 9498469 [PubMed - indexed for MEDLINE]

400. Trop Geogr Med. 1976 Dec;28(4):303-8.

The prevalence of scrub typhus antibodies in residents of West Malaysia.

Robinson DM, Gan E, Donaldson JR.

Based on the prevalence of antibody, an estimated 3% of the population of rural Malaysia is infected with Rickettsia tsutsugamushi each year, resulting in positive antibody rates in focal areas of 6 to 69%. Most of these infections do not appear to produce clinical scrub typhus. A wide range of seropositivity rates was found in areas otherwise resembling each other in predominant occupation, terrain, and nearby habitat. The prevalence rates however were significantly higher in people who worked in forested areas and significantly lower in people with urban occupations.

PMID: 827831 [PubMed - indexed for MEDLINE]

401. Southeast Asian J Trop Med Public Health. 1984 Sep;15(3):402-6.

Scrub typhus and chiggers in northern Thailand.

Takada N, Khamboonruang C, Yamaguchi T, Thitasut P, Vajrasthira S.

It was reconfirmed that L. deliense was the most dominant chigger during the rainy season in northern Thailand. Overall prevalence of IgG antibody against R. tsutsugamushi among the rural villagers in the northern most part of Thailand was nearly 60% at the level over 1 : 10 by an indirect immunofluorescent test. The level of IgG and IgM antibody titers was considerably high as greater than or equal to 1 : 2560 in some sera, but did not markedly vary by age groups or sexes. Based on detection of IgM antibody, the probability of new or reinfections were considered.

PMID: 6441267 [PubMed - indexed for MEDLINE]

402. Trop Geogr Med. 1975 Jun;27(2):143-50.

Clinical observations of scrub typhus on Penghu (the Pescadores Islands).

Fang RC, Lin WP, Chao PS, Kuo NT, Chen CM.

Between May and September 1973, 68 cases of scrub typhus in Chinese military personnel on the Pescadores Islands were studied. The common symptoms and signs were fever, chills, headache, eschar, myalgia, and lymph node enlargement. Most eschars were located in the axilla, waist, groin and genitals, and neck. These lesions were painless and not noticed by the patients themselves. Regional lymph node enlargement at the site of eschar drainage was common. Relative bradycardia

with fever was observed in 40%, a skin rash in 35% of the patients. Leucopenia was noted more frequently in the febrile than in the convalescent stage, but more than half of the patients had a normal count. Lymphocytosis was prominent, especially during the convalescent period. An acceleration of ESR was noted. Instead of depression of the erythroid series in the marrow which was reported previously, 47% of examined patients were found to have erythroid hyperplasia. Two patients showed marked hypocellularity of the marrow in the acute febrile stage; later on became normocellular. Albuminuria was present in 15 and BUN increased in 12 patients. Elevation of serum bilirubin and SGOT was also noted. Biologic false positive VDRL tests were observed in nine patients. In 30 tests elevation of Proteus OX-K titres between 1:160 and 1:640 was noted. A geometric mean OX-K titre rise in the patients is presented; the mean titre reached a peak in the third week of illness, and then fell off. Most of the patients were treated with tetracycline 500 mg every six hours for about nine days. The fever usually subsided within 36 hours. Complications or mortality were not encountered.

PMID: 1179479 [PubMed - indexed for MEDLINE]

403. Bull World Health Organ. 1968;39(2):209-18.

Ecological considerations in scrub typhus. 1. Emerging concepts.

Traub R, Wisseman CL Jr.

Scrub typhus infection is now known to occur in geographical and ecological areas where its presence was hitherto unsuspected-north and west of the Indus River and in south-eastern Siberia; in semi-desert, montane desert, and alpine terrain high in the Himalayas, as well as in primary jungle. Additional data are presented to support the hypothesis that "ecological islands", containing basically similar faunas of rodents and ectoparasites, exist on scattered mountains in the Pakistan Himalayas, despite the intervening "barriers" of desert, broad rivers and massive peaks. Since scrub typhus has been demonstrated in some of these isolated areas, it is felt that the infection may exist, unrecognized, in neighbouring countries as well.A number of larval trombiculid mites, largely species of the subgenus Leptotrombidium, are believed to be vectors, in addition to the well-known L. (L.) deliense and L. (L.) akamushi. The host-range of natural infection in ground-dwelling small mammals, especially rodents, is very broad in endemic areas. An important factor of time, and not only of space, may be involved in an endemic locus, the disease undergoing a sequential evolution involving different chiggers and rodents over a period of years. It is pointed out that new irrigation schemes, road construction, and agricultural projects may introduce scrub typhus into an area or greatly increase its endemicity if the infection is already present.

PMCID: PMC2554547
PMID: 5303404 [PubMed - indexed for MEDLINE]

404. Am J Gastroenterol. 2009 Apr;104(4):1067. doi: 10.1038/ajg.2009.8. Epub 2009 Mar 3.

An unusual presentation of scrub typhus with atraumatic hemoperitoneum.

Lin WY, Lin GM, Chang FY.

DOI: 10.1038/ajg.2009.8
PMID: 19259082 [PubMed - indexed for MEDLINE]

405. Clin Infect Dis. 2006 Jan 1;42(1):e6-8. Epub 2005 Nov 29.

Acute hearing loss due to scrub typhus: a forgotten complication of a reemerging disease.

Premaratna R(1), Chandrasena TG, Dassayake AS, Loftis AD, Dasch GA, de Silva HJ.

Author information:
(1)Department of Medicine, Faculty of Medicine, University of Kelaniya, Sri Lanka. ranjan_premaratna@lycos.com

Comment in
  Clin Infect Dis. 2006 May 15;42(10):1505-6.
  Clin Infect Dis. 2006 May 15;42(10):1506.

We describe 6 patients with scrub typhus who presented with acute hearing loss, a forgotten complication of this reemerging disease. They were admitted with fever of 10-14 days' duration and had clinical evidence of deafness and pneumonitis. Five patients had eschars, which prompted the diagnosis of typhus fever and led to early institution of treatment. Deafness has been described as a clue to the diagnosis of scrub typhus; awareness of this symptom facilitated early diagnosis in 4 of 5 patients who recovered. Acute hearing loss or hearing impairment in a febrile patient should arouse strong suspicion of scrub typhus.

DOI: 10.1086/498747
PMID: 16323083 [PubMed - indexed for MEDLINE]

406. J Microbiol Immunol Infect. 1999 Mar;32(1):57-62.

Meningoencephalitis, myocarditis and disseminated intravascular coagulation in a patient with scrub typhus.

Ben RJ(1), Feng NH, Ku CS.

Author information:
(1)Department of Internal Medicine, Armed Forces Kaohsiung General Hospital, Taiwan, ROC.

A 21-year-old male soldier was admitted due to a sore throat, headache, generalized lymphadenopathy and persistent fever for 12 days. Despite empirical antibiotic treatment for four days at a clinic prior to admission, he continued to have persistent abdominal pain over his right upper quadrant region and progressive jaundice was followed by shock. After admission, he developed an episode of clonic seizures and became delirious and agitated. An

electrocardiogram showed first degree atrioventricular (AV) block and non-specific ST-T wave changes. Hematological studies revealed thrombocytopenia, hypofibrinogenemia, abnormal partial thromboplastin time (PTT) and a positive test for D-dimer. The cerebrospinal fluid analysis showed pleocytosis with white cells of 84/mm3 with a lymphocyte predominance, protein of 97 mg/dL and glucose of 79 mg/dL. Indirect immunofluorescence assay showed a fourfold rise in antibodies to Orientia tsutsugamushi in paired serum with IgM antibody titer of 1:640. The patient had a favorable response after parenteral chloramphenicol in addition to oral tetracycline. Early ricognition of scrub typhus and early prescription of anti-rickettsial agents prevent complications of central nervous system involvement and further deterioration of cardiac and hematological function.

PMID: 11561571 [PubMed - indexed for MEDLINE]

407. Bull Soc Pathol Exot Filiales. 1964 Mar-Apr;57:277-83.

[ENDEMIC FACTORS IN SCRUB TYPHUS IN INDOCHINA].

[Article in French]

LEGAC P, ARQUIE E.

PMID: 14184779 [PubMed - indexed for MEDLINE]

408. Zhonghua Yi Xue Za Zhi (Taipei). 1992 Jan;49(1):61-3.

Scrub typhus and pregnancy: a case report and literature review.

Tsui MS(1), Fang RC, Su YM, Li YT, Lin HM, Sun LS, Tu FC.

Author information:
(1)Department of Obstetrics and Gynecology, Far Eastern Memorial Hospital, Panchiao, Taiwan, R.O.C.

Scrub typhus is still prevalent on Penghu Islands. We herein report a case in a pregnant woman who had been to Yi-Lan County in Taiwan. The patient responded well to Minocycline (Minocin) therapy without complication. Her newborn baby was found not complicated with the disease. The relative literature is reviewed.

PMID: 1312388 [PubMed - indexed for MEDLINE]

409. Infection. 1992 May-Jun;20(3):153-4.

Scrub typhus: an imported Rickettsial disease.

Dupon M(1), Rogues AM, Malou M, d'Ivernois C, Lacut JY.

Author information:
(1)Service de Médecine Interne et Maladies Infectieuses, Tripode 9e étage, Hôpital Pellégrin, Bordeaux, France.

A case of scrub typhus due to Rickettsia tsutsugamushi is reported. This imported rickettsial disease was contracted by a 30-year-old woman while traveling in Thailand, and was transmitted by an infected mite's bite. Diagnosis was confirmed by specific serology and resolution was obtained by tetracycline therapy. Current concepts of the disease are reviewed.

PMID: 1644492 [PubMed - indexed for MEDLINE]

410. Am J Trop Med Hyg. 2010 Oct;83(4):930-5. doi: 10.4269/ajtmh.2010.09-0791.

Antigenic drift of Orientia tsutsugamushi in South Korea as identified by the sequence analysis of a 56-kDa protein-encoding gene.

Park SW(1), Lee CK, Kwak YG, Moon C, Kim BN, Kim ES, Kang JM, Lee CS.

Author information:
(1)Department of Internal Medicine, Seoul National University College of Medicine and Boramae Medical Center, Seoul, Republic of Korea. hswon1@snu.ac.kr

This study was performed to determine the nationwide antigenic diversity of Orientia tsutsugamushi in South Korea. Sequence analysis was performed around variable domains I and II of a 56-kDa protein-encoding gene. We used eschar to overcome the disadvantages of conventional serotyping. The serological passive hemagglutination assay (PHA) was assessed based on the genotyping results. We analyzed 153 isolates from scrub typhus patients in major endemic areas and found that Boryong was the major strain (68.6%). New strains were also identified: Taguchi (19.6%), Kanda/Kawasaki (9.2%), and UAP7 (1.3%). PHA yielded significantly fewer positive results among Kawasaki strains ($P < 0.001$), which are not included in the PHA antigen panel. In South Korea, Boryong was still the predominant strain, but the sequence analysis identified new changes in minor strains (30.1%). This antigenic drift had a negative effect on the PHA results. Periodic surveillance of the contemporary strains using sequence analysis is needed.

DOI: 10.4269/ajtmh.2010.09-0791
PMCID: PMC2946772
PMID: 20889895 [PubMed - indexed for MEDLINE]

411. Trans R Soc Trop Med Hyg. 1980;74(2):253-7.

The longevity of antibody to Rickettsia tsutsugamushi in patients with confirmed scrub typhus.

Saunders JP, Brown GW, Shirai A, Huxsoll DL.

Serological surveillance for up to two years of 114 patients with laboratory confirmed scrub typhus showed that antibody to Rickettsia tsutsugamushi as

demonstrated by the indirect fluorescent antibody test is short-lived. The mean reversion time from mean peak titre (1:499) was 48.9 weeks and the calculated annual reversion rate to a titre less than 1:50 was 61%. This can be used to estimate attack rates based on point prevalence of antibody. The relationship between antibody prevalence and attack rates observed by other workers was confirmed using this model. The possible uses of the finding and its implications in Malaysia are briefly discussed.

PMID: 6770503 [PubMed - indexed for MEDLINE]

412. J Travel Med. 2007 Sep-Oct;14(5):352-5.

Travel-acquired scrub typhus: emphasis on the differential diagnosis, treatment, and prevention strategies.

Nachega JB(1), Bottieau E, Zech F, Van Gompel A.

Author information:
(1)Department of International Health, Johns Hopkins University, Bloomberg School of Public Health, Baltimore, MD 21205, USA, and Department of Internal Medicine, St-Luc University Hospital, Brussels, Belgium. jnachega@jhsph.edu

Scrub typhus should be considered in any febrile patient presenting with a macular rash, a polyadenopathy, an eschar, or a history of environmental exposure in endemic areas. The differential diagnosis includes malaria, typhoid fever, leptospirosis, and arboviroses. Doxycycline 100 mg twice daily for 7 days should be initiated as soon as the disease is suspected.

DOI: 10.1111/j.1708-8305.2007.00151.x
PMID: 17883470 [PubMed - indexed for MEDLINE]

413. World J Gastroenterol. 2007 Jul 7;13(25):3523-5.

Pancreatic abscess following scrub typhus associated with multiorgan failure.

Yi SY(1), Tae JH.

Author information:
(1)Department of Internal Medicine, School of Medicine, Ewha Womans University, Yangcheongu Mokdong 911-1, Seoul 158-710, South Korea. syy@ewha.ac.kr

Clinical severity of scrub typhus ranges from mild to fatal. Acute pancreatitis with abscess formation is a rare complication among patients with scrub typhus. This paper reports a case of scrub typhus in a 75 years old man with acute pancreatitis with abscess formation and multiorgan failure. Abdominal computed tomography showed multiple infected pancreatic pseudocysts with peri-pancreatic infiltration. Multiorgan failure was successfully treated with doxycycline, ceftriaxone, and supportive management. The pancreatic abscess was successfully drained percutaneously and the sizes of pseudocysts decreased remarkably.

PMCID: PMC4146791
PMID: 17659702 [PubMed - indexed for MEDLINE]

414. Int J Cardiol. 2007 Jan 2;114(1):e18-20. Epub 2006 Oct 19.

Acute myocardial infarction following scrub typhus infection.

Kim DG, Kim JW, Choi YS, Kim SH, Kim SM, Park CG, Seo HS, Oh DJ.

Erratum in
Int J Cardiol. 2007 Dec 15;123(1):73. Choi, Yoon Sun [corrected to Choi, Youn Seon].

Up to recently, a few cases of myocarditis in the cardiac manifestations of scrub typhus have been reported. However, acute myocardial infarction (AMI) associated with scrub typhus has not been previously reported. We presented a case of AMI ensued on scrub typhus, which were successfully treated by percutaneous coronary intervention (PCI).

DOI: 10.1016/j.ijcard.2006.07.131
PMID: 17055080 [PubMed - indexed for MEDLINE]

415. Trop Doct. 2002 Apr;32(2):115-6.

Scrub typhus: a rare cause of multiorgan failure in a surgical patient presenting with deep venous thrombosis.

Natarajan A(1), Rozario A, Santhosh AO, Kanth CN.

Author information:
(1)Department of Surgery, St John's Medical College Hospital, Bangalore, Karnataka, India. natraj_muthu@yahoo.com

PMID: 11933899 [PubMed - indexed for MEDLINE]

416. Scand J Infect Dis. 1996;28(4):411-2.

Transmission of scrub typhus by needlestick from a patient receiving pefloxacin.

Jee HG(1), Chung MH, Lee SG, Kim IS, Chang WH.

Author information:
(1)Department of Internal Medicine, Chungang Gil Hospital, Inchon, Republic of Korea.

A nurse experience a needlestick during the care of a patient with scrub typhus, treated with pefloxacin (400 mg twice daily) and cefazolin. Seven days after the needlestick, pain and erythematous swelling developed at the tip of her left fourth finger, the site of the needlestick. Fever and headache developed 10 days later, and her skin lesion became a small vesicle surrounded by a dark

erythematous area. The diagnosis of scrub typhus was made by a rising indirect immunofluorescent antibody titre, and her illness was improved with doxycycline.

PMID: 8893408 [PubMed - indexed for MEDLINE]

417. Scand J Infect Dis. 2007;39(9):826-8.

Guillain-Barré syndrome associated with scrub typhus.

Lee SH(1), Jung SI, Park KH, Choi SM, Park MS, Kim BC, Kim MK, Cho KH.

Author information:
(1)Department of Neurology, Chonnam National University Medical School, Gwangju, South Korea. shleemd@naver.com

We report a 42-y-old female with Guillain-Barré syndrome (GBS) who presented with scrub typhus for a duration of 2 weeks. Subsequently, ascending paralysis and facial diplegia developed. GBS was confirmed with nerve conduction studies and cerebrospinal fluid examinations. After administration of intravenous immunoglobulin, symptoms gradually disappeared.

DOI: 10.1080/00365540701266755
PMID: 17701724 [PubMed - indexed for MEDLINE]

418. Zhonghua Yi Xue Za Zhi (Taipei). 1995 Sep;56(3):205-10.

Scrub typhus complicating acute respiratory distress syndrome: a report of two cases.

Lee WS(1), Wang FD, Wang LS, Wong WW, Young D, Fung CP, Liu CY.

Author information:
(1)Department of Medicine, Veterans General Hospital-Taipei, Taiwan, R.O.C.

Scrub typhus is a zoonotic disease, caused by Rickettsia tsutsugamushi, in which humans are accidental hosts. Although it is an acute febrile illness, severe complications of this disease are very rare since the introduction of specific antibiotic therapy. The authors report two cases of scrub typhus complicating acute respiratory distress syndrome. The first case progressed to multiple organ failure, and the patient expired. In the second case, the patient recovered and was discharged. These two cases were proved to be scrub typhus by their travel history or their having lived in endemic area, clinical manifestations, an eschar and indirect immunofluorescent antibody test. For a good prognosis, early diagnosis and early treatment of this disease are important.

PMID: 8854444 [PubMed - indexed for MEDLINE]

419. Zhonghua Yi Xue Za Zhi (Taipei). 1995 May;55(5):401-4.

Unusual presentation of acute abdomen in scrub typhus: a report of two cases.

Yang CH(1), Young TG, Peng MY, Hsu GJ.

Author information:
(1)Department of Internal Medicine, Tri-Service General Hospital, National Defense Medical Center, Taipei, Taiwan, R.O.C.

Two young soldiers presented with acute abdomens, then received surgical procedures under initial impression of acute cholecystitis and acute appendicitis respectively. Operative findings did not confirm the initial diagnosis, and the clinical condition did not improve after operation. Scrub typhus was suggested later by clinical manifestations of fever, chills, headache, lymphadenopathy, skin rash and presence of eschar formation; this diagnosis was finally confirmed by positive serologic results of high Weil-Felix OXK agglutination and/or Rickettsia tsutsugammushi immunoflorescence titers in paired sera. Both patients rapidly became afebrile after administration of tetracycline. This unusual presentation with acute abdomen in scrub typhus is emphasized, with caution that the possibility of scrub typhus should be taken considered, especially in patients coming from hyperendemic areas.

PMID: 7641127 [PubMed - indexed for MEDLINE]

420. Nihon Densenbyo Gakkai Zasshi. 1961 Dec;35:577-83.

[A small outbreak of scrub typhus at the foot of Mount Fuji. I. Epidemiology and clinical aspects].

[Article in Japanese]

OKAMURA M, OHHASHI T, KAWAMURA H, SHISHIDO A, KITAOKA M.

PMID: 14481531 [PubMed - indexed for MEDLINE]

421. Zhonghua Liu Xing Bing Xue Za Zhi. 1994 Feb;15(1):31-3.

[A new natural focus of scrub typhus found in Hunchun].

[Article in Chinese]

Lu Z(1), Hu L, Chai Z.

Author information:
(1)Institute of Military Medicine, Shenyang.

From May to June 1992, Apodemus agrarius and Apodemus speciosus were captured in Hunchun, Jilin Province. Four strains of Rickettsia tsutsugamushi were isolated from viscera of rats and trombiculid mites. At the same time, the antibody against Rickettsia tsutsugamushi was assayed in the sera of the local people and the wild rats. The positive rates were 15.2% and 16.4%, respectively. The above

results showed that a natural focus of scrub typhus exists in Hunchun area.

PMID: 8082138 [PubMed - indexed for MEDLINE]

422. J Hyg Epidemiol Microbiol Immunol. 1968;12(3):257-64.

The investigation of scrub typhus in the USSR.

Kulagin SM, Tarasevic IV, Kudryasová NI, Plotnikova LF.

PMID: 4239372 [PubMed - indexed for MEDLINE]

423. Am J Hyg. 1949 Jul;50(1):63-74.

Chloramphenicol in the chemoprophylaxis of scrub typhus; epidemiological observations on hyperendemic areas of scrub typhus in Malaya.

PHILIP CB, TRAUB R, SMADEL JE.

PMID: 18135592 [PubMed - indexed for MEDLINE]

424. Am J Hyg. 1949 Jul;50(1):75-91.

Chloramphenicol in the chemoprophylaxis of scrub typhus; results with volunteers exposed in hyperendemic areas of scrub typhus.

SMADEL JE, TRAUB R, et al.

PMID: 18135593 [PubMed - indexed for MEDLINE]

425. Trans R Soc Trop Med Hyg. 1947 Mar;40(4):359.

Epidemiology of scrub typhus in Assam and Burma.

AUDY JR.

PMID: 20341936 [PubMed - indexed for MEDLINE]

426. Acta Med Biol (Niigata). 1967 Dec;15:43-8.

Recent studies of the epidemiology of scrub typhus in North Queensland.

Domrow R, Cook I.

PMID: 4968950 [PubMed - indexed for MEDLINE]

427. J Trop Pediatr. 2006 Jun;52(3):228-9. Epub 2005 Nov 16.

Magnitude and features of scrub typhus and spotted fever in children in India.

Somashekar HR, Moses PD, Pavithran S, Mathew LG, Agarwal I, Rolain JM, Raoult D, Varghese GM, Mathai E.

DOI: 10.1093/tropej/fmi096
PMID: 16291832 [PubMed - indexed for MEDLINE]

428. Jpn J Infect Dis. 2006 Jun;59(3):207-8.

Scrub typhus (tsutsugamushi disease) in Kanagawa Prefecture in 2001-2005.

Katayama T(1), Hara M, Furuya Y, Nikkawa T, Ogasawara H.

Author information:
(1)Department of Biology, Kanagawa Prefectural Institute of Public Health, Chigasaki-shi, Kanagawa 253-0087, Japan. katayama.zups@pref.kanagawa.jp

PMID: 16785710 [PubMed - indexed for MEDLINE]

429. Clin Infect Dis. 2004 Nov 1;39(9):1395-6.

Scrub typhus: prevalence and diagnostic issues in rural Southern India.

Isaac R, Varghese GM, Mathai E, J M, Joseph I.

DOI: 10.1086/424748
PMID: 15494919 [PubMed - indexed for MEDLINE]

430. J Assoc Physicians India. 2009 Oct;57:720-1.

Scrub typhus complicating pregnancy.

Mahajan SK, Rolain JM, Kashyap R, Gupta D, Thakur S, Sharma A, Kaushal SS, Raoult D.

PMID: 20329435 [PubMed - indexed for MEDLINE]

431. Trans R Soc Trop Med Hyg. 1975;69(1):121-30.

Mammals and scrub typhus ecology in peninsular Malaysia.

Muul I, Liat LB, Walker JS.

The overall comparisons of habitats are given in (Table III). The habitats are arranged in order of extent of alterations by man, with the least disturbed at the top. The highest average blood isolation rates came from the least disturbed areas. The highest monthly maximal rickettsial isolation rates from blood and maximal prevalence rates of antibody per month were also obtained at Bukit Lanjan, the habitat least altered by activities of man. The lowest average blood isolation rate (6%) and the lowest monthly maximal rickettsial isolation and antibody prevalence rates were obtained at Bukit Mandol, the habitat most extensively and intensively altered by man. The intermediate habitats had intermediate rates. We caution anyone interpreting these observations, however, in terms of human disease, which seem to be associated with hyperendemic foci. Here we are not dealing with hyperendemicity from the standpoint of human disease, but present evidence of widespread endemicity from which hyperendemic foci may derive. Also, we have not yet identified the prevalent strains and do not know their infectivity to man.

PMID: 806995 [PubMed - indexed for MEDLINE]

432. Nihon Rinsho. 2007 Mar 28;65 Suppl 3:208-11.

[Tsutsugamushi disease (scrub typhus)].

[Article in Japanese]

Tachibana N(1), Okayama A.

Author information:
(1)Miyazaki Wakahisa Hospital.

PMID: 17494150 [PubMed - indexed for MEDLINE]

433. Am J Trop Med Hyg. 1981 Jul;30(4):849-54.

Scrub typhus in the Eastern Solomon Islands and Northern Vanuatu (New Hebrides).

Miles JA, Austin FJ, Jennings LC.

Rickettsia tsutsugmushi has been isolated from Rattus rattus from Vanua Lava island in Northern Vanuatu (New Hebrides) and from R. exulans and Leptotrombidium akamushi on Ndende island in the Eastern Solomon Islands. The well-known vector mite L. deliense was found on Mota Lava and Vanua Lava in Vanuatu, but no isolation was made from pools of this mite. Serology confirms that R. tsutsugamushi infects humans in the Banks group of islands in Northern Vanuatu and that infection is much more widespread in the Solomon Islands than the limited isolations indicate.

PMID: 6789693 [PubMed - indexed for MEDLINE]

434. Am J Trop Med Hyg. 1973 Jul;22(4):503-8.

Some epidemiological considerations of scrub typhus (Rickettsia tsutsugamushi) in a natural focus in the Zambales Mountains, Luzon, Republic of the Philippines.

Reisen WK, Pollard TJ, Tardy WJ.

PMID: 4197953 [PubMed - indexed for MEDLINE]

435. J R Soc Med. 1978 Jul;71(7):507-10.

Recent studies in scrub typhus: a review.

Brown GW.

PMCID: PMC1436672
PMID: 359809 [PubMed - indexed for MEDLINE]

436. Ala J Med Sci. 1971 Jan;8(1):75-81.

Tetracycline therapy for scrub typhus.

Sheehy TW.

PMID: 5578503 [PubMed - indexed for MEDLINE]

437. Jpn J Med Sci Biol. 1980 Oct;33(5):277-82.

Leptotrombidium (Leptotrombidium) Umbricola, new species, a probable vector of scrub typhus in Peninsular Malaysia.

Nadchatram M, Dohany AL.

Leptotrombidium (Leptotrombidium) umbricola, described here as a new species, is a member of the L. (L.) deliense group and most closely resembles L. (L.) vivericola. L. (L.) umbricola was collected from the ground surface and from animal hosts, in similar habitats to the scrub typhus vector, L. (L.) deliense. The host and habitat distribution records and the Rickettsia tsutsugamushi infection rates within unengorged specimens suggest that L. (L.) umbricola may be an important vector of scrub typhus in Peninsular Malaysia.

PMID: 7300039 [PubMed - indexed for MEDLINE]

438. J Neuropathol Exp Neurol. 1946 Oct;5:271-84.

The distribution of the pathologic lesions of the central nervous system in scrub typhus (tsutsugamushi disease).

WEIL A, HAYMAKER W.

PMID: 21000555 [PubMed - indexed for MEDLINE]

439. Integr Zool. 2008 Dec;3(4):267-73. doi: 10.1111/j.1749-4877.2008.00100.x.

Surveys of rodent-borne disease in Thailand with a focus on scrub typhus assessment.

Lerdthusnee K(1), Nigro J, Monkanna T, Leepitakrat W, Leepitakrat S, Insuan S, Charoensongsermkit W, Khlaimanee N, Akkagraisee W, Chayapum K, Jones JW.

Author information:
(1)Department of Entomology, United States Army Medical Component, Armed Forces Research Institute of Medical Sciences, Bangkok, Thailand Science Systems and Applications, National Aeronautics and Space Administration - Goddard Space Flight Center NASA-GSFC, Greenbelt, Maryland, USA.

The epidemiology of many rodent-borne diseases in South-East Asia remains ill-defined. Scrub typhus and lep-tospirosis are common and medically significant, while other zoonotic diseases, such as spotted fever group Rickettsiae have been identified, but their overall medical significance is unknown. Rodent surveillance was conducted from June 2002 to July 2004 in 18 provinces from Thailand. Traps were set up for one to three nights. Blood and serum samples and animal tissue samples (liver, spleen, kidney and urinary bladder) were collected. Chiggermites, ticks and fleas were removed from captured rodents. A total of 4536 wild-caught rodents from 27 species were captured over two years of animal trapping. Rattus rattus was the dominant species, followed by Rattus exulans and Bandicota indica. Almost 43 000 ectoparasites were removed from the captured animals. Approximately 98% of the ectoparasites were chigger-mites, of which 46% belonged to the genus Leptotrombidium (scrub typhus vector). Other genera included Schoengastia and Blankaartia. Tick and flea specimens together comprised less than 1% of the sample. Among the five species of ticks collected, Haemaphysalis bandicota was the predominant species caught, followed by Ixodes granulatus other Haemaphysalis spp., Rhipicephalus spp. and Dermacentor spp. Only two species of fleas were collected and Xenopsylla cheopis (rat flea) was the predominant species. Using both commercial diagnostic kits and in-house molecular assays, animal tissue samples were examined and screened for zoonotic diseases. Seven zoonotic diseases were detected: scrub typhus, leptospirosis, murine typhus, tick typhus, bartonella, babesiosis and trypanosomiasis. Most samples were positive for scrub typhus. Other zoonotic diseases still under investigation include borrelosis, ehrlichiosis, the plague, and other rickettsial diseases. Using geographic information systems, global positioning systems and remote sensing technology, epidemiological and environmental data were combined to assess the relative risk in different biotopes within highly endemic areas of scrub typhus in Thailand.

© 2008 ISZS, Blackwell Publishing and IOZ/CAS.

DOI: 10.1111/j.1749-4877.2008.00100.x
PMID: 21396076 [PubMed]

440. J Parasitol. 2015 Apr;101(2):150-5. doi: 10.1645/14-662.1. Epub 2014 Dec 30.

Rickettsial diseases and ectoparasites from military bases in Japan.

Reeves WK(1), Durden LA, Iwakami M, Vince KJ, Paul RR.

Author information:
(1)U.S. Air Force School of Aerospace Medicine/PHR, 2510 5th Street,
Wright-Patterson Air Force Base 45433.

Ectoparasitic arthropods are often vectors of rickettsiosis. We conducted a survey of ectoparasites on U.S. military facilities throughout Japan with the use of specimens submitted by pest control, public health, and veterinary personnel. Over 1,600 individual ectoparasites were collected. Fifteen species were identified, including several significant vectors of human diseases such as scrub typhus and rickettsial spotted fevers. These ectoparasites included Ctenocephalides felis , Haemaphysalis longicornis, Ixodes persulcatus , Leptotrombidium fuji, Leptotrombidium pallidum, and Rhipicephalus sanguineus . Rickettsial agents were detected by PCR and DNA sequencing. These included Bartonella henselae , Bartonella japonica, a novel Bartonella, Coxiella burnetii , an unnamed Coxiella, Ehrlichia canis , Orientia tsutsugamushi , Rickettsia typhi , and "Rickettsia Rf2125"/"Rickettsia cf1and5".

DOI: 10.1645/14-662.1
PMID: 25548900 [PubMed - indexed for MEDLINE]

441. Ned Milit Geneeskd Tijdschr. 1956 Apr;9(4):99-108.

[Cases of scrub typhus in Netherlands New Guinea].

[Article in Dutch]

HOOGERHEIDE C, ENSINK GJ.

PMID: 13322035 [PubMed - indexed for MEDLINE]

442. J Indian Med Assoc. 1954 Jun;23(9):375-8.

The occurrence of scrub typhus in Karnal District.

MATHUR TN, DATTA KN.

PMID: 13174819 [PubMed - indexed for MEDLINE]

443. J R Army Med Corps. 1954 Apr;100(2):121-6.

Scrub typhus in Hong Kong.

STEWART PD.

PMID: 13152788 [PubMed - indexed for MEDLINE]

444. Can Nurse. 1953 Oct;49(10):803-5.

Conquest of scrub typhus.

WILLIAMS T.

PMID: 13094632 [PubMed - indexed for MEDLINE]

445. J Indian Med Assoc. 1954 Jun;23(9):389-94.

Tsutsugamushi disease (scrub typhus) in Bombay City and suburbs.

SOMAN DW.

PMID: 13174824 [PubMed - indexed for MEDLINE]

446. J Clin Invest. 1954 Feb;33(2):264-75.

Studies on cortisone and antibiotics for prompt therapeutic control of typhoid fever and scrub typhus.

WISSEMAN CL Jr, PATERSON PY, SMADEL JE, DIERCKS FH, LEY HL Jr.

DOI: 10.1172/JCI102895
PMCID: PMC438499
PMID: 13130696 [PubMed - indexed for MEDLINE]

447. Kansenshogaku Zasshi. 2015 Mar;89(2):270-3.

[Three Cases of Tsutsugamushi Disease in Miyakojima Island, Okinawa, Japan].

[Article in Japanese]

Nakamura K, Shinzato T.

Tsutsugamushi disease (Scrub thyphus) has been reported from all over Japan except the Hokkaido area. In Okinawa, only one patient was reported in 2001, who was infected outside Okinawa Prefecture. The first case infected in Okinawa was reported at Miyakojima Island in 2008. We report herein on the second case

diagnosed in 2010, and the third and fourth in 2011, and all three patients were suspected to have been infected at Ikemajima Island adjacent to the island of Miyakojima. The patients recovered without any severe complications after antibiotic therapy with tetracyclines. We should take Tsutsugamushi disease into consideration in the differential diagnosis for a patient with fever, skin rash, and/or eschar even in the Okinawa area. Implementation of appropriate information and education about the disease should be carried out for local residents and tourists.

PMID: 26552125 [PubMed - indexed for MEDLINE]

448. Southeast Asian J Trop Med Public Health. 1975 Dec;6(4):477-82.

Protection against scrub typhus infection engendered by the passive transfer of immune sera.

Robinson DM, Huxsoll DL.

The passive transfer of convalescent sera did not protect the majority of mice against challenge with the homologous strain and was completely ineffective against challenge with strains unrelated by fluorescent antibody techniques. When the immune sera was incubated with the rickettsia in vitro and then inoculated into the mice a dramatic increase occurred in the number of surviving mice. The importance of these data in relation to published results with other species of rickettsia is discussed.

PMID: 818716 [PubMed - indexed for MEDLINE]

449. Nihon Kyobu Shikkan Gakkai Zasshi. 1997 Dec;35(12):1368-71.

[Sero-negative tsutsugamushi disease (scrub typhus) diagnosed by polymerase chain reaction].

[Article in Japanese]

Sugita M(1), Shigeta M, Miyake Y, Sakamoto T, Aoki S, Matsuoka R, Nagayama T, Aoki S, Matsuoka R.

Author information:
(1)Department of Traumatology and Critical Care Medicine, Showa General Hospital, Tokyo, Japan.

We report a case of sero-negative tsutsugamushi disease diagnosed by polymerase chain reaction (PCR). A 54-year-old man who worked in Nagano prefecture presented with flu-like symptoms that did not respond to cephalosporin therapy. On admission to another hospital, chest roentgenography revealed abnormal shadows; liver dysfunction was also present. Despite therapy, the patient's condition gradually worsened and he was transferred to our intensive care unit. Erythema on all extremities and scabs on the right medial femoral region and the dorsum of the left foot suggested a diagnosis of tsutsugamushi disease. We administered

minocycline and gave percutaneous cardiopulmonary support for adult respiratory distress syndrome. Despite all efforts, the patient died. Although serologic tests were not positive, Karp strains of R. tsutsugamuschi were identified on PCR amplification. Autopsy revealed evidence of acute hemorrhagic pancreatitis, which has not been reported previously in tsutsugamushi disease. We conclude that PCR techniques may be useful in confirming a diagnosis of early tsutsugamushi disease.

PMID: 9567083 [PubMed - indexed for MEDLINE]

450. QJM. 2002 Feb;95(2):126-8.

Acute respiratory distress syndrome in scrub typhus.

Tsay RW, Chang FY.

PMID: 11861962 [PubMed - indexed for MEDLINE]

451. Southeast Asian J Trop Med Public Health. 1980 Jun;11(2):232-9.

Rodent and scrub typhus survey in a ricefield at Kramat Tunngak area, Tanjung Priok, Jakarta, Indonesia.

Lim BL, Hadi TR, Sustriayu N.

Trapping of small mammals in a ricefield at Kramat Tunggak around Tanjung Priok in Jakarta city, was carried out from July 1977 through June 1978. Of three species of rodents, R.argentiventer was found to be the predominant species in the ricefield. R.r.diardii was an intermittent resident, and the presence of R.norvegicus was interesting observation. S.murinus, a house shrew, was also present. R.argentiventer was the dominant species during the periods when the rice grains were available as a source of food. Infestation with Gamasid mites and non-vector chiggers was found common in all rodent species examined. R. argentiventer was the only species found infested with the classical scrub typhus vector (L. (L.) deliense). The density and mean chigger-load of the scrub typhus vector chiggers were found to be high in stages 4 and 5 of the ricefields when the micro-habitats were favourable. The oriental rat flea (X. cheopis) was found infesting all species of rodents.

PMID: 7434074 [PubMed - indexed for MEDLINE]

452. J Formos Med Assoc. 2007 Feb;106(2 Suppl):S1-6.

Successful treatment of pulmonary hemorrhage associated with leptospirosis and scrub typhus coinfection by early plasma exchange.

Chen YS(1), Cheng SL, Wang HC, Yang PC.

Author information:
(1)Division of Pulmonary Medicine, Department of Internal Medicine, Far Eastern Memorial Hospital, Taipei, Taiwan.

Leptospirosis and scrub typhus coinfections have been reported in up to 41% of agricultural workers with acute leptospirosis in Thailand, but only sporadically in Taiwan. Because of the nonspecific clinical presentations, it is difficult to differentiate patients with coinfections from leptospirosis alone. However, failure to identify coinfection may lead to mortality if inappropriate antibiotics are used. We report a 31-year-old man coinfected with leptospirosis and scrub typhus, which manifested as diffuse alveolar hemorrhage and acute renal failure mimicking pulmonary-renal syndrome. The patient was treated by early plasma exchange and a 7-day course of moxifloxacin therapy. Both pulmonary hemorrhage and hypoxemia resolved substantially on the 4th day of hospitalization. He had a complete recovery from the disease after 6 weeks of hospitalization.

PMID: 17493889 [PubMed - indexed for MEDLINE]

453. J Assoc Physicians India. 2006 Oct;54:812-3.

ARDS complicating scrub typhus in Sub-Himalayan region.

Pandey D(1), Sharma B, Chauhan V, Mokta J, Verma BS, Thakur S.

Author information:
(1)Department of Medicine, Indira Gandhi Medical College, Shimla.

Scrub typhus is a febrile illness widely endemic in Asia caused by Rickettsiae tsutsugamushi in which humans are accidental hosts. If there is delay in the initiation of the appropriate antimicrobial therapy patient may present with serious complications. We report three cases that presented in emergency with acute respiratory distress syndrome and history of fever for more than one-week duration. On investigation all the three patients were positive for Weil Felix reaction and showed dramatic response to doxycycline.

PMID: 17214279 [PubMed - indexed for MEDLINE]

454. Zhonghua Min Guo Xiao Er Ke Yi Xue Hui Za Zhi. 1988 Jan-Feb;29(1):60-3.

[Scrub typhus--report of two cases].

[Article in Chinese]

Hsiao RL, Lin TY, Su WJ, Wu YC.

PMID: 3272984 [PubMed - indexed for MEDLINE]

455. N Z Med J. 1986 Feb 26;99(796):126-7.

Imported scrub typhus.

Henderson R, Reynolds R, Dickie A, Lang S.

PMID: 3456541 [PubMed - indexed for MEDLINE]

456. Southeast Asian J Trop Med Public Health. 1977 Dec;8(4):503-9.

Electrocardiographic changes in scrub typhus patients.

Fang CY, Dennis DT, Lee JB.

Ninety-eight cases of scrub typhus were examined electrocardiographically. Various findings beyond the normal range were as follows: In the febrile stage, sinus arrhythmia with some beats below 60 per minute, flat or low T waves in the left precordial leads, sinus tachycardia, ST segment elevation of 4-l mm in V2, prominent u waves measuring 1 mm or more in amplitude, tall and peaked T waves in V2-4, incomplete right bundle branch block, T wave inversion in V3-4, first degree A-V block, Q-Tc interval prolongation, notched T waves in V3, AV junctional escapes, prominent Ta waves or depression of PR segments in V2, and right axis deviation; in the convalescent stage, sinus arrhythmia with some beats below 60 per minute, prominent u waves measuring 1 mm or more in amplitude, tall and peaked T waves in V2-4, flat or low T waves in the left precordial leads, incomplete right bundle branch block, sinus tachycardia, first degree A-V block, Q-Tc interval prolongation, T wave inversion in V3-4, ST segment elevation of 4 mm in amplitude in V2, ventricular premature contractions, atrial premature contractions, and right axis deviation. In comparison with the electrocardiographic findings in 101 asymptomatic normal subjects, flat T waves in the precordial leads, tall and peaked T waves in V2-4 in both acute and convalescent stages, and sinus arrhythmia with some beats below 60 per minute in the convalescent stage were more frequent in cases. Electrocardiographic abnormalities were present most commonly in the acute illness, and our findings support the impression that, with few exceptions, prompt treatment of scrub typhus with antibiotics prevents the serious cardiac complications seen prior to the antibiotic era.

PMID: 149373 [PubMed - indexed for MEDLINE]

457. Indian Pediatr. 2004 Dec;41(12):1254-7.

Scrub typhus.

Pavithran S(1), Mathai E, Moses PD.

Author information:
(1)Department of Child Health and Department of Clinical Microbiology, Christian Medical College, Vellore 632 004, India.

Scrub typhus is being increasingly reported in adults in India. It should be considered a strong possibility in all undifferentiated fevers. Two children with this infection are being reported highlighting the wide variation in clinical presentation. Specific tests should be preferred over Weil Felix test wherever possible especially in areas reporting a high incidence of the infection.

PMID: 15623908 [PubMed - indexed for MEDLINE]

458. Arch Neurol. 2000 Dec;57(12):1770-2.

Scrub typhus encephalomyelitis with prominent focal neurologic signs.

Kim DE(1), Lee SH, Park KI, Chang KH, Roh JK.

Author information:
(1)Department of Neurology, Seoul National University Hospital, 28 Yongon-dong, Chongno-gu, Seoul, 110-744, South Korea.

BACKGROUND: Encephalomyelitis with prominent focal neurologic signs and associated neuroradiologic abnormalities has not been previously described in scrub typhus.
CASE DESCRIPTION: A 22-year-old woman was admitted because of fever and an altered mental state. Neurologic examination revealed bilateral sixth and seventh nerve palsies, bilateral gaze evoked nystagmus, anarthria, dysphagia, quadriparesis, and sensory level at T1. Serum and cerebrospinal fluid samples were positive for tsutsugamushi antibody. The patient's magnetic resonance images demonstrated the lesions responsible for the neurologic dysfunctions: in the lower brainstem, cerebellar peduncles, and spinal cord. It was interesting that the gray matter of the spinal cord was predominantly involved.
CONCLUSIONS: The recognition of unusual manifestations and the clinical suspicion of this treatment-responsive disease may be important, particularly in the face of increasing international and intranational travel.

PMID: 11115244 [PubMed - indexed for MEDLINE]

459. J Formos Med Assoc. 1993 May;92(5):475-7.

Acute renal failure associated with scrub typhus: report of a case.

Hsu GJ(1), Young T, Peng MY, Chang FY, Chou MY, Sheu LF.

Author information:
(1)Department of Internal Medicine, Tri-Service General Hospital, National Defense Medical Center, Taipei, Taiwan R.O.C.

We report on a 25-year-old man who had scrub typhus with the unusual complication of acute renal failure. The clinical features of fever, headache, high Weil-Felix OX-K agglutination and Rickettsia tsutsugamushi immunofluorescence titers confirmed the diagnosis of scrub typhus. Acute renal failure was proven by oliguria, urinary diagnostic indices and renal biopsy. The patient had a complete

recovery after adequate medical treatment.

PMID: 8104604 [PubMed - indexed for MEDLINE]

460. J Formos Med Assoc. 1992 Jan;91(1):110-2.

Brachial plexus neuropathy associated with scrub typhus: report of a case.

Ting KS(1), Lin JC, Chang MK.

Author information:
(1)Department of Neurology, National Defense Medical Center, Taipei, Taiwan, R.O.C.

We report on a 20-year-old man who had scrub typhus with the unusual neurologic complication of brachial plexus neuropathy. The clinical features of fever, headache, pneumonitis, eschar, high Weil-Felix OX-K agglutination and Rickettsia tsutsugamushi immunofluorescence titers confirmed the diagnosis of scrub typhus. Brachial plexus neuropathy was proven by an electrophysiologic examination. He had a nearly complete recovery after adequate medical treatment.

PMID: 1352327 [PubMed - indexed for MEDLINE]

461. Am J Trop Med Hyg. 2016 Jan;94(1):3-4. doi: 10.4269/ajtmh.15-0762. Epub 2015 Nov 10.

Changing Dynamics of Human-Rickettsial Interactions.

Walker DH(1).

Author information:
(1)Department of Pathology and Center for Biodefense and Emerging Infectious Diseases, University of Texas Medical Branch at Galveston, Galveston, Texas dwalker@utmb.edu.

Comment on
 Am J Trop Med Hyg. 2016 Jan;94(1):22-5.

DOI: 10.4269/ajtmh.15-0762
PMCID: PMC4710441
PMID: 26556832 [PubMed - indexed for MEDLINE]

462. Southeast Asian J Trop Med Public Health. 1978 Dec;9(4):489-93.

Distribution of rats infected with Rickettsia tsutsugamushi (scrub typhus) in an edge habitat.

Muul I, Chai KS.

No focalization of rats (Rattus tiomanicus and R. argentiventer) infected with Rickettsia tsutsugamushi could be discerned over a 500 m trapping transect at the border between a forest and lalang grass (Imperata cylindrica). R. tiomanicus appeared to occupy 250 m of the transect on the average and had periods during which infections were observed which averaged 97 days. Calulations indicated that more than 50% of individuals become infected over their life-time. The high rate of infection in this and other areas described in earlier publications and the habits of the rats suggest that infected mites are densely and widely dispersed in the areas studied in Malaysia.

PMID: 751214 [PubMed - indexed for MEDLINE]

463. Natl Med J India. 2009 Nov-Dec;22(6):333-4.

Emerging infections in Kerala: a case of scrub typhus.

Ittyachen AM.

PMID: 20387284 [PubMed - indexed for MEDLINE]

464. QJM. 2015 Apr;108(4):347. doi: 10.1093/qjmed/hcu209. Epub 2014 Sep 29.

The 'cigarette burn' sign.

Sharma S(1).

Author information:
(1)Department of Medicine, PGIMER, Dr RML Hospital, New Delhi, India.
drsourabh05@gmail.com.

DOI: 10.1093/qjmed/hcu209
PMID: 25267725 [PubMed - indexed for MEDLINE]

465. Vector Borne Zoonotic Dis. 2016 Feb;16(2):96-102. doi: 10.1089/vbz.2015.1831. Epub 2016 Jan 15.

Prevalence and Phylogenetic Analysis of Orientia tsutsugamushi in Small Mammals in Hanoi, Vietnam.

Hotta K(1), Pham HT(2), Hoang HT(2), Trang TC(2), Vu TN(2), Ung TT(3), Shimizu K(4), Arikawa J(4), Yamada A(1), Nguyen HT(2), Nguyen HL(2), Le MT(2), Hayasaka D(5).

Author information:
(1)1 Graduate School of Agricultural and Life Sciences, University of Tokyo , Tokyo, Japan . (2)2 National Institute of Hygiene and Epidemiology , Hanoi, Vietnam . (3)3 Vietnam Research Station, Nagasaki University , c/o National Institute of Hygiene and Epidemiology, Hanoi, Vietnam . (4)4 Graduate School of Medicine, Hokkiado University , Hokkaido, Japan . (5)5 Institute of Tropical

Medicine, Nagasaki University , Nagasaki, Japan .

Rodents are important reservoirs of many human pathogens transmitted via arthropod vectors. Arthropod-borne bacteria belonging to the family Rickettsiaceae cause acute febrile diseases in humans worldwide, but the real burdens of rickettsial diseases appear to be underestimated in Hanoi, Vietnam, because differential diagnosis on the basis of clinical signs and symptoms is confounded by the presence of other tropical infectious diseases with similar signs and symptoms. To know the prevalence of bacteria of the family Rickettsiaceae among small mammals in Hanoi, 519 animals thriving in the public places were captured and examined for the presence of bacterial sequences using duplex PCR. Nucleotide sequences specific for Orientia tsutsugamushi were detected in seven samples (1.3%). Out of seven animals, two were captured in a market, whereas five were in hospitals. None of the captured small mammals tested positive for the genus Rickettsia. The nucleotide sequence analysis of the genes encoding the 47-kDa high-temperature requirement A (47-kDa HtrA) and 56-kDa type-specific antigen (TSA) showed that these seven isolates were indistinguishable from each other. O. tsutsugamushi isolated in this study was closely related phylogenetically to the Gilliam strain, which was originally isolated at the border of Assam and Burma, rather than to those isolated in the central to southern part of Vietnam. It should be emphasized that Vietnamese hospitals were heavily infested by small rodents and some of them harbored O. tsutsugamushi. Strict hygienic control should be implemented to mitigate the potential risk posed by O. tsutsugamushi in hospital settings.

DOI: 10.1089/vbz.2015.1831
PMID: 26771283 [PubMed - in process]

466. Doc Neerl Indones Morbis Trop. 1949 Jun;1(2):134-41.

Epidemic scrub typhus in Batavia.

GISPEN R, SMIT AM, WESTERMANN CD.

PMID: 18147450 [PubMed - indexed for MEDLINE]

467. Am J Hyg. 1947 Jul;46(1):60-5.

Observations on tsutsugamushi disease (mite-borne or scrub typhus) in Northwest Honshu Island, Japan, in the fall of 1945; systematic comment on the Japanese vole-mites.

PHILIP CB.

PMID: 20252849 [PubMed - indexed for MEDLINE]

468. Medicine (Baltimore). 1946 May;25:155-214.

Scrub typhus in Assam and Burma; a clinical study of 616 cases.

SAYEN JJ, POND HS, et al.

PMID: 20986167 [PubMed - indexed for MEDLINE]

469. Am J Hyg. 1946 May;43:195-218.

Observations on tsutsugamushi disease (scrub typhus) in Assam and Burma; preliminary report.

MACKIE TT, DAVIS GE, et al.

PMID: 20985364 [PubMed - indexed for MEDLINE]

470. Mil Med. 1961 Nov;126:834-7.

Scrub typhus: occurrence in United Nations' personnel in Korea.

LEY HL, MARKELZ RA.

PMID: 14464934 [PubMed - indexed for MEDLINE]

471. J Med Entomol. 1974 Jul 15;11(3):237-303.

The ecology of chigger-borne rickettsiosis (scrub typhus).

Traub R, Wisseman CL Jr.

PMID: 4212400 [PubMed - indexed for MEDLINE]

472. Trans R Soc Trop Med Hyg. 1967;61(1):23-57.

The occurrence of scrub typhus infection in unusual habitats in West Pakistan.

Traub R, Wisseman CL Jr, Ahmad N.

PMID: 4961993 [PubMed - indexed for MEDLINE]

473. Am J Hyg. 1947 Jul;46(1):45-59.

Observations on tsutsugamushi disease (mite-borne or scrub typhus) in Northwest Honshu Island, Japan, in the fall of 1945; epidemiological and ecological data.

PHILIP CB.

PMID: 20252848 [PubMed - indexed for MEDLINE]

474. Trans R Soc Trop Med Hyg. 1947 Mar;40(4):360.

Illustrations of scrub typhus in Burma and Assam.

FULLER HS.

PMID: 20290650 [PubMed - indexed for MEDLINE]

475. Ann Intern Med. 1947 Feb;26(2):201-20.

Scrub typhus in Dutch New Guinea.

IRONS EM, ARMSTRONG HE.

PMID: 20286902 [PubMed - indexed for MEDLINE]

476. Trans R Soc Trop Med Hyg. 1946 Aug;40:15-56.

Observations on tsutsugamushi disease (scrub typhus) in Assam and Burma.

MACKIE TT.

PMID: 20998610 [PubMed - indexed for MEDLINE]

477. Public Health Rep. 1946 Jun 14;61:887-94.

A serological study of 37 cases of tsutsugamushi disease (scrub typhus) occurring in Burma and the Philippine Islands.

BENGSTON IA.

PMID: 20987857 [PubMed - indexed for MEDLINE]

478. Indian J Med Sci. 1972 Apr;26(4):211-5.

New concepts in the epidemiology of scrub typhus.

Varma RN, Mahadevan B.

PMID: 4637285 [PubMed - indexed for MEDLINE]

479. Acta Med Biol (Niigata). 1967 Dec;15:41-2.

Scrub typhus in India.

Kalra SL.

PMID: 4968949 [PubMed - indexed for MEDLINE]

480. Am J Trop Med Hyg. 1993 Oct;49(4):425-9.

A new focus of scrub typhus in tropical Australia.

Currie B(1), O'Connor L, Dwyer B.

Author information:
(1)Menzies School of Health Research, Casuarina, Darwin, Northern Territory, Australia.

A new focus of scrub typhus (Rickettsia tsutsugamushi) is described in a remote rain forest region of the Northern Territory of Australia. Five serologically confirmed cases, two near fatal with multisystem involvement, have occurred since the area became accessible to tourists. As tourism increases, other remote foci of vectors and organisms may also be recognized in tropical Australia.

PMID: 8214272 [PubMed - indexed for MEDLINE]

481. Zhonghua Liu Xing Bing Xue Za Zhi. 1983 Oct;4(5):257-9.

[The vector of scrub typhus in winter in Fujian Province].

[Article in Chinese]

Wang SQ.

PMID: 6675844 [PubMed - indexed for MEDLINE]

482. Jpn J Med Sci Biol. 1974 Feb;27(1):1-5.

Current concepts of the ecology of chigger-borne rickettsiosis (scrub typhus).

Traub R, Wisseman CL Jr.

PMID: 4600524 [PubMed - indexed for MEDLINE]

483. Trans R Soc Trop Med Hyg. 1972;66(4):582-7.

The effect of habitat on the prevalence of human scrub typhus in Malaysia.

Cadigan FC Jr, Andre RG, Bolton M, Gan E, Walker JS.

PMID: 4561007 [PubMed - indexed for MEDLINE]

484. Bull World Health Organ. 1968;39(2):219-30.

Ecological considerations in scrub typhus. 2. Vector species.

Traub R, Wisseman CL Jr.

Certain features, characteristic of outbreaks of scrub typhus, can be explained by the behaviour of the chigger vectors which are remarkably hardy and can survive weeks of freezing or immersion in water. The established vectors are all species of the genus Leptotrombidium (Leptotrombidium), i.e., L. (L.) akamushi (Brumpt, 1910), L. (L.) deliense (Walch, 1922), L. (L.) pallidum (Nagayo et al., 1919), and L. (L.) scutellare (Nagayo et al., 1921). These chiggers (i.e. larval mites) infest a broad variety of birds and mammals, and tend to be found in clusters on certain specific sites on the host. However, the precise site for any species of mite varies with the host, and it is believed that the grooming habits of the infested animal account for this "site preference". The degree of infestation cannot validly be ascribed to the size of the host. L. (L.) pavlovskyi (Schluger, 1948), L. (L.) orientale (Schluger, 1948), L. (L.) arenicola Traub, 1960, and L. (L.) tosa (Sasa & Kowashima, 1951) are regarded as probable vectors. Other species, some belonging to other genera, are under suspicion in this regard.L. (L.) subintermedium (Jameson & Toshioka, 1954) and certain other chiggers were found in "ecological islands" in all montane habitats studied in West Pakistan, despite the intervening high mountains, broad rivers and belts of semi-desert. The infection-rate of Rickettsia tsutsugamushi in vector species in nature is believed to be low, and chiggers may be serving as reservoirs of infection and not just as vectors.

PMCID: PMC2554568
PMID: 5303405 [PubMed - indexed for MEDLINE]

485. Clin Infect Dis. 2006 May 15;42(10):1505-6.

Clinical features of scrub typhus.

Basnyat B, Belbase RH, Zimmerman MD, Woods CW, Reller LB, Murdoch DR.

Comment on
Clin Infect Dis. 2006 Jan 1;42(1):e6-8.

DOI: 10.1086/503680
PMID: 16619174 [PubMed - indexed for MEDLINE]

486. Yonsei Med J. 1989;30(2):111-7.

Application of serologic diagnosis of tsutsugamushi disease (scrub typhus) in Korea where the disease was recently recognized to be endemic.

Chong Y.

In Korea, tsutsugamushi disease is a recently recognized infection. It has become clear that it is more prevalent than leptospirosis or hemorrhagic fever with renal syndrome. Accurate diagnosis of the disease is necessary for the selection of effective antimicrobial agents which can prevent fatalities and shorten the course. For the diagnosis, various serologic tests are used. Sensitivity and specificity of a test depend on various factors. In this report, microbiological aspects of the infection were briefly described and the Weil-Felix, indirect immunofluorescence and indirect immunoperoxidase tests were compared for their applicability in routine use and usefulness in the diagnosis. Their interpretations were also briefly discussed.

DOI: 10.3349/ymj.1989.30.2.111
PMID: 2678763 [PubMed - indexed for MEDLINE]

487. Trans R Soc Trop Med Hyg. 1972;66(4):588-93.

Filter paper collection of blood for use in a screening and diagnostic test for scrub typhus using the IFAT.

Gan E, Cadigan FC Jr, Walker JS.

PMID: 4627176 [PubMed - indexed for MEDLINE]

488. Mil Med. 1970 Jan;135(1):31-4.

Scrub typhus in Vietnam: experience at the 8th Field Hospital.

Hazlett DR.

PMID: 4985186 [PubMed - indexed for MEDLINE]

489. J Med Entomol. 1970 Apr;7(2):131-44.

Correlation of habitat, environment and color of chiggers, and their potential significance in the epidemiology of scrub typhus in Malaya (Prostigmata: Trombiculidae).

Nadchatram M.

PMID: 5425680 [PubMed - indexed for MEDLINE]

490. Southeast Asian J Trop Med Public Health. 1986 Dec;17(4):613-9.

A serological survey of scrub, tick, and endemic typhus in Sabah, East Malaysia.

Taylor AC, Hii J, Kelly DJ, Davis DR, Lewis GE Jr.

A seroepidemiological survey of 837 people and 383 febrile patients was performed in rural areas of Sabah. We determined that the rickettsial diseases scrub typhus and endemic typhus were uncommon causes of febrile illness, as was tick typhus, except in forest dwelling peoples. The rate of occurrence of SFGR specific antibody was 16.5% among 412 forest dwellers, indicating that tick typhus may be a frequent cause of illness in this population.

PMID: 3107139 [PubMed - indexed for MEDLINE]

491. Pediatr Infect Dis J. 1991 Mar;10(3):200-3.

Scrub and murine typhus in children with obscure fever in the tropics.

Silpapojakul K(1), Chupuppakarn S, Yuthasompob S, Varachit B, Chaipak D, Borkerd T, Silpapojakul K.

Author information:
(1)Department of Pediatrics, Haadyai Hospital, Songkla, Thailand.

Between October, 1985, and February, 1987, 28 (8.7%) cases of scrub and murine typhus were diagnosed among 320 children with greater than or equal to 1 week history of obscure fever. Scrub typhus is a rural disease and characterized by fever, tachypnea and hepatosplenomegaly. Skin rash was rare and eschar was absent. Four patients had pneumonia and two had meningitis. Murine typhus, more an urban disease, was milder and half the patients presented exclusively because of night fever. Slightly enlarged liver and skin rash were the only significant physical signs. Lacking the classical textbook presentations, both rickettsioses often were missed or diagnosed as enteric fever. Recognition is important because patients with either disease respond well to treatment with chloramphenicol or doxycycline.

PMID: 2041666 [PubMed - indexed for MEDLINE]

492. Clin Infect Dis. 1997 Dec;25(6):1473-4.

A case of scrub typhus probably acquired in Africa.

Ghorbani RP(1), Ghorbani AJ, Jain MK, Walker DH.

Author information:
(1)Department of Pathology, University of Tennessee, Memphis, USA.

PMID: 9431401 [PubMed - indexed for MEDLINE]

493. Clin Microbiol Infect. 2008 Feb;14(2):174-7. Epub 2007 Dec 7.

Risk-factors for human infection with Orientia tsutsugamushi: a case-control study in Korea.

Kim DM(1), Kim KY, Nam HS, Kweon SS, Park MY, Ryu SY.

Author information:
(1)Division of Infectious Diseases, Department of Internal Medicine, Chosun University College of Medicine, Gwangju, Korea.

A case-control study was conducted involving 156 patients with scrub typhus and 130 controls. Three factors were associated significantly with the risk of developing scrub typhus: engaging in fruit farming (OR 2.44; 95% CI 1.04-5.69), gathering chestnuts (OR 2.05; 95% CI 1.09-3.87) and taking breaks in areas adjacent to agricultural operations (OR 3.06; 95% CI 1.50-6.22). In contrast, receiving information or educational materials concerning the prevention of scrub typhus had a protective effect (OR 0.45; 95% CI 0.24-0.83). These results suggest that a health education programme will lower the risk of developing scrub typhus when applied to high-risk groups.

DOI: 10.1111/j.1469-0691.2007.01901.x
PMID: 18070125 [PubMed - indexed for MEDLINE]

494. Jpn J Med Sci Biol. 1982 Oct-Dec;35(5-6):255-9.

Serologic analysis of scrub typhus isolates from the Pescadores and Philippine Islands.

Shirai A, Coolbaugh JC, Gan E, Chan TC, Huxsoll DL, Groves MG.

PMID: 6819372 [PubMed - indexed for MEDLINE]

495. Clin Nephrol. 2003 Jul;60(1):59-61.

A case of acute renal failure, rhabdomyolysis and disseminated intravascular coagulation associated with scrub typhus.

Lee S, Kang KP, Kim W, Kang SK, Lee HB, Park SK.

PMID: 12872861 [PubMed - indexed for MEDLINE]

496. Nihon Naika Gakkai Zasshi. 1991 Nov 10;80(11):1816-7.

[A case of scrub typhus with disseminated intravascular coagulation, meningitis

and pulmonary fibrosis].

[Article in Japanese]

Kuroda T, Suzuki S, Konno M, Amano T, Mikame M.

PMID: 1783850 [PubMed - indexed for MEDLINE]

497. Q J Med. 1988 Aug;68(256):595-602.

Scrub typhus pneumonitis: an entity which is frequently missed.

Chayakul P(1), Panich V, Silpapojakul K.

Author information:
(1)Department of Medicine, Faculty of Medicine, Prince of Songkla University, Thailand.

Four cases of scrub typhus pneumonitis are reported. Diagnosis was confirmed by positive Weil-Felix OX-K reaction and immunofluorescent antibody test for Rickettsia tsutsugamushi. Two patients presented with atypical pneumonia and two had overwhelming pneumonia resembling adult respiratory distress syndrome. All patients made a full recovery after appropriate treatment.

PMID: 3076676 [PubMed - indexed for MEDLINE]

498. N Engl J Med. 1967 May 25;276(21):1195-6.

Hypofibrinogenemia in scrub typhus. Report of a case.

Chernof D.

DOI: 10.1056/NEJM196705252762109
PMID: 6023239 [PubMed - indexed for MEDLINE]

499. Diagn Microbiol Infect Dis. 1999 Oct;35(2):159-61.

An unusual site of chigger bite in a patient with scrub typhus.

Lau SM(1), Yu WL, Wang JH.

Author information:
(1)Department of Internal Medicine, China Medical College Hospital, Taichung, Taiwan, R.O.C.

A 70-year-old female farmer was admitted to the hospital because of fever, headache, and diarrhea for 7 days. Hypotension, right-sided pleural effusion with respiratory distress and leukocytosis were noted. She was initially treated as systemic bacterial infection by i.v. administration of ampicillin/sulbactam and

amikacin. Because fever persisted in spite of aggressive treatment, a repeat thorough physical examination was done. An eschar was found over the left-sided labium majus and an enlarged lymph node was noted over the left inguinal region. Under the impression of scrub typhus, minocycline was administered. The patient's clinical condition improved dramatically within 3 days. The diagnosis was later confirmed by a serologic test for Rickettsia tsutsugamushi.

PMID: 10579097 [PubMed - indexed for MEDLINE]

500. J Formos Med Assoc. 1997 Mar;96(3):213-6.

Life-threatening scrub typhus with meningoencephalitis and acute respiratory distress syndrome.

Fang CT(1), Ferng WF, Hwang JJ, Yu CJ, Chen YC, Wang MH, Chang SC, Hsieh WC.

Author information:
(1)Department of Internal Medicine, National Taiwan University Hospital, Taipei, ROC.

Erratum in
    J Formos Med Assoc 1997 May;96(5):390.

A 21-year-old man presented with fever, rash, seizure, stiff neck and rapidly progressive bilateral pulmonary infiltrates. Cerebrospinal fluid (CSF) study revealed pleocytosis with predominant polymorphonuclear cells, and hypo-glycorrhachia. Status epilepticus occurred, followed by acute respiratory distress syndrome with respiratory failure. Blood and CSF cultures for bacteria were negative, but an indirect immunofluorescence assay revealed a fourfold rise in antibody to Rickettsia tsutsugamushi in paired serum and a 1:2560 (+) IgM antibody titer. Severe scrub typhus with meningoencephalitis and extensive pneumonitis was diagnosed. The patient survived after intravenous minocycline therapy and intensive care, including aggressive seizure control, supportive mechanical ventilation and avoidance of fluid overloading. He had a nearly complete recovery. Practicing physicians in Taiwan should be aware of this reportable disease and its potentially serious complications if not promptly diagnosed and treated.

PMID: 9080762 [PubMed - indexed for MEDLINE]

501. Am J Hematol. 1991 Feb;36(2):150-1.

Atypical lymphocytes with a multilobated nucleus from a patient with tsutsugamushi disease (scrub typhus) in Japan.

Iwasaki H(1), Ueda T, Uchida M, Nakamura T, Takada N, Mahara F.

Author information:
(1)First Department of Internal Medicine, Fukui Medical School, Japan.

A case of tsutsugamushi disease (scrub typhus) with atypical lymphocytes with a multilobated nucleus is reported. Although this type of atypical lymphocyte has been reported in patients with viral infections such as adult T-cell leukemia, infectious mononucleosis, human immunodeficiency virus (HIV) infection, this is the first reported case of atypical lymphocyte with a multilobated nucleus in a patient with rickettsial infection. This type of atypical lymphocyte seems to exist in a broad spectrum of infectious diseases.

PMID: 2012066 [PubMed - indexed for MEDLINE]

502. Clin Microbiol Infect. 2015 May;21(5):404-15. doi: 10.1016/j.cmi.2015.04.022.
Epub 2015 May 8.

Neglected bacterial zoonoses.

Chikeka I(1), Dumler JS(2).

Author information:
(1)Departments of Pathology and Microbiology & Immunology, University of Maryland School of Medicine, Baltimore, MD, USA. (2)Departments of Pathology and Microbiology & Immunology, University of Maryland School of Medicine, Baltimore, MD, USA. Electronic address: sdumler@som.umaryland.edu.

Bacterial zoonoses comprise a group of diseases in humans or animals acquired by direct contact with or by oral consumption of contaminated animal materials, or via arthropod vectors. Among neglected infections, bacterial zoonoses are among the most neglected given emerging data on incidence and prevalence as causes of acute febrile illness, even in areas where recognized neglected tropical diseases occur frequently. Although many other bacterial infections could also be considered in this neglected category, five distinct infections stand out because they are globally distributed, are acute febrile diseases, have high rates of morbidity and case fatality, and are reported as commonly as malaria, typhoid or dengue virus infections in carefully designed studies in which broad-spectrum diagnoses are actively sought. This review will focus attention on leptospirosis, relapsing fever borreliosis and rickettsioses, including scrub typhus, murine typhus and spotted fever group rickettsiosis. Of greatest interest is the lack of distinguishing clinical features among these infections when in humans, which confounds diagnosis where laboratory confirmation is lacking, and in regions where clinical diagnosis is often attributed to one of several perceived more common threats. As diseases such as malaria come under improved control, the real impact of these common and under-recognized infections will become evident, as will the requirement for the strategies and allocation of resources for their control.

Copyright © 2015 European Society of Clinical Microbiology and Infectious Diseases. Published by Elsevier Ltd. All rights reserved.

DOI: 10.1016/j.cmi.2015.04.022
PMCID: PMC4466158
PMID: 25964152 [PubMed - indexed for MEDLINE]

503. US Army Med Dep J. 2009 Jul-Sep:40-9.

Perspectives on scrub typhus, tick-borne pathogens, and hantavirus in the Republic of Korea.

Sames WJ(1), Kim HC, Klein TA.

Author information:
(1)Multinational Corps-Iraq Force Health Protection.

PMID: 20088033 [PubMed - indexed for MEDLINE]

504. Intern Med. 2011;50(1):37-42. Epub 2011 Jan 1.

Delay in tetracycline treatment increases the risk of complications in Tsutsugamushi disease: data from the Japanese Diagnosis Procedure Combination database.

Yasunaga H(1), Horiguchi H, Kuwabara K, Hashimoto H, Matsuda S.

Author information:
(1)Department of Health Management and Policy, Graduate School of Medicine, The University of Tokyo, Japan. yasunagah-jyo@h.u-tokyo.ac.jp

OBJECTIVE: Tsutsugamushi disease (scrub typhus) is caused by Orientia tsutsugamushi, and has been endemic in Asia and Western Pacific islands. Though sporadic case reports have described the clinical consequences of this vector-borne disease, data on the actual incidence of complications or mortality are scarce. It also remains unclear how a delay in effective treatments affects the occurrence of complications associated with this Rickettsial disease.
METHODS: Using the Japanese Diagnosis Procedure Combination inpatient database in Japan, we identified patients with Tsutsugamushi disease between July 1 and December 31 in 2007 and 2008. We examined location of hospitals, patient's age, sex, comorbidities, complications, inhospital deaths, date of admission, date of starting therapy with tetracyclines. A logistic regression was conducted to analyze the association between delay in effective treatments and the occurrence of complications.
RESULTS: A total of 210 cases were identified. Overall, 29 (13.8%) had at least one complication and two deaths were identified. Age was a significant risk factor for complications [odds ratio (OR), 1.48; 95% confidence interval (CI), 1.08-2.03; p=0.014, for a 10-year age increase]. Patients with ≥2 days delay in treatment with tetracyclines had a significantly higher risk of complications compared to those with no delay (OR, 2.71; 95% CI, 1.03-7.12; p=0.044).
CONCLUSION: Tsutsugamushi disease remains a threat to public health. Our study clearly indicates the importance of early diagnosis and immediate tetracycline treatment to prevent severe complications in Tsutsugamushi disease.

PMID: 21212571 [PubMed - indexed for MEDLINE]

505. Indian J Med Sci. 1973 Dec;27(12):900-19.

The bionomics and vector potential of the scrub typhus vector Leptotrombidium (L.) deliense and other trombiculid populations in Eastern Himalayas, India.

Varma RN, Mahadevan B.

PMID: 4790417 [PubMed - indexed for MEDLINE]

506. Emerg Infect Dis. 2015 Jun;21(6):1073-4. doi: 10.3201/eid2106.141487.

Seroconversions to Rickettsiae in US Military Personnel in South Korea.

Jiang J, Myers TE, Rozmajzl PJ, Graf PC, Chretien JP, Gaydos JC, Richards AL.

DOI: 10.3201/eid2106.141487
PMCID: PMC4451913
PMID: 25989279 [PubMed - indexed for MEDLINE]

507. Southeast Asian J Trop Med Public Health. 2007 Jan;38(1):91-6.

Patient and sample-related factors that effect the success of in vitro isolation of Orientia tsutsugamushi.

Luksameetanasan R(1), Blacksell SD, Kalambaheti T, Wuthiekanun V, Chierakul W, Chueasuwanchai S, Apiwattanaporn A, Stenos J, Graves S, Peacock SJ, Day NP.

Author information:
(1)Faculty of Tropical Medicine, Mahidol University, Bangkok, Thailand.

Orientia tsutsugamushi is the causative agent of scrub typhus infection, a major cause of human disease in rural areas of Southeast Asia. Twenty-six blood samples collected from patients with serologically proven scrub typhus during a six month period were sent to Bangkok (535 km from the clinical site) by road at ambient temperature (average daily temperature range: 27.1-29.1 degrees C) for attempted in vitro isolation in Vero cells. O. tsutsugamushi was isolated from 12 samples (sensitivity 46.7%) with the time to isolation ranging from 16 to 37 days [median 27 days, inter-quartile range (IQR) 22.5-33.5 days]. Patient factors such as days of fever and O. tsutsugamushi IgM antibody titer, transport factors such as transit time, and isolate genotype (Karp and Gilliam/Kawasaki) were assessed to determine their influence on the outcome of in vitro isolation. None of the factors significantly influenced the isolation outcome. This study demonstrates that O. tsutsugamushi can often be isolated in vitro from the blood of scrub typhus patients when transported at ambient tropical temperatures for many days.

PMID: 17539252 [PubMed - indexed for MEDLINE]

508. J Infect Chemother. 2009 Aug;15(4):269-72. doi: 10.1007/s10156-009-0696-6. Epub 2009 Aug 18.

Surveillance, recognition, and reporting of Tsutsugamushi disease (scrub typhus) and Japanese spotted fever by general practice clinics in Miyazaki Prefecture, determined by questionnaire survey in 2007.

Matsui T(1), Kobayashi J, Satoh H, Fujimoto T, Okabe N, Ando S, Kishimoto T, Yamamoto S.

Author information:
(1)Infectious Disease Surveillance Center, National Institute of Infectious Diseases, Toyama 1-23-1, Shinjuku-ku, Tokyo, 162-8640, Japan. djyu@nih.go.jp

In June 2007, a questionnaire survey related to the surveillance, recognition, and reporting of Tsutsugamushi disease (TD) and Japanese spotted fever (JSF)--diseases considered endemic in Miyazaki Prefecture--was distributed to general practice clinics in the prefecture. The response rate was 40.9% (232/567). While 75.5% of the responding clinics knew TD to be a notifiable disease, only 41.8% knew JSF was notifiable. The recognition level of JSF surveillance was lower in the low-incidence areas of JSF within Miyazaki Prefecture. In 2006, 25 cases were clinically suspected as TD by the responding clinics; of the 25 cases, 9 were confirmed and 8 of these were reported to the National Epidemiological Surveillance of Infectious Diseases (NESID). Only 1 of 6 clinically suspected JSF cases from the responding clinics was confirmed in 2006, and it was not reported to NESID. The clinics located in the high-incidence areas for TD tended not to perform laboratory confirmation of the clinically suspected cases of either of the diseases. Considering that NESID requires laboratory confirmation of the reported cases of these diseases, their extent may be underestimated, especially in the high-incidence areas. For clinics in Miyazaki Prefecture, we need to publicize the existence of JSF surveillance and inform clinics about the laboratories available for confirmation of JSF and TD in the prefecture.

DOI: 10.1007/s10156-009-0696-6
PMID: 19688250 [PubMed - indexed for MEDLINE]

509. Emerg Infect Dis. 2009 Jul;15(7):1127-9. doi: 10.3201/eid1507.080399.

Rapid increase of scrub typhus, South Korea, 2001-2006.

Kweon SS, Choi JS, Lim HS, Kim JR, Kim KY, Ryu SY, Yoo HS, Park O.

DOI: 10.3201/eid1507.080399
PMCID: PMC2744253
PMID: 19624938 [PubMed - indexed for MEDLINE]

510. J Hyg Epidemiol Microbiol Immunol. 1968;12(2):147-61.

Monthly observation on rickettsia and complement fixing antibody response in Microtus montebelli placed once on the ground endemic of scrub typhus or inoculated experimentally with Rickettsia orientalis.

Kitaoka M, Asanuma K.

PMID: 5756349 [PubMed - indexed for MEDLINE]

511. Indian J Med Res. 2007 Aug;126(2):128-30.

Serological evidence for wide distribution of spotted fevers & typhus fever in Tamil Nadu.

Kamarasu K(1), Malathi M, Rajagopal V, Subramani K, Jagadeeshramasamy D, Mathai E.

Author information:
(1)Institute of Vector Control & Zoonoses, Hosur, India.

Comment in
Indian J Med Res. 2007 Aug;126(2):101-3.

BACKGROUND & OBJECTIVE: Although the re-emergence of spotted fevers and typhus was documented from southern India a few years ago, there was a paucity of community based data. Therefore a collaborative study was carried out in several districts of Tamil Nadu to understand the distribution of these infections.
METHODS: Blood (3 ml) was collected from patients presenting to primary health centres (PHCs) with fever >10 days duration in 15 districts of Tamil Nadu during January 2004 to December 2005. Patients negative for malaria, were tested by Weil-Felix test. Clinical data were collected from patients visiting two hospitals.
RESULTS: A total 306 samples were tested in 2004 and 115 (37.5%) had titres of >or=80 with OX K antigen, suggesting a diagnosis of scrub typhus. During 2005, 964 patients were tested and 89 (9.2%) were positive for scrub typhus. An additional 44 (4.6%) were positive for other rickettsial illnesses. In both years majority of scrub typhus occurred in individuals above 14 yr of age. Cases increased from August until the earlier part of next year.
INTERPRETATION & CONCLUSION: This community based study from south India involving several districts in Tamil Nadu, showed that scrub typhus and rickettsial illnesses were widely distributed in the State. Measures to increase awareness and also to diagnose and treat this infection in the affected areas are essential.

PMID: 17932437 [PubMed - indexed for MEDLINE]

512. Conn Med. 1976 Jun;40(6):377-8.

Imported scrub typhus.

Maher PH.

PMID: 1277833 [PubMed - indexed for MEDLINE]

513. Front Public Health. 2014 Sep 30;2:168. doi: 10.3389/fpubh.2014.00168.

eCollection 2014.

Emerging vector-borne zoonoses: eco-epidemiology and public health implications in India.

Dhiman RC(1).

Author information:
(1)National Institute of Malaria Research, Indian Council of Medical Research , New Delhi , India.

The diseases originating from animals or associated with man and animals are remerging and have resulted in considerable morbidity and mortality. The present review highlights the re-emergence of emerging mainly zoonotic diseases like chikungunya, scrub typhus, and extension of spatial distribution of cutaneous leishmaniasis from western Rajasthan to Himachal Pradesh, Kerala, and Haryana states; West Nile virus to Assam, and non-endemic areas of Japanese encephalitis (JE) like Maharashtra and JE to Delhi; Crimean-Congo hemorrhagic fever making inroads in Ahmedabad; and reporting fifth parasite of human malaria with possibility of zoonosis have been highlighted, which necessitates further studies for prevention and control. Emphasis has been given on understanding the ecology of reservoir hosts of pathogen, micro niche of vector species, climatic, socioeconomic risk factors, etc. Development of facilities for diagnosis of virus from insects, reservoirs, and human beings (like BSL4, which has been established in NIV, Pune), awareness about symptoms of new emerging viral and other zoonotic diseases, differential diagnosis, risk factors (climatic, ecological, and socioeconomic) and mapping of disease-specific vulnerable areas, and mathematical modeling for projecting epidemiological scenario is needed for preparedness of public health institutes. It is high time to understand the ecological link of zoonotic or anthroponotic diseases for updated risk maps and epidemiological knowledge for effective preventive and control measures. The public health stakeholders in India as well as in Southeast Asia should emphasize on understanding the eco-epidemiology of the discussed zoonotic diseases for taking preventive actions.

DOI: 10.3389/fpubh.2014.00168
PMCID: PMC4179687
PMID: 25325052 [PubMed]

514. Ann Trop Med Parasitol. 1978 Apr;72(2):195-6.

Correlation of scrub typhus incidence with temperature in the Pescadores Islands of Taiwan.

Olson JG, Scheer EJ.

PMID: 666390 [PubMed - indexed for MEDLINE]

515. J Parasitol. 1976 Aug;62(4):653-4.

Correlation of chigger abundance with temperature at a hyperendemic focus of

scrub typhus.

Van Peenen PF, Lien JC, Santana FJ, See R.

PMID: 957048 [PubMed - indexed for MEDLINE]

516. Trans R Soc Trop Med Hyg. 2000 May-Jun;94(3):280-4.

Antibodies to Orientia tsutsugamushi, Rickettsia typhi and spotted fever group rickettsiae among febrile patients in rural areas of Malaysia.

Tay ST(1), Ho TM, Rohani MY, Devi S.

Author information:
(1)Institute for Medical Research, Jalan Pahang, Kuala Lumpur, Malaysia.
tayst@imr.gov.my

A serosurvey was conducted in 1995-97 among 1596 febrile patients from 8 health centres in Malaysia for antibodies against Orientia tsutsugamushi (OT), Rickettsia typhi (RT) and TT118 spotted fever group rickettsiae (SFGR) by using an indirect immunoperoxidase assay. A total of 51.4% patients had antibody against at least 1 of those rickettsiae. Antibody to SFGR was most prevalent (42.5%), followed by RT (28.1%) and OT (24.9%). The seroprevalences of antibodies to SFGR, RT or OT alone were 12.4, 3.6 and 4.3%, respectively. Antibodies against more than 1 species of rickettsiae were presence in 31.1% of the patients, suggesting the possibility of co-infection, previous exposures or serological cross-reactivities. Seroprevalence of the various rickettsiae varied according to locality, with SFGR antibodies being the most prevalent in most areas. There was no significant association of prevalence of rickettsial antibody with gender. The seroprevalence of OT, SFGR and RT increased with patient age but an increase of antibody titre with age was not significant. Those working in the agricultural sectors had significantly higher seroprevalence of OT, SFGR and RT than those not related with agricultural activities. Scrub typhus remains a public health problem with an estimated annual attack rate of 18.5%. Tick typhus and murine typhus as shown in this serosurvey appear much more widespread than scrub typhus in this country.

PMID: 10974999 [PubMed - indexed for MEDLINE]

517. Clin Infect Dis. 1999 Oct;29(4):940-1.

Scrub typhus and military operations in Indochina.

Corwin A(1), Soderquist R, Suwanabun N, Sattabongkot J, Martin L, Kelly D, Beecham J.

Author information:
(1)U.S. Naval Medical Research Unit Number 2, Jakarta, Indonesia.
corwin@smtp.namru2.go.id

DOI: 10.1086/520468
PMID: 10589920 [PubMed - indexed for MEDLINE]

518. Med J Armed Forces India. 2004 Jan;60(1):89-90. doi:
10.1016/S0377-1237(04)80174-6. Epub 2011 Jul 21.

Scrub Typhus, a Case Report : Military and Regional Significance.

Singh P(1).

Author information:
(1)Officer Commanding, SHO, Jalandhar Cantt, 144 005.

DOI: 10.1016/S0377-1237(04)80174-6
PMCID: PMC4923455
PMID: 27407591 [PubMed]

519. BMJ. 2015 Aug 28;351:h4570. doi: 10.1136/bmj.h4570.

Skin lesion in a critically ill man.

Zheng MH(1), Shi KQ(2), Chen YP(2).

Author information:
(1)Department of Infection and Liver Diseases, Liver Research Centre, First
Affiliated Hospital of Wenzhou Medical University, Wenzhou 325000, China
zhengmh@wmu.edu.cn. (2)Department of Infection and Liver Diseases, Liver Research
Centre, First Affiliated Hospital of Wenzhou Medical University, Wenzhou 325000,
China.

PMID: 26318374 [PubMed - indexed for MEDLINE]

520. Clin Infect Dis. 1998 Dec;27(6):1538-9.

Azithromycin therapy for scrub typhus during pregnancy.

Choi EK(1), Pai H.

Author information:
(1)Department of Internal Medicine, University of Dankook, College of Medicine,
Cheonan, Korea.

Comment in
   Clin Infect Dis. 2000 Jan;30(1):237.

PMID: 9868680 [PubMed - indexed for MEDLINE]

521. J Trop Med Hyg. 1995 Apr;98(2):114-6.

Scrub typhus pneumonitis with delayed resolution.

Chan CH(1), Lai F, Daly B, Ho AK, Yu CM, Cheng A.

Author information:
(1)Department of Medicine, Chinese University, Hong Kong, Prince of Wales Hospital, Shatin, NT.

A 35-year-old lady was admitted to hospital with fever and dry cough. Chest radiograph showed bilateral basal infiltrate. Her Weil-Felix test was strongly positive (OX-K > 1:160) and her fever came down with intravenous tetracycline. There was no improvement in the lung shadow and spirometry showed a severe restrictive defect. Open lung biopsy confirmed the diagnosis of interstitial pneumonitis. CT of thorax 6 months after presentation showed partial resolution of the interstitial shadow.

PMID: 7714933 [PubMed - indexed for MEDLINE]

522. Int J Dermatol. 1992 Oct;31(10):693-5.

Tsutsugamushi disease (scrub typhus).

Taniguchi Y(1), Kanno Y, Ando K, Inachi S, Shimizu M.

Author information:
(1)Department of Dermatology, Mie University School of Medicine, Tsu, Japan.

Two patients with Tsutsugamushi disease (fever) were successfully treated with tetracycline derivatives after typical eschars were found, although one of the patients was initially misdiagnosed as having a drug reaction eruption. Prompt diagnosis and treatment are important because this disease can be associated with considerable morbidity and simple effective treatment is easily available.

PMID: 1399194 [PubMed - indexed for MEDLINE]

523. J Assoc Physicians India. 2009 Feb;57:154.

Atypical eschar sites in scrub typhus in sub-Himalayas.

Aggarwal P(1), Mahesh DM, Ravi Kumar V, Himral P, Kaushal SS, Verma BS.

Author information:
(1)Department of Medicine, Indira Gandhi Medical College, Shimla.

PMID: 19582984 [PubMed - indexed for MEDLINE]

524. Nihon Naika Gakkai Zasshi. 2003 Jul 10;92(7):1325-7.

[Tsutsugamushi disease (scrub typhus) in Shiga Prefecture: clinical report of two cases].

[Article in Japanese]

Shimura K(1), Kikuta T, Ueda M, Kawahara A, Tsuruyama K, Kurata H, Matsumoto K, Goto H, Okajima Y, Hino R.

Author information:
(1)Department of Internal Medicine, Shiga National Hospital, Shiga.

PMID: 12924280 [PubMed - indexed for MEDLINE]

525. Int J Hematol. 2002 Apr;75(3):337-8.

Tsutsugamushi disease (scrub typhus)-associated hemophagocytic syndrome.

Takami A, Yamauchi H, Asakura H, Ishiyama K, Nakao S.

PMID: 11999368 [PubMed - indexed for MEDLINE]

526. N Engl J Med. 1997 May 29;336(22):1613-4.

Scrub typhus after a trip to Vietnam.

Thiebaut MM, Bricaire F, Raoult D.

DOI: 10.1056/NEJM199705293362220
PMID: 9173269 [PubMed - indexed for MEDLINE]

527. Nihon Kyobu Shikkan Gakkai Zasshi. 1992 Mar;30(3):447-52.

[A case of new type scrub typhus (tsutsugamushi disease) presenting with acute respiratory failure and hemophagocytic syndrome].

[Article in Japanese]

Kobayashi T(1), Takizawa H, Hiroshima K, Uruma T, Enokihara H, Okuyama A.

Author information:
(1)Internal Medicine Clinic, Shioya Hospital, Yaita City, Japan.

A 47-year-old male was referred to our hospital with persistent high fever. He had received antibiotics (ASPC) for the diagnosis of acute infectious disease, which failed to respond, and subsequently developed marked lymphocytopenia and thrombocytopenia. During his hospitalization, hypoxic respiratory failure rapidly developed. A bone marrow aspiration revealed marked hemophagocytosis, leading to the tentative diagnosis of opportunistic respiratory infection associated with

malignant histiocytosis. Treatment for suspected malignancy and Pneumocystis carinii pneumonia was commenced. However, because of the initial lack of response to ASPC, generalized skin rashes without an ordinary stick wound which had diminished before the hospitalization, and a history of working outdoors in a nearby mountain area, the possibility of new type tsutsugamushi disease was strongly suggested. Intravenous administration of minocycline promptly improved his symptoms and laboratory data. The diagnosis was confirmed by a significant increase in the titer of antibody to Rickettsia tsutsugamushi. The thrombocytopenia was considered to have been caused by co-existent hemophagocytic syndrome.

PMID: 1569724 [PubMed - indexed for MEDLINE]

528. Nouv Presse Med. 1982 Apr 17;11(18):1419.

[Rickettsiosis caused by Rickettsia tsutsugamushi. Scrub typhus].

[Article in French]

Dunand J, Edlinger E, Ronteix JF, Dechy H, Jouin H, Dorra M, Betourne C.

PMID: 7079165 [PubMed - indexed for MEDLINE]

529. Kansenshogaku Zasshi. 1981 Sep;55(9):642-8.

[Four cases of tsutsugamushi disease (scrub typhus) complicated with disseminated intravascular coagulation (author's transl)].

[Article in Japanese]

Suzuki T, Sekikawa H.

PMID: 6798148 [PubMed - indexed for MEDLINE]

530. Am J Trop Med Hyg. 2003 Nov;69(5):519-24.

Occurrence of Orientia tsutsugamushi in small mammals from Thailand.

Coleman RE(1), Monkanna T, Linthicum KJ, Strickman DA, Frances SP, Tanskul P, Kollars TM Jr, Inlao I, Watcharapichat P, Khlaimanee N, Phulsuksombati D, Sangjun N, Lerdthusnee K.

Author information:
(1)United States Army Component, Armed Forces Research Institute of Medical Sciences, Bangkok, Thailand.

Extensive sampling of small mammals was conducted in eight provinces of Thailand between September 9, 1992 and April 29, 2001. A total of 3,498 specimens

representing 22 species were collected. Eighty-eight percent (3,089 of 3,498) of the animals were collected from a region in Chiangrai Province, which is commonly recognized as endemic for human scrub typhus. Blood and tissue samples from each animal were tested for the presence of Orientia tsutsugamushi, the etiologic agent of scrub typhus. The predominant species collected were Rattus rattus (53%, n = 1,863), R. losea (18%, n = 638), Bandicota indica (16%, n = 564), and R. exulans (4%, n = 146). Orientia tsutsugamushi was detected in 10 of the 22 species of mammals that included R. bukit (25% infected, 1 of 4), R. rattus (23%, 419 of 1,855), R. argentiventer (22%, 5 of 23), R. berdmorei (22%, 2 of 9), R. losea (13%, 82 of 638), B. indica (9%, 52 of 564), R. koratensis (8%, 1 of 12), B. savilei (3%, 1 of 30), R. exulans (1%, 2 of 146), and Tupaia glis (2%, 1 of 49). Infected animals were found in Chiangrai (18% infected, 563 of 3,084), Bangkok (11%, 1 of 9), Sukothai (3%, 1 of 30), and Nonthaburi (1%, 1 of 69) Provinces. The implications towards scrub typhus maintenance and transmission are discussed.

PMID: 14695089 [PubMed - indexed for MEDLINE]

531. Am J Trop Med Hyg. 2002 Nov;67(5):497-503.

Comparative evaluation of selected diagnostic assays for the detection of IgG and IgM antibody to Orientia tsutsugamushi in Thailand.

Coleman RE(1), Sangkasuwan V, Suwanabun N, Eamsila C, Mungviriya S, Devine P, Richards AL, Rowland D, Ching WM, Sattabongkot J, Lerdthusnee K.

Author information:
(1)Department of Entomology, U.S. Army Medical Component, Armed Forces Research Institute of Medical Sciences, Bangkok, Thailand.
Russell.coleman@det.amedd.army.mil

We compared the performance of 2 commercially available dipstick assays, 2 enzyme-linked immunosorbent assays (ELISAs), and an indirect immunofluorescent antibody (IFA) assay for the diagnosis of scrub typhus, using the indirect immunoperoxidase (IIP) test as the reference standard. The dipstick assays were the Integrated Diagnostics (Baltimore, MD) Dip-S-Ticks Scrub Recombinant (r56) dipstick test (INDX assay) and the PanBio (Brisbane, Australia) Scrub Typhus IgM and IgG Rapid Immunochromatographic test (PanBio assay). One of the ELISAs used pooled cell lysates of Karp, Kato, and Gilliam strain Orientia tsutsugamushi as antigen (pooled-antigen ELISA), and the other used a recombinant r56 protein as the antigen (recombinant ELISA). With a panel of 123 positive and 227 negative sera, sensitivity and specificity of the assays were as follows: INDX assay, IgG, 60% and 95%, IgM, 60% and 97%; PanBio assay, IgG, 94% and 96%, IgM, 83% and 93%; IFA (1:400 cutoff), IgG, 91% and 96%, IgM, 85% and 98%; pooled-antigen ELISA, IgG (1:1600 cutoff), 97% and 89%, IgM (1:400 cutoff), 94% and 91%; recombinant ELISA, IgG (1:1600 cutoff), 97% and 92%, IgM (1:400 cutoff), 93% and 94%. Because of its excellent performance and use of a standardized, commercially available antigen, the recombinant ELISA is suitable for use in a diagnostic laboratory, where it may be able to replace the IFA and IIP assays. In contrast, the PanBio dipstick assay was easy to perform and did not require sophisticated equipment, making it suitable for use in rural areas where more sophisticated diagnostic tests such as the ELISA and IFA may not be available.

PMID: 12479551 [PubMed - indexed for MEDLINE]

532. Southeast Asian J Trop Med Public Health. 2003 Mar;34(1):165-70.

Antibody prevalence of Orientia tsutsugamushi, Rickettsia typhi and TT118 spotted fever group rickettsiae among Malaysian blood donors and febrile patients in the urban areas.

Tay ST(1), Kamalanathan M, Rohani MY.

Author information:
(1)Department of Medical Microbiology, Faculty of Medicine, University of Malaya, Malaysia. tayst@ummc.edu.my

The seroprevalence of Orientia tsutsugamushi (OT), Rickettsia typhi (RT) and TT118 spotted fever group rickettsiae (SFGR) among blood donors and febrile Malaysian patients in the urban areas was determined. Of the 240 blood donors, 5.4%, 9.2% and 1.7% had either present or previous exposure to OT, RT and SFG rickettsiae, respectively. Patients admitted to an urban hospital had high seroprevalences of OT (43.5%) and RT (22.9%), as compared to SFGR (11.6%). Antibody levels suggestive of recent infections of scrub typhus, murine typhus and tick typhus were detected in 16.8%, 12.7% and 8.2% of patients respectively. No significant difference was noted in the distribution of rickettsial antibodies among urban patients from 2 geographical locations. However, the serologic patterns of rickettsial infection in the urban areas were different form those of rural areas.

PMID: 12971530 [PubMed - indexed for MEDLINE]

533. Am J Trop Med Hyg. 2016 Aug 3;95(2):462-5. doi: 10.4269/ajtmh.15-0775. Epub 2016 Jun 6.

Evidence of Rickettsia and Orientia Infections Among Abattoir Workers in Djibouti.

Horton KC(1), Jiang J(2), Maina A(2), Dueger E(3), Zayed A(4), Ahmed AA(5), Pimentel G(6), Richards AL(2).

Author information:
(1)Global Disease Detection Regional Center, U.S. Naval Medical Research Unit No. 3, Cairo, Egypt. katherinehorton12@gmail.com. (2)Viral and Rickettsial Diseases Department, Naval Medical Research Center, Silver Spring, Maryland. (3)Global Disease Detection Regional Center, U.S. Naval Medical Research Unit No. 3, Cairo, Egypt. Global Disease Detection Branch, Division of Global Health Protection, Center for Global Health, U.S. Centers for Disease Control and Prevention, Atlanta, Georgia. (4)Vector Biology Research Program, U.S. Naval Medical Research Unit No. 3, Cairo, Egypt. (5)Department of Epidemiology and Health Information, Ministry of Health, Djibouti, Djibouti. (6)Biological Defense Research Directorate, Naval Medical Research Center, Silver Spring, Maryland.

Of 49 workers at a Djiboutian abattoir, eight (16%, 95% confidence interval [CI]: 9-29) were seropositive against spotted fever group rickettsiae (SFGR), two (4%, 95% CI: 1-14) against typhus group rickettsiae, and three (6%, 95% CI: 2-17) against orientiae. One worker (9%, 95% CI: 2-38) seroconverted against orientiae during the study period. This is the first evidence of orientiae exposure in the Horn of Africa. SFGR were also identified by polymerase chain reaction in 32 of 189 (11%, 95% CI: 8-15) tick pools from 26 of 72 (36%) cattle. Twenty-five (8%, 95% CI: 6-12) tick pools were positive for Rickettsia africae, the causative agent of African tick-bite fever. Health-care providers in Djibouti should be aware of the possibility of rickettsiae infections among patients, although further research is needed to determine the impact of these infections in the country.

© The American Society of Tropical Medicine and Hygiene.

DOI: 10.4269/ajtmh.15-0775
PMCID: PMC4973201 [Available on 2017-08-03]
PMID: 27273647 [PubMed - in process]

534. Clin Infect Dis. 2015 Jun 15;60(12):1828, 1864-5. doi: 10.1093/cid/civ108.

A cutaneous clue to a systemic illness.

Nampoothiri RV(1), Lakshman A(1), Kesav P(2).

Author information:
(1)Department of Internal Medicine. (2)Department of Neurology, Post Graduate Institute of Medical Education and Research, Chandigarh, India.

DOI: 10.1093/cid/civ108
PMID: 26009551 [PubMed - indexed for MEDLINE]

535. Diagn Microbiol Infect Dis. 2006 Jul;55(3):185-90. Epub 2006 Apr 19.

Determination and geographic distribution of Orientia tsutsugamushi serotypes in Thailand by nested polymerase chain reaction.

Manosroi J(1), Chutipongvivate S, Auwanit W, Manosroi A.

Author information:
(1)Pharmaceutical Cosmetic Raw Materials and Natural Products Research and Development Center (PCRNC), Institute for Science and Technology Research and Development/Faculty of Pharmacy, Chiang Mai University, Chiang Mai 50200, Thailand.

Clotted blood samples of 240 scrub typhus patients were collected from 8 Regional Medical Sciences Centers in Thailand during 1999 to 2002. The serotypes of Orientia tsutsugamushi and their geographic distribution were determined. A nested polymerase chain reaction (PCR) was used to identify the serotypes of O. tsutsugamushi. The number of patients with positive results for O. tsutsugamushi was 25.0%. Two serotypes, Karp and Kato, were detected in these samples. No

Gilliam serotype was detected from any of the study locations. The PCR products were sequenced using an automated DNA sequencer. The nucleotide sequence of gene encoding 56-kDa protein from these samples showed a high sequence homology with the reference sequence of O. tsutsugamushi Karp and Kato serotypes. O. tsutsugamushi Karp serotype was predominant throughout Thailand with the percentage of 96.8% of the total serotype-positive patients, whereas 3.2% for Kato serotype was observed only in the south. The highest number among the region of Karp serotype-positive patients of 31.6% was found in the northeast.

DOI: 10.1016/j.diagmicrobio.2006.01.014
PMID: 16626907 [PubMed - indexed for MEDLINE]

536. Am J Trop Med Hyg. 1998 Apr;58(4):513-8.

Molecular and serologic survey of Orientia tsutsugamushi infection among field rodents in southern Cholla Province, Korea.

Song HJ(1), Seong SY, Huh MS, Park SG, Jang WJ, Kee SH, Kim KH, Kim SC, Choi MS, Kim IS, Chang WH.

Author information:
(1)Microbiology Division, Health and Environment Institute of Chollanam-do, Kwangiu, Korea.

Field rodents were collected from six areas in southern Cholla Province, Korea from October to December 1993. Twenty-eight (24%) of the 119 Apodemus agrarius were seropositive (> 1:10) for Orientia tsutsugamushi by the passive hemagglutination assay (PHA). Of the seropositive cases, 11 specimens had antibody titers greater than 1:80. No seropositive specimens were found among the eight Crocidura lasiura collected. On the other hand, the polymerase chain reaction (PCR) amplified about 520 basepairs of a gene encoding the 56-kD protein from the genomic DNA of 12 strains of O. tsutsugamushi tested. This target DNA sequence was amplified from the 11 (8.7%) blood specimens of A. agrarius, and one of the eight C. lasiura also showed evidence of O. tsutsugamushi infection by PCR. Only one of the PCR-positive samples was also PHA-positive. These results suggest that the PCR combined with a serologic assay more accurately detects the degree of infection of rodents with rickettsiae-causing scrub typhus in epidemiologic surveys.

PMID: 9574801 [PubMed - indexed for MEDLINE]

537. Microbiol Immunol. 2005;49(6):545-9.

Rapid and simple identification of Orientia tsutsugamushi from other group rickettsiae by duplex PCR assay using groEL gene.

Park HS(1), Lee JH, Jeong EJ, Kim JE, Hong SJ, Park TK, Kim TY, Jang WJ, Park KH, Kim BJ, Kook YH, Lee SH.

Author information:
(1)Department of Microbiology, Konkuk University, Chungju, Chungchongbuk-Do,

Korea.

In this study, two new duplex PCR methods based on the groEL gene were developed and investigated for the diagnosis of rickettsiae. The first duplex PCR assay amplified the 229-bp and the 366-bp DNAs of 6 strains including typhus group (TG) and spotted fever group (SFG) rickettsiae, and 5 scrub typhus group (STG) rickettsiae, respectively. The second duplex PCR assay amplified the 397-bp and the 213-bp DNAs of 6 Rickettsia strains and 5 STG strains. These duplex PCR methods could simultaneously perform the rapid identification of rickettsiae and the differential diagnosis of STG and other group rickettsiae in a single reaction.

PMID: 15965302 [PubMed - indexed for MEDLINE]

538. Am J Trop Med Hyg. 2003 Apr;68(4):477-9.

Prevalence of relative bradycardia in Orientia tsutsugamushi infection.

Aronoff DM(1), Watt G.

Author information:
(1)Division of Infectious Diseases, Department of Medicine, University of Michigan Health System, Ann Arbor, Michigan 48109-0642, USA. daronoff@umich.edu

We investigated 100 febrile patients infected with Orientia tsutsugamushi (the etiologic agent of scrub typhus) for the presence of relative bradycardia, defined as in increase in heart rate of < 10 beats/minutes/1 degree C increase in temperature. The median heart rate response for the entire febrile scrub typhus population was 9.3 beats/minute/degrees C and the prevalence of relative bradycardia was 53%. The occurrence of relative bradycardia was independent of patient age or gender. There were no differences in median basal temperature or febrile temperature between those patients exhibiting relative bradycardia and those with a normal febrile pulse increase. However, febrile patients with relative bradycardia had a significantly higher resting pulse rate following recovery from infection than did patients who had a normal pulse increase during their illness. These data demonstrate that relative bradycardia frequently accompanies mild infection with O. tsutsugamushi and that baseline cardiovascular parameters may affect the febrile heart rate response to scrub typhus.

PMID: 12875300 [PubMed - indexed for MEDLINE]

539. Clin Microbiol Infect. 2008 Feb;14(2):168-73. Epub 2007 Dec 10.

Detection of new genotypes of Orientia tsutsugamushi infecting humans in Thailand.

Fournier PE(1), Siritantikorn S, Rolain JM, Suputtamongkol Y, Hoontrakul S, Charoenwat S, Losuwanaluk K, Parola P, Raoult D.

Author information:

(1)Unité des Rickettsies, IFR 48, CNRS UMR6020, Faculté de Médecine, Université de la Méditerranée, Marseille, France.

PCR screening of blood specimens taken from 195 patients with serologically confirmed scrub typhus in three Thai provinces detected the 56-kDa protein-encoding gene from Orientia tsutsugamushi in ten (5%) patients. Significant genetic diversity was found among the ten amplicons, with nine new genotypes identified that were different from those found previously in Thailand. Phylogenetically, the ten sequences obtained in the present study and sequences from 71 strains characterised previously were distributed into several clusters that included the Karp, Gilliam, Kuroki, Saitama, Kawasaki and Kato clusters. Two of the new genotypes found in the present study clearly belonged to the Karp cluster. However, the other new genotypes formed three different clusters, including one cluster that appeared to be distant from all previously known clusters, and which may therefore be representative of a previously undescribed serotype. Other genotypes formed two other clusters that may also be associated with undescribed serotypes.

DOI: 10.1111/j.1469-0691.2007.01889.x
PMID: 18076670 [PubMed - indexed for MEDLINE]

540. Southeast Asian J Trop Med Public Health. 2004 Jun;35(2):353-7.

Characterization of Orientia tsutsugamushi strains isolated in Shandong Province, China by immunofluorescence and restriction fragment length polymorphism (RFLP) analyses.

Liu YX(1), Zhao ZT, Gao Y, Jia CQ, Zhang JL, Yang ZQ, Wang SM, Jiang BF.

Author information:
(1)School of Public Health, Shandong University, Jinan, PR China.

In order to identify the characteristics of the Sta56 gene of the 23 isolates of Orientia (O.) tsutsugamushi isolated in Shandong Province, indirect immunofluorescence assay (IFA) was used to identify the gene type of 23 strains O. tsutsugamushi isolated from scrub typhus patients, chigger mites, and rodents. Restriction fragment length polymorphism (RFLP) analysis was also used to analyze the restriction profiles of the Sta56 gene PCR amplification products of the 23 isolated strains of the O. tsutsugamushi; the results were compared with those acquired by nested PCR. By IFA, 21 of the 23 isolates belonged to the Gilliam type, and 2 to the Karp type. Using RFLP analysis, 21 strains had similar restriction profiles to the Japan Kawasaki strain, but they had no restriction site Hha I, and thus had some difference in gene sequence compared with the Japan Kawasaki strain. The other 2 strains had similar restriction profiles to Karp. These results were identical to that acquired by nested-PCR. In Shandong Province, the gene types of epidemic O. tsutsugamushi strains were similar to the Japan Kawasaki type, but had some differences in gene sequence. In addition, Karp also existed.

PMID: 15691135 [PubMed - indexed for MEDLINE]

541. Southeast Asian J Trop Med Public Health. 2002 Sep;33(3):557-64.

Antigenic types of Orientia tsutsugamushi in Malaysia.

Tay ST(1), Rohani MY, Ho TM, Devi S.

Author information:
(1)Department of Medical Microbiology, Faculty of Medicine, University of Malaya, Malaysia. tayst@ummc.edu.my

The seroprevalence of various Orientia tsutsugamushi (OT) strains among Malaysian patients with suspected scrub typhus infections was determined using an indirect immunoperoxidase (IIP) assay. IgG against a single OT strain were detected in six sera (3 Karp, 1 Gilliam and 2 TC586), whereas IgM antibodies against a single OT strain (Gilliam) were noted in 3 sera (Gilliam). IgG reactive to all OT strains were present in 33 (47.1%) of the 70 sera and IgM reactive to all OT strains were present in 22 (78.6%) of the 28 sera. The fact that most sera were reactive to multiple OT strains suggests that group-specific antigens are involved in scrub typhus infections, whereas very few were due to strain-specific epitopes present on these strains. Peak IgG and IgM titers were noted more frequently against Gilliam, Karp, and TA763 strains: this suggests that these strains may be the commonest infecting strains among Malaysian patients. Two predominant OT polypeptides consistently reacted with patients' sera were the 70 kDa and 56 kDa proteins.

PMID: 12693591 [PubMed - indexed for MEDLINE]

542. Infection. 1999;27(4-5):291-2.

Scrub and murine typhus among Dutch travellers.

Groen J, Nur YA, Dolmans W, Ligthelm RJ, Osterhaus AD.

PMID: 10885849 [PubMed - indexed for MEDLINE]

543. Indian J Dermatol Venereol Leprol. 2003 Nov-Dec;69(6):413-5.

Scrub typhus: a case report.

Rajagopal R(1), Khati C, Vasdev V, Trehan A.

Author information:
(1)Department of Dermatology, 5 Air Force Hospital, C/o 99 APO.
ravi_rajagopal@rediffmail.com

Fever with rash is a common cause for dermatological referral. The causes can range from viral to protozoal, bacterial or spirochaetal. A case of rickettsial fever is reported.

PMID: 17642955 [PubMed]

544. Microbiol Immunol. 1996;40(10):743-7.

Virulence in mice of Orientia tsutsugamushi isolated from patients in a new endemic area in Japan.

Nagano I(1), Kasuya S, Noda N, Yamashita T.

Author information:
(1)Department of Parasitology, Gifu University School of Medicine, Japan.

Four strains of Orientia tsutsugamushi (KN-1, KN-2, KN-3 and GJ-1) isolated from patients in an area of Gifu Prefecture, Japan, in which tsutsugamushi disease is newly endemic, were examined for their virulence in mice. Among these, KN-1 (identified as Kawasaki type), GJ-1 (identified as Kuroki type) and KN-2 strains were found to be non-lethal for BALB/c mice as well as CH3/HeJ mice, even with high doses (10(6) x being the 50% mouse infectious dose). On the other hand, the KN-3 strain was found to be sufficiently virulent to kill BALB/c mice. Among the prototype strains (Gilliam, Karp and Kato), the Karp and Kato strains exhibited high virulence to mice, while the Gilliam strain killed only a susceptible strain of mouse. BALB/c mice infected with KN-1 and KN-2 strains showed significant splenomegaly and moderate ascites accumulation in the first week of infection, while these symptoms became prominent during the second week of infection using KN-3, Karp and Kato strains. After infection with the GJ-1 strain, these symptoms were not observed. Antibody responses induced by infections with highly virulent strains were lower than that with low or intermediate virulent strains.

PMID: 8981347 [PubMed - indexed for MEDLINE]

545. Am J Trop Med Hyg. 1996 Nov;55(5):556-9.

Antibodies to Orientia tsutsugamushi in Thai soldiers.

Eamsila C(1), Singsawat P, Duangvaraporn A, Strickman D.

Author information:
(1)Department of Epidemiology, Armed Forces Research Institute of Medical Sciences, Bangkok, Thailand.

Thai soldiers who were conscripted, Royal Thai Army forces, professional Border Patrol Police, or local militia (Thai Rangers) located in any of seven provinces of Thailand were bled in April and again, four months later, in July 1989. In 1991, soldiers from five different locations in southern Thailand were bled once, in July. Serum samples were tested by indirect fluorescent antibody assay for antibody to Orientia (formerly Rickettsia) tsutsugamushi, etiologic agent of scrub typhus, with any titer > or = 1:50 considered positive. Prior to field exercises, prevalence of antibody varied significantly between different types of units, ranging between 18.6% for Thai Rangers and 6.8% for the Royal Thai Army. The April prevalence, July prevalence, and incidence varied significantly by province in 1989, with highest incidence being 14.5% in Kanchanaburi and the

lowest 0% in Utraladit. The prevalence in southern Thailand in 1991 varied between 1.6% and 6.8%. The data demonstrate that O. tsutsugamushi is widely distributed in Thailand and that military activity consisting of field exercises that simulate combat conditions significantly expose soldiers to infection.

PMID: 8940989 [PubMed - indexed for MEDLINE]

546. J Med Assoc Thai. 2009 Feb;92 Suppl 1:S39-46.

Incidence of rickettsial infection in patients with acute fever in provincial Thai army hospitals.

Thitivichianlert S(1), Panichkul S, Bodhidatta D, Rodkvamtook W, Sukwit S, Boonmee P, Ketupanya A.

Author information:
(1)Department of Medicine, Phramongkutklao Hospital, Bangkok, Thailand.

Scrub typhus is common among patients with acute fever in rural areas of Thailand. The authors prospectively recruited patients with acute fever from provincial Thai army hospitals. Dot-ELISA test for scrub typhus was done in hospitals and then compared with standard immunofluorescent assay for diagnosis of scrub typhus. Among 178 patients, scrub typhus was diagnosed by immunofluorescent assay in 10 patients (5.61%). The incidence was high in the northeastern and northern regions. Dot-ELISA gave positive results in 4 of 115 patients, while immunofluorescent assay gave positive results in 6 patients (sensitivity = 66.7%). No false positive results of Dot-ELISA were found among 109 patients (specificity = 100%). All patients gave negative results for murine typhus and Thai tick typhus using immunofluorescent assay. Regarding this present study, Dot-ELISA for scrub typhus has a good sensitivity and specificity and can be used in rural hospitals. This test could be useful for diagnosis of scrub typhus in hospitals where immunofluorescent assay is not available.

PMID: 21302413 [PubMed - indexed for MEDLINE]

547. Taehan Kan Hakhoe Chi. 2003 Sep;9(3):198-204.

[Outbreak of hepatitis by Orientia tsutsugamushi in the early years of the new millenium].

[Article in Korean]

Park JI(1), Han SH, Cho SC, Jo YH, Hong SM, Lee HH, Yun HR, Yang SY, Yoon JH, Yun YS, Moon JY, Cho KR, Baik SH, Son JH, Kim TW, Lee DH.

Author information:
(1)Department of Internal Medicine, College of Medicine, Hanyang University Institute of Digestive Diseases, Seoul, Korea.

BACKGROUND/AIMS: Orientia -tsutsugamushi infection is an acute febrile disease

due to the accidental transmission through human skin of forest dwelling vector Leptotrombidium larva. The authors observed liver dysfunctions in patients diagnosed with tsutsugamushi disease (Scrub typhus) in the past 3 years and report the data in the hope of bringing attention to this disease in the differential diagnosis of autumn-season hepatitis, especially of non-A, non-B and non-C hepatitis.
METHODS: Medical records of 22 patients diagnosed with tsutsugamushi disease by the hemagglutinin method between October 2000 and November 2002 were reviewed.
RESULTS: Female gender was dominant in the ratio of 3.4:1. Mean age was 56.4 +/- 2.6. Admission was between 23rd September and 15th November with the peak between mid October and early November. Fever, being the most common symptom, was observed in 21 cases, myalgia in 13, arthralgia in 12, chills in 6, and skin rash in 6. An incubation period of 7-9 days was most common (10 cases), 13-15 days (4), 10-12 days (3), within 3 days (3), and 4-6 days (2). Average ALT, AST and GGTP were increased to 93.2 +/- 17.3 IU/L (18 +/- 345 IU/L), 92.5 +/- 11.7 IU/L (34-255 IU/L) and 132.2 +/- 14.5 IU/L (19-251 IU/L), respectively, but total bilirubin was normal. All the patients improved with doxycycline therapy.
CONCLUSIONS: Since it usually shows liver dysfunction, it is important to take Orientia tsutsugamushi into consideration in differential diagnosis of autumn-season, febrile hepatic disease.

PMID: 14515037 [PubMed - indexed for MEDLINE]

548. Southeast Asian J Trop Med Public Health. 1997 Sep;28(3):666-8.

Antibodies to Orientia tsutsugamushi in soldiers in northeastern Thailand.

Frances SP(1), Eamsila C, Strickman D.

Author information:
(1)Department of Entomology, US Army Medical Component, Bangkok, Thailand.

The prevalence and incidence of antibodies to Orientia tsutsugamushi, the etiologic agent of scrub typhus, in Thai soldiers living and working near the Thai-Cambodian border in Si Sa Ket Province was investigated. The point prevalence of antibodies varied from 0 to 4.1%. The incidence of antibodies, calculated from individuals who seroconverted following a negative result in a previous bleeding 3 to 5 months earlier, was 4.21% (9/214) in January 1992, 0 in April 1992 and 3.76% (8/213) in September 1992. An annual infection rate of 2.66% was estimated.

PMID: 9561627 [PubMed - indexed for MEDLINE]

549. Microbiol Immunol. 1999;43(10):975-8.

Decreased prevalence of Orientia tsutsugamushi in trombiculid mites and wild rodents in the Primorye region, Far East Russia.

Urakami H(1), Tamura A, Tarasevich IV, Kadosaka T, Shubin FN.

Author information:
(1)Department of Microbiology, Niigata College of Pharmacy, Japan.
urakami@niigata-pharm.ac.jp

The isolation of Orientia tsutsugamushi was attempted from 249 rodents and approximately 14,000 trombiculid mites captured in the Primorye region, Far East Russia in 1993 and 1994, where high infection rates were recorded in both rodents and mites in the 1960s. However, no rickettsia was isolated from the samples. Low antibody titers against O. tsutsugamushi were detected in 7.1% of the rodents. These results indicate that the prevalence of O. tsutsugamushi in the Primorye region has decreased considerably in the past 30 years.

PMID: 10585144 [PubMed - indexed for MEDLINE]

550. Ann N Y Acad Sci. 2009 May;1166:172-9. doi: 10.1111/j.1749-6632.2009.04514.x.

Epidemiology and clinical aspects of rickettsioses in Thailand.

Suputtamongkol Y(1), Suttinont C, Niwatayakul K, Hoontrakul S, Limpaiboon R, Chierakul W, Losuwanaluk K, Saisongkork W.

Author information:
(1)Department of Medicine, Faculty of Medicine Siriraj Hospital, Mahidol University, Bangkok. siysp@mahidol.ac.th

Scrub typhus and murine typhus are widespread in Thailand. Clinical manifestations of both diseases are nonspecific and vary widely. Acute undifferentiated fever (AUF), with or without organ dysfunction, is a major clinical presentation of these two diseases. The epidemiology and clinical manifestations including severe complications of scrub typhus and murine typhus in Thailand are summarized. Sixteen hundred and sixty-three patients with AUF were studied in six hospitals in Thailand between 2000 and 2003. Scrub typhus and murine typhus were diagnosed in 16.1% and 1.7% of them, respectively. Clinical spectrum of murine typhus was similar to scrub typhus. Hepatic dysfunction and pulmonary involvement were common complications. Multi-organ dysfunction mimicking sepsis syndrome occurred in 11.9% of patients with scrub typhus. The mortality of severe scrub typhus varied from 2.6% to 16.7%. Awareness that scrub typhus and murine typhus are prominent causes of AUF in adults in Thailand improves the probability of an accurate clinical diagnosis. Early recognition and appropriate treatment reduces morbidity and mortality. Results from recent clinical studies from Thailand indicated that rational antimicrobial therapy would be doxycycline in mild cases and a combination of either cefotaxime or ceftriaxone and doxycycline in severe cases. Azithromycin could be considered as an alternative treatment when doxycycline allergy is suspected. This would be either curative, or have no ill effect, in the majority of instances. Failure to improve or defervesce within 48 hours would indicate the need to perform a thorough re-evaluation of clinical findings and initial laboratory investigation results, as well as a need to change antibiotic.

DOI: 10.1111/j.1749-6632.2009.04514.x
PMID: 19538278 [PubMed - indexed for MEDLINE]

551. Am J Trop Med Hyg. 1946 Mar;26:229-42.

Tsutsugamushi disease (scrub or mite-borne typhus) in the Philippine Islands during American re-occupation in 1944-45.

PHILIP CB, WOODWARD TE, SULLIVAN RR.

PMID: 21020342 [PubMed - indexed for MEDLINE]

552. BMJ Case Rep. 2013 Jun 27;2013. pii: bcr2013010105. doi: 10.1136/bcr-2013-010105.

Eschar: an important clue to diagnosis.

Sundriyal D(1), Kumar N, Chandrasekharan A, Sharma B.

Author information:
(1)Department of Internal Medicine, PGIMER & Dr Ram Manohar Lohia Hospital, New Delhi, India.

DOI: 10.1136/bcr-2013-010105
PMCID: PMC3702972
PMID: 23814208 [PubMed - indexed for MEDLINE]

553. Epidemiol Infect. 2015 Jul;143(10):2081-93. doi: 10.1017/S0950268814003707. Epub 2015 Jan 13.

Concomitant leptospirosis-hantavirus co-infection in acute patients hospitalized in Sri Lanka: implications for a potentially worldwide underestimated problem.

Sunil-Chandra NP(1), Clement J(2), Maes P(2), DE Silva HJ(1), VAN Esbroeck M(3), VAN Ranst M(2).

Author information:
(1)Department of Medical Microbiology,Faculty of Medicine,University of Kelaniya,Ragama,Sri Lanka. (2)National Reference Centre for Hantaviruses, Clinical and Epidemiological Virology, University of Leuven,Leuven,Belgium. (3)National Reference Laboratory for Leptospira,Department of Clinical Sciences,Institute of Tropical Medicine,Antwerp,Belgium.

Erratum in
Epidemiol Infect. 2015 Jul;143(10):2094.

Two global (re-)emerging zoonoses, leptospirosis and hantavirus infections, are clinically indistinguishable. Thirty-one patients, hospitalized in Sri Lanka for acute severe leptospirosis, were after exclusion of other potentially involved pathogens, prospectively screened with IgM ELISA for both pathogens. Of these, nine (29·0%) were positive for leptospirosis only, one (3·2%) for hantavirus only, seven (22·5%) for both pathogens concomitantly, whereas 13 (41·9%) remained negative for both. Moreover, in a retrospective study of 23 former patients, serologically confirmed for past leptospirosis, six (26·0%) were also positive in

two different IgG ELISA hantavirus formats. Surprisingly, European Puumala
hantavirus (PUUV) results were constantly higher, although statistically not
significantly different, than Asian Hantaan virus (HTNV), suggesting an
unexplained cross-reaction, since PUUV is considered absent throughout Asia.
Moreover, RT-PCR on all hantavirus IgM ELISA positives was negative. Concomitant
leptospirosis-hantavirus infections are probably heavily underestimated
worldwide, compromising epidemiological data, therapeutical decisions, and
clinical outcome.

DOI: 10.1017/S0950268814003707
PMID: 25582980 [PubMed - indexed for MEDLINE]

554. Am J Trop Med Hyg. 2010 Sep;83(3):658-63. doi: 10.4269/ajtmh.2010.09-0608.

Phylogenetic analysis of 56-kDa type-specific antigen gene of Orientia
tsutsugamushi isolates in Taiwan.

Lu HY(1), Tsai KH, Yu SK, Cheng CH, Yang JS, Su CL, Hu HC, Wang HC, Huang JH, Shu
PY.

Author information:
(1)Research and Diagnostic Center, Centers for Disease Control, Department of
Health, Taipei, Taiwan, Republic of China. hsiuying@cdc.gov.tw

Scrub typhus is a rickettsial disease transmitted to humans through the bite of
chigger mites infected with Orientia tsutsugamushi, and is an endemic disease in
Taiwan. To elucidate the molecular epidemiology of O. tsutsugamushi, the complete
open reading frame of the 56-kDa type-specific antigen gene sequence of strains
isolated from scrub typhus patients were determined and analyzed. A total of 116
isolates of O. tsutsugamushi were successfully isolated from patients infected in
diverse geographic origins including Taiwan and three offshore islets, Kinmen,
Matsu, and Penghu between May 2006 and December 2007. Sequence analysis revealed
that 22 distinct sequence types could be identified that were broadly distributed
in different clusters of the phylogenetic tree. Most of the isolates belong to
Karp, Kawasaki, and Kuroki genotypes and are closely related to strains from
Thailand, Japan, and Korea, whereas unique isolates different from other
countries were also found in Taiwan. Distinct seasonal distributions were found
in different sequence types. Some sequence types caused disease in the cold
season, whereas others caused disease in the warm season.

DOI: 10.4269/ajtmh.2010.09-0608
PMCID: PMC2929066
PMID: 20810835 [PubMed - indexed for MEDLINE]

555. Int J Infect Dis. 2008 Mar;12(2):198-202. Epub 2007 Sep 27.

Rickettsial infections and their clinical presentations in the Western Province
of Sri Lanka: a hospital-based study.

Premaratna R(1), Loftis AD, Chandrasena TG, Dasch GA, de Silva HJ.

Author information:

(1)Department of Medicine, Faculty of Medicine, University of Kelaniya, PO Box 6, Thalagolla Rd, Ragama, Sri Lanka. ranjan_premaratna@lycos.com

BACKGROUND: Rickettsial infections are re-emerging. A study of the geographical distribution of rickettsial infections, their clinical manifestations, and their complications would facilitate early diagnosis.
METHODS: Thirty-one selected patients from the Western Province of Sri Lanka were studied for rickettsial species, clinical manifestations, and complications.
RESULTS: Of 31 patients with possible rickettsioses, 29 (94%) fell into the categories of confirmed, presumptive, or exposed cases of acute rickettsial infections (scrub typhus was diagnosed in 19 (66%), spotted fever group in eight (28%)). Early acute infection or past exposure was suggested in two (7%) cases; cross-reactivity of antigens or past exposure to one or more species was suggested in nine (31%). Seventeen out of 19 (89%) patients with scrub typhus had eschars. Nine out of 29 (32%) patients had a discrete erythematous papular rash: seven caused by spotted fever group, two by scrub typhus. Severe complications were pneumonitis in eight (28%), myocarditis in five (17%), deafness in four (14%), and tinnitus in two (7%). The mean duration of illness before onset of complications was 12.0 (SD 1.4) days. All patients except one made a good clinical recovery with doxycycline or a combination of doxycycline and chloramphenicol.
CONCLUSIONS: In a region representing the low country wet zone of Sri Lanka, the main rickettsial agent seems to be Orientia tsutsugamushi. Delay in diagnosis may result in complications. All species responded well to current treatment.

DOI: 10.1016/j.ijid.2007.06.009
PMID: 17900956 [PubMed - indexed for MEDLINE]

556. Microbiol Immunol. 1996;40(9):627-38.

Demonstration of antigenic and genotypic variation in Orientia tsutsugamushi which were isolated in Japan, and their classification into type and subtype.

Ohashi N(1), Koyama Y, Urakami H, Fukuhara M, Tamura A, Kawamori F, Yamamoto S, Kasuya S, Yoshimura K.

Author information:
(1)Department of Microbiology, Niigata College of Pharmacy, Japan.

A total of 40 strains of Orientia tsutsugamushi (34 isolates from patients and trombiculid mites in Japan, and 6 prototype strains of antigenic variants) were examined for classification based on the reactivities with type-specific monoclonal antibodies in indirect immunofluorescence tests, and on the restriction fragment length polymorphism of a polymerase chain reaction (PCR)-amplified 56-kilodalton type-specific antigenic protein gene. By these methods, several antigenic and genotypic variants were found among the strains, and these variants were classified into types and further into subtypes. These results suggest that there are many variants in O. tsutsugamushi, and the methods used here seem to be useful for the systematic classification of the numerous variants. A strain which may be a new type distinguishable from those identified previously was also found in this study. Furthermore, variety in the degree of pathogenicity in mice related to type and/or subtype classification were observed.

PMID: 8908607 [PubMed - indexed for MEDLINE]

557. Microbiol Immunol. 2008 Mar;52(3):135-43. doi: 10.1111/j.1348-0421.2008.00024.x.

Epidemiological characteristics of tsutsugamushi disease in Oita Prefecture, Japan: yearly and monthly occurrences of its infections and serotypes of its causative agent, Orientia tsutsugamushi, during 1984--2005.

Ogawa M(1), Ono T.

Author information:
(1)Oita Prefectural Institute of Health and Environment, Oita, Japan.
ogawa-masao@pref.oita.lg.jp

Using indirect immunofluorescence assay, we examined the sera of 561 patients from November 1984 to February 2005 to determine the incidence of tsutsugamushi disease (scrub typhus) in Oita Prefecture, Japan. The results obtained were positive in 384 individuals (68.4%). Municipalities where patients were presumed to have been infected with Orientia tsutsugamushi were Taketa City (41.7%), Oyama Town (13.5%), and Ogi Town (8.3%). Infections occurred most often in October, November, and December. A small number of cases occurred from January to May. The serotypes Kuroki (47.5%), Kawasaki (42.5%), and Karp (10.0%) were detected by genetic analysis of O. tsutsugamushi DNA extracted from the blood of 120 patients. The gene sequences of the Kuroki type were highly homologous to that of the Nishino strain. The gene sequences of the Kawasaki type were identical to that of the Kawasaki strain. The gene sequence of the Karp type was highly homologous to that of the JP-2 type. To determine the distribution of vector mites, 558 wild rodents were captured and 72010 mites attached to these rodents were collected from 1982 to 1998. Six genera and 16 species of trombiculid mites were collected. Leptotrombidium pallidum and L. scutellare, which are known to be mite vectors for tsutsugamushi disease, accounted for 20.5% and 5.9%, respectively, of all trombiculid mites collected. The geographical distribution of cases roughly coincided with the distribution of L. scutellare. In Oita Prefecture, L. scutellare is presumed to primarily transmit tsutsugamushi disease. In addition, our results also suggest that L. pallidum transmits the Karp type of the causative rickettsia in some municipalities.

DOI: 10.1111/j.1348-0421.2008.00024.x
PMID: 18402595 [PubMed - indexed for MEDLINE]

558. J Clin Microbiol. 2008 Apr;46(4):1548-50. Epub 2007 Nov 14.

Acute renal failure due to acute tubular necrosis caused by direct invasion of Orientia tsutsugamushi.

Kim DM(1), Kang DW, Kim JO, Chung JH, Kim HL, Park CY, Lim SC.

Author information:
(1)Department of Internal Medicine, Chosun University College of Medicine, 588 Seosuk-dong, Dong-gu, Gwangju 501-717, South Korea.

We describe a scrub typhus patient with acute renal failure for whom a diagnosis was made based on serology as well as immunohistochemical (IHC) staining and an electron microscopic examination (EM) of a renal biopsy specimen. For our case, we demonstrated by IHC staining and EM that renal failure was caused by acute tubular necrosis due to a direct invasion of Orientia tsutsugamushi.

DOI: 10.1128/JCM.01040-07
PMCID: PMC2292935
PMID: 18003808 [PubMed - indexed for MEDLINE]

559. Zhonghua Liu Xing Bing Xue Za Zhi. 2004 Aug;25(8):698-701.

[Amplification and typing of Sta56 gene of Orientia tsutsugamushi from Shandong province].

[Article in Chinese]

Liu YX(1), Gao Y, Zhao ZT, Zhang JL, Yang ZQ, Bu XP, Su JJ.

Author information:
(1)School of Public Health, Shandong University, Jinan 250012, China.

OBJECTIVE: To clarify the gene type of Orientia tsutsugamushi (Ot) from Shandong province.
METHODS: Nested-polymerase chain reaction (nPCR) was used to identify the gene type of 23 isolated Ot strains, 2 pools of homogenized leptotrombidium (L.) scutellare, 10 blood specimens of scrub typhus patients, and at the same time to compare with the international reference strains Gilliam, Karp, Kato. Sequencing analysis of the Sta56 gene was also used to further identify the precise gene types.
RESULTS: Of the 35 samples, 33 had the same products in the amplification of template Ot-DNA. They all belonged to Kawasaki strains endemic in Japan while 2 (FXS4 and LHGM2 strain) belonged to Karp strains. The Sta56 gene sequence homologies to Japan Kawasaki strain of the 2 representative strains (B-16 and FXS2 strain) of the 33 samples were 94.22%, 95.21% respectively, but they were less than 75.87% to other prototype strains; The homologies to Karp strain of FXS4 and LHGM2 strain were 83.03%, 96.45% respectively. B-16 and FXS2 strain were designated as of types strain Japan Kawasaki, FXS4 and LHGM2 as Karp strain.
CONCLUSION: The results indicated that the dominant Ot strains in Shandong Province were similar to Kawasaki strains, but Karp strains also existed.

PMID: 15555396 [PubMed - indexed for MEDLINE]

560. Indian J Med Res. 1969 Jul;57(7):1228-31.

Survey of the Eastern Himalayas for endemicity of scrub typhus.

Varma RN.

PMID: 5390556 [PubMed - indexed for MEDLINE]

561. J Electron Microsc (Tokyo). 2008 Oct;57(5):169-74. doi: 10.1093/jmicro/dfn017.

Electron-microscopic observation of mouse spleen tissue infected with Orientia tsutsugamushi isolated from Shandong, China.

Yang L(1), Zhao Z, Li B, Liu Y, Feng Y.

Author information:
(1)Department of Epidemiology and Biostatistics, School of Public Health, Shandong University, Jinan, China.

Low-virulent Orientia tsutsugamushi (Ot) were successfully isolated from scrub typhus patients in Shandong, China, and the isolates were similar to the Kawasaki type identified by nested polymerase chain reaction (PCR). To identify the morphological characterization of the low-virulent Ot, and elucidate the pathological changes on host cells, mouse spleen tissue infected with the Ot isolated from Shandong was used for the ultrastructural study. Transmission electron microscopy showed that the Ot parasitized in the spleen were different in size, shape and electron density and many significant changes occurred in cytoplasmic organelles of the inoculated mouse spleen cells. Swollen perinuclear cisterna was observed in the nuclear membranes of mononuclear cells and a multivesicular body was found in the intracytoplasm of the macrophage. In the phagosome of the macrophage, many Ot enveloped with an additional membrane were found to push the phagosomal membrane outward from inside. The results indicated that the low-virulent Ot and the spleen cells suffered various damages.

DOI: 10.1093/jmicro/dfn017
PMID: 18799810 [PubMed - indexed for MEDLINE]

562. Microbes Infect. 2001 Jan;3(1):11-21.

Orientia tsutsugamushi infection: overview and immune responses.

Seong SY(1), Choi MS, Kim IS.

Author information:
(1)Department of Microbiology and Immunology, Seoul National University College of Medicine and Institute of Endemic Disease, Seoul National University Medical Research Center, 28 Yongon-dong, Chongno-gu, 110-799, Seoul, South Korea.

Orientia tsutsugamushi, an obligate intracellular bacterium, was isolated for the first time in 1930. Infections by virulent strains are characterized by fever, rash, eschar, pneumonia, myocarditis, and disseminated intravascular coagulation. Here we review the general aspects of O. tsutsugamushi and immune responses in terms of inflammation, protective immune mechanisms, and immunogenic antigens.

PMID: 11226850 [PubMed - indexed for MEDLINE]

563. J Med Entomol. 2015 Sep;52(5):1096-102. doi: 10.1093/jme/tjv083. Epub 2015 Jun 27.

Widespread Rickettsia spp. Infections in Ticks (Acari: Ixodoidea) in Taiwan.

Kuo CC(1), Shu PY(2), Mu JJ(2), Lee PL(2), Wu YW(3), Chung CK(4), Wang HC(5).

Author information:
(1)Department of Life Science, National Taiwan Normal University, Taipei, Taiwan. (2)Center for Research, Diagnostics and Vaccine Development, Centers for Disease Control, Ministry of Health and Welfare, Taipei, Taiwan. (3)Department of Food Science, National Quemoy University, Taiwan. (4)Bureau of Animal and Plant Health Inspection, Kinmen County, Taiwan. (5)Center for Research, Diagnostics and Vaccine Development, Centers for Disease Control, Ministry of Health and Welfare, Taipei, Taiwan. sjwang@cdc.gov.tw.

Ticks are second to mosquitoes as the most important disease vectors, and recent decades have witnessed the emergence of many novel tick-borne rickettsial diseases, but systematic surveys of ticks and tick-borne rickettsioses are generally lacking in Asia. We collected and identified ticks from small mammal hosts between 2006 and 2010 in different parts of Taiwan. Rickettsia spp. infections in ticks were identified by targeting ompB and gltA genes with nested polymerase chain reaction. In total, 2,732 ticks were collected from 1,356 small mammals. Rhipicephalus haemaphysaloides Supino (51.8% of total ticks), Haemaphysalis bandicota Hoogstraal & Kohls (28.0%), and Ixodes granulatus Supino (20.0%) were the most common tick species, and Rattus losea Swinhoe (44.7% of total ticks) and Bandicota indica Bechstein (39.9%) were the primary hosts. The average Rickettsia infective rate in 329 assayed ticks was 31.9% and eight Rickettsia spp. or closely related species were identified. This study shows that rickettsiae-infected ticks are widespread in Taiwan, with a high diversity of Rickettsia spp. circulating in the ticks. Because notifiable rickettsial diseases in Taiwan only include mite-borne scrub typhus and flea-borne murine typhus, more studies are warranted for a better understanding of the real extent of human risks to rickettsioses in Taiwan.

© The Authors 2015. Published by Oxford University Press on behalf of Entomological Society of America. All rights reserved. For Permissions, please email: journals.permissions@oup.com.

DOI: 10.1093/jme/tjv083
PMID: 26336223 [PubMed - indexed for MEDLINE]

564. FEMS Microbiol Lett. 1997 May 15;150(2):225-31.

Characterization of Orientia tsutsugamushi isolated in Taiwan by immunofluorescence and restriction fragment length polymorphism analyses.

Tamura A(1), Ohashi N, Koyama Y, Fukuhara M, Kawamori F, Otsuru M, Wu PF, Lin SY.

Author information:
(1)Department of Microbiology, Niigata College of Pharmacy, Japan.

A total of 10 strains of Orientia tsutsugamushi were isolated from field rodents

and chiggers in Taiwan, and characterized by immunofluorescence analysis with monoclonal antibodies and by restriction fragment length polymorphism analysis of the 56-kilodalton (kDa) protein gene. The isolates were divided into two groups consisting of 1 and 9 strains which showed some relation to Gilliam and Karp type strains, respectively. However, all these isolates possessed characteristics distinct not only from those of known prototype strains including Gilliam and Karp but also from all isolates from Japan. These findings suggest the existence of a large number of immunotypic and genotypic variants among the strains of O. tsutsugamushi, and the distribution of distinguishable strains in each area to which this species is endemic.

PMID: 9170266 [PubMed - indexed for MEDLINE]

565. QJM. 2013 Jan;106(1):79-80. doi: 10.1093/qjmed/hcr211. Epub 2011 Nov 3.

Tache noire.

Uen CC(1), Kao JL, Huang CH, Chen IL, Leu SC, Shiao CC.

Author information:
(1)Division of Neurology, Department of Internal Medicine, Saint Mary's Hospital Luodong, Taiwan.

DOI: 10.1093/qjmed/hcr211
PMID: 22052955 [PubMed - indexed for MEDLINE]

566. Am J Trop Med Hyg. 1999 Jul;61(1):73-7.

Seroepidemiologic survey of Orientia tsutsugamushi, Rickettsia typhi, and TT118 spotted fever group rickettsiae in rubber estate workers in Malaysia.

Tee TS(1), Kamalanathan M, Suan KA, Chun SS, Ming HT, Yasin RM, Devi S.

Author information:
(1)Institute for Medical Research, Jalan Pahang, Kuala Lumpur, Malaysia.

The seroprevalence of Orientia tsutsugamushi, Rickettsia typhi, and TT118 spotted fever group (SFG) rickettsiae in 300 rubber estate workers in Slim River, Malaysia was determined in December 1996 and March 1997. In December, which was the wet season, 23.3%, 3.0%, and 57.3% of the population had antibodies detected against the three rickettsiae, respectively. The highest seropositive rate of 40% was detected for single infection with SFG rickettsiae, followed by a rate of 15.3% for both O. tsutsugamushi and SFG rickettsiae among the rubber estate workers. Subjects less than 21 years old had a lower seroprevalence of SFG rickettsiae compared with the other age groups. Indians had a higher seroprevalence of O. tsutsugamushi compared with other ethnic groups. Rubber tappers had a higher seroprevalence of SFG rickettsiae compared with other occupational groups. During the dry season in March 1997, there was a significant increase in the seroprevalence of R. typhi. The seroconversion rates for IgM against O. tsutsugamushi, R. typhi, and SFG rickettsiae were 5.7%, 12.3%, and 15.1%, respectively, during the four-month period. Significant variations of

antibody titers towards the three rickettsiae was noted among subjects who were bled twice. This suggests a significant and continual exposure of rubber estate workers to the three rickettsiae.

PMID: 10432060 [PubMed - indexed for MEDLINE]

567. Clin Diagn Lab Immunol. 2003 May;10(3):451-8.

Development of a recombinant protein-based enzyme-linked immunosorbent assay and its applications in field surveillance of rodent mice for presence of immunoglobulin G against Orientia tsutsugamushi.

Wang YC(1), Jian TY, Tarn LJ, Hung YW, Chao HY, Ji DD, Liu HW.

Author information:
(1)Institute of Preventive Medicine, National Defense Medical Center, Taipei 100, Taiwan. yeauching@ndmctsgh.edu.tw

A recombinant protein containing the immunodominant conserved epitope region of the 56-kDa outer membrane protein of the Karp strain of Orientia tsutsugamushi was purified to near homogeneity using recombinant DNA techniques. The purified protein was used to immunize rabbits and produced an antibody that could recognize different strains of O. tsutsugamushi, as demonstrated both by Western blotting and immunofluorescence assay. An enzyme-linked immunosorbent assay (ELISA) based on this recombinant protein was developed to detect antibody (immunoglobulin G [IgG]) against O. tsutsugamushi in mice captured in different districts of Taiwan during 2000 to 2001. A significant difference was found in the antibody seroprevalence rates of Suncus murinus mice captured in different districts of Taiwan (chi(2)(4, 0.95) = 26.64; P < 0.05). Furthermore, a significant difference of IgG seropositivity rates was observed among different kinds of mice (chi(2)(5, 0.95) = 93.85; P < 0.05). Antibody seropositivity rates were higher in Bandicota indica (100%), Rattus flavipectus (96.17%), and Rattus losea (95.83%) than in Rattus norvegicus (86.05%) and Rattus mindanensis (83.67%) (chi(2)(diff, 5, 0.95) = 12.59, P < 0.05). The lowest antibody seropositivity rate (54.4%) was observed in Suncus murinus. Antibody seropositivity rates of mice from different districts differed significantly because of the significant difference in antibody seroprevalence rates for S. murinus. The results of this study indicated that the recombinant protein ELISA developed in this study could be used to conduct large-scale surveillance of rodent mice for the presence of antibody against O. tsutsugamushi. The high seroprevalence rates in rodent mice (except S. murinus) suggest that people residing in these districts are at increased risk of developing O. tsutsugamushi infection.

PMCID: PMC154969
PMID: 12738648 [PubMed - indexed for MEDLINE]

568. Microbiol Immunol. 1997;41(6):437-43.

Isolation of a new Orientia tsutsugamushi serotype.

Seong SY(1), Park SG, Kim HR, Han TH, Kang JS, Choi MS, Kim IS, Chang WH.

Author information:
(1)Department of Microbiology, Seoul National University College of Medicine, Republic of Korea.

Orientia tsutsugamushi, the etiological agent of scrub typhus, is an antigenically diverse organism and many serologically distinct strains have been identified. The 56 kDa protein of O. tsutsugamushi, a major protein in the outer membrane, has been thought to be responsible for this antigenic variability. A strain of O. tsutsugamushi isolated in Korea cross-reacted with both Gilliam strain-specific and Karp strain-specific monoclonal antibodies. When its 56 kDa protein gene was cloned and analyzed, its sequence showed variation especially between 1,200 and 1,250 bp, showing that this isolate is a new O. tsutsugamushi strain.

PMID: 9251054 [PubMed - indexed for MEDLINE]

569. Int J Occup Environ Health. 2013 Oct-Dec;19(4):344-51.

Work-related infectious diseases among Korean workers compensated under the Industrial Accident Compensation Insurance Law, 2006-2011.

Myong JP(1), Ahn YS(2), Kim HR(1), Kim YJ(3), Park CY(1), Koo JW(1).

Author information:
(1)Department of Occupational and Environmental Medicine, College of Medicine, The Catholic University of Korea, Seoul, Korea. (2)Department of Occupational and Environmental Medicine, Dongguk University, Ilsan Hospital, Goyang, Republic of Korea. (3)Division of Infectious Disease, Department of Internal Medicine, Seoul St. Mary's Hospital,The Catholic University of Korea, Seoul, Korea.

BACKGROUND: Korea has no surveillance system for work-related infectious disease. However, these diseases are compensated by the Korea Workers' Compensation & Welfare Service (KCOMWEL).
OBJECTIVES: To understand the nature and distribution of compensated occupational infectious diseases in Korea.
METHODS: We used the KCOMWEL electronic database to analyze compensated cases of work-related occupational infectious disease. We reviewed and confirmed diagnoses excluding denied claims, secondary infections, dermatoid diseases, duplicated cases and those with missing information. We calculated the distribution of work-related infectious disease in Korea by occupation, calendar year, gender, age, and employment duration, as well as the annual compensated claim rates (per million).
RESULTS: We included 1,062 compensated cases of work-related infectious disease. The most common was scrub typhus (n = 567, 53.4%), followed by tuberculosis (n = 227, 21.4%), viral hepatitis (n = 55, 5.2%), and viral influenza (n = 53, 5.0%). A sudden increase in scrub typhus was observed in 2009. Unskilled laborers, including short-term contract workers in public sectors, were most commonly affected by these diseases, followed by health care professionals.
CONCLUSIONS: Workers employed in forestry care in the public sectors and in hospitals were most vulnerable to infections. Proper surveillance systems to

monitor infectious diseases among vulnerable working groups and improved prevention measures are needed.

DOI: 10.1179/2049396713Y.0000000042
PMID: 24588041 [PubMed - indexed for MEDLINE]

570. Int J Dermatol. 1992 Dec;31(12):823-32.

Typhus disease group.

Boyd AS(1), Neldner KH.

Author information:
(1)Department of Dermatology, Texas Tech University Health Sciences Center School of Medicine, Lubbock.

PMID: 1478757 [PubMed - indexed for MEDLINE]

571. Parasitol Res. 2016 Feb;115(2):623-32.

Ectoparasitic chigger mites on large oriental vole (Eothenomys miletus) across southwest, China.

Peng PY, Guo XG, Song WY, Hou P, Zou YJ, Fan R.

An investigation of chigger mites on the large oriental vole, Eothenomys miletus (Rodentia: Cricetidae), was conducted between 2001 and 2013 at 39 localities across southwest China, and 2463 individuals of the vole hosts were captured and examined, which is a big host sample size. From the body surface of E. miletus, 49,850 individuals of chigger mites were collected, and they were identified as comprising 175 species, 13 genera, and 3 subfamilies in 2 families (Trombiculidae and Leeuwenhoekiidae). The 175 species of chigger mites from such a single rodent species (E. miletus) within a certain region (southwest China) extremely exceeded all the species of chigger mites previously recorded from multiple species of hosts in a wide region or a whole country in some other countries, and this suggests that E. miletus has a great potential to harbor abundant species of chigger mites on its body surface. Of 175 mite species, Leptotrombidium scutellare was the most dominant species, which has been proved as one of the main vectors of scrub typhus and the potential vector of haemorrhagic fever with renal syndrome (HFRS) in China. The patchiness index (m*/m) was used to measure the spatial patterns of the dominant chigger mite species, and all the three dominant mite species (L. scutellare, Leptotrombidium sinicum, and Helenicula simena) showed aggregated distributions among the different host individuals. The coefficient of association (V) was adopted to measure the interspecies interaction between the dominant mite species and a slightly positive association existed between L. scutellare and L. sinicum ($V = 0.28$, $P < 0.01$), which implies that these two mite species can co-exist on the same species of the host, E. miletus. The tendency curve of species abundance showed that the number of chigger mite species gradually decreased with the increase of mite individuals, and this revealed that most chigger mite species were rare with very few individuals, but few dominant species had abundant individuals. The

species-sample relationship indicated that the number of chigger mite species increased with the increase of the host samples. The results suggest that a big host sample size over a wide realm of geographical regions is needed in the field investigation in order to obtain a true picture of species diversity and species composition.

DOI: 10.1007/s00436-015-4780-9
PMID: 26468149 [PubMed - indexed for MEDLINE]

572. Am J Kidney Dis. 2016 Jul;68(1):148-60. doi: 10.1053/j.ajkd.2016.01.017. Epub 2016 Mar 2.

CKD and Infectious Diseases in Asia Pacific: Challenges and Opportunities.

Jha V(1), Prasad N(2).

Author information:
(1)Department of Nephrology, Postgraduate Institute of Medical Education and Research, Chandigarh, India; Department of Nephrology, George Institute for Global Health, New Delhi, India; Department of Nephrology, University of Oxford, Oxford, United Kingdom. Electronic address: vjha@pginephro.org. (2)Department of Nephrology, Sanjay Gandhi Postgraduate Institute of Medical Sciences, Lucknow, India.

The exact number of patients with chronic kidney disease (CKD) in Asia Pacific is uncertain. In numeric terms, the region is home to the largest population of patients with untreated chronic kidney failure. The climatic, geographic, social, cultural, economic, and environmental diversity within this region is higher than in any other part of the world. Large parts of the region face a climate-related burden of infectious diseases. Infections contribute to the development and progression of CKD and complicate the course of patients with pre-existing CKD (especially those on dialysis therapy or who are immunosuppressed), increase the cost of CKD care, and contribute to mortality and morbidity. Kidney involvement is a feature of several infectious diseases prevalent in Asia Pacific. Examples include malaria, leptospirosis, scrub typhus, tuberculosis, hepatitis B and C virus, dengue hemorrhagic fever, and Hantaan virus infections. The contribution of infection-associated acute kidney injury to the overall burden of CKD has not been evaluated systematically. Research is needed to quantify the impact of infections on kidney health by undertaking prospective studies. Nephrologists need to work with infectious disease research groups and government infection surveillance and control programs.

Copyright © 2016 National Kidney Foundation, Inc. Published by Elsevier Inc. All rights reserved.

DOI: 10.1053/j.ajkd.2016.01.017
PMID: 26943982 [PubMed - in process]

573. Ann N Y Acad Sci. 2003 Jun;990:527-34.

Immunogenicity of a 40kDa fragment of the 47kDa recombinant protein and DNA vaccine from Karp strain of Orientia tsutsugamushi.

Niu D(1), Chen W, Zhang X, Chen M, Cui H, Wei W, Wen B, Chen X.

Author information:
(1)Beijing Institute of Microbiology and Epidemiology, Beijing, China 100071.

In this study, the fragment of 47 kDa gene (301 bp-1428 bp) was cloned into a prokaryotic expression vector pBV220 to construct a recombinant plasmid pBV-47. The E. coli cells were transformed with pBV-47 and the transformants were induced to express the recombinant protein at 42 degrees C. The expression product (40 kDa) was detected by SDS-PAGE analysis and the 40kDa protein was recognized by mouse polyclonal antibodies against O. tsutsugamushi Karp strain in western blot analysis. The entire 47 kDa protein gene was inserted into an eukaryotic expression vector pcDNA3.1(+) to construct a recombinant plasmid pcDNA3.1/47 and Balb/c mice were immunized with recombinant pcDNA3.1/47, control vector pcDNA3.1, PBS buffer, 40 kDa protein, and recombinant pcDNA3.1/47 plus 40 kDa protein (pcDNA3.1/47/40), respectively. The results showed that spleen cells from pcDNA3.1/47/40-immunized mice gave higher proliferation than other groups. A significant IgG rise was detected in mice immunized with 40 kDa protein, but it was less strong than that in mice immunized with pcDNA3.1/47/40. The results suggested that immunization with pcDNA3.1/47 and 40 kDa protein simultaneously could induce a strong immune response.

PMID: 12860686 [PubMed - indexed for MEDLINE]

574. Med J Aust. 1946 Nov 9;2(19):666-8.

The epidemiology of North Queensland tick typhus; natural mammalian hosts.

FENNER F.

PMID: 20277613 [PubMed - indexed for MEDLINE]

575. Indian J Med Res. 1951 Jul;39(3):297-302.

Typhus fevers in Kashmir State. Part II. Murine typhus.

KALRA SL, RAO KN.

PMID: 14937743 [PubMed - indexed for MEDLINE]

576. Infect Immun. 2003 Aug;71(8):4772-9.

Recombinant 56-kilodalton major outer membrane protein antigen of Orientia tsutsugamushi Shanxi and its antigenicity.

Chen WJ(1), Niu DS, Zhang XY, Chen ML, Cui H, Wei WJ, Wen BH, Chen XR.

Author information:
(1)Institute of Microbiology and Epidemiology, Beijing 100071, China.

The gene encoding the 56-kDa protein of Orientia tsutsugamushi Shanxi was amplified by a nested PCR and cloned into the expression vector pQE30. The 56-kDa protein of O. tsutsugamushi Shanxi (Sxh56) was expressed as a fusion protein with the His(6)-binding protein of Escherichia coli by deleting the signal peptide-encoding sequence from the 5' end of the open reading frame. The recombinant protein formed inclusion bodies when expressed in E. coli M15. The recombinant protein was examined for reactivity with mouse sera against three antigenic prototypes of O. tsutsugamushi by an immunoblot assay. The recombinant Sxh56 reacted only to polyclonal antiserum to O. tsutsugamushi Gilliam in an enzyme-linked immunosorbent assay (ELISA) and in an immunoblot assay. Recombinant Sxh56 was purified by Ni-nitrilotriacetic acid affinity chromatography and injected into mice to evaluate its ability to stimulate immune responses. High levels of immunoglobulin G and T-cell proliferation appeared in mice immunized with the recombinant protein. The recombinant Sxh56 was used in an ELISA to evaluate the ability of the method to detect antibodies to O. tsutsugamushi in human and animal sera. Thirty sera from mice infected with O. tsutsugamushi Gilliam or Shanxi and 55 sera from normal mice were detected in the ELISA with recombinant Sxh56, and the sensitivity and specificity were 96.67 and 100%, respectively. One hundred fifty-one positive sera and 412 negative sera to O. tsutsugamushi Gilliam were detected in an indirect immunofluorescence assay with the recombinant protein, and the sensitivity and specificity were 96.36 and 88.08%, respectively. These results strongly suggest that the recombinant Sxh56 is a suitable type-specific immunodiagnostic antigen and vaccine candidate.

PMCID: PMC166048
PMID: 12874360 [PubMed - indexed for MEDLINE]

577. Kaohsiung J Med Sci. 2005 Apr;21(4):173-8.

Fatal septicemic melioidosis in a young military person possibly co-infected with Leptospira interrogans and Orientia tsutsugamushi.

Lu PL(1), Tseng SH.

Author information:
(1)Department of Internal Medicine, Kaohsiung Medical University Hospital, Kaohsiung, Taiwan. d830166@cc.kmu.edu.tw

Concurrent melioidosis, leptospirosis, and scrub typhus after rural activities is rarely reported. A 19-year-old previously healthy man had fever onset after 2 weeks of military training. Pneumonia became evident on the fifth day of fever under intravenous penicillin and oral minocycline therapy. Acute respiratory failure developed the next day with shock and acute renal and liver function deterioration, which resulted in death. Blood cultures on the third and fifth days grew Burkholderia pseudomallei. Serology revealed leptospirosis and scrub typhus. The emergence of melioidosis in Taiwan and this death without antibiotic treatment for melioidosis alert us that B. pseudomallei should be included as a possible pathogen of pneumonia and sepsis, especially after rural activities.

DOI: 10.1016/S1607-551X(09)70297-9
PMID: 15909673 [PubMed - indexed for MEDLINE]

578. Jpn J Infect Dis. 2000 Jun;53(3):126-7.

Annual incidence of tsutsugamushi disease caused by different serotypes of Orientia tsutsugamushi in Miyazaki Prefecture in 1991-1999.

Yamamoto S(1), Kizoe K, Yoshino S, Hagiwara T.

Author information:
(1)Miyazaki Prefectural Institute for Public Health and Environment, Miyazaki 889-2155, Japan.

PMID: 10957711 [PubMed - indexed for MEDLINE]

579. J Infect Dev Ctries. 2016 Jun 30;10(6):657-61. doi: 10.3855/jidc.6822.

Five-year analysis of rickettsial fevers in children in South India: Clinical manifestations and complications.

Thomas R(1), Puranik P, Kalal B, Britto C, Kamalesh S, Rego S, Shet A.

Author information:
(1)St Johns' Medical College Hospital, Bangalore, India.
rwituja.thomas@gmail.com.

INTRODUCTION: Rickettsial infections are re-emerging in the Indian subcontinent, especially among children. Understanding geographical and clinical epidemiology will facilitate early diagnosis and management.
METHODOLOGY: Children aged <18yrs hospitalized with clinically-diagnosed rickettsial fever were reviewed retrospectively. Frequency distributions and odds ratios were calculated from tabulated data.
RESULTS: Among 262 children hospitalized between January 2008-December 2012, median age was five years, and 61% were male children. Hospitalized cases increased steadily every year, with the highest burden (74%) occurring between September and January each year. Mean duration of fever was 11.5 days. Rash was present in 54.2% (142/262) of children, with 37.0% involving palms and soles. Prevalence of malnutrition was high (45% of children were underweight and 28% had stunting). Retinal vasculitis was seen in 13.7% (36/262), and the risk appeared higher in females. Severe complications were seen in 29% (purpura fulminans, 7.6%; meningitis and meningoencephalitis, 28%; septic shock, 1.9%; acute respiratory distress syndrome, 1.1%). Complications were more likely to occur in anemic children. Positive Weil-Felix test results (titers ≥1:160) were seen in 70% of cases. Elevated OX-K titers suggestive of scrub typhus were seen in 80% (147/184). Patients were treated with chloramphenicol (32%) or doxycycline (68%). Overall mortality among hospitalised children was 1.9%.
CONCLUSIONS: This five-year analysis from southern India shows a high burden and increasing trend of rickettsial infections among children. The occurrence of retinal vasculitis and a high rate of severe complications draw attention to the need for early diagnosis and management of these infections.

PMID: 27367015 [PubMed - in process]

580. Zhonghua Liu Xing Bing Xue Za Zhi. 2011 Jan;32(1):13-6.

[Preliminary survey on the distribution of Leptotrombidium deliense in some areas of Yunnan province].

[Article in Chinese]

Zhan YZ(1), Guo XG, Zuo XH, Wang QH, Wu D.

Author information:
(1)Institute of Pathogens and Vectors, Dali University, Dali 671000, China.

OBJECTIVE: To investigate the distribution of Leptotrombidium deliense among different small mammal hosts in some areas of Yunnan province.
METHODS: A field survey was carried out in some counties of Yunnan province and the small mammal hosts were captured, using mouse cages and traps with baits. Chigger mites on the surface of two auricles were scraped off by a bistoury, and then preserved in 70% ethanol. Every specimen of the chigger mites on the slides was finally identified into species under a microscope. Some conventional statistical methods were adopted to calculate all the collected chigger mite species and the constituent ratios of Leptotrombidium deliense in different areas and on different hosts, together with its prevalence and mean abundance on different hosts.
RESULTS: A total of 10 222 small mammal hosts were captured from 19 counties and identified as 11 families, 34 genera and 62 species in 5 orders, and 92 990 individuals of chigger mites were collected from the body surface of these small mammal hosts. All the collected chigger mites were identified as 3 subfamilies, 22 genera, and 225 species. Meanwhile, Leptotrombidium deliensee only accounted for 1.659% of the total. The host specificity of Leptotrombidium deliense was very low and 1544 individuals of Leptotrombidium deliense collected from 8518 small mammal hosts belonged to 6 families, 13 genera and 19 species in 3 orders. Our results showed that Leptotrombidium delienses were mainly collected from Insectivora and Rodentia. Leptotrombidium deliense had long been considered as the dominant species of chigger mites and the main vector of tsutsugamushi disease in Yunnan province of China, but our results seemed not thoroughly supporting this point of view.
CONCLUSION: Traditionally, Leptotrombidium deliense was the dominant species and the main vectors of scrub typhus in Yunnan province. However, based on our results, the above view might be true in some local places and the composition of chigger mites and the main vector of tsutsugamushi disease might be different in regions and habitats in Yunnan province.

PMID: 21518533 [PubMed - indexed for MEDLINE]

581. Am J Trop Med Hyg. 2015 Sep;93(3):539-41. doi: 10.4269/ajtmh.15-0246. Epub 2015 Jun 8.

Outbreak of Human Brucellosis from Consumption of Raw Goats' Milk in Penang, Malaysia.

Leong KN(1), Chow TS(2), Wong PS(2), Hamzah SH(2), Ahmad N(2), Ch'ng CC(2).

Author information:
(1)Penang General Hospital, Georgetown, Penang, Malaysia; Institute of Medical Research, Kuala Lumpur Federal Territory, Malaysia; Clinical Research Center, Penang General Hospital, Georgetown, Penang, Malaysia karniml@gmail.com.
(2)Penang General Hospital, Georgetown, Penang, Malaysia; Institute of Medical Research, Kuala Lumpur Federal Territory, Malaysia; Clinical Research Center, Penang General Hospital, Georgetown, Penang, Malaysia.

We report the largest outbreak of brucellosis in Penang, Malaysia. Brucellosis is not endemic in this region. The index case was a 45-year-old goat farm owner presented with 3 weeks of fever, headache, severe lethargy, poor appetite, and excessive sweating. He claimed to have consumed unpasteurized goat's milk that he had also sold to the public. Tests were negative for tropical diseases (i.e., dengue fever, malaria, leptospirosis and scrub typhus) and blood culture showed no growth. Based on epidemiological clues, Brucella serology was ordered and returned positive. Over a period of 1 year, 79 patients who had consumed milk bought from the same farm were diagnosed with brucellosis. Two of these patients were workers on the farm. Four laboratory staff had also contracted the disease presumably through handling of the blood samples. The mean duration from onset of symptoms to diagnosis was 53 days with a maximum duration of 210 days. A combination treatment of rifampin and doxycycline for 6 weeks was the first line of treatment in 90.5% of patients. One-third of the patients had sequelae after recovering and 21% had a relapse. We highlight the importance of Brucellosis as a differential diagnosis when a patient has unexplained chronic fever.

© The American Society of Tropical Medicine and Hygiene.

DOI: 10.4269/ajtmh.15-0246
PMCID: PMC4559693
PMID: 26055742 [PubMed - indexed for MEDLINE]

582. Vaccine. 2000 Aug 15;19(1):2-9.

Neutralization epitopes on the antigenic domain II of the Orientia tsutsugamushi 56-kDa protein revealed by monoclonal antibodies.

Seong SY(1), Kim MK, Lee SM, Odgerel Z, Choi MS, Han TH, Kim IS, Kang JS, Lim BU.

Author information:
(1)Department of Microbiology and Immunology, Seoul National University College of Medicine and Institute of Endemic Disease, Seoul National University Medical Research Center, 28 Yongon-dong, Chongno-gu, 110-799, Seoul, South Korea.

Monoclonal antibodies (MoAbs) reactive with the authentic Orientia tsutsugamushi 56-kDa protein were generated. MoAb FS10 and FS15 showed in vitro, as well as, in vivo neutralizing activity upon O. tsutsugamushi infection. Deletion mutants of the gene for 56-kDa protein of O. tsutsugamushi Boryong were expressed to map the binding region. FS10 and FS15 are bound to amino acids (aa) located in an

antigenic domain II, at residues 140-160 and 187-214, respectively. Computer modeling indicated that aa 146-153 were important for antigenicity against FS10. A sequence for aa 142-150 was highly homologous between oriential strains. These results suggest that the antigenic determinant for neutralizing MoAbs is an epitope within aa 140-160. Furthermore, this region may be important for the adhesion/invasion or intracellular survival of O. tsutsugamushi within host cells.

PMID: 10924780 [PubMed - indexed for MEDLINE]

583. Zhonghua Liu Xing Bing Xue Za Zhi. 2006 Jun;27(6):475-8.

[Investigation on rodents' natural infection of Orientia tsutsugamushi in some areas of Inner Mongolia and Xinjiang, China].

[Article in Chinese]

Zhang Q(1), Liu YX, Wu XM, Zhao QM, Zhang PH, Yang H, Cao WC.

Author information:
(1)State Key Laboratory of Pathogen and Biosecurity, Institute of Microbiology and Epidemiology, Beijing 100071, China.

OBJECTIVE: To investigate rodents' natural infection of Orientia tsutsugamushi (Ot) in some areas of Inner Mongolia and Xinjiang, China.
METHODS: DNAs were extracted from spleens of the captured mice and nested-polymerase chain reaction (nPCR) technique was used to detect the Ot-Sta56 gene. Six positive samples were sequenced and analyzed by Clustal X (5.0) and DNA Club software.
RESULTS: A total of 90 rodents were captured in Inner Mongolia, and the overall prevalence of Ot was 6.67%. There was no significant difference in infection rates among the positive rodents species. 20 rodents were captured in Xinjiang, and the prevalence of Ot was 5.00%. The geographical difference in infection rates was not statistically significant between Inner Mongolia and Xinjiang. 9 rodents were captured in farmlands of Inner Mongolia and Xinjiang but there was no positive samples found. 101 rodents were captured in grasslands, and the prevalence of Ot was 6.93%. The Sta56 gene nucleotide sequence homology to Karp strain of N59 (from Microtus maximowiczii), N69 (from Cricetulus barabensis) and X33(from Cricetus cricetus) was 99%. The sequence homology to Taitung-2 strain and TW461 strain of N65 (from C. barabensis) was 94%, and the sequence homology to Taitung-2 strain and TW461 strain of N88(from Apodemus agrarius) was also 94%. The sequence homology to Oishi strain of N90 (from A. agrarius) was 96.00%.
CONCLUSION: Our findings indicated that infections of Ot did exist in rodents captured from Inner Mongolia and Xinjiang. The genotypes of Ot in Inner Mongolia and Xinjiang were quite complex, with some of them belonged to Karp type, and the others belonged to Taitung-2, TW461 and Oishi types which providing evidence for further investigation on the scrub typhus fuci in the two areas.

PMID: 17152505 [PubMed - indexed for MEDLINE]

584. Kansenshogaku Zasshi. 1999 Dec;73(12):1194-8.

[Analysis of prevalent Orientia tsutsugamushi in Aichi Prefecture].

[Article in Japanese]

Yamashita T(1), Morishita T, Tsuzuki H, Sakae K, Suzuki Y, Kadosaka T, Kasuya S.

Author information:
(1)Department of Microbiology, Aichi Prefectural Institute of Public Health.

Orientia tsutsugamushi was isolated from one of 8 patients' sera in Aichi Prefecture, and was identified to have the same antigenicity with the KN-2 strain (KN-2 like) based on the reactivity with 13 types of strain-specific or cross-reactive monoclonal antibodies to Karp, Gilliam, and Kato strains. Four isolates from 4 unfed larvae and adult of Leptotrombidium pallidum were also classified as the KN-3 like strains. Using indirect immunofluorescence, sera from 20 patients with tsutsugamushi disease were tested for reactivity with KN-1, KN-2, KN-3, and GJ-1 strains, isolated from patients in Gifu Prefecture. Fifteen sera showed the highest titer against KN-2 strain in Immunogloburin M (IgM). Of the other 5, three were higher for KN-3 strain in IgM, and two were KN-1 or GJ-1, respectively. These results suggested that KN-2 like strains were prevalent in the region where the number of patients has been ranked the highest in Aichi Prefecture. KN-1, KN-3, and GJ-1 like strains were also existed in this area. KN-3 like strain was likely to be distributed in another area. Aichi Prefecture.

PMID: 10655679 [PubMed - indexed for MEDLINE]

585. Emerg Infect Dis. 2006 Feb;12(2):256-62.

Rickettsial infections and fever, Vientiane, Laos.

Phongmany S(1), Rolain JM, Phetsouvanh R, Blacksell SD, Soukkhaseum V, Rasachack B, Phiasakha K, Soukkhaseum S, Frichithavong K, Chu V, Keolouangkhot V, Martinez-Aussel B, Chang K, Darasavath C, Rattanavong O, Sisouphone S, Mayxay M, Vidamaly S, Parola P, Thammavong C, Heuangvongsy M, Syhavong B, Raoult D, White NJ, Newton PN.

Author information:
(1)Mahosot Hospital, Vientiane, Laos.

Rickettsial diseases have not been described previously from Laos, but in a prospective study, acute rickettsial infection was identified as the cause of fever in 115 (27%) of 427 adults with negative blood cultures admitted to Mahosot Hospital in Vientiane, Laos. The organisms identified by serologic analysis were Orientia tsutsugamushi (14.8%), Rickettsia typhi (9.6%), and spotted fever group rickettsia (2.6% [8 R. helvetica, 1 R. felis, 1 R. conorii subsp. indica, and 1 Rickettsia "AT1"]). Patients with murine typhus had a lower frequency of peripheral lymphadenopathy than those with scrub typhus (3% vs. 46%, p<0.001). Rickettsioses are an underrecognized cause of undifferentiated febrile illnesses among adults in Laos. This finding has implications for the local empiric treatment of fever.

DOI: 10.3201/eid1202.050900
PMCID: PMC3373100
PMID: 16494751 [PubMed - indexed for MEDLINE]

586. Parasitol Res. 2015 Aug;114(8):2815-33. doi: 10.1007/s00436-015-4483-2. Epub 2015 May 2.

Faunal analysis of chigger mites (Acari: Prostigmata) on small mammals in Yunnan province, southwest China.

Peng PY(1), Guo XG, Ren TG, Song WY.

Author information:
(1)Vector Laboratory, Institute of Pathogens and Vectors, Dali University (Branch of Yunnan Provincial Key Laboratory for Zoonosis Control and Prevention), Dali, Yunnan Province, 671000, People's Republic of China.

This paper studied the species diversity and fauna distribution of chigger mites on small mammals in Yunnan province, southwest Yunnan. In total, 120,138 individuals of chigger mites were collected from 13,760 individual small mammals, and these mites were identified as comprising two families, 26 genera, and 274 species. Of the five zoogeographical subregions, the mite species diversity in subregions I and II was higher than that in subregions III, IV, and V. Four mite species (Leptotrombidium scutellare, Leptotrombidium sinicum, Leptotrombidium deliense, and Helenicula simena) were the most dominant species in the whole province. Several vector species of chigger mites co-existed in Yunnan, and L. deliense (a main vector of scrub typhus in China) was mainly distributed in subregions IV and V with lower latitude and average altitude whereas L. scutellare (also a main vector in China) was mainly distributed in subregions I, II, and III with higher latitude and average altitude. Some geographically widely distributed mite species were also the mites with wide host ranges and low host specificity. The dominant mite species and their clustering tendency in the dendrogram of hierarchical clustering analysis were highly in accordance with the zoogeographical divisions. The species diversity of chigger mites showed a parabolic tendency from the low altitude (<500 m) to the high altitude (>3,500 m) along the vertical gradients and reached the highest value in the middle altitude regions in 2,000-2,500 m. The highest species diversity of the mites and their small mammal hosts happened in the regions around the Hengduan Mountains, which is a hotspot of biodiversity in Asia continent. The host and its sample size, geographical scope, landscape, topography, and some other factors comprehensively influence the species diversity and faunal distribution of chigger mites. A systematic field investigation with a wide geographical scope and large host sample is strongly recommended in the fauna study of chigger mites and other ectoparasites.

DOI: 10.1007/s00436-015-4483-2
PMID: 25930112 [PubMed - indexed for MEDLINE]

587. Rev Infect Dis. 1987 Jul-Aug;9(4):823-40.

Epidemiology and ecology of rickettsial diseases in the People's Republic of

China.

Fan MY(1), Walker DH, Yu SR, Liu QH.

Author information:
(1)Department of Rickettsiology, Chinese Academy of Preventive Medicine, Beijing.

Since 1949, information on rickettsial diseases in the People's Republic of China has been virtually nonexistent in the West. This is the first comprehensive review of the ecology and epidemiology of Chinese rickettsial diseases to be published outside the People's Republic. At least five rickettsioses exist in China: scrub typhus, murine typhus, epidemic typhus, Q fever, and one or more spotted fever-group (SFG) rickettsioses. Although epidemic typhus has been controlled and scrub typhus has abated in many areas, murine typhus, Q fever, and SFG rickettsiosis are important public health problems. Serologic surveys indicate high prevalences of antibodies to Coxiella burnetii, Rickettsia tsutsugamushi, and SFG rickettsiae in some regions; these rickettsiae have been isolated from humans, arthropods, and animals. Doxycycline has emerged as the best treatment for murine typhus, epidemic typhus, and scrub typhus. China offers both opportunities and challenges for the investigation and alleviation of the problems of rickettsial diseases.

PMID: 3326129 [PubMed - indexed for MEDLINE]

588. J Assoc Physicians India. 2009 Apr;57:334-7.

Rickettsial diseases in Haryana: not an uncommon entity.

Chaudhry D(1), Garg A, Singh I, Tandon C, Saini R.

Author information:
(1)Post Graduate Institute of Medical Sciences, Rohtak.

Rickettsioses have not been reported from the plains of North India and Haryana in particular. Here we are reporting three cases of scrub typhus and one cases of Indian tick typhus in the state of Haryana, all of which presented with fever and multi organ dysfunction, rash and without eschar. All were successfully treated with doxycycline.

PMID: 19702040 [PubMed - indexed for MEDLINE]

589. J Infect Dis. 1946 Mar-Apr;78:167-72.

Endemic typhus in Manila, Philippine Islands; report of cases and identification of the murine rickettsial agent in domestic rats by complement fixation.

WOODWARD TE, PHILIP CB, LORANGER GL.

PMID: 21025260 [PubMed - indexed for MEDLINE]

590. Med Vet Entomol. 2013 Jun;27(2):194-202. doi: 10.1111/j.1365-2915.2012.01053.x.
Epub 2012 Nov 20.

Abundances and host relationships of chigger mites in Yunnan Province, China.

Zhan YZ(1), Guo XG, Speakman JR, Zuo XH, Wu D, Wang QH, Yang ZH.

Author information:
(1)Vector Laboratory, Institute of Pathogens and Vectors, Dali University, Dali,
Yunnan, China.

This paper reports on ectoparasitic chigger mites found on small mammals in Yunnan Province, southwest China. Data were accumulated from 19 investigation sites (counties) between 2001 and 2009. A total of 10 222 small mammal hosts were captured and identified; these represented 62 species, 34 genera and 11 families in five orders. From the body surfaces of these 10 222 hosts, a total of 92 990 chigger mites were collected and identified microscopically. These represented 224 species, 22 genera and three subfamilies in the family Trombiculidae (Trombidiformes). Small mammals were commonly found to be infested by chigger mites and most host species harboured several species of mite. The species diversity of chigger mites in Yunnan was much higher than diversities reported previously in other provinces of China and in other countries. A single species of rodent, Eothenomys miletus (Rodentia: Cricetidae), carried 111 species of chigger mite, thus demonstrating the highest species diversity and heaviest mite infestation of all recorded hosts. This diversity is exceptional compared with that of other ectoparasites. Of the total 224 mite species, 21 species accounted for 82.2% of all mites counted. Two species acting as major vectors for scrub typhus (tsutsugamushi disease), Leptotrombidium scutellare and Leptotrombidium deliense, were identified as the dominant mite species in this sample. In addition to these two major vectors, 12 potential or suspected vector species were found. Most species of chigger mite had a wide range of hosts and low host specificity. For example, L. scutellare parasitized 30 species of host. The low host specificity of chigger mites may increase their probability of encountering humans, as well as their transmission of scrub typhus among different hosts. Hierarchical clustering analysis showed that similarities between different chigger mite communities on the 18 main species of small mammal host did not accord with the taxonomic affinity of the hosts. This suggests that the distribution of chigger mites may be strongly influenced by the environment in which hosts live.

© 2012 The Royal Entomological Society.

DOI: 10.1111/j.1365-2915.2012.01053.x
PMID: 23167491 [PubMed - indexed for MEDLINE]

591. Kansenshogaku Zasshi. 2009 Sep;83(5):496-9.

[Occurrence of Tsutsugamushi disease infection by Orientia tsutsugamushi,
Kawasaki serotype, in Yamagata Prefecture, Japan].

[Article in Japanese]

Otani K(1), Kaneko A, Aoki T, Murata T.

Author information:
(1)Yamagata Prefectural Institute of Public Health.

Of 95 Tsutsugamushi disease case occurring in Yamagata prefecture from 1999 to 2006, four-all women-involved the O. tsutsugamushi Kawasaki serotype. The three major symptoms were fever, exanthema, and eschar present from mid-October to early November. Serodiagnosis by indirect immunofluoresence assay showed elevated IgG and IgM antibody titers against the Kawasaki serotype antigen, with IgM higher than IgG. Nested PCR detected 56-kDa DNA in three of the cases. DNA was amplified in Kawasaki-specific PCR. Two cases for which sequencing was done using nested PCR-amplified DNA showed an identity of 99.8% for the Kawasaki strain (Accession number: M63383). These results confirmed the occurrence of Tsutsugamushi disease infection involving Kawasaki serotype in Yamagata prefecture.

PMID: 19860249 [PubMed - indexed for MEDLINE]

592. J Med Assoc Thai. 2003 Jun;86(6):516-21.

Seroprevalence of rickettsial infection in commensal rodents and shrews trapped in the Bangkok Metropolitan Area.

Siritantikorn S(1), Sangkasuwan V, Eamsila C, Singchai C, Kantakamalakul W, Puthavathana P.

Author information:
(1)Department of Microbiology, Faculty of Medicine Siriraj Hospital, Mahidol University, Bangkok 10700, Thailand.

Murine typhus and scrub typhus are important human rickettsial diseases in Thailand. Small mammals, including many species of rodents and shrews, serve as the reservoir host of rickettsial diseases. Rickettsia typhi can be transmitted to humans by fleas causing murine typhus, while infection with Orientia tsutsugamushi causing scrub typhus in humans is transmitted by chiggers. The prevalence of rickettsial infection depends on the geographic area. The seroprevalence of antibody to R. typhi and O. tsutsugamushi was studied in commensal rodents and shrews trapped in markets in the Bangkok Metropolitan Area (BMA). R. typhi and O. tsutsugamushi antigen prepared in the yolk sac of embryonated eggs were used to determine the specific antibody in trapped animals' sera by using fluorescein isothiocyanate (FITC)-anti rat immunoglobulins as a second antibody. Antibody to R. typhi was found in 25 (5%) of 500 sera tested and no antibody to O. tsutsugamushi was detected. R. typhi antibody titer ranged from 40-1280 and was found in Rattus norvegicus (4.2%), Rattus rattus (0.4%), Rattus exulans (0.2%), and Mus musculus (0.2%) trapped in 8 of 47 markets in the BMA. R. typhi antibody was commonly found in R. norvegicus. The authors concluded that murine typhus is an important rickettsial disease and R. norvegicus is an important reservoir species of rodents found in markets of the BMA.

PMID: 12924799 [PubMed - indexed for MEDLINE]

593. Zhonghua Yu Fang Yi Xue Za Zhi. 2008 Aug;42(8):574-7.

[Molecular epidemiological study on the host and role of the Hantavirus and Orientia tsutsugamushi in the same epidemic area].

[Article in Chinese]

Deng XZ(1), Zhang Y, Kong J, Wang ZC, Yang ZQ, Huang WC, Su DM, Yan GJ, Yang ZQ.

Author information:
(1)Medical Institute of Nanjing Army, Nanjing, China.

OBJECTIVE: To investigate whether Hantavirus (HV) and Orientia tsutsugamushi (OT) can naturally infect and coexist in their host and role.
METHODS: By field epidemiological study, Leptotrombidium scutellare (3829) was collected and separated from mice(166) in epidemic areas. The cells of mites separated from their host and role were cultured. PCR was used to detect HV-RNA and OT-DNA in the cell culture.
RESULTS: In 105 Apodemus agrarius, 3 HV-RNA positive, 2 OT-DNA positive and 2 coinfection with HV and OT were detected;in 41 Brown rattus, 2 HV-RNA positive, 1 OT-DNA positive and 1 co-infection with HV and OT were detected. From 15 mites co-infected with HV and OT, 2 strains of HV pathogen, 2 strains of OT pathogen were separated and 1 HV and OT pathogen in the same mite were separate.
CONCLUSION: The study demonstrates that co-infection of HV and OT did simultaneously exist in wild Leptotrombidium scutellare. This theory has some significance to the epidemic and precaution of HV and OT.

PMID: 19115620 [PubMed - indexed for MEDLINE]

594. J Commun Dis. 2008 Jun;40(2):159-60.

Typhus fever in Pondicherry.

Prabagaravarthanan R(1), Harish BN, Parija SC.

Author information:
(1)Dept of Microbiology, Jawaharlal Institute of Postgraduate Medical Education and Research, Pondicherry, India.

PMID: 19301703 [PubMed - indexed for MEDLINE]

595. Microbiol Immunol. 2003;47(10):727-33.

Electron microscopic observations of Orientia tsutsugamushi in salivary gland cells of naturally Infected Leptotrombidium pallidum larvae during feeding.

Kadosaka T(1), Kimura E.

Author information:
(1)Department of Parasitology, Aichi Medical University School of Medicine, Aichi 480-1195, Japan. kadosaka@aichi-med-u.ac.jp

We performed a detailed electron microscopic observation on the escaping process of Orientia tsutsugamushi from the salivary gland cells of naturally infected trombiculid larvae into the acinar lumen of the gland during feeding on mice. In unfed larvae, many O. tsutsugamushi were intermingled with secretory granules in the cytoplasm of the salivary gland cell. O. tsutsugamushi was neither found in the acinar lumen nor observed escaping from the apical surface of the gland cell. In contrast, in the larvae fed on mice, many O. tsutsugamushi were observable in the acinar lumen. They were enveloped with the host glandular cell membrane. In salivary gland cells, secretory granules changed the distribution and accumulated in the apical region. In such cells, the majority of O. tsutsugamushi were found at the base of the cell. Some O. tsutsugamushi were pushing the glandular cell membrane outward in various degrees, showing different stages of escape. These findings suggest that larval feeding induced O. tsutsugamushi escape from salivary gland cells, that the escape was by budding, during which O. tsutsugamushi were enveloped in the host cell membrane, and that O. tsutsugamushi would be injected into the mouse skin as a mixture with mite saliva. The study also revealed the presence of many small vesicles that had the same cell wall structure as O. tsutsugamushi in the cytoplasm of the salivary gland cell. Most of them seemed to be products from degenerated Orientia.

PMID: 14605439 [PubMed - indexed for MEDLINE]

596. Ugeskr Laeger. 2012 Mar 12;174(11):736.

[Picture of the month: tsutsugamushi disease].

[Article in Danish]

Mikkelsen CS(1).

Author information:
(1)Dermatovenereologisk Afdeling, Odense Universitetshospital, Denmark.
c.s.mikkelsen@hotmail.com

PMID: 22409900 [PubMed - indexed for MEDLINE]

597. Am J Med. 1988 Dec;85(6):799-805.

Imported rickettsial disease: clinical and epidemiologic features.

McDonald JC(1), MacLean JD, McDade JE.

Author information:
(1)McGill University Center for Tropical Disease, Montreal, Quebec, Canada.

PURPOSE AND METHODS: The rickettsioses continue to constitute major health problems in many parts of the world. With increasing international travel, recognition of rickettsial diseases by physicians is becoming more important. The clinical features of four cases of rickettsial disease imported into Canada over a five-year period are presented; two patients with tick typhus (Rickettsia conorii), one patient with scrub typhus (R. tsutsugamushi), and one patient with murine typhus (R. typhi). We also present the North American data over the past 10 years from the Centers for Disease Control (CDC) (Atlanta).
RESULTS: Since 1983 in the United States, three cases of imported scrub typhus, all after travel to India, were confirmed, as well as six cases of murine typhus after travel to southeast Asia. At the CDC, 67 imported cases of tick typhus have been confirmed by indirect fluorescent antibody test since 1976; most illnesses occurred after travel to Africa.
CONCLUSION: Rickettsial diseases are underrecognized by physicians, who should consider these diagnoses in travelers returning from endemic areas. Since effective treatment is available, prompt diagnosis and treatment are important. In all cases, specific serologic confirmation should be obtained.

PMID: 3195604 [PubMed - indexed for MEDLINE]

598. Infection. 1995 Mar-Apr;23(2):94-7.

Imported rickettsioses in German travelers.

Marschang A(1), Nothdurft HD, Kumlien S, von Sonnenburg F.

Author information:
(1)Abteilung für Infektions- und Tropenmedizin, Klinikum Innenstadt der Universität, München, Germany.

Twenty-two cases of rickettsiosis imported to Germany (13 men, nine women, average age 42 years) in a 5-year period were analyzed retrospectively regarding the travel histories, symptoms and clinical findings, laboratory features and course of the disease. The two primary rickettsial diseases were boutonneuse fever (18 patients) and scrub typhus (three patients). One patient had murine typhus. The main symptom was fever in 91% followed by headache (64%), myalgia (40%), arthralgia (50%) and diarrhea (36%). The most frequent clinical finding was lymphadenopathy in 65%. Eschar was detectable in 55% of patients with Rickettsia conori infection and in one patient with Rickettsia tsutsugamushi infection. All patients with R. tsutsugamushi infection as well as 33% of the patients with R. conori infection had a macular exanthema. One patient with scrub typhus had pleural and pericardial effusions. Seventy-three percent had an increased ESR. Three patients had leucocytosis, three increased transaminases and two normochromic anemia. The incubation period for R. conori infection was 5 to 28 days (average 14 days), for R. tsutsugamushi infection 7 to 21 days (average 16 days). Twenty-one patients were treated with tetracycline or doxycycline, one with erythromycin. All patients were cured. One patient had a relapse. Due to the fact that the symptoms are often not characteristic and that the routine laboratory findings are of only marginal help, the diagnosis of rickettsial diseases is often not easy. A detailed travel history sometimes gives an important hint for diagnosis.

PMID: 7622271 [PubMed - indexed for MEDLINE]

599. Arch Intern Med. 1991 Sep;151(9):1753-7.

Rickettsial meningitis and encephalitis.

Silpapojakul K(1), Ukkachoke C, Krisanapan S, Silpapojakul K.

Author information:
(1)Department of Medicine, Faculty of Medicine, Prince of Songkla University, Thailand.

Nine of 72 patients with scrub typhus and three of 137 with murine typhus presented with meningitis and/or encephalitis syndromes. Focal neurologic signs were rare, and cerebrospinal fluid profiles were similar to those of leptospirosis and viral and tuberculous meningitis. One patient had papilledema, and another had cerebellitis. Other major organ involvement (renal, liver, or lungs) occurred in five patients. One patient died and four spontaneously recovered, while the conditions of the rest responded well to either chloramphenicol or doxycycline. Scrub and murine typhus should be included in the differential diagnoses of aseptic meningitis and encephalitis in patients exposed to endemic areas, especially when accompanied by renal insufficiency and/or jaundice. They are treatable forms of virallike meningoencephalitis.

PMID: 1888241 [PubMed - indexed for MEDLINE]

600. Emerg Infect Dis. 2007 Jul;13(7):1105-7. doi: 10.3201/eid1307.050088.

Three rickettsioses, Darnley Island, Australia.

Unsworth NB(1), Stenos J, Faa AG, Graves SR.

Author information:
(1)The Australian Rickettsial Reference Laboratory, Geelong, Victoria, Australia.

We report 3 rickettsioses on Darnley Island, Australia, in the Torres Strait. In addition to previously described cases of Flinders Island spotted fever (Rickettsia honei strain "marmionii"), we describe 1 case of Queensland tick typhus (R. australis) and 2 cases of scrub typhus caused by a unique strain (Orientia tsutsugamushi).

DOI: 10.3201/eid1307.050088
PMCID: PMC2878210
PMID: 18214193 [PubMed - indexed for MEDLINE]

601. J Infect Dis. 1984 Mar;149(3):330-8.

Rickettsial diseases of the Far East: new perspectives.

Rapmund G.

The rickettsial disease of man found only in Asia is mite-borne (scrub) typhus, caused by Rickettsia tsutsugamushi. Unique to southern Japan is a little-known human mononucleosis-like disease caused by Rickettsia sennetsu. In 1981 and 1982, there was a remarkable resurgence in the number of reported cases of mite-borne typhus in Japan after some years of virtual absence. Recent studies of R sennetsu have resulted in its reclassification to the genus Ehrlichia, members of which until now have been exclusively pathogens of animals. The historical background of ecologic investigations, in Malaysia and elsewhere, of these two developments suggest directions for future research.

PMID: 6425420 [PubMed - indexed for MEDLINE]

602. Am J Trop Med Hyg. 1994 Aug;51(2):149-53.

Prevalence of antibodies to rickettsiae in the human population of suburban Bangkok.

Strickman D(1), Tanskul P, Eamsila C, Kelly DJ.

Author information:
(1)Department of Medical Entomology, Armed Forces Research Institute of Medical Sciences, Bangkok, Thailand.

Following a report of three cases of scrub typhus in suburban Bangkok, we performed a serosurvey in the patients' communities. Both IgG and IgM antibodies were measured in an indirect immunoperoxidase assay, using separate spots of antigen from Rickettsia tsutsugamushi (scrub typhus), R. typhi (murine typhus), and TT-118 spotted fever group rickettsiae. Of 215 people donating blood, antibody levels indicative of most recent exposure to R. tsutsugamushi were most prevalent (21%), followed by R. typhi (8%), and TT-118 (4%). Seroprevalence suggesting most recent exposure to R. tsutsugamushi varied by location (range 13-31%), gender (26% of females and 13% of males), age (61-80-year-old individuals were the highest, with a prevalence of 38%), and contact with orchards and orchid farms (29% of those with extensive contact, 38% of those with occasional contact, and 10% of those with no contact). These patterns indicated that exposure to R. tsutsugamushi was related to occupation and behavior, as has been observed in areas of rural transmission. Expansion of metropolitan Bangkok has created a situation in which people employed in agriculture live with people employed in the city. As a result, a serosurvey in suburban Bangkok reveals evidence of murine typhus, which is usually transmitted in urban areas, as well as scrub and tick typhus, which are usually transmitted in rural areas.

PMID: 8074248 [PubMed - indexed for MEDLINE]

603. Ind Med Gaz. 1949 Feb;84(2):63-8.

Epidemiological observations on XK or mite-borne typhus in Barrackpore, Bengal.

KRISHNAN KV, SMITH RO, et al.

PMID: 18130770 [PubMed - indexed for MEDLINE]

604. Kansenshogaku Zasshi. 2010 Jul;84(4):454-6.

[A case of classical tsutsugamushi disease confirmed after an interval of 15 years in Akita Prefecture, Japan].

[Article in Japanese]

Sato H(1), Kokusho Y, Shibata C, Saito H, Saito S, Fujita H, Suto T.

Author information:
(1)Public Health Division Microbiology Section, Akita Prefectural Research Center for Public Health and Environment.

We report the confirmation of classical tsutsugamushi disease in August 2008. A 17-year-old woman seen for fever and eschar on the back reported having been bitten by an insect nine days earlier while fishing on the Omonogawa river. The suspected culprit was Leptotrombidium akamushi. During convalescence serum IgM and IgG antibody titers rose significantly against the Kato serotype antigen in indirect immunoperoxidase staining. Epidemiology, clinical symptoms and the antibodies detected suggested classical tsutsugamushi disease infection. Such disease transmitted by L. akamushi have not been reported since 1993 in Akita Prefecture. The public should thus be informed about Orientia tsutsugamushi prevention, in case such disease re-care in this area in the future.

PMID: 20715556 [PubMed - indexed for MEDLINE]

605. PLoS Negl Trop Dis. 2014 Jan 30;8(1):e2533. doi: 10.1371/journal.pntd.0002533. eCollection 2014.

Estimating the burden of Japanese encephalitis virus and other encephalitides in countries of the mekong region.

Tarantola A(1), Goutard F(2), Newton P(3), de Lamballerie X(4), Lortholary O(5), Cappelle J(2), Buchy P(1).

Author information:
(1)Institut Pasteur du Cambodge, Phnom Penh, Cambodia. (2)Institut Pasteur du Cambodge, Phnom Penh, Cambodia ; Centre de coopération internationale en recherche agronomique pour le développement (CIRAD), Département ES, Unité AGIRs, Montpellier, France. (3)Lao-Oxford-Mahosot Hospital-Wellcome Trust Research Unit, Microbiology Laboratory, Mahosot Hospital, Vientiane, Lao PDR and Centre for Tropical Medicine, Nuffield Department of Medicine, Churchill Hospital, University of Oxford, Oxford, United Kingdom. (4)Aix Marseille University, IRD French Institute of Research for Development, EHESP French School of Public Health, UMR_D 190 "Emergence des Pathologies Virales", Marseille, France. (5)Université René Descartes, Hôpital Necker-Enfants malades, Centre

d'Infectiologie Necker Pasteur, IHU Imagine, Labex IBEID, Paris, France.

Diverse aetiologies of viral and bacterial encephalitis are widely recognized as significant yet neglected public health issues in the Mekong region. A robust analysis of the corresponding health burden is lacking. We retrieved 75 articles on encephalitis in the region published in English or in French from 1965 through 2011. Review of available data demonstrated that they are sparse and often derived from hospital-based studies with significant recruitment bias. Almost half (35 of 75) of articles were on Japanese encephalitis virus (JEV) alone or associated with dengue. In the Western Pacific region the WHO reported 30,000-50,000 annual JEV cases (15,000 deaths) between 1966 and 1996 and 4,633 cases (200 deaths) in 2008, a decline likely related to the introduction of JEV vaccination in China, Vietnam, or Thailand since the 1980s. Data on dengue, scrub typhus and rabies encephalitis, among other aetiologies, are also reviewed and discussed. Countries of the Mekong region are undergoing profound demographic, economic and ecological change. As the epidemiological aspects of Japanese encephalitis (JE) are transformed by vaccination in some countries, highly integrated expert collaborative research and objective data are needed to identify and prioritize the human health, animal health and economic burden due to JE and other pathogens associated with encephalitides.

DOI: 10.1371/journal.pntd.0002533
PMCID: PMC3907313
PMID: 24498443 [PubMed - indexed for MEDLINE]

606. Infect Immun. 1972 May;5(5):745-9.

Zoonotic infections in military scout and tracker dogs in Vietnam.

Alexander AD, Binn LN, Elisberg B, Husted P, Huxsoll DL, Marshall JD Jr, Needy CF, White AD.

Significant levels of antibodies indicative of a variety of zoonotic infections were demonstrated in sera collected from 64 U.S. military scout and tracker dogs after service in the Republic of Vietnam (RVN). Scrub typhus antibodies were found in 45% of the specimens, melioidosis in 19%, group B arbovirus in 49%, and leptospirosis in 62%. Only 38% of the seropositive reactions for leptospirosis could be related to overseas infections. Tests were conducted on paired sera obtained from 32 of the dogs before service in RVN. Significant increases in titer were demonstrated with scrub typhus, melioidosis, leptospirosis, plague, group B arbovirus, and Rickettsia canada antigens. After 2 to 6 months of service in RVN, 8 of 19 dogs developed antibody titers to at least one of four zoonotic diseases. Only 3 of 45 dogs with 7 or more months of service failed to develop antibodies to one or more of the agents. The serological findings pose questions on the potential epidemiological importance and veterinary significance of scrub typhus, group B arbovirus, leptospirosis, and melioidosis infections in dogs.

PMCID: PMC422434
PMID: 4564881 [PubMed - indexed for MEDLINE]

607. Trop Doct. 2011 Apr;41(2):111-2. doi: 10.1258/td.2010.100303. Epub 2010 Dec 13.

Rickettsial infection with hemophagocytosis.

Jayakrishnan MP(1), Veny J, Feroze M.

Author information:
(1)Department of Paediatrics, Institute of Maternal and Child Health, Calicut Medical College, Calicut 8, Kerala 673 008, India. mp.jayakrishnan@gmail.com

A five-year-old girl admitted with scrub typhus developed multiple organ dysfunction associated with hyperferritinaemia, hypofibrinogenaemia and hyperlipidaemia. Bone marrow aspiration studies confirmed haemophagocytic lymphohistiocytosis (HLH). HLH is a syndrome characterized by the uncontrolled activation and proliferation of macrophages and T-cells and can occur together with infections, connective tissue disorders, malignancies and genetic disorders.

DOI: 10.1258/td.2010.100303
PMID: 21149571 [PubMed - indexed for MEDLINE]

608. Enferm Infecc Microbiol Clin. 2005 Mar;23(3):163-72.

[Rickettsioses].

[Article in Spanish]

Bernabeu-Wittel M(1), Segura-Porta F.

Author information:
(1)Servicio de Medicina Interna, Hospitales Universitarios Virgen del Rocío, Sevilla, Spain. wittel@cica.es

Species of the genus Rickettsia are small, obligate intracelular, gramnegative bacteria, many of which are considered nowadays a paradigm of emergent pathogens. With the exception of R. prowazekii, they are maintained in the natural environment through a cycle involving different hosts (mainly mammals), and arthropod vectors (in general ticks, and fleas); humans are affected only by incidental transmission due to arthropod bites. The common pathogenesis of these diseases lie on the predominantly infection of endotelial cells, that determines the development of multisistemic small vessel vasculitis, which may affect lungs (interstitial pneumonitis), heart (miopericarditis), skin (rash), central nervous system (meningoencephalitis), as well as liver, and kidneys. They are classified in two groups: spotted-fever group, and typhus group rickettsia. In Spain the most prevalent rickettsioses of both groups are mediterranean spotted fever (caused by R. conorii), and murine typhus (caused by R. typhi), respectively. This review focuses mainly in these two diseases, and also in other rickettsioses of interest due to their recently emergence or reemergence (R. slovaca, R. africae, R. prowazekii, R. felis), or to their high incidence in other areas (R. rickettsii, Orientia tsutsugamushi).

PMID: 15757589 [PubMed - indexed for MEDLINE]

609. Zhonghua Liu Xing Bing Xue Za Zhi. 2000 Jun;21(3):212-5.

[The recognition of the epidemic area of tsutsugamushi disease on Nan Peng Lie Island in China and the strategy of prevention].

[Article in Chinese]

Wang S(1), Jiang P, Huang J, Peng G, Zeng N, Liu J, Zhu S, Wang Z, Liang L, You X, Xie Z, Tang Z, Wu Z, Huang J, Yuan W, Zhao X, Huang T.

Author information:
(1)Military Medical Institute, Guangzhou Command PLA, Guangzhou 510507, China.

OBJECTIVE: To investigate the region for the epidemic area of tsutsugamushi disease.
METHODS: Epidemiological studies, Orientia tsutsugamushi isolation, and preventive measures were used.
RESULTS: The region belonged to epidemic area of south subtropical zone. The main host was found Rattus norvegicu. The main biological vector was Leptotrombidium deliens. The seasonal trends of the quantity of Rattus norvegicu and Leptotrombidium deliens were consistent with the incidence change of the disease in the region. Orientia tsutsugamushi has been isolated from Rattus norvegicu and Leptotrombidium deliens. Data showed that Orientia tsutsugamushi isolated strains mainly belongs to Karp. Sero-epidemiological data showed that antibodies of Orientia tsutsugamushi are 100.0% positive in the residents of the region and 4.0% in army men. After taking comprehensive preventive measures, the incidence was descending.
CONCLUSION: Nan Peng Lie island was proved an epidemic area of tsutsugamushi disease.

PMID: 11860788 [PubMed - indexed for MEDLINE]

610. PLoS One. 2014 Feb 27;9(2):e89896. doi: 10.1371/journal.pone.0089896. eCollection 2014.

Co-circulation of multiple hemorrhagic fever diseases with distinct clinical characteristics in Dandong, China.

Chen ZH(1), Qin XC(2), Song R(1), Shen Y(3), Chen XP(2), Wang W(2), Zhao YX(3), Zhang JS(2), He JR(2), Li MH(2), Zhao XH(3), Liu DW(3), Fu XK(1), Tian D(1), Li XW(1), Xu J(2), Plyusnin A(4), Holmes EC(5), Zhang YZ(2).

Author information:
(1)Department of Infectious Diseases, Beijing Ditan Hospital, Capital Medical University, Beijing, China. (2)State Key Laboratory for Infectious Disease Prevention and Control, Collaborative Innovation Center for Diagnosis and Treatment of Infectious Diseases, Department of Zoonoses, National Institute for Communicable Disease Control and Prevention, Chinese Center for Disease Control and Prevention, Beijing, China. (3)Department of Infectious Diseases, Dandong Infectious Hospital, Dandong, Liaoning Province, China. (4)State Key Laboratory for Infectious Disease Prevention and Control, Collaborative Innovation Center for Diagnosis and Treatment of Infectious Diseases, Department of Zoonoses,

National Institute for Communicable Disease Control and Prevention, Chinese
Center for Disease Control and Prevention, Beijing, China ; Department of
Virology, Haartman Institute, University of Helsinki, Finland. (5)Marie Bashir
Institute for Infectious Diseases and Biosecurity, School of Biological Sciences
and Sydney Medical School, The University of Sydney, Sydney, Australia.

Hemorrhagic fevers (HF) caused by viruses and bacteria are a major public health problem in China and characterized by variable clinical manifestations, such that it is often difficult to achieve accurate diagnosis and treatment. The causes of HF in 85 patients admitted to Dandong hospital, China, between 2011-2012 were determined by serological and PCR tests. Of these, 34 patients were diagnosed with Huaiyangshan hemorrhagic fever (HYSHF), 34 with Hemorrhagic Fever with Renal Syndrome (HFRS), one with murine typhus, and one with scrub typhus. Etiologic agents could not be determined in the 15 remaining patients. Phylogenetic analyses of recovered bacterial and viral sequences revealed that the causative infectious agents were closely related to those described in other geographical regions. As these diseases have no distinctive clinical features in their early stage, only 13 patients were initially accurately diagnosed. The distinctive clinical features of HFRS and HYSHF developed during disease progression. Enlarged lymph nodes, cough, sputum, and diarrhea were more common in HYSHF patients, while more HFRS cases presented with headache, sore throat, oliguria, percussion pain kidney area, and petechiae. Additionally, HYSHF patients displayed significantly lower levels of white blood cells (WBC), higher levels of creations kinase (CK) and alanine aminotransferase (ALT), while HFRS patients presented with an elevation of blood urea nitrogen (BUN) and creatinine (CREA). These clinical features will assist in the accurate diagnosis of both HYSHF and HFRS. Overall, our data reveal the complexity of pathogens causing HFs in a single Chinese hospital, and highlight the need for accurate early diagnosis and a better understanding of their distinctive clinical features.

DOI: 10.1371/journal.pone.0089896
PMCID: PMC3937409
PMID: 24587107 [PubMed - indexed for MEDLINE]

611. Indian J Med Res. 2007 Aug;126(2):101-3.

Spotted fevers & typhus fever in Tamil Nadu.

Batra HV(1).

Author information:
(1)Defence Food Research Laboratory, Siddharth Nagar, Mysore 570011, India.
h_v_batra@rediffmail.com

Comment on
Indian J Med Res. 2007 Aug;126(2):128-30.

PMID: 17932432 [PubMed - indexed for MEDLINE]

612. PLoS One. 2012;7(9):e44269. doi: 10.1371/journal.pone.0044269. Epub 2012 Sep 6.

Mapping the aetiology of non-malarial febrile illness in Southeast Asia through a systematic review--terra incognita impairing treatment policies.

Acestor N(1), Cooksey R, Newton PN, Ménard D, Guerin PJ, Nakagawa J, Christophel E, González IJ, Bell D.

Author information:
(1)Malaria/Acute Febrile Syndrome Programme, Foundation for Innovative New Diagnostics, Geneva, Switzerland.

BACKGROUND: An increasing use of point of care diagnostic tests that exclude malaria, coupled with a declining malaria burden in many endemic countries, is highlighting the lack of ability of many health systems to manage other causes of febrile disease. A lack of knowledge of distribution of these pathogens, and a lack of screening and point-of-care diagnostics to identify them, prevents effective management of these generally treatable contributors to disease burden. While prospective data collection is vital, an untapped body of knowledge already exists in the published health literature.
METHODS: Focusing on the Mekong region of Southeast Asia, published data from 1986 to 2011 was screened to for frequency of isolation of pathogens implicated in aetiology of non-malarial febrile illness. Eligibility criteria included English-language peer-reviewed studies recording major pathogens for which specific management is likely to be warranted. Of 1,252 identified papers, 146 met inclusion criteria and were analyzed and data mapped.
RESULTS: Data tended to be clustered around specific areas where research institutions operate, and where resources to conduct studies are greater. The most frequently reported pathogen was dengue virus (n = 70), followed by Orientia tsutsugamushi and Rickettsia species (scrub typhus/murine typhus/spotted fever group n = 58), Leptospira spp. (n = 35), Salmonella enterica serovar Typhi and Paratyphi (enteric fever n = 24), Burkholderia pseudomallei (melioidosis n = 14), and Japanese encephalitis virus (n = 18). Wide tracts with very little published data on aetiology of fever are apparent.
DISCUSSION AND CONCLUSIONS: This mapping demonstrates a very heterogeneous distribution of information on the causes of fever in the Mekong countries. Further directed data collection to address gaps in the evidence-base, and expansion to a global database of pathogen distribution, is readily achievable, and would help define wider priorities for research and development to improve syndromic management of fever, prioritize diagnostic development, and guide empirical therapy.

DOI: 10.1371/journal.pone.0044269
PMCID: PMC3435412
PMID: 22970193 [PubMed - indexed for MEDLINE]

613. FEMS Microbiol Lett. 2009 Aug;297(1):95-100. doi: 10.1111/j.1574-6968.2009.01663.x. Epub 2009 May 27.

Novel polysaccharide antigen of Orientia tsutsugamushi revealed by a monoclonal antibody.

Lee SM(1), Kim MK, Kim MJ, Kang JS.

Author information:

(1)Department of Microbiology and Center for Advanced Medical Education by BK21
Project, Inha University School of Medicine, Incheon, Korea.

Orientia tsutsugamushi, the causative agent of scrub typhus, is an obligate intracellular bacterium that replicates in the cytosol of host cells. Although several protein antigens have been characterized and cloned, little information exists regarding the polysaccharide antigen of this bacterium. In this study, we identified and characterized a novel antigen defined by a monoclonal antibody (MAb), NT19, against O. tsutsugamushi. Immunofluorescence microscopic studies showed that the NT19 antigen is released from the bacteria in the cytosol of host cells forming aggregates with bacteria. Immunoblot analysis showed that MAb NT19 recognized a strong band with a molecular mass of 20 kDa that was resistant to proteinase K digestion and sensitive to periodate oxidation, suggesting that the NT19 antigen is a polysaccharide. The function of this polysaccharide is not known, but considering its distribution within a bacterial microcolony, it is suspected to be involved in forming a biofilm-like structure within host cells.

DOI: 10.1111/j.1574-6968.2009.01663.x
PMID: 19566581 [PubMed - indexed for MEDLINE]

614. Bull World Health Organ. 1964;31:411-6.

MITES (FAMILY TROMBICULIDAE) PARASITIZING BIRDS MIGRATING FROM AFRICA TO EUROPE.

VARMA MG.

The mechanisms of dissemination of arthropod-borne human and animal pathogens are of considerable interest to the epidemiologist, veterinarian and biologist. Birds which are hosts to such pathogens and their arthropod vectors could transport them over long distances during their spring and autumn migratory flights.In April 1961, birds migrating from Africa to Europe were collected in south-western Spain and examined for ectoparasites and antibodies to arboviruses. Fully engorged larvae of two species of trombiculid mites unknown in Europe (genera Neoschoengastia and Blankaartia) but found in Africa were collected from two of the migrating birds (redstart and little bittern), suggesting that the birds were carrying the mites from Africa to Europe.Trombiculid mites are the proven vectors of scrub typhus; they have also been implicated in the transmission of human haemorrhagic nephroso-nephritis. The finding of the mite larvae on migrating birds is therefore of some epidemiological interest and underlines the importance of obtaining more data on the dispersal of trombiculids by migrating birds.

PMCID: PMC2555110
PMID: 14267750 [PubMed - indexed for MEDLINE]

615. Nihon Naika Gakkai Zasshi. 1985 Sep;74(9):1218-22.

[Tsutsugamushi disease, in special relation to the new type disease].

[Article in Japanese]

Tachibana N.

PMID: 3935745 [PubMed - indexed for MEDLINE]

616. Jpn J Exp Med. 1990 Dec;60(6):325-35.

Aggregated distribution of infective spots composed of Leptotrombidium pallidum, highly prevalent with Rickettsia tsutsugamushi, demonstrated by sentinel voles, Microtus montebelli, on the ground.

Takahashi M(1), Murata M, Machida K, Hori E, Kawamura A Jr, Tanaka H.

Author information:
(1)Department of Medical Zoology, Saitama Medical School, Japan.

In the epidemiological surveys on scrub typhus at Chichibu City near Tokyo, an area, 350 m by 35 m, in the Hitsujiyama Park was found to be heavily infested with Rickettsia tsutsugamushi (Rt). Distribution of trombiculid mites and their infection rates in this area were studied using sentinel animals, 48 Microtus montebelli and 10 ddY mice. The surveys were conducted 6 times in the autumn in 1985 and 1986. At the first 2 surveys, 16 animals were placed at random, and 4 restricted areas were identified as highly populated with trombiculid mites. For the detailed survey, each of 4 areas was divided into 2 m x 2 m quadrats to settle a sentinel animal to each. A total of mites collected by all sentinel animals was 331 Leptotrombidium pallidum, 175 L. fuji and 16 Gahrliepia saduski. Almost all mites were collected by M. montebelli, except only 1 L. fuji in 10 mice. From a M. montebelli in a quadrat, 157 L. pallidum were recovered, whereas the number/vole was 0 to 24 in the others. L. fuji was also highly aggregated at 2 sites. Rt was detected from individual mites by avidin-biotin immunofluorescence or isolated by the mouse passage from individual or pooled mites. Only Karp strain of Rt was detected or isolated from L. pallidum at a ratio of 31/286 (10.8%). No Rt was found from L. fuji or G. saduski. The infection rate in L. pallidum was especially high in 3 voles at ratios of 6/11, 2/5 and 3/5, respectively. Out of 14 sentinel M. montebelli with infected L. pallidum, 12 (85.7%) were infected with Rt. It was concluded that L. pallidum was distributed in aggregated clusters to form the mite islands and was infected heavily at the specific sites to make the infective spots.

PMID: 2128945 [PubMed - indexed for MEDLINE]

617. J Med Assoc Thai. 1987 Feb;70(2):55-62.

Murine typhus in southern Thailand.

Silpapojakul K, Woodtayagone J, Lekakula A, Vimuktalaba A, Krisanapan S.

PMID: 3585210 [PubMed - indexed for MEDLINE]

618. Am J Trop Med Hyg. 1990 Nov;43(5):520-6.

Murine typhus identified as a major cause of febrile illness in a camp for displaced Khmers in Thailand.

Duffy PE(1), Le Guillouzic H, Gass RF, Innis BL.

Author information:
(1)Armed Forces Research Institute of Medical Sciences, Bangkok, Thailand.

Scrub and murine typhus have been identified as causes of illness among the 238,000 displaced Khmer people residing in temporary settlements on the Thai side of the Thai-Cambodian border. Still, the true extent of the problem and the relative frequency of infection with scrub typhus as compared to murine typhus are unknown. We evaluated consecutive patients with unexplained pyrexia (documented fever, no exclusionary diagnosis, and constitutional symptoms) in 1 temporary settlement over 1 month. Laboratory studies included culture of blood and assay of paired sera for rickettsial IgM and IgG antibody, for dengue IgM and IgG antibody, and for leptospiral IgM and IgG antibody. Among 37 patients (27 adults and 10 children), 28 (75%) had a rickettsiosis (26 cases of murine typhus and 2 cases of scrub typhus). No case of enteric fever, dengue, or leptospirosis was diagnosed. The illnesses of 9 patients were not identified. Signs and symptoms did not distinguish confirmed rickettsial infections from undiagnosed illnesses. The 1 month attack rate of rickettsial infection was 29/100,000 for children and 185/100,000 for adults. Murine typhus was a major cause of febrile illness in this settlement.

PMID: 2122749 [PubMed - indexed for MEDLINE]

619. Jpn J Exp Med. 1988 Aug;58(4):169-84.

Rickettsiosis in Japan.

Kawamura A Jr(1), Tanaka H.

Author information:
(1)Department of Parasitology, University of Tokyo, Japan.

The rickettsial diseases of man in Japan include Tsutsugamushi disease (scrub typhus), murine typhus, which occurs sporadically, and one of spotted fever group diseases, recognized as a new entity since 1984. Starting from 1976, there has been a remarkable resurgence in the number of reported cases of Tsutsugamushi disease in Japan after several years of virtual absence, and endemics are still continuing after reaching a prominent outbreak in 1984. Its yearly incidences from 1982 to 1986 were 538, 749, 971, 890 and 738, respectively. This resurgence is most likely related to an increase of vector mite colonies that carry Rickettsia tsutsugamushi. However, it cannot be explained as to how these foci of vector mites developed. The existence of spotted fever group disease had not been recognized until quite recently. This rickettsiosis was first identified in Tokushima and Kochi Prefectures in 1984. To date, the estimated total number of sero-positive cases is over 45 cases. The causative rickettsiae were isolated from the blood of 5 patients using cell culture methods and the identification of these rickettsiae is now being carried out.

PMID: 3210342 [PubMed - indexed for MEDLINE]

620. Pediatr Infect Dis J. 2008 Jun;27(6):569-70. doi: 10.1097/INF.0b013e318168db08.

Importance of a thorough examination.

Pau WS(1), Tan KK.

Author information:
(1)Paediatric Department, Hospital Tuanku Jaafar Seremban, Seremban, Malaysia.
drwilsonpau@yahoo.co.uk

Scrub typhus is a common cause of febrile illness among children from rural regions in tropical countries. We described 2 cases of scrub typhus with an eschar localized in the genitalia that was missed during the routine medical examination of a febrile child.

DOI: 10.1097/INF.0b013e318168db08
PMID: 18449061 [PubMed - indexed for MEDLINE]

621. Uirusu. 1986 Jun;36(1):55-70.

[Present status of Tsutsugamushi disease, especially its epidemiology, clinical features and etiological diagnosis in Japan].

[Article in Japanese]

Suto T.

PMID: 3095984 [PubMed - indexed for MEDLINE]

622. BMC Infect Dis. 2013 Jul 30;13:355. doi: 10.1186/1471-2334-13-355.

Fever in the tropics: aetiology and case-fatality - a prospective observational study in a tertiary care hospital in South India.

Abrahamsen SK(1), Haugen CN, Rupali P, Mathai D, Langeland N, Eide GE, Mørch K.

Author information:
(1)Department of Medicine, National Centre for Tropical Infectious Diseases, Haukeland University Hospital, Bergen, Norway.

BACKGROUND: The objective of this study was to describe aetiology and case fatality of fever among inpatients in a tertiary care hospital in South India.
METHODS: This was an observational, prospective study conducted in a tertiary care hospital in Vellore, Tamil Nadu, India. Between July 2nd 2007 and August 2nd in 2007, adult patients admitted to the hospital with temperature ≥ 38.0°C were included consecutively and followed during the hospitalisation period.

Demographic and clinical data were collected and analysed for each patient. Associations were sought between death and various clinical and demographic variables.
RESULTS: One hundred patients were included, 61 male and 39 female. Mean age was 37.5 (range: 16 to 84) years. Mean fever duration was 5.4 (range: 0.1 to 42.9) weeks.The following infectious aetiologies were recorded: tuberculosis (19%), lower respiratory infection (11%) including three with sepsis, urinary tract infection (10%) including three with E. coli sepsis, Plasmodium falciparum malaria (5%) including three patients with mixed P. vivax infection, scrub typhus (5%), typhoid fever (4%), cryptococcal meningitis (4%) including three HIV positive patients, endocarditis (3%) including two patients with Staphylococcus aureus sepsis, spleen abscess (2%), amoebic liver abscess (2%), sepsis undefined focus (1%), HIV infection (1%), hepatitis B (1%), rubella (1%), peritonitis (1%) and cholecystitis (1%).Non-infectious causes of fever were diagnosed in 15%, including systemic lupus erythematosus in four and malignancy in six patients. Cause of fever remained unknown in 13%.Case fatality during hospitalisation was 7% (7/100). Six of those who died were male. Five fatalities had bacterial sepsis, one spleen abscess and malignancy, and one had lymphomalignant disorder.Diabetes and increasing age were significant risk factors for fatal outcome in unadjusted analyses, but only increasing age was a risk factor for death in adjusted analysis.
CONCLUSIONS: A high number of tuberculosis and bacterial infections and a high case fatality rate from sepsis were found in this cohort, underlining the importance of microbiological diagnostics and targeted antimicrobial treatment in the management of fever. P. falciparum was identified in all malaria cases, and this rapidly fatal infection should be considered in patients with acute undifferentiated fever in India.

DOI: 10.1186/1471-2334-13-355
PMCID: PMC3750507
PMID: 23899336 [PubMed - indexed for MEDLINE]

623. Ann Trop Med Parasitol. 2006 Jun;100(4):363-70.

Causes of acute, undifferentiated, febrile illness in rural Thailand: results of a prospective observational study.

Suttinont C(1), Losuwanaluk K, Niwatayakul K, Hoontrakul S, Intaranongpai W, Silpasakorn S, Suwancharoen D, Panlar P, Saisongkorh W, Rolain JM, Raoult D, Suputtamongkol Y.

Author information:
(1)Department of Medicine, Maharat Nakhon Ratchasima Hospital, Amphur Mueng, Nakhon Ratchasima Province, 30000, Thailand.

The adult patients who, between July 2001 and June 2002, presented at any of five hospitals in Thailand with acute febrile illness in the absence of an obvious focus of infection were prospectively investigated. Blood samples were taken from all of the patients and checked for aerobic bacteria and leptospires by culture. In addition, at least two samples of serum were collected at different times (on admission and 2-4 weeks post-discharge) from each patient and tested, in serological tests, for evidence of leptospirosis, rickettsioses, dengue and influenza. The 845 patients investigated, of whom 661 were male, had a median age

of 38 years and a median duration of fever, on presentation, of 3.5 days. Most (76.5%) were agricultural workers and most (68.3%) had the cause of their fever identified, as leptospirosis (36.9%), scrub typhus (19.9%), dengue infection or influenza (10.7%), murine typhus (2.8%), Rickettsia helvetica infection (1.3%), Q fever (1%), or other bacterial infection (1.2%). The serological results indicated that 103 (12.2%) and nine (1%) of the patients may have had double and triple infections, respectively. Leptospirosis and rickettsioses, especially scrub typhus, were thus found to be major causes of acute, undifferentiated fever in Thai agricultural workers.

DOI: 10.1179/136485906X112158
PMID: 16762116 [PubMed - indexed for MEDLINE]

624. J Clin Microbiol. 1992 Nov;30(11):2842-6.

Epidemiology of Tsutsugamushi disease in relation to the serotypes of Rickettsia tsutsugamushi isolated from patients, field mice, and unfed chiggers on the eastern slope of Mount Fuji, Shizuoka Prefecture, Japan.

Kawamori F(1), Akiyama M, Sugieda M, Kanda T, Akahane S, Uchikawa K, Yamada Y, Kumada N, Furuya Y, Yoshida Y, et al.

Author information:
(1)Department of Microbiology, Shizuoka Prefectural Institute of Public Health and Environmental Science, Shizuoka, Japan.

A total of 59 strains of Rickettsia tsutsugamushi were isolated from patients (24 isolates), Apodemus speciosus mice (30 isolates), and unfed larvae of Leptotrombidium scutellare (2 isolates) and Leptotrombidium pallidum (3 isolates) in the Gotenba-Oyama District, Shizuoka Prefecture, Japan. All these isolates were classified into the three serotypes Karp, Kawasaki, and Kuroki based on reactivity with strain-specific monoclonal antibodies. Kawasaki- and Karp-type rickettsiae were isolated from L. scutellare and L. pallidum, respectively, and the geographic distribution of patients and rodents infected with these two types of rickettsiae coincided with the areas densely populated by the respective chiggers. From these results, we conclude that Kawasaki-type rickettsiae are transmitted by L. scutellare and Karp-type ones are transmitted by L. pallidum. Kawasaki-type rickettsial infections were prevalent in early autumn, and Karp-type infections showed a peak of occurrence in the late autumn, reflecting the seasonal fluctuations of L. scutellare and L. pallidum. Isolates of Kuroki-type rickettsiae were obtained only from four patients in October and November, and the relationship between this type of rickettsia and its vector species could not be fully defined.

PMCID: PMC270539
PMID: 1452653 [PubMed - indexed for MEDLINE]

625. Am J Trop Med Hyg. 1999 May;60(5):786-9.

Rickettsia serosurvey in Kimberley, Western Australia.

Graves S(1), Wang L, Nack Z, Jones S.

Author information:
(1)Australian Rickettsial Reference Laboratory, The Geelong Hospital, Victoria.

To determine if antibodies to rickettsiae (scrub typhus, spotted fever, and typhus group rickettsiae) occur among persons living in the Kimberley (northern tropical) region of Western Australia, 920 sera collected in a non-random manner in 1996 from patients in Kununurra, Broome, Fitzroy Crossing, Wyndham, Derby, and Halls Creek were tested by micro-immunofluorescence for antibodies to a panel of rickettsial antigens. Of 920 sera examined, 52 (5.6%) were positive for antibodies to one or more of the three groups of rickettsial microorganisms. The largest group of sera (24; 2.6%) were positive for scrub typhus (Orientia tsutsugamushi). Eleven other sera (1.2%) were positive for scrub typhus and spotted fever group rickettsiae and four (0.4%) were positive for scrub typhus, spotted fever group, and typhus group rickettsiae. In addition 13 sera (1.4%) were positive only for spotted fever group rickettsiae. In this study, only titers > or = 1:256 were considered significant. Thus, there is serologic evidence for scrub typhus and spotted fever group rickettsial infections in the Kimberley region of Western Australia. Because of the method of serum collection, it is not possible to determine the prevalence of seropositivity, but the data support the need for a proper epidemiologic study of rickettsial diseases in this region of Australia.

PMID: 10344653 [PubMed - indexed for MEDLINE]

626. Kansenshogaku Zasshi. 1989 Feb;63(2):109-17.

[Antigenic types of Rickettsia tsutsugamushi isolated from patients with tsutsugamushi fever and their distribution in Miyazaki Prefecture].

[Article in Japanese]

Yamamoto S, Kawabata N, Ooura K, Murata M, Minamishima Y.

Rickettsia tsutsugamushi (Rt) isolated from patients with tsutsugamushi fever were examined for their antigenicity. This was done by indirect immunofluorescence (IIF) with guinea pig antisera against three standard strains (Karp, Kato and Gilliam) and two local strains (Kawasaki and Kuroki) isolated in 1981, and with mouse monoclonal antibodies against the three standard strains. In the meantime, antibodies in sera from 317 out of 442 patients registered during 1985 to 1988 were titrated by IIF with those five Rt strains. 1) Local isolates, Kawasaki and Kuroki strains, reacted most effectively with the homologous antiserum, respectively, showing four fold lower IIF titers against the heterologous antisera. 2) Kawasaki strain reacted with none of the monoclonal antibodies, whereas Kuroki strain showed a slight reaction with anti-Karp and anti-Kato, but not anti-Gilliam, monoclonal antibodies. 3) Seventeen out of 27 strains isolated in 1985 resembled the Kawasaki strain in their reaction patterns with the antisera and monoclonal antibodies, and the other 10 strains showed reactivity similar to the Kuroki strain. 4) Sera of 233 (74%) out of 317 patients showed the highest antibody titers against the Kawasaki strain and 69 (22%) of 317 against the Kuroki strain. It is thus evident that Kawasaki and Kuroki

strains are antigenically different from the standard strains, and Kawasaki and Kuroki strains also differ from each other. It is suggested that two antigenic types (Kawasaki and Kuroki) of Rt were distributed in Miyazaki Prefecture, Rt of the Kawasaki type slightly dominates Rt of the Kuroki type, and recent tsutsugamushi fever has been caused by either one or the other type of Rt.

PMID: 2501427 [PubMed - indexed for MEDLINE]

627. Zhonghua Liu Xing Bing Xue Za Zhi. 2007 Oct;28(10):996-9.

[Study on the characteristics of Tsutsugamushi disease in the epidemic areas of south islands in China].

[Article in Chinese]

Wang SS(1), Huang JL, Su JX, Xi YZ, Wang Y, Li MM.

Author information:
(1)Center for Disease Control and Prevention of Guangzhou Command People's Liberation Army, Guangzhou 510507, China.

OBJECTIVE: To study the increasing incidence and the characteristics of Tsutsugamushi disease in the areas of Nan Peng Lie islands, Nan Ao island, Wan Shan archipelago, Nao Zhou island and Lei Zhou peninsula, located in the southern part of China and to develop strategies for preventive measures.
METHODS: Both epidemiological investigation, isolation and gene identification of Orientia tsutsugamushi, as well as pilot preventive measures were carried out.
RESULTS: These islands belonged to the epidemic area of south subtropical zone of Tsutsugamushi disease. The main host was Rattus norvegicu and the overall rates of infection on Orientia tsutsugamushi were 22.78%-33.75%. The main biological vector was Leptotrombidium (Leptotrombidium) deliens and the rates of infection on Orientia tsutsugamushi were 40.00%-75.00%. 25 strains of Orientia tsutsugamushi had been isolated from Rattus norvegicu and Leptotrombidium (Leptotrombidium) deliens. Results showed that the isolated strains of Orientia tsutsugamushi were 15 Karp, 8 Kato, 2 Yonchon. Results from serological studies showed that the positive rate of anti-Orientia tsutsugamushi antibodies was high, in both residents and soldiers stationed in these islands. On these islands, rats and biological vectors were killed. Results showed that these measures had positive impact in reducing the incidence.
CONCLUSION: Islands from the southern part of the country belonged to the epidemic area of Tsutsugamushi disease. People visiting this areas should be under protection.

PMID: 18399148 [PubMed - indexed for MEDLINE]

628. Med J Aust. 1953 Jul 25;2(4):121-9.

Fevers of the Mackay district, Queensland.

DERRICK EH, BERRY AH, TONGE JI, BROWN HE.

PMID: 13086055 [PubMed - indexed for MEDLINE]

629. BMC Infect Dis. 2011 Nov 25;11:328. doi: 10.1186/1471-2334-11-328.

Seroepidemiology of rickettsioses in Sri Lanka: a patient based study.

Liyanapathirana VC(1), Thevanesam V.

Author information:
(1)Department of Microbiology, Faculty of Medicine, University of Peradeniya, Peradeniya, Sri Lanka. veranjacl@yahoo.com

BACKGROUND: Rickettsioses are emerging infections in Sri Lanka as shown by the increase in the number of clinically diagnosed rickettsial patients being reported to the Epidemiology Unit, Sri Lanka. However, mapping the disease for the whole island with laboratory confirmed cases has not been previously carried out.
METHODS: 615 samples received from 23 hospital representing 8 provinces were tested using ELISA or IFA methods and clinical data was collected using a validated questionnaire.
RESULTS: Rash was found among more spotted fever seropositive patients than scrub typhus seropositive patients while the opposite was true for the presence of eschar. Spotted fever and scrub typhus was found in a geographically restricted manner. Consistent temporal patterns were seen for the presentation of patients with rickettsioses in Kandy and Kurunegala districts for 2009 and 2010.
CONCLUSIONS: This study expanded knowledge on the distribution of rickettsioses in Sri Lanka and their clinical profiles which in turn helps in the clinical diagnosis of these infections.

DOI: 10.1186/1471-2334-11-328
PMCID: PMC3248378
PMID: 22118601 [PubMed - indexed for MEDLINE]

630. MMW Munch Med Wochenschr. 1982 Feb 12;124(6):133-5.

[Tsutsugamushi fever. Case report on an imported disease].

[Article in German]

Schomerus P, Holzer E.

PMID: 6804859 [PubMed - indexed for MEDLINE]

631. Lancet Glob Health. 2013 Jul;1(1):e46-54.

Causes of non-malarial fever in Laos: a prospective study.

Mayxay M, Castonguay-Vanier J, Chansamouth V, Dubot-Pérès A, Paris DH,

Phetsouvanh R, Tangkhabuanbutra J, Douangdala P, Inthalath S, Souvannasing P, Slesak G, Tongyoo N, Chanthongthip A, Panyanouvong P, Sibounheuang B, Phommasone K, Dohnt M, Phonekeo D, Hongvanthong B, Xayadeth S, Ketmayoon P, Blacksell SD, Moore CE, Craig SB, Burns MA, von Sonnenburg F, Corwin A, de Lamballerie X, González IJ, Christophel EM, Cawthorne A, Bell D, Newton PN.

Comment in
Lancet Glob Health. 2013 Jul;1(1):e11-2.

BACKGROUND: Because of reductions in the incidence of Plasmodium falciparum malaria in Laos, identification of the causes of fever in people without malaria, and discussion of the best empirical treatment options, are urgently needed. We aimed to identify the causes of non-malarial acute fever in patients in rural Laos.
METHODS: For this prospective study, we recruited 1938 febrile patients, between May, 2008, and December, 2010, at Luang Namtha provincial hospital in northwest Laos (n=1390), and between September, 2008, and December, 2010, at Salavan provincial hospital in southern Laos (n=548). Eligible participants were aged 5-49 years with fever (≥38°C) lasting 8 days or less and were eligible for malaria testing by national guidelines.
FINDINGS: With conservative definitions of cause, we assigned 799 (41%) patients a diagnosis. With exclusion of influenza, the top five diagnoses when only one aetiological agent per patient was identified were dengue (156 [8%] of 1927 patients), scrub typhus (122 [7%] of 1871), Japanese encephalitis virus (112 [6%] of 1924), leptospirosis (109 [6%] of 1934), and bacteraemia (43 [2%] of 1938). 115 (32%) of 358 patients at Luang Namtha hospital tested influenza PCR-positive between June and December, 2010, of which influenza B was the most frequently detected strain (n=121 [87%]). Disease frequency differed significantly between the two sites: Japanese encephalitis virus infection (p=0·04), typhoid (p=0·006), and leptospirosis (p=0·001) were more common at Luang Namtha, whereas dengue and malaria were more common at Salavan (all p<0·0001). With use of evidence from southeast Asia when possible, we estimated that azithromycin, doxycycline, ceftriaxone, and ofloxacin would have had significant efficacy for 258 (13%), 240 (12%), 154 (8%), and 41 (2%) of patients, respectively.
INTERPRETATION: Our findings suggest that a wide range of treatable or preventable pathogens are implicated in non-malarial febrile illness in Laos. Empirical treatment with doxycycline for patients with undifferentiated fever and negative rapid diagnostic tests for malaria and dengue could be an appropriate strategy for rural health workers in Laos.
FUNDING: Wellcome Trust, WHO-Western Pacific Region, Foundation for Innovative New Diagnostics, US Centers for Disease Control and Prevention

DOI: 10.1016/S2214-109X(13)70008-1
PMCID: PMC3986032
PMID: 24748368 [PubMed - indexed for MEDLINE]

632. Zhonghua Liu Xing Bing Xue Za Zhi. 2000 Feb;21(1):34-6.

[Studies on three types of natural foci of tsutsugamushi disease in eastern part of China].

[Article in Chinese]

Wu G(1), Guo H, Yu M.

Author information:
(1)Department of Epidemiology, Institute of Military Medicine, Nanjing Command,
Nanjing 210002, China.

OBJECTIVE: In order to identify the types of natural foci of tsutsugamushi disease to provide basis for prevention, a series of studies were carried out in Fujian, Zhejiang and Jiangsu Provinces.
METHODS: (1) Representative points for investigation in three Provinces were selected. (2) Data on geographical landscape and epidemiology were collected. (3) Field survey and experimental studies on rats and chigger mites in relation to the disease.
RESULTS: Three representative types of natural foci of tsutsugamushi disease were demonstrated. They were (1) Coastal island type, mainly in Fujian with major reservoir host as Rattus losea and major vector mite as L. deliense; prevalent season: summer; type of Rt: Gilliam: (2) Inland mountain - forest type in Zhejiang with major reservoir host as R. confucianus and major vector miteas L. gaohuense; prevalent season: summer; type of Rt: Gilliam. (3) Inland plain - hills type in Jiangsu with major reservoir hosts: Apodemus agrarius, R. confucianus, R. norvegicus and Crocidura lasiura; major vector mite: L. scutellare; prevalent seasons: autumn and winter; type of Rt: Kawasaki.
CONCLUSION: The types of Rt, reservoir hosts, vector chigger mites and epidemiological features of tsutsugamushi disease in Fujian, Zhejiang and Jiangsu Province were found different. Thus, the preventive measures should also be distinct from one another.

PMID: 11860755 [PubMed - indexed for MEDLINE]

633. Riv Ital Ig. 1967 Jan-Apr;27(1):80-9.

[An African rickettsial disease in a focus of Kenya pseudo-typhus].

[Article in Italian]

Del Trono L.

PMID: 5632584 [PubMed - indexed for MEDLINE]

634. PLoS One. 2013 Apr 9;8(4):e60634. doi: 10.1371/journal.pone.0060634. Print 2013.

A prospective study of the causes of febrile illness requiring hospitalization in children in Cambodia.

Chheng K(1), Carter MJ, Emary K, Chanpheaktra N, Moore CE, Stoesser N, Putchhat H, Sona S, Reaksmey S, Kitsutani P, Sar B, van Doorn HR, Uyen NH, Van Tan L, Paris DH, Blacksell SD, Amornchai P, Wuthiekanun V, Parry CM, Day NP, Kumar V.

Author information:
(1)Angkor Hospital for Children, Siem Reap, Kingdom of Cambodia.

Erratum in
PLoS One. 2015;10(3):e0119976. Paris, Daniel [corrected to Paris, Daniel H].

BACKGROUND: Febrile illnesses are pre-eminent contributors to morbidity and mortality among children in South-East Asia but the causes are poorly understood. We determined the causes of fever in children hospitalised in Siem Reap province, Cambodia.
METHODS AND FINDINGS: A one-year prospective study of febrile children admitted to Angkor Hospital for Children, Siem Reap. Demographic, clinical, laboratory and outcome data were comprehensively analysed. Between October 12(th) 2009 and October 12(th) 2010 there were 1225 episodes of febrile illness in 1180 children. Median (IQR) age was 2.0 (0.8-6.4) years, with 850 (69%) episodes in children <5 years. Common microbiological diagnoses were dengue virus (16.2%), scrub typhus (7.8%), and Japanese encephalitis virus (5.8%). 76 (6.3%) episodes had culture-proven bloodstream infection, including Salmonella enterica serovar Typhi (22 isolates, 1.8%), Streptococcus pneumoniae (13, 1.1%), Escherichia coli (8, 0.7%), Haemophilus influenzae (7, 0.6%), Staphylococcus aureus (6, 0.5%) and Burkholderia pseudomallei (6, 0.5%). There were 69 deaths (5.6%), including those due to clinically diagnosed pneumonia (19), dengue virus (5), and melioidosis (4). 10 of 69 (14.5%) deaths were associated with culture-proven bloodstream infection in logistic regression analyses (odds ratio for mortality 3.4, 95% CI 1.6-6.9). Antimicrobial resistance was prevalent, particularly in S. enterica Typhi, (where 90% of isolates were resistant to ciprofloxacin, and 86% were multi-drug resistant). Comorbid undernutrition was present in 44% of episodes and a major risk factor for acute mortality (OR 2.1, 95% CI 1.1-4.2), as were HIV infection and cardiac disease.
CONCLUSION: We identified a microbiological cause of fever in almost 50% of episodes in this large study of community-acquired febrile illness in hospitalized children in Cambodia. The range of pathogens, antimicrobial susceptibility, and co-morbidities associated with mortality described will be of use in the development of rational guidelines for infectious disease treatment and control in Cambodia and South-East Asia.

DOI: 10.1371/journal.pone.0060634
PMCID: PMC3621876
PMID: 23593267 [PubMed - indexed for MEDLINE]

635. Microbiol Immunol. 1985;29(9):859-72.

Epidemiological studies on the background of the endemic occurrence of tsutsugamushi disease in Toyama Prefecture. I. Epidemiology of infection with Rickettsia tsutsugamuchi among field rodents in endemic and nonendemic areas.

Ishikura M, Watanabe M, Morita O, Uetake H.

In order to clarify the epidemiological background of the endemic occurrence of tsutsugamushi disease in Toyama Prefecture, Japan, since 1978, comparative surveys have been carried out between endemic and nonendemic areas. Rickettsia tsutsugamushi (Rt) was isolated at a rate of about 36% (158/439) from field rodents in the endemic area while it was not isolated from any of 280 in nonendemic areas. In all of six stations in the endemic area, a significantly high proportion of rodents were found to be Rt carriers. However, no Rt was

isolated from rodents captured from July to September. The organism was isolated from rodents captured in the other months, especially in a high proportion in November when infestation of rodents with Leptotrombidium pallidum was at its peak. When the rodents were examined by indirect immunofluorescence staining, the rate of anti-Rt antibody-positive animals was about 55% (157/287) and about 17% (62/368) in endemic and nonendemic areas, respectively. Larvae of mites collected from the rodents were found to belong to four genera and 11 species. Among them L. pallidum was the only mite that had been known to be a vector of Rt. L. pallidum was found most frequently and in abundance from rodents in the endemic area, whereas it was present in very small numbers in rodents in nonendemic areas. The infestation of rodents with L. pallidum showed a seasonal variation, i.e. two peaks per year, in spring and autumn, and the number of mites detected was markedly greater in November than in spring. Rt was isolated from L. pallidum on rodents captured in the endemic area.

PMID: 3934506 [PubMed - indexed for MEDLINE]

636. Chin Med J (Engl). 2002 Feb;115(2):272-5.

Natural foci of tsutsugamushi disease in the Nan Peng Lie Islands in China.

Wang S(1), Huang J, Peng G, Jiang P, Zheng N, Liu J, Zhu S, Wang Z.

Author information:
(1)Department of Epidemiology, Medical Institute, Guangzhou Command PLA, Guangzhou 510507, China.

OBJECTIVE: To investigate natural foci of tsutsugamushi disease whose incidence has increased in the Nan Peng Lie Islands in China, an area where this disease has not been previously recorded.
METHODS: We recorded the natural foci and isolated Orientia tsutsugamushi (O. tsutsugamushi) organism. We also studied prevention measures.
RESULTS: These islands had the natural foci of a south subtropical zone. The main host and vector were Rattus norvegicus and Leptotrombidium deliens (L. deliens), respectively. The seasonal quantity trends of Rattus norvegicus and Leptotrombidium deliens were consistent with the incidence of human infection. Thirty-five strains of O. tsutsugamushi were isolated from Rattus norvegicus and L. deliense. The identification of 7 strains showed that most strains were Karp. Seroepidemiology showed a high prevalence of antibody against O. tsatsugamushi among local people. After prevention measures were used, the incidence was decreased.
CONCLUSION: This was the first successful confirmation that the Nan Peng Lie Islands were natural foci of tsutsugamushi disease.

PMID: 11940347 [PubMed - indexed for MEDLINE]

637. Jpn J Exp Med. 1954 Dec;24(6):335-61.

Comparative epidemiology of Tsutsugamushi disease in Japan.

SASA M.

PMID: 13270967 [PubMed - indexed for MEDLINE]

638. Med Parazitol (Mosk). 1964 Nov-Dec;33:718-21.

[RED MITES IN A NATURAL FOCUS OF TSUTSUGAMUSHI FEVER IN THE SOUTHERN MARITIME TERRITORY].

[Article in Russian]

KUDRIASHOVA NI, TARASEVICH IV.

PMID: 14321587 [PubMed - indexed for MEDLINE]

639. Zh Mikrobiol Epidemiol Immunobiol. 1964 May;41:19-24.

[A NATURAL FOCUS OF TSUTSUGAMUSHI FEVER].

[Article in Russian]

TARASEVICH IV, KULAGIN SM, KUDRIASHOVA NI, GOPACHENKO IM, SOMOV GP.

PMID: 14231861 [PubMed - indexed for MEDLINE]

640. Ann Soc Belges Med Trop Parasitol Mycol. 1964;44:207-16.

SCRUBTYPHUS, AN EPIDEMIOLOGICAL STUDY.

BEKKER BV, DINGER JE, WOLFF HL.

PMID: 14237562 [PubMed - indexed for MEDLINE]

641. Jpn J Infect Dis. 2006 Aug;59(4):273.

Epidemiology of tsutsugamushi disease and Japanese spotted fever in Kagoshima Prefecture, Japan.

Gokuden M(1), Ishitani K, Yoshikuni K, Ueno N, Shinkawa N, Kuramoto T, Honda T, Miyata Y.

Author information:
(1)Kagoshima Prefectural Institute of Environmental Research and Public Health, Kagoshima 892-0853, Japan. gokuden@kg-env.org

PMID: 16936351 [PubMed - indexed for MEDLINE]

642. Zh Mikrobiol Epidemiol Immunobiol. 1968 Aug;45(8):86-92.

[Various results of a study of tsutsugamushi fever in the Primorsk region].

[Article in Russian]

Shapiro MI, Somov GP, Lazarev AN, Gopachenko IM, Netskiĭ KV.

PMID: 4978504 [PubMed - indexed for MEDLINE]

643. J Korean Med Sci. 1995 Aug;10(4):227-38.

Current status of tsutsugamushi disease in Korea.

Chang WH(1).

Author information:
(1)College of Medicine, Hallym University, Korea.

DOI: 10.3346/jkms.1995.10.4.227
PMCID: PMC3054065
PMID: 8593201 [PubMed - indexed for MEDLINE]

644. Nihon Rinsho. 2001 Nov;59 Suppl 7:162-9.

[Rickettsial diseases].

[Article in Japanese]

Oda H(1).

Author information:
(1)Department of Bacteriology, Faculty of Medicine, Kagoshima University.

PMID: 11808116 [PubMed - indexed for MEDLINE]

645. Kansenshogaku Zasshi. 1993 Mar;67(3):196-201.

[Epidemiology of Tsutsugamushi disease and typing of isolated Rickettsia in Chiba Prefecture].

[Article in Japanese]

Kaiho I(1), Tokieda M, Yoshida Y, Furuya Y, Murata M, Tanaka H, Kawamura A Jr.

Author information:

(1)Pubulic Health Laboratory of Chiba Prefecture.

In Chiba Prefecture, the first patient of Tsutsugamushi disease was found in the southern part in the 1950's, but after that no patient was detected until 1982. After 1982, patients have been noticed again, the number of patients has been increasing year by year. The number of serologically confirmed cases was 152 and 157 in 1989 and 1990, respectively with indirect immunofluorescence assay. About 90 percent of the patients were found in November and December, prevalently in the southern part of the prefecture. On the other hand, a few patients were found yearly in a harf of towns and villages in this prefecture. The number of isolated strains of Rickettsia tsutsugamushi from patients was 4, i.e., TR6030, TR6310, TR6311, and TR6324 in 1986 and 3, TR1811, TR1827 and TR1829, in 1991. Six isolates except TR6303 reacted with anti-Kawasaki monoclonal antibody but not with other strain specific monoclonal antibodies. Therefore, these 6 isolates were determined as Kawasaki type strain. An isolate. TR6303, reacted with anti-Kuroki monoclonal antibody at a titer of 1:2560 and anti-Karp monoclonal antibody at a titer of 1:320. This result suggested that the recent Tsutsugamushi disease is mostly caused by Kawasaki types in this prefecture.

PMID: 8486974 [PubMed - indexed for MEDLINE]

646. Vestn Ross Akad Med Nauk. 2008;(7):36-8.

[Contribution of I.V. Tarasevich to the study of tsutsugamushi disease].

[Article in Russian]

Shubin FN.

This article summarizes the results of study of tsutsugamushi disease in the Primorye and Tadzhikistan, in its epidemiology, diagnosis and biology of the Rickettsia tsutsugamushi. There is also information on the vectors of the disease, rickettsial infection within chiggers and transtadial transmission from larva to nymph, hosts of trombiculids and natural infection with R. tsutsugamushi.

PMID: 18756782 [PubMed - indexed for MEDLINE]

647. Kansenshogaku Zasshi. 1984 Dec;58(12):1279-84.

[Epidemiology of tsutsugamushi disease in Hokuriku district, Japan. 1. Prevalence of antibody to Rickettsia tsutsugamushi].

[Article in Japanese]

Takada N, Tatefuji N, Hoshino T, Ogata A, Fujiki N.

PMID: 6442722 [PubMed - indexed for MEDLINE]

648. Zh Mikrobiol Epidemiol Immunobiol. 1969 Aug;46(8):42-8.

[The epidemiology of tsutsugamushi fever in the Primorsk region].

[Article in Russian]

Shaliro MI, Somov GP, Golachenko IM, Natskiĭ KV.

PMID: 5362261 [PubMed - indexed for MEDLINE]

649. Schweiz Med Wochenschr. 1990 Jul 28;120(30):1109-11.

[Imported tsutsugamushi fever].

[Article in German]

Albisser M(1), Ritschard T.

Author information:
(1)Medizinische Klinik, Kantonsspital Luzern.

Scrub typhus rarely figures among the imported tropical diseases in western Europe. Rickettsia tsutsugamushi is transmitted by larval mites. A typical eschar develops at the site of the mite bite during the incubation period, after which systemic symptoms (remittent or continuous high fever, severe headache, tender lymphatic glands and symptoms of bronchitis) develop with sudden onset. Antibodies appear during the second week and can be shown by a positive and increasing titer against Prot. OXK (Weil-Felix) or by specific rickettsial agglutination. The Weil-Felix reaction is neither very sensitive nor very specific. Therefore, treatment should be started as soon as suspicion arises whether a positive serology is available or not. Tetracycline drugs are effective treatment and fever subsides in less than 24 hours in most patients. - We report the rare observation of a tourist who imported tsutsugamushi fever from India.

PMID: 2392662 [PubMed - indexed for MEDLINE]

650. Kansenshogaku Zasshi. 1995 Jul;69(7):840-3.

[A case of tsutsugamushi disease which occurred in south western Shikoku].

[Article in Japanese]

Yamauchi H(1), Soga S, Kono H, Kondo T, Sayama K, Tange Y, Fujita S.

Author information:
(1)Department of Internal Medicine, Uwajima City Hospital.

We report a case of tsutsugamushi disease found in south western Shikoku. A 64-year-old male who lived in Towa Village in Kochi, developed a fever and

headache on April 6, 1994, and was admitted to Uwajima City Hospital on April 15, with a ten-day history of illness. He had an eschar on the right anterior side of the breast and an enlargement of the right axillary lymph node, without a rash. Laboratory data showed mild liver injury and atypical lymphocytes with 6% in peripheral blood. After his blood was drawn for rickettsial isolation, the minocycline was administered. His symptoms improved rapidly and was discharged in good condition. We successfully isolated the causative agent, Rickettsia tsutugamushi, and designated it as the Shiba strain. High antibody titer against the Kato, Karp and Gilliam strains was detected in serum on admission and increased during the course of the disease. In Shikoku, tsutsugamushi disease is rare and only 13 cases were reported during last ten years. Especially in south western district of Shikoku, there have been no case reported since 1960. This case is important epidemiologically and suggests that we should pay attention to this disease.

PMID: 7561256 [PubMed - indexed for MEDLINE]

651. Trop Med Int Health. 2003 Sep;8(9):803-11.

Emerging rickettsial infections in Sri Lanka: the pattern in the hilly Central Province.

Kularatne SA(1), Edirisingha JS, Gawarammana IB, Urakami H, Chenchittikul M, Kaiho I.

Author information:
(1)Department of Medicine, Faculty of Medicine, University of Peradeniya, Peradeniya, Sri Lanka. samkul@sltnet.lk

OBJECTIVES: To identify different rickettsial infections using a specific immunofluorescent technique in patients clinically diagnosed as 'typhus fever' in the Central Province of Sri Lanka, and to define the clinical picture, assess the severity of infection and to determine the pattern of geographical distribution of the infections of the hospital-based patients.
METHODS: A specific indirect immunofluorescent antibody technique was used on the sera of two groups of patients in laboratories in Japan and Thailand.
RESULTS: We serodiagnosed infections with Orientia tsutsugamushi, Rickettsia typhi and spotted fever group in 56 of 118 clinically investigated patients. There were eight infections with O. tsutsugamushi, two with R. typhi and 10 spotted fever group patients with IgM antibodies suggestive of acute infection. Nineteen patients had antibodies against these three rickettsial species, suggestive of past exposure, co-infection or cross-reactivity of antigens. Discrete, erythematous maculopapular rash was common to all three types of infection except for five patients who had no rash. Five patients positive for spotted fever antibodies developed fern-leaf type skin necrosis with severe illness. Duration of the febrile period ranged from 4 to 23 days with defervescence occurring after specific antibiotic treatment.
CONCLUSIONS: The study has shown the presence of different types of rickettsial infections in the Central Province of Sri Lanka. The characterization of the clinical picture and the severity of infection provide useful information for the proper management of the patients in the future.

PMID: 12950666 [PubMed - indexed for MEDLINE]

652. J Assoc Physicians India. 1989 May;37(5):357.

Typhus myocarditis.

Ganjoo RK, Sharma SN, Roy AK.

PMID: 2613658 [PubMed - indexed for MEDLINE]

653. Ann N Y Acad Sci. 2006 Oct;1078:60-73.

Rickettsioses in Japan and the far East.

Mahara F(1).

Author information:
(1)Mahara Hospital, 6-1, Aratano, Anan-city, Tokushima, 779-1510, Japan.
mahara@tokushima.med.or.jp

Three rickettsial diseases are known to exist in Japan currently: Japanese spotted fever (JSF), Tsutsugamushi disease (TD; scrub typhus), and Q fever. Since April 1999, the system for infection control and prevention in Japan has changed drastically. JSF, Q fever, and TD, as emerging infectious diseases, are designated as national notifiable diseases.The geographic distribution of JSF patients is along the coast of central and southwestern Japan, whereas TD and Q fever occur almost all over the country. The number of JSF patients reported was 216 cases during 1984-1998 and 268 cases, under the revised law, in 1999-2004. About 300-1000 cases of TD occur every year, and 7-46 cases of Q fever in 1999-2004. The number of cases of JSF and its endemic area are gradually increasing. There was only one fatality due to JSF until 2003, whereas two patients died of JSF in 2004, so JSF is still a life-threatening disease in Japan. Treatment of fulminant JSF consists of prompt administration of a combination of tetracycline and quinolone. Recent tick surveys revealed that the most probable vectors of JSF are Haemophysalis flava and Haemophysalis hystericis. In addition to R. japonica, two serotypes or species of spotted fever group rickettsiae have been isolated from ticks in Japan; one is closely related to R. helvetica and the other is a new genotype of unknown genotype AT, which is closely related to a Slovakian genotype. These serotypes are of uncertain clinical significance. Epidemiology of rickettsioses in the Far East is mentioned briefly.

DOI: 10.1196/annals.1374.007
PMID: 17114681 [PubMed - indexed for MEDLINE]

654. Tokai J Exp Clin Med. 2001 Apr;26(1):19-24.

Tsutsugamushi disease in Kanagawa Prefecture, Japan: clinical report of two cases and survey of other incidences in 1998.

Ozaki H(1), Matsuyama T, Hirabayashi K, Iizuka M, Urano K, Kawakubo Y, Kanno S, Ozawa A, Ohkido M, Hirota F, Nagashima N.

Author information:
(1)Department of Dermatology, Tokai University School of Medicine, Isehara, Kanagawa, Japan.

Tsutsugamushi disease is characterized by the early appearance of a black crust at the bitten area and the subsequent development of macular or macropapular rush on the whole body with high fever. While treatment with tetracycline derivatives and chloramphenicols is effective, delayed diagnosis or inappropriate treatment will lead to fatality. In this report, we describe two typical cases of tsutsugamushi disease and discuss other incidences in Kanagawa Prefecture, Japan, in 1998. One of the present two patients was diagnosed to be a case of the new type by Kawasaki strain of Rickettsia tsutsugamushi, while responsible strain was not identified for the other case. Since the disease is spreading widely even to suburban areas, we emphasize the need to consider the possible diagnosis of tsutsugamushi disease in patients with generalized eruption and high fever.

PMID: 11592298 [PubMed - indexed for MEDLINE]

655. Trop Dis Bull. 1971 Aug;68(8):913-9.

Summary of recent abstracts. VII. Rickettsial diseases.

Cannon DA.

PMID: 4998338 [PubMed - indexed for MEDLINE]

656. Nihon Hifuka Gakkai Zasshi. 1986 Jul;96(8):791-803.

[A case of tsutsugamushi disease with isolation of Rickettsia from peripheral blood and tsutsugamushi disease in Japan].

[Article in Japanese]

Kameda Y, Takigami T.

PMID: 3097353 [PubMed - indexed for MEDLINE]

657. Southeast Asian J Trop Med Public Health. 2002 Jun;33(2):314-20.

The use of the indirect immunoperoxidase test for the serodiagnosis of rickettsial diseases in Malaysia.

Tay ST, Rohani MY.

The indirect immunoperoxidase (HP) test has been used extensively in most government hospitals in Malaysia for the serodiagnosis of scrub typhus, murine typhus and tick typhus during the 1990s. The test was used to determine the IgG and IgM antibody titers in patients' sera for three rickettsial species, ie Orientia tsutsugamushi OT; the causative agent of scrub typhus), Rickettsia typhi (RT; the causative agent of murine typhus), and TT118 spotted fever group rickettsiae (TT; the causative agent of tick typhus). The serological findings obtained from Malaysian hospitals using the IIP test (1994-1999) were analyzed. During the six-year period, a total of 61,501 patients' sera were tested, of which 9.6%, 10.5%, and 12.9% had antibody (IgG and/or IgM of > or = 1:50) for OT, RT and TT respectively. A total of 8.6%, 9.8%, and 9.7% of sera had IgG antibody of > or = 1:50 for OT, RT, and TT respectively, indicating past infection. A total of 3.4%, 3.8%, and 6.4 % of sera had IgM antibody of > or = 1:50 for OT, RT, and TT respectively, indicating recent infection. A total of 2,986 (4.9%), 1,882 (3.1%), and 1,574 (2.6%) of sera had IgG and/or IgM antibody titers of > or = 1:400 for OT, RT, and TT respectively, suggesting active rickettsial infection. The seropositivity rates of OT, RT and TT varied according to geographical locations. While the seropositivity of OT remained constant during the six-year period, a reduction in the seropositivity of both RT and TT was noted during recent years. The serological findings reflect the endemicity of rickettsial diseases, including tick typhus, and endemic typhus in various parts of Malaysia. Awareness of these diseases by health and medical staff and by the general public is important if the mortality and morbidity associated with scrub typhus, tick typhus, and murine typhus in Malaysia, are to be reduced.

PMID: 12236431 [PubMed - indexed for MEDLINE]

658. Nihon Rinsho. 1984 Mar;42(3):725-39.

[Tsutsugamushi disease--on the recent multiple incidences of a new type].

[Article in Japanese]

Otsuru M.

PMID: 6433079 [PubMed - indexed for MEDLINE]

659. Jpn J Exp Med. 1980 Apr;50(2):91-105.

Studies on inapparent infection of tsutsugamuschi disease in Izu Shichito Islands: seroepidemiology and demonstration of an avirulent Rickettsia strain for mice.

Kawamura A Jr, Murata M, Osono M, Nogami S, Shirasaka A, Tanaka H, Sudo K, Suzuki K, Miyairi T, Kijima H.

PMID: 6779033 [PubMed - indexed for MEDLINE]

660. Acta Med Biol (Niigata). 1967 Dec;15:1-7.

An outline of research history of the tsutsugamushi disease in Niigata prefecture of Japan.

Ito T.

PMID: 4968947 [PubMed - indexed for MEDLINE]

661. Nihon Naika Gakkai Zasshi. 1984 Mar;73(3):401-7.

[An autopsy case of the first occurrence of tsutsugamushi disease in Gifu Prefecture--with special reference to complication with disseminated intravascular coagulation].

[Article in Japanese]

Sassa H, Shibata T, Oba M, Okubo M, Niwa T, Matsui E.

PMID: 6376667 [PubMed - indexed for MEDLINE]

662. Yakugaku Zasshi. 1987 Oct;107(10):751-66.

[Microbiological studies on Rickettsia tsutsugamushi].

[Article in Japanese]

Tamura A.

PMID: 3126288 [PubMed - indexed for MEDLINE]

663. Kansenshogaku Zasshi. 1982 Aug;56(8):655-63.

[Epidemiological, immunological and etiological study on tsutsugamushi disease in Miyazaki district].

[Article in Japanese]

Tachibana N, Kusune E, Yokota T, Shishime E, Tsuda K, Oshikawa T.

PMID: 6818301 [PubMed - indexed for MEDLINE]

664. Indian J Pediatr. 2013 Mar;80(3):183-9. doi: 10.1007/s12098-012-0791-z. Epub 2012 Jun 14.

Incidence and etiology of acute kidney injury in southern India.

Krishnamurthy S(1), Mondal N, Narayanan P, Biswal N, Srinivasan S, Soundravally R.

Author information:
(1)Department of Pediatrics, Jawaharlal Institute of Postgraduate Medical Education and Research (JIPMER), Pondicherry 605006, India. drsriramk@yahoo.com

Comment in
  Indian J Pediatr. 2013 Sep;80(9):797.
  Indian J Pediatr. 2013 Sep;80(9):796.

OBJECTIVES: To determine the incidence, etiology, short term outcome and predictors of mortality in hospitalized children aged 1 mo to 13 y with Acute Kidney Injury (AKI).
METHODS: This prospective observational study was conducted in the pediatric wards and the pediatric intensive care unit (PICU) of a tertiary hospital in southern India, to study the clinico-etiological profile of AKI (defined according to the Acute Kidney Injury Network criteria). From June 2010 through March 2011, 2376 children were included in the study.
RESULTS: The incidence of AKI was 5.2 % in the pediatric wards and 25.1 % in the PICU. AKI occurred in association with infections (55.4 %), acute glomerulonephritis (16.9 %), cardiac disease (4.8 %), envenomations (4.2 %) and hemolytic uremic syndrome (3.6 %). Pneumonia constituted 26.1 % of the infections. Tropical febrile illnesses (dengue, scrub typhus, enteric fever, cholera, tuberculosis, malaria and leptospirosis) constituted 15.6 % of children with AKI. Dialysis was required in 14.5 % of patients; mortality was 17.5 %. A significant proportion of children (17.5 % of survivors) had partial renal recovery at discharge. On multivariate logistic regression, dysnatremia and meningoencephalitis were independent predictors of mortality in AKI.
CONCLUSIONS: The incidence of AKI is high in the patient population, including the non-critically ill children. AKI continues to be associated with adverse outcomes. Presence of dysnatremia and meningoencephalitis are poor predictors of outcome in AKI.

DOI: 10.1007/s12098-012-0791-z
PMID: 22692434 [PubMed - indexed for MEDLINE]

665. Zhongguo Ji Sheng Chong Xue Yu Ji Sheng Chong Bing Za Zhi. 2006 Oct;24(5):398-9.

[Five cases of Tsutsugamushi disease in Ganzhou].

[Article in Chinese]

Wu ZF, Deng HZ, Su ZL.

PMID: 17361830 [PubMed - indexed for MEDLINE]

666. Intern Med. 2002 May;41(5):408-11.

Successful diagnosis using scab for PCR specimen in Tsutsugamushi disease.

Ono A(1), Nakamura K, Higuchi S, Miwa Y, Nakamura K, Tsunoda T, Kuwabara H,
Furuya Y, Dobashi K, Mori M.

Author information:
(1)Department of Internal Medicine, Numata National Hospital, Gunma.

We treated a case of Tsutsugamushi disease diagnosed by polymerase chain reaction (PCR) using a scab specimen at the bite site of trombiculid mites. Otherwise the diagnosis could not be confirmed by serum antibody test nor the PCR test of blood. The genome of Rickettsia tsutsugamushi was detected and identified as the Kawasaki serotype strain. An attempt to extract the genome from the scab has not been reported, thus our data suggest that the scab is a useful specimen to confirm the diagnosis of Tsutsugamushi disease.

PMID: 12058894 [PubMed - indexed for MEDLINE]

667. Jpn J Ophthalmol. 1997 May-Jun;41(3):196-9.

Conjunctival injection, episcleral vessel dilation, and subconjunctival hemorrhage in patients with new tsutsugamushi disease.

Kato T(1), Watanabe K, Katori M, Terada Y, Hayasaka S.

Author information:
(1)Department of Ophthalmology, Asahi General Hospital, Japan.

Tsutsugamushi disease is found in two types: classical and new. There have been very few reports describing the ocular findings in patients with the new form. We have described four patients with this type, selected according to their clinical and laboratory findings, including immunofluorescent titers and polymerase chain reaction results. Eyes were examined by standard ophthalmic procedures. Patient 1 had bilateral conjunctival injection and subconjunctival hemorrhage; patients 2-4 had conjunctival injection and episcleral vessel dilation bilaterally. We believe that conjunctival injection is found in most eyes of patients with new tsutsugamushi disease.

PMID: 9243318 [PubMed - indexed for MEDLINE]

668. Microbiol Immunol. 1987;31(10):985-99.

Epidemiological studies on the background of the endemic occurrence of tsutsugamushi disease in Toyama Prefecture. II. Anti-Rickettsia tsutsugamushi antibody-positive rate in inhabitants of endemic and nonendemic areas.

Ishikura M(1), Watanabe M, Matsuura K, Nakayama T, Morita O, Uetake H, Sakurada
N.

Author information:
(1)Toyama Institute of Health.

With a view to clarifying the actual state of inapparent infection of tsutsugamushi diseases, inhibitants of endemic and nonendemic areas were screened for anti-Rickettsia tsutsugamushi antibody (anti-Rt antibody) by the indirect immunofluorescence test. The anti-Rt antibody-positive rate in the inhabitants of the endemic area (about 50%) was statistically significantly higher than that in the nonendemic area (14.7%). The antibody titer in the inhabitants of the endemic area was 10-160, and the number of inhibitants showing a high antibody titer was 2-4 times larger than that of the nonendemic area. A total of 257 volunteers in the endemic area were analyzed for the changes in anti-Rt antibody titer over 1.5-2 years on an individual basis. An increase in the antibody titer was found in 20 inhabitants. There was no difference in the anti-Rt antibody-positive rate between male and female in either the endemic or the nonendemic area. The positive rate was also compared as to the distribution by 10 years of age. In the endemic area, there were no significant differences in the positive rate between any pair of 10-year age groups from 30s to 60s, whereas in the nonendemic area, the positive rate in the teen-age group was significantly lower than those in the age groups of 20 years or older. In Yamada district, the numbers of serum samples obtained from each age group were about the same, and the distribution of the positive rates showed a normal distribution. The nurse students having their homes in Toyama Prefecture were plotted on the map as for their anti-Rt antibody and geographical distribution. The results showed that many of them having homes in the endemic area were positive for the antibody, while some antibody-positives were scattered all over Toyama Prefecture.

PMID: 3123897 [PubMed - indexed for MEDLINE]

669. Zhonghua Liu Xing Bing Xue Za Zhi. 1997 Aug;18(4):233-5.

[Studies on clinical epidemiology of Tsutsugamushi disease of the autumn-winter type in the easten suburbs of Jinan].

[Article in Chinese]

Yang ZQ(1), Yu XM, Liu YX.

Author information:
(1)Institute of Military Medicine, Jinan Command.

This paper reported the epidemiological characteristics of 154 cases under investigation for tsutsugamushi disease of autumn-winter type in the eastern suburb of Jinan. Results showed that the characteristics differed greatly from those in the southern part of China. Majority of patients had toxic symptoms due to infections but specific characteristics were less obvious. Eschars and ulcers appeared only in 14.94% of the cases and occurred only in autumn-winter season. Clinically mild cases constituted the majority. Cases not only distributed sporadically, but also had multi-organ lesions (MOL). Young peasants seemed to be most vulnerable. The pathogen of the disease belonged to low virulent strain. Serological typing belonged to the Gilliam type. The diagnosis and the treatment of the disease were discussed. Specific serum IgG of the disease was detected 6 years after convalescence.

PMID: 9812527 [PubMed - indexed for MEDLINE]

670. P N G Med J. 2006 Mar-Jun;49(1-2):43-6.

A serological survey of rickettsial infections in the Gazelle Peninsula, East New Britain and a review of the literature.

Faa AG(1), Graves SR, Nguyen C, Stenos J.

Author information:
(1)St Mary's Hospital Vunapope, Kokopo, Papua New Guinea.

Of the various rickettsial diseases, only scrub typhus has been well documented in Papua New Guinea. A review of the historical literature confirms this. A serological survey was conducted on 113 antenatal patients presenting to a district hospital in Kokopo, East New Britain. Results suggested that a spotted fever rickettsial infection is common in this area with a seroprevalence of about 17% in young women. There was no evidence of scrub typhus or murine (endemic) typhus in the population sampled. Clinical implications of these findings are discussed.

PMID: 18396611 [PubMed - indexed for MEDLINE]

671. Kansenshogaku Zasshi. 1997 Dec;71(12):1193-8.

[Clinical and serological study of tsutsugamushi disease in northern Osumi, Kagoshima Prefecture].

[Article in Japanese]

Yagi Y(1), Yamamoto S, Yoshiie K, Noda S.

Author information:
(1)Yagi Clinic, Fukuyama-cho, Kagoshima Prefecture.

We report 34 cases of tsutsugamushi disease seen from 1989 to 1993 at Yagi Clinic, northern Osumi, Kagoshima Prefecture. Nineteen patients (55.9%) showed the highest antibody titers against the Kawasaki strain Orientia tsutsugamushi (Ot) and 13 (38.2%) against the Kuroki strain Ot. It is suggested that two antigenic types (Kawasaki and Kuroki) of Ot were distributed in Kagoshima Prefecture, and the Kawasaki type Ot more or less dominates Kuroki type Ot. There was no difference in clinical features between the two groups of patients.

PMID: 9483878 [PubMed - indexed for MEDLINE]

672. Nihon Naika Gakkai Zasshi. 2008 Aug 10;97(8):1873-5.

[Tsutsugamushi disease complicated with duodenal ulcer bleeding].

[Article in Japanese]

Yoshinaga S(1), Hashigo S, Nagaoka K, Hijioka S, Takekuma Y, Kitada H, Kawaguchi T, Hifumi M, Nakamura T.

Author information:
(1)Department of Gastroenterology, Kumamoto Red Cross Hospital, Kumamoto.

PMID: 18788418 [PubMed - indexed for MEDLINE]

673. Kansenshogaku Zasshi. 1997 May;71(5):474-6.

[The patients without specific antibodies, were diagnosed by PCR as Tsutsugamushi disease].

[Article in Japanese]

Furuya Y(1), Katayama T, Hara M, Yoshida Y, Kaiho I, Kawamura A Jr.

Author information:
(1)Kanagawa Prefectural Public Health Laboratory, Tokyo University.

PMID: 9209131 [PubMed - indexed for MEDLINE]

674. Nihon Kokyuki Gakkai Zasshi. 2008 May;46(5):385-9.

[Severe case of Tsutsugamushi disease with disseminated intravascular coagulation and acute respiratory distress syndrome].

[Article in Japanese]

Izumo T(1), Yamaguchi M, Onizawa S, Kiguchi T, Nagai A.

Author information:
(1)Department of Respiratory Medicine, Tachikawa General Hospital.

A 65-year-old man was admitted because of high grade fever and cough after 3 days of gathering edible wild plants. Although ceftriaxone was given to him, his symptoms did not improve. His high grade fever escalated after changing the antibiotics (imipenem with erythromycin). His situation further declined with disseminated intravascular coagulation (DIC) and acute respiratory distress syndrome (ARDS). As a result, he was transferred to the Department of Respiratory Medicine 7 days after admission. He was intubated and placed on mechanical ventilation and treated by polymyxin-direct hemoperfusion. The eschar on his chest wall caused us to suspect Tsutsugamushi disease and a blood test confirmed our suspicion. Since the antibodies for Tsutsugamushi were elevated we arrived at the diagnosis of Tsutsugamushi disease with DIC and ARDS. The administration of tetracycline was sufficient to significantly improve his condition. Because its complications are life threatening, when we see a patient with fever and

eruptions, it is necessary to keep in mind the possibility of Tsutsugamushi disease. Careful anamnesis and physical examinations are most important for the diagnosis of Tsutsugamushi disease.

PMID: 18517015 [PubMed - indexed for MEDLINE]

675. Dtsch Med Wochenschr. 1998 Apr 30;123(18):562-6.

[Tsutsugamushi fever. Rare rickettsiosis after a stay in the Philippines].

[Article in German]

Fischer BP(1), Müller A, Strauss R, Schneider HT, Hahn EG.

Author information:
(1)Medizinische Klinik I mit Poliklinik, Friedrich-Alexander-Universität Erlangen-Nürnberg.

HISTORY AND CLINICAL FINDINGS: After returning to his native Germany from a holiday in the Philippines a 37-year-old man was admitted because of high fever, cervical lymphadenopathy, pharyngitis and conjunctivitis, transient skin rash, nausea and vomiting, leukocytosis with shift to the left, atypical lymphocytes, as well as increased transaminases, LDH and cholestasis-indicating enzymes.
INVESTIGATIONS: Stool, sputum and urine cultures were negative. The chest radiogram showed bilateral mild interstitial infiltration. Antibody titres against Rickettsia tsutsugamushi were markedly raised (IgG 1:128, IgM 1:2048).
DIAGNOSIS, TREATMENT AND COURSE: Empirical antibiotic treatment with ciprofloxacin (200 mg twice daily intravenously) had no effect. As the mild signs of interstitial pneumonia progressed, clarithromycin (500 mg twice daily orally) was substituted with rapid fall in fever and gradual improvement. Tsutsugamushi infection was diagnosed serologically and the antibiotic changed to doxycycline (100 mg twice daily orally), continued for 14 days. Full remission occurred.
CONCLUSIONS: Tsutsugamushi fever should be included in the differential diagnosis if, in addition to a history of a visit to an endemic area, there is the clinical triad of skin necrosis at the site of a mite bite, regional lymphadenopathy and skin rash (in this case, no skin lesion). The infection can be lethal without adequate treatment. Tetracyclines and possibly also macrolide antibiotics are effective against the causative organism.

DOI: 10.1055/s-2007-1024011
PMID: 9615696 [PubMed - indexed for MEDLINE]

676. Am J Trop Med Hyg. 2012 Jan;86(1):46-51. doi: 10.4269/ajtmh.2012.10-0497.

Etiology of acute, non-malaria, febrile illnesses in Jayapura, northeastern Papua, Indonesia.

Punjabi NH(1), Taylor WR, Murphy GS, Purwaningsih S, Picarima H, Sisson J, Olson JG, Baso S, Wangsasaputra F, Lesmana M, Oyofo BA, Simanjuntak CH, Subekti D, Corwin AL, Richie TL.

Author information:
(1)U.S. Naval Medical Research Unit No. 2, Jakarta, Indonesia. narainhp@yahoo.com

We conducted a prospective, inpatient fever study in malaria-endemic Papua, Indonesia to determine non-malaria fever etiologies. Investigations included malaria blood films, blood culture, paired serologic samples analysis for dengue, Japanese encephalitis, leptospirosis, scrub typhus, murine typhus, and spotted fever group rickettsia. During 1997-2000, 226 patients (127 males and 99 females) 1-80 years of age (median age = 25 years) were enrolled. Positive blood cultures (n = 34, 15%) were obtained for Salmonella Typhi (n = 13), Escherichia coli (n = 8), Streptococcus pneumoniae (n = 6), Staphylococcus aureus (n = 5), Streptococcus pyogenes (n = 1), and Klebsiella pneumoniae (n = 1). Twenty (8.8%) patients were positive for leptospirosis by polymerase chain reaction. Eighty (35.4%) of 226 patients had ≥ 1 positive serology, diagnostic for 15 rickettsial and 9 dengue cases. Acid-fast bacilli-positive sputum was obtained from three patients. Most common confirmed (81 of 226, 35.8%)/suspected diagnoses were typhoid fever (n = 41), pneumonia (n = 29), leptospirosis (n = 28), urinary tract infections (n = 20), rickettsioses (n = 19), dengue (n = 17), and meningitis/encephalitis (n = 15). There were 17 deaths, 7 (46.7%) were caused by meningitis/encephalitis. Multiple positive serologic results and few confirmed diagnoses indicate the need for improved diagnostics.

DOI: 10.4269/ajtmh.2012.10-0497
PMCID: PMC3247108
PMID: 22232450 [PubMed - indexed for MEDLINE]

677. Jpn J Ophthalmol. 2001 Jan-Feb;45(1):108-10.

Branch retinal vein occlusion in the right eye and retinal hemorrhage in the left in a patient with classical Tsutsugamushi disease.

Nagaki Y(1), Hayasaka S, Kadoi C, Matsumoto M, Sakagami T.

Author information:
(1)Department of Ophthalmology, Toyama Medical and Pharmaceutical University, Toyama, Japan.

PURPOSE: To report branch retinal vein occlusion and retinal hemorrhages associated with tsutsugamushi disease.
METHODS: Case report of a 60-year-old woman who complained of fever, chills, headache, lymphadenopathy, and blurred vision in the right eye following an insect bite to the lower right forehead.
RESULTS: Serological findings showed elevated titers for the strains of Rickettsia tsutsugamushi. Ophthalmologic examination disclosed bilateral conjunctival injection, flame-shaped hemorrhage in her right fundus, and scattered hemorrhage in her left fundus. Fluorescein angiography demonstrated dye leakage and dilation of capillaries.
CONCLUSIONS: Branch retinal vein occlusion associated with classical tsutsugamushi disease, as demonstrated in our patient, may be rare.

PMID: 11163055 [PubMed - indexed for MEDLINE]

678. Eur J Epidemiol. 1991 May;7(3):237-45.

Epidemiology of rickettsial diseases.

Walker DH(1), Fishbein DB.

Author information:
(1)Department of Pathology, University of Texas Medical Branch, Galveston 77550.

Rickettsial diseases have a diversity of epidemiologic characteristics reflective of the variety of ecologic situations in which the obligate intracellular bacteria are transmitted to humans. For the spotted fever group (SFG) rickettsiae, Rickettsia typhi, R. tsutsugamushi, Coxiella burnetii, and the human ehrlichial agent, humans are a dead-end host who plays no role in the maintenance of the organism in nature. All rickettsioses exist as zoonoses. Moreover, all rickettsiae are found in infected arthopods, which generally serve as the natural hosts and can transmit the infection to the next generation of ticks, mites, chiggers, or fleas. From our anthropocentric viewpoint, Q fever aerosol infection from parturient animals and Brill-Zinsser disease ignited epidemics of louse-borne epidemic typhus are exceptions. However, silent cycles of C. burnetii in ticks and R. prowazekii in the flying squirrel flea may have maintained these agents in transovarial or enzootic cycles for eons before humans and their domestic animals arrived on the scene. Thus, the epidemiology of rickettsial diseases must be recognized as an unfortunate aberration of the rickettsial economy. Several excellent reviews of rickettsial ecology contain a wealth of useful information.

PMID: 1884775 [PubMed - indexed for MEDLINE]

679. Intern Med. 1993 Dec;32(12):937-9.

Transfiguration of rickettsial diseases: tsutsugamushi disease and spotted fever group rickettsiosis in Japan.

Tange Y(1), Kobayashi Y.

Author information:
(1)First Department of Internal Medicine, School of Medicine, Ehime University.

PMID: 8204977 [PubMed - indexed for MEDLINE]

680. Jpn J Infect Dis. 2006 Dec;59(6):404-5.

Annual incidence of tsutsugamushi disease in Miyazaki prefecture, Japan in 2001-2005.

Yamamoto S(1), Ganmyo H, Iwakiri A, Suzuki S.

Author information:

(1)Miyazaki Prefectural Institute for Public Health and Environment, Miyazaki
889-2155, Japan. yamamoto-seigo@pref.miyazaki.lg.jp

PMID: 17186964 [PubMed - indexed for MEDLINE]

681. Zhonghua Liu Xing Bing Xue Za Zhi. 1994 Feb;15(1):27-30.

[Studies on natural foci of tsutsugamushi disease of the autumn-winter type in Jiangsu].

[Article in Chinese]

Guo H(1), Wu G, Xu M.

Author information:
(1)Institute of Military Medicine, Nanjing Command, PLA.

Before 1986, tsutsugamushi disease was known only prevalent in south to Zhejiang Province in our country, belonged to the summer type and Leptotrombidium (L.) deliense was regarded as the main vector. In October 1986, the authors found this disease in Nanjing and carried out a series of studies in 1986-1992. The results were as follows: Tsutsugamushi disease was epidemic in Nanjing and north of Jiangsu (including: Dongtai, Haian, Rudong, Jinhu, Hanjiang, Jiangdu); belonged to the autumn-winter type; the main reservoir hosts were Apodemus agrarius, Rattus confucianus, R. norvegicus and Crocidura lasiura; the transmitting vector was L. (L.) scutellare; the pathogen of tsutsugamushi disease of the autumn-winter type-Rickettsia tsutsugamushi belonged to low-virulent strain, and could not easily be detected; after the inoculated mice were treated with diluted cyclophosphamide solution, 14 strains of R. tsutsugamushi have been isolated from rats, mites and patients; serological typing of their sera showed that they belonged to the Gilliam type; natural foci in Jiangsu could be divided into two types--flat land and hilly land.

PMID: 8082137 [PubMed - indexed for MEDLINE]

682. J Prev Med Public Health. 2010 Sep;43(5):436-44. doi:
10.3961/jpmph.2010.43.5.436.

[Correlations between climate change-related infectious diseases and meteorological factors in Korea].

[Article in Korean]

Kim SH(1), Jang JY.

Author information:
(1)Department of Preventive Medicine and Public Health, School of Medicine, Ajou University, Korea.

OBJECTIVES: Infectious diseases are known to be affected by climate change. We

investigated if the infectious diseases were related to meteorological factors in Korea.
METHODS: Scrub typhus, hemorrhagic fever with renal syndrome (HFRS), leptospirosis, malaria and Vibrio vulnificus sepsis among the National Notifiable Infectious Diseases were selected as the climate change-related infectious diseases. Temperature, relative humidity and precipitation were used as meteorological factors. The study period was from 2001 through 2008. We examined the seasonality of the diseases and those correlations with meteorological factors. We also analyzed the correlations between the incidences of the diseases during the outbreak periods and monthly meteorological factors in the hyper-endemic regions.
RESULTS: All of the investigated diseases showed strong seasonality; malaria and V. vulnificus sepsis were prevalent in summer and scrub typhus, HFRS and leptospirosis were prevalent in the autumn. There were significant correlations between the monthly numbers of cases and all the meteorological factors for malaria and V. vulnificus sepsis, but there were no correlation for the other diseases. However, the incidence of scrub typhus in hyper-endemic region during the outbreak period was positively correlated with temperature and humidity during the summer. The incidences of HFRS and leptospirosis had positive correlations with precipitation in November and temperature and humidity in February, respectively. V. vulnificus sepsis showed positive correlations with precipitation in April/May/July.
CONCLUSIONS: In Korea, the incidences of the infectious diseases were correlated with meteorological factors, and this implies that the incidences could be influenced by climate change.

DOI: 10.3961/jpmph.2010.43.5.436
PMID: 20959714 [PubMed - indexed for MEDLINE]

683. Trans R Soc Trop Med Hyg. 2007 Jul;101(7):686-90. Epub 2007 Apr 12.

Serological and blood culture investigations of Nepalese fever patients.

Blacksell SD(1), Sharma NP, Phumratanaprapin W, Jenjaroen K, Peacock SJ, White NJ, Pukrittayakamee S, Day NP.

Author information:
(1)Faculty of Tropical Medicine, Mahidol University, Bangkok, Thailand. stuart@tropmedres.ac

Serological testing of paired (i.e. admission and convalescent) sera from 103 fever patients in Kathmandu, Nepal, was performed to estimate the prevalence rates of scrub typhus, murine typhus, Leptospira and dengue virus antibodies and to determine their role in the cause of active infections. Blood cultures from 15 patients grew Salmonella enterica serovar Typhi, 8 grew S. Paratyphi A and 6 grew other bacteria. Diagnostic antibody levels were detected against murine typhus (27/103; 26%), scrub typhus (23/103; 22%), Leptospira (10/103; 10%) and dengue virus (8/103; 8%). Nineteen patients (18%) had diagnostically raised antibodies to more than one infectious agent. Seven S. Typhi (7/15; 47%) and two S. Paratyphi A (2/8; 25%) patients had significant scrub typhus, murine typhus, Leptospira or dengue virus IgM antibody titres. This study confirms the presence of leptospiral, rickettsial and dengue infections in Kathmandu as well as evidence for mixed infections with S. Typhi and Orientia tsutsugamushi or

Rickettsia typhi. These infections should be kept in mind when considering the differential diagnoses of fever and empirical treatment options in Nepal. Many patients demonstrated static IgM antibody results between paired serum collections, suggesting recent rather than acutely active infections.

DOI: 10.1016/j.trstmh.2007.02.015
PMID: 17433390 [PubMed - indexed for MEDLINE]

684. Kansenshogaku Zasshi. 1989 Mar;63(3):262-7.

[The first case of tsutsugamushi disease in Ehime Prefecture].

[Article in Japanese]

Matsumoto I, Shiroguchi T, Murakami S, Kobayashi Y, Kanemitsu N, Tange Y.

The first case of tsutsugamushi disease in Ehime Prefecture was experienced in December 1987 with successful isolation of the causative agent. The patient was taken ill twelve days after infection. Immunofluorescent antibody tests using the isolate, Yamazaki strain, and Gilliam, Karp, Kato, Irie and Shimokoshi strains as antigens revealed that the specific antibodies against these antigens appeared and increased in the blood of the patient during the course of the disease. And the antibody titers to the Yamazaki antigen were the highest of these antigens. Agglutinin for Proteus OXK did not appear in the blood of the patient. The immunofluorescent antibody test using type-specific monoclonal antibodies to Gilliam, Karp, Kato, Irie and Shimokoshi strains and these five strains and the Yamazaki strain as antigens revealed that the Yamazaki strain was identified as Karp type of Rickettsia tsutsugamushi.

PMID: 2504840 [PubMed - indexed for MEDLINE]

685. Korean J Anesthesiol. 2010 Dec;59 Suppl:S233-7. doi: 10.4097/kjae.2010.59.S.S233. Epub 2010 Dec 31.

A case of back pain caused by Salmonella spondylitis -A case report-.

Choi YS(1), Cho WJ, Yun SH, Lee SY, Park SH, Park JC, Jang EH, Shin HY.

Author information:
(1)Department of Anesthesiology and Pain Medicine, College of Medicine, Jeju National University, Jeju, Korea.

Salmonella spondylitis is a rare illness, and it generally occurs in patients who have already had sickle cell anemia, and it is even rarer in patients who are without sickle cell anemia. A 61-year-old male patient was hospitalized for the evaluation of his renal function and then treatment was started for his back pain. His back pain had developed about 2 months previously without any specific trauma. Only a bulging disc was detected on the initial lumbar MRI. Regarding his fever, it was diagnosed as possible atypical pneumonia, scrub typhus, etc., and multiple antibiotic therapy was administered. At the time of transfer, the leucocytes and hs-CRP were normal and the ESR was elevated. A diagnostic epidural

block was performed for his back pain, but his symptoms were not improved. Lumbar MRI was performed again and it showed findings of infective spondylitis. Salmonella D was identified on the abscess culture and so he was diagnosed as suffering from Salmonella spondylitis. After antibiotic treatment, his back pain was improved and the patient was able to walk.

DOI: 10.4097/kjae.2010.59.S.S233
PMCID: PMC3030045
PMID: 21286449 [PubMed]

686. J Hyg Epidemiol Microbiol Immunol. 1968;12(1):18-25.

Antigenic diversity of rickettsia tsutsugamushi: epidemiologic and ecologic significance.

Elisberg BL, Campbell JM, Bozeman FM.

PMID: 4975526 [PubMed - indexed for MEDLINE]

687. Ann N Y Acad Sci. 2006 Oct;1078:74-9.

Rickettsioses in Australia.

Graves S(1), Unsworth N, Stenos J.

Author information:
(1)Australian Rickettsial Reference Laboratory, The Geelong Hospital, PO BOX 281, Geelong, Victoria, Australia. Stephen.graves@hnehealth.nsw.gov.au

Australia, an island continent in the southern hemisphere, has a range of rickettsial diseases that include typhus group rickettsiae (Rickettsia typhi), spotted fever group rickettsiae (R. australis, R. honei), scrub typhus group rickettsiae (R. tsutsugamushi), and Q fever (C. burnetii). Our knowledge of Australian rickettsiae is expanding with the recognition of an expanded range of R. honei (Flinders Island spotted fever) to Tasmania and southeastern mainland Australia (not just on Flinders Island), and the detection of a new SFG species (or subspecies), tentatively named "R. marmionii" in the eastern half of Australia. This rickettsia causes both acute disease (7 cases, recognized so far) and is also associated (as a "R. marmionii" bacteriaemia) with patients having a chronic illness. The significance of the latter is under investigation. It may be a marker of autoimmune disease or chronic fatigue in some patients.

DOI: 10.1196/annals.1374.008
PMID: 17114682 [PubMed - indexed for MEDLINE]

688. Emerg Infect Dis. 2012 May;18(5):825-9. doi: 10.3201/eid1805.111563.

Unsuspected rickettsioses among patients with acute febrile illness, Sri Lanka, 2007.

Reller ME(1), Bodinayake C, Nagahawatte A, Devasiri V, Kodikara-Arachichi W, Strouse JJ, Flom JE, Østbye T, Woods CW, Dumler JS.

Author information:
(1)Johns Hopkins University School of Medicine, Baltimore, Maryland 21205, USA. mreller1@jhmi.edu

We studied rickettsioses in southern Sri Lanka. Of 883 febrile patients with paired serum samples, 156 (17.7%) had acute rickettsioses; rickettsioses were unsuspected at presentation. Additionally, 342 (38.7%) had exposure to spotted fever and/or typhus group rickettsioses and 121 (13.7%) scrub typhus. Increased awareness of rickettsioses and better tests are needed.

DOI: 10.3201/eid1805.111563
PMCID: PMC3358078
PMID: 22516455 [PubMed - indexed for MEDLINE]

689. Kansenshogaku Zasshi. 1988 Feb;62(2):135-40.

[Epidemiology of tsutsugamushi disease in Hokuriku District, Japan. 2. Seasonal observations of vectors and rickettsial pathogens at fixed points, associated with these distributions].

[Article in Japanese]

Takada N, Tada T, Kondo K, Akao N.

PMID: 3137292 [PubMed - indexed for MEDLINE]

690. Jpn J Infect Dis. 2006 Jun;59(3):204-5.

Epidemiological study of Japanese spotted fever and tsutsugamushi disease in Shimane Prefecture, Japan.

Tabara K(1), Hoshina K, Itagaki A, Katayama T, Fujita H, Kadosaka T, Yano Y, Takada N, Kawabata H.

Author information:
(1)Shimane Prefectural Institute of Public Health and Environmental Science, Matsue, Shimane 690-0122, Japan. tabara-kenji@pref.shimane.lg.jp

PMID: 16785708 [PubMed - indexed for MEDLINE]

691. Nihon Naika Gakkai Zasshi. 1993 Sep 10;82(9):1461-5.

[Changing pattern in rickettsia infections--with special reference to tsutsugamushi disease and spotted fever].

[Article in Japanese]

Tange Y, Kobayashi Y.

PMID: 8245645 [PubMed - indexed for MEDLINE]

692. Kansenshogaku Zasshi. 1989 Jun;63(6):654-8.

[The first case of tsutsugamushi disease in 20 years infected in a rural region of Osaka].

[Article in Japanese]

Asai S, Kawarano M.

Recently cases of tsutsugamushi disease have been reported in various areas in Japan. We met a case infected in a rural area, Sennan of Osaka prefecture. The patient suffered from high fever, left axillary lymphadenitis. At his left elbow an eschar was detected, so tsutsugamushi disease was suspected, despite no rash nor CRP elevation. By indirect immunofluorescence (IF) method, the diagnosis was confirmed. At an early stage (the fifth day after onset) he was followed by probable DIC, remitted successfully by administration of minocycline. For 20 years tsutsugamushi disease has not been reported in Osaka prefecture. This case is the second one and the first child case in Wakayama prefecture. Epidemiologic assessments may need to be investigated in Osaka and Wakayama Prefecture.

PMID: 2515227 [PubMed - indexed for MEDLINE]

693. Curr Probl Pediatr. 1981 Apr;11(6):1-38.

Rickettsial diseases and Rocky Mountain spotted fever--Part II.

Riley HD Jr.

PMID: 7016457 [PubMed - indexed for MEDLINE]

694. Kansenshogaku Zasshi. 1991 May;65(5):591-6.

[The first two cases of tsutsugamushi disease found in Toshima Island, Tokyo Metropolis].

[Article in Japanese]

Nagasaka S(1), Imagawa Y, Murata M.

Author information:
(1)Toshima National Health Insurance Clinic.

It has been known that tsutsugamushi disease, so-called "Shichito-fever", is

widely spread among the Izu Islands, Tokyo Metropolis. The cases were reported in Oshima Island, Niijima Island, Shikine Island, Kozu Island, Miyake Island, Mikura Island, and Hachijo Island previously, although no case has been reported in Toshima Island. In this paper, we report the first two cases of tsutsugamushi disease found in Toshima Island in December 1988 and December 1989. The first case was a 73 year-old male and the second case was a 83 year-old female, respectively. Fever, erythema and eschar were observed in both cases, while lymphadenopathy and hepatosplenomegaly were not detected. After tetracycline was administered, the fever immediately went down and erythema gradually disappeared in both cases. Specific immunofluorescence tests demonstrated that IgG antibody titers rose against Karp, Gilliam and Kato strains, and that IgM antibody titers rose only against Gilliam strain in both cases. Therefore, Gilliam-like strain of Rickettsia tsutsugamushi may play an important role in Toshima Island.

PMID: 1908885 [PubMed - indexed for MEDLINE]

695. Eur J Clin Microbiol Infect Dis. 1991 Feb;10(2):95-6.

A case of Tsutsugamushi disease probably contracted in Africa.

Osuga K, Kimura M, Goto H, Shimada K, Suto T.

PMID: 1907545 [PubMed - indexed for MEDLINE]

696. Bull Soc Pathol Exot Filiales. 1985;78(2):153-6.

[Serological approach to the occurrence of rickettsioses in the Central African Republic].

[Article in French]

Gonzalez JP, Fiset P, Georges AJ, Saluzzo JF, Wisseman CL Jr.

A serosurvey for evidence of human rickettsial infections was carried out in the Republic of Central Africa on 144 sera by indirect immunofluorescence (IIF) and microagglutination tests (MA). There was no serological evidence of epidemic typhus and only two sera were positive for murine typhus. Approximately 15% of the surveyed population was serologically positive by MA for R. conorii antibodies. However, 48% of this population had spotted fever group antibodies as detected by IIF but were negative in MA for R. conorii, R. rickettsii and R. akari antibodies. These sera with high titers in IIF and negative in MA lead us to believe that in Central Africa there are rickettsiae pathogenic for man that are related to the Spotted Fever group and are yet to be identified.

PMID: 3896545 [PubMed - indexed for MEDLINE]

697. Jpn J Infect Dis. 2002 Dec;55(6):197-203.

Evaluation of National Tsutsugamushi Disease Surveillance--Japan, 2000.

Matsui T(1), Kramer MH, Mendlein JM, Osaka K, Ohyama T, Takahashi H, Ono T, Okabe N.

Author information:
(1)Field Epidemiology Training Program Japan, National Institute of Infectious Diseases, Tokyo 162-8640, Japan. djyu@kaiju.medic.kumamoto-u.ac.jp

In Japan, Tsutsugamushi disease, which is caused by Orientia tsutsugamushi, is re-emerging with newly recognized strains and is now endemic in all prefectures except Hokkaido and Okinawa. We analyzed recent surveillance data to describe the epidemiology of Tsutsugamushi disease and to evaluate the newly implemented national surveillance system according to the CDC guidelines for evaluating surveillance systems. In 2000, 756 cases of Tsutsugamushi disease were reported from 37 of 47 prefectures; two of these cases were fatal. The median age of case-patients was 64 years (range: 2 - 94 years); 414 (54.8%) were male. In northern Japan, most cases were diagnosed in the months of May through July and in the months of October through December, and in southern Japan, cases were diagnosed almost year-round with a peak from October through December and in January. Reporting and transfer of surveillance information from the prefecture to the national level was effective and timely, but the completeness and quality of case reporting could still be improved. The current system for Tsutsugamushi disease surveillance is useful for describing epidemiologic patterns by time, prefecture, and demographic characteristics. However, collection of additional information on suspected place of transmission, activity performed at the place of transmission, or the case-patient's profession would likely make the system more valuable for outbreak detection and for better defining populations at risk.

PMID: 12606829 [PubMed - indexed for MEDLINE]

698. Kansenshogaku Zasshi. 1995 Oct;69(10):1118-25.

[Epidemiological study of tsutsugamushi disease in Gunma prefecture. A special field study and serotype].

[Article in Japanese]

Wakai K(1), Funada K, Nakajima T, Kanazawa K, Hara Y, Tanaka N, Abe O, Otsuki K.

Author information:
(1)Gunma Prefectural Institute of Public Health and Environmental Sciences.

An epidemiologically investigated of invasion of Rickettsia tsutsugamushi, inhabitant of mites and serum sample from patients with Tsutsugamushi disease in Gunma prefecture from 1984 to 1994 was made. Our data clearly indicated that Rickettsia tsutsugamushi was not located but widely spreaded throughout the Prefecture. Mites on rodents, were classified into 4 genus and 12 species and about 15% of them were Leptotrombidium pallidum and Leptotrombidium scuttellare, well known virulent vectors. The highest incidence rate of this disease was observed in the northwest area of the Prefecture from October to December, while a smaller number of patients occurred in other areas and in other months. About

fifty percent of the serum samples from the patients were positive to the Karp strain. These results suggest that the major cause of this disease is the Karp strain and the disease could occur potentially in various areas of the Prefecture.

PMID: 7499914 [PubMed - indexed for MEDLINE]

699. Kansenshogaku Zasshi. 1980 May;54(5):235-41.

[Shichito fever in Izu Shichito Islands. 1. Outbreak of Shichito fever and isolation of R. tsutsugamushi from a patient, field rodents and trombiculid mites (author's transl)].

[Article in Japanese]

Murata M, Nogami S, Shirasaka A, Tanaka H, Kawamura A Jr, Miyairi T.

PMID: 6772713 [PubMed - indexed for MEDLINE]

700. Trop Med Int Health. 1998 Mar;3(3):242-8.

Electron-microscopic examination of Rickettsia tsutsugamushi-infected human liver.

Pongponratn E(1), Maneerat Y, Chaisri U, Wilairatana P, Punpoowong B, Viriyavejakul P, Riganti M.

Author information:
(1)Department of Tropical Pathology, Faculty of Tropical Medicine, Mahidol University, Bangkok, Thailand.

A 33 year-old Thai woman was diagnosed with scrub typhus infection according to clinical symptoms, eschar lesions compatible with the disease, and specific antibody to Rickettsia tsutsugamushi detected by indirect immunoperoxidase. Percutaneous transhepatic needle biopsies were taken before and 7 days after treatment with tetracycline to study the pathology of the liver. The liver tissue was evaluated by light microscopy, using H & E and Pinkerton's stains, and by transmission electron microscopy (TEM). Before treatment it showed reactive hepatitis. Rickettsia organisms within the hepatocytes and sinusoids detected by Pinkerton's stain appeared as tiny bright-red organisms. By TEM, the rod-shaped double-membrane Rickettsiae appeared intact in the cytoplasm of Kupffer's cells and hepatocytes. After tetracycline treatment, moderate levels of acidophilic and ballooning liver cells were observed. The degree of cytoplasmic organelle damage varied, including fatty metamorphosis, depletion of glycogen granules, loss of the mitochondrial cristae, dilatation of endoplasmic reticulum and cytoplasmic vacuolation. Rickettsia organisms cannot be visualized by Pinkerton's stain but were detected by TEM, in markedly vacuolated hepatocytes, in congested sinusoids and in Kupffer's cells. Intranuclear Rickettsia were discovered in the endothelial nucleus, showing various degrees of injury. Some were mildly degenerated, while others exhibited clumping of nucleoprotein at the cytoplasm

periphery and large vacuolation centrally. Many indented organisms were found, and binary fission during Rickettsiae multiplication was always affected. Electron-microscopic examination of hepatic injury associated with scrub typhus is rare. This is the first ultrastructural localization of Rickettsiae in the infected human liver.

PMID: 9593364 [PubMed - indexed for MEDLINE]

701. Acta Virol. 1991 Nov;35(6):580-6.

A ten years experience on diagnosis of rickettsial diseases using the indirect immunoperoxidase methods.

Suto T(1).

Author information:
(1)Department of Microbiology, Akita University School of Medicine, Japan.

An accurate diagnosis of Tsutsugamushi disease (TD) can be made within a few hours after receiving serum specimens by detecting the specific IgG and IgM antibodies in the patient's serum. Using the indirect immunoperoxidase test we found a total of 730 cases of TD in 32 out of 47 prefectures when serum samples from 2,224 cases of suspected disease or fever of unknown origin or from patients with febrile exanthema of unknown cause were examined during a 10 years period (from May 1980 till December 1989). In addition, 27 cases of spotted fever group rickettsial infection were confirmed and one case of African spotted fever group infection was also detected in 1988. Furthermore, possibly the first case of TD was found in People's Republic of Congo (Africa) in 1989.

PMID: 1687642 [PubMed - indexed for MEDLINE]

702. Nihon Naika Gakkai Zasshi. 1990 Mar 10;79(3):275-9.

[Epidemiologic study and diagnosis of endemic Rickettsia infections of tsutsugamushi and spotted fever].

[Article in Japanese]

Sutou T.

PMID: 2332681 [PubMed - indexed for MEDLINE]

703. Emerg Infect Dis. 2005 Feb;11(2):237-44.

Spotted fever group and typhus group rickettsioses in humans, South Korea.

Choi YJ(1), Jang WJ, Ryu JS, Lee SH, Park KH, Paik HS, Koh YS, Choi MS, Kim IS.

Author information:
(1)Konkuk University, Choongbuk, Republic of Korea.

Comment in
Emerg Infect Dis. 2006 Mar;12(3):531-2.
Emerg Infect Dis. 2006 Mar;12(3):531.

The presence of the nucleic acid of the spotted fever group (SPG) and typhus group (TG) rickettsiae was investigated in 200 serum specimens seropositive for SFG rickettsiae by multiplex-nested polymerase chain reaction with primers derived from the rickettsial outer membrane protein B gene. The DNA of SFG, TG, or both rickettsiae was amplified in the 24 serum specimens, and sequence analysis showed Rickettsia conorii, R. japonica, and R. felis in the specimens. R. conorii and R. typhi were found in 7 serum specimens, which indicated the possibility of dual infection in these patients. These findings suggest that several kinds of rickettsial diseases, including boutonneuse fever, rickettsialpox, R. felis infection, and Japanese spotted fever, as well as scrub typhus and murine typhus, are occurring in Korea.

DOI: 10.3201/eid1102.040603
PMCID: PMC3320442
PMID: 15752441 [PubMed - indexed for MEDLINE]

704. J Dermatol. 1992 Apr;19(4):229-33.

Tsutsugamushi disease in the central part of Japan.

Kanno Y(1), Taniguchi Y, Sakamoto T, Shimizu M, Ando K.

Author information:
(1)Department of Dermatology, Mie University School of Medicine, Tsu, Japan.

Two cases of tsutsugamushi disease misdiagnosed initially as drug eruption were reported. Clinical symptoms of both cases disappeared dramatically after starting minocycline. Statistical examinations were performed on 29 cases of tsutsugamushi disease, including those observed in Mie Prefecture since 1982. Half of them were seen in the last 2 years. The onset was predominantly recorded in November (59%). Presumptive sites of infection were forests (63%) and fields (21%). No patients were infected along river-banks.

PMID: 1607485 [PubMed - indexed for MEDLINE]

705. Microbiol Immunol. 2001;45(6):439-46.

Epidemiological survey of Orientia tsutsugamushi distribution in field rodents in Saitama Prefecture, Japan, and discovery of a new type.

Tamura A(1), Yamamoto N, Koyama S, Makisaka Y, Takahashi M, Urabe K, Takaoka M, Nakazawa K, Urakami H, Fukuhara M.

Author information:

(1)Department of Microbiology, Niigata College of Pharmacy, Japan.
tamura@niigata-pharm.ac.jp

Erratum in
Microbiol Immunol. 2006;50(5):419.

There are various antigenic variants of Orientia tsutsugamushi which are distinguished by immunological and molecular genetic methods targeted at the antigenic diversity of 56-kDa type-specific antigen proteins. The present study was performed to analyze 15 strains successfully isolated from rodents in Saitama Prefecture, Japan, by 56-kDa gene sequence homologies, reactivities with type-specific monoclonal antibodies and polymerase chain reaction (PCR) using type-specific primer-pairs. We demonstrated the presence of a new type of O. tsutsugamushi among the isolates. This new type, designated as the Saitama type, was located in the branch of Karp type in the phylogenetic tree based on 56-kDa gene sequences, but distant from the known Karp types, such as Karp, JP-1 and JP-2, showing less than 90% homology. Strains of this type could not be distinguished by immunological methods from Karp type strains, but a new primer-pair for PCR which specifically amplifies the DNA of this new type strain was designed. This primer-pair may serve to find this strain type in future studies.

PMID: 11497219 [PubMed - indexed for MEDLINE]

706. Kisaengchunghak Chapchi. 1992 Dec;30(4):341-8.

Study on vector mites of tsutsugamushi disease in Cheju Island, Korea.

Ree HI(1), Lee IY, Cho MK.

Author information:
(1)Department of Parasitology, College of Medicine, Yonsei University, Seoul, Korea.

Because no reference on trombiculid mites (Acarina: Trombiculidae) in Cheju Island where tsutsugamushi disease is highly endemic had been available, studies on trombiculid mites in Cheju Island were implemented during the period of August 1991-April 1992, and the results obtained are summarized as follows: (1) The species and numbers of the field rodents collected were 143 Apodemus agrarius chejuensis (92.3%), 11 Crocidura lasiura (7.1%) and 1 Micromys minutus (0.6%). From total 12,075 chiggers harvested, 9 species of 4 genera in Trombiculidae were identified. (2) The predominant species through all seasons was L. zetum (43.3%), followed by L. orientale (27.4%) and L. scutellare (26.6%). However, in autumn when the most cases of tsutsugamushi disease occur, L. scutellare was prominently predominant, having 79.8% of the collected chiggers. (3) Among 1,142 L. scutellare examined for Rickettsia tsutsugamushi by means of IFA test, 6 individuals were found positive showing 0.5% of infection rate. This is the first finding that L. scutellare is the second vector species of tsutsugamushi disease in Korea. (4) Antibody positive rate of A. agrarius chejuensis sera were 31.2% (44/139), and 1 M. minutus serum was also found positive. The seropositive rates by season were not so significantly different.

PMID: 1297424 [PubMed - indexed for MEDLINE]

707. Nihon Saikingaku Zasshi. 1979 Mar;34(2):375-93.

[Current remarks on Rickettsiae and rickettsiosis--studies on virulence and prevalence of Tsutsugamushi disease Rickettsia (author's transl)].

[Article in Japanese]

Kawamura A.

PMID: 114685 [PubMed - indexed for MEDLINE]

708. Nihon Naika Gakkai Zasshi. 1985 Dec;74(12):1652-7.

[Studies on epidemiological and diagnostic problems in tsutsugamushi disease. With special reference to 9 cases occurring in Gifu Prefecture, Japan].

[Article in Japanese]

Kasuya S, Hirano A, Iwata T, Hamano H, Tsunekawa J, Shigemura M, Shimada E, Izumi K, Hioki A.

PMID: 3938471 [PubMed - indexed for MEDLINE]

709. Bull Soc Pathol Exot Filiales. 1971 Jan-Feb;64(1):30-7.

[Rickettsia tsutsugamushi: current studies of the vectors and the antigens].

[Article in French]

Capponi M, Kawai K.

PMID: 4997680 [PubMed - indexed for MEDLINE]

710. J Med Entomol. 1990 Jul;27(4):501-8.

Trombiculid mites (Acari: Trombiculidae) and Rickettsia tsutsugamushi isolated from wild rodents in a new endemic area of Japan.

Iwasa M(1), Kasuya S, Noda N, Hioki A, Ito A, Ohtomo H.

Author information:
(1)Department of Parasitology, Gifu University School of Medicine, Japan.

Investigations of trombiculid mites and Rickettsia tsutsugamushi in wild rodents

were made in southern Gifu Prefecture where patients infected with tsutsugamushi disease recently have been found. A total of 16,396 trombiculid mites, consisting of 10 species from three genera, was collected from 170 Apodemus speciosus in two locations. Kani-Sakahogi and Kuze. Leptotrombidium scutellare (Nagayo et al.) (44.0%) was most predominant, followed by L. pallidum (Nagayo et al.) (26.9%); L. fuji (Kuwata et al.) (13.6%); and Gahrliepia saduski Womersley (14.2%). These four species constituted the bulk of the chigger mite fauna. L. scutellare was present from October to February with a remarkably high peak in November, whereas pallidum occurred from November to March with the highest peak in December. L. fuji and G. saduski showed their highest peaks in December and moderate peaks in early summer (April and May). Positive identification of Rickettsia tsutsugamushi in wild rodents from Kani-Sakahogi were found to be 50 and 58.3% in November 1985 and 1986, respectively, and 38.5% in November 1986 from Kuze. R. tsutsugamushi was isolated from chigger mites of an L. pallidum-rich group, displaying the highest titer to Karp strain. Serological investigation of rodents to R. tsutsugamushi antibodies were calculated as 41.6 and 50% positive in November 1985 and 1986 in Kani-Sakahogi, respectively, and 50% in November 1986 in Kuze. The Karp strain was dominant in specificity to antibodies. These results indicate that the surveyed areas have a high probability of occurrence of tsutsugamushi disease, and L. scutellare and L. pallidum may serve as the vectors in these areas. Particularly, we suggest that L. scutellare is the most important vector which has caused a recent outbreak of this disease in southern Gifu Prefecture.

PMID: 2117663 [PubMed - indexed for MEDLINE]

711. Cutis. 2006 Jun;77(6):350-2.

What's eating you? Chiggers.

Elston DM(1).

Author information:
(1)Department of Dermatology, Geisinger Medical Center, 100 N Academy Ave, Danville, PA 17821, USA. dmelston@geisinger.edu

PMID: 16838766 [PubMed - indexed for MEDLINE]

712. Zhonghua Yu Fang Yi Xue Za Zhi. 1980 Feb;14(1):43-5.

[Epidemiologic survey of Tsutsugamushi disease: analysis of 46 cases (author's transl)].

[Article in Chinese]

Li G.

PMID: 7227083 [PubMed - indexed for MEDLINE]

713. Am J Trop Med Hyg. 2001 Nov;65(5):528-34.

Determination and geographical distribution of Orientia tsutsugamushi serotypes in Korea by nested polymerase chain reaction.

Ree HI(1), Kim TE, Lee IY, Jeon SH, Hwang UW, Chang WH.

Author information:
(1)Institute of Tropical Medicine, College of Medicine, Yonsei University, Seoul, Korea.

Field rodents and chigger mites were collected at 30 locations in Korea in October and November 1997-1999 to determine the serotypes of Orientia tsutsugamushi and their geographical distribution. A nested polymerase chain reaction was performed with the spleen tissues from 546 field-striped mice (Apodemus agrarius) and 104 pools of chigger mites. The positivity rate of O. tsutsugamushi was 45.6% in A. agrarius and 39.4% in the chigger mite pools. Two serotypes, Boryong and Karp, were found in these samples; the former was predominant (78.3% in the mice and 82.9% in the chigger mite pools), with wide distribution throughout the country, including Cheju-do. The latter was confined to the middle of the Korean peninsula, with positivity rates of 15.7% in the mice and 12.2% in the chigger mite pools. The double infection of Karp and Boryong serotypes was found in 15 (6.0%) A. agrarius mice. Gilliam serotype was not detected at any of the study locations. The Boryong and Kuroki serotypes were identical in amino acid sequence of the 56-kDa protein, although they differed in virulence to BALB/c mice.

PMID: 11716109 [PubMed - indexed for MEDLINE]

714. Kansenshogaku Zasshi. 2003 Feb;77(2):60-7.

[Tsutsugamushi disease found in the northern districts of Awaji Island--epidemiological study of the outbreak season temperature].

[Article in Japanese]

Okada N(1).

Author information:
(1)Okada Clinic of Internal Medicine.

Ten cases of tsutsugamushi disease which were diagnosed and treated among the outpatients from 1987 through 1999 in a clinic of internal medicine locating at the northern districts of Awaji Island were investigated. The patients were consisted of 8 males and 2 females and their ages ranged from 6 to 69 years old. The infected areas were distributed in hilly districts extending over three towns. The outbreak season was from November through December. The following two facts seemed to be worthy of notice. 1) There was a time lag on the patient's onset among the different years. 2) When the incidences were more than two cases a year, the time difference of onset was within a few days each other. Then the relationship between atmospheric temperature and the date of onset was studied. Meterological observing records at Gunge Ichinomia Town near the northern

districts of Awaji Island were investigated into the temperature every 10 days from 1987 to 1999. Average temperature every 10 days including the presumable day when each patient was infected with Orientia tsutsugamushi (O.T) ranged between 9.1 degrees C and 13.3 degrees C and in 7 of 10 cases ranged between 11.6 degrees C and 13.3 degrees C. The maximum temperature ranged between 11.3 degrees C and 17.5 degrees C, the minimum one between 6.6 degrees C and 9.1 degrees C. It is likely that the seasonal occurrence of tsutsugamushi disease greatly depends upon the optimum temperature for the activity of tsutsugamushi mites in these areas. The high titer of serum IgM and IgG antibody to Karp, Gilliam Kato type O. tsutsugamushi was detected by an immunofluorescense assay (IF) in all cases. IgG antibody, titers fell drastically to 20 or 10 times within a few years but 10 times titer was still detected in three cases in more than ten years. It is suggested that IgG antibody titer more than 10 times in healthy people indicates the infection in the past and titer more than 20 times implies the infection during the past few years.

PMID: 12661080 [PubMed - indexed for MEDLINE]

715. J Dermatol. 2000 Nov;27(11):724-9.

Evaluation on the occurrence of Tsutsugamushi disease in Tottori Prefecture.

Rivera MR(1), Mihara M, Shibata M.

Author information:
(1)Department of Dermatology, Faculty of Medicine, Tottori University, 36-1, Nishimachi, Yonago, 683-8504, Japan.

In Tottori Prefecture, we gathered the data of all patients known to have been infected with tsutsugamushi disease (TD) over a span of 49 years, from 1950 to 1998. The total number of patients reported so far has been 19, 16 of which occurred after 1988. Recently, it has been observed that the incidence tended to increase. Of the 13 laboratory confirmed patients, 7 came from Saji Village and Chizu Town, in the Yazu district, a place whereby, a body of water known as the Sendai River partly passes through, and is located in the eastern region of Tottori Prefecture. The remaining 6 came from Nichinan Town in the Hino district, a locality whereby the Hino River partly passes through; in the western part of the same prefecture. Both are mountainous areas, where corniferous trees such as Japanese cedars and pines grow. These results indicate that the occurrence or prevalence of TD in Tottori Prefecture was restricted only to particular places. Seasonal occurrence was during April and May in Nichinan, while October and November in Yazu, particularly in Saji, with one exceptional case in Chizu in which TD occurred during spring. It is likely that the causative chigger mite is the Leptotrombidium pallidum (L. pallidum). The causative agent in Yazu was the Gilliam strain only of Orientia tsutsugamushi (O. tsutsugamushi), while in the latter, it was the Karp, Kato or some unknown strains with antigen(s) common to the Gilliam, Karp and Kato strains. The seasonal difference in the occurrence among the three places may be caused by the difference in temperature because the average autumn/spring temperature difference in the 2 districts was 2-3 degrees C lower in Nichinan than in the Saji-Chizu areas. As a result, the activity of larval chigger mites may be temporarily halted or stopped by the relatively lower temperature in Nichinan during autumn.

PMID: 11138539 [PubMed - indexed for MEDLINE]

716. Kansenshogaku Zasshi. 1992 Mar;66(3):306-13.

[Tsutsugamushi disease found in Haruna District, Gunma Prefecture--evaluations of the clinical features and the outbreak pattern].

[Article in Japanese]

Utsugi T(1), Ohta N, Nakano M, Kasahara K, Takada N.

Author information:
(1)Department of Internal Medicine, Haruna Hospital, Gunma, Japan.

From November to December in 1990, 7 cases of tsutsugamushi disease were found first in the southern foot of Mt. Haruna of Gunma Pref., Japan. The present study was conducted to clarify the clinical features and the outbreak pattern of rickettsial infection in this area. All the patients consisting of 6 males and 1 female farmers were admitted to our hospital, complaining of high fever, chills and skin rash on 5-12 days after working in the field. Based on laboratory examinations and one (or two) typical eschar, a tentative diagnosis of tsutsugamushi disease was made and all patients became better soon after the therapy with intravenous administration of minocyclin (200 mg/day). The high titer of serum antibody to Karp type Rickettsia (Orientia?) tsustugamushi was detected by an immunoperoxidase test (IP) in most of the patients, and also a Karp-like strain was isolated from only one patient, probably due to low virulence to mice. The agricultural areas along the Agatuma and Nakuta rivers in the northern foot of Mt. Haruna have been well known as the most endemic foci of the disease in Gunma Pref. Nevertheless, it was suggested that outbreaks of the disease might be potentially wide-spread throughout this Pref., when the prevalences of the disease were evaluated as incidences to a hundred thousand inhabitants or adjusted by the density of agricultural populations of each administrative divisions. As most of the patients in Gunma Pref. have been officially reported in autumn, a statistical trial indicates that there is a significant correlation between the levels of temperature and outbreaks of the disease in autumn.(ABSTRACT TRUNCATED AT 250 WORDS)

PMID: 1624819 [PubMed - indexed for MEDLINE]

717. Zhonghua Liu Xing Bing Xue Za Zhi. 2000 Aug;21(4):283-6.

[Investigation on natural foci of autumn-winter type tsutsugamushi disease in Shandong province].

[Article in Chinese]

Yang Z(1), Liu Y, Yu X, Wu Q, Xing R.

Author information:

(1)Department of Epidemiology, Military Medical Research Institute of Jinan Command, Jinan 250014, China.

OBJECTIVE: In order to provide basic data of tsutsugamushi disease in Shandong province, for the control and prevention of this disease in residents and army-men.
METHODS: Aetiological, serological and epidemiological methods were used to investigate the natural foci of autumn-winter type tsutsugamushi disease.
RESULTS: The peak of tsutsugamushi disease epidemic in Shandong province was in October with cases in this month accounted for 80% of all in a year, that showed autumn-winter type. The main reservoir hosts were Apodemus agrarius, Rattus norvegicus and Cricetulus triton, accounted for 82.87%, 85.47% and 13.04% respectively. It was confirmed that natural infection existed among Leptotrombidium (L.) scutellare, L. palpalis, L. linhuaikonense and Walchia pacifica. The main vector of transmission was Leptotrombidium scutellare. 41 strains of Rickettsia tsutsugamushi were isolated from the blood samples of patients, rodents and chigger mites but with weak toxicity. 90% of the serotypes of the strains belonged to Gilliam type. The distribution of the cases had a sporadic nature. More than 80% of the cases were young or middle aged peasants. Clinical symptoms were mild, with some differences in different areas.
CONCLUSION: The natural foci type of tsutsugamushi disease in Shandong could be divided into plain and hilly types.

PMID: 11860801 [PubMed - indexed for MEDLINE]

718. Zhonghua Liu Xing Bing Xue Za Zhi. 2006 Dec;27(12):1061-4.

[Genotype identification and sequence analysis of Orientia tsutsugamushi isolated from Shandong area].

[Article in Chinese]

Yang LP(1), Zhao ZT, Liu YX, Feng YQ, Wang XJ, Li Z.

Author information:
(1)Department of Epidemiology and Health Statistics, School of Public Health, Shandong University, Jinan 250012, China.

OBJECTIVE: To determine genotype, nucleotide sequence homology and phylogenesis of Orientia tsutsugamushi isolated from Shandong, China.
METHODS: Orientia tsutsugamushi isolated from patients, Apodemus agrarius and Leptotrombidium scutellare in Shandong area were identified by nested-PCR. On the basis of the nucleotide sequence of the gene that encoding the Ot M, 56 x 10(3) antigen, the primers were frequently used in Japan and Korea. Nucleotide sequences of three isolates were determined. The DNA sequences were compared with nucleotide sequences of Orientia tsutsugamushi registered in GenBank for sequence homology analysis. Phylogenetic analysis of the isolates and'some published sequences was carried out with Neighbor-joining method by MEGA 3.1 software.
RESULTS: 481- 507 bp DNA fragments encoding Orientia tsutsugamushi M, 56 x 10(3) protein were amplified successfully in the samples of Gilliam, Karp, Kato and Shandong isolates by group-specific primers. The corresponding target fragments of the three international reference strains of Gilliam, Karp, and Kato were

amplified successfully with each of their own type specific primers. 523 bp DNA fragments were amplified successfully from Shandong isolates by the nPCR with Kawasaki-specific primer, and no DNA fragment was amplified by the nPCR with Gilliam, Karp, Kato, Kuroki and Saitama-specific primer. Comparing with the sequences of Orientia tsutsugamushi registered in GenBank, all the Shandong isolates shared higher than 95% nucleotide sequence homology with Kawasaki strain founded in Japan. Data from phylogenetic analysis showed that Shandong isolates belonged to the same branch with Kawasaki strain.
CONCLUSION: To facilitate international comparison and communication, the primers should be employed in the Orientia tsutsugamushi research in China. Orientia tsutsugamushi isolated in China were similar to Kawasaki strain

PMID: 17415986 [PubMed - indexed for MEDLINE]

719. Curr Opin Infect Dis. 2011 Oct;24(5):457-63. doi: 10.1097/QCO.0b013e32834a1bd2.

Travel-associated zoonotic bacterial diseases.

Leshem E(1), Meltzer E, Schwartz E.

Author information:
(1)Center for Geographic Medicine and Internal Medicine C, Chaim Sheba Medical Center, Tel Hashomer, Israel.

PURPOSE OF REVIEW: Bacterial zoonoses are increasingly described in association with travel. Some bacterial zoonoses constitute important causes of post-travel illness. We focus on leptospirosis and rickettsiosis - the most common travel-associated bacterial zoonoses.
RECENT FINDINGS: Leptospirosis is regarded to be the most common zoonotic disease worldwide. In industrialized countries recreational exposures, both domestic and overseas, are increasingly becoming a major source of infection. Asymptomatic infection is rare among travelers. Rickettsial diseases account for approximately 1.5-3.5% of febrile travelers. In several series of travel-related rickettsioses, the most common travel-related rickettsial disease is Rickettsia africae. Other rickettsioses including Q fever, scrub typhus and murine typhus are considered rare among travelers. Whereas timely diagnosis of both diseases is still based on exposure history, antigen detection tools to aid the diagnosis during the acute illness are under research and far from being available. Due to these constrains, currently, the true incidence of both diseases is probably underestimated.
SUMMARY: Both leptospirosis and spotted fever may be rapidly fatal. Empiric doxycycline in severely ill febrile travelers should be considered. There is an urgent need for widely available antigen detection diagnostic tools to improve the detection of leptospirosis and rickettsial infections during the acute illness.

DOI: 10.1097/QCO.0b013e32834a1bd2
PMID: 21788890 [PubMed - indexed for MEDLINE]

720. Kansenshogaku Zasshi. 1997 May;71(5):464-7.

[A case of Tsutsugamushi disease as an imported infection].

[Article in Japanese]

Ueda S(1), Yumisashi T, Yoshida K, Maeda T, Karasuno T, Teshima H, Hiraoka A, Nakamura H, Masaoka T.

Author information:
(1)Fifth Department of Internal Medicine, Osaka Medical Center for Cancer and Cardiovascular Diseases.

Tsutsugamushi disease is widely spread throughout Japan. A case of tsutsugamushi disease was seen in October, 1996. A 64-year-old male developed typical symptoms of tsutsugamushi disease with Rickettsia tsutsugamushi, after he returned to Japan from Cheju Island, Korea. Not only in Japan but also in other Asian countries including Korea, China, Taiwan, and Thailand, tsutsugamushi disease is one of the most important rickettsial diseases carried by ticks or mites. If a traveller returning from an Asian country has symptoms such as high fever, skin eruption, and lymphadenitis, we should suspect that he is suffering from tsutsugamushi disease and should search if he has an eschar on any area of his body. We should not forget that tsutsugamushi disease is an imported disease. Patients of tsutsugamushi disease often have hematological disorders. They are sometimes referred to the hematological section of the hospital. Hematologists should be familiar with this disease.

PMID: 9209129 [PubMed - indexed for MEDLINE]

721. Kansenshogaku Zasshi. 1994 Nov;68(11):1433-6.

[A case of severe tsutsugamushi disease without eruption].

[Article in Japanese]

Nakagawa Y(1), Huruie H, Satou H, Matsumoto Y.

Author information:
(1)Department of Internal Medicine, Minamata Medical Center.

A 64-year-old male was admitted to our division because of fever. After admission, the patient was given beta-lactam antibiotics intravenously because he had no eruption and eschar. However, the fever continued, and he became unconsciousness and DIC appeared. We diagnosed the patient as Tsutsugamushi disease from indirect fluorescent antibody technique. Minocycline was excellently effective. Several reports of Tsutsugamushi disease without eruption have been given, so we must always be careful of Tsutsugamushi disease.

PMID: 7829913 [PubMed - indexed for MEDLINE]

722. Scand J Infect Dis. 2000;32(1):101-2.

Pericarditis due to Tsutsugamushi disease.

Chang JH(1), Ju MS, Chang JE, Park YS, Han WS, Kim IS, Chang WH.

Author information:
(1)Department of Internal Medicine, Ewha Womans University, Seoul, Korea.

Tsutsugamushi Disease is an acute febrile illness caused by Rickettsia tsutsugamushi, which enters into the human bloodstream through the bite of leptotrombidium. It is characterized by eschar, fever and cutaneous rash. Pericardial effusion in Tsutsugamushi Disease is not a common manifestation, although a high rate of effusion was reported in autopsy in those who had died of the disease. Here, we report a case of Tsutsugamushi pericarditis documented by indirect immunofluorescent test of pericardial fluid, and give a brief review of the literature.

PMID: 10716090 [PubMed - indexed for MEDLINE]

723. Jpn Circ J. 1991 Feb;55(2):149-53.

Review of a case of tsutsugamushi disease showing myocarditis and confirmation of Rickettsia by endomyocardial biopsy.

Yotsukura M(1), Aoki N, Fukuzumi N, Ishikawa K.

Author information:
(1)Second Department of Internal Medicine, Kyorin University School of Medicine, Tokyo, Japan.

A patient suffering from tsutsugamushi disease underwent endomyocardial biopsy for the purpose of diagnosis of myocarditis. Proliferation of Rickettsia tsutsugamushi was observed in the vascular endothelial cells of the myocardium. There have been no reports describing the identification of Rickettsia tsutsugamushi endomyocardial biopsy. This report indicates that endomyocardial biopsy may be a useful adjunct to the clarification of myocardial involvement of Rickettsia tsutsugamushi.

PMID: 1902270 [PubMed - indexed for MEDLINE]

724. Kansenshogaku Zasshi. 1985 May;59(5):496-9.

[First case of tsutsugamushi disease in Fukui Prefecture with isolation of Rickettsia from its donated blood].

[Article in Japanese]

Takada N, Ohtsuki N.

PMID: 3932538 [PubMed - indexed for MEDLINE]

725. Ryoikibetsu Shokogun Shirizu. 1995;(7):109-11.

[Rickettsiosis].

[Article in Japanese]

Hosoi H(1), Miyake K.

Author information:
(1)First Department of Medicine, Teikyo University School of Medicine, Tokyo, Japan.

PMID: 8749430 [PubMed - indexed for MEDLINE]

726. Internist (Berl). 1990 Apr;31(4):291-4.

[Fever, pericardial and pleural effusion in a 27-year-old patient following a stay in Thailand].

[Article in German]

Bosch T(1), Hacker H, Höfling B, Löscher T.

Author information:
(1)Medizinische Klinik I, Ludwig-Maximilians-Universität München.

PMID: 2354903 [PubMed - indexed for MEDLINE]

727. J Leukoc Biol. 1984 Apr;35(4):385-96.

Macrophages in resistance to rickettsial infections: protection against lethal Rickettsia tsutsugamushi infections by treatment of mice with macrophage-activating agents.

Nacy CA, Meltzer MS.

Peritoneal macrophages of BALB/c and C3H/HeN mice activated in vivo by intraperitoneal inoculation of viable Mycobacterium bovis strain BCG or the nonliving macrophage-activating agent Propionibacterium acnes (Corynebacterium parvum), were resistant to infection with Rickettsia tsutsugamushi, and they killed bacteria that did gain entry into the intracellular environment of these cells. This macrophage resistance to infection and intracellular destruction of rickettsiae was dependent upon development of an immune response to the activating agents, since macrophages elicited by sterile inflammatory agents failed to display either microbicidal activity unless cells were exposed to factors present in lymphokine-rich culture fluids from antigen or mitogen stimulated spleen cells (LK) in vitro. C3H/HeN mice that had been treated with activating agents, but not sterile inflammatory irritants, also survived intraperitoneal inoculation of up to 10(4) R. tsutsugamushi. This nonspecific

protection required the chronic presence of activated macrophages: acute immune response induced by intraperitoneal injection of PPD into mice inoculated intradermally with BCG, or intraperitoneal inoculation of conconavalin A, were not sufficient to induce survival of rickettsial disease, although macrophages from these animals were activated to kill rickettsiae at the time of challenge. The critical nature of activated macrophages in nonspecific protection against rickettsial infection was demonstrated with the macrophage-defective C3H/HeJ mice. These mice are equally as susceptible as C3H/HeN mice to intraperitoneal inoculation of R. tsutsugamushi, but do not develop activated macrophages in response to BCG infection, and are not protected against lethal rickettsial challenge following BCG treatment.

PMID: 6584528 [PubMed - indexed for MEDLINE]

728. Retina. 2008 Oct;28(8):1166-9.

Diagnostic and therapeutic challenges.

Han SB, Kim JH, Kim SJ, Yu YS, Cunningham ET Jr.

PMID: 18788103 [PubMed - indexed for MEDLINE]

729. Nihon Naika Gakkai Zasshi. 2006 Dec 10;95(12):2544-6.

[Tsutsugamushi disease with hemophagocytosis complicated by Parvovirus B19 infection].

[Article in Japanese]

Miyakawa K(1), Ohsugi K, Sugahara S, Kuriyama C, Kikuchi A, Ohta M.

Author information:
(1)Ohtanishinouchi Hospital, Department of General Internal Medicine, Koriyama.

PMID: 17240885 [PubMed - indexed for MEDLINE]

730. J Gastroenterol Hepatol. 2005 Jun;20(6):969-71.

Direct cytopathic liver injury and acute respiratory distress syndrome associated with gilliam-type tsutsugamushi disease.

Watanabe H, Saito T, Misawa K, Suzuki A, Sanjo M, Okumoto K, Hattori E, Adachi T, Takeda T, Ito JI, Sugahara K, Saito K, Togashi H, Kawata S.

DOI: 10.1111/j.1440-1746.2005.03802.x
PMID: 15946157 [PubMed - indexed for MEDLINE]

731. Korean J Intern Med. 2003 Dec;18(4):248-50.

Tsutsugamushi infection-associated acute rhabdomyolysis and acute renal failure.

Young PC(1), Hae CC, Lee KH, Hoon CJ.

Author information:
(1)Department of Internal Medicine, College of Medicine, Chosun University Kwangju, Korea.

Rhabdomyolysis is a rare complication that emerges in a variety of infectious diseases, such as tsutsugamushi infection. In this study, we report a 71-year-old female patient with tsutsugamushi infection who exhibiting rhabdomyolysis and acute renal failure. On admission, an eschar, which is characteristic of tsutsugamushi infection, was found on her right flank area. Moreover, her tsutsugamushi antibody titer was 1:40960. The elevated values of serum creatinine phosphokinase (CPK), aldolase, creatinine and dark brown urine secondary to myoglobinuria are consistent with indications of rhabdomyolysis and acute renal failure due to tsutsugamushi infection. Her health improved without any residual effects after treatment with doxycyclin and hydration with normal saline.

PMCID: PMC4531640
PMID: 14717236 [PubMed - indexed for MEDLINE]

732. Microbiol Immunol. 1991;35(9):687-94.

Serologic survey of spotted fever group rickettsiosis on Hainan Island of China.

Feng HM(1), Chen TS, Lin BH, Lin YZ, Wang PF, Su QH, Xia HB, Kumano K, Uchida T.

Author information:
(1)Department of Microbiology and Immunology, Sun Yat-Sen University of Medical Sciences, Guangzhou, Guangdong Province, People's Republic of China.

A serosurvey for antibodies to Rickettsia japonica was conducted on Hainan Island of China. Serum specimens were collected from 1,030 outpatients at hospitals in different parts of the island regardless of their diagnosis. Only two among 538 serum specimens collected in Baoting and Tongshi counties, located in the southern part of the island, were demonstrated to contain antibodies reactive with R. japonica at a high dilution. The specimens also reacted with R. rickettsii at the same titer as with R. japonica. These two specimens reacted with other pathogenic spotted fever group (SFG) rickettsiae to a lesser extent. On the other hand, the specimens were shown to possess antibodies reactive with R. typhi at a significantly lower dilution or were not reactive at all. The findings suggested the occurrence of an SFG rickettsiosis on Hainan Island. More than half of the serum specimens collected from patients with suspected rickettsial infections in the southern area were found to contain IgM and IgG antibodies to R. typhi, indicating a high incidence of murine typhus.

PMID: 1808466 [PubMed - indexed for MEDLINE]

733. Kansenshogaku Zasshi. 1985 May;59(5):471-7.

[Studies on tsutsugamushi disease in Gifu Prefecture. I. Isolation of Rickettsia tsutsugamushi from wild rodents at a new area and epidemiological studies on patients and latent patients in the prefecture].

[Article in Japanese]

Kasuya S, Iwasa M, Hioki A, Ito A, Ohtomo H, Noda N, Watanabe M, Yamada F.

PMID: 3932534 [PubMed - indexed for MEDLINE]

734. Southeast Asian J Trop Med Public Health. 2001 Sep;32(3):541-6.

Demonstration of the natural foci of tsutsugamushi disease in Nan Peng Lie Islands in China.

Shanshan W(1), Pulin J, Jialiang H, Guifu P, Nianhua Z, Jinhua L, Shaofan Z, Zhibing W.

Author information:
(1)Medical Institute, Guangzhou Command PLA, People's Republic of China.

In recent years, the incidence of tsutsugamushi disease has increased in Nan Peng Lie Islands in China, and the disease has not been recorded in this region. The natural foci of tsutsugamushi disease were investigated in this paper. Isolation of Orientia tsutsugamushi and the study of preventive measures were also performed. The region was the island natural foci of south subtropical zone. The main host and vector were Rattus norvegicus and Leptotrombidium (L.) deliens respectively. The seasonal quantity trends of Rattus norvegicus and Leptotrombidium (L.) deliense were consistent with the incidence of human infection in the region. The strains of O. tsutsugamushi were isolated from Rattus norvegicus and Leptotrombidium (L.) deliense. The identification showed that most strains were Karp. The seroepidemiology showed a high prevalence of antibody against O. tsutsugamushi. After preventive measures were implemented, the incidence was descent. So Nan Peng Lie Islands were the natural foci of tsutsugamushi disease.

PMID: 11944714 [PubMed - indexed for MEDLINE]

735. Kansenshogaku Zasshi. 2001 May;75(5):365-70.

[Epidemiological analysis on many cases of tsutsugamushi disease found in Hiroshima Prefecture, Japan].

[Article in Japanese]

Iwasaki H(1), Yano T, Kaneko S, Egi M, Takada N, Ueda T.

Author information:
(1)Division of Transfusion Medicine, Fukui Medical University.

The clinical findings of tsutsugamushi disease and the fauna of trombiculid mites in Hiroshima Prefecture were studied in this report. We reviewed 63 cases of tsutsugamushi disease occurring between 1990 and 1999, and most of cases were contracted in the area around the midportion of the Oota River (55 cases; 87.3%). Of these, 30 cases (47.1%) lived in Asakita-ku in Hiroshima City. Eschar was detected in 33/19 (84.6%) cases, and 97.6% (40/41), 88.9% (16/18) patients showed eruption and lymphadenopathy respectively. Biochemical examination revealed liver dysfunction in 38.1% (8/21) patients. Of the 11 cases examined on peripheral blood smear, atypical lymphocytes were detected in 10 cases (90.9%). Fifty-five cases (90.2%) occurred during the restricted season between September and December each year. The predominance of Leptotrombidium scutellare was verified by collection of trombiculid mites along the basin of the Oota River. Serum antibody titration on a patient in Asakita-ku showed reaction to Kawasaki type antigen definitive to L. scutellare. Therefore, we speculate that L. scutellare is a candidate for the vector of Orientia tsutsugamushi in Hiroshima Prefecture.

PMID: 11424485 [PubMed - indexed for MEDLINE]

736. J Med Entomol. 1998 Jul;35(4):556-60.

Immunocytochemical methods to study the distribution of Orientia tsutsugamushi in Leptotrombidium (Acari: Trombiculidae) chiggers.

Myint KS(1), Linthicum KJ, Tanskul P, Lerdthusnee K, Vaughn DW, Manomuth C, Mongkolsirichaikul D, Hansukjariya P, Hastriter MW.

Author information:
(1)Department of Virology, U.S. Army Medical Component, Armed Forces Research Institute of Medical Sciences, Bangkok, Thailand.

Immunocytochemical methods were developed and tested for their ability to detect the distribution of Orientia tsutsugamushi in paraffin sections of adult chiggers (Leptotrombidium imphalum Vercammen-Grandjean & Langston). Rickettsial antigen was detected by application of a simple direct or amplified immunocytochemistry procedure and an indirect immunofluorescent procedure. In the direct procedure alkaline phosphatase conjugation to the mouse polyclonal antibody to the Karp strain was followed by the HistoMark Red test system to detect rickettsial antigen. The amplification procedure used a similar method but used an unlabeled primary antibody followed by secondary biotinylated antimouse IgG, streptavidin-alkaline phosphatase, and the HistoMark Red test system. The immunofluorescent procedure included a biotinylated secondary antibody followed by addition of a streptavidin-FITC conjugate. Specific tissue tropisms in infected chiggers were observed in the salivary glands, nervous tissue, and ovaries of adult female mites in all procedures; however, nonspecific fluorescence of the chigger limited definitive identification of tissue tropisms with the indirect immunofluorescent procedure.

PMID: 9701944 [PubMed - indexed for MEDLINE]

737. J Med Entomol. 1971 Oct 30;8(4):401-6.

Distribution of Leptotrombidium (Leptotrombidium) arenicola (Acarina: Trombiculidae) on the ground in West Malaysia.

Upham RW Jr, Hubert AA, Phang OW, Mat YB, Rapmund G.

PMID: 5159254 [PubMed - indexed for MEDLINE]

738. Southeast Asian J Trop Med Public Health. 1998 Sep;29(3):560-2.

Serological evidence of natural infection of wild rodents (Rattus spp and Tupaia glis) with rickettsiae in Malaysia.

Tay ST(1), Kaewanee S, Ho TM, Rohani MY, Devi S.

Author information:
(1)Institute for Medical Research, Jalan Pahang, Kuala Lumpur, Malaysia.

PMID: 10437957 [PubMed - indexed for MEDLINE]

739. Zhonghua Liu Xing Bing Xue Za Zhi. 1990 Apr;11(2):65-7.

[The natural foci of the tsutsugamushi disease in the suburbs of Hang Zhou City].

[Article in Chinese]

Shi S(1).

Author information:
(1)Hangzhou Sanitation and Anti-Epidemic.

In 1986, investigating tsutsugamushi disease in the suburbs of Hangzhou, We isolated Rickettsia tsutsugamushi from leptotrombidium gaohuense and its reservoir hosts, Rattus confucianus and Rattus fulvescens, which were collected in Yang Shan Wu village, Linan county. It proved there was a natural focus of tsutsugamushi disease in the suburbs of Hang zhou. Which is located at 30 degrees 35'. North latitude, and main forest of the East Tianmu Mountain, covered by wide and needle leaves, with more wide leaves than needle one. The vector is leptotrombidium gaohuense. The host are Rattus confucianus and fulvescens. Such focus belongs to inland-mountain type.

PMID: 2115400 [PubMed - indexed for MEDLINE]

740. Med Parazitol (Mosk). 1969 Jan-Feb;38(1):72-8.

[Features of ecology and distribution of Trombiculidae mites of Viet Nam].

[Article in Russian]

Grokhovskaia IM, Nguen Suan Khoe.

PMID: 5392652 [PubMed - indexed for MEDLINE]

741. Kansenshogaku Zasshi. 1991 Feb;65(2):151-6.

[Studies on tsutsugamushi disease in Gifu prefecture. 4. Survey results in Ena and Takayama City, and the summary of the studies in the prefecture].

[Article in Japanese]

Kasuya S(1), Koga K, Hioki A, Nagano I, Yamashita T, Ohtomo H, Iwasa M, Noda N.

Author information:
(1)Department of Parasitology, Gifu University School of Medicine.

Investigations of trombiculid mites and Rickettsia tsutsugamushi in wild rodents were made in Ena (Nov. 1988) and Takayama (May 1989) City. In the former area where no patient has been reported so far, Leptotrombidium pallidum (63.9%) was most predominant and no L. scutellare was found. A Karp-related rickettsia (11.1%) was isolated from wild rodents and 30% of them had antibody to R. tsutsugamushi (anti-Karp was prominent). The latter area where patients were reported in spring as well as in autumn, L. pallidum (22.4%) was one of the dominant species in spring. Karp-related strains were isolated from 37.5% of wild rodents. And the antibody possession rate was 50.0%. These data reconfirmed our hypothesis that the majority of patients in autumn were infected by L. scutellare and a part by L. pallidum. In spring in Takayama area, the vector was L. pallidum which possessed Karp-related strain(s).

PMID: 1906079 [PubMed - indexed for MEDLINE]

742. Korean J Parasitol. 1995 Mar;33(1):27-36.

Comparative epidemiological studies on vector/reservoir animals of tsutsugamushi disease between high and low endemic areas in Korea.

Ree HI(1), Cho MK, Lee IY, Jeon SH.

Author information:
(1)Institute of Tropical Medicine, Yonsei University, Seoul, Korea.

Comparative epidemiological studies on vector reservoir animals of tsutsugamushi disease were carried out in between south coast (the highest endemic) areas and east coast (low endemic) areas in October 1993. Fauna of field rodents and their population densities were not different between two areas. Antibody positive rate of Apodemus agrarius sera was higher in east coast (43.2% in south coast and

63.6% in east coast). High correlation (r = 0.87) was shown between antibody positive rate of A. agrarius sera and population density of the vector mites (Leptotrombidium scutellare and L. pallidum). L. scutellare was predominant in south coast, showing 110.6 chigger index (74.9% of the total chiggers), whereas L. pallidum was predominant in east coast, showing 126.3 chigger index (60.4% of the total). As higher population density of L. scutellare was found in south coast where the prevalence rate of tsutsugamushi disease is the highest, it is believed that L. scutellare is more important vector species than L. pallidum, which may result from more frequent vector-human contact.

PMID: 7735783 [PubMed - indexed for MEDLINE]

743. Zhonghua Liu Xing Bing Xue Za Zhi. 1994 Feb;15(1):54-7.

[Advances in the epidemiologic study of Tsutsugamushi disease of the autumn-winter type in China].

[Article in Chinese]

Wu GH.

PMID: 8082142 [PubMed - indexed for MEDLINE]

744. Zhonghua Yu Fang Yi Xue Za Zhi. 1996 May;30(3):133-5.

[Role of Leptotrombidium (L.) scutellare in transmission of human diseases].

[Article in Chinese]

Wu G(1), Zhang Y, Guo H.

Author information:
(1)Military Medical Institute Nanjing Command, PLA, Nanjing.

A series of cases of hemorrhagic fever with renal syndrome (HFRS) in Shaanxi province and autumn-type tsutsugamushi disease in Jiangsu province were studied during 1988 to 1990 and 1986 to 1993 to explore the role of Leptotrombidium (L.) scutellare in transmission of these diseases. Results showed that L. (L.) scutellare is a dominant strain of mites inhabiting in rats and its seasonal fluctuation conformed to the seasonal distribution of these diseases. L. (L.) scutellare could naturally infect with HFRS virus and Rickettsia tsutsugamushi and transmit them by stinging and transovarial route. It indicated that L. (L.) scutellare could be a vector in transmission of HFRS and autumntype tsutsugamushi disease.

PMID: 9208519 [PubMed - indexed for MEDLINE]

745. Kansenshogaku Zasshi. 1980 Jun;54(6):291-8.

[Tsutsugamushi disease in the foothills of Mt. Fuji (author's transl)].

[Article in Japanese]

Murata M, Nogami S, Shirasaka A, Tanaka H, Kawamura A Jr, Mochizuki H, Oyama H.

PMID: 6778934 [PubMed - indexed for MEDLINE]

746. Am J Trop Med Hyg. 1978 Jan;27(1 Pt 1):121-3.

Serological evidence for a high incidence of transmission of Rickettsia tsutsugamushi in two Orang Asli settlements in Peninsular Malaysia.

Brown GW, Robinson DM, Huxsoll DL.

Two communities of Orang Asli (aborigines) in Peninsular Malaysia were observed for evidence of Rickettsia tsutsugamushi infection over periods of 1-8 mo. Sequential sera were examined for antibody by the indirect immunofluorescence test. The incidence of infection in the two self-selected populations in the two communities was calculated to be 3.9% per month and 3.2% per month.

PMID: 415625 [PubMed - indexed for MEDLINE]

747. Med J Aust. 1947 Oct 11;2(15):441-50.

Observations on the epidemiology of tsutsugamushi disease in North Queensland.

SOUTHCOTT RV.

PMID: 20271246 [PubMed - indexed for MEDLINE]

748. Indian Pediatr. 2011 Nov 11;48(11):867-72. Epub 2011 Mar 15.

Rickettsial diseases in central India: proposed clinical scoring system for early detection of spotted fever.

Rathi NB(1), Rathi AN, Goodman MH, Aghai ZH.

Author information:
(1)Rathi Childrens Hospital and Maternity Home, Akola, MS, India.
drnbrathi@hotmail.com

Comment in
    Indian Pediatr. 2012 Mar;49(3):251; discussion 251-2.
    Indian Pediatr. 2012 Jul;49(7):589-90.

OBJECTIVES: To report a series of cases of rickettsial infections from central

India and to develop a clinical scoring system for its early detection.
DESIGN: Retrospective review of children hospitalized during one year period with fever without a source, and presence of one or more of the clinical features suggestive of rickettsial infection. Diagnosis of rickettsial disease was made by classical clinical features and detection of IgM antibody by ELISA. A clinical scoring system was developed to diagnose spotted fever group by using classical clinical and laboratory findings.
RESULTS: 161 patients were admitted and met the inclusion criteria, 75 (45.6%) were diagnosed with rickettsial diseases. 52 (69.3%) had spotted fever group and 23 (30.7%) scrub typhus. The mortality rate with rickettsial diseases was 9%. By using proposed clinical scoring system, a score of 14 has sensitivity and specificity of 96.15% and 98.84%, respectively in making a diagnosis of spotted fever group.
CONCLUSION: Rickettsial diseases are common in the central part of India and should be included in the differential diagnosis of patients with fever of undetermined source. The proposed scoring system can be used for early detection, treatment and prevention of mortality and morbidity from spotted fever group.

PMID: 21555807 [PubMed - indexed for MEDLINE]

749. U S Nav Med Bull. 1946 Nov;46(11):1669-73.

Tsutsugamushi disease on Samar, Philippine Islands.

ERRINGTON AF, KING AN, et al.

PMID: 21002704 [PubMed - indexed for MEDLINE]

750. Med J Aust. 1946 Jul 6;2:20.

Tsutsugamushi fever in natives.

NOAD KB.

PMID: 20991510 [PubMed - indexed for MEDLINE]

751. Sov Med. 1975 Aug;(8):67-70.

[Rickettsial diseases (results and perspectives of the epidemiological studies)].

[Article in Russian]

Skvortsov VV, Tarasevich IV, Lobanov AV.

PMID: 1105811 [PubMed - indexed for MEDLINE]

752. Kisaengchunghak Chapchi. 1991 Jun;29(2):181-8.

Epidemiological studies on host animals of tsutsugamushi disease in Korea.

Ree HI(1), Lee HS, Lee IY, Yoshida Y.

Author information:
(1)Department of Parasitology, Yonsei University College of Medicine, Seoul, Korea.

Epidemiological studies on host rodents of tsutsugamushi disease were carried out during the period of July-September 1990 at nine localities of central Korea. Among total 111 wild rodents trapped by the modified Sherman live traps, 103 were Apodemus agrarius (92.8%), seven were Crocidura lasiura (6.3%) and one was Microtus fortis (0.9%), showing 24.0% of trapping rate in winter, 11.7% in spring, 11.2% in summer and 12.0% in autumn. Out of 103 A. agrarius 84 were parasitized by chiggers, showing 81.6% of the infestation rate and 43.0 of the chigger index. The antibody positive rate of A. agrarius sera to Rickettsia tsutsugamushi was significantly variable by locality, being in the range of 0-78.6%. The seasonal change of the antibody positive rate at Dorai 5-ri, Goyang-gun was 75.8% in average during November-March, decreased to 30.3% in April and further decreased to 13.3% in average during May-August. Among 33 antibody positives, 31 were Karp strain and two were Gilliam. Seven Crocidura lasiura sera showed all negative. R. tsutsugamushi organisms were isolated from three A. agrarius out of 94 mice tested, showing 3.2% of the infection rate.

PMID: 1954201 [PubMed - indexed for MEDLINE]

753. J Antimicrob Chemother. 2011 Aug;66(8):1821-30. doi: 10.1093/jac/dkr218. Epub 2011 Jun 4.

Analysis of risk factors for malignant Mediterranean spotted fever indicates that fluoroquinolone treatment has a deleterious effect.

Botelho-Nevers E(1), Rovery C, Richet H, Raoult D.

Author information:
(1)URMITE UMR CNRS 6236, IRD 198, Université de la Méditerranée, Faculté de Médecine, 27 Bd Jean Moulin, 13385 Marseille cedex 05, France.

OBJECTIVES: To identify risk factors for malignant Mediterranean spotted fever (MSF) caused by Rickettsia conorii conorii.
PATIENTS AND METHODS: Epidemiological, clinical and biological characteristics as well as risk factors (including treatment regimens) for severe MSF cases were analysed retrospectively. A patient with two or more organ dysfunctions or patient death was defined as a severe case.
RESULTS: During the study period (January 1999 to December 2009), 161 MSF cases were referred to our centre for rickettsioses. Twenty-six cases (16.1%) were considered severe, which is 3-fold higher than in our previous studies. The clinical and laboratory findings were comparable to those reported elsewhere except that the type of antibiotic treatment was associated with disease severity. Doxycycline administration prior to deterioration of disease (in 31

patients) protected patients from development of severe MSF [relative risk (RR) 0.248, 95% confidence interval (CI) 0.08-0.76] and induced earlier defervescence compared with the other treatment regimens (3.02 ± 2.2 days versus 7.1 ± 6.57 days, P = 0.021). In contrast, fluoroquinolone treatment (in 21 patients) was significantly and independently associated with MSF severity (RR 2.53, 95% CI 1.40-4.55) and was associated with a significantly longer hospital stay.
CONCLUSIONS: In this retrospective study fluoroquinolone treatment was associated with increased MSF disease severity. Fluoroquinolones have been previously associated with treatment failure in typhus and scrub typhus cases. Thus, we do not recommend the use of fluoroquinolones to treat rickettsial diseases.

DOI: 10.1093/jac/dkr218
PMID: 21642652 [PubMed - indexed for MEDLINE]

754. Kansenshogaku Zasshi. 1994 Sep;68(9):1057-62.

[Five cases of tsutsugamushi disease].

[Article in Japanese]

Fujii T(1), Sakata S, Mori N, Ishino T, Kadota J, Kohno S, Hara K.

Author information:
(1)Department of Internal Medicine, Hokusyo Central Hospital.

We experienced 5 cases of tsutusgamushi disease from October to November 1993 at Hokusyo Central Hospital. All patients showed high fever, skin rash and eschar and four patients showed lymph node swelling. All cases were diagnosed serologically by indirect-immunofluorescence technique and treated with minocycline. In a survey of anti-Karp, Kato, Gilliam, Kawasaki, Kuroki antibodies, all patients showed the highest antibody titers against the Kawasaki strain and they were considered Kawasaki type. In Nagasaki Prefecture, the number of patients with tsutsugamushi disease has been increasing since 1982. We carried out immunologic and epidemiologic studies about this disease in Nagasaki Prefecture.

PMID: 7963795 [PubMed - indexed for MEDLINE]

755. Rickettsiae.

Walker DH.
In: Baron S, editor. Medical Microbiology. 4th edition. Galveston (TX): University of Texas Medical Branch at Galveston; 1996. Chapter 38.

Rickettsiae are small, Gram-negative bacilli that have evolved in such close association with arthropod hosts that they are adapted to survive within the host cells. They represent a rather diverse collection of bacteria, and therefore listing characteristics that apply to the entire group is difficult. The common threads that hold the rickettsiae into a group are their epidemiology, their obligate intracellular lifestyle, and the laboratory technology required to work with them. In the laboratory, rickettsiae cannot be cultivated on agar plates or

in broth, but only in viable eukaryotic host cells (e.g., in cell culture, embryonated eggs, or susceptible animals). The exception, which shows the artificial nature of using obligate intracellular parasitism as a defining phenotypic characteristic, is Bartonella (Rochalimaea) quintana, which is cultivable axenically, but was traditionally considered as a rickettsia. The diversity of rickettsiae is demonstrated in the variety of specific intracellular locations where they live and the remarkable differences in their major outer membrane proteins and genetic relatedness (Table 38-1). An example of extreme adaptation is that the metabolic activity of Coxiella burnetii is greatly increased in the acidic environment of the phagolysosome, which is a harsh location for survival for most other organisms. Obligate intracellular parasitism among bacteria is not unique to rickettsiae. Chlamydiae also have evolved to occupy an intracellular niche, and numerous bacteria (e.g., Mycobacteria, Legionella, Salmonella, Shigella, Francisella, and Brucella) are facultative intracellular parasites. In contrast with chlamydiae, all rickettsiae can synthesize ATP. Coxiella burnetii is the only rickettsia that appears to have a developmental cycle. Some organisms in the family Rickettsiaceae are closely related genetically (e.g., Rickettsia rickettsii, R akari, R prowazekii, and R typhi); others are related less closely to Rickettsia species (e.g., Ehrlichia and Bartonella); and others not related to Rickettsia species (e.g., C burnetii). The phenotypic traits of the medically important organism Orientia (Rickettsia) tsutsugamushi suggest that the species may be an example of convergent evolution in a similar ecologic niche. Rickettsioses are zoonoses that, except for Q fever, are usually transmitted to humans by arthropods (tick, mite, flea, louse, or chigger) (Table 38-2). Therefore, their geographic distribution is determined by that of the infected arthropod, which for most rickettsial species is the reservoir host. Rickettsiae are important causes of human diseases in the United States (Rocky Mountain spotted fever, Q fever, murine typhus, sylvatic typhus, human monocytic ehrlichiosis, human granulocytic ehrlichiosis, and rickettsialpox) and around the world (Q fever, murine typhus, scrub typhus, epidemic typhus, boutonneuse fever, and other spotted fevers) (Table 38-2).

PMID: 21413251 [PubMed]

756. Kansenshogaku Zasshi. 1986 Sep;60(9):1022-6.

[Studies on tsutsugamushi disease in Gifu Prefecture. 3. Seasonal fluctuation in positive rates of Rickettsia tsutsugamushi in wild rodents and number of patients].

[Article in Japanese]

Kasuya S, Hioki A, Ito A, Ohtomo H, Noda N, Watanabe M, Yamada F, Iwasa M.

PMID: 3100698 [PubMed - indexed for MEDLINE]

757. Mil Med. 1979 Mar;144(3):175-6.

Seroepidemiological evidence of infectious diseases in United States Marine Corps personnel, Okinawa, Japan, 1975--1976.

Olson JG, Irving GS, Bourgeois AL, Hodge FA, Van Peenen PF.

PMID: 107484 [PubMed - indexed for MEDLINE]

758. U S Nav Med Bull. 1946 Mar;46:459-72.

Tsutsugamushi disease; epidemiology and methods of survey and control.

JOHNSON DH, WHARTON GW.

PMID: 21012959 [PubMed - indexed for MEDLINE]

759. Kansenshogaku Zasshi. 1987 Jun;61(6):645-51.

[Fourteen cases of Tsutsugamushi disease in Nagasaki prefecture].

[Article in Japanese]

Watanabe K, Shigeno Y, Kohno S.

PMID: 3119736 [PubMed - indexed for MEDLINE]

760. Kansenshogaku Zasshi. 1987 Mar;61(3):297-302.

[Clinical study of tsutsugamushi disease in Miyazaki District].

[Article in Japanese]

Shishime E, Tachibana N, Okayama A, Ishizaki J, Yokota T, Tsuda K.

PMID: 3112283 [PubMed - indexed for MEDLINE]

761. Kansenshogaku Zasshi. 1980 Dec;54(12):755-65.

[Observations on the new type tsutsugamushi disease in Akita Prefecture (author's transl)].

[Article in Japanese]

Suzuki T, Suto T.

PMID: 6788861 [PubMed - indexed for MEDLINE]

762. Zh Mikrobiol Epidemiol Immunobiol. 1976 Feb;(2):69-73.

[Tsutsugamushi fever in the Kuril Islands].

[Article in Russian]

Somoz GP, Shubin FN, Gopachenko IM, Kononova DG.

The authors present the results of a 6-year study of tsutsugamushi fever at the Kuril islands. The area of the disease proved to include Southern Kuril islands - Shikotan and Kunashir. The principal natural carriers of tsutsugamushi among the mouse-like rodents and trombiculidae larvae were revealed; highly virulent strains were found to prevail among the tsutsugamushi rickettsia strains. Tsutsugamushi fever in the population is characterized by a benign course, pyrexia, primary affects and lymphadenitis.

PMID: 1266470 [PubMed - indexed for MEDLINE]

763. Acta Leiden. 1968;36:1-8.

Scrubtyphus, an epidemiological study.

Bekker BV, Dinger JE, Wolff HL.

PMID: 5761081 [PubMed - indexed for MEDLINE]

764. Klin Med (Mosk). 1969 May;47(5):106-10.

[The clinical picture of Tsutsugamushi fever in the Maritime Territory].

[Article in Russian]

Shapiro MI, Dandurov IuV, Sobolev IN, Zalmover IIu, Lazarev AN, Zeeman EV, Shylkov BI.

PMID: 5367566 [PubMed - indexed for MEDLINE]

765. Zh Mikrobiol Epidemiol Immunobiol. 1969 Feb;46(2):139.

[A study of the immunologic structure of the population of various regions of Tadzhik SSR with regard to tsutsugamushi fever].

[Article in Russian]

Nazarenko SI, Tarasevich IV, Plotnikova LF, Fetisova NF.

PMID: 4239989 [PubMed - indexed for MEDLINE]

766. Rev Inst Med Trop Sao Paulo. 1968 Jul-Aug;10(4):256-61.

[Macular fever in São Paulo. Therapeutic results in some cases with tetracycline laurylsulfate].

[Article in Portuguese]

da Tiriba AC, de Godoy CV, de Brito T, Jordão FM, de Penna DO, de Souza AR.

PMID: 5679660 [PubMed - indexed for MEDLINE]

767. Trop Doct. 2010 Oct;40(4):230-4. doi: 10.1258/td.2010.100132. Epub 2010 Sep 24.

Acute undifferentiated febrile illness in adult hospitalized patients: the disease spectrum and diagnostic predictors - an experience from a tertiary care hospital in South India.

Chrispal A(1), Boorugu H, Gopinath KG, Chandy S, Prakash JA, Thomas EM, Abraham AM, Abraham OC, Thomas K.

Author information:
(1)Department of Medicine Unit 2, Christian Medical College, Vellore, Tamil Nadu, India. anugrahchrispal@gmail.com

Local prevalences of individual diseases influence the prioritization of the differential diagnoses of a clinical syndrome of acute undifferentiated febrile illness (AFI). This study was conducted in order to delineate the aetiology of AFI that present to a tertiary hospital in southern India and to describe disease-specific clinical profiles. An 1-year prospective, observational study was conducted in adults (age >16 years) who presented with an undifferentiated febrile illness of duration 5-21 days, requiring hospitalization. Blood cultures, malarial parasites and febrile serology (acute and convalescent), in addition to clinical evaluations and basic investigations were performed. Comparisons were made between each disease and the other AFIs. A total of 398 AFI patients were diagnosed with: scrub typhus (47.5%); malaria (17.1%); enteric fever (8.0%); dengue (7.0%); leptospirosis (3.0%); spotted fever rickettsiosis (1.8%); Hantavirus (0.3%); alternate diagnosis (7.3%); and unclear diagnoses (8.0%). Leucocytosis, acute respiratory distress syndrome, aseptic meningitis, mild serum transaminase elevation and hypoalbuminaemia were independently associated with scrub typhus. Normal leukocyte counts, moderate to severe thrombocytopenia, renal failure, splenomegaly and hyperbilirubinaemia with mildly elevated serum transaminases were associated with malaria. Rash, overt bleeding manifestations, normal to low leukocyte counts, moderate to severe thrombocytopenia and significantly elevated hepatic transaminases were associated with dengue. Enteric fever was associated with loose stools, normal to low leukocyte counts and normal platelet counts. It is imperative to maintain a sound epidemiological database of AFIs so that evidence-based diagnostic criteria and treatment guidelines can be developed.

DOI: 10.1258/td.2010.100132
PMID: 20870680 [PubMed - indexed for MEDLINE]

768. Jpn J Med Sci Biol. 1981 Feb;34(1):37-9.

Rickettsia tsutsugamushi antibody in mother/cord pairs of sera.

Shirai A, Brown GW, Gan E, Huxsoll DL, Groves MG.

PMID: 6790744 [PubMed - indexed for MEDLINE]

769. Zh Mikrobiol Epidemiol Immunobiol. 1990 Mar;(3):94-8.

[Epidemiologic surveillance of obligate transmissible rickettsiosis with natural foci].

[Article in Russian]

Iastrebov VK.

PMID: 2195814 [PubMed - indexed for MEDLINE]

770. J Bacteriol. 1946 May;51:629.

The cultivation of Rickettsia orientalis in fertile hens' eggs.

CLANCY CF, COX HR.

PMID: 21064746 [PubMed - indexed for MEDLINE]

771. Am J Trop Med Hyg. 1973 Nov;22(6):796-801.

Epidemiology of the acute fevers of unknown origin in South Vietnam: effect of laboratory support upon clinical diagnosis.

Berman SJ, Irving GS, Kundin WD, Gunning JJ, Watten RH.

PMID: 4355456 [PubMed - indexed for MEDLINE]

772. Kansenshogaku Zasshi. 1991 Jun;65(6):749-53.

[A case of tsutsugamushi disease in the urban area of Komatsu City].

[Article in Japanese]

Kajinami K(1), Fujita H, Hirata M, Ueda K, Kameda S, Noto M, Kaseda H.

Author information:
(1)Department of Internal Medicine, Komatsu Municipal Hospital.

Most of patients with tsutsugamushi disease are diagnosed by their clinical histories suggesting the opportunities of Rickettsia infection in a rural region. We reported a 76-year-old female patient, who was considered to be infected in her house in the urban area of Komatsu City. She has shown typical clinical manifestations of tsutsugamushi disease, and was remitted successfully by oral administration of minocycline. Although specific antibodies to Rickettsia tsutsugamushi could not be detected in her serum by the complement fixation (CF) method during her clinical course, their significant elevation was confirmed by the indirect immunofluorescence (IF) method.

PMID: 1919106 [PubMed - indexed for MEDLINE]

773. Trans R Soc Trop Med Hyg. 1982;76(1):1-3.

Sero-epidemiological survey of Rickettsia tsutsugamushi infection in a rural Thai village.

Johnson DE, Crum JW, Hanchalay S, Saengruchi C.

A sero-epidemiological survey of a rural Thai village demonstrated a 77% prevalence of antibody against Rickettsia tsutsugamushi in adults. Acquisition of antibody occurred very early in life, especially in females, but the prevalence of antibody in the adult population showed no statistically significant sexual distinction. Antibody against all three prototype strains was present in Thailand but antibody titres did not vary by strain type or the age of the individual.

PMID: 6805102 [PubMed - indexed for MEDLINE]

774. Nephrol Dial Transplant. 2011 Feb;26(2):524-31. doi: 10.1093/ndt/gfq477. Epub 2010 Aug 11.

Acute kidney injury in tropical acute febrile illness in a tertiary care centre--RIFLE criteria validation.

Basu G(1), Chrispal A, Boorugu H, Gopinath KG, Chandy S, Prakash JA, Thomas K, Abraham AM, John GT.

Author information:
(1)Department of Nephrology, Christian Medical College, Vellore, India.

BACKGROUND: Acute febrile illnesses are a common cause of tropical acute kidney injury (AKI). The incidence and severity of AKI in tropical febrile illnesses and validity of RIFLE classification are unclear.
METHODS: Consecutive adult inpatients of a tertiary hospital in southern India with tropical acute febrile illness between January 2007 and January 2008 were prospectively studied for the incidence and severity of AKI based on RIFLE classification and its association with mortality and dialysis requirement.

RESULTS: The 367 patients (mean age 39.7±16.9 years; 60% males) with tropical acute febrile illness due to scrub typhus (51.2%), falciparum malaria (10.4%), enteric fever (8.7%), dengue (7.6%), mixed malaria (6.5%), leptospirosis (3.3%), undifferentiated acute febrile illness (8.4%) and others (3.8%) (spotted fever, vivax malaria and Hantaan virus infection) had an overall mortality rate of 12.3%. The incidence of AKI was 41.1%; of which, 17.4%, 9.3% and 14.4% were in the Risk, Injury and Failure classes, respectively. Of the patients, 7.9% required dialysis. Among the Risk, Injury and Failure groups, there was an incremental risk of mortality (OR 6.9, 20.2 and 25.6; P<0.001) and dialysis requirement (OR 3.4, 28.8 and 178.8; P<0.001).
CONCLUSIONS: The incidence of AKI in the common tropical acute febrile illnesses in our study such as scrub typhus, falciparum malaria, enteric fever, dengue and leptospirosis is 41.1%. RIFLE classification is valid and applicable in AKI related to tropical acute febrile illnesses, with an incremental risk of mortality and dialysis requirement.

DOI: 10.1093/ndt/gfq477
PMID: 20702532 [PubMed - indexed for MEDLINE]

775. J Clin Microbiol. 2004 May;42(5):2310-3.

First serologic evidence of human spotted fever group rickettsiosis in Korea.

Jang WJ(1), Kim JH, Choi YJ, Jung KD, Kim YG, Lee SH, Choi MS, Kim IS, Walker DH, Park KH.

Author information:
(1)Department of Microbiology, Kon-Kuk University College of Medicine, Choongju-si, Choongbuk 380-701, Republic of Korea.

Comment in
  J Clin Microbiol. 2005 Aug;43(8):4306; author reply 4306-7.

To investigate the prevalence of spotted fever group rickettsioses in Korea, a serosurvey of Japanese spotted fever rickettsiosis in patients with acute febrile illness was conducted with an indirect immunofluorescence assay. Overall, 19.88% of the patients were found to have polyvalent antibody against Rickettsia japonica. This study is the first documentation of spotted fever group rickettsiosis in Korea.

PMCID: PMC404613
PMID: 15131221 [PubMed - indexed for MEDLINE]

776. Am J Trop Med Hyg. 1997 Nov;57(5):569-70.

Short report: surveillance of rickettsial infections in Indonesian military personnel during peace keeping operations in Cambodia.

Corwin AL(1), Soeprapto W, Widodo PS, Rahardjo E, Kelly DJ, Dasch GA, Olson JG, Sie A, Larasati RP, Richards AL.

Author information:
(1)U.S. Naval Medical Research Unit No. 2, Jakarta, Indonesia.

Indonesian peacekeepers in Cambodia provided a unique study population to estimate the threat of rickettsial exposure to Rickettsia typhi (murine typhus), Orientia tsutsugamushi, (scrub typhus), and R. conorii (spotted fever) for the region. Prescreening prevalence measure showed a large proportion (36%) of soldiers with antibodies to R. typhi. Predeployment prevalence for antibodies to O. tsutsugamushi was 8%, with no evidence of background R. conorii infections. Actual seroconversions of R. typhi (3) and O. tsutsugamushi (1), attributed to exposure(s) in Cambodia, translated into annualized incidence rates of 24 and 8 per 1,000 per year, respectively. Surveillance of rickettsial infections and/or disease is particularly warranted in Cambodia with recent recognition of drug-resistant scrub typhus in neighboring Thailand.

PMID: 9392597 [PubMed - indexed for MEDLINE]

777. Bull World Health Organ. 1982;60(5):693-701.

[Rickettsioses: a problem of persistent morbidity. WHO study group on rickettsial diseases].

[Article in French]

[No authors listed]

PMCID: PMC2536041
PMID: 6983924 [PubMed - indexed for MEDLINE]

778. J Med Entomol. 1992 Mar;29(2):284-92.

Description of six new species of the genus Leptotrombidium from Mindanao Island and notes on the medically important genera of chiggers (Acari: Trombiculidae) of the Philippine Islands.

Brown WA(1).

Author information:
(1)Department of Entomology, University of Hawaii, Manoa, Honolulu 96822.

In the Philippine Islands, the genus Leptotrombidium currently consists of members of the subgenera Leptotrombidium and Trombiculindus. The following species are described as new: L. (L.) macacaphilus, L. (L.) longimedian, L. (L.) mindanensis, L. (L.) urogale, L. (L.) oculascutum, L. (T.) roseannleilaniae. The distribution of, and comments on, the known vectors of Rickettsia tsutsugamushi, L. (L.) deliense and L. (L.) fletcheri and the previously reported L. (L.) sandfordi, are given. Information on species of the potential scrub itch genera Blankaartia, Eutrombicula, and Schoengastia and a key to the genera and species of medically important trombiculids is provided.

PMID: 1495044 [PubMed - indexed for MEDLINE]

779. Nihon Shokakibyo Gakkai Zasshi. 2004 Jan;101(1):52-6.

[A case of Tsutsugamushi disease complicated with cholecystitis].

[Article in Japanese]

Inaba H(1), Sugawara Y, Oouchi K, Tomichi N, Sato S, Takikawa Y, Suzuki K.

Author information:
(1)First Department of Internal Medicine, Iwate Medical University.

PMID: 14763158 [PubMed - indexed for MEDLINE]

780. Dermatol Online J. 1997 Dec;3(2):3.

Eschar, fever and rash in a 43 year old man. Tsutsugamushi disease.

Taniguchi Y(1).

Author information:
(1)Department of Dermatology, Mie University Faculty of Medicine, Japan.

PMID: 9452369 [PubMed - indexed for MEDLINE]

781. Dtsch Med Wochenschr. 2004 Oct 15;129(42):2233-5.

[Tsutusgamushi fever after travel to Southeast Asia].

[Article in German]

Seilmaier M(1), Guggemos W, Böhme CC, Löscher T.

Author information:
(1)l. Medizinische Abteilung, Städtisches Krankenhaus München Schwabing, München.

HISTORY AND CLINICAL FINDINGS: A 49-year-old travel guide fell ill during his return from a 6-week-stay in Vietnam, Myanmar and Thailand. He developed high fever and severe headache. On admission, the body temperature was at 39.5 degrees C with relative bradycardia. A black, crusted skin lesion of approximately 5 mm diameter was noted on the chest wall and was interpreted as an insect bite.
INVESTIGATIONS: CRP and liver enzymes were elevated. Total leucocyte count was normal but the differential count showed a left shift and aneosinophilia. Imaging procedures revealed splenomegaly and small pleural effusions on both sides.
TREATMENT AND COURSE: The patient was treated with a parenteral quinolone based on the initial suspicion of typhoid fever. Failure of this treatment and negative blood cultures raised concerns about the possibility of Tsutsugamushi fever,

based on travel history and a re-evaluation of the skin lesion as an eschar.
Tsutsugamushi fever was suspected on epidemiological and clinical grounds and was confirmed by the detection of specific IgM to Orienta tsutsugamushi and by seroconversion of IgG antibodies during follow-up. Even before immunodiagnostic confirmation was available, a course of doxycycline was started. This led to rapid improvement of the patient's condition.
CONCLUSION: In febrile travellers returning from Southeast Asia, Tsutsugamushi fever has to be considered in the differential diagnosis. The causative agent, Orienta tsutsugamushi is transmitted by larvae of trombiculid mites (chiggers). Leading symptoms are fever continua, cephalgia, and a primary lesion (eschar) at the site of cutaneous inoculation. The Eschar is easily overlooked and has to be searched carefully. Diagnosis is confirmed by the detection of specific antibodies. However, serology may be negative in the beginning. Therefore, treatment with doxycycline should be initiated on clinical grounds.

DOI: 10.1055/s-2004-831868
PMID: 15483757 [PubMed - indexed for MEDLINE]

782. Rinsho Shinkeigaku. 1991 Oct;31(10):1103-6.

[Meningitis associated with tsutsugamushi disease--detection of intrathecal interferon-gamma synthesis and lymphocyte subsets in blood and CSF].

[Article in Japanese]

Ikeda M(1), Sato T, Takahashi H, Yoshida S, Tsukagoshi H.

Author information:
(1)Department of Neurology, Asahi General Hospital.

We report a case of meningitis associated with tsutsugamushi disease. The lymphocyte subpopulation study of peripheral blood revealed a reversed CD4/CD8 ratio (0.14), an increased number of activated T cells and an elevated cytotoxic T cell activity. In cerebrospinal fluid (CSF), the CD4/CD8 ratio was much higher (0.84) and activated T cells were less prevalent than in blood. These pictures of lymphocyte subsets were characteristic and different from that of viral meningitis. We also verified an elevation of interferon-gamma levels in CSF as well as in serum and intrathecal interferon-gamma synthesis while the specific antibody production in CSF compartment was not demonstrated. T cell-mediated immunity many play an important role not only systemically but also locally in the central nervous system in tsutsugamushi disease.

PMID: 1802466 [PubMed - indexed for MEDLINE]

783. J Indian Med Assoc. 1978 Aug 16;71(4):104-7.

Rickettsiosis in India : a review.

Padbidri VS, Gupta NP.

PMID: 366022 [PubMed - indexed for MEDLINE]

784. Sov Med. 1975 Aug;(8):67-70.

[Rickettsioses (results and prospects for epidemiologic studies)].

[Article in Russian]

Skvortsov VV, Tarasevich IV, Lobanov AV.

PMID: 788186 [PubMed - indexed for MEDLINE]

785. Southeast Asian J Trop Med Public Health. 2002 Sep;33(3):551-6.

Sequence analysis of Orientia tsutsugamushi DNA from mites collected in the Xisa archipelago, China.

Wang SS(1), Zhan DC, Peng GF, Pan H, Xiao H, Zeng NH, Huang JL, Wang ZB.

Author information:
(1)Department of Epidemiology, Medical Institute of Guangzhou Command PLA, Yanling, Guangzhou, People's Republic of China.

The genotype of Orientia tsutsugamushi DNA from mites in the Xisa archipelago of China were identified. A natural focus of tsutsugamushi disease in the archipelago was found. The DNA sequence that codes for the 56 kDa protein of O. tsutsugamushi was amplified by nested polymerase chain reaction (N-PCR). The purified positive products were cloned into a pGEM-T vector and sequenced. The DNA sequence was compared with various sequences on the internet for sequence homology. A 507 bp DNA fragment encoding the 56 kDa protein was amplified from the samples. The sequence homology was 85% (Karp strain), 68% (Gilliam strain), 65% (Kato strain), and 67% (Yonchon strain). Orientia tsutsugamushi is carried by the mites of the Xisa archipelago; the main genotype is the Karp strain.

PMID: 12693590 [PubMed - indexed for MEDLINE]

786. J Med Entomol. 2001 Mar;38(2):308-11.

Detection of Orientia tsutsugamushi (Rickettsiales: rickettsiaceae) in unengorged chiggers (Acari: Trombiculidae) from Oita Prefecture, Japan, by nested polymerase chain reaction.

Pham XD(1), Otsuka Y, Suzuki H, Takaoka H.

Author information:
(1)Department of Infectious Disease Control, Oita Medical University, Hasama, Japan.

The current study surveyed the 56-kDa type-specific antigen (TSA) gene DNAs of

Orientia tsutsugamushi (Hayashi) in approximately 4.000 unengorged chiggers obtained from the soil or ground surface in an endemic and a nonendemic area of the Tsutsugamushi disease in Oita Prefecture, southwestern Japan, by nested polymerase chain reaction (PCR). Serotypes of O. tsutsugamushi were identified by restriction fragment-length polymorphism (RFLP) analysis. In the endemic area, 242 pools from five species [234 pools of Leptotrombidium scutellare (Nagayo, Miyagawa, Mitamura, Tamiya and Tenjin), two L. pallidum (Nagayo, Miyagawa, Mitamura and Tamiya), four L. kitasatoi (Fukuzumi & Obata), one L. fuji (Kuwata, Berge and Philip), and one Neotrombicula japonica (Tanaka, Kaiwa, Teramura and Kagaya)] were tested, and eight (seven pools of L. scutellare and one N. japonica) were positive for O. tsutsugamushi. Among the seven positive pools of L. scutellare, the distribution of serotypes was as follows: Kuroki (4), Gilliam (1), Karp (1), and Kawasaki (1). The first two serotypes (Kuroki and Gilliam) were identified for the first time in this species. In the nonendemic area, 128 pools from eight species were tested, and 13 were positive for O. tsutsugamushi. The positive rate was as follows: L. pallidum (4/41). L. kitasatoi (1/18), Gahrliepia saduski Womersley (2/10), L. fuji (4/50), L. himizu (Sasa, Kumada, Hayashi, Enomoto, Fukuzumi and Obata) (1/2), and Miyatrombicula kochiensis (Sasa, Kawashima and Egashira) (1/3). The latter three species were shown for the first time to harbor O. tsutsugamushi. All ofthe positive pools were Kuroki, except for two pools (one L. pallidum and one L. fuji), which were Gilliam (this serotype was also detected for the first time in L. pallidum). Further analysis revealed no differences in the nucleotide sequences (125 bp of variable domain 1 of TSA gene) of the same serotypes (i.e., Kuroki and Gilliam) among the positive samples. These data indicate that O. tsutsugamushi was widely distributed in various trombiculid species, even in the nonendemic area. The data are also suggestive of a possible horizontal transmission of O. tsutsugamushi among trombiculid species.

PMID: 11296840 [PubMed - indexed for MEDLINE]

787. Med Klin. 1975 Feb 14;70(7):249-54.

[Current significance of rickettsial diseases].

[Article in German]

Weyer F.

PMID: 47603 [PubMed - indexed for MEDLINE]

788. Commun Dis Intell Q Rep. 2005;29(2):206-25.

Communicable diseases surveillance: highlights for 1st quarter, 2005.

[No authors listed]

PMID: 16119769 [PubMed - indexed for MEDLINE]

789. Ann Intern Med. 1968 Mar;68(3):662-78.

Potential medical problems in personnel returning from Vietnam.

Gilbert DN, Moore WL Jr, Hedberg CL, Sanford JP.

PMID: 4311893 [PubMed - indexed for MEDLINE]

790. JAMA. 1969 Jan 27;207(4):697-702.

Public health problems relating to the Vietnam returnee.

Greenberg JH.

PMID: 4302950 [PubMed - indexed for MEDLINE]

791. Ann N Y Acad Sci. 2003 Jun;990:419-23.

Seroimmunological monitoring of several species of Rickettsiaceae and Bartonellaceae circulating in the Moscow region.

Ignatovich V(1), Penkina G, Umnova N.

Author information:
(1)The Gamaleya Research Institute of Epidemiology and Microbiology, Gamaleya Street 18, 123098 Moscow, Russia.

PMID: 12860667 [PubMed - indexed for MEDLINE]

792. J Epidemiol. 2007 Dec;17 Suppl:S48-55.

Epidemics of vector-borne diseases observed in infectious disease surveillance in Japan, 2000-2005.

Hashimoto S(1), Kawado M, Murakami Y, Izumida M, Ohta A, Tada Y, Shigematsu M, Yasui Y, Taniguchi K, Nagai M.

Author information:
(1)Department of Hygiene, Fujita Health University School of Medicine, 1-98, Kutsukake-cho, Toyoake, Aichi 470-1192, Japan. hasimoto@fujita-hu.ac.jp

BACKGROUND: Observing the epidemics of vector-borne diseases is important. One or more cases of 6 vector-borne diseases were reported to the National Epidemiological Surveillance of Infectious Diseases in Japan in 2000-2005.
METHODS: The reports of those cases were available. The incidence was observed by region of acquired infection, prefecture reporting, and week and year of diagnosis.

RESULTS: The incidence rate per year per 1,000,000 population was 0.36 for dengue fever, 0.04 for Japanese encephalitis, 0.38 for Japanese spotted fever, 0.08 for Lyme disease, 0.74 for malaria, and 3.50 for scrub typhus. There were no cases of dengue fever or malaria derived from domestic infections. The yearly incidence rate increased for dengue fever and Japanese spotted fever, and declined for malaria and scrub typhus. The proportion of cases reported in Tokyo was 44% for dengue fever and 37% for malaria. The number of prefectures reporting one or more cases of Japanese spotted fever increased in western Japan. The cases of scrub typhus increased in autumn-winter in prefectures of eastern Japan, and increased both in autumn-winter and spring in western prefectures.
CONCLUSIONS: The study reveals the epidemiologic features of both temporal and geographic distributions of cases of 6 vector-borne diseases in Japan, 2000-2005.

PMCID: PMC4809254
PMID: 18239342 [PubMed - indexed for MEDLINE]

793. Presse Med. 1968 Feb 3;76(6):259-62.

[How do rickettsial and related diseases appear at present?].

[Article in French]

Giroud P.

PMID: 4967919 [PubMed - indexed for MEDLINE]

794. Travel Med Infect Dis. 2011 May;9(3):95-105. doi: 10.1016/j.tmaid.2010.01.004. Epub 2010 Jun 11.

Review of vector-borne diseases in Hong Kong.

Ma SK(1), Wong WC, Leung CW, Lai ST, Lo YC, Wong KH, Chan MC, Que TL, Chow KW, Yuen MC, Lau TW, Simon J.

Author information:
(1)Enteric and Vector-borne Disease Office, Surveillance and Epidemiology Branch, Centre for Health Protection, Department of Health, Hong Kong Special Administrative Region Government, Hong Kong.

The epidemiology of vector-borne diseases in Hong Kong has changed over the past decade but still poses a significant public health risk. We provided a comprehensive review of the epidemiological information and analysed the trends of major vector-borne diseases, including the vector situation in Hong Kong. The incidence of malaria has dropped dramatically in the past few decades and is now mainly an imported disease acquired from malaria endemic countries. Locally acquired dengue fever occurred in 2002 and 2003, and thereafter all cases were imported, mainly from Southeast Asia areas. Only a few local cases of Japanese encephalitis were reported in the past decade. In contrast, there is a notable increase in scrub typhus and spotted fever cases. The emergence of chikungunya fever in Asia and Indian Ocean countries also resulted in importation of human

cases. Given the heavy traffic between this international city and other parts of the world, as well as the presence of vectors in this densely populated area, vigilance should be maintained against these infections. Comprehensive public health measures encompassing disease surveillance, vector surveillance and control measures with support from all sectors of the community are required to combat the old and newly emerging vector-borne diseases in Hong Kong.

Copyright © 2010 Elsevier Ltd. All rights reserved.

DOI: 10.1016/j.tmaid.2010.01.004
PMID: 21679886 [PubMed - indexed for MEDLINE]

795. Korean J Parasitol. 1996 Mar;34(1):27-33.

[Population density of chigger mites, the vector of tsutsugamushi disease in Chollanam-do, Korea].

[Article in Korean]

Song HJ(1), Kim KH, Kim SC, Hong SS, Ree HI.

Author information:
(1)Division of Microbiology, Health and Environment Institute of Chollanam-do, Kwang-ju, Korea.

The geographical distribution and population density of rodents and chigger mites at six localities of Chollanam-do were investigated from October to December in 1993. Among total 142 field rodents collected by the modified Chemla wooden traps, 131 rodents were Apodemus agrarius (92.3%) and 11 were Crocidura lasiura (7.7%). Out of 142 field rodents, 92 were parasitized by chiggers, showing 69.0% of the infestation rate and 74.2 of the chigger index. Infestation rate and chigger index of A. agrarius and C. lasiura were 73.3%, 80.4 and 18.2%, 0.5, respectively. From the trapped field rodents, 10,532 chiggers were collected and identified with 11 species of 4 genera. Leptotrombidium pallidum, the vector species of tsutsugamushi disease, was the dominant species, showing 8,038 chiggers (76.3%). L. scutellare was the second dominant species showing 1,359 chiggers (12.9%). The distribution of chigger mites was clearly localized by the species, showing the different dominant species according to localities. The predominant species was L. scutellare (100%) in Changhung-gun, L. scutellare (41.5%) in Posong-gun, L. pallidum (88.8%) in Hwasun-gun, L. pallidum (59.2%) in Koksong-gun, L. zetum (77.3%) in Hampyong-gun, and L. palpale (63.4%) in Tamyang-gun. Regarding to the geographical distribution of chigger population density, the infestation rate and chigger index was most high in Hwasun-gun as 62.4% and 216.2 respectively, and next high in Koksong-gun as 22.4% and 77.7% respectively.

PMID: 8820739 [PubMed - indexed for MEDLINE]

796. J Vector Ecol. 1998 Jun;23(1):1-46.

Vectors vs. humans in Australia--who is on top down under? An update on

vector-borne disease and research on vectors in Australia.

Russell RC(1).

Author information:
(1)Department of Medical Entomology, University of Sydney, Westmead, NSW, Australia.

Australia has a diversity of vectors and vector-borne human diseases. Mosquito-borne arboviruses are of greatest concern, but there are issues with other vector and pathogen systems. Mosquitoes were responsible for more than 35,000 cases of Ross River virus during 1991-1997. Barmah Forest virus is increasing nationwide, and unidentified bunyaviruses suspected of causing illness have been isolated. Cases of Murray Valley encephalitis have occurred in 14 of the past 20 years in northern Australia. Dengue is a continuing problem for northern Queensland, with various serotypes being active. Japanese encephalitis has appeared in the Torres Strait Islands and threatens mainland Australia. Although malaria is eradicated, almost 1,000 cases are imported annually and occasional cases of local transmission occur. With ticks, paralysis in children occurs annually in eastern Australia. Tick typhus (Queensland Tick Typhus--Rickettsia australis) occurs down the east coast, and (Flinders Island Spotted Fever--Rickettsia honei) in Bass Strait and probably Tasmania. Lyme disease is reported but its presence is controversial. Fleas were responsible for a recent outbreak of murine typhus (Rickettsia typhi) in Western Australia. Mites cause scrub typhus (Orientia tsutsugamushi), and there was a recent fatality in the Northern Territory. Overall, resources for investigation and control of vector-borne disease have generally been meager. However, various avenues of basic and applied research have been pursued, and have included investigations into mosquito ecology, vector competence, disease epidemiology, and vector control. Disease surveillance programs vary between states, and mosquito control programs are organized and effective in only a few regions. There are concerns for import of vectors such as Aedes albopictus and export of pathogens such as Ross River virus; the former has occurred but the species has not become established, and the latter has occurred and has resulted in a major outbreak in the South Pacific. The predicted scenarios of increased temperature and rainfall with global warming are also causing concern for increases in vector-borne diseases, particularly the endemic arboviruses. Interest by health authorities is gravitating more towards epidemiological reporting and less towards public health action. In many respects, humans have much to do to get 'on top' of vectors and their pathogens 'down under' in Australia.

PMID: 9673928 [PubMed - indexed for MEDLINE]

797. Southeast Asian J Trop Med Public Health. 2000 Dec;31(4):733-5.

Rickettsial infection in five remote Orang Ulu villages in upper Rejang River, Sarawak, Malaysia.

Sagin DD(1), Ismail G, Nasian LM, Jok JJ, Pang EK.

Author information:
(1)Faculty of Medicine and Health Sciences, Universiti Malaysia Sarawak, Kota

Samarahan. dsagin@fhs.unimas.my

People in 5 Orang Ulu villages in Sarawak, Malaysia were tested for rickettsial infection by Weil-Felix reaction and by indirect immunoperoxidase reaction. Of those surveyed 9.6% were positive for typhus. Of the positives, 3.8% were positive for tick typhus (7/11), scrub typhus (4/11) or endemic typhus (1/11). The incidence of typhus was higher among semi-nomadic Penans compared with the settled Kayans.

PMID: 11414421 [PubMed - indexed for MEDLINE]

798. Mil Med. 1969 Jan;134(1):36-42.

Observations on fevers of unknown origin in the Rephblic of Vietnam.

Reiley CG, Russell PK.

PMID: 4991620 [PubMed - indexed for MEDLINE]

799. Med Vet Entomol. 1992 Oct;6(4):389-95.

Chigger mites of the genus Leptotrombidium: key to species and their distribution in China.

Wang DQ(1), Yu ZZ.

Author information:
(1)Department of Parasitology, Fujian Medical College, Fuzhou, China.

Chigger mites of the genus Leptotrombidium (Acari: Trombiculidae) transmit scrub typhus, caused by Rickettsia tsutsugamushi (= R.orientalis) in South-East Asia. In China, eighty-two species of Leptotrombidium have been recorded; these are listed with the names of Provinces where they were found. Five species, L.deliense, L.insularae, L.kaohuense, L.rubellum and L.scutellare, have been implicated as Chinese vectors of scrub typhus. A brief key is given to the larvae of all but three of the Leptotrombidium mites known in China.

PMID: 1463907 [PubMed - indexed for MEDLINE]

800. Kansenshogaku Zasshi. 1995 Oct;69(10):1110-7.

[Studies on tsutsugamushi disease in Gifu prefecture. 6. Correlation between number of patients and meteorological elements].

[Article in Japanese]

Kasuya S(1).

Author information:
(1)Department of Parasitology, Gifu University School of Medicine.

The correlations between numbers of tsutsugamushi disease patients and meteorological elements were analyzed for 11 years from 1982 to 1992 in Gifu Prefecture, Japan by using regression analysis. The number of patients in early winter was closely correlated independently to both the mean of the minimum temperatures from 11th May to 31st July and the mean of the maximum temperatures in November in the same year. Regression coefficients (R2) were 0.689 and 0.560, respectively. On this basis, an equation for prediction of the number of patients in early winter was designed as follows: N = [e (j - 17.6) + 2.3(v - 13)] x j x v/156 (prediction formula 1) N:predicted number of patients in early winter j:the mean of the minimum temperature from 11th May to 31st July v: the mean of the maximum temperature in November e:the base of the natural logarithm (= 2.718...) The number of patients in early winter was also closely correlated to j in an equation of the fifth degree (R2 = 0.930). N = 22.524656384 j5 - 2218.23705 j4 + 87272.992 j3 - 1714734.329 j2 + 16825634.235 j - 65963810.254 Based on these formulas, the temperature in early summer has a significant effect upon the prevalence of tsutsugamushi disease in early winter.

PMID: 7499913 [PubMed - indexed for MEDLINE]

801. Kansenshogaku Zasshi. 1988 Aug;62(8):753-9.

[A case of Tsutsugamushi disease, occurring in the rural area of Tama, Tokyo].

[Article in Japanese]

Ito T, Yamazaki K, Nakamura K, Shinkai T, Terayama T, Yabuuchi K, Ohashi M, Matsui K.

PMID: 3150419 [PubMed - indexed for MEDLINE]

802. Kansenshogaku Zasshi. 1979 Aug;53(8):374-8.

[A case of tsutsugamushi disease infected at the foot of Mt. Fuji (author's transl)].

[Article in Japanese]

Shoda M, Takigami T, Murata M, Kawamura A.

PMID: 119818 [PubMed - indexed for MEDLINE]

803. Parasitology. 2011 Mar;138(3):344-53. doi: 10.1017/S003118201000140X. Epub 2010 Oct 15.

Variation within and among host species in engorgement of larval trombiculid

mites.

Kuo CC(1), Wang HC, Huang CL.

Author information:
(1)Department of Wildlife, Fish, & Conservation Biology, University of
California, Davis, One Shields Avenue, Davis, CA 95616, USA. ccckuo@ucdavis.edu

We recovered larval trombiculid mites (i.e. chiggers), vectors of scrub typhus, from small mammal hosts in Taiwan, and compared the relative degree of engorgement (RDE) of the dominant chigger (Leptotrombidium imphalum) from different hosts. Naturally occurring chiggers recovered from Rattus losea and Bandicota indica were 1·4x and 1·3x as engorged as those from Apodemus agrarius. Within each host species, RDE was negatively related to chigger loads, but was mostly unrelated to gender or to body or reproductive condition of hosts. We documented significant variation in chigger engorgement both within and among host species; to the extent that RDE is a proxy for fitness, this contradicts predictions of the Ideal Free Distribution (IFD) that the per capita fitness of vectors should be similar among hosts. Failure to meet predictions of the IFD may reflect the limited mobility of chiggers, which consequently must be less selective in the hosts on which they feed. Further disease control efforts should consider vector feeding success in addition to vector abundance and may be able to capitalize on the unsuitability of certain hosts in supporting disease vectors.

DOI: 10.1017/S003118201000140X
PMID: 20946695 [PubMed - indexed for MEDLINE]

804. Chin Med J (Engl). 1996 Sep;109(9):670-3.

The role of Leptotrombidium scutellare in the transmission of human diseases.

Wu G(1), Zhang Y, Guo H, Jiang K, Zhang J, Gan Y.

Author information:
(1)Institute of Military Medicine, Nanjing.

OBJECTIVE: To investigate the role of Leptotrombidium (L.) scutellare in the transmission of hemorrhagic fever with renal syndrome (HFRS) and tsutsugamushi disease of autumn-type.
MATERIALS AND METHODS: Four criteria were used to ascertain this mite as transmitting vector of disease: (1) epidemiological evidence; (2) natural infection of pathogen; (3) ability to transmit pathogen via biting; (4) ability to transfer pathogen via transovarian route.
RESULTS: The results indicated that L. scutellare was the dominant species of chigger mite on rats. Its seasonal distribution was correlated with the incidence of these two diseases. This mite could be naturally infected by HFRS virus (HFRSV) and Rickettsia tsutsugamushi (Rt) and the mite could transmit HFRSV and Rt to vertebrates by biting and to its offspring via transovarian transmission.
CONCLUSIONS: These results have proved that L. scutellare can be a transmitting vector of HFRS and tsutsugamushi disease of autumn-type.

PMID: 9275333 [PubMed - indexed for MEDLINE]

805. Zhonghua Yi Xue Za Zhi. 1994 Feb;74(2):94-6, 127.

[Leptotrombidium (L.) scutellare as the transmitting vector of tsutsugamushi disease of autumn-winter type in Jiangsu Province].

[Article in Chinese]

Wu GH(1), Guo HB, Yu MM.

Author information:
(1)Institute of Military Medicine, Nanjing Command.

In 1986, epidemic of tsutsugamushi disease of autumn-winter type was found in Jiangsu province. To clarify the vector of this disease, we carried out a series of studies in 1986-1992. Leptotrombidium (L.) scutellare was found to be a dominant species of chigger mite on rats in the endemic areas and its seasonal distribution was correlated with the incidence of tsutsugamushi disease in inhabitants. This mite could naturally be infected by Rickettsia tsutsugamushi, and R. tsutsugamushi could be transmitted via biting and transovarial transmission. Specific antibodies could be detected in the sera of mice bitten by the mites or inoculated with the suspension of mites. Serological typing of the sera of mice was of Gilliam type. The above results demonstrate that L. (L.) scutellare can serve as transmitting vector of tsutsugamushi disease of autumn-winter type.

PMID: 8069730 [PubMed - indexed for MEDLINE]

806. Med Parazitol (Mosk). 1972 May-Jun;41(3):287-90.

[Trombiculid mites and foci of tsutsugamushi fever in Tadzhikistan].

[Article in Russian]

Kudriashova NI.

PMID: 4262458 [PubMed - indexed for MEDLINE]

807. Zh Mikrobiol Epidemiol Immunobiol. 1976 Feb;(2):124-7.

[Sporadic cases of tsutsugamushi fever on Kamchatka].

[Article in Russian]

Zak MR.

PMID: 1266453 [PubMed - indexed for MEDLINE]

808. Zh Mikrobiol Epidemiol Immunobiol. 1970 Sep;47(9):112-5.

[Vectors of tsutsugamushi fever in the Far East].

[Article in Russian]

Shubin FN, Natskiĭ KV, Somov GP.

PMID: 5519097 [PubMed - indexed for MEDLINE]

809. Indian J Med Microbiol. 2009 Jul-Sep;27(3):267-70. doi: 10.4103/0255-0857.53215.

Hantavirus infection: a case report from India.

Chandy S(1), Boorugu H, Chrispal A, Thomas K, Abraham P, Sridharan G.

Author information:
(1)Department of Clinical Virology, Christian Medical College, Vellore, India.

The clinical presentation of hantavirus infections in India is unclear. We report here a case of hantavirus infection in a 46 year old quarry worker presenting with fever, abdominal pain, jaundice, thrombocytopenia and renal dysfunction. Seroconversion and rising anti-hantavirus IgG titers were taken as evidence of hantavirus infection. Clinicians should consider hantavirus infections in the differential diagnosis of acute febrile illness along with scrub typhus, leptospirosis and dengue.

DOI: 10.4103/0255-0857.53215
PMID: 19584514 [PubMed - indexed for MEDLINE]

810. BMJ. 2001 Sep 29;323(7315):714.

India wakes up to threat of bioterrorism.

Sharma R.

PMCID: PMC1121283
PMID: 11576976 [PubMed - indexed for MEDLINE]

811. J Assoc Physicians India. 2006 Apr;54:291-8.

Tropical infections in the ICU.

Kothari VM(1), Karnad DR, Bichile LS.

Author information:
(1)Medical Intensive Care Unit, Department of Medicine, Seth GS Medical College

and KEM College, Mumbai.

Certain arthropod-borne infections are common in tropical regions because of favorable climatic conditions. Water-borne infections like leptospirosis are common due to contamination of water especially during the monsoon floods. Infections like malaria, leptospirosis, dengue fever and typhus sometimes cause life threatening organ dysfunction and have several overlapping features. Most patients present with classicial clinical syndromes: fever and thrombocytopenia are common in dengue, malaria and leptospirosis; coagulopathy is frequent in leptospirosis and viral hepatitis. Hepatorenal syndrome is seen in leptospirosis, falciparum malaria and scrub typhus. The pulmonary renal syndrome is caused by falciparium malaria, leptospirosis, Hantavirus infection and scrub typhus. Fever with altered mental status is produced by bacterial meningitis, Japanese B encephalitis, cerebral malarial, typhoid encephalopathy and fulminant hepatic failure due to viral hepatitis. Subtle differences in features of the organ failure exist among these infections. The diagnosis in some of these diseases is made by demonstration of antibodies in serum, and these may be negative in the first week of the illness. Hence empiric therapy for more than one disorder may be justified in a small proportion of cases. In addition to specific anti-infective therapy, management of organ dysfunction includes use of mechanical ventilation, vasopressor drugs, continuous renal replacement therapy and blood products. Timely transfer of these patients to well-equipped ICUs with experience in managing these cases can considerably decrease mortality and morbidity.

PMID: 16944613 [PubMed - indexed for MEDLINE]

812. Trop Dis Bull. 1970 Aug;67(8):913-20.

Summary of recent abstracts. VII. Rickettsial diseases.

Hennessey RS.

PMID: 4992946 [PubMed - indexed for MEDLINE]

813. Ann N Y Acad Sci. 2006 Oct;1078:143-9.

Current knowledge of rickettsial diseases in Italy.

Ciceroni L(1), Pinto A, Ciarrocchi S, Ciervo A.

Author information:
(1)Department of Infectious, Parasitic and Immune-Mediated Diseases, Istituto Superiore di Sanità, Viale Regina Elena 299, 00161 Rome, Italy. ciceroni@iss.it

Rickettsial diseases continue to be the cause of serious health problems in Italy. From 1998 to 2002, 4,604 clinical cases were reported, with 33 deaths in the period from 1998 to 2001. Almost all the cases reported in Italy are cases of Mediterranean spotted fever (MSF). Other rickettsioses that have been historically documented are murine typhus and epidemic typhus. Since 1950, only

sporadic cases of murine typhus have been reported, and Italy currently appears to be free of epidemic typhus. As in other European countries, imported cases of rickettsialpox, African tick-bite fever (ATBF), and scrub typhus have been reported. In 2004, three cases of a mild form of rickettsiosis were serologically attributed to Rickettsia helvetica.

DOI: 10.1196/annals.1374.024
PMID: 17114696 [PubMed - indexed for MEDLINE]

814. Bull Soc Pathol Exot Filiales. 1969;62(2):288-95.

[Rickettsioses endemic to the USSR and their reservoirs].

[Article in French]

Zdrodowski P, Golinevitch H.

PMID: 5409108 [PubMed - indexed for MEDLINE]

815. J Med Entomol. 1994 Sep;31(5):691-9.

Detection and characterization of Rickettsia tsutsugamushi (Rickettsiales: Rickettsiaceae) in infected Leptotrombidium (Leptotrombidium) fletcheri chiggers (Acari: Trombiculidae) with the polymerase chain reaction.

Kelly DJ(1), Dasch GA, Chan TC, Ho TM.

Author information:
(1)Naval Medical Research Institute, Bethesda, MD 20889-5607.

We developed a method for detecting and characterizing the DNA of Rickettsia tsutsugamushi in chiggers (larval trombiculid mites) by polymerase chain reaction (PCR). Three procedures for extracting DNA from frozen chiggers were compared by evaluating the yield of PCR amplicand obtained with nine oligonucleotide primer pairs derived from the rickettsial 22 kD, 47 kD, groESL, 56 kD, and 110 kD antigen genes. Although extracts and primer pairs differed in amplification efficiency, R. tsutsugamushi DNA was successfully detected in extracts of colonized infected Leptotrombidium (Leptotrombidium) fletcheri (Wormersley & Heaslip) chiggers and in uninfected chigger extracts seeded with known amounts of Karp-strain rickettsiae. The 22 kD gene restriction fragment length polymorphisms (RFLP) observed in PCR amplicands from five rickettsial isolates obtained from the infected chigger colony over a 26-yr period were identical to those of PCR amplicands derived directly from infected chiggers taken from the same colony. This suggests that stable transmission of R. tsutsugamushi occurs in mites (62 generations), and isolates encompass the full genetic heterogeneity found in the chigger. PCR/RFLP analysis is an important new tool for investigating the complex epidemiology of scrub typhus rickettsiae in their mite vectors.

PMID: 7966172 [PubMed - indexed for MEDLINE]

816. Aust J Exp Biol Med Sci. 1978 Apr;56(2):147-56.

Rodent zoonoses in North Queensland: the occurrence and distribution of zoonotic infections in North Queensland rodents.

Glazebrook JS, Campbell RS, Hutchinson GW, Stallman ND.

A study of potentially zoonotic infections was carried out on 351 rodents trapped in north-eastern Queensland. Their ecosystems included towns, agricultural and livestock areas, wookland and rainforest. Nine serotypes of salmonellae were obtained from asymptomatic carries in predominantly settled locations. Two strains of Ps. pseudomallei occurred in rainforest near Innisfail and one on a cattle property adjacent to Townsville. Ps. aeruginosa caused bronchopneumonia in one animal from Townsville harbour. Ifection by leptospirae of six serogroups and seven serovars were identified by serological or cultural examinations. Enzootic foci occurred on the Mount Spec rainforest where celledoni and australis were being excreted by rats adjacent to the Paluma dam system. In addition to the scrub typhus locations at Rocky Creek, Atherton Tableland and Bullocky Creek, near Ingham, which were confirmed, a new focus of infection by R. tsutsugamushi was identified at El Arish near Tully. Water rat (H. chrysogaster) at Townsville harbour constituted a reservoir of toxoplasmosis. In addition to the known human pathogenic helminths H. nana and H. diminuta, localized foci of hookworms (Ancylostoma spp.) were found. Histological evidence of cytomegalic disease of the salivary glands or kidneys was a common finding.

PMID: 678225 [PubMed - indexed for MEDLINE]

817. Nihon Naika Gakkai Zasshi. 1987 May;76(5):736-9.

[A fatal case of probable Tsutsugamushi disease in Saitama Prefecture, with particular reference to 8 fatal cases occurring in Japan from 1962 to 1985].

[Article in Japanese]

Sakai H, Honda K, Oowada A, Shimada N.

PMID: 3334393 [PubMed - indexed for MEDLINE]

818. Int J Zoonoses. 1980 Jun;7(1):1-14.

Surveillance of small mammals and their flea-indices in plague endemic area at Boyolali, Central Java, Indonesia.

Kusharyono C, Udayati, Sustriayu N, Liat LB.

PMID: 7461913 [PubMed - indexed for MEDLINE]

819. Chin Med J (Engl). 2002 Dec;115(12):1881-2.

Genotype identification of Orientia tsutsugamushi isolated from Nan Peng Lie Islands in China.

Peng G(1), Wang Z, Wang S, Huang J, Jiang P, Zeng N, Liu J, Zhu S.

Author information:
(1)Department of Epidemiology, Medical Institute, Guangzhou Command PLA, Guangzhou 510507, China.

OBJECTIVE: To identify genotype of eight strains of Orientia tsutsugamushi (O. tsutsugamushi) isolated from Nan Peng Lie Islands in China and establish tsutsugamushi disease nature foci for this region.
METHODS: The nested polymerase chain reaction and restriction fragment length polymorphism (PCR-RFLP) were used. Three primers were selected from the DNA sequence of the gene encoding type-specific 56-kDa protein of the Karp strain. The positive products were digested by Hine II and Pst I, meanwhile profiles specific to each strain were analyzed.
RESULTS: Three genotypes of O. tsutsugamushi including Karp, Kato and a new strain existed on Nan Peng Lie Islands.
CONCLUSION: Nan Peng Lie Islands is tsutsugamushi disease nature foci.

PMID: 12622944 [PubMed - indexed for MEDLINE]

820. J Commun Dis. 2010 Sep;42(3):209-13.

Serological study of rickettsial diseases in human and rodent population in Chittoor dist. (A.P.).

Prabhakaran A(1), Lal S, Biswas S, Vinoth S, Asraf AS, Mittal V.

Author information:
(1)Department of Zoology, Presidency College, Chennai.

Erratum in
J Commun Dis. 2010 Sep;42(3):2 p preceding 171.

In India the presence of Rickettsial disease in human is documented in many states however, the data on presence of Rickettsial infection in Andhra Pradesh is very scare. Therefore, a study was undertaken in Chittoor district (A.P.) to see the prevalence of Rickettsial infection in human and rodent population. 3-5 ml of human blood samples were collected from the patients attending the nearest hospitals of Tirumala, Tirupathi, Palmner and Chittoor areas. Live rodents were trapped and blood samples were collected from them during January and February 2008. Sera was separated and tested by Weil Felix test. Two hundred human sera samples were tested. Of these 39 samples were found reactive with Weil Felix antigen. Of the 39 reactive, 31 were male and 8 female. All the human samples were showing reactivity at 1:20 dilution. Out of the 343 rodents samples tested, only 24 samples were showing reactivity. These were reactive at 1:20, 1:40 and 1:80 dilutions with different types of Weil Felix antigens. Eight rodent sera samples were having titer 1:80 with Proteus OXK which is suggestive of presence

of Scrub typhus in this region.

PMID: 22471185 [PubMed - indexed for MEDLINE]

821. Nihon Naika Gakkai Zasshi. 1997 Apr 10;86(4):681-3.

[Case of a new type of Tsutsugamushi disease associated with meningoencephalitis].

[Article in Japanese]

Takekuma K, Arai Y, Yamauchi H, Makino H, Takazakura E, Fukui Y, Yasui S.

PMID: 9198662 [PubMed - indexed for MEDLINE]

822. Nihon Shokakibyo Gakkai Zasshi. 1991 Nov;88(11):2827-30.

[A case of hepatitis with granulomatous lesions by Rickettsia tsutsugamushi].

[Article in Japanese]

Satoh T(1), Nagai K, Katoh T, Ishii K, Aikawa K, Inoue K, Hosaka H, Itoh K, Abei T.

Author information:
(1)Second Department of Internal Medicine, Toho University School of Medicine, Tokyo, Japan.

PMID: 1766140 [PubMed - indexed for MEDLINE]

823. Nihon Naika Gakkai Zasshi. 1982 Jan 10;71(1):59-64.

[An autopsy case of tsutsugamushi disease complicated with disseminated intravascular coagulation syndrome (author's transl)].

[Article in Japanese]

Akai H, Ito M, Shindo T, Yamazaki H, Ishizuka Y, Shozawa T.

PMID: 7086252 [PubMed - indexed for MEDLINE]

824. Jpn J Antibiot. 1980 Nov;33(11):1223-31.

[Clinical studies on dry filled doxycycline for intravenous administration; including one case of tsutsugamushi disease (author's transl)].

[Article in Japanese]

Kanazawa Y.

On a new dry filled doxycycline derivative for intravenous administration, some clinical studies were performed. The results obtained were summarized as follows; 1. Intravenous administration was effective in three cases of pulmonary abscess, in one case of bronchopneumonia, in one case of urinary tract infection complicated to nephrolithiasis, in one case of Mycoplasma pneumonia and in one case of tsutsugamushi disease. 2. In one case of FUO (fever of unknown origin), in one case of non-bacterial meningitis and in one case of ampicillin induced hemorrhagic colitis, from whom Klebsiella oxytoca was isolated in feces, satisfactory clinical course was observed with the drug administration. However, as it was impossible to give any comments for the causative agent of each disease, the effectiveness of the derivative was not necessarily clear. 3. Neither side effects nor abnormal laboratory finding caused by the derivative were noticed in any cases.

PMID: 7241797 [PubMed - indexed for MEDLINE]

825. Am J Pathol. 1946 Jan;22:89-101.

Growth of the rickettsiae of Tsutsugamushi fever on the chorioallantoic membrane of the developing chick embryo.

HAMILTON HL.

PMID: 21009181 [PubMed - indexed for MEDLINE]

826. Nihon Naika Gakkai Zasshi. 2002 Feb 10;91(2):746-8.

[Two cases of rickettsiosis--the clinical characteristics of Japanese spotted fever and tsutsugamushi disease].

[Article in Japanese]

Yamane T(1), Arai T, Yamami A, Morio T, Takao K, Morio K, Kuwata S, Matsuzaki K, Nakamura T.

Author information:
(1)Third Department of Internal Medicine, Teikyo University School of Medicine, Ichihara.

PMID: 11917502 [PubMed - indexed for MEDLINE]

827. Tohoku J Exp Med. 1996 Jul;179(3):213-7.

Liver involvement in Tsutsugamushi disease.

Kanno A(1), Yamada M, Murakami K, Torinuki W.

Author information:
(1)Department of Internal Medicine, Tohoku Koseinenkin Hospital, Sendai, Japan.

Tsutsugamushi disease, one of the rickettsiosis, is known to be occasionally accompanied by elevation of hepatic enzyme levels. However, there are only a few reports on histopathological findings of the liver. We presented a case of Tsutsugamushi disease with liver involvement. A 51-year-old man suffered from eruptions and a high fever with a mild transaminasemia. He was diagnosed as Tsutsugamushi disease by detection of IgM class antibody against Rickettsia tsutsugamushi. Laparoscopic examination showed a dark-brown liver with diffuse whitish markings. Microscopic findings were consistent with the features of non-specific reactive hepatitis: sinusoidal small lymphocyte infiltrations, mild disarray of hepatocytes and aggregation of T lymphocytes and macrophages in the lobule.

PMID: 8888510 [PubMed - indexed for MEDLINE]

828. Kansenshogaku Zasshi. 1996 May;70(5):516-9.

[A case of Tsutsugamushi disease infected by mountain climbing in the Republic of Korea].

[Article in Japanese]

Kitahama S(1), Suzuki J, Kawakami Y.

Author information:
(1)First Department of Medicine, Hokkaido University School of Medicine, Japan.

A 47-year-old male was infected with Tsutsugamushi disease showing typical findings after mountain climbing in the Republic of Korea (ROK). Immunoserological examinations suggested that he had been infected in ROK. In this case, detection of the characteristic sting was useful for early diagnosis and treatment. Until now, Tsusugamushi disease has not been reported in Hokkaido. It is the first case registered in Hokkaido. With extensive international intercourse these days, this case suggests that Tsutsugamushi disease can occur potentially all over Japan.

PMID: 8699103 [PubMed - indexed for MEDLINE]

829. Lancet. 1994 May 14;343(8907):1236.

Fulminant Rickettsia tsutsugamushi infection associated with haemophagocytic syndrome.

Iwasaki H, Hashimoto K, Takada N, Nakayama T, Ueda T, Nakamura T.

PMID: 7909908 [PubMed - indexed for MEDLINE]

830. Zhonghua Min Guo Xiao Er Ke Yi Xue Hui Za Zhi. 1988 May-Jun;29(3):180-3.

[Meningitis associated with Rickettsia tsutsugamushi infection].

[Article in Chinese]

Lin YC, Lo ZJ.

PMID: 3272991 [PubMed - indexed for MEDLINE]

831. J Med Entomol. 2016 Jul 29. pii: tjw111. [Epub ahead of print]

Development of a Rickettsia bellii-Specific TaqMan Assay Targeting the Citrate Synthase Gene.

Hecht JA(1), Allerdice ME(2), Krawczak FS(2), Labruna MB(2), Paddock CD(2), Karpathy SE(2).

Author information:
(1)Rickettsial Zoonoses Branch, National Center for Emerging and Zoonotic Infectious Diseases, Centers for Disease Control and Prevention, Atlanta, GA (jhecht@cdc.gov; mallerdice@cdc.gov; CPaddock@cdc.gov; skarpathy@cdc.gov) jhecht@cdc.gov. (2)Rickettsial Zoonoses Branch, National Center for Emerging and Zoonotic Infectious Diseases, Centers for Disease Control and Prevention, Atlanta, GA (jhecht@cdc.gov; mallerdice@cdc.gov; CPaddock@cdc.gov; skarpathy@cdc.gov).

Rickettsia bellii is a rickettsial species of unknown pathogenicity that infects argasid and ixodid ticks throughout the Americas. Many molecular assays used to detect spotted fever group (SFG) Rickettsia species do not detect R. bellii, so that infection with this bacterium may be concealed in tick populations when assays are used that screen specifically for SFG rickettsiae. We describe the development and validation of a R. bellii-specific, quantitative, real-time PCR TaqMan assay that targets a segment of the citrate synthase (gltA) gene. The specificity of this assay was validated against a panel of DNA samples that included 26 species of Rickettsia, Orientia, Ehrlichia, Anaplasma, and Bartonella, five samples of tick and human DNA, and DNA from 20 isolates of R. bellii, including 11 from North America and nine from South America. A R. bellii control plasmid was constructed, and serial dilutions of the plasmid were used to determine the limit of detection of the assay to be one copy per 4 µl of template DNA. This assay can be used to better determine the role of R. bellii in the epidemiology of tick-borne rickettsioses in the Western Hemisphere.

© The Authors 2016. Published by Oxford University Press on behalf of Entomological Society of America. All rights reserved. For Permissions, please email: journals.permissions@oup.com.

DOI: 10.1093/jme/tjw111

PMID: 27473178 [PubMed - as supplied by publisher]

832. Emerg Infect Dis. 2009 Nov;15(11):1791-8. doi: 10.3201/eid1511.090677.

Multicenter GeoSentinel analysis of rickettsial diseases in international travelers, 1996-2008.

Jensenius M(1), Davis X, von Sonnenburg F, Schwartz E, Keystone JS, Leder K, Lopéz-Véléz R, Caumes E, Cramer JP, Chen L, Parola P; GeoSentinel Surveillance Network.

Collaborators: Kain KC, Kozarsky PE, Franco-Paredes C, Loutan L, Chappuis F, Torresi J, Brown G, Hale DC, Gelman SS, Pérignon A, Burchard GD, Wilson ME, Simon F, Delmont J, Stauffer WM, Walker PF, Lim PL, Wilder-Smith A, Perez Molina JA, Connor BA, Licitra C, Crespo A, Freedman DO, Gkrania-Klotsas E, Carosi G, Castelli F, Shaw M, Pandey P, Sack RB, McKenzie R, Barnett ED, Coyle CM, Wittner M, Hagmann S, Miller A, Lynch MW, Field V, Libman MD, Maclean JD, Gurtman A, Kanagawa S, Kato Y, Schlagenhauf P, Weber R, Steffen R.

Author information:
(1)Department of Infectious Diseases, Oslo University Hospital, Ullevål, NO-0407 Oslo, Norway. mogens.jensenius@ioks.uio.no

We investigated epidemiologic and clinical aspects of rickettsial diseases in 280 international travelers reported to the GeoSentinel surveillance Network during 1996-2008. Of these 280 travelers, 231 (82.5%) had spotted fever (SFG) rickettsiosis, 16 (5.7%) scrub typhus, 11 (3.9%) Q fever, 10 (3.6%) typhus group (TG) rickettsiosis, 7 (2.5%) bartonellosis, 4 (1.4%) indeterminable SFG/TG rickettsiosis, and 1 (0.4%) human granulocytic anaplasmosis. One hundred ninety-seven (87.6%) SFG rickettsiosis cases were acquired in sub-Saharan Africa and were associated with higher age, male gender, travel to southern Africa, late summer season travel, and travel for tourism. More than 90% of patients with rickettsial disease were treated with doxycycline, 43 (15.4%) were hospitalized, and 4 had a complicated course, including 1 fatal case of scrub typhus encephalitis acquired in Thailand.

DOI: 10.3201/eid1511.090677
PMCID: PMC2857242
PMID: 19891867 [PubMed - indexed for MEDLINE]

833. J Med Entomol. 1999 Nov;36(6):869-74.

Seasonal occurrence of Leptotrombidium deliense (Acari: Trombiculidae) attached to sentinel rodents in an orchard near Bangkok, Thailand.

Frances SP(1), Watcharapichat P, Phulsuksombati D, Tanskul P, Linthicum KJ.

Author information:
(1)Australian Army Malaria Institute, Enoggera, Queensland, Australia.

Leptotrombidium deliense Walch that attached to sentinel laboratory mice and the roof rat, Rattus rattus (L.), placed in an orchard habitat near Bangkok,

Thailand, were studied between April 1993 and April 1995. A single L. deliense larva was attached to only 1 of 51 laboratory mice placed in the study area between April and September 1993. Overall, 89/202 (44.1%) R. rattus had 1 or more L. deliense larvae attached, and Orientia tsutsugamushi (Hayashi), the etiologic agent for scrub typhus, was isolated from liver/spleen samples of 2/202 (1.0%) rats placed in an endemic area for a single night. A total of 474 L. deliense attached to sentinel R. rattus, of which 314 larvae successfully fed to repletion and were recovered, and 2 (0.6%) of these were naturally infected with O. tsutsugamushi. The occurrence of L. deliense was influenced by rainfall, with more chiggers attached to rodents in the wetter months of the year. The study showed that the risk of exposure to infection with O. tsutsugamushi is greater during the wetter months of the year, and that only a relatively small number of chigger attachments are needed to infect potential hosts.

PMID: 10593093 [PubMed - indexed for MEDLINE]

834. Southeast Asian J Trop Med Public Health. 2006 Jan;37(1):1-4.

Possible acute coinfections in Thai malaria patients.

Singhsilarak T(1), Phongtananant S, Jenjittikul M, Watt G, Tangpakdee N, Popak N, Chalermrut K, Looareesuwan S.

Author information:
(1)Department of Clinical Tropical Medicine, Faculty of Tropical Medicine, Mahidol University, Bangkok, Thailand.

We conducted serodiagnostic testing for dengue virus infection, murine typhus, scrub typhus and leptospirosis in Plasmodium falciparum-infected individuals in Thailand. Sera from 194 malaria patients with a median age of 24 years were tested. No antibody titers diagnostic of dengue virus infection were demonstrated, but 29 (15%) of patients had serological evidence of scrub typhus, 45 (23.2 %) patients had evidence of murine typhus, and 15 (7.7%) sera tested positive for leptospirosis. Our serological results suggested that duel infections are not uncommon in malaria that is acquired in Thailand. However, our results must be confirmed by prospective studies aimed at describing the causative organisms. Mixed infections would have multiple implications for clinicians, including unexpected clinical findings and apparent poor responses to antimalarial treatment in patients thought only to have malaria.

PMID: 16771204 [PubMed - indexed for MEDLINE]

835. Emerg Infect Dis. 2015 Feb;21(2):280-9. doi: 10.3201/eid2102.140291.

Infectious causes of encephalitis and meningoencephalitis in Thailand, 2003-2005.

Olsen SJ, Campbell AP, Supawat K, Liamsuwan S, Chotpitayasunondh T, Laptikulthum S, Viriyavejakul A, Tantirittisak T, Tunlayadechanont S, Visudtibhan A, Vasiknanonte P, Janjindamai S, Boonluksiri P, Rajborirug K, Watanaveeradej V, Khetsuriani N, Dowell SF; Thailand Encephalitis Surveillance Team.

Collaborators: Henchaichon S, Limpakarnjanarat K, Sawanpanyalert P, Pongsuwanna Y, Kijphati R, Dejsirilert S, Thawatsupha P, Anantapreecha S, Pattamadilok S, Chenchittikul M, Wootha W, Ruangsuwan S, Techatuwanan S, Watchaputi A, Angsuwan P, Dhiravibulya K, Lusawat A, Samsen M, Witoonpanich R, Chiemchanya S, Veerapradist K, Ngaothamatasn W, Warachit B, Peeraputhi S, Yodsawat J, Pinjaroen S, Sathirapanya P, Setthawatcharawanich S, Ruengrairattanaroj P, Nabangchang C, Phiboonbanakit D, Bresee J, Lingappa J, Pallansch M, Oberste MS, Erdman D, Bellini WJ, Rota P, Tong S, Schmid DS, Loparev V, Dasch G, Petersen L, Marfin AA, Campbell GL, Powers A, Montgomery S, Fischer M, Laven J, Kosoy O, Panella A, Ellis C, Mayer L, Dull P, Whitney A, Fields B, Talkington D, Thacker L, Maguire J, Ksiazek T, Comer JA, Klimov A, Uyeki T, Lindstrom S, Cox N, Siwek A, Douglass J, Bartley S, Rupprecht CE.

Acute encephalitis is a severe neurologic syndrome. Determining etiology from among ≈100 possible agents is difficult. To identify infectious etiologies of encephalitis in Thailand, we conducted surveillance in 7 hospitals during July 2003-August 2005 and selected patients with acute onset of brain dysfunction with fever or hypothermia and with abnormalities seen on neuroimages or electroencephalograms or with cerebrospinal fluid pleocytosis. Blood and cerebrospinal fluid were tested for >30 pathogens. Among 149 case-patients, median age was 12 (range 0-83) years, 84 (56%) were male, and 15 (10%) died. Etiology was confirmed or probable for 54 (36%) and possible or unknown for 95 (64%). Among confirmed or probable etiologies, the leading pathogens were Japanese encephalitis virus, enteroviruses, and Orientia tsutsugamushi. No samples were positive for chikungunya, Nipah, or West Nile viruses; Bartonella henselae; or malaria parasites. Although a broad range of infectious agents was identified, the etiology of most cases remains unknown.

DOI: 10.3201/eid2102.140291
PMCID: PMC4313633
PMID: 25627940 [PubMed - indexed for MEDLINE]

836. Ann Trop Med Parasitol. 2001 Jun;95(4):395-8.

Serological evidence for the continued presence of human rickettsioses in southern India.

Mathai E(1), Lloyd G, Cherian T, Abraham OC, Cherian AM.

Author information:
(1)Department of Clinical Microbiology, Christian Medical College Hospital, Vellore, Tamil Nadu 632004, India. mathaim@cmcvellore.ac.in

Rickettsiosis is generally believed to have disappeared from many parts of India. However, the serological testing of 37 residents of southern India who presented with fever of unknown aetiology in 1996-1998 confirmed that spotted fever, epidemic/endemic typhus and scrub typhus continue to occur in southern India. The epidemiology and magnitude of the problem need to be evaluated.

DOI: 10.1080/00034980120065804
PMID: 11454249 [PubMed - indexed for MEDLINE]

837. PLoS Negl Trop Dis. 2010 Nov 16;4(11):e888. doi: 10.1371/journal.pntd.0000888.

Arthropod borne disease: the leading cause of fever in pregnancy on the Thai-Burmese border.

McGready R(1), Ashley EA, Wuthiekanun V, Tan SO, Pimanpanarak M, Viladpai-Nguen SJ, Jesadapanpong W, Blacksell SD, Peacock SJ, Paris DH, Day NP, Singhasivanon P, White NJ, Nosten F.

Author information:
(1)Shoklo Malaria Research Unit, Mae Sot, Tak, Thailand. rose@shoklo-unit.com

BACKGROUND: Fever in pregnancy is dangerous for both mother and foetus. In the 1980's malaria was the leading cause of death in pregnant women in refugee camps on the Thai-Burmese border. Artemisinin combination therapy has significantly reduced the incidence of malaria in the population. The remaining causes of fever in pregnancy are not well documented.
METHODOLOGY: Pregnant women attending antenatal care, where weekly screening for malaria is routine, were invited to have a comprehensive clinical and laboratory screen if they had fever. Women were admitted to hospital, treated and followed up weekly until delivery. A convalescent serum was collected on day 21. Delivery outcomes were recorded.
PRINCIPAL FINDINGS: Febrile episodes (n = 438) occurred in 5.0% (409/8,117) of pregnant women attending antenatal clinics from 7-Jan-2004 to 17-May-2006. The main cause was malaria in 55.5% (227/409). A cohort of 203 (49.6% of 409) women had detailed fever investigations and follow up. Arthropod-borne (malaria, rickettsial infections, and dengue) and zoonotic disease (leptospirosis) accounted for nearly half of all febrile illnesses, 47.3% (96/203). Coinfection was observed in 3.9% (8/203) of women, mostly malaria and rickettsia. Pyelonephritis, 19.7% (40/203), was also a common cause of fever. Once malaria, pyelonephritis and acute respiratory illness are excluded by microscopy and/or clinical findings, one-third of the remaining febrile infections will be caused by rickettsia or leptospirosis. Scrub and murine typhus were associated with poor pregnancy outcomes including stillbirth and low birth weight. One woman died (no positive laboratory tests).
CONCLUSION/SIGNIFICANCE: Malaria remains the leading cause of fever in pregnancy on the Thai-Burmese border. Scrub and murine typhus were also important causes of fever associated with poor pregnancy outcomes. Febrile pregnant women on the Thai-Burmese border who do not have malaria, pyelonephritis or respiratory tract infection should be treated with azithromycin, effective for typhus and leptospirosis.

DOI: 10.1371/journal.pntd.0000888
PMCID: PMC2982829
PMID: 21103369 [PubMed - indexed for MEDLINE]

838. Am J Trop Med Hyg. 2003 Apr;68(4):480-4.

Serologic evidence of infection with ehrlichiae and spotted fever group rickettsiae among residents of Gag Island, Indonesia.

Richards AL(1), Ratiwayanto S, Rahardjo E, Kelly DJ, Dasch GA, Fryauff DJ, Bangs

MJ.

Author information:
(1)U.S. Naval Medical Research Unit No. 2, Jakarta, Indonesia.
RichardsA@nmrc.navy.mil

The causative agents of scrub and murine typhus are considered endemic to Indonesia. However, the presence of spotted fever group rickettsiae and ehrlichiae have not been previously described in this country. During an investigation of arthropod-borne diseases on Gag Island, located northwest of the island of New Guinea in eastern Indonesia, the prevalence of antibody to the etiologic agents of monocytic ehrlichiosis, spotted fever rickettsiosis, and scrub and murine typhus were determined. Analysis of 55 blood samples from residents of Gag Island showed seroreactivity to antigen preparations of Ehrlichia chaffeensis (7 of 48, 14.6%), two spotted fever group rickettsiae: Rickettsia rickettsii (5 of 48, 10.4%) and R. conorii (10 of 49, 20.4%), Orientia tsutsugamushi (5 of 53, 9.4%), and R. typhi (1 of 48, 2.1% [by an indirect immunofluorescence assay] and 1 of 50, 2.0% [by an enzyme-linked immunosorbent assay]). These results show serologic evidence of infection with ehrlichiae and spotted fever group rickettsiae for the first time in Indonesia in a location where the prevalence of antibody to O. tsutsugamushi and R. typhi was lower.

PMID: 12875301 [PubMed - indexed for MEDLINE]

839. J La State Med Soc. 2010 May-Jun;162(3):140-5, 147-9.

Endemic mite-transmitted dermatoses and infectious diseases in the South.

Diaz JH(1).

Author information:
(1)School of Public Health, Louisiana State University Health Sciences Center, New Orleans, USA.

Mites are mostly ubiquitous, bothersome pests with few species of medical importance and, of these, most are scabies mites, trombiculid larval mites and animal and plant mites. All patients with scabies and their close household, institutional and sexual contacts should be informed that scabies is a highly transmissible ectoparasitic infestation and that several topical treatments and an effective oral treatment are readily available and highly effective at present. Sexually active patients with scabies should be screened for other sexually transmitted diseases, particularly human immunodeficiency virus (HIV) and human T-cell lymphotrophic virus type1 (HTLV-1) infections. Finally, only the Asian and Eurasian Leptotrombidium species of trombiculid larval mites (or chiggers) can transmit scrub typhus in endemic regions of Asia, Eurasia, and the South Pacific, and only the house-mouse mite can transmit rickettsialpox in both urban and rural dwellings worldwide, including the southern United States.

PMID: 20666166 [PubMed - indexed for MEDLINE]

840. Ann N Y Acad Sci. 2009 May;1166:151-5. doi: 10.1111/j.1749-6632.2009.04530.x.

Rickettsioses in Australia.

Graves S(1), Stenos J.

Author information:
(1)Director, Australian Rickettsial Reference Laboratory, Geelong, Victoria, Australia. Stephen.graves@hnehealth.nsw.gov.au

The rickettsial diseases of Australia are described in their chronological order of discovery. The include epidemic typhus (R. prowazekii); murine typhus (R. typhi) found Australia-wide; scrub typhus (O. tsutsugamushi) only in tropical, northen Australia; Q. fever (C. burnetti) found Australia-wide; Queensland tick typhus (R. australis) along the east coast of Australia; Flinders Island spotted fever (R. honei) in southeast Australia; Variant Flinders Island spotted fever (R. honei, strain "marmionii") in eastern Australia; Rickettsia felis, Western Australia; eight new RFG rickettsiae from ticks (of unknown pathogenicity); and two nonhuman pathogens in A. platys (dogs) and A. marginale (cattle).

DOI: 10.1111/j.1749-6632.2009.04530.x
PMID: 19538275 [PubMed - indexed for MEDLINE]

841. P N G Med J. 2003 Mar-Jun;46(1-2):53-62.

Survey of rickettsial antibodies at two local sites and review of rickettsiosis in Papua New Guinea.

Kende M(1), Graves S.

Author information:
(1)The Geelong Hospital, Victoria, Australia and Port Moresby General Hospital, Papua New Guinea.

The status of rickettsial infection in Papua New Guinea (PNG) is unknown although several reports of typhus-like illnesses infecting predominantly white settlers and the Allied troops during World War 2 were published between 1930 and 1945. We performed a serological survey for evidence of rickettsial infection by measuring rickettsia-specific antibody levels in the blood of 191 non-randomly selected Papua New Guineans living in Port Moresby (n=93) and in the highland villages of Samberigi (n=98). Antibodies were measured by a microimmunofluorescence method using a panel of rickettsial antigens of a number of species and strains. In addition, we have reviewed the current status of rickettsiosis in PNG. Overall, we were able to demonstrate significant titres of antibodies to two groups of rickettsiae, the scrub typhus group (STG) and the spotted fever group (SFG). All positive individuals (7/191) were residents of Port Moresby. None from the highlands showed any significant levels of antibodies to rickettsiae. The strains detected within each group were Gilliam and Karp for STG and, for SFG, Rickettsia honei, R. conorii, R. sibirica, R. rickettsii, R. australis and R. akari. No significant antibody titres to typhus group infection were detected in either Port Moresby or highland volunteers. These findings were not surprising given previous reports of typhus-like illnesses and favourable environmental characteristics for rickettsiae in some parts of PNG. Until a definite status of

this disease is known, we suggest that rickettsial infection be included as a differential diagnosis for patients presenting with acute febrile illness in Port Moresby and surrounding areas.

PMID: 16450784 [PubMed - indexed for MEDLINE]

842. Bull Soc Pathol Exot. 2000 Jan;93(5):348-52.

[The state of vector-borne diseases in Indonesia].

[Article in French]

Rodhain F(1).

Author information:
(1)Unité d'écologie des systèmes vectoriels, Institut Pasteur, Paris, France.

From epidemiological point of view, Indonesia is an extremely interesting area owing its insular structure and ecological, anthropological, cultural and economical diversity. As everywhere, vector-borne diseases are the result of complex and variable epidemiological systems, subject both to biogeographical rules and human activity. Two main arboviroses are present in Indonesia: dengue and Japanese encephalitis. Dengue appears as an endemoepidemic disease and is mostly circumscribed to urban areas. Haemorrhagic cases were first observed in 1968; since then, the incidence has been constantly increasing and the disease is now one of the principal causes of child lethality. Japanese encephalitis is a rural endemic disease transmitted by rice-field mosquitoes; its incidence remains relatively low since pigs, which are usual link-hosts for the virus, are uncommon in this mainly Muslem country. Human clinical cases are recorded from non-Muslem islands such as Bali or Irian Jaya which raises the question of immunisation for travellers. Recently, Japanese encephalitis was observed on east of the Wallace line which had been considered as the eastern cut-off line. Malaria is common throughout the country, Plasmodium vivax being the most frequent species. Some of the Anopheline vectors are related to brackish water as are coastal species; others have been favoured by rice growing. Several species bite and rest outdoors, rendering control measures complex. Moreover, chloroquine resistance is increasing in both P. falciparum and P. vivax. All three filaria species responsible for human lymphatic filariasis exist in Indonesia. Bancroft filariasis is present in rather limited foci on most of the islands; malayan filariasis is very prevalent on many islands, mostly in coastal areas, and Timor filariasis exist only on a few small islands. These parasitic diseases are cumulative and do not practically endanger the health of travellers. In the past, plague was common on Java island, but today, human cases are very rare. Scrub typhus is prevalent everywhere, as is murine typhus, being very frequent in harbour cities and one of the main causes of hospitalisation for febrile syndromes.. On the whole, the situation of several of these diseases has been worsening in Indonesia for about thirty years. Although epidemiological situations constantly evolve, two recent occurrences should be paid particular attention: -transmigration which is now a national priority and greatly facilitates the spread of many pathogens, arboviroses or chloroquine-resistant plasmodia, but also of rats, mosquitoes, etc. -deforestation due either to land-farming by Javanese transmigrants or to sudden climatic changes such as El

Niño in 1997. Such deep ecological transformations may have considerable and unforeseeable consequences on the epidemiology of vector-borne diseases in Indonesia.

PMID: 11775322 [PubMed - indexed for MEDLINE]

843. Eur J Epidemiol. 1991 May;7(3):282-6.

Control of rickettsial diseases.

Kazár J(1), Brezina R.

Author information:
(1)Institute of Virology, Slovak Academy of Sciences, Bratislava, C.S.F.R.

Prevention of rickettsial infections is aimed at individual control and epidemic measures (especially in epidemic typhus), vector and rodent control, milk pasteurization (in Q fever), chemoprophylaxis and immunoprophylaxis. In vector and rodent control, the main obstacle is the rise in resistance to insecticides and rodenticides. For this reason in vector control, apart from insecticides, enhancement of the natural immunity acquired by animals in response to tick infestation and vaccination with concealed tick antigens as well as the use of hormones, chemosterilants and genetic manipulation can also be considered. For short-term high-risk exposure, doxycycline may be an effective prophylaxis of illness but may not prevent infection with scrub typhus or spotted fever group rickettsiae. At present, for specific prevention by vaccination, only Q fever vaccines are available for common use. However, development of subunit vaccines, namely immunogenic rickettsial proteins, cloned and expressed in Escherichia coli, seems to be very promising.

PMID: 1884780 [PubMed - indexed for MEDLINE]

844. J Exp Med. 1946 Jun 30;84(1):37-50.

A RICKETTSIAL INFECTION IN CANADIAN VOLES.

Baker JA(1).

Author information:
(1)War Disease Control Station, Grosse Isle, Province of Quebec, Canada.

From apparently normal voles captured on Grosse Isle, Province of Quebec, Canada, an infective agent has been grown in embryonated eggs, and by inoculation an inapparent infection was established in voles, mice, guinea pigs, hamsters, and rats. No growth of the agent was obtained in the absence of living cells, and the manner of its development in the yolk sac of embryonated eggs, as well as morphological, epidemiological, and pathogenic features, indicates a rickettsial nature. The inability to transmit infection by either cage or intrauterine contact points to a vector, and mites are shown to have a probable part in the epidemiology. Mice infected with the vole agent resist lethal doses of the Karp

strain of scrub typhus, and certain epidemiological, morphological, and immunological features support the relationship indicated by the mouse tests. It is therefore concluded that voles on this island have an inapparent infection due to a rickettsia that may be related to the rickettsia of scrub typhus.

PMCID: PMC2135639
PMID: 19871552 [PubMed]

845. Emerg Infect Dis. 2007 Apr;13(4):566-73.

Flinders Island spotted fever rickettsioses caused by "marmionii" strain of Rickettsia honei, Eastern Australia.

Unsworth NB(1), Stenos J, Graves SR, Faa AG, Cox GE, Dyer JR, Boutlis CS, Lane AM, Shaw MD, Robson J, Nissen MD.

Author information:
(1)Australian Rickettsial Reference Laboratory, Geelong, Victoria, Australia.

Australia has 4 rickettsial diseases: murine typhus, Queensland tick typhus, Flinders Island spotted fever, and scrub typhus. We describe 7 cases of a rickettsiosis with an acute onset and symptoms of fever (100%), headache (71%), arthralgia (43%), myalgia (43%), cough (43%), maculopapular/petechial rash (43%), nausea (29%), pharyngitis (29%), lymphadenopathy (29%), and eschar (29%). Cases were most prevalent in autumn and from eastern Australia, including Queensland, Tasmania, and South Australia. One patient had a history of tick bite (Haemaphysalis novaeguineae). An isolate shared 99.2%, 99.8%, 99.8%, 99.9%, and 100% homology with the 17 kDa, ompA, gltA, 16S rRNA, and Sca4 genes, respectively, of Rickettsia honei. This Australian rickettsiosis has similar symptoms to Flinders Island spotted fever, and the strain is genetically related to R. honei. It has been designated the "marmionii" strain of R. honei, in honor of Australian physician and scientist Barrie Marmion.

DOI: 10.3201/eid1304.050087
PMCID: PMC2725950
PMID: 17553271 [PubMed - indexed for MEDLINE]

846. BMC Public Health. 2009 Mar 31;9:93. doi: 10.1186/1471-2458-9-93.

Timeliness of national notifiable diseases surveillance system in Korea: a cross-sectional study.

Yoo HS(1), Park O, Park HK, Lee EG, Jeong EK, Lee JK, Cho SI.

Author information:
(1)Division of Infectious Disease Surveillance, Korea Centers for Disease Control and Prevention, Seoul, Republic of Korea. hsyoo@cdc.go.kr

BACKGROUND: With the increase of international travels, infectious disease control is gaining a greater importance across regional borders. Adequate surveillance system function is crucial to prevent a global spread of infectious

disease at the earliest stage. There have been limited reports on the
characteristics of infectious disease surveillance in Asia. The authors studied
the timeliness of the Korean National Notifiable Disease Surveillance System with
regard to major notifiable diseases from 2001 to 2006.
METHODS: Six notifiable infectious diseases reported relatively frequently were
included in this study. Five diseases were selected by the criteria of reported
cases > 100 per year: typhoid fever, shigellosis, mumps, scrub typhus, and
hemorrhagic fever with renal syndrome. In addition, dengue fever was also
included to represent an emerging disease, despite its low number of cases. The
diseases were compared for the proportion notified within the recommended time
limits, median time lags, and for the cumulative distribution of time lags at
each surveillance step between symptom onset and date of notification to the
Korea Centers for Disease Control and Prevention (KCDC).
RESULTS: The proportion of cases reported in time was lower for disease groups
with a recommended time limit of 1 day compared with 7 days (60%-70% vs. > 80%).
The median time from disease onset to notification to KCDC ranged between 6 and
20 days. The median time from onset to registration at the local level ranged
between 2 and 15 days. Distribution of time lags showed that main delays arose in
the time from onset to diagnosis. There were variations in timeliness by disease
categories and surveillance steps.
CONCLUSION: Time from disease onset to diagnosis generally contributed most to
the delay in reporting. It is needed to promote public education and to improve
clinical guidelines. Rapid reporting by doctors should be encouraged, and
unification of recommended reporting time limit can be helpful. Our study also
demonstrates the utility of the overall assessment of time-lag distributions for
disease-specific strategies to improve surveillance.

DOI: 10.1186/1471-2458-9-93
PMCID: PMC2676269
PMID: 19331696 [PubMed - indexed for MEDLINE]

847. Emerg Infect Dis. 2003 May;9(5):592-5.

Emerging rickettsioses of the Thai-Myanmar border.

Parola P(1), Miller RS, McDaniel P, Telford SR 3rd, Rolain JM, Wongsrichanalai C,
Raoult D.

Author information:
(1)Armed Forces Research Institute of Medical Sciences (AFRIMS), Bangkok,
Thailand.

To investigate the presence of rickettsioses in rural residents of the central
Thai-Myanmar border, we tested the blood of 46 patients with fever. Four patients
had murine typhus, three patients had scrub typhus, and eight patients had
spotted fever group rickettsioses, including the first case of Rickettsia felis
infection reported in Asia.

PMCID: PMC2972759
PMID: 12737744 [PubMed - indexed for MEDLINE]

848. J Microbiol Immunol Infect. 2008 Oct;41(5):362-8.

Clinical prediction of endemic rickettsioses in northern Taiwan--relevance of peripheral blood atypical lymphocytes.

Chen NY(1), Huang PY, Leu HS, Chiang PC, Huang CT.

Author information:
(1)Division of Infectious Diseases, Department of Internal Medicine, Chang Gung Memorial Hospital, Chang Gung University College of Medicine, Taoyuan, Taiwan.

BACKGROUND AND PURPOSE: Several rickettsioses are endemic in Taiwan. They are under-reported not only because of ignorance but also due to difficulty in recognition caused by their nonspecific manifestations, which overlap with other acute febrile illnesses. We conducted a retrospective study to delineate distinctive clinical features of rickettsiosis, in order to develop a system for differential diagnosis of rickettsiosis.
METHODS: Patients admitted to Chang Gung Memorial Hospital Linkou Medical Center, Taoyuan, Taiwan, with suspected rickettsiosis during the period from January 2004 to May 2006 were included. Clinical suspicion was based on the presence of acute fever with eschar formation, relevant contact history, poor response to broad-spectrum empiric antibacterial therapy, unexplained thrombocytopenia, leukopenia, or abnormal liver biochemistry, or unexplained major organ involvement. Serum samples were sent to the Centers for Disease Control, Taiwan, for serologic diagnosis of the 3 rickettsioses endemic to Taiwan - scrub typhus (Tsutsugamushi's disease), murine typhus (endemic typhus) and Q fever. Serologically confirmed and excluded cases were compared for signs and symptoms, risk factors, laboratory findings and response to treatment.
RESULTS: Among 138 suspected cases, 88 were excluded from the study because of incomplete serological tests or insufficient information, 28 were confirmed to have one of the 3 rickettsioses and 22 were negative for all of them. Distinct features among confirmed cases, compared to controls, were eschar formation, relevant contact history, and presence of atypical lymphocytes in peripheral blood. Normal or low leukocyte count, thrombocytopenia and relative bradycardia were not significant in predicting diagnosis. We propose a predictive system for tentative diagnosis of rickettsiosis based on relevant clinical attributes. This system has a positive predictive value of 80% and a negative predictive value of 100%.
CONCLUSIONS: The predictive scoring system may allow institution of appropriate treatment for rickettsiosis in a more timely manner. However, a low probability of diagnosis should prompt vigorous search for other etiologies.

PMID: 19122916 [PubMed - indexed for MEDLINE]

849. Clin Dermatol. 2006 May-Jun;24(3):191-200.

Tropical rickettsioses.

Parola P(1), Raoult D.

Author information:
(1)Unité des Rickettsies CNRS UMR 6020, IFR48, WHO Collaborative Center for

Rickettsial Reference and Research, Medicine School of Marseille, France.

In recent years, a bewildering array of emerging rickettsial pathogens have been described throughout the world, including in the tropics. Here we present an updated overview of scrub typhus, murine typhus, and epidemic typhus. We also present an update on the emerging spotted fever group rickettsioses described in the tropics through 2005, focusing on epidemiologic and clinical data and management.

DOI: 10.1016/j.clindermatol.2005.11.007
PMID: 16714200 [PubMed - indexed for MEDLINE]

850. J Assoc Physicians India. 2010 Jun;58:390-1.

Leptospirosis presenting as acute respiratory distress syndrome (ARDS) in sub-Himalayan region.

Chauhan V(1), Mahesh DM, Panda P, Mokta J, Thakur S.

Author information:
(1)Department of Medicine, Indira Gandhi Medical College, Shimla 171 001, Himachal Pradesh.

Indira Gandhi Medical College, Shimla receives referred patients of pyrexia with multi-organ dysfunction during the monsoon season from all over the state of Himachal Pradesh. Most common etiologies of pyrexia are enteric fever, scrub typhus, malaria, viral, tubercular, and some patients of dengue fever from adjoining states. Leptospirosis has not yet been reported in sub-Himalayan state of Himachal Pradesh, India. We present here a case of leptospirosis presenting as ARDS, proven on IgM Elisa and confirmed by PCR. Leptospirosis is a new etiology in this region for patients presenting with pyrexia and ARDS.

PMID: 21125784 [PubMed - indexed for MEDLINE]

851. Med Vet Entomol. 1991 Jan;5(1):1-7.

Ectoparasites of commensal rodents in Sulawesi Utara, Indonesia, with notes on species of medical importance.

Durden LA(1), Page BF.

Author information:
(1)Department of Entomology, Smithsonian Institution, Washington, D.C. 20560.

Ectoparasite records are presented for four species of commensal murid rodents (Rattus rattus palelae Miller & Hollister, R. argentiventer (Robinson & Kloss), R. exulans (Peale) and Mus musculus castaneus Waterhouse) in Sulawesi Utara, with particular reference to the potential for these arthropods to bite and transmit pathogens to humans. The flea, Xenopsylla cheopis (Rothschild), was most common on R.r. palelae and is capable of transmitting plague and other pathogens to humans although no current foci for these diseases are known in Sulawesi.

Hoplopleura pacifica Ewing and Polyplax spinulosa (Burmeister) sucking lice parasitized all three Rattus species although H. pacifica was mainly associated with R. exulans and P. spinulosa with R.r. palelae. These lice do not bite humans but may be intramurid vectors of murine typhus and other zoonoses. The mites Laelaps echidnina Berlese and L. nuttalli Hirst were both collected; the latter was recorded from all four murid species, mainly R. exulans. The mite Ornithonyssus bacoti Hirst was rare. Only one chigger mite, Walchiella oudemansi (Walch), was retrieved from murids (from R. exulans) and a single Leptotrombidium deliense (Walch) chigger was taken from a human subject. Although L. deliense is a significant vector of scrub typhus, a disease known from Sulawesi, the L. deliense-R. argentiventer relationship frequently noted in the ecology of this rickettsial disease, was not evident in this survey. Other ectoparasites collected from murids were the ticks, Ixodes granulatus Supino (first record for Sulawesi), Haemaphysalis sp. and Dermacentor sp., the mites Myocoptes musculinus (Koch) and Listrophoroides cucullatus (Trouessart), acarids and a uropodid.(ABSTRACT TRUNCATED AT 250 WORDS)

PMID: 1768888 [PubMed - indexed for MEDLINE]

852. Southeast Asian J Trop Med Public Health. 2004 Sep;35(3):657-63.

A study of'febrile illnesses on the Thai-Myanmar border: predictive factors of rickettsioses.

PicKard AL(1), McDaniel P, Miller RS, Uthaimongkol N, Buathong N, Murray CK, Telford SR 3rd, Parola P, Wongsrichanalai C.

Author information:
(1)Armed Forces Research Institute of Medical Sciences, Bangkok, Thailand.

We have performed a case-control analysis to determine the significance of clinical, laboratory and epidemiological features as predictive factors of rickettsioses among patients in Sangkhla Buri, Thailand (Thai-Myanmar border). Fifteen serologically-confirmed rickettsiosis patients including Spotted Fever Group (SFG) rickettsioses, scrub typhus, and murine typhus were classified as 'cases'; one hundred and sixty-three acutely febrile patients presenting to the same hospital during the same time period, who had no serological evidence of acute rickettsiosis, were classified as 'controls'. Patients' report of rash/arthropod bite [Odds ratio (OR) 22.90, 95% CI (confidence interval) 6.23, 84.13] and history of jungle trips (OR 5.30, 95% CI 1.69-16.62) were significant risk factors. Elevated ALT (OR 3.04, 95% CI 1.04, 8.88) and depressed platelet count (OR 3.38, 95% CI 1.13, 10.10) were also useful differentiating markers of rickettsioses in this population. Definitive diagnosis of rickettsioses is difficult without specialized diagnostic capabilities that are rarely available in remote areas such as Sangkhla Buri, where other acute febrile illnesses with similar presentation are commonly found. The relative importance of predictive factors presented here may provide clinicians with some useful guidance in distinguishing rickettsioses from other acute febrile illnesses. Timely administration of empiric treatment in highly suspicious cases can deter potential morbidity from these arthropod-borne infections.

PMID: 15689083 [PubMed - indexed for MEDLINE]

853. Am J Trop Med Hyg. 1994 Jun;50(6):682-6.

First cases of spotted fever group rickettsiosis in Thailand.

Sirisanthana T(1), Pinyopornpanit V, Sirisanthana V, Strickman D, Kelly DJ, Dasch GA.

Author information:
(1)Faculty of Medicine, Chiang Mai University, Thailand.

The first three cases of spotted fever group rickettsiosis from Thailand are reported. The patients presented with fever, headache, lymphadenopathy, and petechial maculopapular rash. One patient also had an eschar and overt evidence of confusion. An indirect fluorescent antibody test, an indirect immunoperoxidase test, and an enzyme-linked immunosorbent assay demonstrated a broad, strong reactions of the sera of the patients with spotted fever group rickettsia antigens of many species, but not with antigens of typhus or scrub typhus rickettsiae. All three patients responded to treatment with a single dose of doxycycline.

PMID: 8024059 [PubMed - indexed for MEDLINE]

854. Southeast Asian J Trop Med Public Health. 2003 Mar;34(1):175-8.

The etiology of acute pyrexia of unknown origin in children after a flood.

Pradutkanchana J(1), Pradutkanchana S, Kemapanmanus M, Wuthipum N, Silpapojakul K.

Author information:
(1)Department of Pathology, Faculty of Medicine, Prince of Songkla University, Hat Yai, Songkhla, Thailand. pjintana@ratree.psu.ac.th

Acute pyrexia of unknown origin (PUO) is a major public health problem in Thailand. We studied the etiology of 180 cases of acute PUO in children after a sudden severe flood in Hat Yai city in 2000. Dengue infection and leptospirosis accounted for more than half of the total cases. Dengue hemorrhagic fever was the most common (29.4%) followed by leptospirosis (27.2%) and scrub typhus infection (1.1%). Five serovars of leptospires were involved in this study. Leptospira interrogans bataviae was the most common (86.5%). Acute serum antibody testing could detect only 52.8% and 40.8% of dengue and leptospirosis cases, respectively. This study showed both should be included in the presumptive diagnosis of acute PUO in patients after flooding.

PMID: 12971532 [PubMed - indexed for MEDLINE]

855. J Clin Microbiol. 1997 Jul;35(7):1715-21.

Production of monoclonal antibodies against Rickettsia massiliae and their use in antigenic and epidemiological studies.

Xu W(1), Raoult D.

Author information:
(1)Unité des Rickettsies, CNRS UPRES-A 6020, Faculté de Médecine, Université de la Mediterranée, Marseille, France.

Rickettsiae are gram-negative, obligate intracellular bacteria which have historically been divided into three groups: the typhus group, the scrub typhus group, and the spotted fever group (SFG). Recently, several new SFG rickettsiae have been characterized, and most of these species are associated with ticks and have, as yet, no known pathogenicity toward humans. Rickettsia massiliae, which is widely distributed in Europe and Africa, is one such rickettsia. In order to investigate the antigenic relationships between R. massiliae and other rickettsial species and to develop a more convenient methodology for identifying R. massiliae, we produced monoclonal antibodies against the type strain (Mtu1T) of R. massiliae by fusing immunized splenocytes with SP2/0-Ag14 myeloma cells. A panel of 16 representatives were selected from the 163 positive hybridomas identified on initial screening, and their secreted monoclonal antibodies were further characterized. The reactivities of these 16 monoclonal antibodies with a large panel of rickettsial species were assessed by the microimmunofluorescence assay. All species of the SFG rickettsiae reacted with the monoclonal antibodies directed against epitopes on lipopolysaccharide, which is the common antigen among the SFG rickettsiae. Some closely related species of the SFG, such as Bar29, "R. aeschlimanni," and R. rhipicephali, showed strong cross-reactivities with the monoclonal antibodies directed against epitopes on the two major high-molecular-mass heat-labile proteins (106 and 120 kDa). In addition, species-specific monoclonal antibodies demonstrated that R. massiliae is antigenically different from other rickettsial species. Moreover, these species-specific monoclonal antibodies were successfully used for identifying R. massiliae in the ticks collected from southern France, and are therefore potentially useful tools in the identification and investigation of R. massiliae in ticks in large-scale field work.

PMCID: PMC229828
PMID: 9196180 [PubMed - indexed for MEDLINE]

856. J Microbiol Immunol Infect. 1998 Jun;31(2):73-83.

Current outlook of infectious diseases in Taiwan.

Ho M(1).

Author information:
(1)Division of Clinical Research, National Health Research Institutes, Taipei, Taiwan, ROC.

The "emerging" infectious diseases have received global attention. Taiwan is a country which is going through the process of becoming "developed" from being

"developing". If we compare five leading causes of death in 1952 and in 1993, three were infectious diseases in 1952 and there was none in 1993. And yet today, infectious diseases remain a major problem in this country as well in every country in the world, whether developing or developed. Some of the problems Taiwan faces are old problems with old faces. They have never been adequately solved because the societal and environmental sanitary infrastructure does not ensure proper sewage disposal, safe potable water and freedom from dangerous vectors. Examples are the diarrheal diseases, parasitic diseases, scrub typhus and Japanese encephalitis. Some of the Taiwan's problems are caused by old agents which present a new face. Mortality from tuberculosis took a dramatic and gratifying plunge in the last fifty years. Yet tuberculosis is ever present and a constant public health threat. Dengue has become a problem again because of a world breakdown in the control of the mosquito, Aedes egypti, and it is partly contributed to by increased urbanization and world travel. The problem of antibiotic resistant bacteria causing hospital acquired and community acquired infections is probably the most serious "new" problem. The most important cause is excessive and indiscriminate use of antibiotics in the community and in hospitals. We propose the establishment of "Bacterial Infections Reference Laboratory" at the National Health Research Institutes to be a national facility to study the epidemiology and control of antibiotic resistance. All infectious diseases require a rigorous system of surveillance, and precise etiological diagnosis before they can be treated or prevented. This should be kept clearly in mind when one considers the changing role of the infectious disease physician in Taiwan in the face of unsolved disease problems and a new health care system. There is inadequate attention to precise microbiological definition of most infectious diseases in Taiwan. The community of infectious disease specialists may well redirect its attention to improving the competence and utilization of microbiological laboratory diagnosis.

PMID: 10596983 [PubMed - indexed for MEDLINE]

857. Zhonghua Liu Xing Bing Xue Za Zhi. 2011 Jan;32(1):47-50.

[An outbreak of endemic typhus in Baoshan city, Yunnan province].

[Article in Chinese]

Ya HX(1), Zhang HL, Zhou JH, Duan HX, Zhao XZ, Sun GJ, Zheng WB, Huang WL.

Author information:
(1)Yunnan Institute of Endemic Disease Control and Prevention, Dali 671000, China. yahongxiang@163.com

OBJECTIVE: To understand the epidemiologic characteristics of endemic typhus in Baoshan city.
METHODS: Epidemiological data were collected and characteristics were analyzed. IgG antibody (Ab) of Rickettsia mooseri and Orientia tsutsugamushi in serum of patients were tested using both Weil-Felix and IFA method. The Rickettsia mooseri gltA gene, Rickettsia prowazekii gltA gene, Orientia tsutsugamushi 56 kDa protein gene, SFGR ompA gene, Ehrlichia sp. 16S rRNA gene and Anaplasma sp. 16S rRNA gene in spleen of mice were examined by PCR.
RESULTS: Fifty-eight endemic typhus cases were found in Longyang district of

Baoshan city, during July to August, 2009. Among them, 48 cases were confirmed by clinical diagnosis and 10 cases by laboratory tests. The Ab of Orientia tsutsugamushi Karp serotype was detected in 3 cases from laboratory diagnosis. The spleen samples from 85 Rattus flavipectus were tested using PCR. Of them, 3 samples for Rickettsia mooseri gltA gene showed positive (positive rate was 3.5%), and the homology of 3 Rickettsia mooseri and Rickettsia mooseri Wilmington strain (GenBank U59714.1) was 100% through comparing gene sequence. The results of PCR for detecting Rickettsia prowazekii, Orientia tsutsugamushi, SFGR, Anaplasma sp. and Ehrlichia. sp were all negative.
CONCLUSION: The outbreak of endemic typhus was confirmed in Longyang district of Baoshan city through epidemiological data, clinical diagnosis and laboratory tests. Rickettsia mooseri DNA was detected in the dominant Rattus flavipectus, suggesting that endemic typhus did exist in the local areas.

PMID: 21518541 [PubMed - indexed for MEDLINE]

858. Prog Med Virol. 1989;36:62-102.

Hemorrhagic fever with renal syndrome.

Lee HW, van der Groen G.

Hantaviruses, the causative agents of HFRS, have become more widely recognized. Epidemiologic evidence indicates that these pathogens are distributed worldwide. People who come into close contact with infected rodents in urban, rural and laboratory environments are at particular risk. Transmission to man occurs mainly via the respiratory tract. The epidemiology of the hantaviruses is intimately linked to the ecology of their principal vertebrate hosts. Four distinct viruses are now recognized within the hantavirus genus and that number is likely to increase to six very soon; however, further investigations are necessary. Much more work is still needed before we fully understand the wide spectrum of clinical signs and symptoms of HFRS as well as the pathogenicity of the different viruses in the hantavirus genus of the Bunyaviridae family. HFRS is difficult to diagnose on clinical grounds alone and serological evidence is often needed. A fourfold rise in IgG antibody titer in a 1-week interval, and the presence of the IgM type of antibodies against hantaviruses are good evidence for an acute hantavirus infection. Physicians should be alert for HFRS each time they deal with patients with acute febrile flu-like illness, renal failure of unknown origin and sometimes hepatic dysfunction. Especially the mild form of HFRS is difficult to diagnose. Acute onset, headache, fever, increased serum creatinine, proteinuria and polyuria are signs and symptoms compatible with a mild form of HFRS. Differential diagnosis should be considered for the following diseases in the endemic areas of HFRS: acute renal failure, hemorrhagic scarlet fever, acute abdomen, leptospirosis, scrub typhus, murine typhus, spotted fevers, non-A, non-B hepatitis, Colorado tick fever, septicemia, dengue, heartstroke and DIC. Treatment of HFRS is mainly supportive. Recently, however, treatment of HFRS patients with ribavirin in China and Korea, within 7 days after onset of fever, resulted in a reduced mortality as well as shortened course of illness.

PMID: 2573914 [PubMed - indexed for MEDLINE]

859. P N G Med J. 2009 Mar-Jun;52(1-2):54-68.

Malaria survey and malaria control detachments in the South-West Pacific Area in World War 2.

Crocker DW(1).

Author information:
(1)Skidmore College, New York, United States of America. dcrocke1@nycap.rr.com

Malaria among troops in the South-West Pacific Area (SWPA) in World War 2 affected the military effort to the degree that special units were formed to combat it. These malaria survey detachments (MSDs) and malaria control detachments (MCDs) were self-contained and so could move quickly to wherever their services were needed. In SWPA by 25 September 1944 there were 32 MSDs and 65 MCDs. Tables of organization called for 11 enlisted men in MSDs and MCDs, two officers in MSDs and one in MCDs. Detachments served throughout the SWPA. Detailed records of the 31st MSD show that in addition to antimalarial efforts it worked at control of scrub typhus, dengue and venereal disease, at reduction of rat populations and in experimental work involving DDT and schistosomiasis. Specific locations of the 31st MSD were New Guinea (3 sites), Morotai, Leyte, Mindoro, Okinawa and Japan. The detachment served overseas for 21 months. Experience in combating malaria in SWPA in World War 2 points to the need for better and continuous training of both medical and line officers in malaria prevention and control.

PMID: 21125991 [PubMed - indexed for MEDLINE]

860. Trop Biomed. 2005 Dec;22(2):243-7.

A preliminary survey of ectoparasites of small mammals in Kuala Selangor Nature Park.

Chuluun B(1), Mariana A, Ho T, Mohd Kulaimi B.

Author information:
(1)School of Veterinary Science & Biotechnology, Zaisan 210153, Ulaanbaatar, Mongolia.

Trapping of small mammals was conducted at 5 study sites in Kuala Selangor Nature Park (KSNP) from 20-24 June 2005. A total of 11 animals comprising 2 species of rodents, Maxomys whiteheadi and Rattus exulans were caught from 3 sites, i.e from an area of mixed secondary forest and mangrove swamp; an area of mangrove swamp, and from an area of lalang fringing mangrove swamp. From these animals, the following 7 species of ectoparasites were found: Laelaps echidninus, Laelaps nuttalli, Ascoschoengastia indica, Leptotrombidium deliense, Hoplopleura pectinata, Hoplopleura pacifica and Polyplax spinulosa. One of the ectoparasites found, L. deliense is a known vector of scrub typhus and thus may pose potential health risks to visitors to KSNP.

PMID: 16883294 [PubMed - indexed for MEDLINE]

861. PLoS Negl Trop Dis. 2007 Dec 26;1(3):e111.

A comparative study of leptospirosis and dengue in Thai children.

Libraty DH(1), Myint KS, Murray CK, Gibbons RV, Mammen MP, Endy TP, Li W, Vaughn DW, Nisalak A, Kalayanarooj S, Hospenthal DR, Green S, Rothman AL, Ennis FA.

Author information:
(1)Center for Infectious Disease and Vaccine Research, University of Massachusetts Medical School, Worcester, Massachusetts, USA. daniel.libraty@umassmed.edu

BACKGROUND: Leptospirosis is an emerging zoonosis that is often under-recognized in children and commonly confused with dengue in tropical settings. An enhanced ability to distinguish leptospirosis from dengue in children would guide clinicians and public health personnel in the appropriate use of limited healthcare resources.
METHODOLOGY/PRINCIPAL FINDINGS: We conducted a prospective, hospital-based, study of children with acute febrile illnesses and dengue in Thailand. Among the children without dengue, we identified those with leptospirosis using anti-leptospira IgM and microscopic agglutination titers in paired acute and convalescent blood samples. We then performed a case-control comparison of symptoms, signs, and clinical laboratory values between children with leptospirosis and dengue. In a semi-rural region of Thailand, leptospirosis accounted for 19% of the non-dengue acute febrile illnesses among children presenting during the rainy season. None of the children with leptospirosis were correctly diagnosed at the time of hospital discharge, and one third (33%) were erroneously diagnosed as dengue or scrub typhus. A predictive model to distinguish pediatric leptospirosis from dengue was generated using three variables: the absolute neutrophil count, plasma albumin, and aspartate aminotransferase levels in the first 72 hours of illness.
CONCLUSIONS/SIGNIFICANCE: Unrecognized leptospirosis can be a significant cause of "dengue-like" febrile illness in children. Increased awareness of pediatric leptospirosis, and an enhanced ability to discriminate between leptospirosis and dengue early in illness, will help guide the appropriate use of healthcare resources in often resource-limited settings.

DOI: 10.1371/journal.pntd.0000111
PMCID: PMC2154391
PMID: 18160980 [PubMed - indexed for MEDLINE]

862. Scand J Infect Dis. 2004;36(10):743-51.

Clinical deterioration in community acquired infections associated with lymphocyte upsurge in immunocompetent hosts.

Cheng VC(1), Wu AK, Hung IF, Tang BS, Lee RA, Lau SK, Woo PC, Yuen KY.

Author information:
(1)Division of Infectious Diseases, Centre of Infection Queen Mary Hospital, The

University of Hong Kong, Hong Kong Special Administrative Region, China.

Clinical deterioration during the course of community-acquired infections can occur as a result of an exaggerated immune response of the host towards the inciting pathogens, leading to immune-mediated tissue damage. Whether a surge in the peripheral lymphocyte count can be used as a surrogate marker indicating the onset of immunopathological tissue damage is not known. In this study, we report the clinical presentations and outcomes of a cohort of immunocompetent patients with non-tuberculous community acquired infections who experienced clinical deterioration during hospital stay (n=85). 12 (14.1%) patients had a surge in lymphocyte count preceding their clinical deteriorations, and their diagnoses included viral pneumonitis , viral encephalitis , scrub typhus , leptospirosis , brucellosis , and dengue haemorrhagic fever . The clinical manifestations during deterioration ranged from interstitial pneumonitis , airway obstruction , CNS disturbances , and systemic capillary leak syndrome , all of which were thought to represent immunopathological tissue damages. When compared with patients without lymphocyte surge, these patients were more likely to be infected with fastidious/viral pathogens (0 vs 12; p<0.05), in addition to having lower mean baseline lymphocyte counts (403+/-181 vs 1143+/-686 cells/microl; p<0.05). We postulate that the peripheral lymphocyte count may be a useful surrogate marker indicating the presence of immunopathological damage during clinical deterioration in certain infectious diseases.

DOI: 10.1080/00365540410022602
PMID: 15513401 [PubMed - indexed for MEDLINE]

863. Am J Trop Med Hyg. 2012 Feb;86(2):246-53. doi: 10.4269/ajtmh.2012.11-0409.

Infectious etiologies of acute febrile illness among patients seeking health care in south-central Cambodia.

Kasper MR(1), Blair PJ, Touch S, Sokhal B, Yasuda CY, Williams M, Richards AL, Burgess TH, Wierzba TF, Putnam SD.

Author information:
(1)United States Naval Medical Research Unit 2, Jakarta, Indonesia.
matthew.kasper@med.navy.mil

The agents of human febrile illness can vary by region and country suggesting that diagnosis, treatment, and control programs need to be based on a methodical evaluation of area-specific etiologies. From December 2006 to December 2009, 9,997 individuals presenting with acute febrile illness at nine health care clinics in south-central Cambodia were enrolled in a study to elucidate the etiologies. Upon enrollment, respiratory specimens, whole blood, and serum were collected. Testing was performed for viral, bacterial, and parasitic pathogens. Etiologies were identified in 38.0% of patients. Influenza was the most frequent pathogen, followed by dengue, malaria, and bacterial pathogens isolated from blood culture. In addition, 3.5% of enrolled patients were infected with more than one pathogen. Our data provide the first systematic assessment of the etiologies of acute febrile illness in south-central Cambodia. Data from syndromic-based surveillance studies can help guide public health responses in developing nations.

DOI: 10.4269/ajtmh.2012.11-0409
PMCID: PMC3269275
PMID: 22302857 [PubMed - indexed for MEDLINE]

864. Trans R Soc Trop Med Hyg. 2008 Apr;102(4):312-3. doi:
10.1016/j.trstmh.2008.01.006. Epub 2008 Mar 5.

Infections in travellers arriving from Australia.

McBride WJ(1).

Author information:
(1)Cairns Campus, School of Medicine, James Cook University, C/- Cairns Base
Hospital, P.O. Box 902, Cairns, Queensland 4870, Australia.
john.mcbridge@jcu.edu.au

An unwell traveller whose itinerary has included Australia may be infected with agents that are uniquely found in that country or by more cosmopolitan agents that exist there. This brief review will discuss some of the more common or important infectious disease diagnoses and discuss some features that can be elicited on examination or from the history that would be useful in directing investigation and treatment. Diseases discussed include epidemic polyarthritis, dengue fever, Murray Valley encephalitis, melioidosis, leptospirosis, Buruli ulcer, scrub typhus and spotted fever.

DOI: 10.1016/j.trstmh.2008.01.006
PMID: 18321549 [PubMed - indexed for MEDLINE]

865. Respirology. 2008 Nov;13(7):937-49. doi: 10.1111/j.1440-1843.2008.01409.x.

Respiratory infections unique to Asia.

Tsang KW(1), File TM Jr.

Author information:
(1)LKS Faculty of Medicine, The University of Hong Kong, Hong Kong, China.
kwttsang@hku.hk

Asia is a highly heterogeneous region with vastly different cultures, social constitutions and populations affected by a wide spectrum of respiratory diseases caused by tropical pathogens. Asian patients with community-acquired pneumonia differ from their Western counterparts in microbiological aetiology, in particular the prominence of Gram-negative organisms, Mycobacterium tuberculosis, Burkholderia pseudomallei and Staphylococcus aureus. In addition, the differences in socioeconomic and health-care infrastructures limit the usefulness of Western management guidelines for pneumonia in Asia. The importance of emerging infectious diseases such as severe acute respiratory syndrome and avian influenza infection remain as close concerns for practising respirologists in Asia.
Specific infections such as melioidosis, dengue haemorrhagic fever, scrub typhus, leptospirosis, salmonellosis, penicilliosis marneffei, malaria, amoebiasis, paragonimiasis, strongyloidiasis, gnathostomiasis, trinchinellosis, schistosomiasis and echinococcosis occur commonly in Asia and manifest with a

prominent respiratory component. Pulmonary eosinophilia, endemic in parts of Asia, could occur with a wide range of tropical infections. Tropical eosinophilia is believed to be a hyper-sensitivity reaction to degenerating microfilariae trapped in the lungs. This article attempts to address the key respiratory issues in these respiratory infections unique to Asia and highlight the important diagnostic and management issues faced by practising respirologists.

DOI: 10.1111/j.1440-1843.2008.01409.x
PMID: 18945321 [PubMed - indexed for MEDLINE]

866. J Microbiol Immunol Infect. 2005 Oct;38(5):343-9.

A retrospective study of hantavirus infection in Kinmen, Taiwan.

Chow L(1), Shu PY, Huang JH, Wang HC, Chang SF, Lu HY, Lin TH.

Author information:
(1)Center for Research and Diagnostics, Center for Disease Control, Department of Health, Taipei, Taiwan.

In this retrospective study, 140 serum samples collected from 85 scrub typhus-negative patients in Kinmen Island in 2000 were tested for antibodies to hantavirus using enzyme-linked immunosorbent assay. Seven patients (8.23%) were confirmed as having hantavirus infection as demonstrated by increased hantavirus-specific immunoglobulin M and/or immunoglobulin G antibodies in their convalescent serum samples. Analysis of indirect immunofluorescence assay showed that Seoul type was the etiologic agent. Serosurvey of rodents caught in the resident township of these hantavirus-infected human cases showed that the seroprevalence of antibodies to hantavirus among Rattus norvegicus, Mus musculus, and R. flavipectus was 50% (4/8), 20% (1/5), and 2% (7/348), respectively. Molecular analysis showed that these reservoir hosts carried a Seoul type hantavirus. To our knowledge, this is the first report demonstrating indigenous hantavirus cases in Kinmen.

PMID: 16211143 [PubMed - indexed for MEDLINE]

867. Indian J Med Microbiol. 2013 Oct-Dec;31(4):343-8. doi: 10.4103/0255-0857.118874.

First case series of emerging Rickettsial neonatal sepsis identified by polymerase chain reaction-based deoxyribonucleic acid sequencing.

Aarthi P(1), Bagyalakshmi R, Mohan KR, Krishna M, Nitin M, Madhavan HN, Kalyani S.

Author information:
(1)Larsen and Toubro Microbiology Research Centre, Kamal Nayan Bajaj Research Centre, Vision Research Foundation, Nungambakkam, Chennai, Tamil Nadu, India.

PURPOSE: To detect and identify the aetiological agent in the peripheral blood from the cases of neonatal sepsis.
MATERIALS AND METHODS: Four neonates from geographically different regions of

South India presented with signs of neonatal sepsis and all the routine clinical
and laboratory investigations were performed. Blood culture by Bac T Alert 3D was
negative. To establish the aetiology, polymerase chain reaction (PCR) for
eubacterial genome and subsequent amplification with Gram positive and Gram
negative primers were performed followed by deoxyribonucleic acid (DNA)
sequencing.
RESULTS: PCR for the detection of eubacterial genome was positive in all the four
neonates and further amplification with designed Gram positive and Gram negative
primers revealed the presence of Gram negative bacteria. The amplicons were
identified as Orientia tsutsugamushi in three neonates and Coxiella burnetti in
the other neonate. Multalin analysis was done to further characterise the strain
variation among the three strains.
CONCLUSION: PCR-based DNA sequencing is a rapid and reliable diagnostic tool to
identify the aetiological agents of neonatal sepsis. This is the first case
series of emerging Rickettsial neonatal sepsis in India .

DOI: 10.4103/0255-0857.118874
PMID: 24064639 [PubMed - indexed for MEDLINE]

868. Rev Infect Dis. 1989 May-Jun;11 Suppl 4:S864-76.

Hemorrhagic fever with renal syndrome in Korea.

Lee HW(1).

Author information:
(1)World Health Organization Collaborating Centre for Virus Reference and
Research, Institute for Viral Diseases, Korea University, Seoul.

Several clinical variants of hemorrhagic fever with renal syndrome (HFRS) are
caused by Hantaan and related viruses. Since 1951, 500-900 patients with HFRS
have been hospitalized annually in Korea. Although HFRS is associated primarily
with rural areas, it is now being recognized as an urban problem and a particular
hazard to laboratory staff using rodents for research. Recently, epidemic
outbreaks of leptospirosis and scrub typhus have occurred during the HFRS season,
leading to confusion in diagnosis. Serologic diagnosis of HFRS is based on the
demonstration of IgM antibodies to Hantaviruses by the indirect fluorescent
antibody technique or enzyme-linked immunosorbent assay. The specific Hantavirus
causing infection can be identified on the basis of titers of plaque-reduction
neutralizing antibody. Results of studies with monoclonal antibodies suggest that
viral subtypes exist for each Hantaviral serotype presently recognized. While
infection with Hantaviruses is known to be a problem of worldwide dimensions,
present evidence indicates that it occurs over a wider area than previously
recognized. Vertical transmission of Hantaan virus in a pregnant woman has been
documented.

PMID: 2568676 [PubMed - indexed for MEDLINE]

869. Sem Hop. 1983 Jul 7;59(27-28):2053-4.

[Rickettsioses. A disease of tourism].

[Article in French]

Edlinger E, Navarro P.

Clinical diagnosis of rickettsial diseases, which are acute infections of variable severity, cannot be done without knowledge of the epidemiologic background. It must be confirmed subsequently by indirect immunofluorescence, which is the choice serologic test. Among the 225 cases of rickettsial disease confirmed between 1980 and 1982, 118 cases of mediterranean spotted fever were acquired in the south of France during the summer. The other cases of spotted fever and all the cases of murine, louse-borne and scrub typhus occurred in tourists who had recently come back from countries where rickettsial morbidity still persists.

PMID: 6312574 [PubMed - indexed for MEDLINE]

870. MMW Munch Med Wochenschr. 1979 Feb 9;121(6):209-12.

[Boutonneuse fever in tourists as a model for clinical diagnosis of rickettsioses (author's transl)].

[Article in German]

Eichenlaub D.

Case histories of boutonneuse fever are described in order to exemplify major characteristics of most rickettsioses: recent travel history, feverish illness with severe headache, skin eruptions and histological findings. Up-to-date informations concerning the epidemiologic situation of typhus, scrub typhus and Rocky Mountain spotted fever are given. The characteristics of Q fever and the possibility of rickettsial laboratory infections are pointed out.

PMID: 104166 [PubMed - indexed for MEDLINE]

871. Ren Fail. 2000 May;22(3):337-43.

Renal failure in vibrio vulnificus infection.

Lerstloompleephunt N(1), Tantawichien T, Sitprija V.

Author information:
(1)Department of Medicine, King Chulalongkorn Memorial Hospital and Queen Saovabha Memorial Institute Bangkok, Thailand.

Vibrio vulnificus infection with septicemia is a life threatening disease in the immunocompromised hosts. Renal involvement has not been documented. We reported herein 8 patients with V. vulnificus septicemia. All were immunocompromised hosts. Four patients had cirrhosis of the liver, 3 were heavy alcohol drinkers and one had systemic lupus erythematosis. Presenting symptomatology included

fever, chills, leg pain and skin rash. Renal failure was observed in 6 patients. Four patients died shortly after admission. Two survived with clinical course of tubular necrosis. Renal failure is therefore common in V. vulnificus infection. This should be brought to attention, and vigorous antibiotic treatment is required. The disease may be confused with leptospirosis, scrub typhus, malaria and other forms of sepsis which also present with renal failure.

PMID: 10843244 [PubMed - indexed for MEDLINE]

872. Clin Infect Dis. 2003 Apr 15;36(8):1067-9. Epub 2003 Apr 4.

Decrease in human immunodeficiency virus type 1 load during acute dengue fever.

Watt G(1), Kantipong P, Jongsakul K.

Author information:
(1)Department of Retrovirology, US Army Medical Component, Armed Forces Research Institute of Medical Sciences, Bangkok, Thailand. wattgh@thai.amedd.army.mil

Rather than the expected increase in human immunodeficiency virus type 1 (HIV-1) load, there was transient suppression of HIV-1 replication during acute dengue infection in a 29-year-old Thai woman. Acute-phase (but not convalescent-phase) serum samples obtained from an HIV-1-uninfected patient with dengue fever reduced HIV-1 infectivity, as determined by a peripheral blood mononuclear cell assay, suggesting the possibility that HIV-1 replication is suppressed during acute dengue fever, as occurs during some cases of scrub typhus infection and measles.

DOI: 10.1086/374600
PMID: 12684921 [PubMed - indexed for MEDLINE]

873. Korean J Parasitol. 1997 Sep;35(3):171-9.

Geographical distribution of vectors and sero-strains of tsutsugamushi disease at mid-south inland of Korea.

Ree HI(1), Lee IY, Jeon SH, Yoshida Y.

Author information:
(1)Department of Parasitology, College of Medicine, Yonsei University, Seoul, Korea.

Studies of geographical distributions and relative population densities of the vector mites of tsutsugamushi disease were carried out in October 1996 at 12 locations of the mid-south inland of the Korean peninsula, where chigger mites have been never studied. Of 177 field rodents and insectivores collected, 154 (87.0%) were Apodemus agrarius. Total 25,707 chigger mites were collected and 14 species were identified, of which Leptotrombidium pallidum was predominant (79.8%) and L. palpale the next (8.9%). L. pallidum, the vector species, was widely distributed in all study areas, showing the highest density at Cho-o 2-dong, Sangju-si (chigger index 201.8), and the lowest at Tanwol-dong. Chungju-si (chigger index 40.7). The other vector species, L. scutellare was

found only at the southern part of the study area such as Yobae and Mipyong, Kumrunggun and Unsu, Kimchon-si. The northernmost areas of the L. scutellare distribution were coincided with the areas where anual mean air temperature is above 10.0 C. Among 157 A. agrarius sera tested, 48.3% was Karp, 1.7% Gilliam and 3.3% Kuroki. The rest of the sera were not able to determine the sero-type because of the cross antigen-antibody reactions among the tested sero-types.

PMID: 9335182 [PubMed - indexed for MEDLINE]

874. Clin Infect Dis. 2002 Jun 15;34(Suppl 4):S145-69.

The past and present threat of rickettsial diseases to military medicine and international public health.

Kelly DJ(1), Richards AL, Temenak J, Strickman D, Dasch GA.

Author information:
(1)Department of Molecular Genetics, Ohio State University, Columbus, OH, USA. daryljkelly@prodigy.net

Morbidity and mortality caused by rickettsioses have had a major influence on military activities and public health for >2000 years. The threat posed by the rickettsioses is reviewed, focusing on the impact and epidemiology of those that have adversely influenced wartime operations and the current challenges posed by these diseases. With their uneven worldwide distribution, the discovery of drug-refractory strains of Orientia tsutsugamushi, the increased threat of their use in acts of bioterrorism, frequent deployment of troops to regions of endemicity, and exposures due to increased humanitarian missions, these diseases continue to be a threat to military personnel in the field. Effective strategies to reduce the impact of these diseases include development of effective vaccines, enhanced surveillance, and development of new safe, effective, and odorless repellants. The continuation of a proven, highly productive military infectious disease research program is essential for providing solutions to these daunting tasks.

DOI: 10.1086/339908
PMID: 12016590 [PubMed - indexed for MEDLINE]

875. Rev Infect Dis. 1991 Sep-Oct;13(5):876-86.

Spotted fever group rickettsial infections in Australia.

Sexton DJ(1), Dwyer B, Kemp R, Graves S.

Author information:
(1)Division of Infectious Diseases, Duke University Medical Center, Durham, North Carolina 27710.

More than four decades ago, Rickettsia australis was discovered to be the etiologic agent of Queensland tick typhus (QTT), yet many unanswered questions persist about the ecology, epidemiology, and clinical features of this disease.

We review 46 previously published cases of QTT along with 16 cases discovered by active surveillance. QTT is usually a mild disease. Patients often have regional lymphadenopathy and eschars. Some have vesicular rashes. Because clinical features overlap, serologic tests are necessary to distinguish QTT from other endemic Australian rickettsial diseases (scrub and murine typhus). Only two tick vectors of R. australis have been identified: Ixodes holocyclus and Ixodes tasmani. Until rickettsiae are isolated from patients in Victoria and Tasmania, it remains unproven that spotted fever group infections in these locations are due to R. australis. However, available serologic, epidemiologic, and clinical data suggest that QTT is not confined to the area in which R. australis was first isolated (Queensland); rather, it occurs along a 3,200-km span of eastern coastal Australia, from tropical to temperate climates.

PMID: 1962102 [PubMed - indexed for MEDLINE]

876. J Med Entomol. 1984 Jan 26;21(1):17-27.

Observations on the ultrastructure and distribution of Rickettsia tsutsugamushi in naturally infected Leptotrombidium (Leptotrombidium) arenicola (Acari: Trombiculidae).

Wright JD, Hastriter MW, Robinson DM.

PMID: 6420571 [PubMed - indexed for MEDLINE]

877. Asian Pac J Trop Med. 2011 Jul;4(7):568-72. doi: 10.1016/S1995-7645(11)60148-X.

Investigation of anaplasmosis in Yiyuan County, Shandong Province, China.

Zhang L(1), Cui F, Wang L, Zhang L, Zhang J, Wang S, Yang S.

Author information:
(1)Department of Rickettsiology and Anaplasmosis, National Institute of Communicable Disease Control and Prevention, China CDC, Beijing 102206, China. zhanglijuan@icdc.cn

OBJECTIVE: To investigate the situation of anaplasmosis in Yiyuan county, Shandong Province.
METHODS: A total of 26 blood samples from febrile patients suspected of anaplasmosis, 48 blood samples from healthy farmers, 8 from dogs, and 10 from goats and 170 ticks were collected in the same area during 2005-2007, and detected by serological and molecular methods.
RESULTS: Eight confirmed cases and 6 probable cases were determined using serologic and molecular methods. The seroprevalence of Anaplasma phagocytophilum (A. phagocytophilum) was 26.7% in healthy cases. Nine out of 10 sheep samples and 7 out of 8 dog samples reacted positively to the A. phagocytophilum antigen. PCR amplification and sequencing of the 16SrRNA of A. phagocytophilum gene showed that some samples from patients, goats and ticks were 100% identical. The seroprevalence of Rickettsia typhi was 22.9%, Orientia tsutsugamushi 6.3%, Rickettsia sibirica 27.1%, Coxiella burnetii 18.8%, Bartonella henselae 31.3%,

and Borrelia burgdorferi 41.6%.
CONCLUSIONS: It is important to make differential diagnosis of febrile patients and to apply treatment with specific antibiotics. It is needed to enforce essential prevention and control measures including tick control and to improve sanitation conditions.

Copyright © 2011 Hainan Medical College. Published by Elsevier B.V. All rights reserved.

DOI: 10.1016/S1995-7645(11)60148-X
PMID: 21803311 [PubMed - indexed for MEDLINE]

878. Jpn J Med Sci Biol. 1994 Jun;47(3):127-39.

Electron microscopic study of the distribution and the vertical transmission of Rickettsia tsutsugamushi in Leptotrombidium pallidum.

Urakami H(1), Takahashi M, Murata M, Tamura A.

Author information:
(1)Niigata College of Pharmacy.

Leptotrombidium pallidum naturally infected with Rickettsia tsutsugamushi was reared and bred in our laboratory for several generations by brother and sister mating. The larvae and adults at the 8th and 9th generations were sectioned and observed by electron microscopy for analysis for the distribution of rickettsiae in the mites. The distribution densities of rickettsiae were markedly different among organs in each mite, but rickettsiae were seen in all the organs and tissues. Rickettsiae were distributed in the highest density in the salivary gland of larvae, and in the salivary gland, excretory bladder, epidermal layer, ovary and testis of adult mites. Only a few rickettsiae were recognized in the muscle of both larvae and adults. On the other hand, we found, in the infected family line used, a significant number of mites in which no rickettsiae were found by electron microscopy. The grouping of rickettsia-positive and -negative mites according to the parent family revealed that the efficiency of vertical transmission of rickettsia was different from one parent family to another. Thus, it became clear that a significant number of rickettsia-negative mites are produced in an infected family line.

PMID: 7823409 [PubMed - indexed for MEDLINE]

879. Zhonghua Liu Xing Bing Xue Za Zhi. 2010 Oct;31(10):1144-7.

[Sero-epidemiologic investigation on tick-borne diseases of humans and domestic animals in Zhejiang province].

[Article in Chinese]

Chai CL(1), Lu QY, Sun JM, Jiang LP, Ling F, Zhang LJ, Zheng SG, Zhang H, Ge JH.

Author information:

(1)Zhejiang Provincial Center for Disease Control and Prevention, Hangzhou
310051, China. chlchai@yahoo.cn

OBJECTIVE: To investigate the seroprevalence of tick-borne diseases in humans and domestic animals from rural areas of Zhejiang province.
METHODS: Anji county, Jindong district and Tiantai county were selected for samples collection according to their geographic locations and historical prevalence of tick-borne diseases. Blood samples of humans and domestic animals were collected in the three sites. An indirect immuno-fluorescent antibody test was used to determine the presence of IgG antibodies of Rickettsiae heilongjiangii, Orientia tsutsugamushi, R. typhi, Anaplasma phagocytosis, Ehrlichia chaffeensis, Bartonella, R. hainan and Coxiella burnetii in these samples.
RESULTS: Six hundred and eighty-three blood samples including 579 from humans and 104 from domestic animals (53 from cattles and 51 from sheep) were collected from the three sites. Antibody positive rates of Orientia tsutsugamushi, R. typhi, Ehrlichia chaffeensis and Coxiella burnetii were significantly different between these sites. IgG from all the 8 pathogens were detected in samples from humans. It was found that the sero-prevalence rates of R. typhi, Bartonella and C. burnetii (20.7%, 10.9%, 5.5%) of adults were higher than those of other Rickettsiae under investigation. The seroprevalence of R. typhi increased along with age. IgG from the 7 pathogens were detected in samples from domestic animals except for Anaplasma phagocytosis. The sero-prevalence rates of R. typhi, Bartonella and R. hainan (69.2%, 51.0%, 22.1%) of adults were higher than those of other Rickettsiae investigated.
CONCLUSION: Tick-borne diseases did spread widely in humans and domestic animals from different rural areas of Zhejiang province. The sero-prevalence rates of R. typhi, B. henselae, R. hainan and C. burnetii were higher than that from other pathogens.

PMID: 21162818 [PubMed - indexed for MEDLINE]

880. Postgrad Med J. 1988 Aug;64(754):614-6.

Rickettsial infection presenting as culture-negative meningitis.

Woo ML(1), Leung JW, French GL.

Author information:
(1)Department of Microbiology, Chinese University of Hong Kong, Prince of Wales Hospital, Shatin, N.T.

Meningitis is an unusual presentation of rickettsial infection. We report a patient who was confirmed by serology to have typhus fever (not scrub), but who presented with the typical findings of culture-negative bacterial meningitis. Neurological manifestations of different types of rickettsial infection are discussed.

PMCID: PMC2428940
PMID: 3249708 [PubMed - indexed for MEDLINE]

881. Jpn J Med Sci Biol. 1964 Jun;17:59-72.

STRAIN VARIATION OF RICKETTSIA ORIENTALIS IN THE COMPLEMENT FIXATION TEST.

SHISHIDO A.

PMID: 14206509 [PubMed - indexed for MEDLINE]

882. Bull Soc Pathol Exot Filiales. 1963 Sep-Oct;56:875-8.

[POSSIBLE ROLE OF RICKETTSIA ORIENTALIS IN THE ETIOLOGY OF "KURU" IN NEW GUINEA AND THE ISLAND OF GUAM].

[Article in French]

LEGAC P, ARQUIE E.

PMID: 14127353 [PubMed - indexed for MEDLINE]

883. J Med Entomol. 1975 Sep 25;12(3):345-8.

Distribution of Rickettsia tsutsugamushi in organs of Leptotrombidium (Leptotrombidium) fletcheri (Prostigmata: Trombiculidae).

Roberts LW, Robinson DM, Rapmund G, Walker JS, Gan E, Ram S.

PMID: 810585 [PubMed - indexed for MEDLINE]

884. Ann Acad Med Singapore. 1997 Sep;26(5):609-15.

Emerging infections in Australia.

Beaman MH(1).

Author information:
(1)University of Western Australia.

Over the last 10 years, novel infectious agents including Equine Morbillivirus, Lyssavirus, Barmah Forest Virus, Rickettsia honei and two as-yet-unnamed bunyaviruses have been identified as causes of human disease in Australia. Previously described agents, such as Japanese B Encephalitis virus, Dengue virus, Ross River virus, Orientia tsutsugamushi, Rickettsia australis, Burkholderia pseudomallei, Mycobacterium ulcerans and Trichinella pseudospiralis, have increased their geographical distribution over the last 20 years. Widespread antibiotic use has also resulted in selection for, and dissemination (especially in hospitals) of, multiresistant bacteria such as multiple-resistant Staphylococcus aureus (MRSA), expanded-spectrum beta-lactamase (ESBL) producing

gram-negative bacteria, penicillin-resistant pneumococci and vancomycin-resistant enterococci (VRE).

PMID: 9494666 [PubMed - indexed for MEDLINE]

885. Microbiol Immunol. 2007;51(4):359-67.

Molecular survey of Babesia microti, Ehrlichia species and Candidatus neoehrlichia mikurensis in wild rodents from Shimane Prefecture, Japan.

Tabara K(1), Arai S, Kawabuchi T, Itagaki A, Ishihara C, Satoh H, Okabe N, Tsuji M.

Author information:
(1)The Shimane Prefectural Institute of Public Health and Environmental Science, Matsue, Shimane, Japan.

Erratum in
Microbiol Immunol. 2012 Jan;56(1):83.

A significant number of patients are diagnosed with "fevers of unknown origin" (FUO) in Shimane Prefecture in Japan where tick-borne diseases are endemic. We conducted molecular surveys for Babesia microti, Ehrlichia species, and Candidatus Neoehrlichia mikurensis in 62 FUO cases and 62 wild rodents from Shimane Prefecture, Japan. PCR using primers specific for the Babesia 18S small-subunit rRNA (rDNA) gene and Anaplasmataceae groESL amplified products from 45% (28/62) and 25.8% (16/62) of captured mice, respectively. Of the 28 18S rDNA PCR positives, 23 and five samples were positive for Hobetsu- and Kobe-type B. microti, respectively. In contrast, of the 16 groESL PCR positives, eight, one and seven samples were positive for Ehrlichia muris, Ehrlichia sp. HF565 and Candidatus N. mikurensis, respectively. Inoculation of selected blood samples into Golden Syrian hamsters indicated the presence of Hobetsu- and Kobe-type B. microti in four and one sample, respectively. Isolation of the latter strain was considered important as previous studies suggested that the distribution of this type was so far confined to Awaji Island in Hyogo Prefecture, where the first case of transfusion-associated human babesiosis originated. DNA samples from 62 FUO human cases tested negative for B. microti 18S rDNA gene, Anaplasmataceae groESL gene, Rickettsia japonica 17K genus-common antigen gene and Orientia tsutsugamushi 56K antigen gene by PCRs. We also conducted seroepidemiological surveys on 62 human sera collected in Shimane Prefecture from the FUO patients who were suspected of carrying tick-borne diseases. However, indirect immunofluorescent antibody tests using B. microti- and E. muris-infected cells detected IgG against E. muris in only a single positive sample. This study demonstrates the presence of several potentially important tick-borne pathogens in Shimane Prefecture and suggests the need for further study on the causative agents of FUOs.

PMID: 17446675 [PubMed - indexed for MEDLINE]

886. Clin Rheumatol. 2009 Jul;28(7):867-8. doi: 10.1007/s10067-009-1166-3. Epub 2009

Mar 25.

Rickettsioses presenting as major joint arthritis and erythema nodosum: description of four patients.

Premaratna R(1), Chandrasena TG, Rajapakse RP, Eremeeva ME, Dasch GA, Bandara NK, de Silva HJ.

Author information:
(1)Department of Medicine, Faculty of Medicine, University of Kelaniya, P.O. Box 6, Thalagolla Road, Ragama, Sri Lanka. ranjan_premaratna@lycos.com

Erythema nodosum and aseptic arthritis are recognized associations of rickettsial infections. However, they usually present with a febrile illness rather than with severe arthritis. We report three patients who presented with incapacitating major joint arthritis and one who presented with severe spondyloarthropathy in addition to major joint arthritis due to serologically confirmed Orientia tsutsugamushi and Rickettsia conorii infections. All of them had erythema nodosum and low-grade fever. They had rapid clinical response to doxycycline.

DOI: 10.1007/s10067-009-1166-3
PMID: 19319622 [PubMed - indexed for MEDLINE]

887. Z Tropenmed Parasitol. 1970 Sep;21(3):313-28.

[Recent results and actual problems in research on Rickettsiae].

[Article in German]

Weyer F.

PMID: 4990719 [PubMed - indexed for MEDLINE]

888. Southeast Asian J Trop Med Public Health. 2002 Dec;33(4):772-9.

Isolation and PCR detection of rickettsiae from clinical and rodent samples in Malaysia.

Tay ST(1), Rohani MY, Ho TM, Devi S.

Author information:
(1)Department of Medical Microbiology, Faculty of Medicine, University of Malaya, Malaysia. tayst@ummc.edu.my

Isolation of rickettsiae from patients' blood samples and organ samples of wild rodents from areas with high seroprevalence of rickettsial infections was attempted using cell culture assay and animal passages. L929 mouse fibroblast cells grown in 24 well tissue culture plate were inoculated with buffy coat of febrile patients and examined for the growth of rickettsiae by Giemsa, Gimenez staining and direct immunofluorescence assay. No rickettsiae were isolated from 48 patients' blood samples. No symptomatic infections were noted in mice or

guinea pigs infected with 50 organ samples of wild rodents. There was no rickettsial DNA amplified from these samples using various PCR detection systems for Orientia tsutsugamushi, typhus and spotted fever group rickettsiae.

PMID: 12757225 [PubMed - indexed for MEDLINE]

889. Clin Diagn Lab Immunol. 2003 Nov;10(6):1059-64.

Detection of antibodies to Anaplasma phagocytophilum and Ehrlichia chaffeensis antigens in sera of Korean patients by western immunoblotting and indirect immunofluorescence assays.

Park JH(1), Heo EJ, Choi KS, Dumler JS, Chae JS.

Author information:
(1)Division of Medical Microbiology, Department of Pathology, The Johns Hopkins Medical Institution, Baltimore, Maryland 21287, USA.

Two hundred seventy one serum samples from South Korean patients were tested to detect antibodies against Anaplasma phagocytophilum (the human granulocytic ehrlichiosis agent) and Ehrlichia chaffeensis (the human monocytic ehrlichiosis agent) by indirect fluorescent-antibody assay (IFA) and the Western blot assay. These sera were collected from patients with symptoms of high fever. The rate of seropositivity for Orientia tsutsugamushi was 50.9% by IFA at the Public Health & Environmental Research Institute and National Institute of Health in South Korea. By IFA, 30 (11.1%) and 39 (14.4%) of the serum samples reacted with A. phagocytophilum and E. chaffeensis antigens, respectively. By the Western blot assays, 24 (8.9%) and 29 (10.7%) of the serum samples reacted with purified A. phagocytophilum and E. chaffeensis protein antigens, respectively. This report strengthens other evidence regarding the presence of A. phagocytophilum and E. chaffeensis infections in humans in South Korea.

PMCID: PMC262439
PMID: 14607867 [PubMed - indexed for MEDLINE]

890. Genome Biol. 2008;9(2):R42. doi: 10.1186/gb-2008-9-2-r42. Epub 2008 Feb 26.

Visualization of pseudogenes in intracellular bacteria reveals the different tracks to gene destruction.

Fuxelius HH(1), Darby AC, Cho NH, Andersson SG.

Author information:
(1)Department of Molecular Evolution, Evolutionary Biology Center, Uppsala University, Norbyvägen 18C, S-752 36 Uppsala, Sweden.
Hans-Henrik.Fuxelius@ebc.uu.se

BACKGROUND: Pseudogenes reveal ancestral gene functions. Some obligate intracellular bacteria, such as Mycobacterium leprae and Rickettsia spp., carry substantial fractions of pseudogenes. Until recently, horizontal gene transfers

were considered to be rare events in obligate host-associated bacteria.
RESULTS: We present a visualization tool that displays the relationships and positions of degraded and partially overlapping gene sequences in multiple genomes. With this tool we explore the origin and deterioration patterns of the Rickettsia pseudogenes and find that variably present genes and pseudogenes tend to have been acquired more recently, are more divergent in sequence, and exhibit a different functional profile compared with genes conserved across all species. Overall, the origin of only one-quarter of the variable genes and pseudogenes can be traced back to the common ancestor of Rickettsia and the outgroup genera Orientia and Wolbachia. These sequences contain only a few disruptive mutations and show a broad functional distribution profile, much like the core genes. The remaining genes and pseudogenes are extensively degraded or solely present in a single species. Their functional profile was heavily biased toward the mobile gene pool and genes for components of the cell wall and the lipopolysaccharide.
CONCLUSION: Reductive evolution of the vertically inherited genomic core accounts for 25% of the predicted genes in the variable segments of the Rickettsia genomes, whereas 75% stems from the flux of the mobile gene pool along with genes for cell surface structures. Thus, most of the variably present genes and pseudogenes in Rickettsia have arisen from recent acquisitions.

DOI: 10.1186/gb-2008-9-2-r42
PMCID: PMC2374718
PMID: 18302730 [PubMed - indexed for MEDLINE]

891. Front Biosci. 2003 Jan 1;8:e197-201.

Rickettsial, ehrlichial and Bartonella infections of the myocardium and pericardium.

Shah SS(1), McGowan JP.

Author information:
(1)Division of Infectious Diseases, Department of Medicine, Bronx-Lebanon Hospital Center, and the Albert Einstein College of Medicine, Bronx, NY 10457, USA. sshah@bronxleb.org

Myocarditis and pericarditis are uncommon complications of human rickettsial, ehrlichial and Bartonella infections. Myocardial inflammation usually occurs in the setting of acute disseminated infection. Organisms associated with myocarditis include: Rickettsia rickettsii, R. conorii, Orientia tsutsugamushi, Coxiella burnetii, Anaplasma phagocytophila (the causative agent of Human Granulocytic Ehrlichiosis) and Bartonella henselae. Pericarditis has been described in the setting of R. conorii and Coxiella burnetii infections. This article reviews the epidemiology, pathologic characteristics, clinical manifestations, diagnosis and treatment of myocarditis and pericarditis caused by these organisms.

PMID: 12456377 [PubMed - indexed for MEDLINE]

892. Am J Trop Med Hyg. 2002 Apr;66(4):431-4.

Evidence of Rickettsia typhi and the potential for murine typhus in Jayapura, Irian Jaya, Indonesia.

Richards AL(1), Rahardjo E, Rusjdi AF, Kelly DJ, Dasch GA, Church CJ, Bangs MJ.

Author information:
(1)U.S. Naval Medical Research Unit No. 2, Jakarta, Indonesia.
RichardsA@nmrc.navy.mil

Murine typhus (etiologic agent: Rickettsia typhi) is endemic to Indonesia, especially on the highly populated island of Java. A survey of rodents from Irian Jaya, the eastern-most province of Indonesia, indicated striking geographic variation in risk factors associated with murine typhus. Murid rodents (n = 112) collected from two villages in the Arso district of northeastern Irian Jaya, were found to be free of ectoparasites normally associated with transmission of R. typhi (i.e., Xenopsylla cheopis). All rodents (n = 72) tested by enzyme-linked immunosorbent assay were negative for antibodies to R. typhi, whereas 12.5% (9/72) were positive for antibodies to Orienta tsutsugamushi. In contrast, both Rattus norvegicus and R. rattles (combined n = 87) from the harbor area of the provincial capital, Jayapura, were infested with flea ectoparasites. X. cheopis was found on 31 (35.6%) of the live-captured rodents. Serum samples from nine of 82 rodents contained antibodies reactive to R. typhi (11.0%). These data show for the first time that rodents exposed to R. typhi are well established in Jayapura, and that some of these rodents harbor fleas potentially capable of transmitting murine typhus and plague.

PMID: 12164301 [PubMed - indexed for MEDLINE]

893. Infect Immun. 1980 Oct;30(1):231-43.

Localization of electron-dense tracers during entry of Rickettsia tsutsugamushi into polymorphonuclear leukocytes.

Rikihisa Y, Ito S.

The invasion of Rickettsia tsutsugamushi, Gilliam strain, into guinea pig polymorphonuclear leukocytes (PMNs) and the localization and distribution of tracers were followed during the process by electron microscopy. The seven tracers used were: cationized ferritin, ferritin, thorium dioxide (ThO2), carbon particles, latex spheres, paraffin oil, and Escherichia coli. These markers were added to the incubation medium containing the PMNs before or simultaneously with R. tsutsugamushi-infected BHK-21 cells. Both morphologically intact and degenerating rickettsiae were present in the phagosomes in PMNs, but only the viable-appearing rickettsiae were free in the cytoplasm. The intact rickettsiae were singly and selectively phagocytized in tightly enclosed phagosomal membranes which usually excluded the tracers, except when ThO2 or ferritin was used. When ThO2, which labels the plasma membrane of PMNs, was used. ThO2-labeled phagosomal membranes enclosing rickettsiae were observed and short membrane fragments still labeled with this tracer were found in the vicinity of rickettsiae in the cytoplasmic matrix of PMNs. When ferritin or ThO2 was used as a tracer, some of the phagosomes contained rickettsiae still enclosed in an envelope of BHK-21 cytoplasm and cell membrane. Phagolysosomes preloaded with electron-dense markers

fused with subsequently formed phagosomes containing degenerated rickettsiae but not with those containing intact rickettsiae. These results support our interpretation that viable rickettsial entry into PMNs is by selective phagocytosis and escape from these phagosomes.

PMCID: PMC551300
PMID: 6777300 [PubMed - indexed for MEDLINE]

894. J Infect Dis. 1980 Jan;141(1):112-8.

From the National Institute of Allergy and Infectious Diseases. Rickettsiology conference.

Philip RN, Paretsky D, Weiss E, Wisseman CL Jr.

PMID: 6154107 [PubMed - indexed for MEDLINE]

895. Trans R Soc Trop Med Hyg. 2009 Sep;103(9):961-3. doi: 10.1016/j.trstmh.2009.04.003. Epub 2009 May 15.

Unusual pancytopenia secondary to haemophagocytosis syndrome in rickettsioses.

Premaratna R(1), Williams HS, Chandrasena TG, Rajapakse RP, Kularatna SA, de Silva HJ.

Author information:
(1)Department of Medicine, Faculty of Medicine, University of Kelaniya, Sri Lanka. ranjan premaratna@lycos.com

We report two patients who presented with a long-lasting febrile illness associated with pancytopenia. Both of them had evidence of hypercellular marrow with haemophagocytosis. They were confirmed as having rickettsial infections by serology and had a rapid haematological recovery with anti-rickettsial antibiotics. We highlight the importance of considering rickettsial infections in patients with such clinical presentations, especially in areas where these infections are endemic or re-emerging. Empirical use of anti-rickettsial antibiotics in such situations could be beneficial, when facilities to diagnose rickettsial diseases are not readily available.

DOI: 10.1016/j.trstmh.2009.04.003
PMID: 19446860 [PubMed - indexed for MEDLINE]

896. Southeast Asian J Trop Med Public Health. 2004 Mar;35(1):113-8.

A new member of the trombiculid mite family Neotrombicula nagayoi (Acari: Trombiculidae) induces human dermatitis.

Takahashi M(1), Misumi H, Urakami H, Saito T, Misumi M, Matsumoto I, Suzuki H.

Author information:
(1)Kawagoe Sogo Senior High School, Kosenba-machi, Kawagoe, Japan.
mamoru0917@hotmail.com

We present the first definitive evidence that the mite Neotrombicula nagayoi bites humans under natural conditions in Japan. Initially, bites resulted in mild pruritus without pain. However, skin reactions increased gradually year by year with severe pruritus with pain being reported by the victim after being bitten repeatedly. Six species of trombiculid mites comprising three genera were isolated from soil samples collected from August to October in both 2001 and 2002 at a study site where a man was bitten by N. nagayoi. The dominant species was L. intermedium (72.4%) followed by L. pallidum (8.3%) and N. nagayoi (8.1%). N. nagayoi was found only in August and September. We did not detect the pathogen Orientia tsutsugamushi in any of the unfed larvae, including N. nagayoi, collected from the soil samples.

PMID: 15272753 [PubMed - indexed for MEDLINE]

897. J Infect Chemother. 2002 Sep;8(3):266-8.

Fulminant Japanese spotted fever definitively diagnosed by the polymerase chain reaction method.

Kodama K(1), Senba T, Yamauchi H, Chikahira Y, Katayama T, Furuya Y, Fujita H, Yamamoto S.

Author information:
(1)Department of Internal Medicine, Hyogo Prefectural Awaji Hospital, 1-6-6 Shimokamo, Sumoto, Hyogo 656-0013, Japan. kodama@awaji-hosp.sumoto.hyogo.jp

A 72-year-old man was admitted to the emergency ward in our hospital on July 20, 2001, because of consciousness disturbance, fever, generalized skin eruption, and severe general weakness beginning 7 days previously. Physical examination on admission revealed marked systemic cyanosis, erythema, and purpura. Laboratory findings indicated disseminated intravascular coagulation (DIC) and multiorgan failure (platelet count, 0.9 x 10(4)/micro l; fibrin degradation product, 110 micro g/ml; C-reactive protein, 22.6 mg/dl). Soluble interleukin 2-receptor (sIL-2R) was markedly increased to 14 710 U/ml. Blood gas analysis demonstrated severe metabolic acidosis. He was diagnosed with multiorgan failure due to DIC. Administration of heparin and sodium bicarbonate was started immediately, but respiratory failure was exacerbated and systemic spasm caused by encephalitis was noted. Although he was supported by an artificial ventilator, deterioration of metabolic acidosis occurred, and the blood pressure decreased to less than 60 mm Hg. He died 5.5 h after admission. The serological test showed no positive antibody titers against Orientia tsutsugamushi, Rickettsia japonica, or Rickettsia typhi. However, a specific DNA band derived from R. japonica was detected by the polymerase chain reaction (PCR) method using a primer from a blood clot. Therefore, he was definitively diagnosed as having Japanese spotted fever. The PCR method may be markedly useful for establishing a definitive diagnosis of Japanese spotted fever during the critical stage.

DOI: 10.1007/s10156-002-0185-7

PMID: 12373493 [PubMed - indexed for MEDLINE]

898. J Immunol. 1956 Jun;76(6):475-88.

Study on the growth of Rickettsiae. I. A tissue culture system for quantitative estimations of Rickettsia tsutsugamushi.

BOZEMAN FM, HOPPS HE, DANAUSKAS JX, JACKSON EB, SMADEL JE.

PMID: 13332249 [PubMed - indexed for MEDLINE]
